# Overcoming the Barrier to Intermolecular Alkoxy Radical Reactivity: Proton-Coupled Electron Transfer-Mediated Alkene Hydroetherification

**DOI:** 10.1021/jacs.6c05777

**Published:** 2026-04-23

**Authors:** Lucien C. Delgutte, Yunkai Hua, Jaeyong Lee, Alexis N. Lugo, Aadarsh R. Iyengar, Daria E. Kim

**Affiliations:** Department of Chemistry, 5718 Vanderbilt University, Nashville, Tennessee 37235, United States

## Abstract

Herein, we report an intermolecular *anti*-Markovnikov hydroetherification initiated through proton-coupled electron transfer (PCET). Though PCET has facilitated formation of alkoxy radicals, accessing the intermolecular reactivity of these intermediates remains challenging due to their inherent instability and kinetic facility of competitive unimolecular transformations such as β-scission and π-cyclization or preemptive reduction through electron transfer. To accelerate intermolecular trapping, nucleophilic olefins such as enoxysilanes and enamides were selected as bimolecular traps. To attenuate the off-target oxidation of these electron-rich alkenes, we designed a novel organophotocatalyst that undergoes stimulus-gated redox activation upon hydrogen bonding with alcohol substrates, deactivating the catalyst outside of these precursor complex associations. This species has enabled efficient intermolecular hydroetherification with alkoxy radical intermediates generated directly from diverse alcohol precursors under mild conditions and with low catalyst loadings. Notably, alcohol substrates susceptible to 1,5-hydrogen atom abstraction, β-scission, and π-cyclization are shown to undergo intermolecular addition. Luminescence titration experiments and reactivity studies were performed to support a hydrogen bond-activated PCET mechanism.

## Graphical abstract

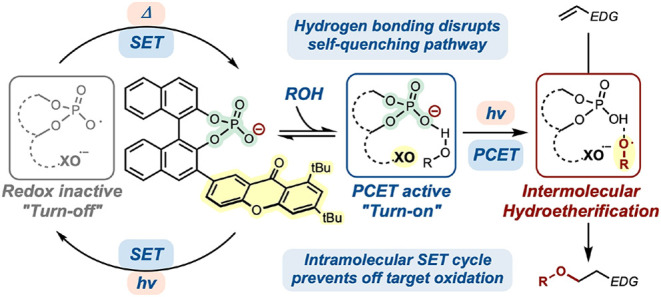

## Introduction

Polar functionalization reactions using alcohols as nucleophiles are ubiquitous throughout synthetic chemistry and biology. In contrast, the reactivity of the analogous open-shell intermediates is less explored.^1^ To address this limitation, several stoichiometric and catalytic activation strategies have been developed to access alkoxy radicals from weak O–X precursors or through the direct engagement of alcohols or alkoxide intermediates (Figure 1A).^2–7^ These studies have enabled reaction development through three elementary processes: β-scission, hydrogen atom abstraction, and addition into olefins^4,6,8–17^ Generally, the thermolysis/photolysis of stoichiometric adducts and ligand-to-metal charge transfer (LMCT) liberate discrete *O*-centered radicals resulting in reactivity that is primarily under substrate control. In the context of LMCT, this enhanced propensity for scission and intramolecular hydrogen atom abstraction has facilitated the development of deracemization reactions, revealed efficient fragmentation pathways to methyl radical intermediates, and enabled remote functionalization.^10–13,18,19^

**Figure 1:**
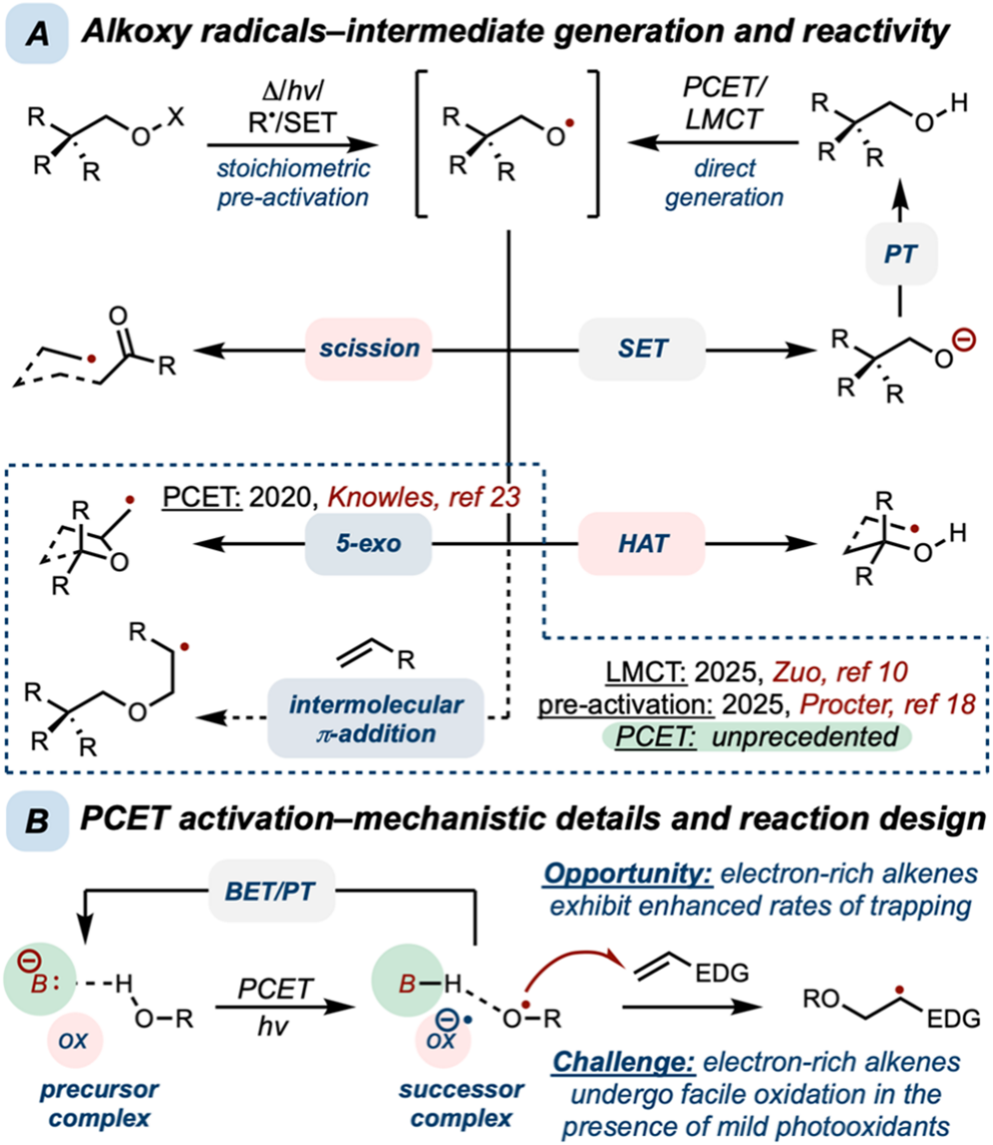
(A) Generation and reaction pathways for alkoxy radicals. (B) Structural requirements for PCET activation.

In contrast, the associative mechanism of proton-coupled electron transfer (PCET) provides unique opportunities for both substrate regiocontrol and intermediate stabilization.^17,20–24^ Specifically, oxidative PCET activation requires hydrogen-bonding association between the substrate and the base in the proximity of an oxidant, termed the precursor complex (Figure 1B).^25–27^ Formal homolysis of the X–H bond yields a successor complex that imparts stabilization to the generated radical intermediate through residual hydrogen bonding to the conjugate acid.^22^ The foundational work of Knowles and co-workers underscored the importance of precursor complex assembly dynamics by demonstrating that strong Coulombic interactions between the photooxidant and the base enhance the efficiency of PCET-initiated reactivity in ternary systems.^28^ Further, they showed that stronger substrate–base association constants can promote preferential substrate activation by contrasting the facility of thiyl and amidyl radical generation.^29^ Beyond these precursor complex effects, the intermediacy of the successor complex has enabled enhanced catalyst control over generated open-shell species, as is evidenced by the high degree of enantioinduction achieved in the context of hydroamidation and reductive PCET processes.^21,22,24,30^ These mechanistic nuances and capabilities motivated us to develop a new organophotocatalyst for the activation of strong O–H bonds in order to facilitate bimolecular hydroetherification, a transformation that remains a challenge within the scope of reported PCET-initiated reactivity.^23^

Intramolecular competition studies performed by Sammis and co-workers demonstrated that electron-rich alkenes such as enoxysilanes exhibit enhanced rates of *O*-centered radical trapping, a phenomenon that is supported by polarity-matching concepts.^31–33^ This property made polarized nucleophilic olefins an appealing starting point for the development of an intermolecular hydroetherification reaction (Figure 1B). While these functional groups display an affinity for electrophilic radicals, they are susceptible to single-electron oxidation in the presence of the photooxidants necessary for PCET.^34–37^ Zuo and co-workers recently developed an LMCT-initiated method for the functionalization of enoxysilanes with alkoxy radicals derived from industrial-grade alcohols.^10^ We anticipated that similar selectivity could be achieved through outer sphere electron transfer via the PCET mechanism. Additionally, we believed that the stabilizing PCET successor complex could provide complementary tolerance for complex alcohols prone to unimolecular decomposition under LMCT conditions.^9,11,13,38,39^ To mitigate competitive oxidation of electron-rich alkenes, we endeavored to design a bifunctional organophotocatalyst scaffold that undergoes redox activation upon precursor complex assembly.^40^

## Results and Discussion

Accordingly, we synthesized **PC1**, a bifunctional catalyst with steric blocking groups incorporated into the xanthone substructure to attenuate photodegradation.^41–44^ While reduction of precursor complex molecularity has been explored as a strategy for facilitating PCET activation, we envisioned that covalent tethering of a photooxidant and base might also prevent competitive single-electron transfer (SET) from alkene coupling partners (Figure 2A).^45–50^ BINOL phosphate possesses an oxidation potential of 1.12 V vs F_c_^+^/F_c_, while the corresponding phosphoric acid is considerably less oxidizable (*E*_*ox*_ = 1.34 V vs F_c_^+^/F_c_).^51^ We reasoned that this effective range of 0.22 V could be used to achieve hydrogen bond-mediated redox activation in the presence of a proximal photooxidant. We selected xanthone, an organic photooxidant that has an excited-state reduction potential (*E*_red_^*^ = 1.19 V vs F_c_^+^/F_c_) between the oxidation potential of BINOL phosphate and BINOL phosphoric acid.^51–53^ Thus, photoinduced electron transfer from the phosphate to the excited-state xanthone is exergonic, while oxidation of the phosphoric acid is not thermodynamically favorable. Further, the modest driving force associated with phosphate oxidation (Δ*G* = −1.61 kcal/mol) suggested that hydrogen-bond acceptor interactions could raise the oxidation potential of the phosphate to shift intramolecular charge transfer into a thermodynamically uphill process. We anticipated that the covalently linked oxidant–base pair would remain functionally redox-inactive in the absence of hydrogen-bond donors (Figure 2B). Upon formation of a hydrogen-bonded precursor complex with an alcohol substrate, however, recovery of the xanthone oxidizing ability would enable the formal homolysis of the associated O–H bond. We envisioned that this structural requirement for redox activation could promote PCET in the presence of readily oxidizable functional groups.

**Figure 2:**
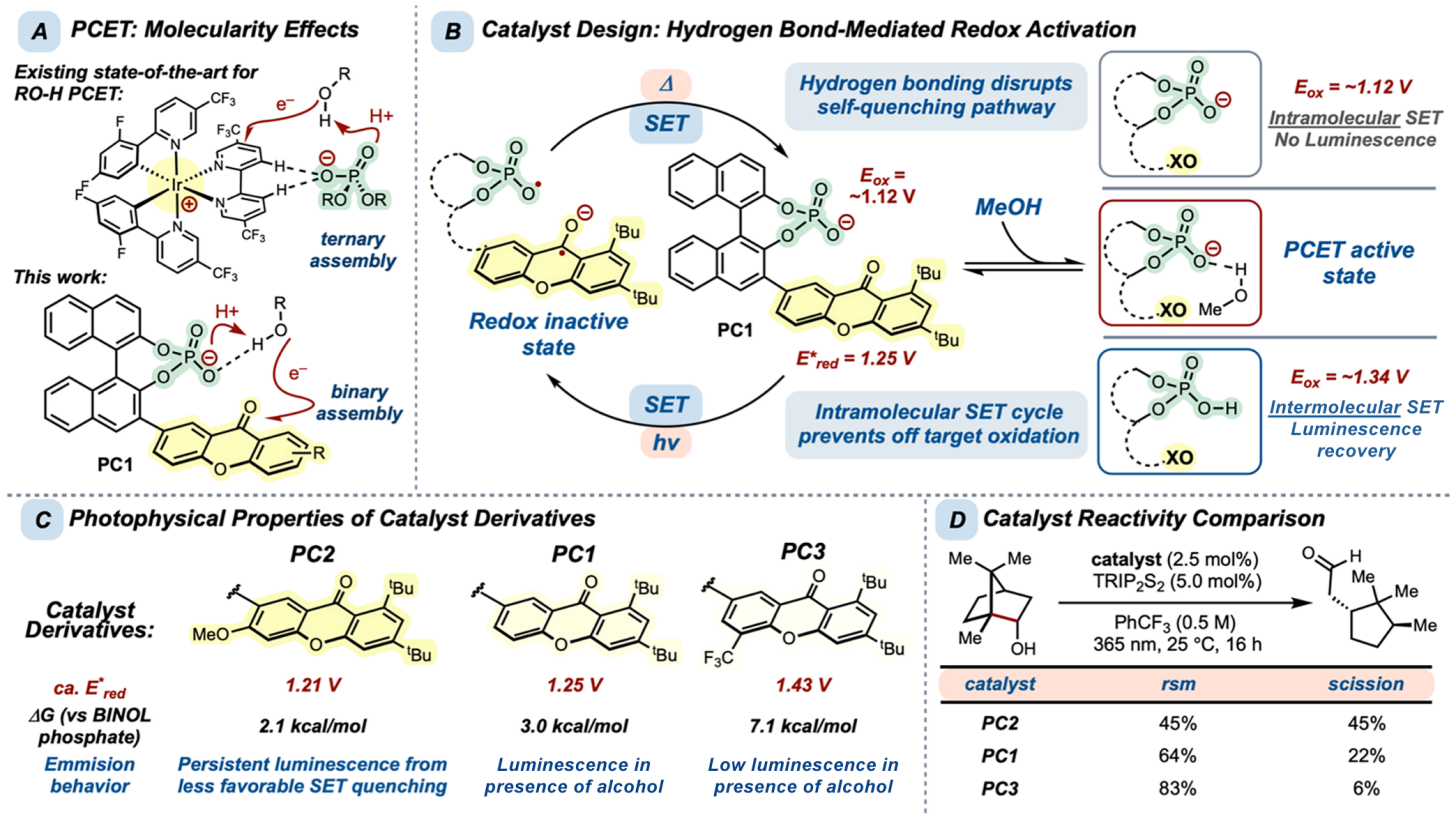
(A) Molecularity effects in precursor complex assembly. (B) Hydrogen bonding as a reversible trigger for catalyst redox activation. (C) Catalyst derivatives and photophysical behavior. (D) Borneol scission reactivity comparison with **PC1**, **PC2**, and **PC3**.

In accordance with the analysis presented above, **PC1** displayed a strong UV absorbance signal with minimal fluorescence emission indicative of self-quenching. In contrast, the parent phosphoric acid (*ca. E*_*ox*_ = 1.34 V vs F_c_^+^/F_c_) of **PC1** produced a strong emission signal.^51^ We derived an excited-state reduction potential (ca. E*_red_ = 1.25 V vs F_c_^+^/F_c_) for **PC1** which is similar to that of xanthone and well within range for BINOL phosphate oxidation (see Supporting Information). Addition of methanol to the catalyst solution resulted in the recovery of fluorescence emission, demonstrating stimulus-gated activation of intermolecular excited-state charge-transfer ability. To further probe the hydrogen bond-dependent fluorescence recovery behavior of our bifunctional catalyst, we synthesized two additional derivatives of **PC1**: **PC2** (*ca. E*_red_^*^ = 1.21 V vs F_c_^+^/F_c_) featuring a methoxy group and **PC3** (*ca. E*_red_^*^ = 1.43 V vs F_c_^+^/F_c_) incorporating a trifluoromethyl group. These species enabled us to sample the excited-state xanthone oxidizing ability below and above **PC1** (Figure 2C). The less oxidizing photocatalyst **PC2** was found to emit in the absence of methanol. The more oxidizing **PC3**, however, exhibited weak baseline emission and required much higher concentrations of alcohol to produce even low levels of fluorescence. Benchmarking the derived catalyst excited-state reduction potentials against the oxidation potentials of BINOL phosphate and BINOL phosphoric acid revealed the following thermodynamic relationships; **PC2** emits because a minor fraction of the xanthone avoids reduction (Δ*G* = −2.08 kcal/mol). **PC1** exhibits reversible fluorescence turn-on behavior as the excited-state charge-transfer event with the phosphate is quite exergonic (Δ*G* = −3.00 kcal/mol), while the oxidation of the corresponding phosphoric acid is endergonic (Δ*G* = +2.08 kcal/mol). Meanwhile, the phosphoric acid derivative of **PC3** exhibits comparatively weak emission due to the retained exergonicity of the charge-transfer event (Δ*G* = −2.08 kcal/mol).

The reactivity of these catalysts was assessed using the *β-*scission reaction of *(−)*-borneol to campholenic aldehyde (Figure 2D).^9^ The resulting product mixtures showed that the least oxidizing photocatalyst (**PC2**) afforded the greatest conversion to product, while the most oxidizing species (**PC3**) generated only trace amounts of aldehyde. This result illustrated that **PC1** can mediate the formation of alkoxy radical intermediates and that reversible self-quenching limits intermolecular oxidation. Further, the poor performance of **PC3** does not support the transiently generated phosphate radical as a viable reactive intermediate. If O**–**H HAT by a phosphate radical intermediate were operative, we would expect to observe increased conversion from more oxidizing xanthone catalysts (*i.e*. **PC3**).^51^ As the inverse trend is operative, we concluded that a PCET mechanism is implicated in this transformation. Thus, we propose that in the absence of hydrogen-bond donors, **PC1** is engaged in rapid and reversible intramolecular SET. Formation of a hydrogen bond between the phosphate and alcohol substrate suppresses self-quenching to enable PCET. Full protonation of **PC1** generates an intermolecular excited-state oxidant.

With this paradigm in mind, we investigated the *anti*-Markovnikov addition of a methoxy radical into model substrate **1a** (Table 1). We selected **PC1** as the catalyst of choice due to its hydrogen bond-mediated redox activation behavior. To this point, although all three catalysts performed well with enamide **1a** (entry 2), we subsequently found that **PC1** provided a broader substrate tolerance for various alcohols and more oxidizable alkenes. Unsurprisingly, maintaining a high concentration of alcohol throughout the duration of the reaction was found to be beneficial, resulting in an optimized superstoichiometric methanol loading of 7.5 equiv relative to the enamide substrate (entry 3). Assessing the effect of additives and solvent revealed that 5 mol % of TRIP_2_S_2_ and trifluorotoluene afforded optimal conversion to product (entries 4 and 5). Evaluation of key controls confirmed that no product formation was observed in the absence of either the catalyst or light (entries 6 and 7). Exclusion of TRIP_2_S_2_ suppressed conversion to the desired product (entry 8, 19% yield). To demonstrate the necessity of a covalently linked catalyst, the “decoupled” catalyst system composed of BINOL phosphate (**BINOL-Phos**) and xanthone (**XO**) yielded only 8% of **1b** (entry 9).^48,49,54^ The poor mass balance of this control experiment suggests that substantial competitive oxidation of the enamide occurs when the catalyst components are not covalently linked. This oxidation primarily led to the formation of the Markovnikov product, in line with predicted SET regioselectivity (see Supporting Information).^40,55^ To further investigate this oxidation, we ran a reaction with just **XO**, which led exclusively to the Markovnikov product.

**Table 1:**
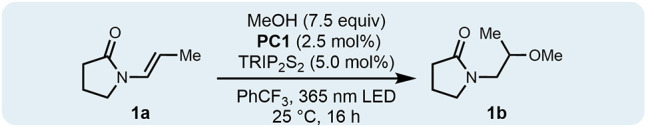

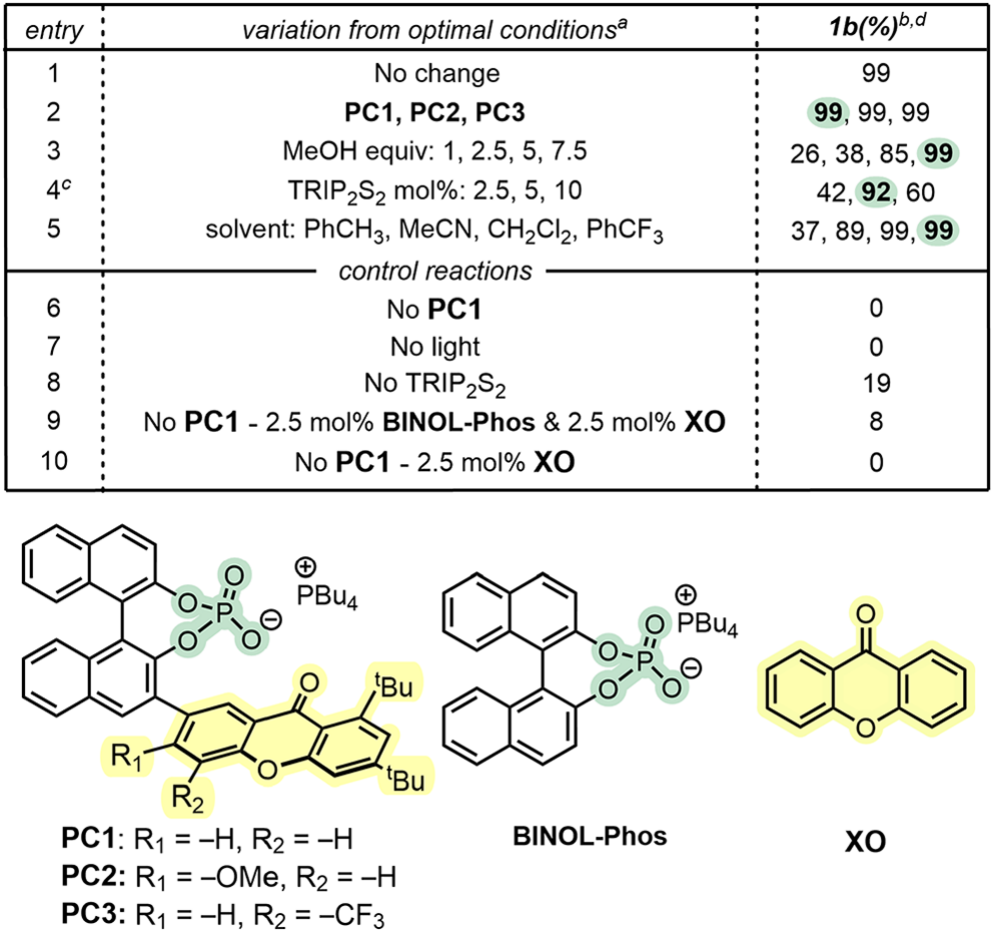
Optimization Table^a^

a **1a** (0.1 mmol), photocatalyst (0.0025 mmol, 2.5 mol %), TRIP_2_S_2_ (0.005 mmol, 5.0 mol %), in trifluorotoluene (PhCF_3_, 200 μL) under nitrogen atmosphere, 16 h, 25 °C, 365 nm LED irradiation.

b Yield was assessed by ^1^H NMR yields of the crude mixture using 1,1,2,2-tetrachloroethane as an internal standard.

c Reactions were stirred for 4 h.

d See Supporting Information for full mass balance.

With these optimized conditions, we began to investigate the scope of the alkene coupling partners with enamide acceptors (Scheme 1). Both di- (**1–2a**, **4–5a**) and trisubstituted enamides (**3a, 6–8a**, **10a**) afforded the exclusively *anti*-Markovnikov product in high yields. Isoindolone-derived substrates susceptible to intermolecular hydrogen atom abstraction (**4–8a**) also performed well. To assess the chemoselectivity of the reaction toward alkene reaction partners containing a competitive hydrogen-bond donor functionality, an enamide with a free N–H (**9a**) was evaluated, resulting in the formation of **9b** in 35% yield. Efficient hydroetherification of estrone-derived substrate (**10a**) demonstrated reaction tolerance toward complex scaffolds. Additionally, a range of more highly oxidizable enoxysilanes were investigated. Enoxysilanes derived from linear aldehydes were well tolerated (**11–12a**, **16–17a**, **19–20a**), as well as substrates with proximal steric bulk (**13a**, **18a**) and derivatives of cyclic ketones (**14–15a**). The compatibility of highly oxidizable enoxysilanes (*E*_1/2_ = 0.80–1.10 V vs F_c_^+^/F_c_) with our reaction conditions underscores the chemoselectivity afforded by the hydrogen bond-gated PCET activation mechanism, as well as the enhanced functional group tolerance imparted by binary precursor complex assembly.^28^ While enamide substrates exhibited complete selectivity for *anti*-Markovnikov products, most enoxysilanes, delivered regioisomeric mixtues of *anti-*Markovnikov products, and mixed ketal/acetal products resulting from Markovnikov addition, with a ratio of approximately 3:2 in favor of the *anti*-Markovnikov products. An exception to this was substrate **14a** which afforded complete selectivity for the *anti-*Markovnikov product, potentially due to a ring strain release driver for radical addition.

**Scheme 1:**
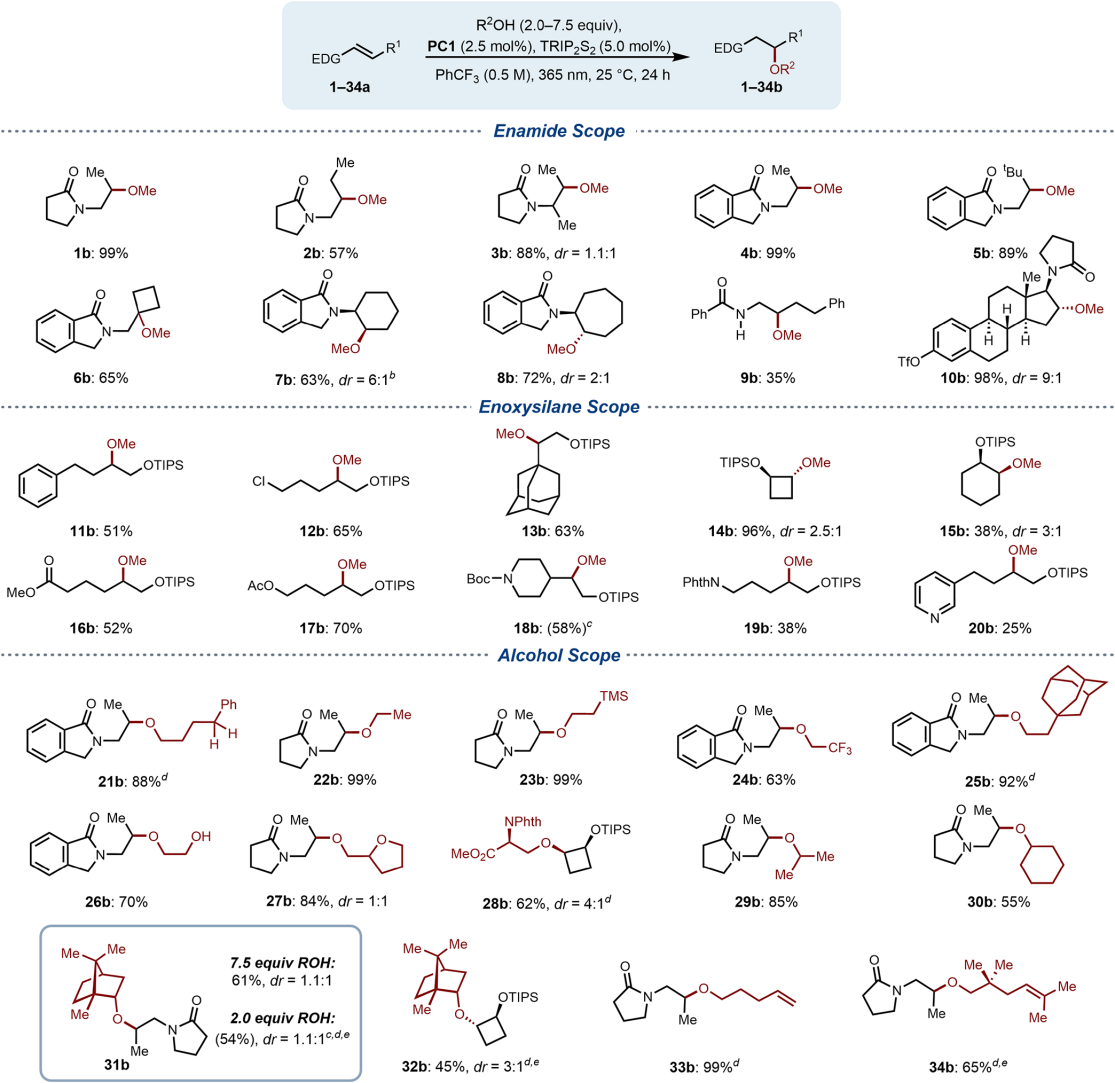
Scope of Intermolecular Hydroetherification Reaction^e^

a Optimized conditions: **1a**–**34a** (0.1 mmol), ROH (0.75 mmol, 7.5 equiv), **PC1** (0.0025 mmol, 2.5 mol %), TRIP_2_S_2_ (0.005 mmol, 5 mol %), in PhCF_3_ (200 μL) under nitrogen, 24 h, 25 °C, 365 nm light irradiation.
b Variation from optimized conditions: **PC1** (0.005 mmol, 5 mol %), TRIP_2_S_2_ (0.010 mmol, 10 mol %), 36 h.
c NMR yield assessed through ^1^H NMR quantification of the crude reaction mixture using 1,1,2,2-tetrachloroethane as an internal standard.
d Variation from optimized conditions: ROH (0.20 mmol).
e Reaction was stirred for 48 h.

At this juncture, we wanted to assess the scope of the reaction with respect to complex alcohols. Specifically, alcohols shown to be susceptible to 1,5-HAT and *β-*scission under complementary activation modes.^6,13,39^ Simple primary (**22–25b**) and secondary (**29–30b**) alcohols afforded hydroetherification products in good yield, accommodating proximal steric bulk as well as electron donating and withdrawing functional groups. Although measurably slower, trifluoroethanol was still converted to hydroetherification product **24b** in a good yield. This is despite the more challenging thermodynamic requirement for the PCET activation of trifluoroethanol (BDE = 107.0 kcal/mol) relative to methanol (BDE = 104.4 kcal/mol), which showcases the catalyst’s versatility with respect to formal O–H bond homolysis across a range of substrates.^56^ Further, subjecting 4-phenyl-1-butanol, a substrate prone to 1,5-HAT via LMCT, resulted in the formation of the intermolecular addition product (**21b**) in 88% yield.^13^ Alcohols potentially susceptible to β-scission were evaluated; notably, a serine derivative and (−)-borneol. These alcohols afforded access to their respective hydroetherification products (**28b**, **31–32b**) in high yields with minimal scission products (<5%) detected in the ^1^H NMR spectra of unpurified reaction mixtures. Finally, several alcohols with pendant alkenes known to undergo 5-*exo*-trig cyclization under existing PCET and LMCT conditions were investigated.^10,23^ When utilized as intermolecular reaction partners, both alcohols afforded efficient conversion to intermolecular addition products **33b** (99%) and **34b** (65%). As we hypothesized that saturation of the catalyst binding site is crucial for inducing redox activity, most industrial alcohols were utilized in a large excess of 7.5 equiv. However, we evaluated the efficiency of hydroetherification with complex alcohols in a modest excess of 2.0–2.5 equiv in the cases of products **21b** (88%), **25b** (92%), **28b** (62%), and **31b** (54%). Quantification of the unpurified reaction mixtures through ^1^H NMR analysis demonstrated almost full retention of the unreacted alcohol starting material in all cases, suggesting that **PC1** is inactivated when alcohol concentration falls below a requisite threshold (see Supporting Information).

To further support a PCET-initiated reaction pathway, we investigated substrates for intramolecular cyclization with unpolarized alkene acceptors (Scheme 2). These alkenes are outside the oxidation range of **PC1** (*E*_red_^*^ = 1.25 V vs F_c_^+^/F_c_) and thus cannot undergo *anti*-Markovnikov addition through SET from the catalyst.^23,36,55^ We discovered that 1,3-diol precursors yielded efficient conversion to their corresponding 5-*exo*-trig cyclization products (**35–38b**), while simple alcohols exhibited comparatively low levels of reactivity. We hypothesized that 1,3-diols (**35–38a**) promote catalyst activation at a lower equivalency through cooperative binding, while monoalcohols require higher concentrations to achieve a comparable degree of catalyst activation. In line with this, cyclization reactions of monoalcohols **39a** and **40a** gave diminished yields with **PC1** (21 and 2%). Subjecting these same substrates to cyclization with **PC2**, a catalyst with a baseline fluorescence emission, significantly increased conversion to product (61 and 36%) despite its lower oxidizing ability. These results mirror the reactivity trend established with our initial (−)-borneol scission studies. Intramolecular reactions utilizing electron-rich radical acceptors (**41–51a**), anticipated to proceed at increased rates or to be subject to additional preorganization effects (**43–45a**), did not require **PC2** to achieve efficient conversion. The oxidizable styrene **39a** and enoxysilane **40a** afforded excellent yields of 68 and 70% yields, respectively. Further surveying the scope of this transformation revealed a tolerance for a variety of enamide nucleophiles (**43–51a**). Notably, substrates with competitive hydrogen-bond donors proved to be competent substrates for the reaction (**43–45a**). The 6-*exo*-trig precursor **49a** afforded the target product in diminished yield, likely due to competitive 1,5-HAT.

**Scheme 2:**
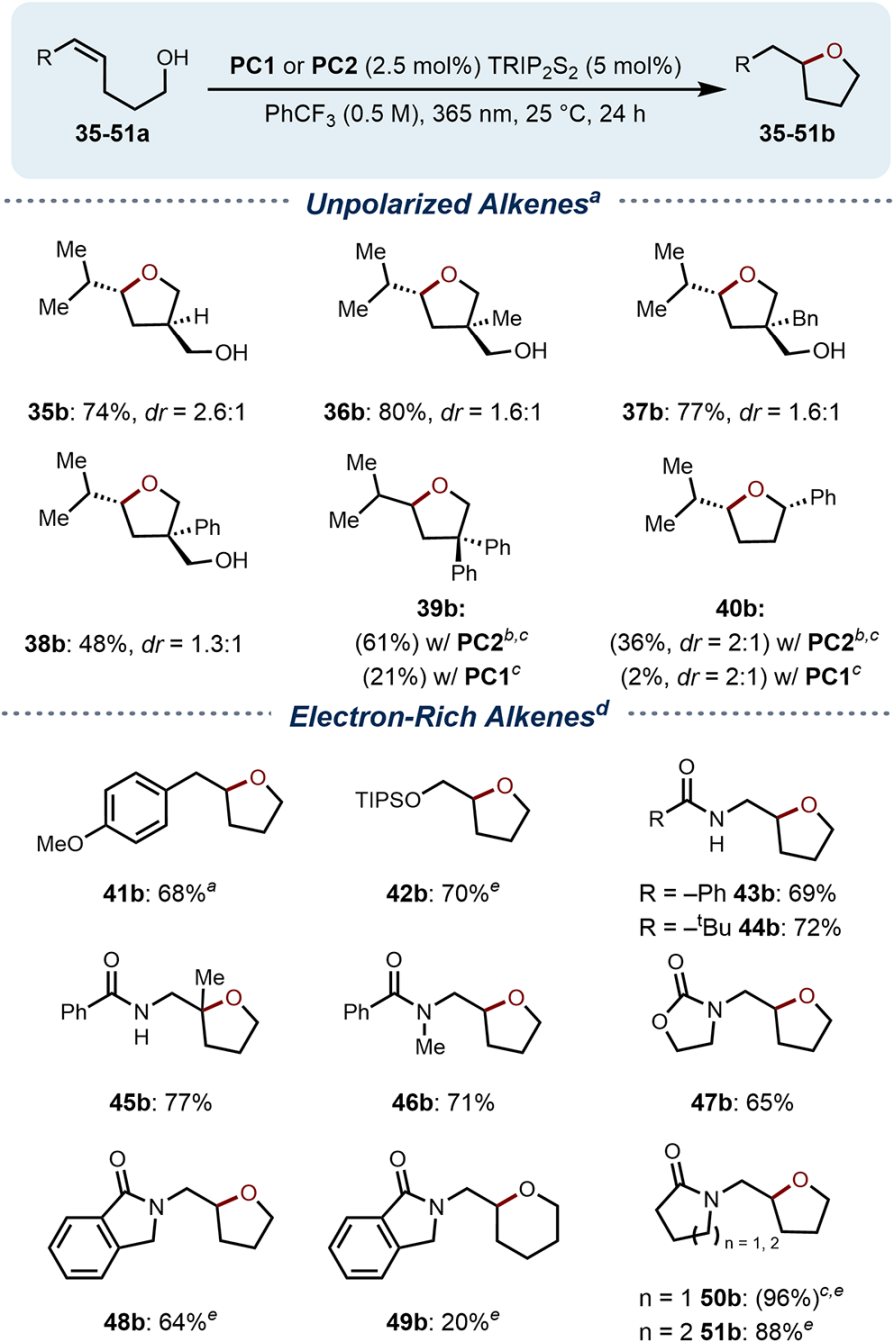
Scope of Intramolecular Hydroetherification^e^

a
**35a**–**51a** (0.1 mmol), **PC1** (0.0025 mmol, 2.5 mol %), TRIP_2_S_2_ (0.005 mmol, 5 mol %) in trifluorotoluene (PhCF_3_, 200 μL) under nitrogen atmosphere, 24 h, 25 °C, under 365 nm light irradiation.
b Variation from standard conditions: **PC2** (0.0025 mmol, 2.5 mol %).
c NMR yield assessed through ^1^H NMR quantification of the crude reaction mixture using 1,1,2,2-tetrachloroethane as an internal standard.
d Variation from standard conditions: TRIP_2_S_2_ (0.0075 mmol, 7.5 mol %), 36 h.
e 24 hour reaction.

To better understand the hydrogen bond-gated redox activation mechanism, we quantified the effect of various alcohol hydrogen-bond donor additives on the emission behavior of **PC1** (Figure 3A, tabulated as *I*/*I*_o_). We performed fluorescence titrations with a series of alcohols. Addition of trifluoroethanol (p*K*_a_ = 23.6) triggered rapid fluorescence recovery, followed by ethylene glycol (p*K*_a_ = 28.4), which is a cooperative hydrogen-bond donor. In contrast, methanol (p*K*_a_ = 29.4) and ethanol (p*K*_a_ = 29.7) required much higher concentrations to achieve enhanced emission. Further, comparing the fluorescence behavior of all three catalysts in the presence of increasing concentrations of methanol revealed that **PC1** was the most responsive to hydrogen-bond donors, **PC3** demonstrated very slow recovery, and **PC2** exhibited modest quenching with a 40:1 ratio of methanol to catalyst (1 equiv at 2.5 mol % loading). Although the quenching of **PC2** is convoluted by an opposing fluorescence enhancement effect, the observed signal dampening relative to the baseline emission of **PC2** is indicative of an alcohol-dependent charge-transfer process.

**Figure 3:**
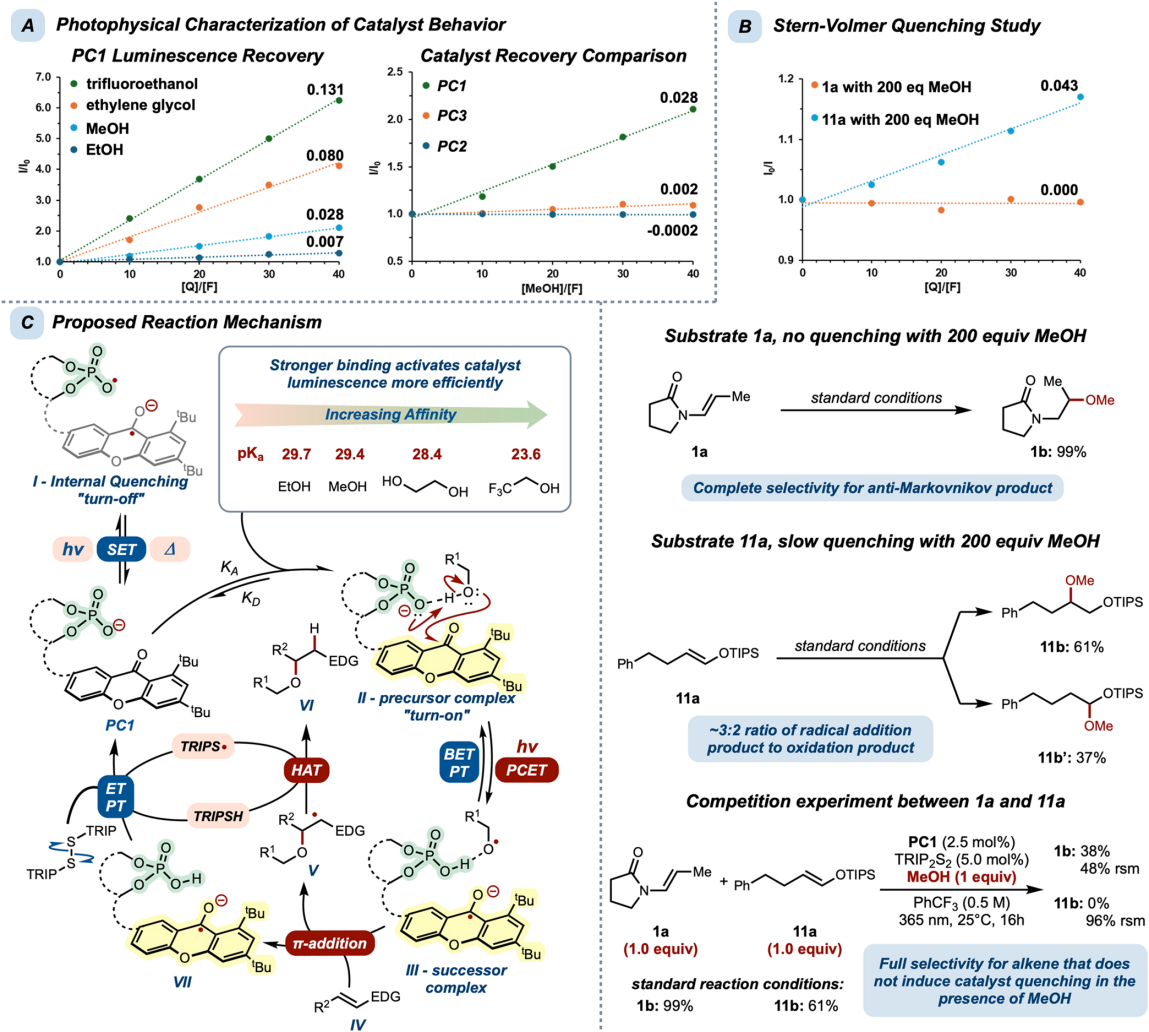
(A) Luminescence recovery studies of various alcohols and catalysts. (B) Stern–Volmer studies and competition experiment of substrates **1a** and **11a** with **PC1**. (C) Proposed PCET mechanism.

Further, Stern–Volmer quenching analysis of **PC1** in the presence of increasing concentrations of **1a** and **11a** confirmed that neither substrate elicits fluorescence quenching in the absence of alcohol (see Supporting Information). With alcohol at reaction-relevant stoichiometry (*ca*. 200:1), enamide **1a** displayed no fluorescence quenching while enoxysilane **11a** exhibited some slow quenching (Figure 3B). These results are in agreement with our observed reaction selectivity with respect to enamide and enoxysilane substrates. Enamide **1a** yielded near-quantitative conversion to the *anti*-Markovnikov addition product **1b**, while enoxysilane **11a** provided the *anti*-Markovnikov (**11b**) and Markovnikov products (**11b′**) in an approximately 3:2 ratio favoring *anti*-Markovnikov addition. To further probe selectivity, a competition experiment between **1a** and **11a** was conducted in the presence of 1 equiv of methanol. The results showed exclusive formation of **1b** with almost complete mass balance recovery of the starting material for both substrates. This experiment suggests that **1a**, a substrate that does not elicit catalyst quenching, exhibits a faster rate of radical addition than **11a**. Thus, differences in product distributions between enamides (**1a–10a**) and enoxysilanes (**11a–20a**) are attributable to slow competitive oxidation of enoxysilanes. While the hydrogen bond-gated fluorescence turn-on behavior of our catalyst system precludes direct observation of **PC1** fluorescence quenching with alcohol, the mechanistic experiments and the reactivity studies outlined, including intramolecular reaction studies with unpolarized alkenes, support a reaction mechanism featuring the intermediacy of an *O*-centered radical generated through PCET.

Based on these results, we propose the following reaction mechanism; in the absence of appropriate hydrogen-bond donors, **PC1** engages in a rapid and reversible intramolecular charge-transfer process under photoirradiation. This charge-transfer transiently generates phosphate radical **I** (Figure 3C). Hydrogen-bonding association between alcohol and catalyst provides a precursor complex (**II**). This association increases the oxidation potential of the phosphate base, thereby preferencing a PCET pathway over intramolecular phosphate oxidation. Upon photoexcitation, **II** undergoes PCET, generating an alkoxy radical intermediate in stabilized successor complex **III**. At this stage, a BET/PT sequence can regenerate **II**, or alternatively, the *O*-centered radical may intercept an alkene coupling partner (**IV**). The resulting *C*-centered radical **V** is then quenched through HAT with thiol formed *in situ* to provide the target hydroetherification product (**VI**). The corresponding thiyl radical then serves as the electron and proton acceptors to regenerate active catalyst **PC1** from **VII**. While the alkene radical acceptors are well within the oxidation range of the xanthone photocatalyst, the hydrogen bond-gated activation of **PC1** mitigates the otherwise thermodynamically favorable SET pathway in the intermolecular case, as evidenced by the high degree of oxidation observed in the “decoupled” catalyst system composed of BINOL phosphate (**BINOL-Phos**) and xanthone (**XO**).

## Conclusion

To conclude, this report describes the design of a new organophotocatalyst for the PCET activation of O–H bonds. The bifunctional structure of **PC1** has afforded access to efficient intermolecular hydroetherification reactions of alkoxy radicals generated directly from alcohol precursors with highly oxidizable π-acceptors. Further, steric occlusion of the xanthone, a photooxidant that typically requires elevated loadings due to its high propensity for photodegradation, has resulted in a catalytic species that is comparable in efficiency to existing transition metal catalyzed systems. While hydrogen bond-mediated catalyst-substrate association is an integral feature of both organocatalysis and PCET, this work, to the best of our knowledge, is the first example of hydrogen bond-mediated redox activation utilized in the context of synthetic methods development. We anticipate that our general catalyst design strategy might be used to achieve substrate selectivity and regiocontrol with respect to complex hydrogen-bond donors and provides an avenue for preferencing challenging PCET processes over thermodynamically favorable SET events.

## Supplementary Material



## Notes

Materials and methods, experimental procedures, mechanistic studies, optimization studies, NMR spectra, and other characterization data (PDF)

## Abbreviations

BET: back electron transfer
HAT: hydrogen atom transfer
LMCT: ligand-to-metal charge transfer
PCET: proton-coupled electron transfer.
