# Supplementary material for: Overcoming the Barrier to Intermolecular Alkoxy Radical Reactivity: Proton-Coupled Electron Transfer-Mediated Alkene Hydroetherification
Source: J Am Chem Soc. 2026 Apr 23;148(17):18540–8. doi: 10.1021/jacs.6c05777 (PMC13154191; doi:10.1021/jacs.6c05777)
Supplement: Supplementary file 1 [file ja6c05777_si_001.pdf]

Overcoming the Barrier to Intermolecular Alkoxy Radical Reactivity: Proton-Coupled Electron Transfer-Mediated Alkene Hydroetherification

Lucien C. Delgutte[†§], Yunkai Hua[†§], Jaeyong Lee[†§], Alexis N. Lugo[§], Aadarsh R. Iyengar[§], and Daria E. Kim*[§]

[†] L.C.D, Y.H., and J.L. contributed equally to this work

[§]Department of Chemistry, Vanderbilt University, 1234 Stevenson Center Lane, Nashville, Tennessee 37240, United States

Email: daria.e.kim@vanderbilt.edu

Table of Contents

A. General Experimental Details ..... - 3 -
B. Optimization of the Reaction Conditions ..... - 6 -
C. Synthesis and characterization of photocatalyst derivatives ..... - 8 -
i) General Procedures ..... - 8 -
ii) NMR Spectra of Catalyst Intermediates ..... - 24 -
iii) Physical Characterization of Catalysts ..... - 56 -
D. Synthesis and Characterization of Intramolecular Substrates ..... - 57 -
i) General Procedures ..... - 57 -
ii) NMR Spectra of Intramolecular Substrates ..... - 73 -
E. Synthesis and Characterization of Intermolecular Substrates ..... - 97 -
i) General Procedures ..... - 97 -
ii) NMR Spectra of Intermolecular Substrates ..... - 105 -
F. Mechanistic Studies ..... - 120 -
i) Crude NMR Quantification ..... - 120 -
ii) Cyclic Voltammetry Measurements ..... - 126 -
iii) Luminescence Experiments ..... - 129 -
iv) Competition Experiments ..... - 138 -
v) Comparison Experiments ..... - 139 -
G. Catalytic Intramolecular Reaction Procedure ..... - 140 -
i) General Procedure ..... - 140 -
ii) NMR Spectra of Intramolecular Hydroetherification Products ..... - 148 -
H. Catalytic Intermolecular Reaction Procedure ..... - 173 -
i) General Procedures ..... - 173 -
ii) NMR Spectra of Intermolecular Hydroetherification Products ..... - 186 -
I. References ..... - 225 -

A. General Experimental Details

General Experimental Procedures: All reactions were performed in flame or oven dried glassware fitted with rubber septa or PTFE/silicone crimp caps under a positive pressure of N_2 or argon utilizing standard Schlenk technique unless otherwise noted. Screening and standard catalytic reactions were performed in ChemGlass® Vials, 4mL (1 Dram), 15x45mm, 13–425 Thread (CG–4904–05). Air and moisture-sensitive liquids were transferred via syringe or stainless-steel cannula through rubber or PTFE/silicone crimp caps. Solids were added under inert gas counter flow or were dissolved in the appropriate solvent. Reactions carried out at temperatures above room temperature were conducted in a preheated oil bath.

All reactions were magnetically stirred and monitored by ^{1}H NMR spectroscopy, gas chromatography/mass spectrometry (GC/MS), or analytical thin-layer chromatography (TLC), using glass-backed plates precoated with silica gel (250 μm thickness, 10–12 μm particle size, 60 Å pore diameter, Supelco 1.05715.001) impregnated with a fluorescent indicator (254 nm). TLC plates were visualized by exposure to ultraviolet light (UV) or were stained by submersion in an acidic solution of *p*–anisaldehyde (PAA), an acidic solution of cerium ammonium molybdate (CAM), or an aqueous potassium permanganate solution ($KMnO_4$) and were developed by heating on a hot plate. Flash column chromatography was performed using Sorbtech® Standard Grade Silica Gel (40–63 μm particle size, 470–530 mesh, 60 Å pore diameter, Sorbtech® #40930H–25) or Sorbtech Alumina Basic, Act 1 (50–200μm particle size, 60 Å pore diameter, Sorbtech® #1564901) in glass columns. Automated column chromatography was performed using a Biotage® Selekt Flash Chromatography System using prepacked Biotage® Sfär C18 D–Duo 100 Å 30 μm cartridges (6–30g) or Biotage® Sfär Silica Duo (60 μm particle size, 5–25g). Purification through preparative thin-layer chromatography was performed using glass-backed plates pre-coated with silica gel (250 μm thickness, 10–12 μm particle size, 60 Å pore diameter, Supelco 1.05715.001) impregnated with a fluorescent indicator (254 nm). The reported yields refer to chromatographically and spectroscopically (^{1}H, ^{13}C, ^{19}F, and ^{31}P NMR) pure material. All ^{1}H NMR yields are corrected through reference to an internal standard. For light irradiation at 390 nm and 427 nm, two Kessil PR160L blue LED lamps at 100% intensity (λ_{max} = 390 nm & 427 nm max 52 W & 45 W respectively) were placed 3 cm away from the reaction vials in a custom-made temperature-controlled LED photoreactor setup. The reactor was cooled with a 75 mm fan. For light irradiation at 340 nm and 365 nm, a Lucent360™ Advanced Photoreactor by Hepatochem (340 nm, HCK1021–01–043, max 54 W, 365 nm, HCK1021–01–012, max 54 W) at 100% intensity was used. The reactor was cooled using a water recirculating chiller (NESLAB RTE–1111) set to 25 °C. A Mikrouna Super (1220/750/900) N_2 filled glovebox was used for catalyst storage, reaction optimization and substrate screening setup.

Materials: Unless noted otherwise, all reagents and starting materials were purchased from commercial sources and used as received (Millipore Sigma, TCI America, CombiBlocks, Ambeed, Oakwood). $CDCl_3$ and DMSO-d_6 were purchased from Millipore Sigma. Tetrahydrofuran (THF), dichloromethane (CH_2Cl_2), toluene (PhMe), acetonitrile (MeCN) and dimethylformamide (DMF) were obtained from Fisher Scientific and purified by successive filtrations through packed columns of neutral alumina or 4 Å molecular sieves under N_2 pressure. Trifluorotoluene ($PhCF_3$), 1,4–dioxane and methanol (MeOH), were obtained as SureSeal™ bottles from Millipore Sigma and were freeze-pump-thaw or sparge degassed with N_2 before use. Solvents for extraction,

crystallization, and flash column chromatography were obtained in ACS grade from Fisher Scientific.

Instrumentation: All NMR spectra were acquired on Bruker Avance III HD 400, 500, or 600 MHz spectrometers. Proton chemical shifts are expressed in parts per million (ppm, δ scale) and are referenced to the residual proton in the NMR solvent ($CDCl_3$: δ 7.26 or DMSO-d_6: δ 2.50). ^{1}H NMR spectroscopic data are reported as follows: chemical shift in ppm (multiplicity, coupling constants *J* (Hz), integration intensity). The multiplicities are abbreviated with s (singlet), d (doublet), t (triplet), q (quartet), p (pentet), sxt (sextet), hept (heptet), m (multiplet), br (broad), with a (apparent) indicated as a modifier where relevant. All ^{13}C spectra recorded are proton-decoupled. The carbon chemical shifts are expressed in parts per million (ppm, δ scale) and are referenced to the carbon resonance of the NMR solvent ($CDCl_3$: δ 77.16 or DMSO-d_6: δ 39.52). ^{13}C NMR spectroscopic data are reported as follows: chemical shift in ppm (multiplicity, coupling constants *J* (Hz)). All ^{19}F spectra recorded are proton-decoupled. The ^{19}F chemical shifts are expressed in parts per million (ppm, δ scale). All raw ".fid" files were processed and analyzed using MestReNOVA 14.3 from Mestrelab Research. High-resolution mass spectra were obtained on a LTQ Orbitrap XL™ Hybrid FT Mass Spectrometer and an Agilent Technologies 6550 Q–TOF LC/MS system using an Agilent Zorbax 300 SB-C3 (2.1 × 150 mm, 5–μm particle size). UV–Vis data was collected using a Hitachi U–3000 spectrophotometer utilizing the UV solutions software (program #: 1344331–15). All samples were measured in absorbance mode with a wavelength range from 250–700 nm and a slit width of 2.0nm. Fluorescence data was obtained using a Varian Cary Eclipse Fluorescence Spectrophotometer. FTIR spectra were acquired from thin-film, neat samples. If required, substances were dissolved in CH_2Cl_2 or Et_2O prior to direct application on the ATR unit. Data are reported as follows: frequency of absorption (cm^{-1}). Melting points were determined on a Mel–Temp® 3.0 capillary system. The reported values are uncorrected. Cyclic voltammograms were collected on a Pine Research WaveDriver 40 DC Bipotentiostat against an Ag/$AgNO_3$ reference electrode in MeCN and calibrated against ferrocenium (F_c^+/F_c). All electrochemical data extracted from existing literature was re-referenced from V vs. SCE to V vs. F_c^+/F_c using the following conversion: $E_{1/2}$ (F_c^+/F_c) = +0.40 V vs. SCE. The MP–8000 Ozone Generator (A2Z Ozone, Inc.) was used for generation of continuous O_3/O_2 flow.

Synthesized compounds:
The following starting materials used in this study were prepared according to the listed references:

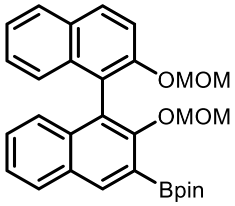

reference 1

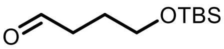

reference 7

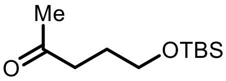

reference 8

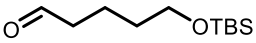

reference 9

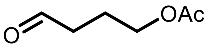

reference 10

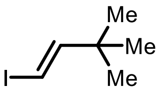

reference 14

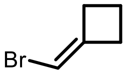

reference 15

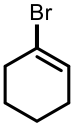

reference 16

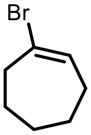

reference 16

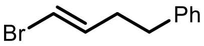

reference 17

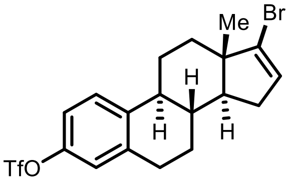

reference 19

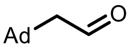

reference 20

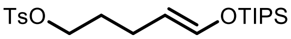

reference 21

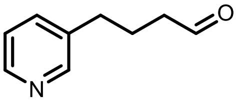

reference 22

Figure SI-1 – Synthesized compounds for substrate and catalyst synthesis

B. Optimization of the Reaction Conditions

Table SI-1 – Intermolecular Enamide Reaction Optimization

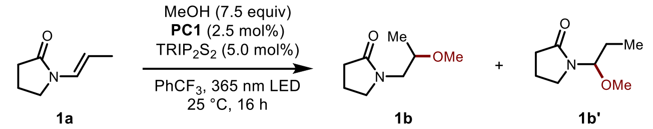

entry	variation from optimal conditions[a]	1b (%)[b]	1b' (%)[b]
1	No change	99	0
2	**SI-PC1, SI-PC2, PC1, 2, 3**	28, 29, **99**, 99, 99	0, 0, 0, 0, 0
3	MeOH equiv: 1, 2.5, 5, 7.5	26, 38, 85, **99**	0, 0, 0, 0
4[c]	$TRIP_2S_2$ mol%: 2.5, 5, 10	42, **92**, 60	0, 0, 0
5	solvent: $PhCH_3$, MeCN, CH_2Cl_2, $PhCF_3$	37, 89, 99, **99**	0, 0, 0, 0
6	wavelength (nm): 340, 365, 390	0, **99**, 91	0, 0, 0
	control reactions		
7	No **PC1**	0	0
8	No $TRIP_2S_2$	19	0
9	No light	0	0
10	No catalyst & no $TRIP_2S_2$	0	0
11	No **PC1** - **Me-PC1**	14	19
12	No **PC1** - 2.5 mol% **BP** & **XO**	8	0
13	No **PC1** - 2.5 mol% **XO**	0	12

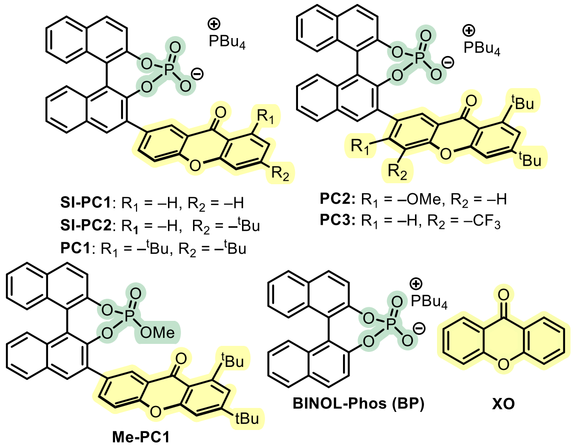

[a]**1a** (0.1 mmol), MeOH (0.75 mmol, 7.5 equiv), **PC1** (0.0025 mmol, 2.5 mol%), $TRIP_2S_2$ (0.0075 mmol, 5.0 mol%), in trifluoromethylbenzene ($PhCF_3$, 200 µL) under nitrogen atmosphere, 16 h, 25 °C, 365 nm LED irradiation. [b]Yield was assessed by 1H NMR yields of the crude mixture using 1,1,2,2-tetrachloroethane as an internal standard. [c]Reaction stirred for 4 h.

Table SI-2 – Intermolecular Enoxysilane Reaction Optimization

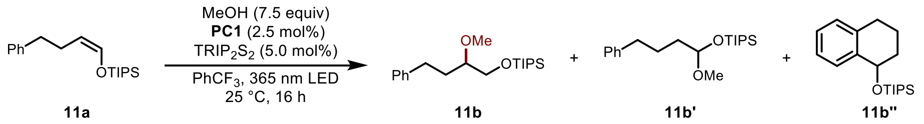

entry	variation from optimal conditions[a]	11b (%)[b]	11b' or 11b'' (%)[b,c]
1	No change	61	37
2	**SI-PC1, SI-PC2, PC1, 2, 3**	36, 41, **61**, 62, 7	18, 21, 37, 35, 2
3	MeOH equiv: 1, 2.5, 5, 7.5	26, 47, 49, **61**	15, 28, 28, 37
4[d]	$TRIP_2S_2$ mol%: 2.5, 5, 10	43, **51**, 35	26, 37, 20
5	solvent: $PhCH_3$, MeCN, CH_2Cl_2, $PhCF_3$	17, 47, 53, **61**	11, 21, 31, 37
6	wavelength (nm): 340, 365, 390	36, **61**, 46	22, 37, 28
	control reactions		
7	No **PC1**	0	20
8	No $TRIP_2S_2$	<5	27
9	No light	0	0
10	No **PC1** & No $TRIP_2S_2$	0	0
11	No **PC1** - **Me-PC1**	19	81
12	No **PC1** - 2.5 mol% **BP** & 2.5 mol% **XO**	<5	30

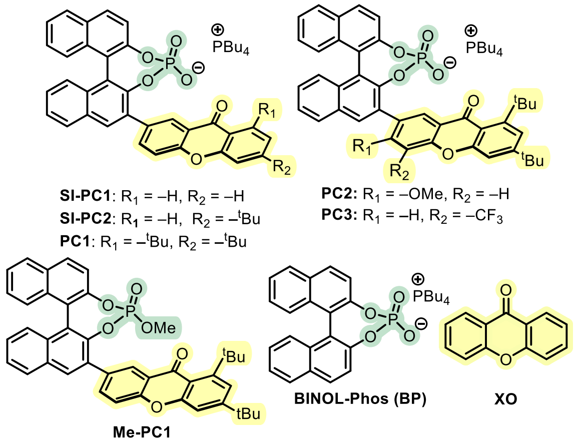

[a]**11a** (0.1 mmol), MeOH (0.75 mmol, 7.5 equiv), **PC1** (0.0025 mmol, 2.5 mol%), $TRIP_2S_2$ (0.005 mmol, 5.0 mol%), in trifluoromethylbenzene ($PhCF_3$, 200 µL) under nitrogen atmosphere, 16 h, 25 °C, 365 nm LED irradiation. [b]Yield was assessed by 1H NMR yields of the crude mixture using 1,1,2,2-tetrachloroethane as an internal standard. [c]Mixture of two Markovnikov addition products. [d]Reaction stirred for 4 h.

Table SI-3 –Intramolecular Reaction Optimization

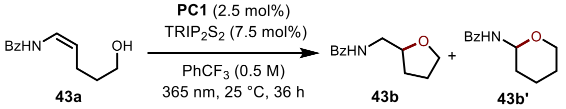

entry	*variation from optimal conditions*[a]	***43b** (%)*[b]	***43b'** (%)*[b]
1	No change	**91**	9
2[c]	**SI-PC1, SI-PC2, PC1**	80, 66, **88**	8, 9, 9
3[d]	$TRIP_2S_2$ mol%: 2.5, 5, 7.5, 10	78, 81, **88**, 88	7, 7, 7, 9
4	solvent: $PhCH_3$, MeCN, CH_2Cl_2, $PhCF_3$	87, 88, 87, **91**	11, 11, 7, 9
5	wavelength (nm): 340, 365, 390	60, **91**, 78	10, 9, 10
	control reactions[d]		
6	No **PC1**	0	96
7	No light	0	0
8	No $TRIP_2S_2$	0	71
9	No **PC1** & No $TRIP_2S_2$	0	99
11	No **PC1** - 2.5 mol% **BP** & 2.5 mol% **XO**	31	47
12	No **PC1** - 2.5 mol% of **Me-PC1**	33	58

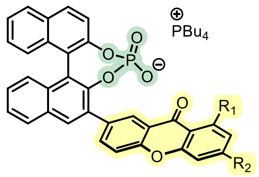

SI-PC1: R_1 = –H, R_2 = –H
SI-PC2: R_1 = –H, R_2 = –^{t}Bu
PC1: R_1 = –^{t}Bu, R_2 = –^{t}Bu

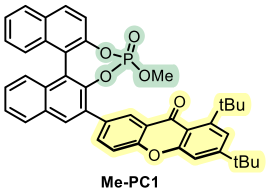

Me-PC1

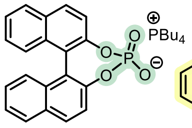

BINOL-Phos (BP)

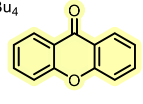

XO

[a]**43a** (0.1 mmol), **PC1** (0.0025 mmol, 2.5 mol%), $TRIP_2S_2$ (0.0075 mmol, 5.0 mol%), in trifluoromethylbenzene ($PhCF_3$, 200 µL) under nitrogen atmosphere, 16 h, 25 °C, 365 nm LED irradiation. [b]Yield was assessed by 1H NMR yields of the crude mixture using 1,1,2,2-tetrachloroethane as an internal standard. [c]**43a** (0.1 mmol), **photocatalyst** (0.0075 mmol, 7.5 mol%), $TRIP_2S_2$ (0.020 mmol, 20 mol%), acetonitrile (MeCN, 200 µL) under nitrogen atmosphere, 36 h, 25 °C, 365 nm LED irradiation. [d]Reaction stirred for 24 h.

C. Synthesis and characterization of photocatalyst derivatives

i) General Procedures

General Procedure C1: Synthesis of xanthone precursor aldehydes S1-S4 via S_NAr

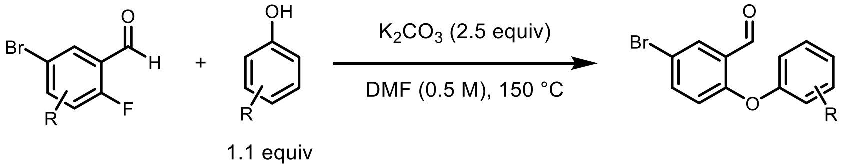

An oven-dried 50 mL round-bottomed flask was equipped with a magnetic stir bar, charged with 5-bromo-2-fluorobenzaldehyde (1.0 equiv), the respective phenol (1.1 equiv), and K_2CO_3 (2.5 equiv). The reaction flask was equipped with a reflux condenser with a greased ground glass joint, after which the reaction apparatus was sealed with a rubber septum, evacuated and backfilled with nitrogen three times and placed under a nitrogen atmosphere. The solid mixture was then dissolved in anhydrous DMF (0.5 M). The joint of the reflux condenser was reinforced with Teflon tape, and the reaction mixture was heated to 150 °C by transferring the reaction apparatus to a pre-heated oil bath. The reaction was allowed to stir for 2 hours at this temperature or until completion (as monitored by TLC).

Upon full consumption of 5-bromo-2-fluorobenzaldehyde, the reaction was removed from the oil bath and allowed to cool to room temperature. The reaction mixture was then diluted with H_2O (40 mL) and transferred to a separatory funnel. The aqueous layer was extracted with EtOAc (3 × 60 mL). The combined organic layer was dried over anhydrous Na_2SO_4 and concentrated under reduced pressure to provide a crude white solid. Purification by flash column chromatography on silica gel or recrystallization in EtOH provided the S_NAr product.

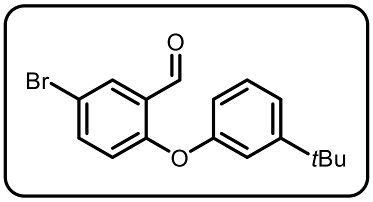

5-bromo-2-(3-(tert-butyl)phenoxy)benzaldehyde (S1)

S1 was prepared using general procedure **C1** using 5-bromo-2-fluorobenzaldehyde (11.7 mL, 98.5 mmol, 1.0 equiv), 3-(*tert*-butyl)phenol (16.3 g, 108 mmol, 1.1 equiv) and K_2CO_3 (34.0 g, 246 mmol, 2.5 equiv) at 150 °C. Following this, the crude product was purified by silica gel chromatography (hexanes/Et_2O 100/0 to hexanes/Et_2O 85/15) to afford **S1** in 66% yield (21.7 g) as a white needle crystal.

IR (Diamond-ATR, neat) ṽ (cm^{-1}): 2960, 2866, 1662, 1586, 1466, 1435, 782.

^{1}H NMR (600 MHz, $CDCl_3$): δ 10.48 (s, 1H), 8.03 (d, *J* = 2.6 Hz, 1H), 7.57 (dd, *J* = 8.8, 2.6 Hz, 1H), 7.33 (at, *J* = 8.0 Hz, 1H), 7.26 (d, *J* = 8.5, 1H), 7.13 (at, *J* = 2.2 Hz, 1H), 6.85 (dt, *J* = 8.0, 1.8 Hz, 1H), 6.78 (d, *J* = 8.8 Hz, 1H), 1.33 (s, 9H).

^{13}C NMR (151 MHz, $CDCl_3$): δ 188.1, 159.4, 155.5, 154.3, 138.3, 131.0, 129.7, 127.7, 122.0, 119.8, 117.0, 116.5, 115.8, 34.9, 31.3.

HRMS (ESI): m/z: $[M+H]^+$ calc'd for $C_{17}H_{18}BrO_2^+$: 333.0490. Found: 333.0493.

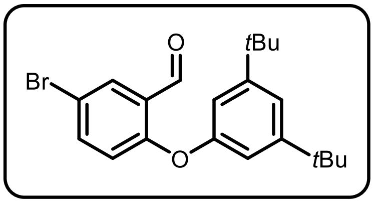

5-bromo-2-(3,5-di-tert-butylphenoxy)benzaldehyde (S2)

S2 was prepared using the general procedure **C1** using 5-bromo-2-fluorobenzaldehyde (5.85 mL, 49.2 mmol, 1.0 equiv), 3,5-di-*tert*-butylphenol (11.2 g, 54.2 mmol, 1.1 equiv) and K_2CO_3 (17.0 g, 123 mmol, 2.5 equiv) at 150 °C. Following this, the crude product was

purified by recrystallization from EtOH to afford **S2** in 52% yield (10.1 g) as a yellow needle crystal.
IR (Diamond-ATR, neat) ṽ (cm^{-1}): 2955, 2869, 1966, 1686, 1587, 1468, 819.
Melting Point: 125-130 °C.
^{1}H NMR (600 MHz, $CDCl_3$): δ 10.50 (s, 1H), 8.03 (d, *J* = 2.6 Hz, 1H), 7.56 (dd, *J* = 8.9, 2.6 Hz, 1H), 7.27 (s, 1H), 6.91 (d, *J* = 1.7 Hz, 2H), 6.77 (d, *J* = 8.9 Hz, 1H), 1.32 (s, 18H).
^{13}C NMR (151 MHz, $CDCl_3$) δ 188.4, 159.9, 155.1, 153.6, 138.4, 131.1, 127.6, 119.5, 119.1, 115.5, 114.3, 35.2, 31.5.
HRMS (ESI): m/z: $[M+H]^+$ calc'd for $C_{21}H_{26}BrO_2^+$: 389.1116. Found: 389.0928.

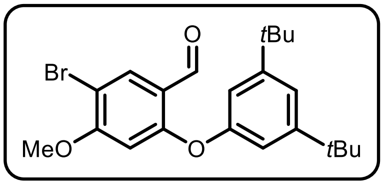

5-bromo-2-(3,5-di-tert-butylphenoxy)-4 methoxybenzaldehyde (S3)

S3 was prepared using the general procedure **C1** using 2-fluoro-4-methoxybenzaldehyde (2.33 g, 10.0 mmol, 1.0 equiv), 3,5-di-*tert*-butylphenol (2.26 g, 11 mmol, 1.1 equiv) and K_2CO_3 (3.45 g, 25 mmol, 2.5 equiv) at 150 °C. Following this, the crude product was purified by silica gel chromatography (hexanes/Et_2O 100/0 to hexanes/Et_2O 85/15) to afford **S3** in 59% yield (2.48 g) as a white amorphous solid.
IR (Diamond-ATR, neat) ṽ (cm^{-1}): 2963, 2866, 1678, 1595, 1271, 1141, 804, 707.
^{1}H NMR (600 MHz, $CDCl_3$): δ 10.34 (s, 1H), 8.12 (s, 1H), 6.93 (d, *J* = 1.7 Hz, 2H), 6.37 (s, 1H), 3.75 (s, 3H), 1.32 (s, 18H).
^{13}C NMR (151 MHz, $CDCl_3$) δ 187.2, 161.6, 161.5, 155.3, 153.5, 132.9, 121.1, 118.8, 113.9, 106.1, 101.1, 56.7, 35.3, 31.5.
HRMS (ESI): m/z: $[M+H]^+$ calc'd for $C_{22}H_{28}BrO3^+$: 419.1222. Found: 419.1194.

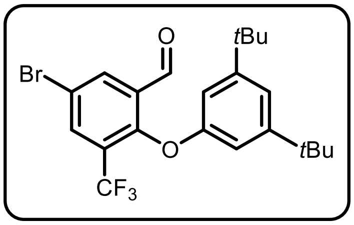

5-bromo-2-(3,5-di-tert-butylphenoxy)-3-(trifluoromethyl) benzaldehyde (S4)

S4 was prepared using the general procedure **C1** using 5-bromo-2-fluorobenzaldehyde (3.50 mg, 12.9 mmol, 1.0 equiv), 3,5-di-*tert*-butylphenol (2.93 g, 14.2 mmol, 1.1 equiv) and K_2CO_3 (4.46 g, 32.3 mmol, 2.5 equiv) at 150 °C. Following this, the crude product was purified by silica gel chromatography (hexanes/Et_2O 100/0 to hexanes/Et_2O 85/15) to afford **S4** in 90% yield (5.30 g) as a white amorphous solid.
IR (Diamond-ATR, neat) ṽ (cm^{-1}): 2963, 2931, 1457, 1319, 1236, 1151.
^{1}H NMR (600 MHz, $CDCl_3$): 9.99 (s, 1H), 8.23 (d, *J* = 2.6 Hz, 1H), 8.07 (d, *J* = 2.6 Hz, 1H), 7.12 (t, *J* = 1.6 Hz, 1H), 6.64 (d, *J* = 1.6 Hz, 2H), 1.24 (s, 18H).
^{13}C NMR (151 MHz, $CDCl_3$): δ 186.3, 160.5, 154.6, 153.5, 135.9 (q, $^3J_{C-F}$ = 5.0 Hz), 135.5, 132.0, 127.5 (q, $^2J_{C-F}$ = 32.3 Hz), 121.9 (q, $^1J_{C-F}$ = 274.2 Hz), 118.6, 117.6, 109.8, 35.2, 31.4.
^{19}F NMR (376 MHz, $CDCl_3$): δ -62.5
HRMS (ESI): m/z: $[M+H]^+$ calc'd for $C_{22}H_{25}BrF_3O_2^+$: 457.09901. Found: 457.0940.

General Procedure C2: Synthesis of xanthones S5-S8 via Pinnick oxidation and Friedel-Crafts reaction

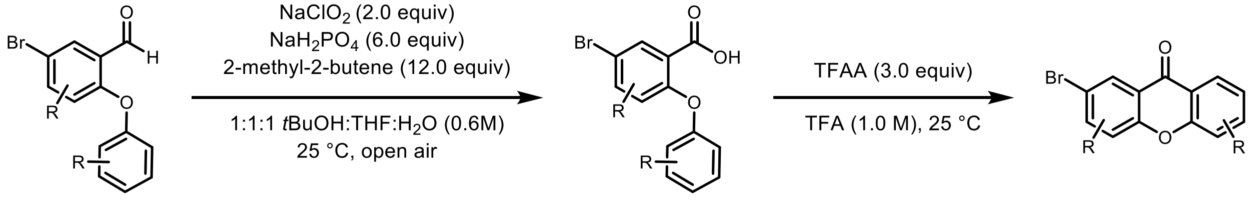

An oven-dried 100 mL round-bottomed flask was equipped with a magnetic stir bar and charged with aldehyde (1.0 equiv) and NaH_2PO_4 (6.0 equiv). Following these solid additions, the reaction was placed under air atmosphere and lightly covered with a rubber septum. The solid was then dissolved in ^tBuOH (0.6 M) and anhydrous THF (0.6 M). 2-methyl-2-butene (12.0 equiv) was then added to the reaction neat in a single portion via syringe transfer. An additional flame-dried round-bottomed flask was charged with solid $NaClO_2$ (2.0 equiv), after which the flask was placed under air atmosphere and covered with a rubber septum. The solid was then dissolved in H_2O (0.6 M) and the resulting colorless solution was added to the reaction flask containing the aldehyde substrate dropwise via syringe over the course of 5 minutes. The reaction mixture was then allowed to stir at room temperature for 12 hours.

After stirring at room temperature for 12 hours, the reaction was diluted with Et_2O (50 mL) and H_2O (100 mL). The layers were separated and the aqueous layer was extracted with Et_2O (3 × 50 mL). The combined organic layers were washed with brine (100 mL), dried over anhydrous Na_2SO_4 and concentrated under reduced pressure by rotary evaporation to provide a crude pale-yellow oil which was carried forward directly into the next step without further purification.

An oven-dried 50 mL round-bottomed flask was equipped with a magnetic stir bar and charged with the crude carboxylic acid (1.0 equiv), after which the flask was sealed with a rubber septum, evacuated and backfilled with nitrogen three times and placed under a nitrogen atmosphere. The solid was then dissolved in 2,2,2-trifluoroacetic acid (1.0 M). 2,2,2-trifluoroacetic anhydride (3.0 equiv) was then added to the reaction dropwise via syringe over a course of 5 minutes. The resulting reaction mixture was then allowed to stir at room temperature for 12 hours.

After stirring at room temperature for 12 hours, the reaction was slowly poured into a large Erlenmeyer flask containing saturated aqueous sodium bicarbonate solution. Excess saturated aqueous sodium bicarbonate solution was added until the solution became slightly basic (pH 8), resulting in the precipitation of a brown solid. The precipitate was collected via vacuum filtration to provide a crude amorphous brown solid. Purification by flash column chromatography on silica gel afforded the target xanthone intermediate.

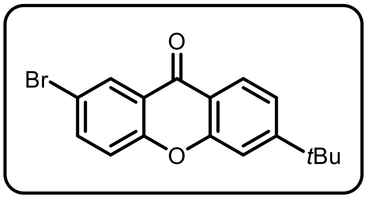

2-bromo-6-(tert-butyl)-9H-xanthen-9-one (S5)

5-bromo-2-(3-(*tert*-butyl)phenoxy)benzoic acid was prepared via general procedure **C2** using **S1** (21.7 g, 65.1 mmol, 1.0 equiv), NaH_2PO_4 (46.8 g, 391 mmol, 6.0 equiv), 2-methyl-2-butene (82.8 mL, 781 mmol, 12.0 equiv) dissolved in ^tBuOH (36.2 mL, 0.6 M), THF (36.2 mL, 0.6 M) and $NaClO_2$ (11.7 g, 130 mmol, 2.0 equiv) dissolved in H_2O (36.2 mL, 0.6 M) at room temperature for 12 hours to provide crude 5-bromo-2-(3-(*tert*-butyl)phenoxy)benzoic acid.

S5 was prepared via general procedure **C2** using crude 5-bromo-2-(3-(*tert*-butyl)phenoxy)benzoic acid (698 mg, 2.0 mmol, 1.0 equiv) and 2,2,2-trifluoroacetic anhydride (835 μL, 6.0 mmol, 3.0

equiv) in 2,2,2-trifluoroacetic acid (2.10 mL, 1.0 M) at room temperature for 12 hours. Following this, the crude solid was purified by vacuum filtration followed by flash column chromatography on silica gel (hexanes/EtOAc 85/15) to afford 2-bromo-6-(*tert*-butyl)-9H-xanthen-9-one as a white amorphous solid in 64% yield (425 mg).

IR (Diamond-ATR, neat) ṽ (cm^{-1}): 3851, 3743, 3733, 3674, 3647, 2962, 2363, 1653, 1635, 1622, 1602, 781.

^{1}H NMR (600 MHz, $CDCl_3$): δ 8.45 (d, *J* = 2.5 Hz, 1H), 8.24 (d, *J* = 8.4 Hz, 1H), 7.78 (dd, *J* = 8.9, 2.5 Hz, 1H), 7.48 (d, *J* = 1.7 Hz, 1H), 7.45 (dd, *J* = 8.4, 1.7 Hz, 1H), 7.39 (d, *J* = 8.8 Hz, 1H), 1.40 (s, 9H).

^{13}C NMR (151 MHz, $CDCl_3$): δ 175.8, 159.9, 156.2, 155.0, 137.4, 129.2, 126.4, 123.2, 122.3, 119.9, 119.2, 116.9, 114.4, 35.6, 30.9.

HRMS (ESI): m/z: $[M+H]^+$ calc'd for $C_{17}H_{16}BrO_2^+$: 331.0334. Found: 331.0317.

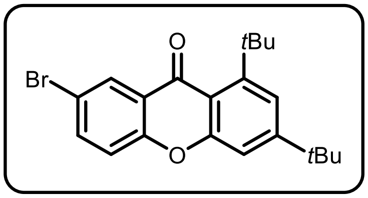

<u>7-bromo-1,3-di-tert-butyl-9H-xanthen-9-one (S6)</u>

5-bromo-2-(3,5-di-*tert*-butylphenoxy)benzoic acid was prepared via general procedure **C2** using **S2** (10.1 g, 26.0 mmol, 1.0 equiv), NaH_2PO_4 (18.7 g, 156 mmol, 6.0 equiv), 2-methyl-2-butene (33.1 mL, 312 mmol, 12.0 equiv) dissolved in ^{t}BuOH (14.4 mL, 0.6 M), THF (14.4 mL, 0.6 M) and $NaClO_2$ (4.7 g, 52 mmol, 2.0 equiv) dissolved in H_2O (14.4 mL, 0.6 M) at room temperature for 12 hours to provide crude 5-bromo-2-(3,5-di-*tert*-butylphenoxy)benzoic acid.

S6 was prepared via general procedure **C2** using crude 5-bromo-2-(3,5-di-*tert*-butylphenoxy)benzoic acid (10.5 g, 26 mmol, 1.0 equiv) and 2,2,2-trifluoroacetic anhydride (10.8 mL, 78 mmol, 3.0 equiv) in 2,2,2-trifluoroacetic acid (26.0 mL, 1.0 M) at room temperature for 12 hours. Following this, the crude solid was purified by vacuum filtration followed by flash column chromatography on silica gel (hexanes/Et_2O 95/5) to afford 7-bromo-1,3-di-*tert*-butyl-9H-xanthen-9-one as a white amorphous solid in >95% yield.

IR (Diamond-ATR, neat) ṽ (cm^{-1}): 2955, 2870, 2200, 2150, 2032, 1966, 1783, 1657, 814.

^{1}H NMR (600 MHz, $CDCl_3$): δ 8.42 (d, *J* = 2.3 Hz, 1H), 7.72 (dd, *J* = 8.9, 1.4 Hz, 1H), 7.57 (d, *J* = 2.0 Hz, 1H), 7.36 (d, *J* = 1.9 Hz, 1H), 7.30 (d, *J* = 8.7 Hz, 1H), 1.59 (s, 9H), 1.39 (s, 9H).

^{13}C NMR (151 MHz, $CDCl_3$): δ 176.6, 159.3, 158.0, 153.3, 153.2, 136.8, 130.0, 125.2, 121.2, 118.9, 118.6, 116.6, 113.9, 37.8, 35.6, 31.2, 31.0.

HRMS (ESI): m/z: $[M+H]^+$ calc'd for $C_{21}H_{24}BrO_2^+$: 387.0959. Found: 387.0954.

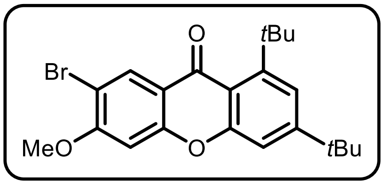

<u>7-bromo-1,3-di-tert-butyl-6-methoxy-9H-xanthen-9-one (S7)</u>

5-bromo-2-(3,5-di-*tert*-butylphenoxy)-4-methoxybenzoic acid was prepared via general procedure **C2** using **S3** (2.40 g, 5.72 mmol, 1.0 equiv), NaH_2PO_4 (4.12 g, 34.3 mmol, 6.0 equiv), 2-methyl-2-butene (7.28 mL, 68.7 mmol, 12.0 equiv) dissolved in ^{t}BuOH (3.2 mL, 0.6 M), THF (3.2 mL, 0.6 M) and $NaClO_2$ (1.03 g, 11.4 mmol, 2.0 equiv) dissolved in H_2O (3.2 mL, 0.6 M) at room temperature for 12 hours to provide crude 5-bromo-2-(3,5-di-*tert*-butylphenoxy)benzoic acid.

S7 was prepared via general procedure **C2** using crude 5-bromo-2-(3,5-di-*tert*-butylphenoxy)-4-methoxybenzoic acid (2.4 g, 6.73 mmol, 1.0 equiv) and 2,2,2-trifluoroacetic anhydride (2.81 mL, 20.2 mmol, 3.0 equiv) in 2,2,2-trifluoroacetic acid (7.1 mL, 1.0 M) at room temperature for 12 hours. Following this, the crude solid was purified by vacuum filtration followed by flash column

chromatography on silica gel (hexanes/Et_2O 95/5) to afford **S7** as a white amorphous solid in 78% yield (2.20 g).

IR (Diamond-ATR, neat) ṽ (cm^{-1}): 2954, 2866, 1686, 1597, 1442, 1270, 1241, 1204, 705.

^{1}H NMR (600 MHz, $CDCl_3$): δ 8.47 (s, 1H), 7.56 (d, *J* = 2.0 Hz, 1H), 7.33 (d, *J* = 2.0 Hz, 1H), 6.83 (s, 1H), 4.01 (s, 3H), 1.59 (s, 9H), 1.39 (s, 9H).

^{13}C NMR (151 MHz, $CDCl_3$): δ 176.0, 160.3, 159.4, 157.4, 155.3, 153.3, 131.8, 121.2, 118.8, 118.6, 113.8, 108.1, 98.9, 56.9, 37.9, 35.6, 31.4, 31.0.

HRMS (ESI): m/z: [M+H]+ calc'd for $C_{22}H_{26}BrO_3^+$: 417.10655. Found: 417.1068

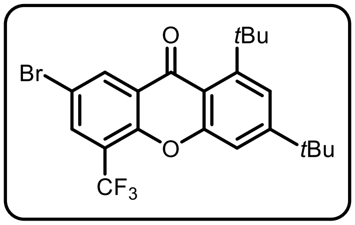

7-bromo-1,3-di-tert-butyl-5-(trifluoromethyl)-9H-xanthen-9-one (S8)

2-(3,5-di-*tert*-butylphenoxy)-3-(trifluoromethyl)benzoic acid was prepared via general procedure **C2** using **S4** (5.30 g, 11.6 mmol, 1.0 equiv), NaH_2PO_4 (8.34 g, 69.5 mmol, 6.0 equiv), 2-methyl-2-butene (14.7 mL, 139 mmol, 12.0 equiv) dissolved in ^{t}BuOH (6.4 mL, 0.6 M), THF (6.4 mL, 0.6 M) and $NaClO_2$ (2.10 g, 23 mmol, 2.0 equiv) dissolved in H_2O (6.4 mL, 0.6 M) at room temperature for 12 hours to provide crude 2-(3,5-di-*tert*-butylphenoxy)-3-(trifluoromethyl)benzoic acid

S8 was prepared via general procedure **C2** using crude 2-(3,5-di-*tert*-butylphenoxy)-3-(trifluoromethyl)benzoic acid (500 mg, 1.1 mmol, 1.0 equiv) and 2,2,2-trifluoroacetic anhydride (441 µL, 3.2 mmol, 3.0 equiv) in 2,2,2-trifluoroacetic acid (1.1 mL, 1.0 M) at room temperature for 12 hours. Following this, the crude solid was purified by vacuum filtration followed by flash column chromatography on silica gel (hexanes/Et_2O 98/2) to afford **S8** as a white amorphous solid in 84% yield.

IR (Diamond-ATR, neat) ṽ (cm^{-1}): 2963, 2870, 1663, 1466, 1347, 1140, 804.

^{1}H NMR (600 MHz, $CDCl_3$): δ 8.54 (d, *J* = 2.7 Hz, 1H), 7.96 (d, *J* = 2.5 Hz, 1H), 7.55 (d, *J* = 2.0 Hz, 1H), 7.31 (d, *J* = 2.0 Hz, 1H), 1.51 (s, 9H), 1.33 (s, 9H).

^{13}C NMR (151 MHz, $CDCl_3$): δ 175.4, 159.0, 158.9, 153.5, 150.5, 134.3, 134.2 ($^3J_{C\text{-}F}$ = 4.9 Hz), 126.3, 122.2 ($^1J_{C\text{-}F}$ = 274.0 Hz), 122.0, 120.5 ($^2J_{C\text{-}F}$ = 33.0 Hz), 118.5, 115.6, 114.2, 37.8, 35.8, 31.2, 31.0.

^{19}F NMR (376 MHz, $CDCl_3$): δ -62.7.

HRMS (ESI): m/z: [M+H]+ calc'd for $C_{22}H_{23}BrF_3O_2^+$: 455.08336. Found: 455.0832

General Procedure C3: Synthesis of S9-11 via debromination

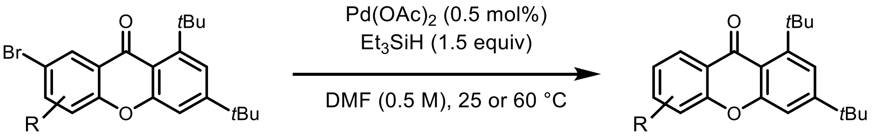

An oven-dried 5 mL microwave vial was equipped with a magnetic stir bar, charged with $Pd(OAc)_2$ (0.5 mol%) and the respective xanthone (1.0 equiv). The reaction vial was sealed with a crimp cap and evacuated and backfilled with nitrogen three times and placed under a nitrogen atmosphere. The solid mixture was then dissolved in anhydrous DMF (0.5 M) to which triethylsilane (2.0 equiv) was added via syringe. The reaction was allowed to stir for 12 hours at room temperature or until completion (as assessed by TLC and GC).

Upon full consumption of the xanthone, the reaction was diluted in Et_2O and washed with H_2O (2 × 100 mL) and brine (1 × 100 mL). The combined organic layers were dried over anhydrous

Na_2SO_4 and concentrated under reduced pressure by rotary evaporation to provide a crude yellow oil. Purification by flash column chromatography on silica gel afforded the target debrominated xanthone.

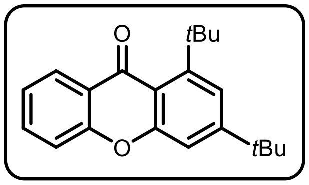

1,3-di-tert-butyl-9H-xanthen-9-one (S9)

S9 was prepared via general procedure **C3** using **S6** (100 mg, 258 µmol, 1.0 equiv), $Pd(OAc)_2$ (290 µg, 1.29 µmol, 0.5 mol%), and triethylsilane (61.9 µL, 387 µmol, 1.5 equiv) at room temperature for 12 hours. Following this, the crude solid was purified by flash column chromatography on silica gel (hexanes/Et_2O 95/5) to afford **S9** as a white amorphous solid in 29% yield (23 mg).

IR (Diamond-ATR, neat) ṽ (cm^{-1}): 2960, 1649, 1604, 1467, 1110, 982, 874, 758, 682.

^{1}H NMR (600 MHz, $CDCl_3$): δ 8.29 (dd, *J* = 8.2, 1.9 Hz, 1H), 7.65 (ddd, *J* = 8.5, 7.1, 1.7 Hz, 1H), 7.56 (d, *J* = 2.1 Hz, 1H), 7.40 (dd, *J* = 8.4, 0.5 Hz, 1H), 7.37 (d, *J* = 1.9 Hz, 1H), 7.33 (ddd, *J* = 8.0, 7.2, 1.0 Hz, 1H), 1.61 (s, 9H), 1.40 (s, 9H).

^{13}C NMR (151 MHz, $CDCl_3$): δ 178.0, 159.4, 157.5, 154.6, 153.1, 134.0, 127.3, 124.0, 123.6, 120.9, 119.0, 116.9, 113.9, 37.8, 35.6, 31.5, 31.0.`

HRMS (ESI): m/z: $[M+H]^+$ calc'd for $C_{21}H_{25}O_2^+$: 309.18547. Found: 309.1846.

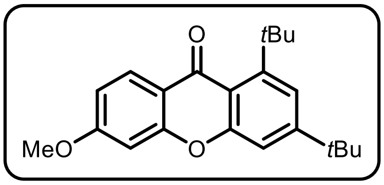

1,3-di-tert-butyl-6-methoxy-9H-xanthen-9-one (S10)

S10 was prepared via a modified version general procedure **C3** using **S7** (150 mg, 359 µmol, 1.0 equiv), $Pd(PPh_3)_4$ (4.15 mg, 3.59 µmol, 1.0 mol%), and paraformaldehyde (10.8 mg, 3.59 µmol, 1.0 equiv) at room temperature for 12 hours. Following this, the crude solid was purified by flash column chromatography on silica gel (hexanes/Et_2O 95/5) to afford **S10** as a yellow amorphous solid in 20% yield (25 mg).

IR (Diamond-ATR, neat) ṽ (cm^{-1}): 2957, 1648, 1607, 1442, 1274, 1160, 1101, 837, 679.

^{1}H NMR (600 MHz, $CDCl_3$): δ 8.20 (d, *J* = 8.9 Hz, 1H), 7.54 (d, *J* = 2.0 Hz, 1H), 7.33 (d, *J* = 2.0 Hz, 1H), 6.89 (dd, *J* = 8.9, 2.4 Hz, 1H), 6.80 (d, *J* = 2.4 Hz, 1H), 3.91 (s, 3H), 1.61 (s, 9H), 1.39 (s, 9H).

^{13}C NMR (151 MHz, $CDCl_3$): δ 177.1, 164.5, 159.5, 156.9, 156.3, 153.0, 128.9, 120.8, 119.0, 117.9, 113.7, 112.9, 99.1, 55.9, 37.8, 35.6, 31.4, 31.0.

HRMS (ESI): m/z: $[M+H]^+$ calc'd for $C_{22}H_{25}BrO_3^+$: 339.19604. Found: 339.1953.

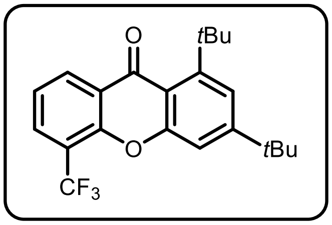

1,3-di-tert-butyl-5-(trifluoromethyl)-9H-xanthen-9-one (S11)

S11 was prepared via general procedure **C3** using **S8** (68.6 mg, 151 µmol, 1.0 equiv), $Pd(OAc)_2$ (169 µg, 0.75 µmol, 0.5 mol%), and triethylsilane (36.1 µL, 227 µmol, 1.5 equiv) at room temperature for 12 hours. Following this, the crude solid was purified by flash column chromatography on silica gel (hexanes/Et_2O 95/5) to afford **S11** as a white amorphous solid in 60% yield (34 mg).

IR (Diamond-ATR, neat) ṽ (cm^{-1}): 2961, 2906, 1664, 1597, 1259, 1109, 1023, 784.

^{1}H NMR (600 MHz, $CDCl_3$): δ 8.49 (dd, *J* = 8.1, 1.7 Hz, 1H), 7.94 (dd, *J* = 7.6, 1.7 Hz, 1H), 7.61 (d, *J* = 2.0 Hz, 1H), 7.39 (d, *J* = 2.1 1H), 6.78 (t, *J* = 7.57, 1H), 1.60 (s, 9H), 1.41 (s, 9H).

^{13}C NMR (151 MHz, $CDCl_3$): δ 176.7, 158.9, 158.4, 153.2, 151.7, 131.7, 131.3 ($^3J_{C\text{-}F}$ = 4.53 Hz), 124.9, 123.1 ($^1J_{C\text{-}F}$ = 273.31 Hz), 122.7, 121.7, 118.8, 118.6 ($^2J_{C\text{-}F}$ = 33.2 Hz), 114.2, 37.8, 35.8, 31.2, 31.0.

HRMS (ESI): m/z: $[M+H]^+$ calc'd for $C_{22}H_{24}F_3O_2^+$: 377.17284. Found: 377.1728.

General Procedure C4: Synthesis of S12-S16 via Suzuki cross-coupling

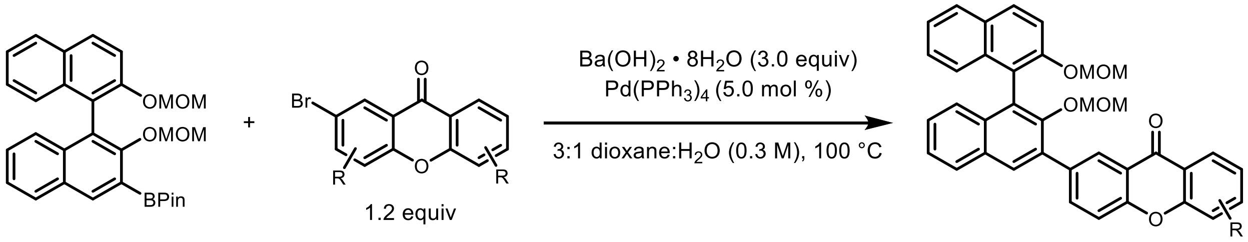

A flame dried 25 mL round-bottomed flask was equipped with a magnetic stir bar, charged with 2-(2,2'-bis(methoxymethoxy)-[1,1'-binaphthalen]-3-yl)-4,4,5,5-tetramethyl-1,3,2-dioxaborolane[1] (1.0 equiv), bari-um hydroxide octahydrate (3.0 equiv), $Pd(PPh_3)_4$ (5 mol%), and the respective bromoxanthone derivative (1.2 equiv) as solids. The reaction apparatus was fitted with a greased reflux condenser, sealed with a rubber septum, evacuated and backfilled with nitrogen three times and placed under a nitrogen atmosphere. The solid reaction components were then dissolved in a 3:1 mixture of 1,4-dioxane (0.3 M) and H_2O (0.3 M). The solution was then degassed by sparging argon gas through the solution concomitant with sonication for 1 min per mL of solvent added at 25 °C. The reflux condenser was then reinforced with Teflon tape and the reaction mixture was heated to 100 °C by transferring the reaction apparatus to a pre-heated oil bath. The reaction mixture was allowed to stir at this temperature for 18 hours or until completion (as assessed by TLC and GC).

Upon full consumption of 2-(2,2'-bis(methoxymethoxy)-[1,1'-binaphthalen]-3-yl)-4,4,5,5-tetramethyl-1,3,2-dioxaborolane, the reaction was filtered through a celite plug and the crude mixture was concentrated under reduced pressure to provide a crude yellow oil. The oil was dissolved in CH_2Cl_2 (100 mL) and transferred to a separatory funnel. The organic layer was washed with H_2O (2 × 75 mL) and brine (1 × 75 mL). The combined organic layers were dried over anhydrous Na_2SO_4 and concentrated under reduced pressure by rotary evaporation to provide a crude yellow oil. Purification by flash column chromatography on silica gel afforded the target cross coupling product.

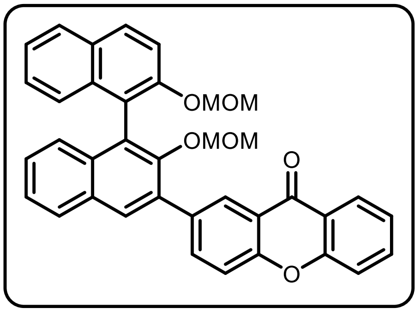

2-(2,2'-bis(methoxymethoxy)-[1,1'-binaphthalen]-3-yl)-9H-xanthen-9-one (S12)

S12 was prepared via general procedure **C4** using 2-(2,2'-bis(methoxymethoxy)-[1,1'-binaphthalen]-3-yl)-4,4,5,5-tetramethyl-1,3,2-dioxaborolane[1] (1.00 g, 2.0 mmol, 1.0 equiv), barium hydroxide octahydrate (1.90 g, 6.0 mmol, 3.0 equiv), $Pd(PPh_3)_4$ (115 mg, 0.1 mmol, 5 mol%), and 2-bromo-9H-xanthen-9-one (660 mg, 2.4 mmol, 1.2 equiv) in 1,4-dioxane (5 mL, 0.3 M) and H_2O (1.6 mL, 0.3 M) at 100 °C for 16 hours. Purification by flash column chromatography on silica gel (hexanes/EtOAc 100/0 to hexanes/EtOAc 80/20) afforded **S12** in 89% yield (1.00 g).

IR (Diamond-ATR, neat) ṽ (cm^{-1}): 3054, 2926, 2822, 1732, 1651, 1614, 1592, 1506, 1493, 1465, 1434, 1146.

^{1}H NMR (600 MHz, $CDCl_3$) δ 8.69 (d, J = 2.3 Hz, 1H), 8.40 (dd, J = 7.9, 1.7 Hz, 1H), 8.19 (dd, J = 8.7, 2.3 Hz, 1H), 8.07 (s, 1H), 7.97 (d, J = 9.1 Hz, 1H), 7.94 (d, J = 8.2 Hz, 1H), 7.87 (d, J = 8.3

Hz, 1H), 7.76 (atd, J = 7.1, 1.7 Hz, 1H), 7.62 (d, J = 9.0 Hz, 1H), 7.59 (d, J = 8.7 Hz, 1H), 7.54 (d, J = 1.1 Hz, 1H), 7.46 – 7.42 (m, 1H), 7.43 – 7.40 (m, 1H), 7.40 – 7.36 (m, 1H), 7.30 (dd, J = 33.1, 1.3 Hz, 3H), 7.25 (d, J = 1.3 Hz, 1H), 5.20 (d, J = 7.0 Hz, 1H), 5.12 (d, J = 7.0 Hz, 1H), 4.36 (d, J = 5.7 Hz, 1H), 4.31 (d, J = 5.7 Hz, 1H), 3.27 (s, 3H), 2.35 (s, 3H).
^{13}C NMR (151 MHz, $CDCl_3$) δ 177.2, 156.2, 155.5, 152.9, 150.9, 136.8, 135.2, 134.9, 134.0, 133.9, 133.6, 131.1, 130.6, 129.8, 129.7, 128.1, 127.9, 127.1, 126.8, 126.7, 126.5, 125.9, 125.7, 125.4, 124.2, 124.1, 124.0, 121.9, 121.8, 120.8, 118.1, 117.8, 116.6, 98.8, 95.0, 56.2, 56.0.
HRMS (ESI): m/z: $[M+H]^+$ calc'd for $C_{37}H_{29}O_6^+$: 569.1964. Found: 569.1923.

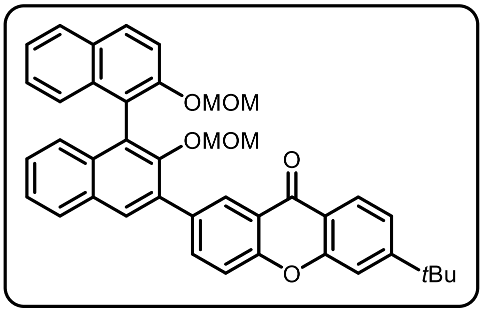

2-(2,2'-bis(methoxymethoxy)-[1,1'-binaphthalen]-3-yl)-6-(tert-butyl)-9H-xanthen-9-one (S13)

S13 was prepared via general procedure **C4** using 2-(2,2'-bis(methoxymethoxy)-[1,1'-binaphthalen]-3-yl)-4,4,5,5-tetra-m- ethyl-1,3,2-dioxaborolane[1] (667 mg, 1.0 mmol, 1.0 equiv), barium hydroxide octahydrate (946 mg, 3.0 mmol, 3.0 equiv), $Pd(PPh_3)_4$ (57.8 mg, 0.05 mmol, 5 mol%), and **S5** (397 mg, 1.2 mmol, 1.2 equiv) in 1,4-dioxane (2.5 mL, 0.3 M) and H_2O (0.8 mL, 0.3 M) at 100 °C for 16 hours. Purification by flash column chromatography on silica gel (hexanes/Et_2O 100/0 to hexanes/Et_2O 80/20) afforded **S13** in 76% yield (472 mg).
IR (Diamond-ATR, neat) ṽ (cm^{-1}): 2957, 2930, 2869, 1653, 1618, 1589, 1497, 1484, 1463, 1420, 1362.
^{1}H NMR (600 MHz, $CDCl_3$) δ 8.71 (s, 1H), 8.33 (dd, J = 8.5, 2.1 Hz, 1H), 8.19 (dd, J = 8.6, 2.1 Hz, 1H), 8.10 (s, 1H), 8.00 (d, J = 9.1 Hz, 1H), 7.97 (d, J = 8.3 Hz, 1H), 7.90 (d, J = 8.2 Hz, 1H), 7.64 (dd, J = 9.0, 2.1 Hz, 1H), 7.60 (dd, J = 8.7, 2.0 Hz, 1H), 7.55 (s, 1H), 7.49 (d, J = 8.5 Hz, 1H), 7.48 – 7.44 (m, 1H), 7.40 – 7.35 (m, 1H), 7.33 – 7.28 (m, 2H), 7.28 – 7.25 (m, 1H), 7.25 – 7.22 (m, 1H), 5.22 (d, J = 7.0 Hz, 1H), 5.14 (d, J = 7.0 Hz, 1H), 4.38 (d, J = 5.8 Hz, 1H), 4.33 (d, J = 5.8 Hz, 1H), 3.29 (s, 3H), 2.37 (s, 3H), 1.45 (s, 9H).
^{13}C NMR (151 MHz, $CDCl_3$) δ 177.1, 159.7, 159.7, 156.5, 155.7, 153.1, 151.0, 136.7, 135.2, 134.2, 134.1, 133.7, 131.2, 130.7, 129.9, 129.8, 128.3, 128.0, 127.2, 126.8, 126.6, 126.5, 126.0, 125.9, 125.5, 124.3, 122.2, 122.1, 121.0, 119.7, 117.8, 116.7, 114.5, 98.9, 95.1, 56.3, 56.2, 35.7, 31.2.
HRMS (ESI): m/z: $[M+H]^+$ calc'd for $C_{41}H_{36}O_6^+$: 625.2590. Found: 625.2563.

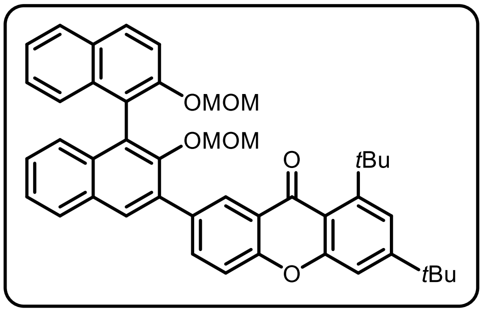

7-(2,2'-bis(methoxymethoxy)-[1,1'-binaphthalen]-3-yl)-1,3-di-tert-butyl-9H-xanthen-9-one (S14)

S13 was prepared via general procedure **C4** using 2-(2,2'-bis(methoxymethoxy)-[1,1'-binaphthalen]- 3-yl)-4,4,5,5-tetra-methyl-1,3,2-dioxaborolane[1] (2.67 g, 4.00 mmol, 1.0 equiv), barium hydroxide octahydrate (3.79 g, 12.0 mmol, 3.0 equiv), $Pd(PPh_3)_4$ (231 mg, 0.2 mmol, 5 mol%), and **S6** (1.86 g, 4.8 mmol, 1.2 equiv) in 1,4-dioxane (10 mL, 0.3 M) and H_2O (3.3 mL, 0.3 M) at 100 °C for 16 hours. Purification by flash column chromatography on silica gel (hexanes/Et_2O 100/0 to hexanes/Et_2O 80/15) afforded **S14** in 82% yield (2.22 g).
IR (Diamond-ATR, neat) ṽ (cm^{-1}): 2962, 1652, 1622, 1612, 1506, 1466, 1453, 1435.

^{1}H NMR (600 MHz, $CDCl_3$) δ 8.65 (d, *J* = 2.2 Hz, 1H), 8.12 (dd, *J* = 8.7, 2.2 Hz, 1H), 8.09 (s, 1H), 7.97 (d, *J* = 9.1 Hz, 1H), 7.92 (d, *J* = 8.2 Hz, 1H), 7.87 (d, *J* = 8.1 Hz, 1H), 7.61 (d, *J* = 9.0 Hz, 1H), 7.58 (d, *J* = 2.1 Hz, 1H), 7.48 (d, *J* = 8.6 Hz, 1H), 7.45 – 7.41 (m, 1H), 7.40 (d, *J* = 1.9 Hz, 1H), 7.37 (ddd, *J* = 8.1, 6.2, 1.7 Hz, 1H), 7.32 – 7.28 (m, 2H), 7.26 (at, *J* = 6.5 Hz, 1H), 7.22 (d, *J* = 8.5 Hz, 1H), 5.19 (d, *J* = 7.0 Hz, 1H), 5.09 (d, *J* = 7.0 Hz, 1H), 4.36 (d, *J* = 5.7 Hz, 1H), 4.31 (d, *J* = 5.7 Hz, 1H), 3.23 (s, 3H), 2.35 (s, 3H), 1.63 (s, 9H), 1.41 (s, 9H).
^{13}C NMR (151 MHz, $CDCl_3$) δ 178.0, 159.5, 157.7, 154.0, 153.3, 153.2, 151.2, 136.1, 134.9, 134.4, 134.3, 133.8, 131.3, 130.8, 130.0, 129.9, 128.3, 128.1, 127.9, 126.9, 126.6, 126.1, 126.0, 125.5, 124.4, 124.0, 121.3, 121.1, 119.1, 116.9, 116.8, 114.1, 99.0, 95.3, 56.4, 56.2, 38.0, 35.8, 31.6, 31.5, 31.1.
HRMS (ESI): m/z: $[M+H]^+$ calc'd for $C_{45}H_{45}O_6^+$: 681.3216. Found: 681.3225.

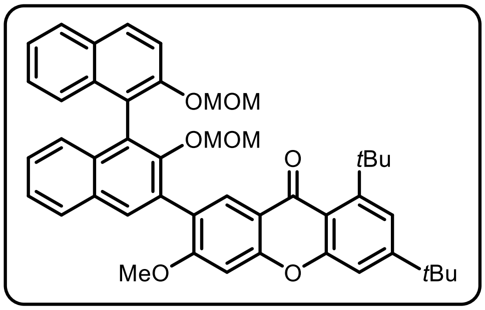

7-(2,2'-bis(methoxymethoxy)-[1,1'-binaphthalen]-3-yl)-1,3-di-tert-butyl-6-methoxy-9H-xanthen-9-one (S15)

S15 was prepared via general procedure **C4** using 2-(2,2'-bis(methoxymethoxy)-[1,1'-binaphthalen]- 3-yl)-4,4,5,5-tetra-methyl-1,3,2-dioxaborolane[1] (2.00 g, 3.00 mmol, 1.0 equiv), barium hydroxide octahydrate (2.83 g, 9.00 mmol, 3.0 equiv), $Pd(PPh_3)_4$ (173 mg, 0.15 mmol, 5 mol%), and **S7** (1.50 g, 3.60 mmol, 1.2 equiv) in 1,4-dioxane (7.5 mL, 0.3 M) and H_2O (2.5 mL, 0.3 M) at 100 °C for 16 hours. Purification by flash column chromatography on silica gel (hexanes/Et_2O 100/0 to hexanes/Et_2O 80/15) afforded **S15** in 43% yield (908 mg).
IR (Diamond-ATR, neat) ṽ (cm^{-1}): 2961, 2926, 2866, 1598, 1442, 1271, 1242, 753, 673.
^{1}H NMR (600 MHz, $CDCl_3$) δ 8.39 (s, 1H), 7.95 (s, 1H), 7.94 (ad, 1H), 7.87 (dd, *J* = 14.6, 7.7 Hz, 2H), 7.59 (d, *J* = 9.8 Hz, 1H), 7.57 (s, 1H), 7.42 – 7.28 (m, 5H), 7.25 (as, 2H), 6.89 (s, 1H), 5.17 (d, *J* = 6.9 Hz, 1H), 5.09 (d, *J* = 6.8 Hz, 1H), 4.38 (br s, 1H), 4.29 (d, *J* = 5.9 Hz, 1H), 3.93 (s, 3H), 3.24 (s, 3H), 2.35 (s, 3H), 1.62 (s, 9H), 1.40 (s, 9H).
^{13}C NMR (151 MHz, $CDCl_3$) δ 176.8, 162.6, 159.3, 156.8, 155.9, 153.0, 152.1, 149.5, 134.1, 133.7, 131.9, 130.9, 129.8, 129.7, 129.7, 128.0, 127.8, 126.5, 126.2, 126.2, 125.9, 125.8, 125.3, 125.0, 124.1, 124.1, 124.1, 120.8, 119.0, 117.3, 113.6, 102.5, 98.7, 97.4, 56.4, 56.0, 55.9, 37.7, 35.4, 31.3, 30.9.
HRMS (ESI): m/z: $[M+H]^+$ calc'd for $C_{46}H_{47}O_7^+$: 711.3322. Found: 711.3323.

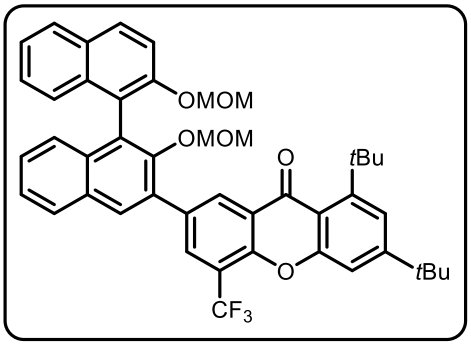

7-(2,2'-bis(methoxymethoxy)-[1,1'-binaphthalen]-3-yl)-1,3-di-tert-butyl-5-(trifluoromethyl)-9H-xanthen-9-one (S16)

S16 was prepared via general procedure **C4** using 2-(2,2'-bis(methoxymethoxy)-[1,1'-binaphthalen]- 3-yl)-4,4,5,5-tetra-methyl-1,3,2-dioxaborolane[1] (369 mg, 7.38 mmol, 1.0 equiv), barium hydroxide octahydrate (698 mg, 2.21 mmol, 3.0 equiv), $Pd(PPh_3)_4$ (42.6 mg, 36.9 μmol, 5 mol%), and **S8** (403 mg, 0.89 mmol, 1.2 equiv) in 1,4-dioxane (2.21 mL, 0.3 M) and H_2O (0.74 mL, 0.3 M) at 100 °C for

16 hours. Purification by flash column chromatography on silica gel (hexanes/Et_2O 100/0 to hexanes/Et_2O 80/15) afforded **S16** in 46% yield (0.232 g).

IR (Diamond-ATR, neat) ṽ (cm^{-1}): 2965, 1657, 1616, 1485, 1241, 1135, 970, 750.

^{1}H NMR (600 MHz, $CDCl_3$) δ 8.85 (d, *J* = 2.2 Hz, 1H), 8.41 (d, *J* = 2.2 Hz, 1H), 8.08 (s, 1H), 7.98 (d, *J* = 9.1 Hz, 1H), 7.94 (d, *J* = 8.1 Hz, 1H), 7.91 – 7.86 (m, 1H), 7.65 – 7.59 (m, 2H), 7.47 – 7.42 (m, 2H), 7.38 (ddd, *J* = 8.1, 6.6, 1.3 Hz, 1H), 7.32 (ddd, *J* = 8.2, 6.6, 1.3 Hz, 1H), 7.30 – 7.26 (m, 2H), 7.22 (d, *J* = 8.5 Hz, 1H), 5.19 (d, *J* = 7.1 Hz, 1H), 5.14 (d, *J* = 7.1 Hz, 1H), 4.40 (d, *J* = 5.9 Hz, 1H), 4.32 (d, *J* = 5.9 Hz, 1H), 3.27 (s, 3H), 2.46 (s, 3H), 1.62 (s, 9H), 1.43 (s, 9H).

^{13}C NMR (151 MHz, $CDCl_3$) δ 176.7, 159.0, 158.5, 153.3, 153.0, 151.3, 150.8, 134.2, 134.1, 134.0, 133.3 ($^3J_{C\text{-}F}$ = 4.53 Hz), 133.1, 132.1, 131.1, 130.7, 130.1, 129.8, 128.3, 128.0, 126.9, 126.8, 126.7, 126.0, 125.7, 125.6, 124.9, 124.3, 123.2 ($^1J_{C\text{-}F}$ = 271.8 Hz), 121.8, 120.6, 118.8, 118.3 ($^2J_{C\text{-}F}$ = 31.72), 116.4, 114.2, 99.2, 94.9, 56.4, 56.1, 37.9, 35.8, 31.3, 31.0.

^{19}F NMR (377 MHz, $CDCl_3$) δ -62.2.

HRMS (ESI): m/z: $[M+H]^+$ calc'd for $C_{46}H_{44}F_3O_6^+$: 749.30902. Found: 794.3104

General Procedure C5: Synthesis of SI-PC1, SI-PC2, PC1, PC2, PC3, Me-PC1

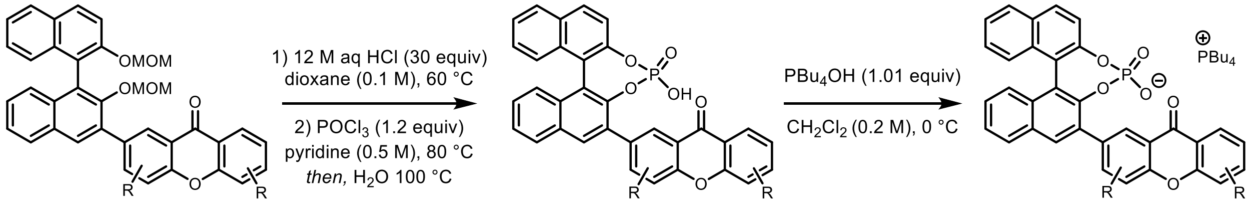

An oven-dried 50 mL round-bottomed flask was equipped with a magnetic stir bar and charged with the respective MOM-BINOL intermediate (**S12**–**S16**) (1.0 equiv). The flask was equipped with a greased reflux condenser, sealed with a rubber septum, evacuated and backfilled with nitrogen three times, and placed under an argon atmosphere. The solid was then dissolved in anhydrous 1,4-dioxane (0.1 M) followed by a dropwise addition of 12 M HCl (30.0 equiv). The joint of the reflux condenser was reinforced with Teflon tape, and the reaction mixture was heated to 60 °C by transferring the reaction apparatus to a pre-heated oil bath. The reaction was allowed to stir for 1 hour at 60 °C or until complete consumption of starting material (as assessed by TLC). Upon full consumption of starting material, the reaction apparatus was removed from the oil bath and allowed to cool to room temperature. The reaction mixture was then diluted with CH_2Cl_2 (40 mL) and transferred to a separatory funnel. The organic layer was washed with H_2O (3 × 50 mL), dried over anhydrous Na_2SO_4 and concentrated under reduced pressure resulting in a yellow solid which was carried forward directly into the next reaction.

An oven-dried vial (**1**) was equipped with a magnetic stir bar, charged with the bisphenol precursor (1.0 equiv), and sealed with a crimp cap. The vial was then evacuated and backfilled with nitrogen three times and placed under a nitrogen atmosphere. Concurrently, a separate oven-dried vial (**2**) was sealed with rubber septum, evacuated and backfilled with nitrogen three times and placed under a nitrogen atmosphere. The vial (**2**) was charged with $POCl_3$ (1.2 equiv) and anhydrous pyridine (0.5 M). The pyridine solution was then transferred to the first vial (**1**) containing the bisphenol substrate via syringe in a dropwise fashion. The vial (**1**) was then heated to 80 °C through transfer to a pre-heated oil bath and stirred for 2 hours. Following this, the vial was removed from the oil bath and allowed to cool to room temperature at which point H_2O (1 mL) was added to the reaction solution via syringe. The vial was then placed in a pre-heated 100 °C

oil bath and allowed to stir at this temperature for 5 minutes (or until completion as assessed by ^{1}H NMR aliquots).

Upon completion, the reaction was removed from the oil bath and allowed to cool to room temperature. The reaction mixture was then diluted with CH_2Cl_2 (8 mL) and transferred to a separatory funnel. The organic layer was washed with 6 M HCl (3 × 5 mL), dried over anhydrous Na_2SO_4 and concentrated under reduced pressure to provide a brown solid which was carried forward directly into the next step.

The crude mixture was then transferred to an oven-dried 25-mL round-bottomed flask, equipped with a magnetic stir bar and sealed with a rubber septum. The round-bottomed flask was evacuated and backfilled with nitrogen three times and placed under nitrogen atmosphere. The solid was dissolved in CH_2Cl_2 (0.2 M), after which the reaction apparatus was placed into an ice-water bath to cool it to 0 °C. After stirring at this temperature for 5 minutes, tetrabutyl(hydroxy)-1-phosphane (40% in H_2O, 1.0 equiv) was added to the reaction dropwise. The resulting mixture was then stirred at 0 °C for 30 minutes (or until completion by ^{31}P NMR aliquots). Upon completion, the reaction was concentrated under reduced pressure and purified via reversed-phase column chromatography (MeCN/H_2O 0/100 to MeCN/H_2O 100/0) to afford the target catalyst derivative.

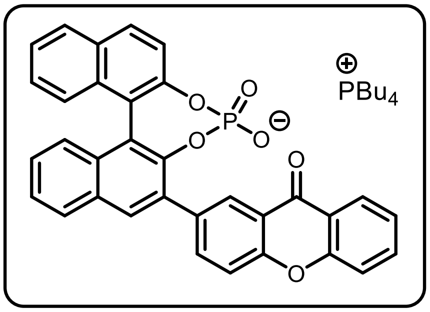

Tetrabutylphosphonium2-(9-oxo-9H-xanthen-2-yl)dinaphtho[2,1-d:1',2'-f][1,3,2]dioxaphosphepin-4-olate 4-oxide (SI-PC1)

2-(2,2'-dihydroxy-[1,1'-binaphthalen]-3-yl)-9H-xanthen-9-one was prepared via the general deprotection procedure **C5** using **S12** (0.76 g, 1.3 mmol, 1.0 equiv) and 12 M HCl (3.30 mL, 40 mmol, 30.0 equiv) in 1,4-dioxane (13.0 mL, 0.1 M) at 60 °C for 1 hour. Following this, crude 2-(2,2'-dihydroxy-[1,1'-binaphthalen]-3-yl)-9H-xanthen-9-one was afforded in 99% yield (640 mg) as a yellow foam.

2-(4-hydroxy-4-oxidodinaphtho[2,1-d:1',2'-f][1,3,2]dioxaphosphepin-2-yl)-9H-xanthen-9-one was prepared via the general phosphorylation procedure **C5** using 2-(2,2'-dihydroxy-[1,1'-binaphthalen]-3-yl)-9H-xanthen-9-one (1.49 g, 3.1 mmol, 1.0 equiv) and $POCl_3$ (347 µL, 3.7 mmol, 1.2 equiv) in pyridine (6.20 mL, 0.5 M) at 85 °C for 5 hours. Following this, crude 2-(4-hydroxy-4-oxidodinaphtho[2,1-d:1',2'-f][1,3,2]dioxaphosphepin-2-yl)-9H-xanthen-9-one was accessed in 87% yield (1.49 g) as a brown foam.

SI-PC1 was prepared via the general salt formation procedure **C5** using 2-(4-hydroxy-4-oxidodinaphtho[2,1-d:1',2'-f][1,3,2]dioxaphosphepin-2-yl)-9H-xanthen-9-one (1.21 g, 2.2 mmol, 1.0 equiv) and tetrabutyl(hydroxy)-1-phosphane (1.75 mL, 40% in H_2O, 1.0 equiv) in CH_2Cl_2 (11.1 mL, 0.2 M) at 0 °C for 30 minutes. Following this, **SI-PC1** was afforded in 21% yield (380 mg).

IR (Diamond-ATR, neat) ṽ (cm-1): 2958, 2930, 2871, 1652, 1614, 1592, 1493, 1465.

^{1}H NMR (600 MHz, $CDCl_3$) δ 8.91 (dd, *J* = 8.8, 2.3 Hz, 1H), 8.56 (d, *J* = 2.3 Hz, 1H), 8.34 (dd, *J* = 8.0, 1.7 Hz, 1H), 8.05 (s, 1H), 7.91 (at, *J* = 8.9 Hz, 2H), 7.86 (d, *J* = 8.2 Hz, 1H), 7.75 (at, *J* = 7.7, 1H), 7.59 (at, *J* = 8.9 Hz, 2H), 7.55 (d, *J* = 8.4 Hz, 1H), 7.39 (atd, *J* = 7.4, 3.3 Hz, 2H), 7.34 (d, *J* = 9.0 Hz, 3H), 7.21 (at, *J* = 7.7 Hz, 2H), 1.97 – 1.86 (m, 8H), 1.18 – 1.09 (m, 16H), 0.74 (t, *J* = 6.8 Hz, 12H).

^{13}C NMR (151 MHz, $CDCl_3$) δ 177.5, 156.3, 155.6, 150.4 (d, $^{2}J_{C-P}$ = 9.3 Hz), 147.8 (d, $^{2}J_{C-P}$ = 9.4 Hz), 138.9, 135.4, 134.9, 133.4, 132.9, 132.8, 130.9, 130.7, 130.6, 129.9, 128.3, 128.2, 127.2, 127.1, 127.0, 126.6, 125.9, 125.8, 124.9, 124.3, 123.9, 123.6, 122.7, 122.7, 121.9, 121.3, 118.3, 117.7, 23.8 (d, $^{2}J_{C-P}$ = 15.1 Hz), 23.5 (d, $^{3}J_{C-P}$ = 4.8 Hz), 18.3 (d, $^{1}J_{C-P}$ = 47.3 Hz), 13.4.
^{31}P NMR (162 MHz, $CDCl_3$) δ 33.6, 6.4.
HRMS (ESI): m/z: $[M+H]^+$ calc'd for $C_{49}H_{55}O_6P_2$: 801.3472. Found: 801.3459.

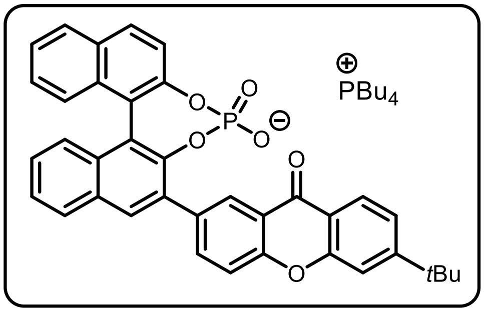

Tetrabutylphosphonium-2-(6-(tert-butyl)-9-oxo-9H-xanthen-2-yl)dinaphtho[2,1-d:1',2'-f][1,3,2]dioxaphosphepin-4-olate 4-oxide (SI-PC2)

6-(*tert*-butyl)-2-(2,2'-dihydroxy-[1,1'-binaphthalen]-3-yl)-9H-xanthen-9-one was prepared using the general deprotection procedure **C5** using **S13** (0.40 g, 0.64 mmol, 1.0 equiv) and 12 M HCl (1.60 mL, 19 mmol, 30.0 equiv) in 1,4-dioxane (6.40 mL, 0.1 M) at 60 °C for 1 hour. Following this, crude 6-(*tert*-butyl)-2-(2,2'-dihydroxy-[1,1'-binaphthalen]-3-yl)-9H-xanthen-9-one was afforded in 99% yield (340 mg) as a yellow foam.

6-(*tert*-butyl)-2-4-hydroxy-4-oxidodinaphtho[2,1-d:1',2'-f][1,3,2]dioxaphosphepin-2-yl)-9H-xanthen-9-one was prepared using the general phosphorylation procedure **C5** using 6-(*tert*-butyl)-2- (2,2'-dihydroxy-[1,1'-binaphthalen]-3-yl)-9H-xanthen-9-one (0.34 g, 0.63 mmol, 1.0 equiv) and $POCl_3$ (71.1 µL, 0.76 mmol, 1.2 equiv) in pyridine (1.20 mL, 0.5 M) at 85 °C for 5 hours. Following this, crude 6-(*tert*-butyl)-2-(4-hydroxy-4-oxidodinaphtho[2,1-d:1',2'-f][1,3,2]dioxaphosphepin-2-yl)-9H-xanthen-9-one was afforded in 75% yield (283 mg) as a brown foam.

SI-PC2 was prepared using the general salt formation procedure **C5** using 6-(*tert*-butyl)-2-(4-hydroxy-4-oxidodinaphtho[2,1-d:1',2'-f][1,3,2]dioxaphosphepin-2-yl)-9H-xanthen-9-one (0.28 g, 0.47 mmol, 1.0 equiv) and tetrabutyl(hydroxy)-1-phosphane (0.54 mL, 40% in H_2O, 1.0 equiv) in CH_2Cl_2 (2.39 mL, 0.2 M) at 0 °C for 30 minutes. Following this, **SI-PC2** was afforded in 22% yield (88.0 mg).

IR (Diamond-ATR, neat) ṽ (cm^{-1}): 2957, 2930, 2869, 1653, 1611, 1589, 1497, 1484, 1463, 1420, 1362.
^{1}H NMR (600 MHz, $CDCl_3$) δ 8.95 (d, *J* = 9.7 Hz, 1H), 8.52 (s, 1H), 8.25 (d, *J* = 8.4 Hz, 1H), 8.06 (s, 1H), 7.90 (at, *J* = 9.2 Hz, 2H), 7.86 (d, *J* = 8.2 Hz, 1H), 7.58 (at, *J* = 7.8 Hz, 2H), 7.53 (s, 1H), 7.44 (d, *J* = 8.4 Hz, 1H), 7.39 (at, *J* = 7.4 Hz, 1H), 7.34 (d, *J* = 8.5 Hz, 3H), 7.20 (at, *J* = 7.5 Hz, 2H), 1.89 (s, 8H), 1.42 (s, 9H), 1.19 – 1.11 (m, 16H), 0.73 (t, *J* = 6.8 Hz, 12H).
^{13}C NMR (151 MHz, $CDCl_3$) δ 177.2, 159.4, 156.4, 155.6, 150.5 (d, $^{2}J_{C-P}$ = 9.1 Hz), 147.8 (d, $^{2}J_{C-P}$ = 9.4 Hz), 138.7, 135.3, 133.5, 132.8, 132.7, 130.8, 130.7, 130.4, 129.8, 128.2, 128.1, 127.1, 127.0, 126.9, 126.1, 125.8, 125.6, 124.7, 124.2, 123.5, 122.8, 122.6, 121.8, 121.4, 119.5, 117.5, 114.5, 35.6, 31.0, 23.8 (d, $^{2}J_{C-P}$ = 15.3 Hz), 23.5 (d, $^{3}J_{C-P}$ = 4.9 Hz), 18.3 (d, $^{1}J_{C-P}$ = 47.4 Hz), 13.4.
^{31}P NMR (162 MHz, $CDCl_3$) δ 33.9, 6.6.
HRMS (ESI): m/z: $[M+H]^+$ calc'd for $C_{53}H_{63}O_6P_2$: 857.4100. Found: 857.4070.

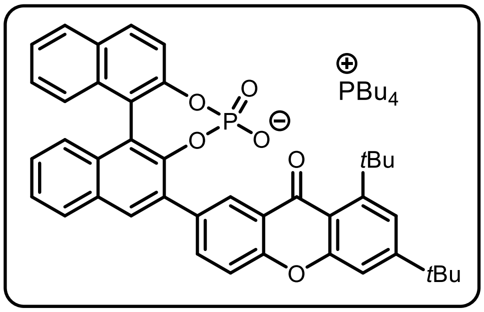

Tetrabutylphosphonium2-(6,8-di-tert-butyl-9-oxo-9H-xanthen-2-yl)dinaphtho[2,1-d:1',2'-f][1,3,2]dioxaphosphepin-4-olate 4-oxide (PC1)

1,3-di-*tert*-butyl-7-(2,2'-dihydroxy-[1,1'-binaphthalen]-3-yl)-9H-xanthen-9-one was prepared via the general deprotection procedure **C5** using **S14** (3.50 g, 5.1 mmol, 1.0 equiv) and 12 M HCl (10.3 mL, 123 mmol, 30.0 equiv) in 1,4-dioxane (51.6 mL, 0.1 M) at 60 °C for 1 hour. Following this, crude 1,3-di-*tert*-butyl-7-(2,2'-dihydroxy-[1,1'-binaphthalen]-3-yl)-9H-xanthen-9-one was afforded in 97% yield (2.96 g) as a yellow foam.

1,3-di-*tert*-butyl-7-(4-hydroxy-4-oxidodinaphtho[2,1-d:1',2'-f][1,3,2]dioxaphosphepin-2-yl)-9H-xanthen-9-one was prepared using the general phosphorylation procedure **C5** using 1,3-di-*tert*-butyl-7-(2,2'-dihydroxy-[1,1'-binaphthalen]-3-yl)-9H-xanthen-9-one (2.96 g, 5.0 mmol, 1.0 equiv) and $POCl_3$ (559 µL, 6.0 mmol, 1.2 equiv) in pyridine (10.0 mL, 0.5 M) at 85 °C for 5 hours. Following this, crude 1,3-di-*tert*-butyl-7-(-4-hydroxy-4-oxidodinaphtho[2,1-d:1',2'-f][1,3,2]dioxaphosphepin-2-yl)-9H-xanthen-9-one was afforded in 95% yield (3.11 g) as a brown foam.

PC1 was prepared via the general salt formation procedure **C5** using 1,3-di-*tert*-butyl-7-(-4-hydroxy-4-oxidodinaphtho[2,1-d:1',2'-f][1,3,2]dioxaphosphepin-2-yl)-9H-xanthen-9-one (654 mg, 1.0 mmol, 1.0 equiv) and tetrabutyl(hydroxy)-1-phosphane (1.15 mL, 40% in H_2O, 1.0 equiv) in CH_2Cl_2 (5.00 mL, 0.2 M) at 0 °C for 30 minutes. Following this, **PC1** was afforded in 59% yield (540 mg).

IR (Diamond-ATR, neat) ṽ (cm^{-1}) 2960, 2872, 2175, 2046, 1984, 1615, 1495, 829.

^{1}H NMR (600 MHz, $CDCl_3$) δ 8.92 (dd, *J* = 8.7, 2.3 Hz, 1H), 8.51 (d, *J* = 2.3 Hz, 1H), 8.11 (s, 1H), 7.91 (dd, *J* = 8.5, 3.2 Hz, 2H), 7.87 (d, *J* = 8.2 Hz, 1H), 7.60 (d, *J* = 8.7 Hz, 1H), 7.57 (d, *J* = 2.0 Hz, 1H), 7.48 (d, *J* = 8.7 Hz, 1H), 7.42 (d, *J* = 2.0 Hz, 1H), 7.41 – 7.37 (m, 1H), 7.36 – 7.32 (m, 3H), 7.21 (dddd, *J* = 8.4, 6.9, 3.4, 1.3 Hz, 2H), 1.96 – 1.86 (m, 8H), 1.63 (s, 9H), 1.41 (s, 9H), 1.23 – 1.10 (m, 16H), 0.74 (t, *J* = 6.9 Hz, 12H).

^{13}C NMR (151 MHz, $CDCl_3$) δ 178.1, 159.5, 157.4, 153.8, 152.9, 150.5 (d, $^2J_{C-P}$ = 9.3 Hz), 148.0 (d, $^2J_{C-P}$ = 9.5 Hz), 138.1, 135.1, 133.6, 133.6, 133.0, 132.8, 131.0, 130.9, 130.5, 129.9, 128.3, 127.6, 127.2, 127.1, 125.8, 125.7, 124.8, 124.3, 123.7, 123.4, 122.8, 122.8, 120.9, 118.9, 116.5, 114.1, 37.9, 35.6, 31.4, 31.0, 23.9 (d, $^2J_{C-P}$ = 15.1 Hz), 23.7 (d, $^3J_{C-P}$ = 4.9 Hz), 18.5 (d, $^1J_{C-P}$ = 47.3 Hz), 13.5.

^{31}P NMR (162 MHz, $CDCl_3$) δ 34.0, 6.5.

HRMS (ESI): m/z: $[M+H]^+$ calc'd for $C_{57}H_{71}O_6P_2$: 913.4726. Found: 913.4647.

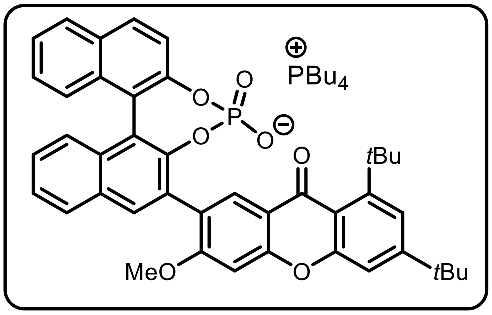

tetrabutylphosphonium 2-(6,8-di-tert-butyl-3-methoxy-9-oxo-9H-xanthen-2-yl)dinaphtho[2,1-d:1',2'-f][1,3,2]dioxaphosphepin-4-olate 4-oxide (PC2)

1,3-di-*tert*-butyl-7-(2,2'-dihydroxy-[1,1'-binaphthalen]-3-yl)-6-methoxy-9H-xanthen-9-one was prepared via the general deprotection procedure **C5** using **S15** (0.75 g, 1.06 mmol, 1.0 equiv) and 12 M HCl (2.64 mL, 31.7 mmol, 30.0 equiv) in 1,4-dioxane (10.6 mL, 0.1 M) at 60

°C for 1 hour. Following this, crude 1,3-di-*tert*-butyl-7-(2,2'-dihydroxy-[1,1'-binaphthalen]-3-yl)-6-methoxy-9H-xanthen-9-one was afforded in >95% yield as a yellow foam.

1,3-di-*tert*-butyl-7-(4-hydroxy-4-oxidodinaphtho[2,1-d:1',2'-f][1,3,2]dioxaphosphepin-2-yl)-6-methoxy-9H-xanthen-9-one was prepared using the general phosphorylation procedure **C5** using 1,3-di-*tert*-butyl-7-(2,2'-dihydroxy-[1,1'-binaphthalen]-3-yl)-6-methoxy-9H-xanthen-9-one (0.680 g, 1.09 mmol, 1.0 equiv) and $POCl_3$ (122 µL, 1.31 mmol, 1.2 equiv) in pyridine (2.2 mL, 0.5 M) at 85 °C for 4 hours. Following this, crude 1,3-di-*tert*-butyl-7-(4-hydroxy-4-oxidodinaphtho[2,1-d:1',2'-f][1,3,2]dioxaphosphepin-2-yl)-6-methoxy-9H-xanthen-9-one was afforded in 93% yield (694 mg) as a brown foam.

PC2 was prepared via the general salt formation procedure **C5** using 1,3-di-*tert*-butyl-7-(4-hydroxy-4-oxidodinaphtho[2,1-d:1',2'-f][1,3,2]dioxaphosphepin-2-yl)-6-methoxy-9H-xanthen-9-one (694 mg, 1.06 mmol, 1.0 equiv) and tetrabutyl(hydroxy)-1-phosphane (857 µL, 40% in H_2O, 1.0 equiv) in CH_2Cl_2 (5.3 mL, 0.2 M) at 0 °C for 30 minutes. Following this, **PC2** was afforded in 21% yield (200 mg).

IR (Diamond-ATR, neat) ṽ (cm^{-1}): 2958, 2931, 2871, 1707, 1587, 1266, 1219, 1099, 830.

^{1}H NMR (600 MHz, $CDCl_3$) δ 8.29 (s, 1H), 7.93 (s, 1H), 7.89 (d, *J* = 8.3 Hz, 1H), 7.86 (t, *J* = 8.3 Hz, 2H), 7.56 (d, *J* = 8.6 Hz, 1H), 7.54 (brs, 1H), 7.48 (d, *J* = 8.5 Hz, 1H), 7.40 (d, *J* = 8.6 Hz, 1H), 7.39 – 7.37 (m, 1H), 7.37 – 7.33 (m, 2H), 7.24 – 7.17 (m, 2H), 6.86 (s, 1H), 3.84 (s, 3H), 2.12 – 2.01 (m, 8H), 1.61 (s, 9H), 1.40 (s, 9H), 1.33 – 1.21 (m, 8H), 1.21 – 1.12 (m, 8H), 0.72 (at, *J* = 7.1 Hz, 12H).

^{13}C NMR (151 MHz, $CDCl_3$) δ 177.2, 163.3, 159.5, 156.8, 156.0, 152.4, 150.6 (d, $^2J_{C-P}$ = 9.5 Hz), 149.0 (d, $^2J_{C-P}$ = 9.1 Hz), 133.3, 133.0, 131.7, 131.0, 130.9, 130.7, 130.1, 129.8, 128.2, 128.1, 127.4, 127.3, 125.5, 125.5, 124.3, 124.1, 123.0, 122.8, 120.7, 119.1, 116.8, 114.0, 97.7, 56.7, 37.9, 35.5, 31.5, 31.0, 23.9 (d, $^2J_{C-P}$ = 15.7 Hz), 23.8 (d, $^3J_{C-P}$ = 4.9 Hz), 18.7 (d, $^1J_{C-P}$ = 46.6 Hz), 13.5.

^{31}P NMR (162 MHz, $CDCl_3$) δ 32.9, 5.0.

HRMS (ESI): m/z: $[M+H]^+$ calc'd for $C_{58}H_{73}O_7P_2^+$: 943.48319. Found: 943.4215.

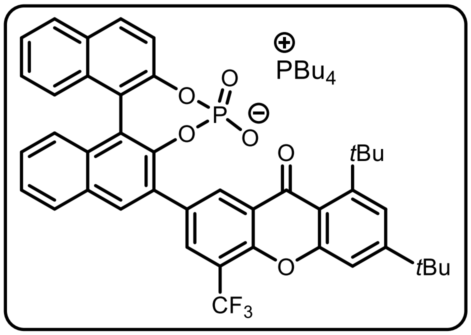

tetrabutylphosphonium 2-(6,8-di-tert-butyl-9-oxo-4-(trifluoromethyl)-9H-xanthen-2-yl)dinaphtho[2,1-d:1',2'-f][1,3,2]dioxaphosphepin-4-olate 4-oxide (PC3)

1,3-di-*tert*-butyl-7-(2,2'-dihydroxy-[1,1'-binaphthalen]-3-yl)-5-(trifluoromethyl)-9H-xanthen-9-one was prepared via the general deprotection procedure **C5** using **S16** (232 mg, 310 µmol, 1.0 equiv) and 12 M HCl (0.62 mL, 0.74 mmol, 30.0 equiv) in 1,4-dioxane (1.2 mL, 0.1 M) at 60 °C for 1 hour. Following this, crude 1,3-di-*tert*-butyl-7-(2,2'-dihydroxy-[1,1'-binaphthalen]-3-yl)-5-(trifluoromethyl)-9H-xanthen-9-one was afforded in in >95% yield as a yellow foam.

1,3-di-*tert*-butyl-7-(4-hydroxy-4-oxidodinaphtho[2,1-d:1',2'-f][1,3,2]dioxaphosphepin-2-yl)-5-(trifluoromethyl)-9H-xanthen-9-one was prepared using the general phosphorylation procedure **C5** using 1,3-di-*tert*-butyl-7-(2,2'-dihydroxy-[1,1'-binaphthalen]-3-yl)-5-(trifluoromethyl)-9H-xanthen-9-one (205 mg, 310 µmol, 1.0 equiv) and $POCl_3$ (34.7 µL, 372 µmol, 1.2 equiv) in pyridine (1.0 mL, 0.5 M) at 85 °C for 5 hours. Following this, crude 1,3-di-*tert*-butyl-7-(4-hydroxy-

4-oxidodinaphtho[2,1-d:1',2'-f][1,3,2]dioxaphosphepin-2-yl)-5-(trifluoromethyl)-9H-xanthen-9-one was afforded in 98% yield (220 mg) as a brown foam.

PC3 was prepared via the general salt formation procedure **C5** using 1,3-di-*tert*-butyl-7-(4-hydroxy-4-oxidodinaphtho[2,1-d:1',2'-f][1,3,2]dioxaphosphepin-2-yl)-5-(trifluoromethyl)-9H-xanthen-9-one (220 mg, 304 μmol, 1.0 equiv) and tetrabutyl(hydroxy)-1-phosphane (239 μL, 40% in H_2O, 1.0 equiv) in CH_2Cl_2 (1.5 mL, 0.2 M) at 0 °C for 30 minutes. Following this, **PC3** was afforded in 63% yield (589 mg).

IR (Diamond-ATR, neat) ṽ (cm^{-1}): 2962, 2363, 2344, 1595, 1442, 1350, 1240, 1147, 1014.

^{1}H NMR (600 MHz, $CDCl_3$) δ 9.12 (s, 1H), 8.79 (d, *J* = 1.4 Hz, 1H), 8.05 (s, 1H), 7.90 (d, *J* = 8.4 Hz, 2H), 7.87 (d, *J* = 8.3 Hz, 1H), 7.61 (d, *J* = 1.9 Hz, 1H), 7.56 (d, *J* = 8.7 Hz, 1H), 7.43 – 7.34 (m, 5H), 7.22 (t, *J* = 7.7 Hz, 2H), 3.13 – 3.08 (m, 8H), 1.61 (s, 9H), 1.51 – 1.43 (m, 8H), 1.42 (s, 9H), 1.24 (h, *J* = 7.2 Hz, 8H), 0.85 (t, *J* = 7.4 Hz, 12H).

^{13}C NMR (151 MHz, $CDCl_3$) δ 176.8, 159.0, 158.2, 153.1, 150.8, 150.0 (d, $^2J_{C-P}$ = 11.0 Hz), 147.1 ($^2J_{C-P}$ = 10.4 Hz), 134.4 (q, $^3J_{C-F}$ = 4.3 Hz), 134.3, 133.0, 132.4, 132.4, 132.2, 131.2, 131.0, 130.2, 128.3, 128.3, 127.3, 127.2, 127.1, 126.3, 126.0, 125.2, 124.6, 124.5, 123.9, 123.4 (ad, $^1J_{C-F}$ = 272.1 Hz), 122.5, 122.4, 121.6, 118.8, 117.8 (ad, $^2J_{C-F}$ = 32.6 Hz), 114.2, 37.9, 35.8, 31.3, 31.0, 24.0 (d, $^2J_{C-P}$ = 14.9 Hz), 23.9 (d, $^3J_{C-P}$ = 5.0 Hz), 18.7 (d, $^1J_{C-P}$ = 47.0 Hz), 13.6.

^{31}P NMR (162 MHz, $CDCl_3$) δ 33.0, 5.0

^{19}F NMR (376 MHz, $CDCl_3$) δ -61.5.

HRMS (ESI): m/z: $[M+H]^+$ calc'd for $C_{58}H_{70}O_6F_3P_2^+$: 981.45999. Found: 981.4568.

Synthesis of Me-PC1

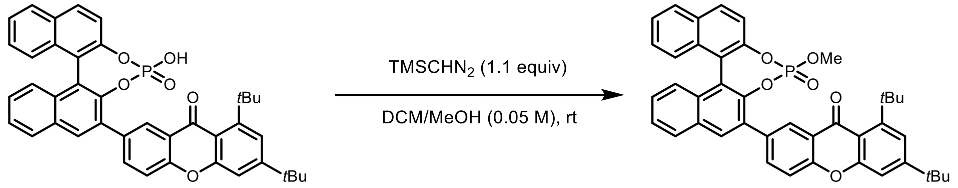

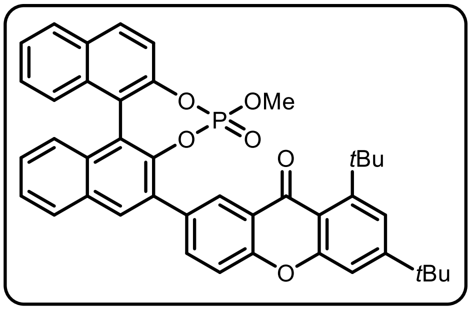

1,3-di-tert-butyl-7-(4-methoxy-4-oxidodinaphtho[2,1-d:1',2'-f][1,3,2]dioxaphosphepin-2-yl)-9H-xanthen-9-one (Me-PC1)

An oven-dried 25 mL round-bottom flask equipped with magnetic stir bar was added with 1,3-di-*tert*-butyl-7-(4-hydroxy-4-oxidodinaphtho[2,1-d:1',2'-f][1,3,2]dioxaphos phepin-2-yl)-9H-xanthen-9-one (65.5 mg, 0.10 mmol, 1.0 equiv). The flask was sealed with rubber septum, evacuated and backfilled with nitrogen three times and placed under a nitrogen atmosphere. CH_2Cl_2/MeOH (1:1, 0.05 M, 2 mL) was added followed by (trimethylsilyl) diazomethane (0.6 M solution in hexane, 183 µL, 0.11 mmol, 1.1 equiv). After stirring for 2 hours, the mixture was concentrated under reduced pressure, and the crude product was purified by silica gel chromatography (hexanes/EtOAc 75/25) to afford **Me-PC1** in 28% yield (18.5 mg) as a white amorphous solid.

IR (Diamond-ATR, neat) ṽ (cm^{-1}): 2959, 1654, 1607, 1298, 1048, 962, 739.

Major Diastereomer:

^{1}H NMR (600 MHz, $CDCl_3$) δ 8.63 (d, J = 2.3 Hz, 1H), 8.23 (s, 1H), 8.12 (dd, J = 8.7, 2.2 Hz, 1H), 8.07 (d, J = 8.8 Hz, 1H), 8.00 (d, J = 8.4 Hz, 2H), 7.63 – 7.58 (m, 2H), 7.56 – 7.52 (m, 3H), 7.45 – 7.40 (m, 2H), 7.37 – 7.33 (m, 3H), 3.29 (d, $^3J_{H\text{-}P}$ = 12.0 Hz, 3H), 1.64 (s, 9H), 1.43 (s, 9H).

^{13}C NMR (151 MHz, $CDCl_3$) δ 177.6, 159.5, 157.9, 154.2, 153.3, 146.3 ($^2J_{C\text{-}P}$ = 8.3Hz), 144.9 ($^2J_{C\text{-}P}$ = 10.6 Hz), 135.9, 132.6, 132.6, 132.3 ($^3J_{C\text{-}P}$ = 3.0 Hz), 132.1, 132.0, 131.9, 131.7, 131.7, 128.7, 128.7, 128.6, 128.2, 127.2, 127.1, 127.1, 126.5, 126.0, 124.0, 122.4($^3J_{C\text{-}P}$ = 3.0 Hz), 121.9($^3J_{C\text{-}P}$ = 3.0 Hz), 121.2, 120.8 ($^3J_{C\text{-}P}$ = 3.0 Hz), 118.9, 117.1, 114.1, 55.3 ($^2J_{C\text{-}P}$ = 4.5 Hz), 37.9, 35.7, 31.4, 31.0.

^{31}P NMR (162 MHz, $CDCl_3$) δ 3.5.

Minor Diastereomer:

^{1}H NMR (600 MHz, $CDCl_3$): δ 8.62 (d, J = 2.4 Hz, 1H), 8.32 (dt, J = 8.6, 1.6 Hz, 1H), 8.21 (s, 1H), 8.04 (d, J = 9.0 Hz, 1H), 7.97 (d, J = 9.8 Hz, 2H), 7.58 (d, J = 1.9 Hz, 1H), 7.52 – 7.49 (m, 3H), 7.48 – 7.45 (m, 2H), 7.38 (s, 1H), 7.33 (s, 1H), 7.32 – 7.30 (m, 2H), 3.84 (d, $^3J_{H\text{-}P}$ = 11.3 Hz, 3H), 1.61 (s, 9H), 1.42 (s, 9H).

^{13}C NMR (151 MHz, $CDCl_3$) δ 177.8, 159.4, 157.5, 154.3, 153.1, 147.4($^2J_{C\text{-}P}$ = 10.6 Hz), 143.9 ($^2J_{C\text{-}P}$ = 8.3Hz), 135.7, 132.7($^3J_{C\text{-}P}$ = 3.0 Hz), 132.6, 132.4, 132.1, 132.1, 131.9, 131.8, 131.4, 128.7, 128.6, 128.6, 128.2, 127.3, 127.1, 127.0, 126.5, 126.0, 123.9, 122.5($^3J_{C\text{-}P}$ = 3.0 Hz), 121.5($^3J_{C\text{-}P}$ = 3.0 Hz), 121.0, 120.1 ($^3J_{C\text{-}P}$ = 3.0 Hz), 119.0, 116.9, 114.0, 55.8($^2J_{C\text{-}P}$ = 4.5 Hz), 37.8, 35.7, 31.3, 31.0.

^{31}P NMR (162 MHz, $CDCl_3$) δ 2.7.

HRMS (ESI): m/z: $[M+H]^+$ calc'd for $C_{42}H_{38}O_6P^+$: 669.24063. Found: 669.2408.

ii) NMR Spectra of Catalyst Intermediates

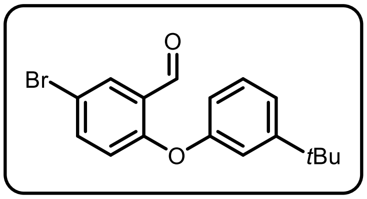

5-bromo-2-(3-(tert-butyl)phenoxy)benzaldehyde (S1)

^{1}H NMR (600 MHz, $CDCl_3$)

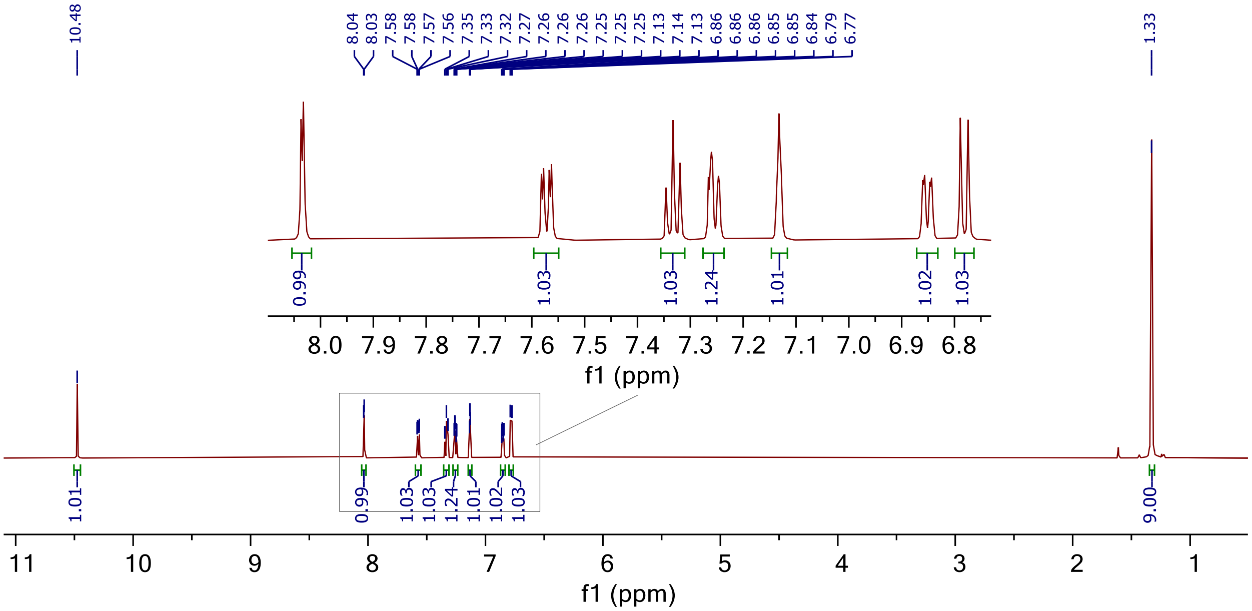

^{13}C NMR (151 MHz, $CDCl_3$)

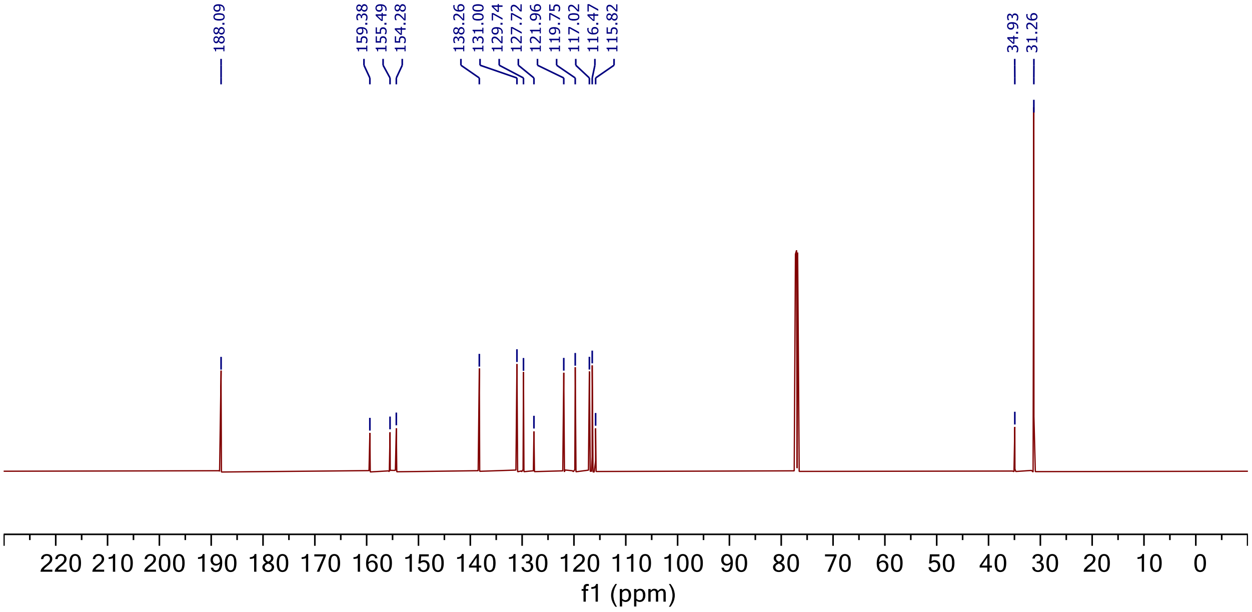

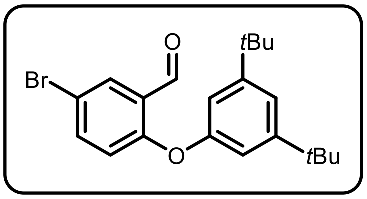

5-bromo-2-(3,5-di-tert-butylphenoxy)benzaldehyde (S2)

^{1}H NMR (600 MHz, $CDCl_3$)

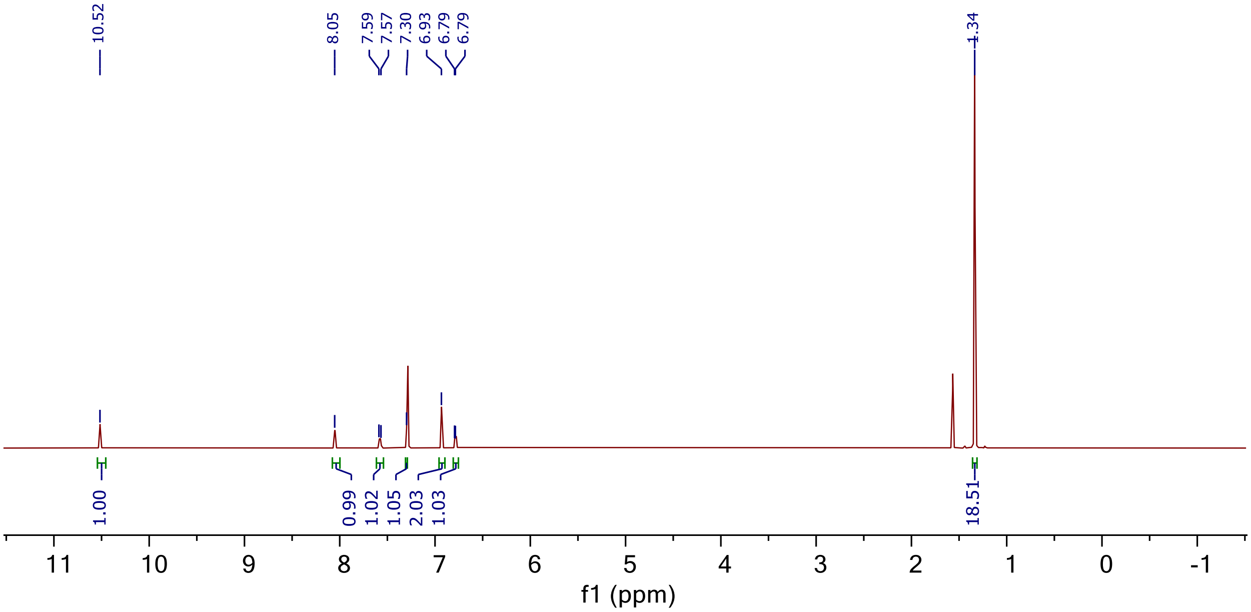

^{13}C NMR (151 MHz, $CDCl_3$)

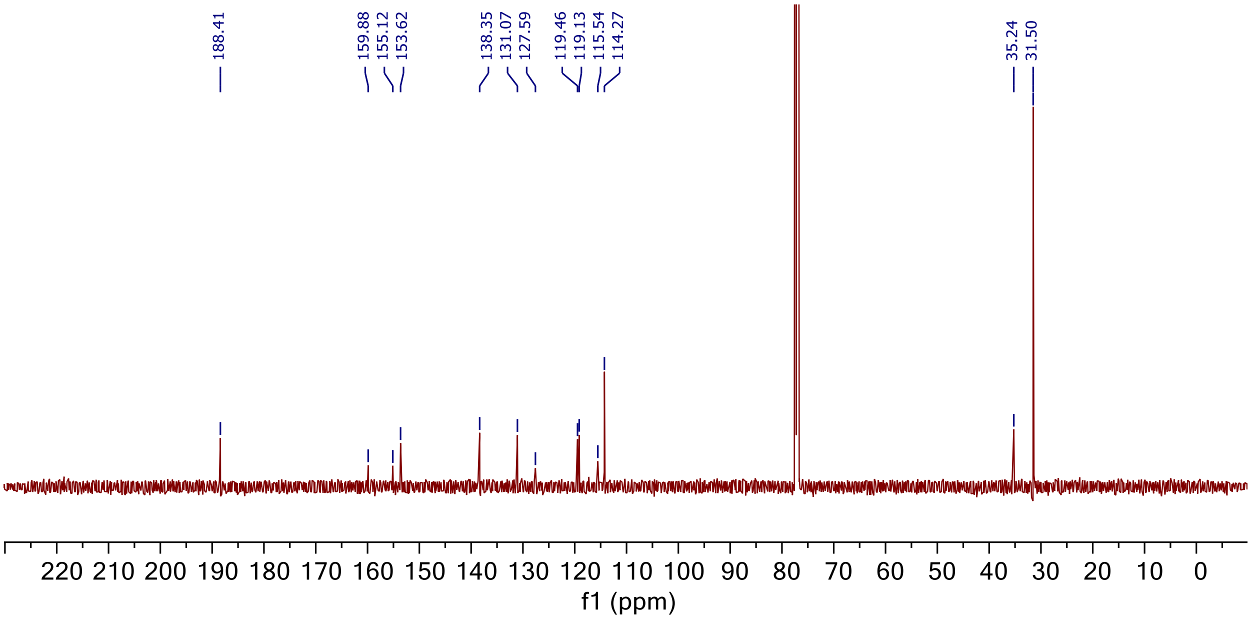

5-bromo-2-(3,5-di-tert-butylphenoxy)-4-methoxy benzaldehyde (S3)

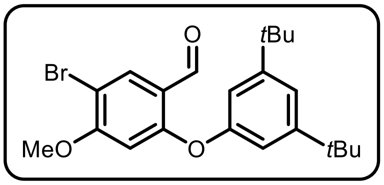

^{1}H NMR (600 MHz, $CDCl_3$)

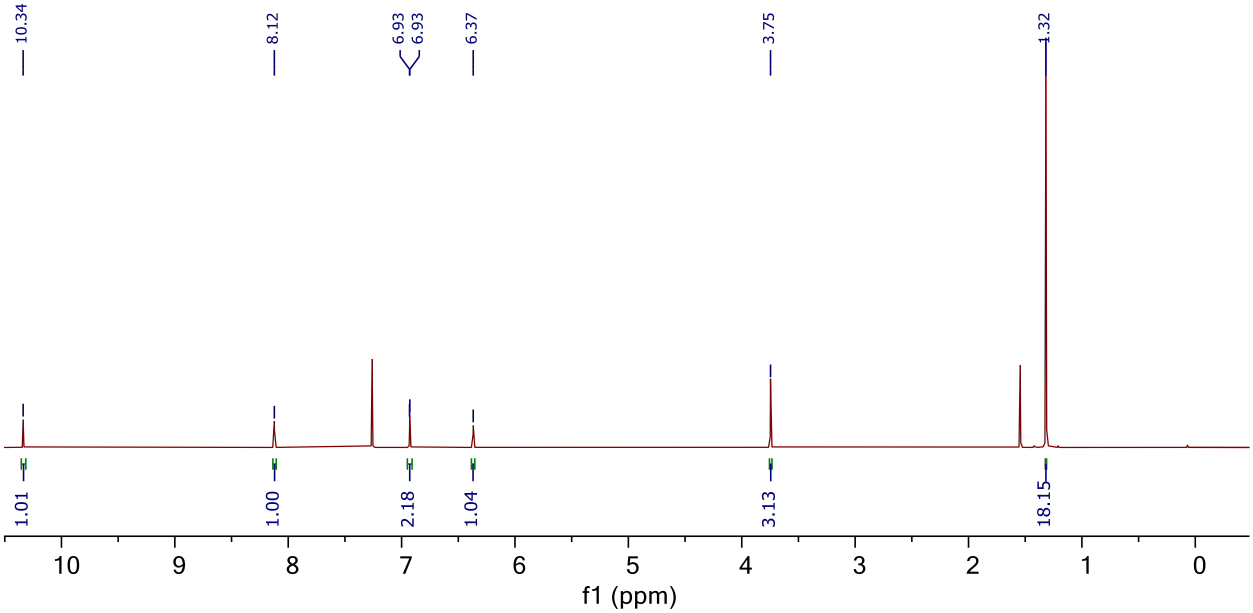

^{13}C NMR (151 MHz, $CDCl_3$)

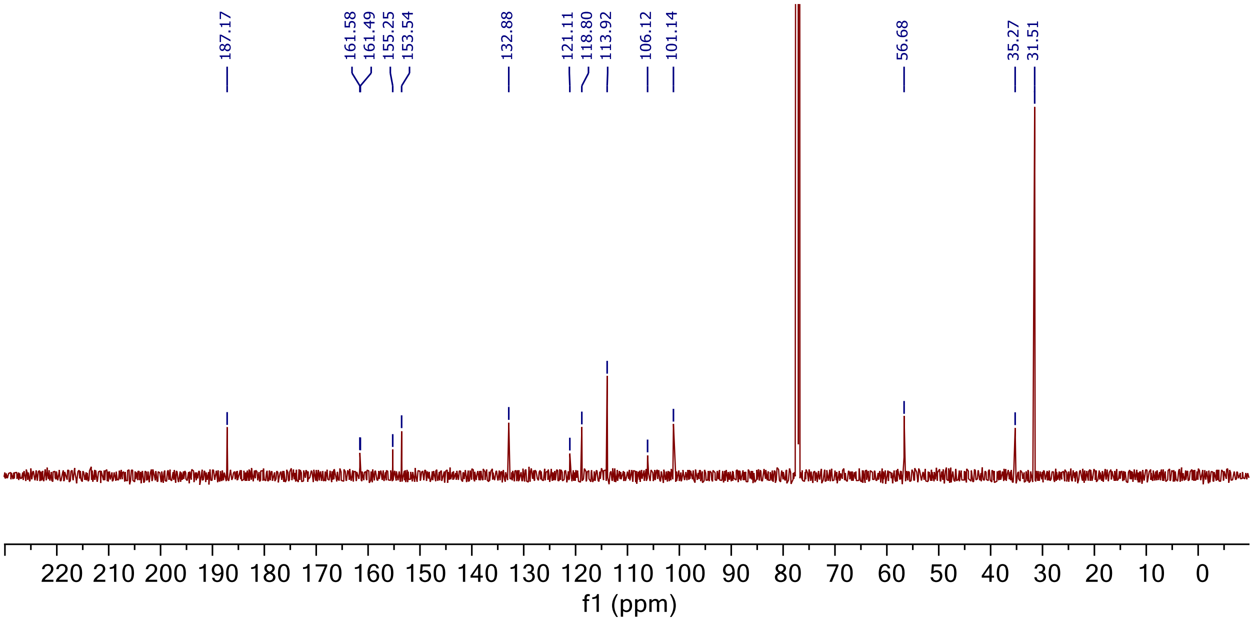

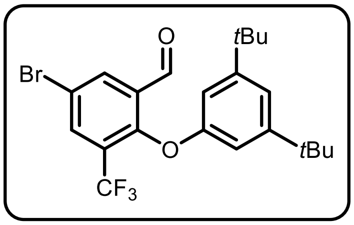

5-bromo-2-(3,5-di-tert-butylphenoxy)-3-(trifluoromethyl) benzaldehyde (S4)

^{1}H NMR (600 MHz, $CDCl_3$)

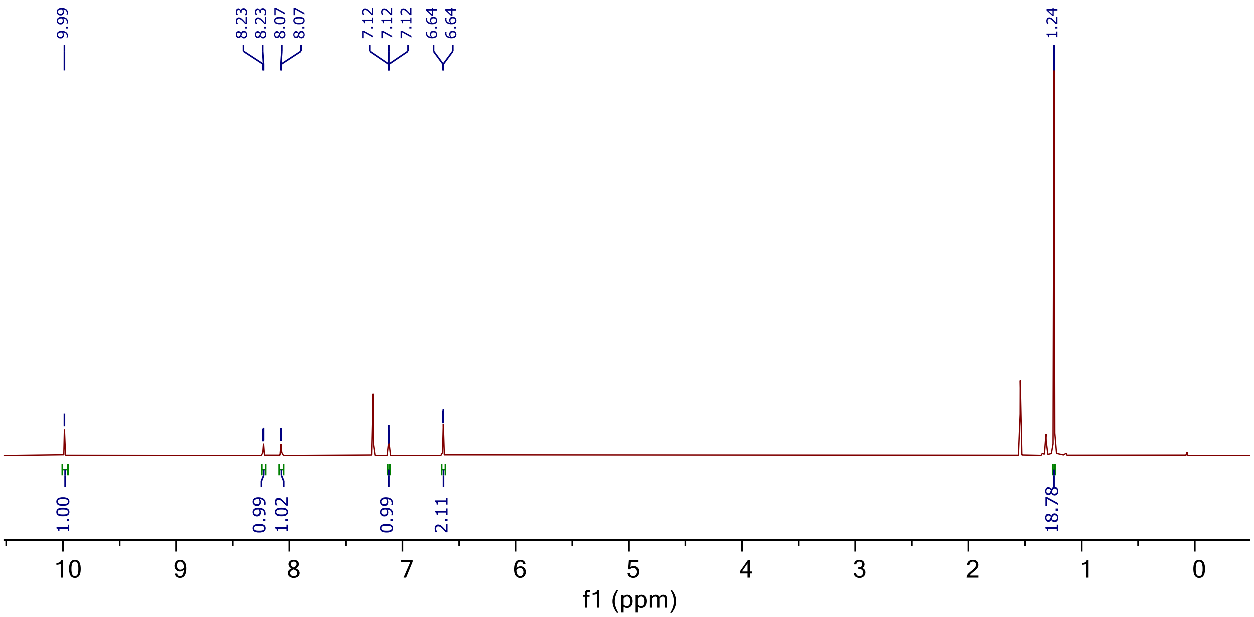

^{13}C NMR (151 MHz, $CDCl_3$)

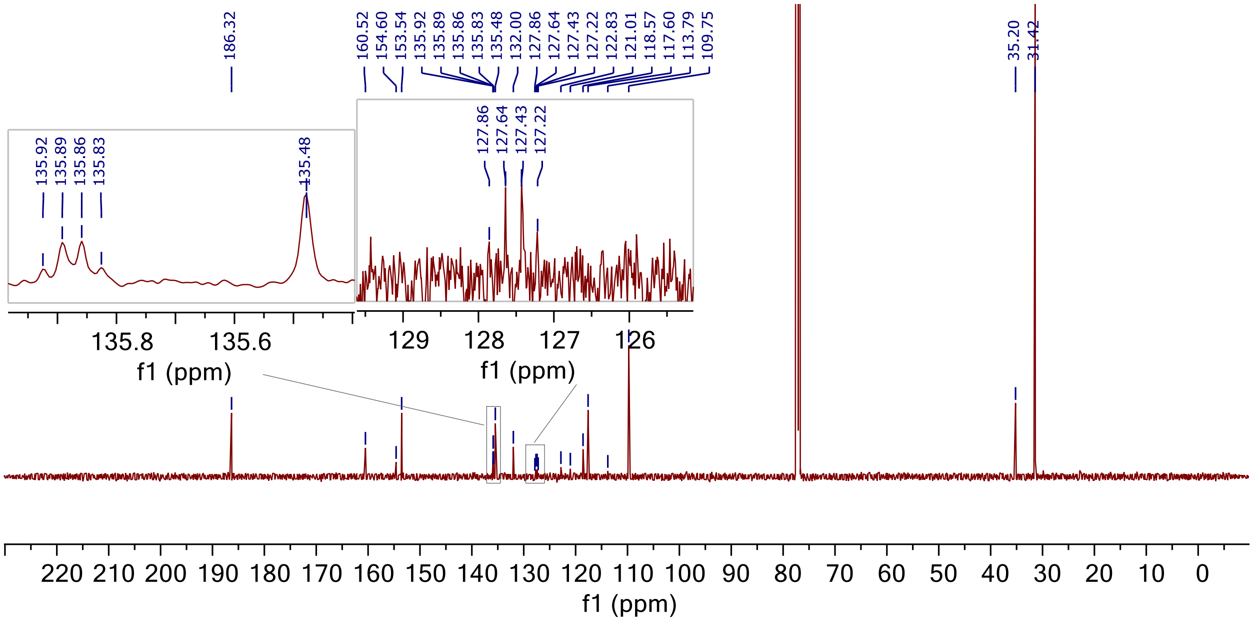

^{19}F NMR (376 MHz, $CDCl_3$)

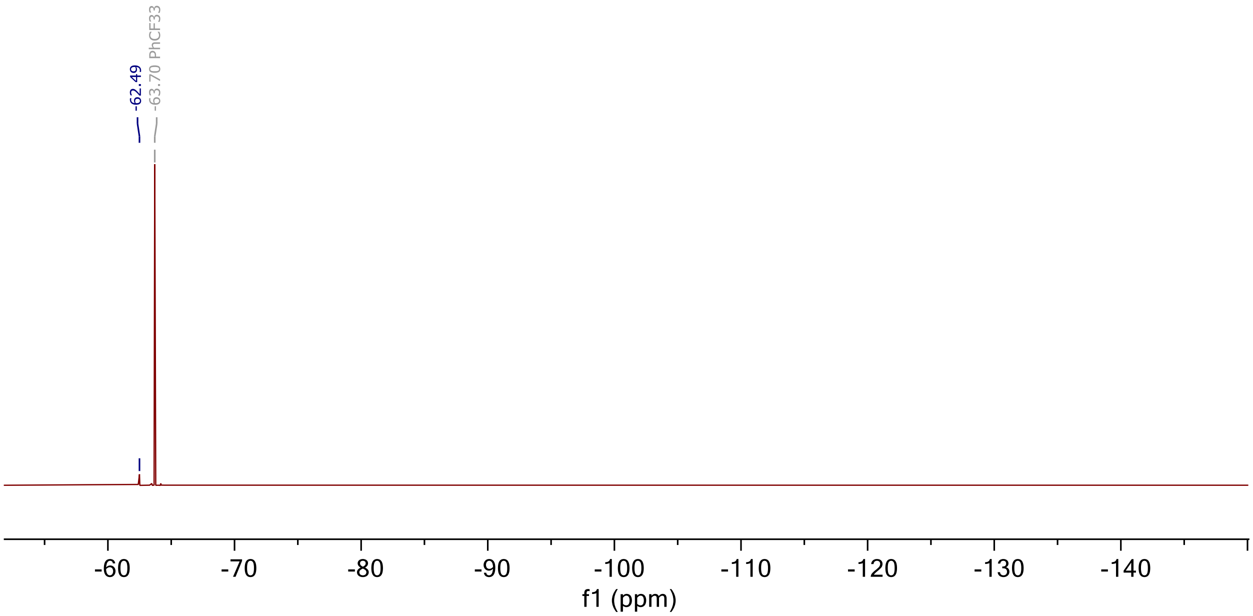

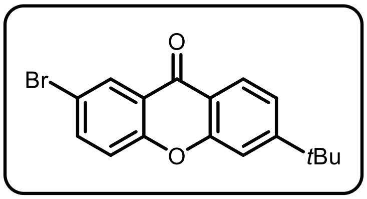

2-bromo-6-(tert-butyl)-9H-xanthen-9-one (S5)

^{1}H NMR (600 MHz, $CDCl_3$)

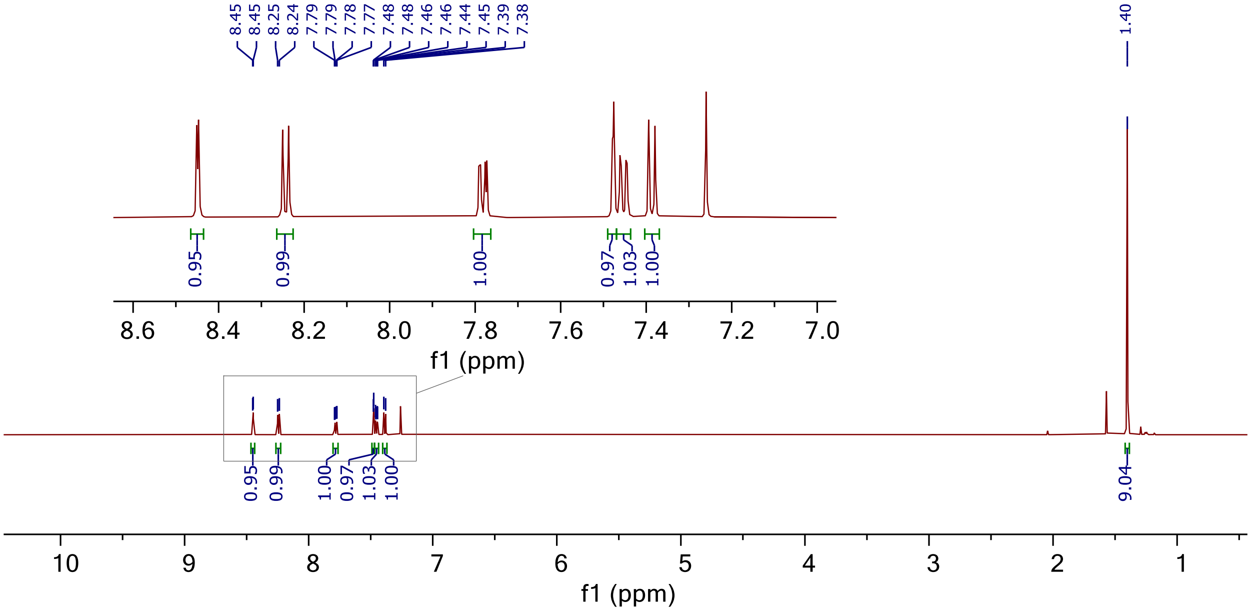

^{13}C NMR (151 MHz, $CDCl_3$)

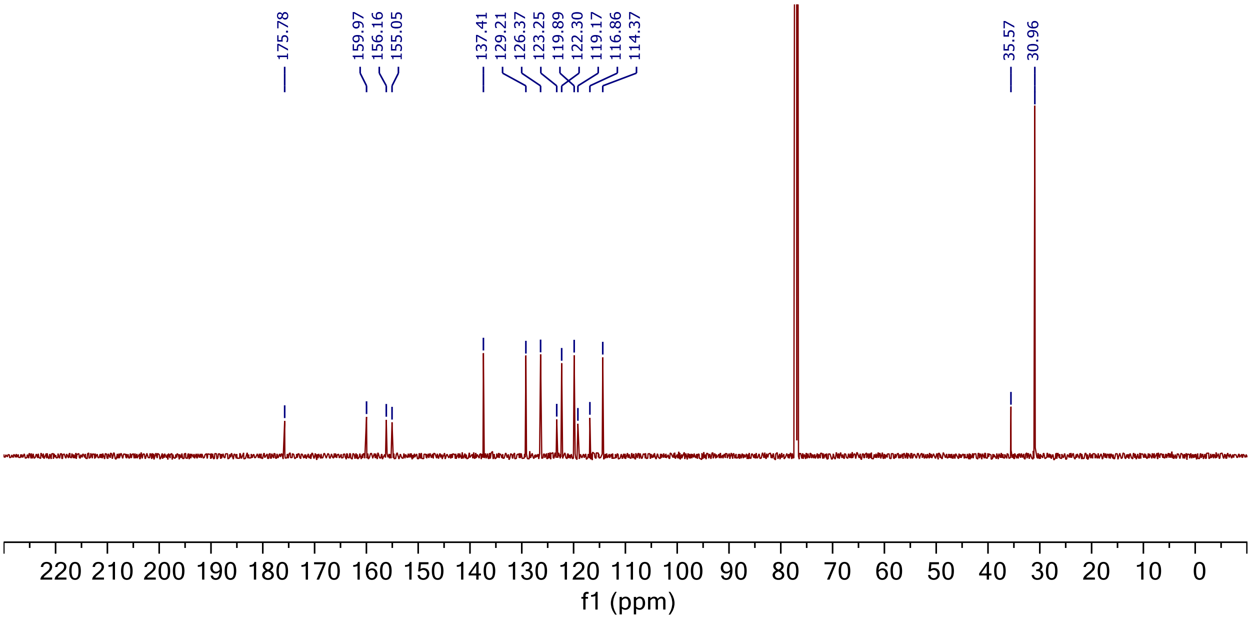

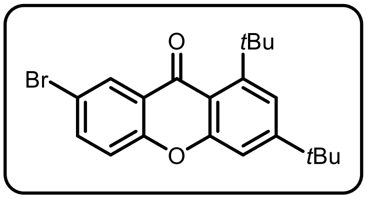

7-bromo-1,3-di-tert-butyl-9H-xanthen-9-one (S6)

^{1}H NMR (600 MHz, $CDCl_3$)

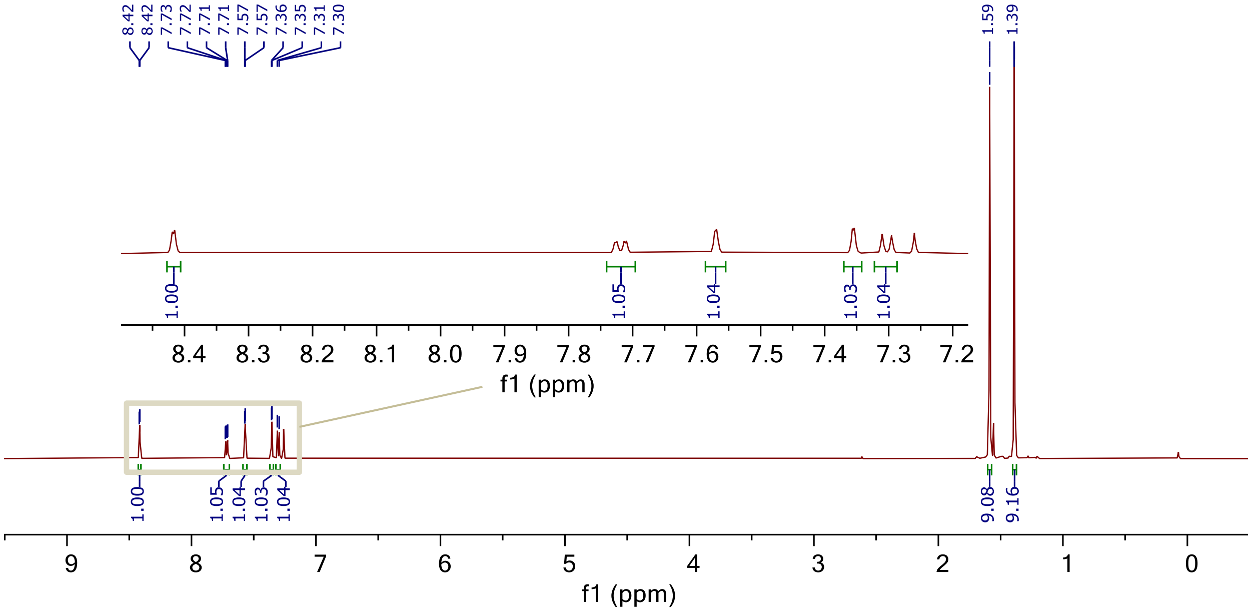

^{13}C NMR (151 MHz, $CDCl_3$)

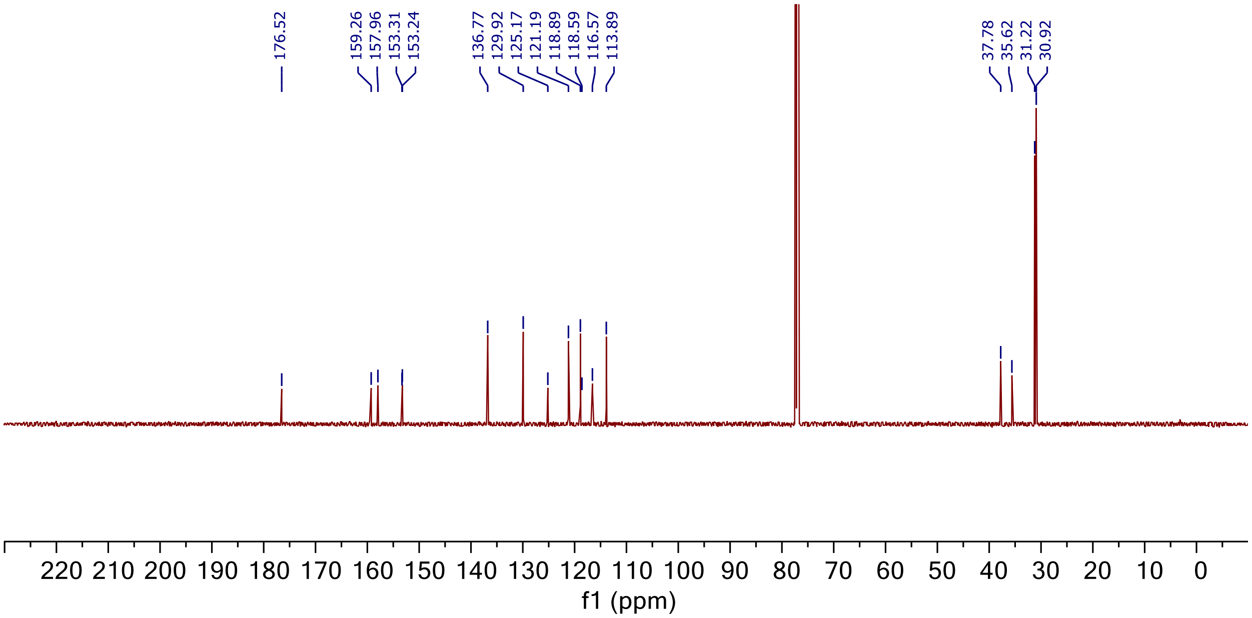

7-bromo-1,3-di-tert-butyl-6-methoxy-9H-xanthen-9-one (S7)

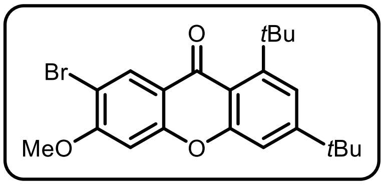

^{1}H NMR (600 MHz, $CDCl_3$)

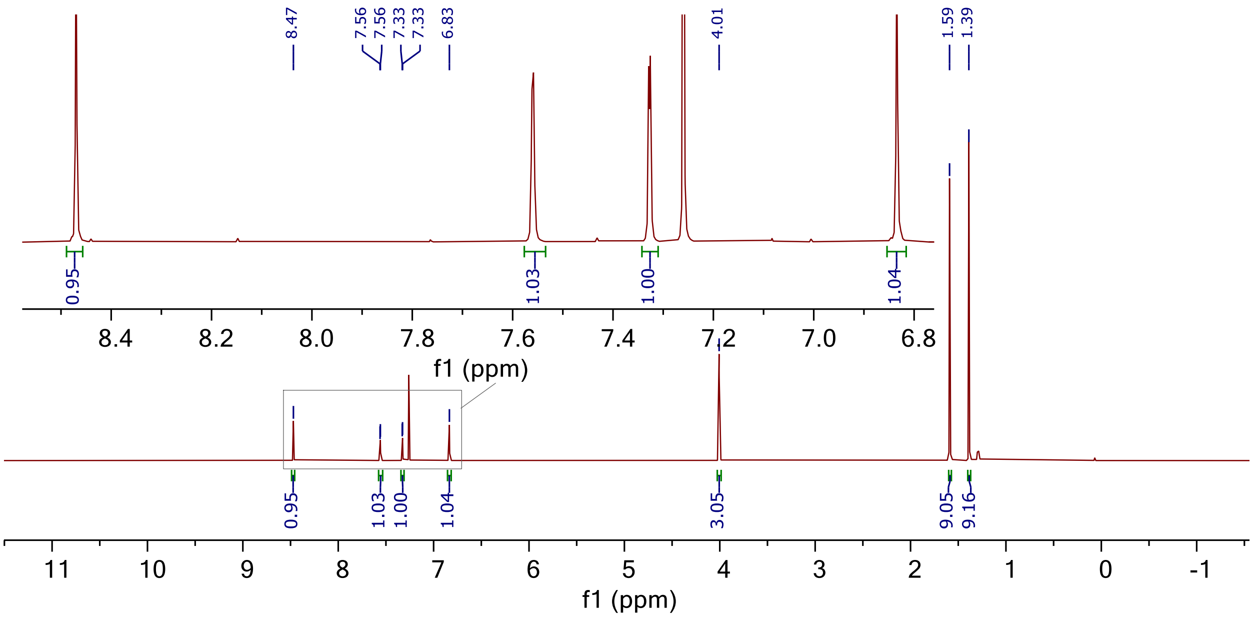

^{13}C NMR (151 MHz, $CDCl_3$)

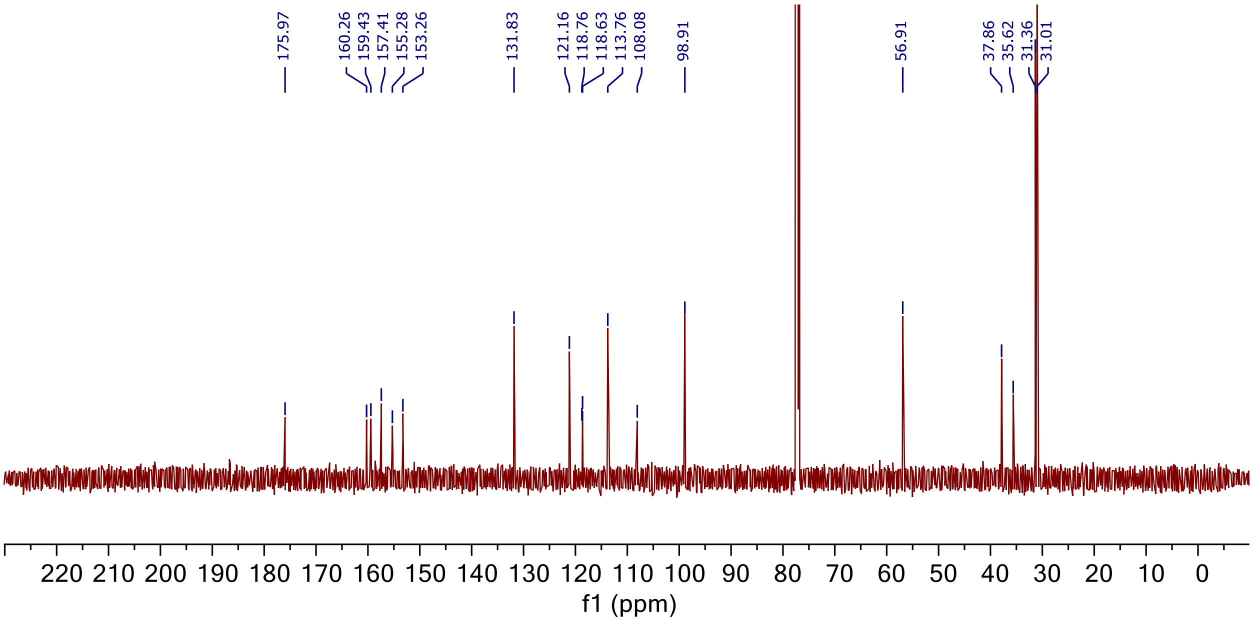

7-bromo-1,3-di-tert-butyl-5-(trifluoromethyl)-9H-xanthen-9-one (S8)

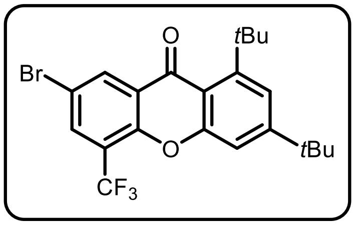

^{1}H NMR (600 MHz, $CDCl_3$)

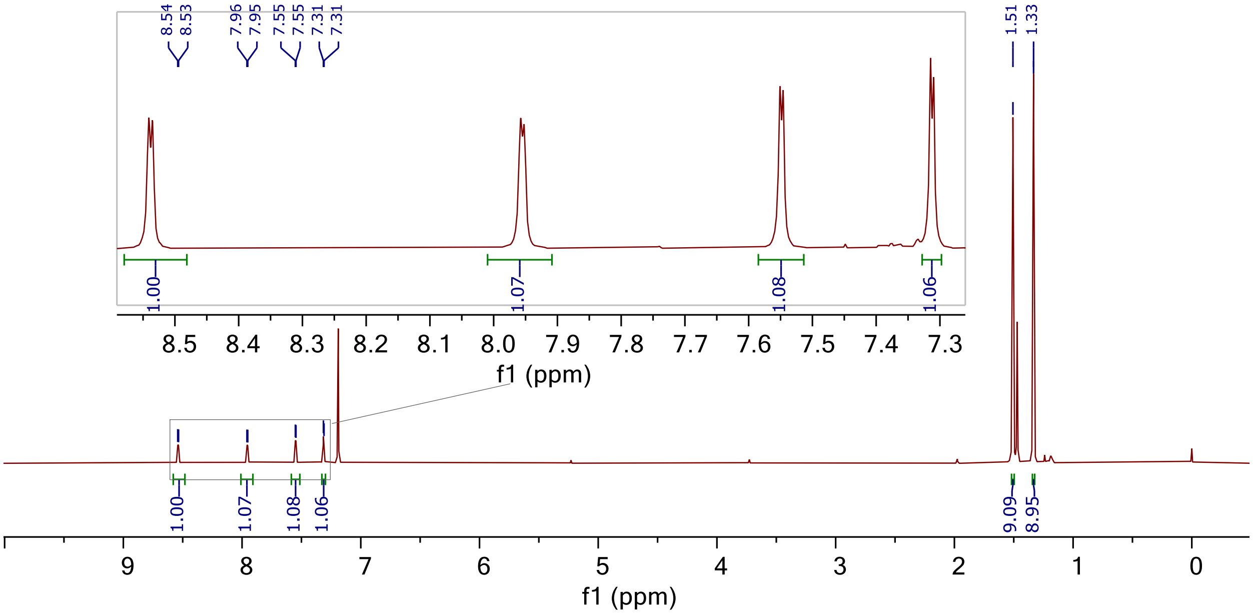

^{13}C NMR (151 MHz, $CDCl_3$)

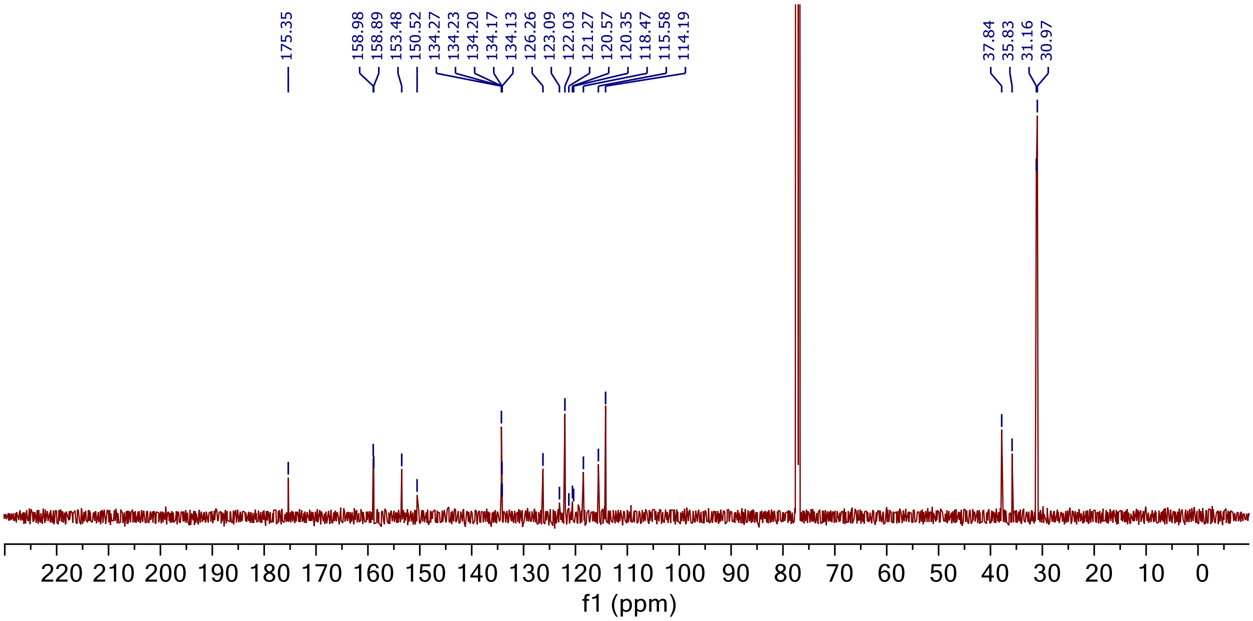

19F NMR (376 MHz, $CDCl_3$)

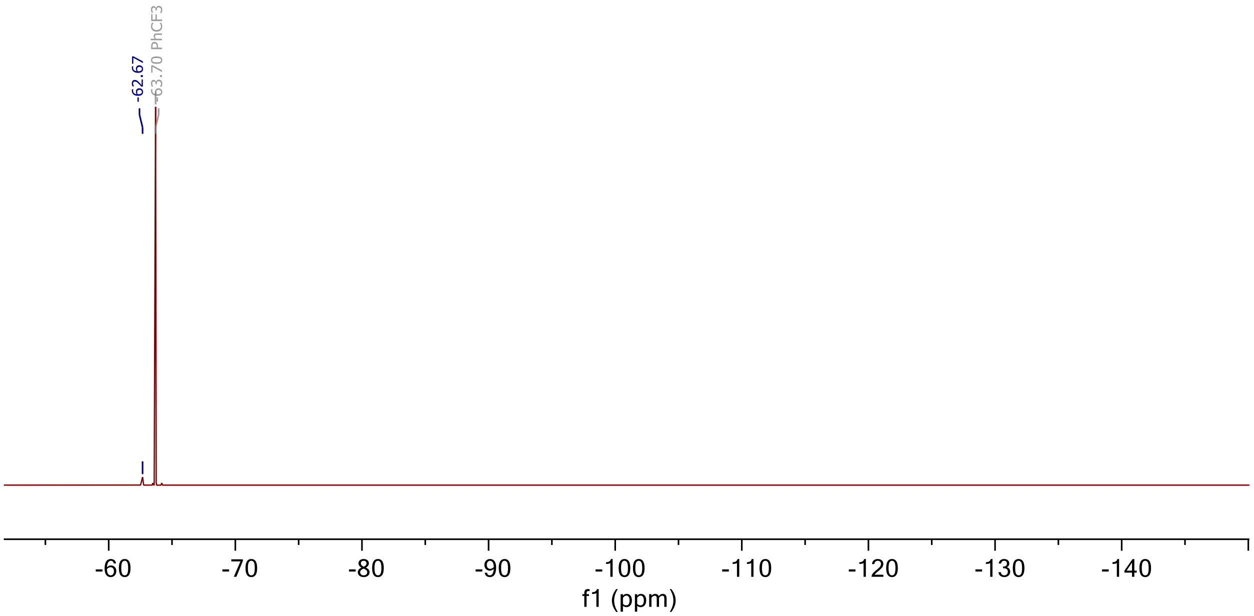

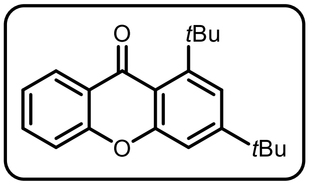

1,3-di-tert-butyl-9H-xanthen-9-one (S9)

^{1}H NMR (600 MHz, $CDCl_3$)

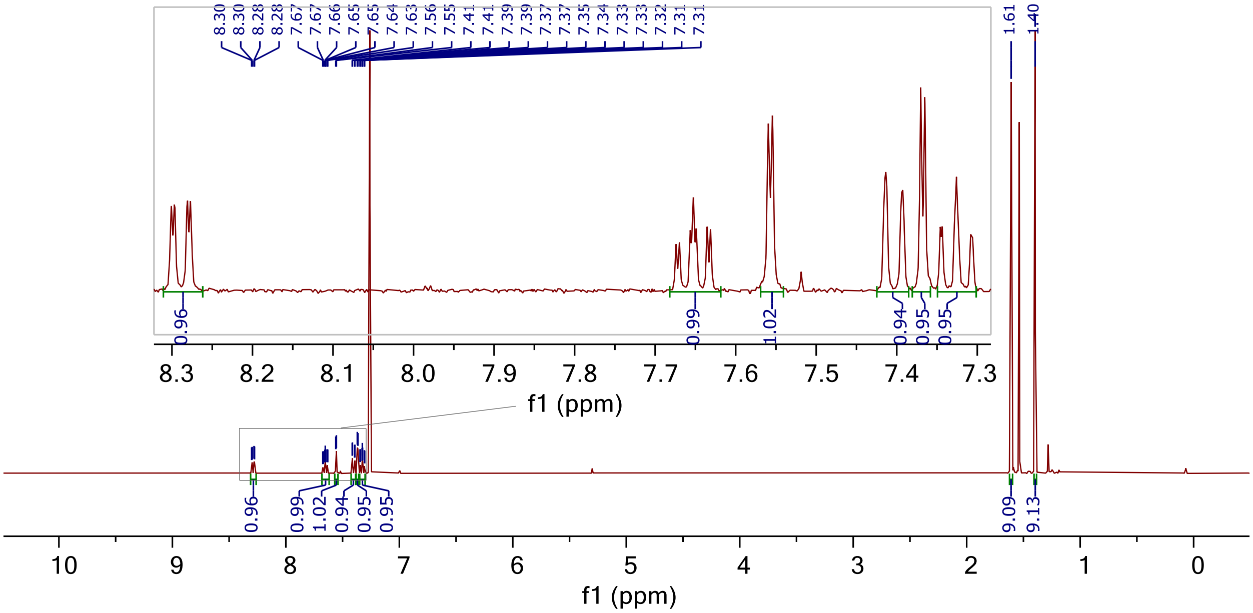

^{13}C NMR (151 MHz, $CDCl_3$)

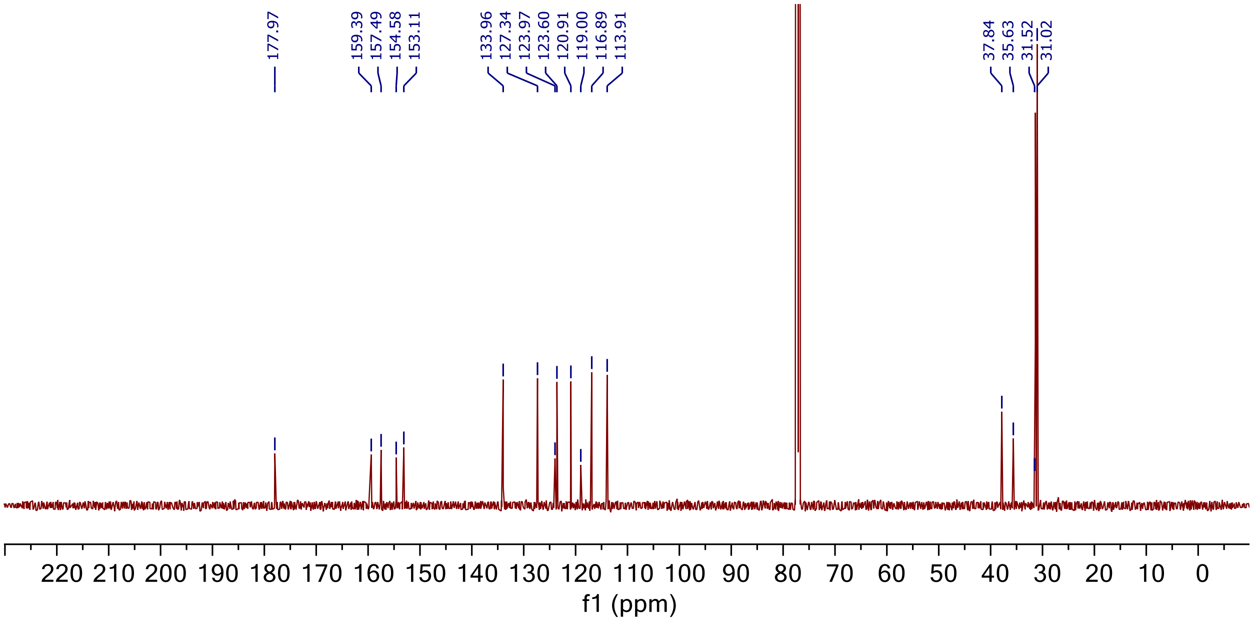

1,3-di-tert-butyl-6-methoxy-9H-xanthen-9-one (S10)

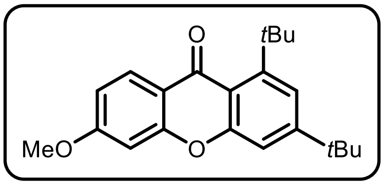

¹H NMR (600 MHz, $CDCl_3$)

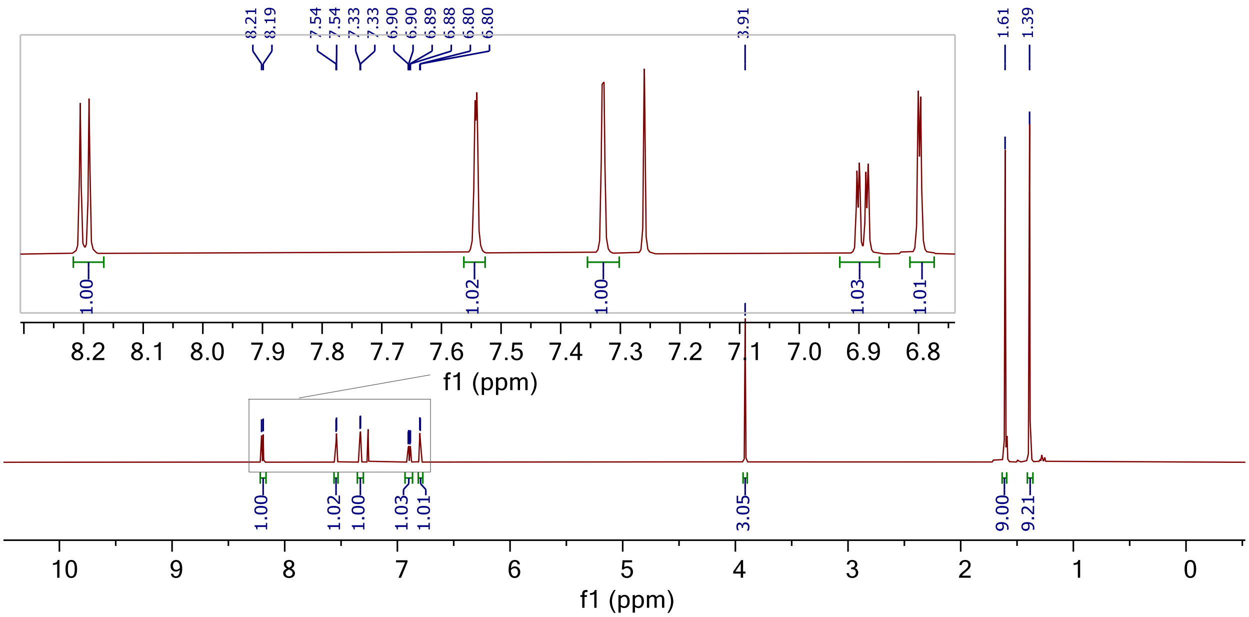

¹³C NMR (151 MHz, $CDCl_3$)

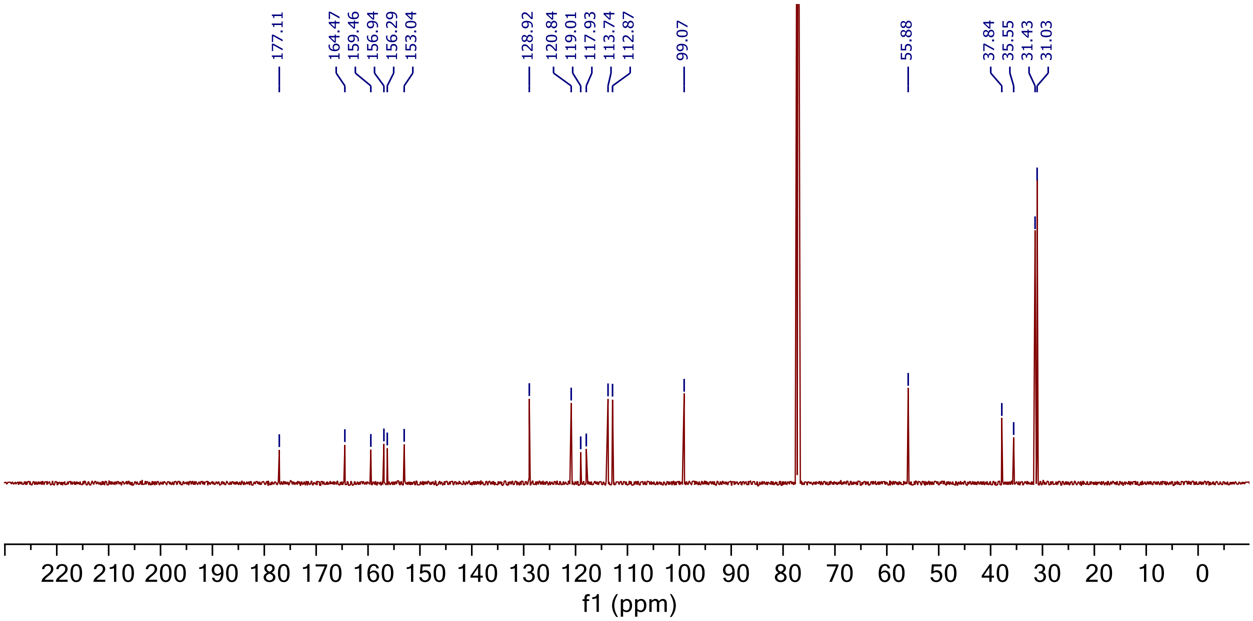

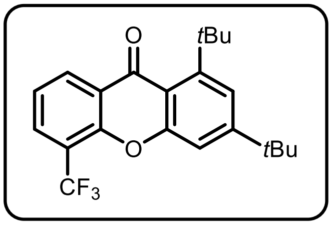

1,3-di-tert-butyl-5-(trifluoromethyl)-9H-xanthen-9-one (S11)

^{1}H NMR (600 MHz, $CDCl_3$)

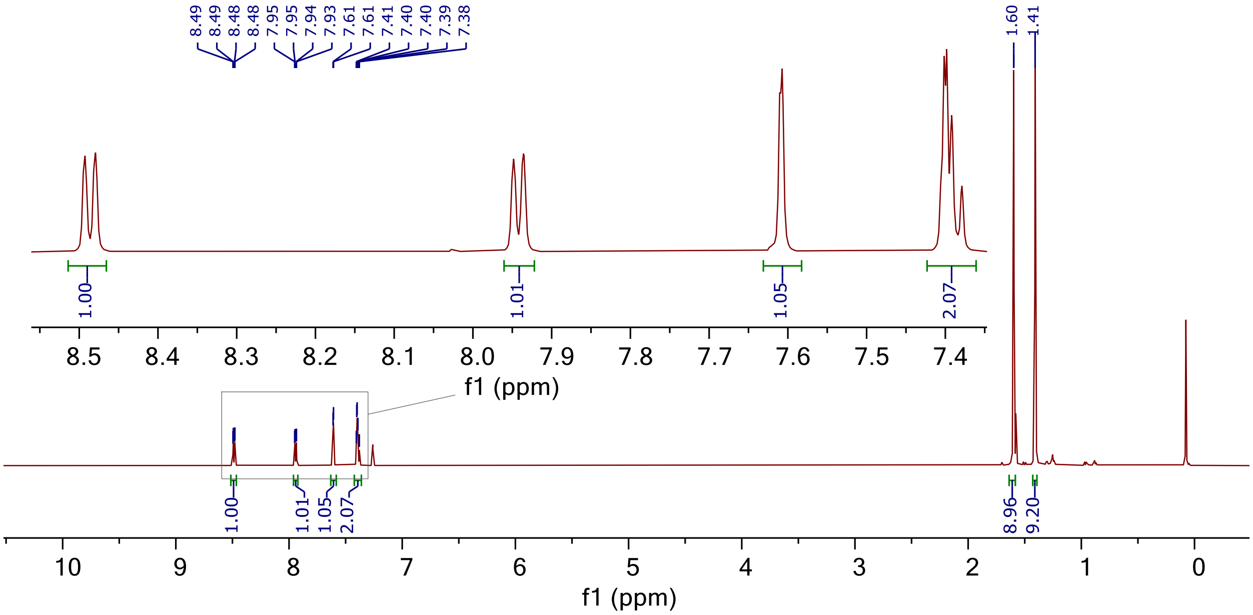

^{13}C NMR (151 MHz, $CDCl_3$)

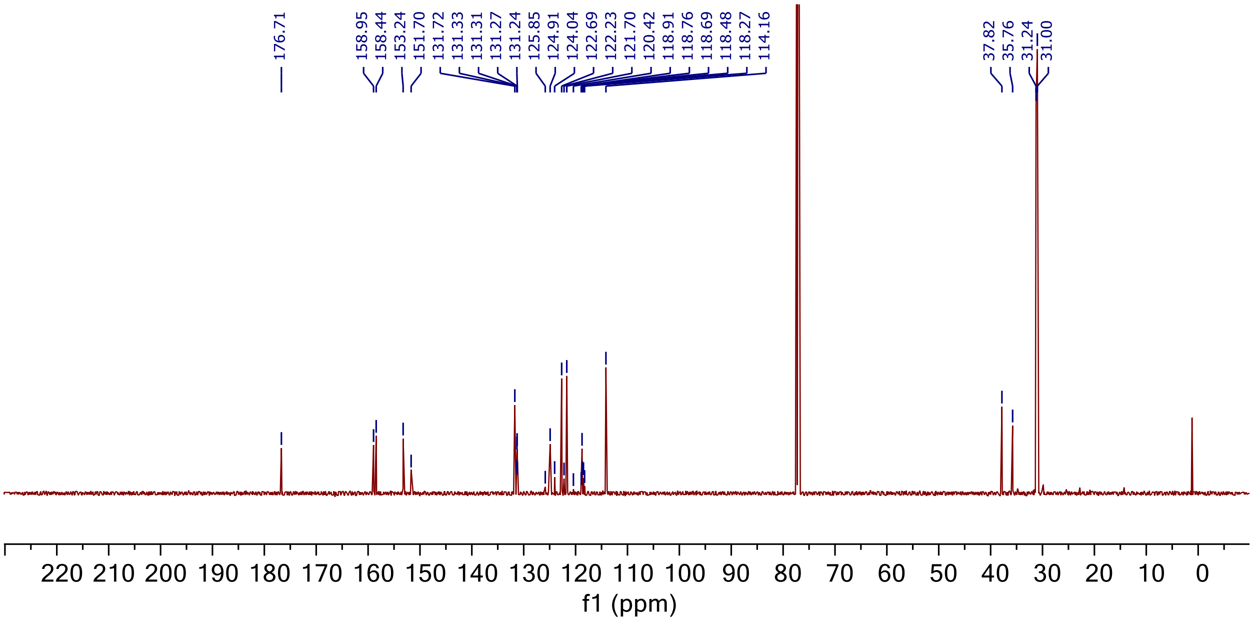

^{19}F NMR (376 MHz, $CDCl_3$)

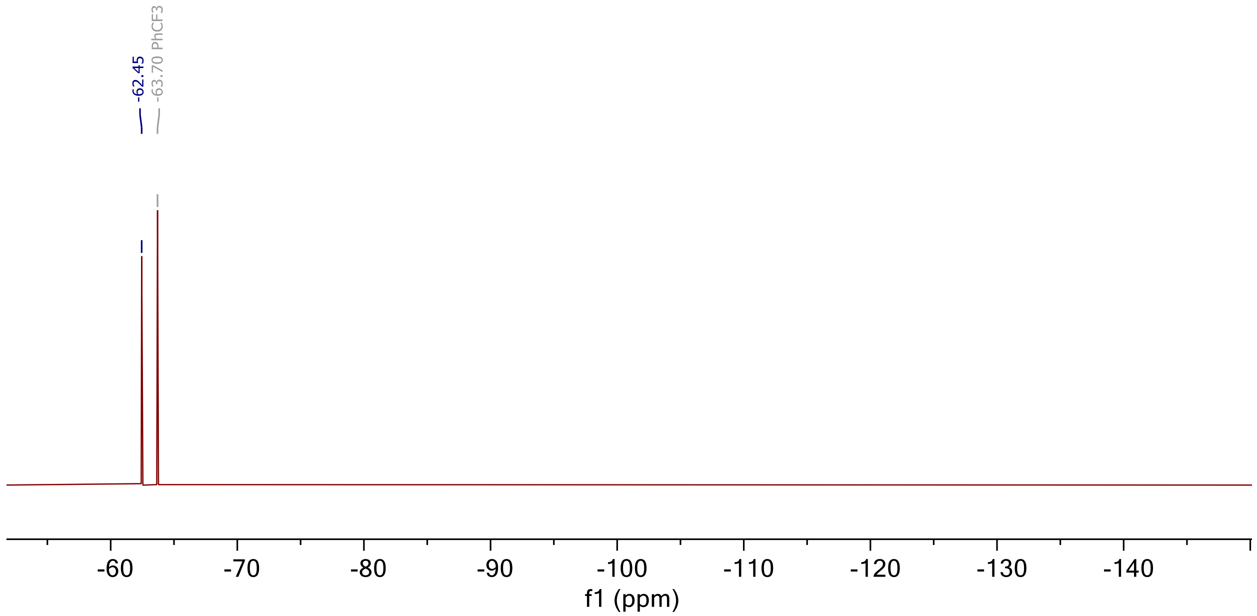

2-(2,2'-bis(methoxymethoxy)-[1,1'-binaphthalen]-3-yl)-9H-xanthen-9-one (S12)

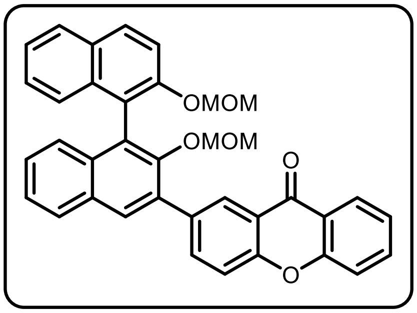

^{1}H NMR (600 MHz, $CDCl_3$)

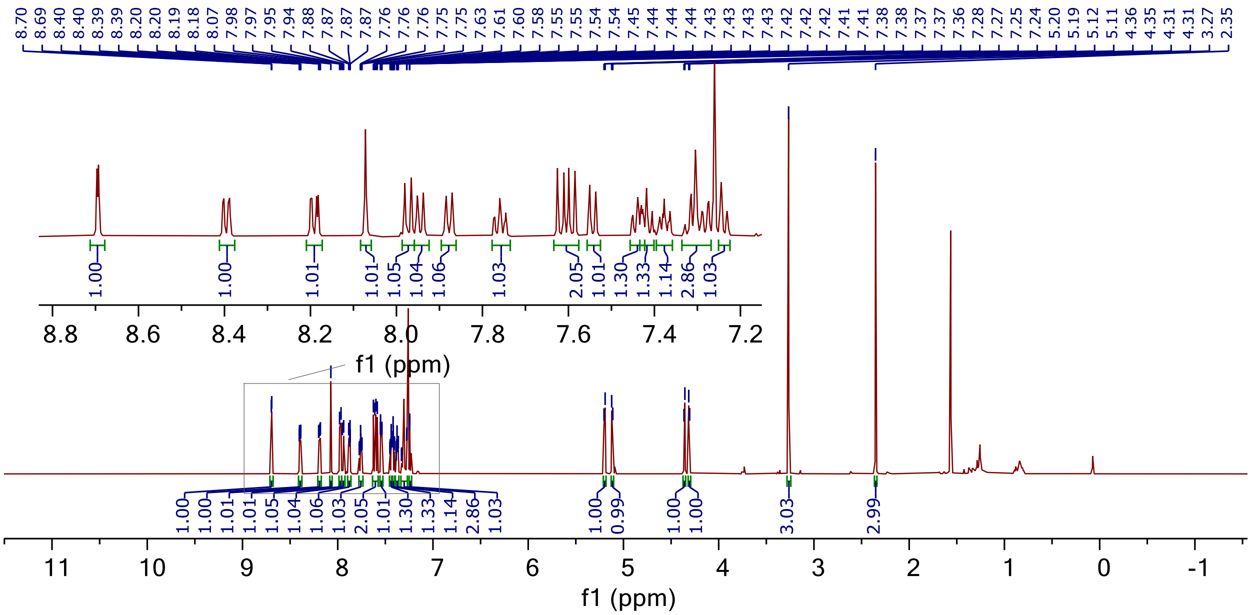

^{13}C NMR (151 MHz, $CDCl_3$)

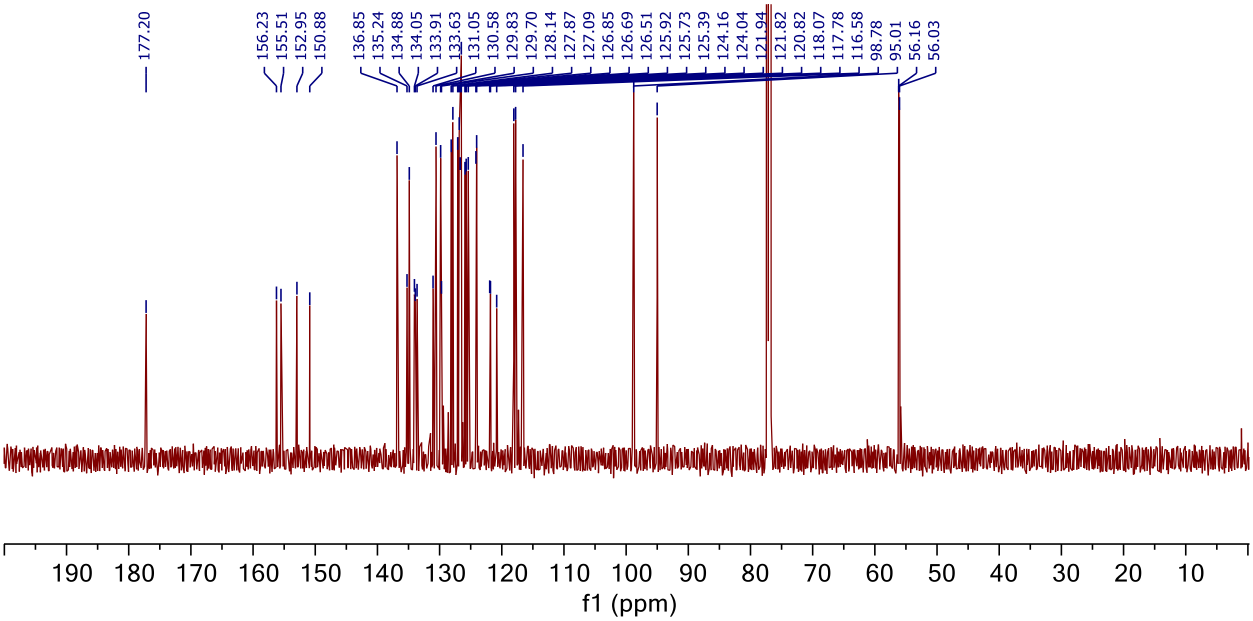

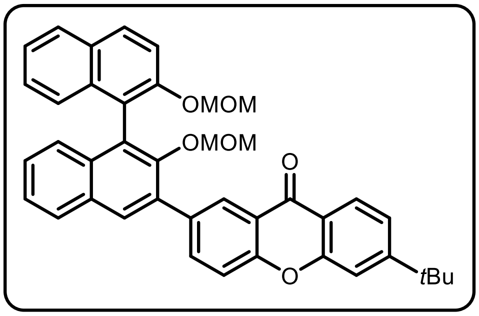

2-(2,2'-bis(methoxymethoxy)-[1,1'-binaphthalen]-3-yl)-6-(tert-butyl)-9H-xanthen-9-one (S13)

^{1}H NMR (600 MHz, $CDCl_3$)

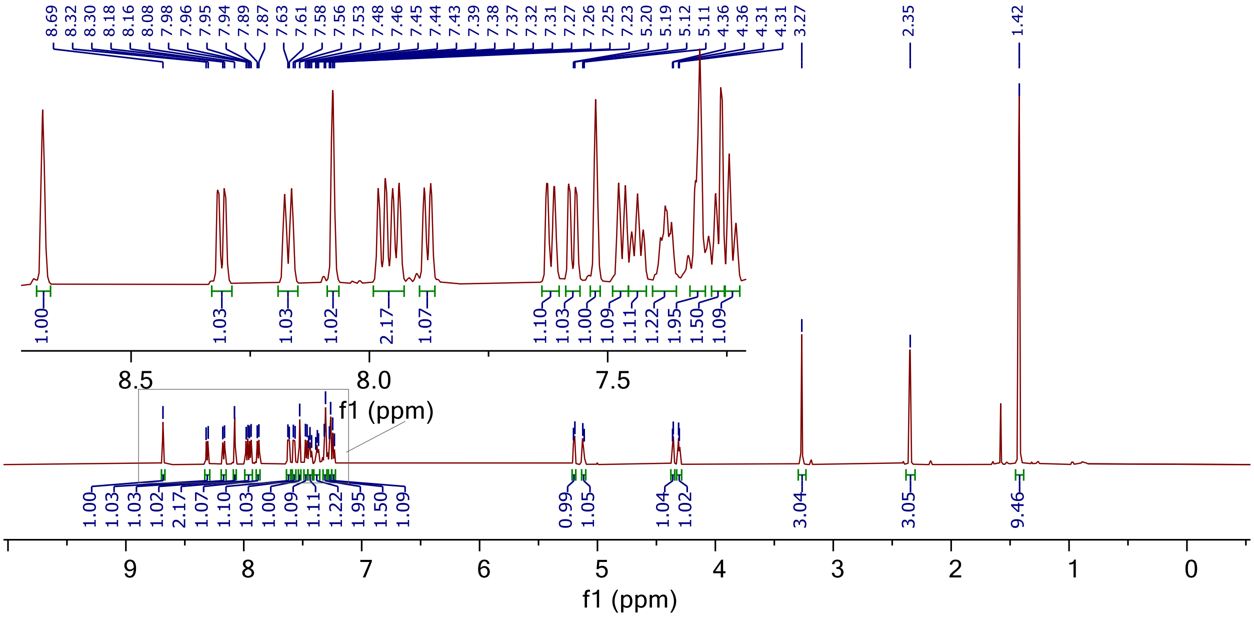

^{13}C NMR (151 MHz, $CDCl_3$)

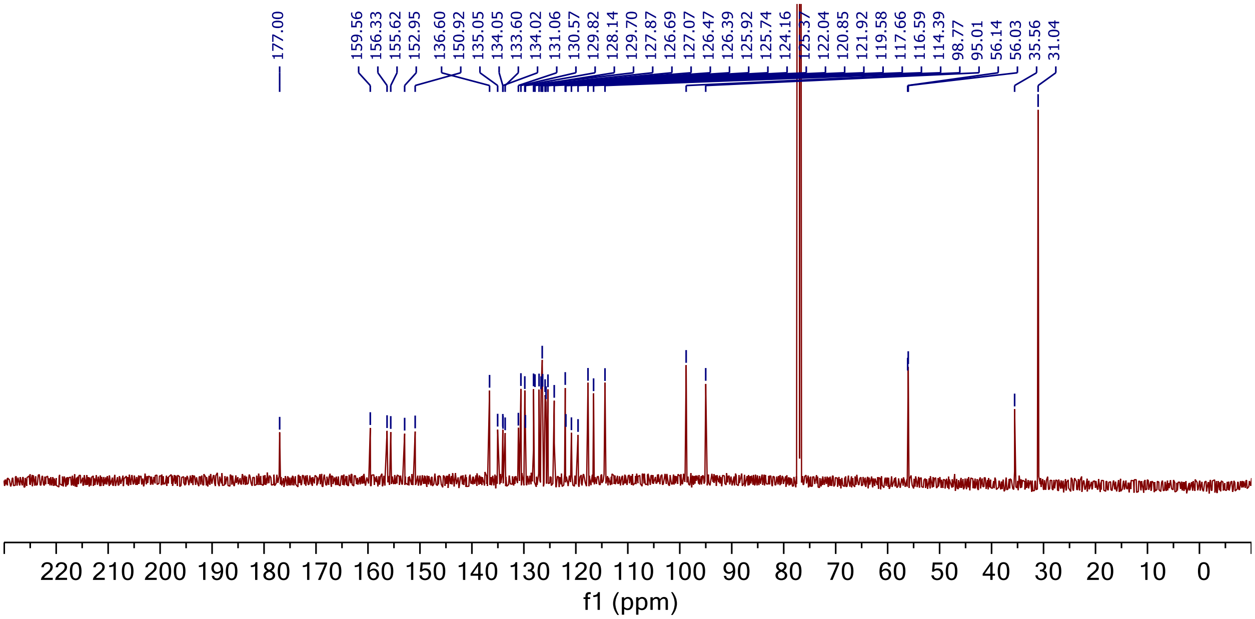

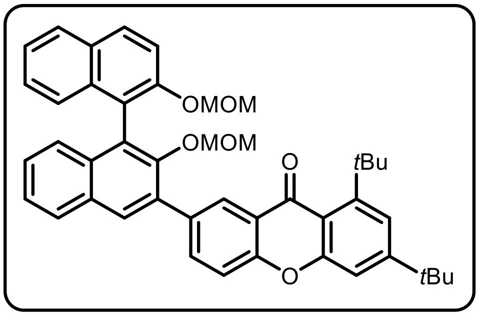

7-(2,2'-bis(methoxymethoxy)-[1,1'-binaphthalen]-3-yl)-1,3-di-tert-butyl-9H-xanthen-9-one (S14)

^{1}H NMR (600 MHz, $CDCl_3$)

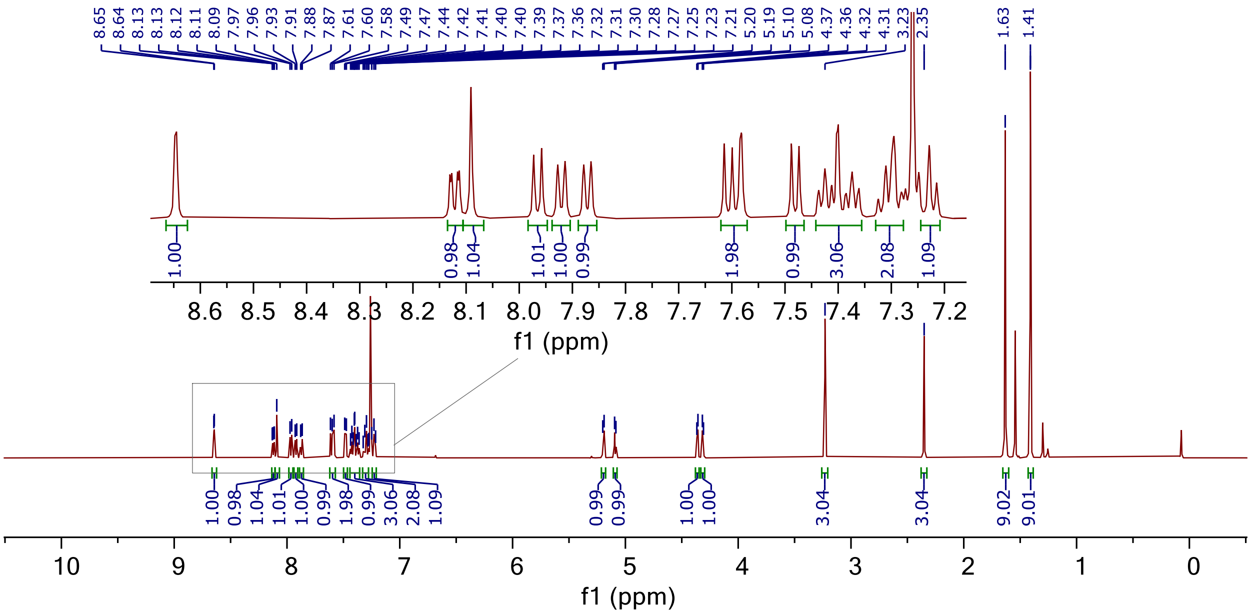

^{13}C NMR (151 MHz, $CDCl_3$)

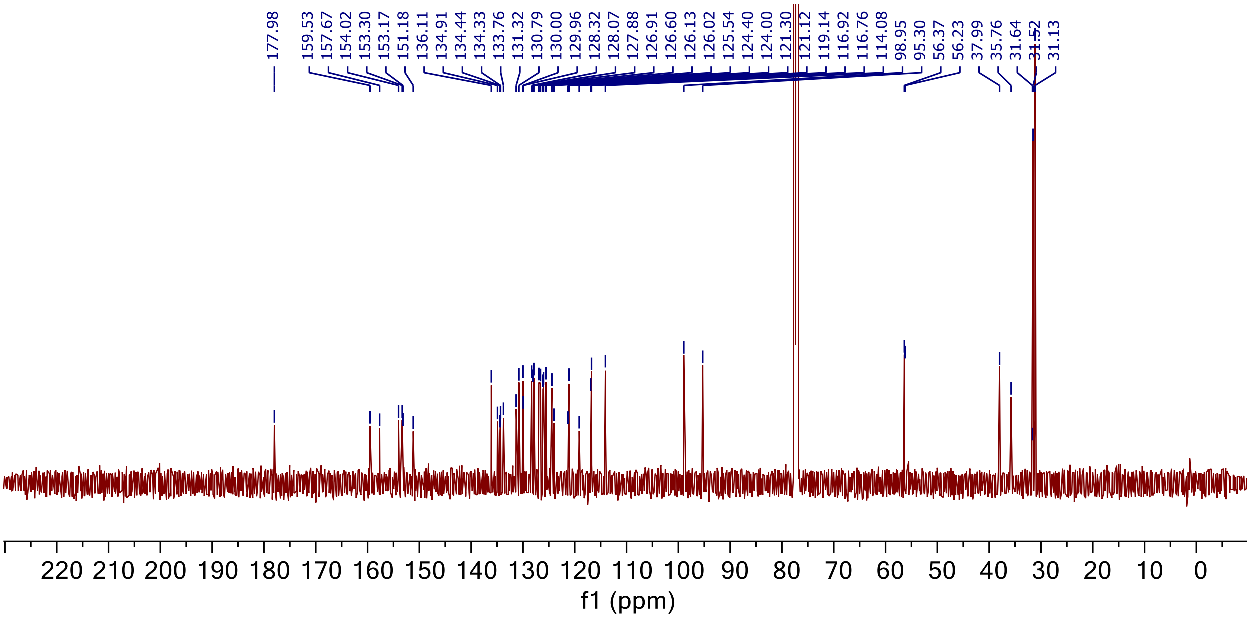

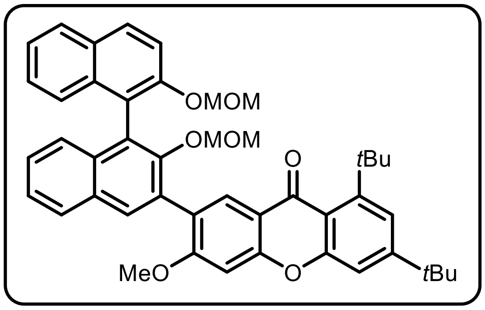

7-(2,2'-bis(methoxymethoxy)-[1,1'-binaphthalen]-3-yl)-1,3-di-tert-butyl-6-methoxy-9H-xanthen-9-one (S15)

^{1}H NMR (600 MHz, $CDCl_3$)

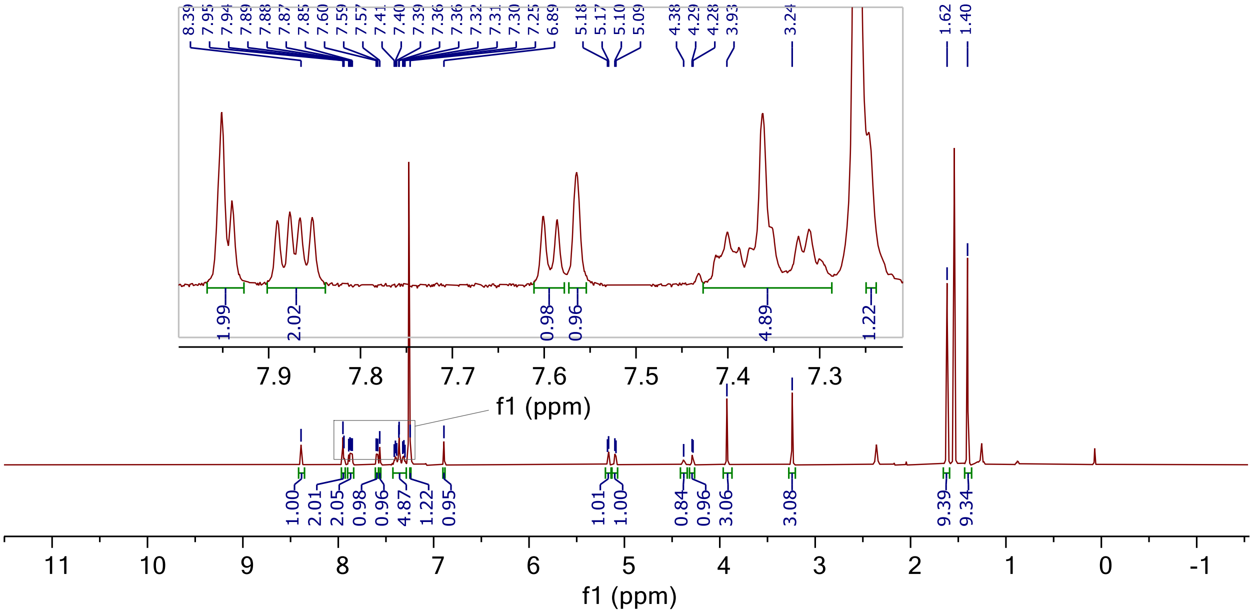

^{13}C NMR (151 MHz, $CDCl_3$)

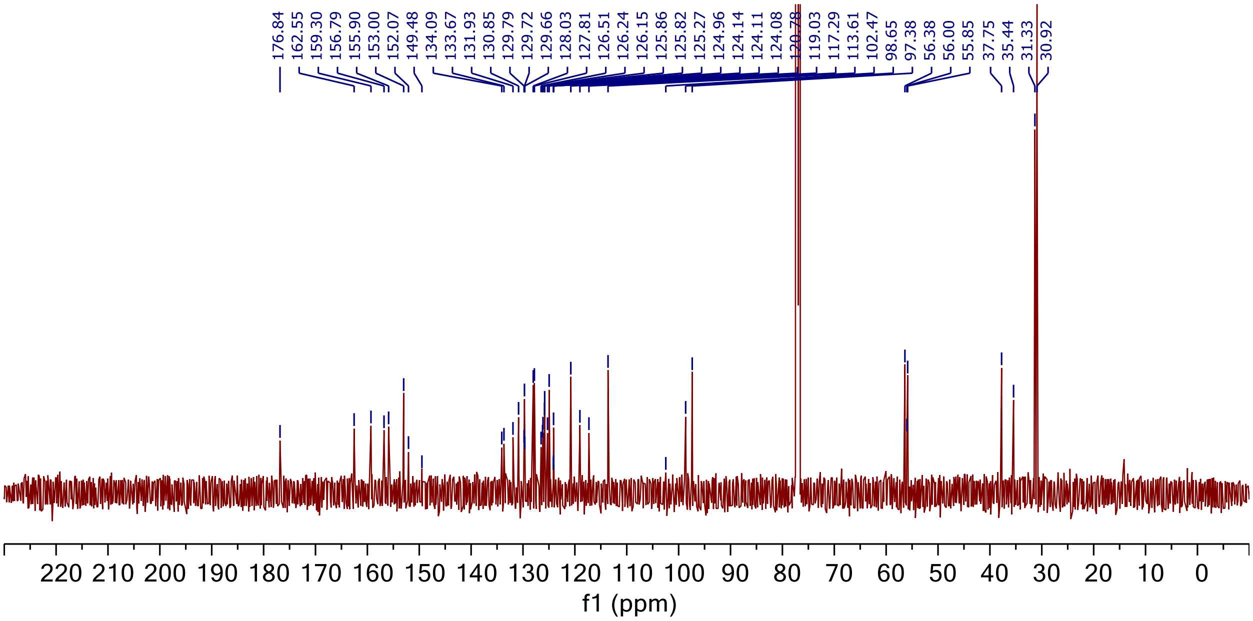

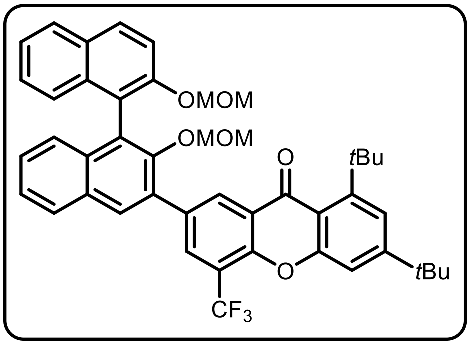

7-(2,2'-bis(methoxymethoxy)-[1,1'-binaphthalen]-3-yl)-1,3-di-tert-butyl-5-(trifluoromethyl)-9H-xanthen-9-one (S16)

^{1}H NMR (600 MHz, $CDCl_3$)

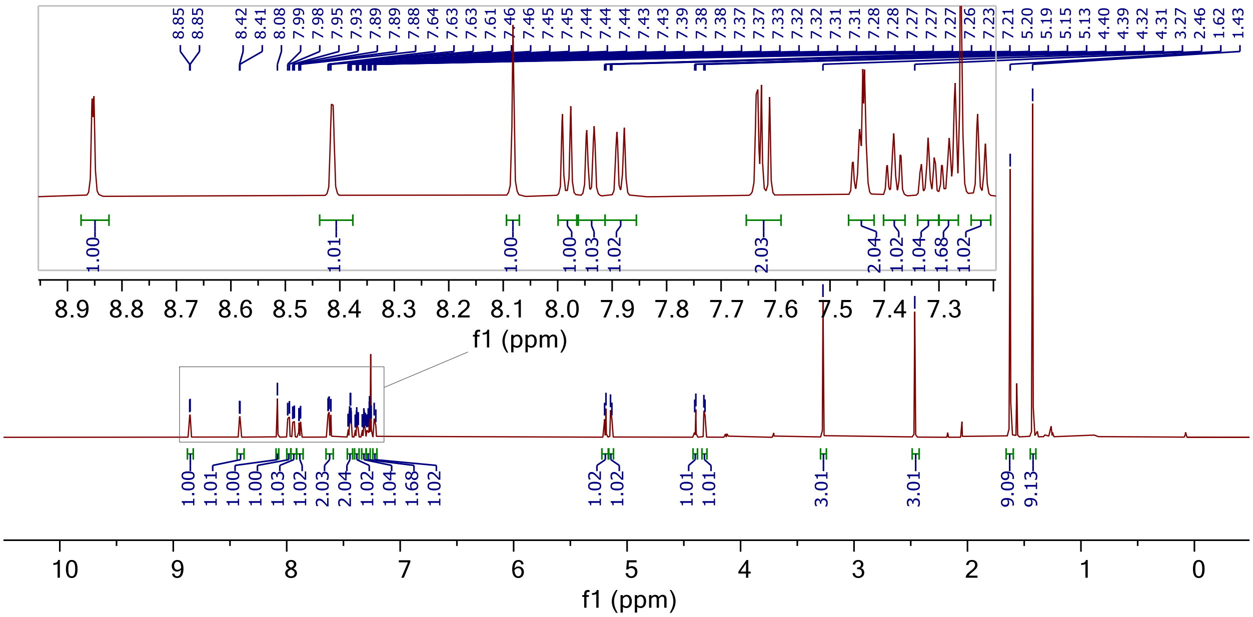

^{13}C NMR (151 MHz, $CDCl_3$)

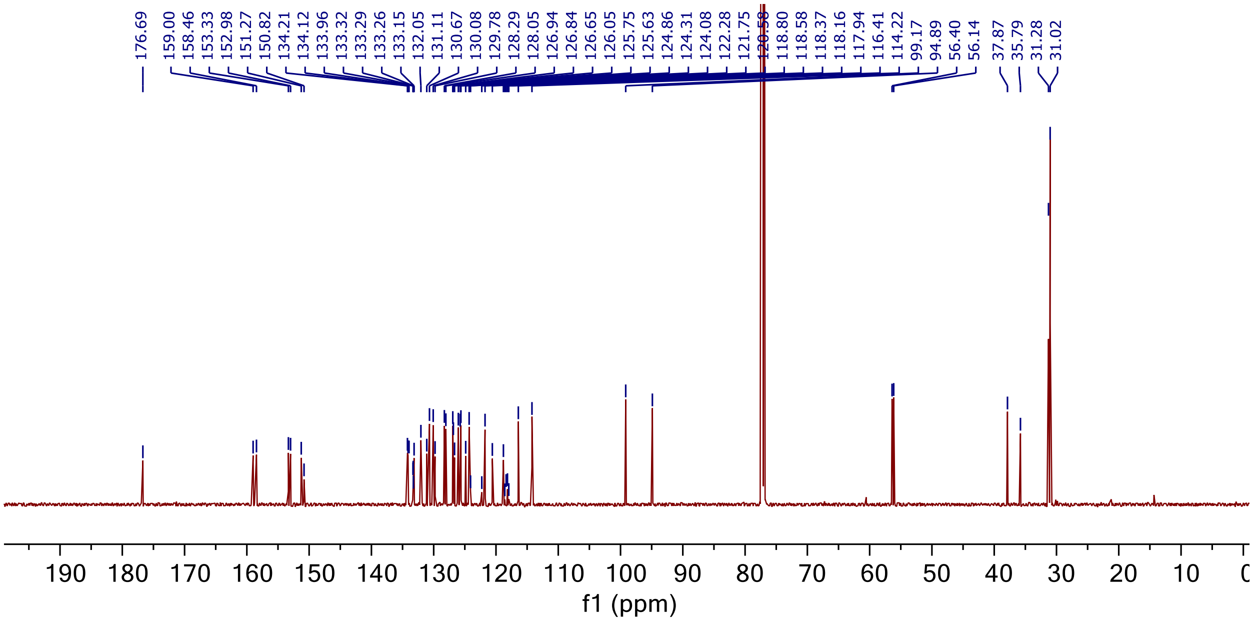

^{19}F NMR (376 MHz, $CDCl_3$)

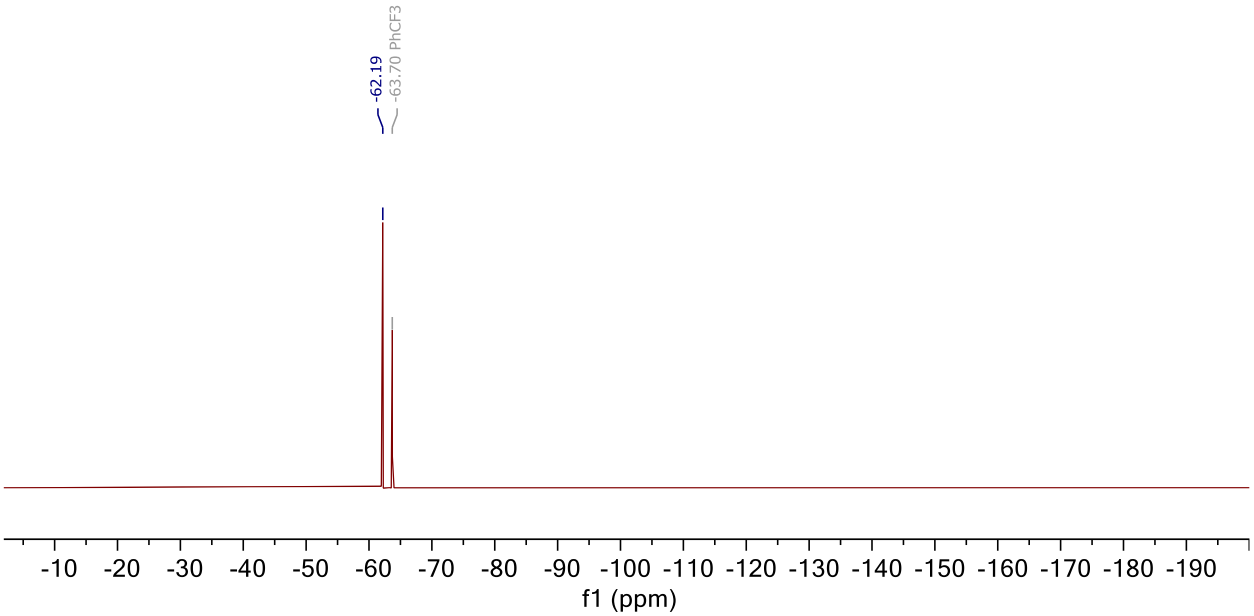

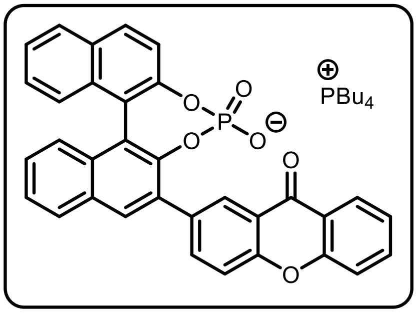

Tetrabutylphosphonium2-(9-oxo-9H-xanthen-2-yl)dinaphtho[2,1-d:1',2'-f][1,3,2]dioxaphosphepin-4-olate 4-oxide (SI-PC1)

^{1}H NMR (600 MHz, $CDCl_3$)

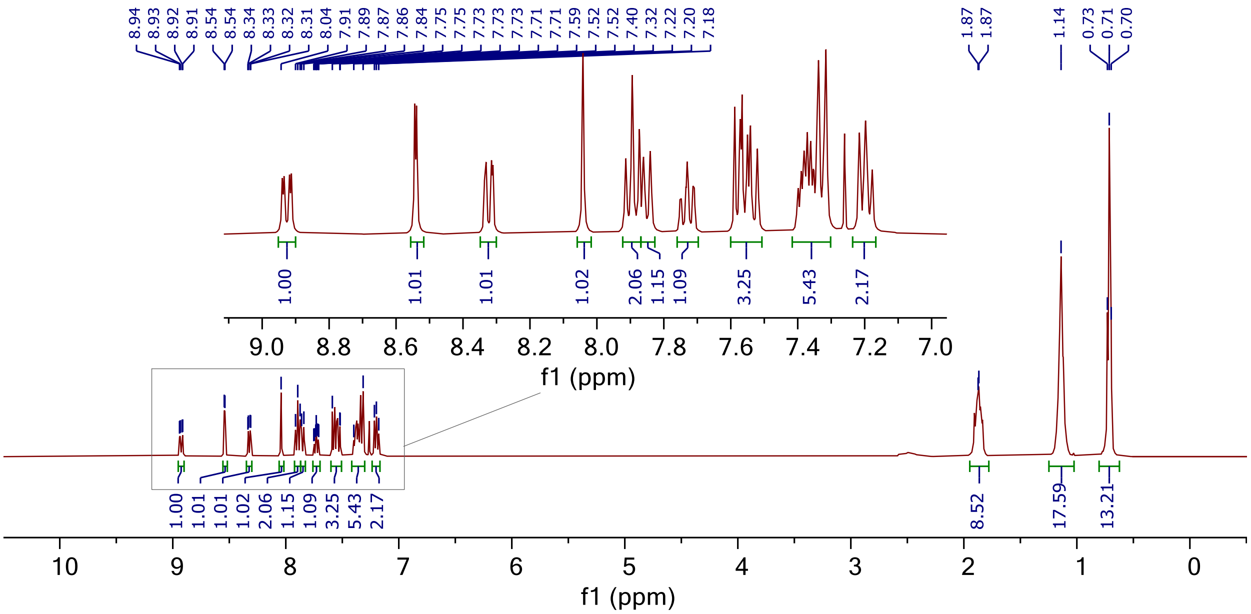

^{13}C NMR (151 MHz, $CDCl_3$)

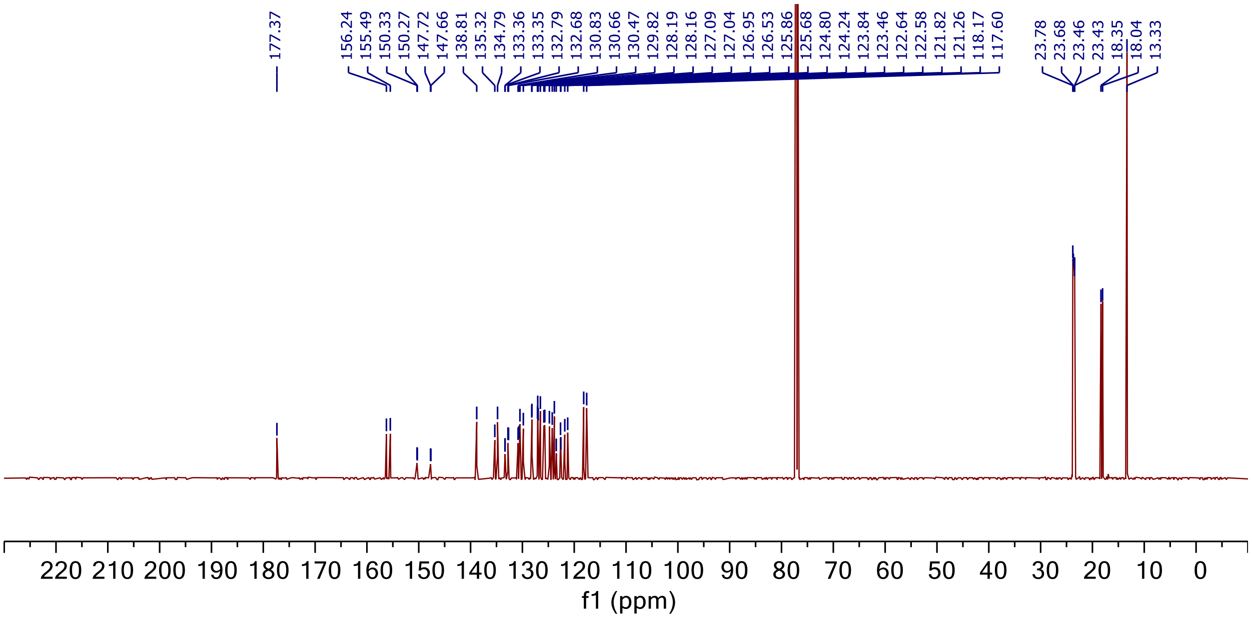

^{31}P NMR (162 MHz, $CDCl_3$)

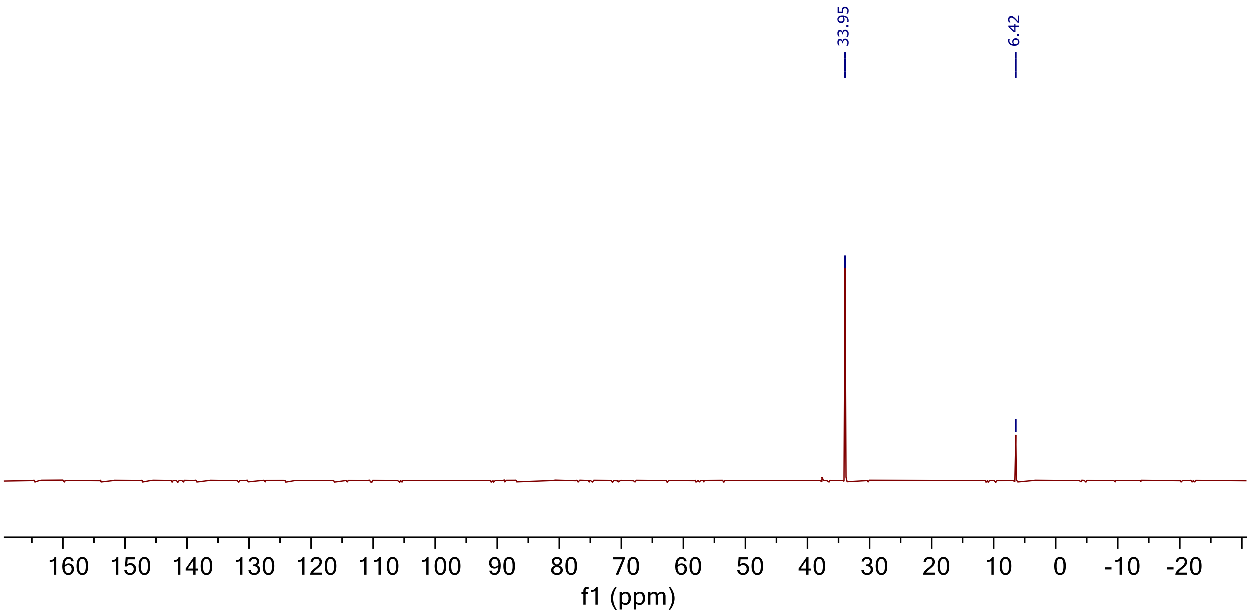

Tetrabutylphosphonium-2-(6-(tert-butyl)-9-oxo-9H-xanthen-2-yl)dinaphtho[2,1-d:1',2'-f][1,3,2]dioxaphosphepin-4-olate 4-oxide (SI-PC2)

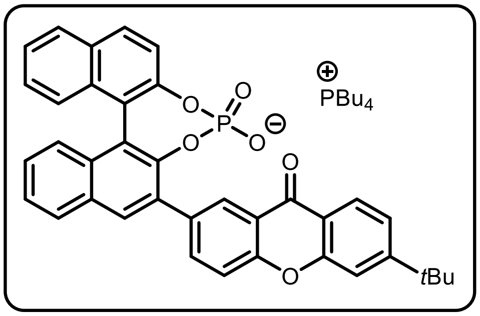

^{1}H NMR (600 MHz, $CDCl_3$)

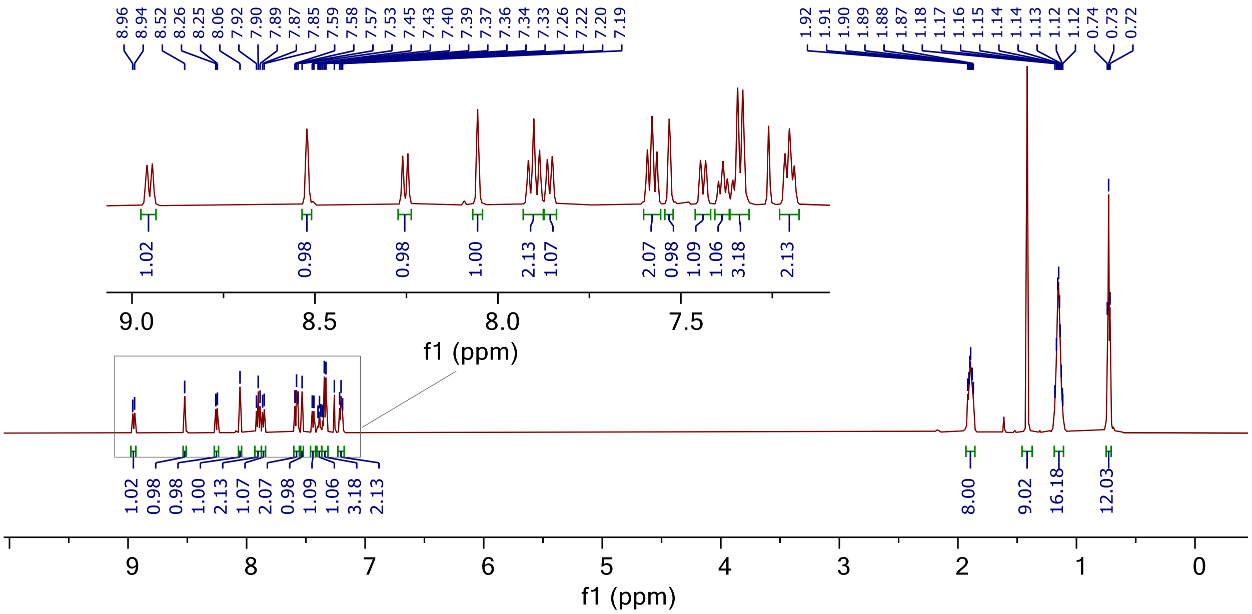

^{13}C NMR (151 MHz, $CDCl_3$)

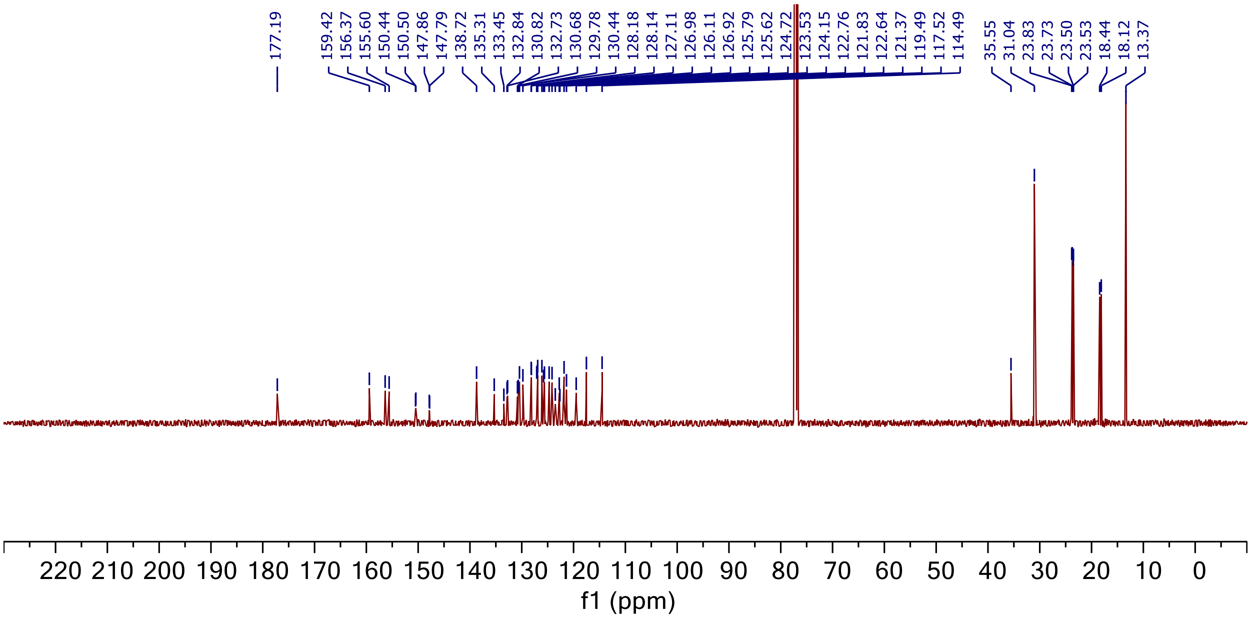

^{31}P NMR (162 MHz, $CDCl_3$)

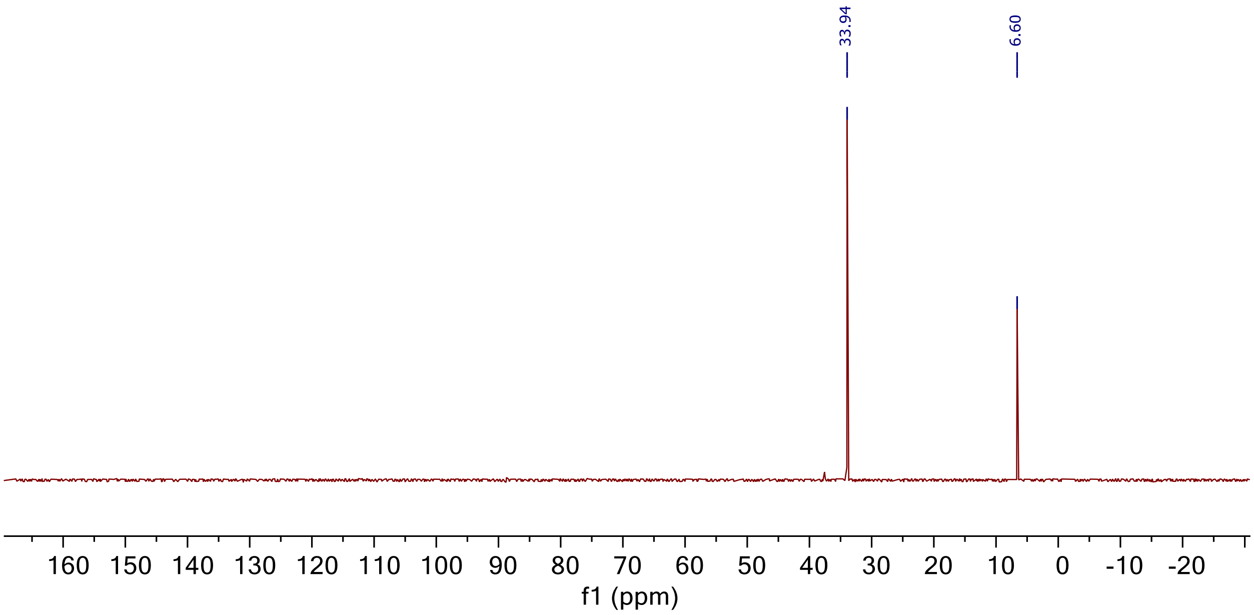

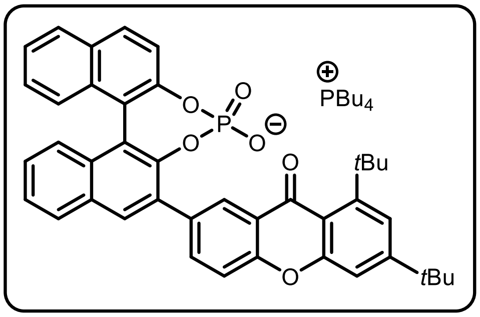

Tetrabutylphosphonium2-(6,8-di-tert-butyl-9-oxo-9H-xanthen-2-yl)dinaphtho[2,1-d:1',2'-f][1,3,2]dioxaphosphepin-4-olate 4-oxide (PC1)

^{1}H NMR (600 MHz, $CDCl_3$)

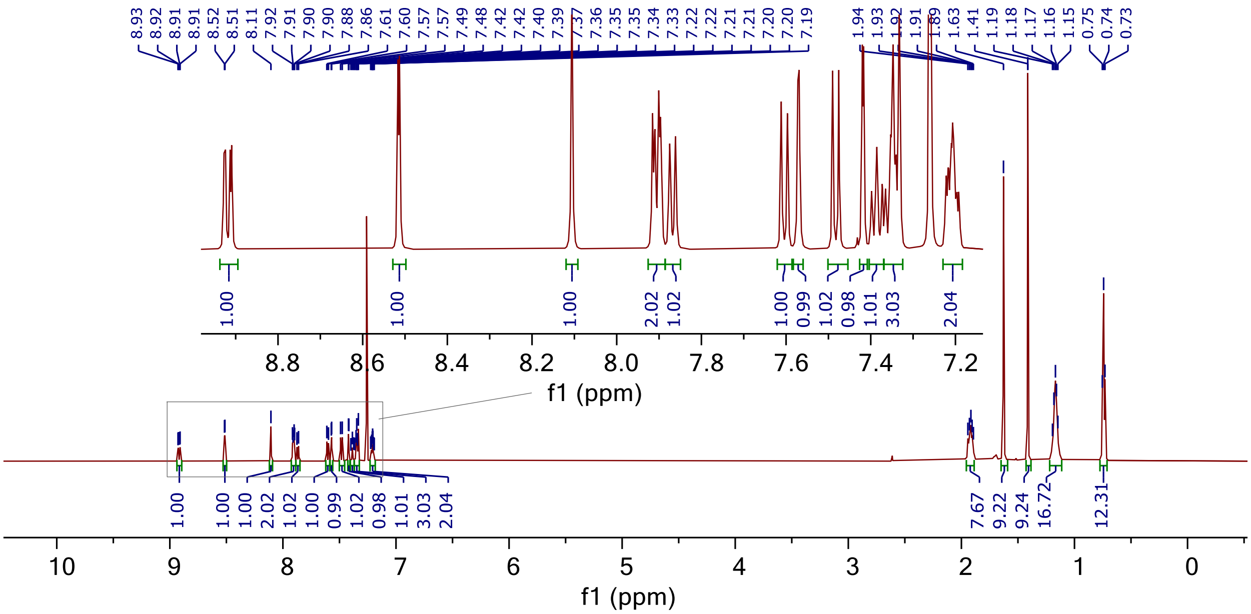

^{13}C NMR (151 MHz, $CDCl_3$)

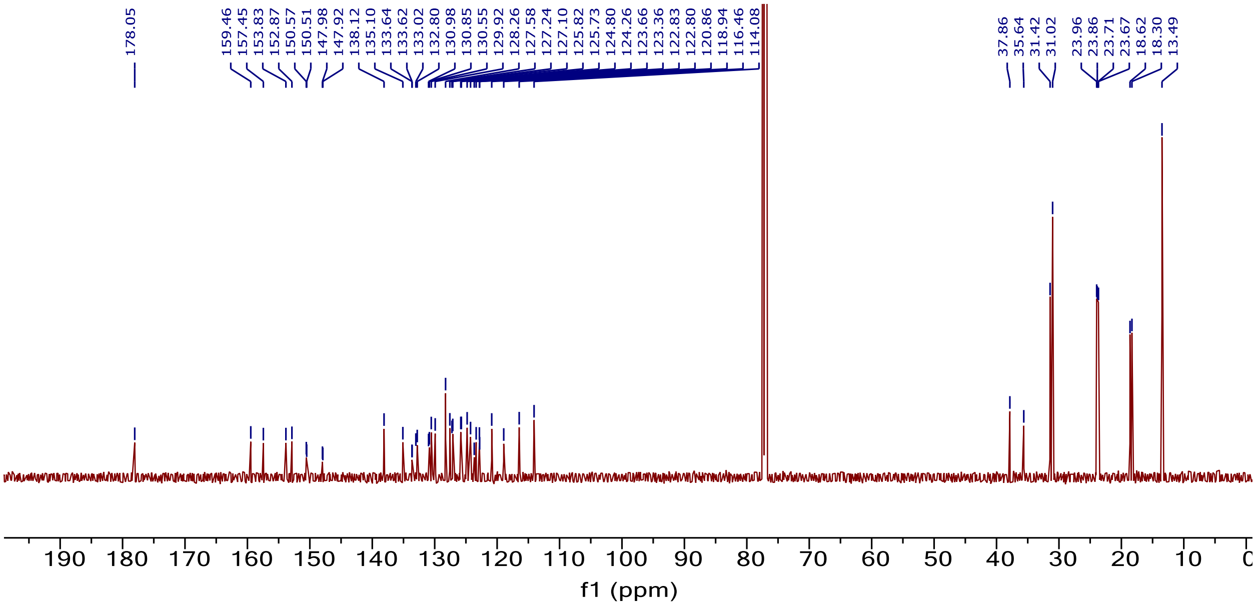

^{31}P NMR (162 MHz, $CDCl_3$)

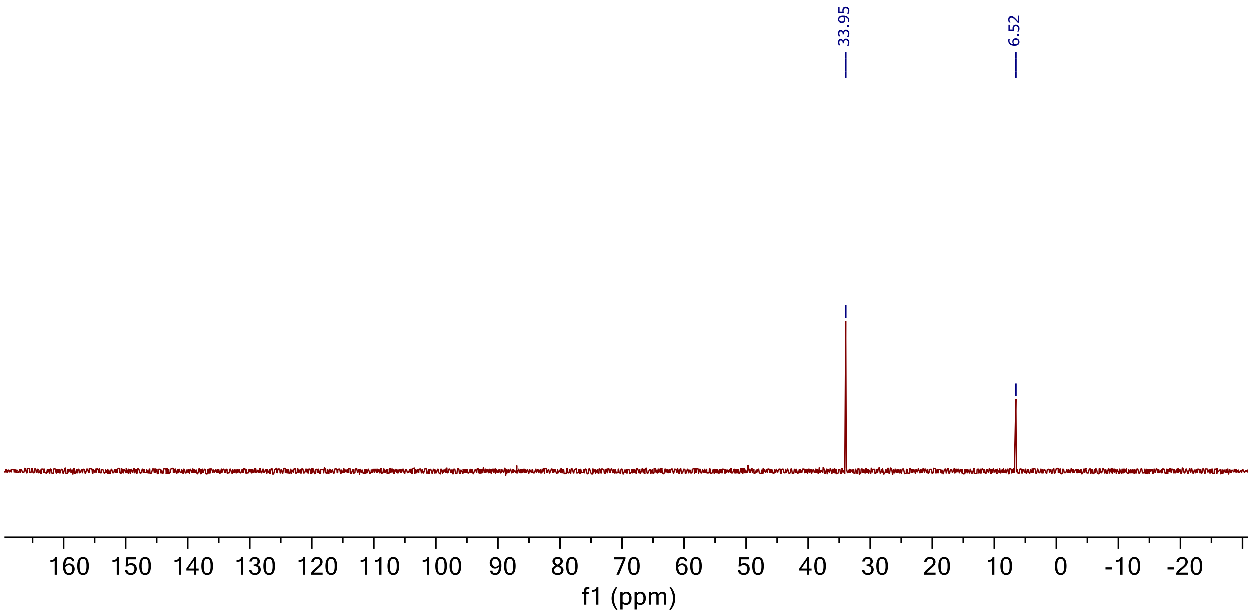

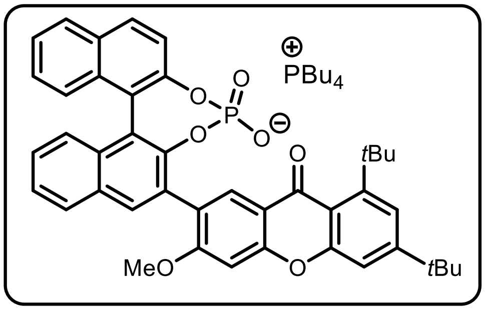

tetrabutylphosphonium 2-(6,8-di-tert-butyl-3-methoxy-9-oxo-9H-xanthen-2-yl)dinaphtho[2,1-d:1',2'-f][1,3,2]dioxaphosphepin-4-olate 4-oxide (PC2)

^{1}H NMR (600 MHz, $CDCl_3$):

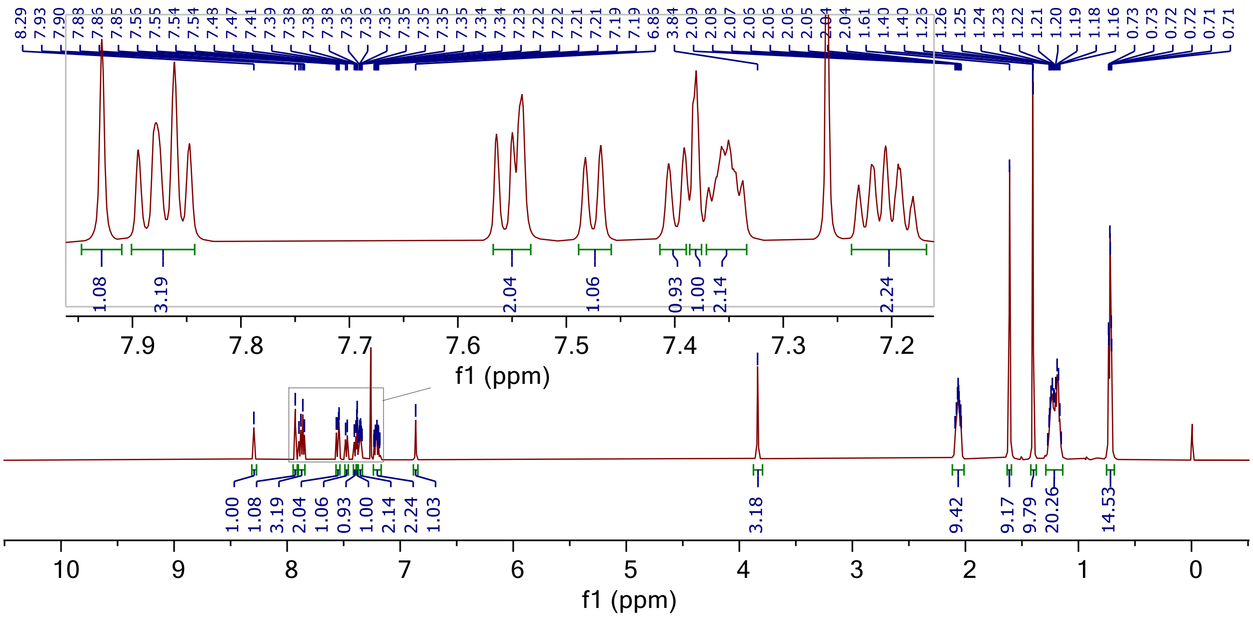

^{13}C NMR (151 MHz, $CDCl_3$)

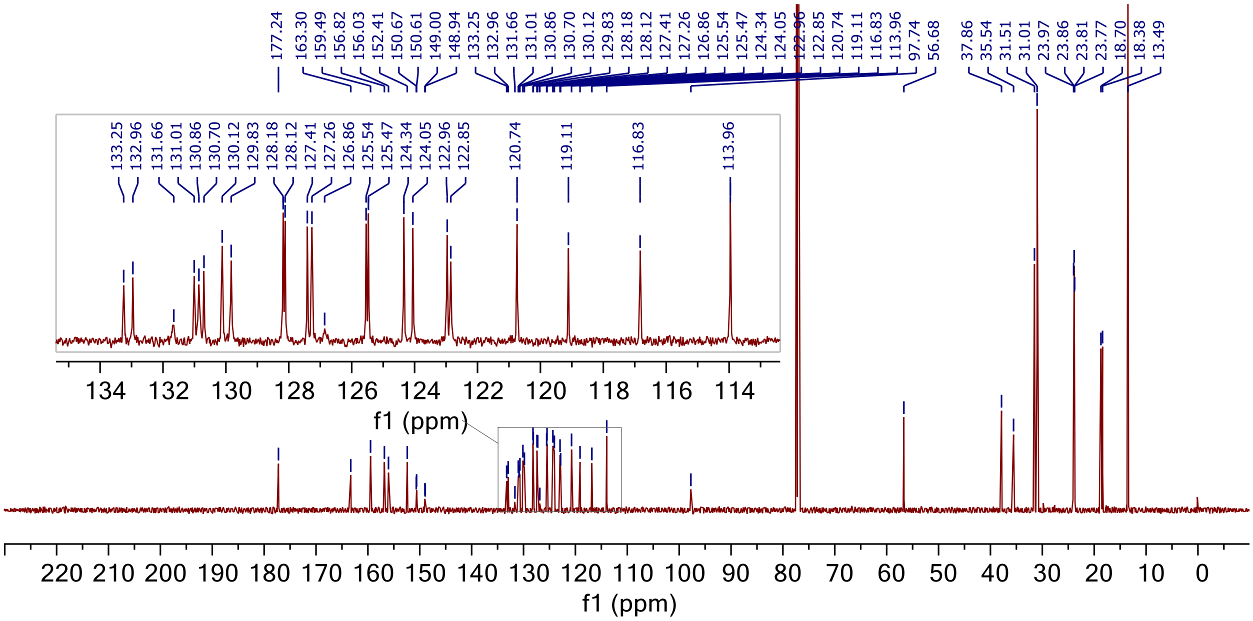

^{31}P NMR (162 MHz, $CDCl_3$)

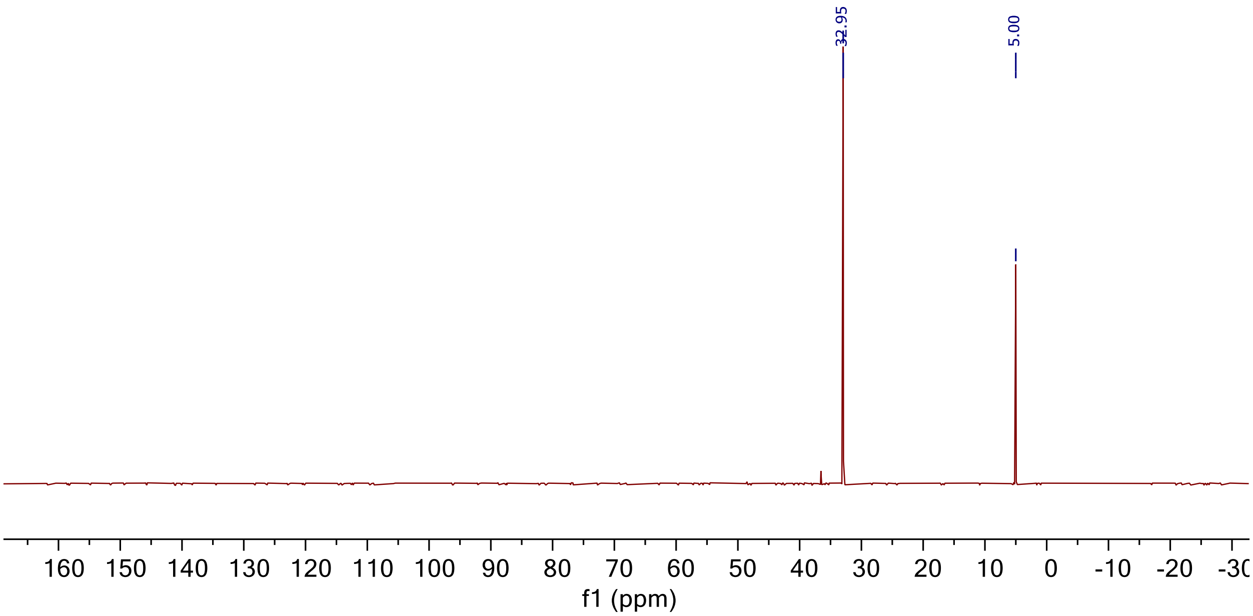

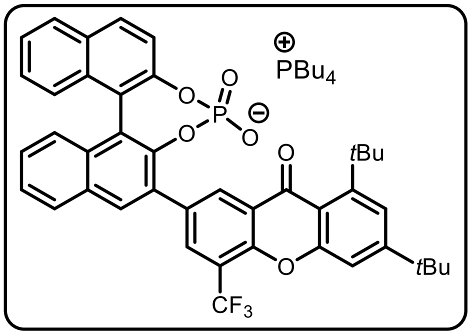

tetrabutylphosphonium 2-(6,8-di-tert-butyl-9-oxo-4-(trifluoromethyl)-9H-xanthen-2-yl)dinaphtho[2,1-d:1',2'-f][1,3,2]dioxaphosphepin-4-olate 4-oxide (PC3)

^{1}H NMR (600 MHz, $CDCl_3$)

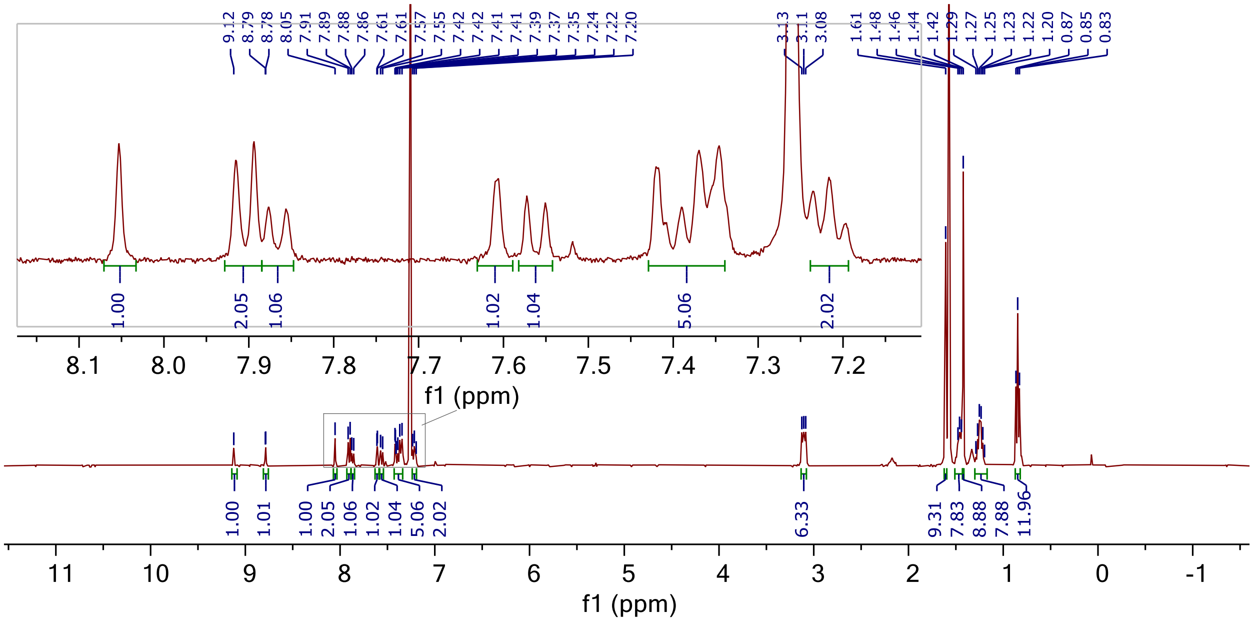

^{13}C NMR (151 MHz, $CDCl_3$)

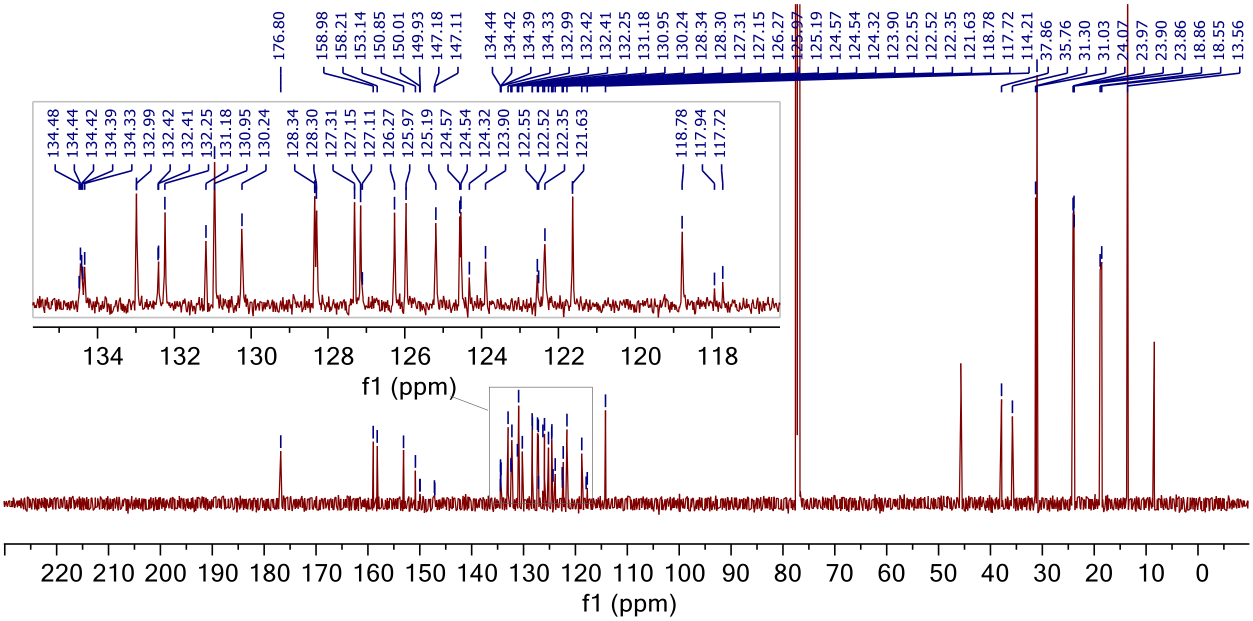

^{31}P NMR (162 MHz, $CDCl_3$)

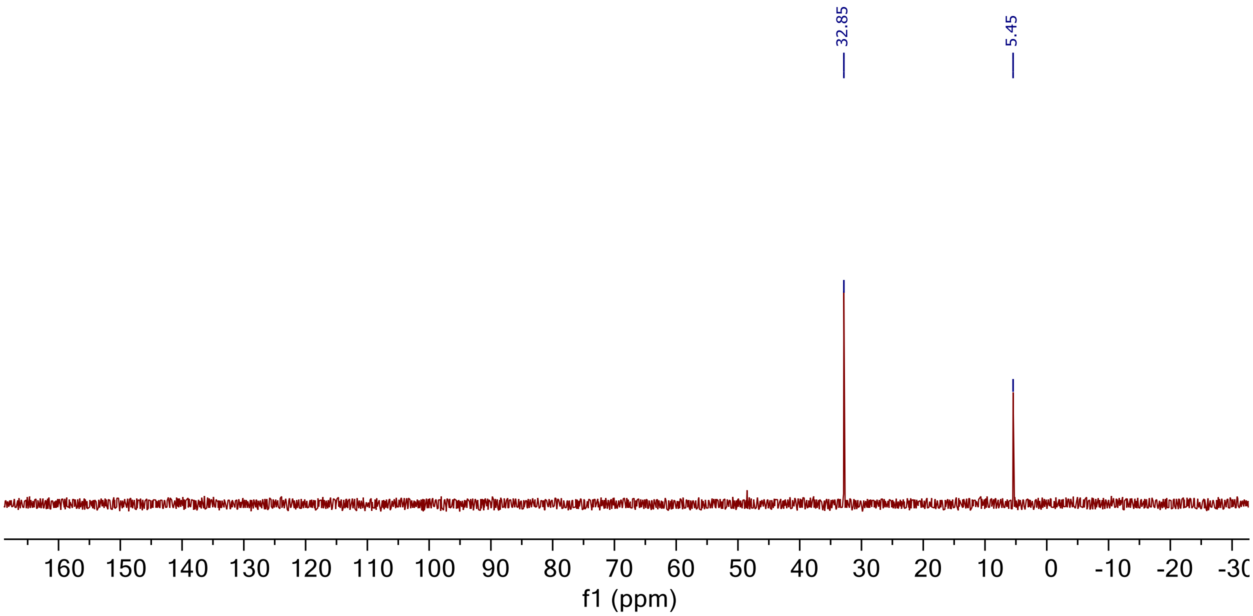

^{19}F NMR (376 MHz, $CDCl_3$)

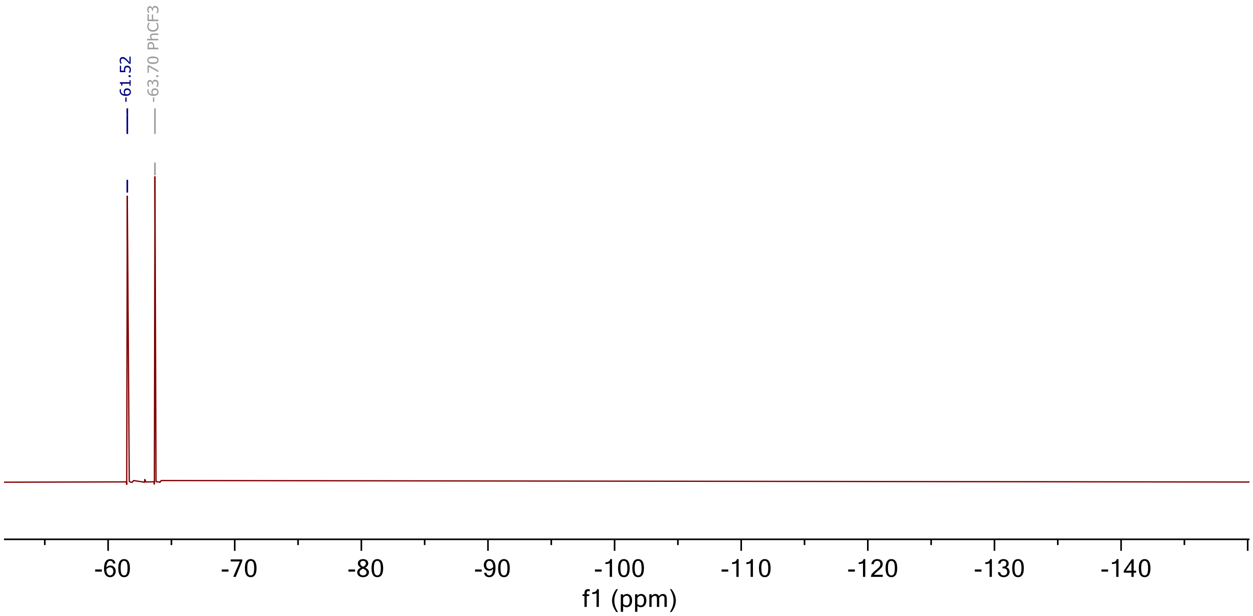

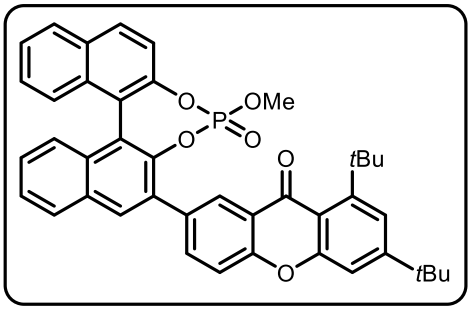

1,3-di-tert-butyl-7-(4-methoxy-4-oxidodinaphtho[2,1-d:1',2'-f][1,3,2]dioxaphosphepin-2-yl)-9H-xanthen-9-one (Me-PC1)

^{1}H NMR (600 MHz, $CDCl_3$)

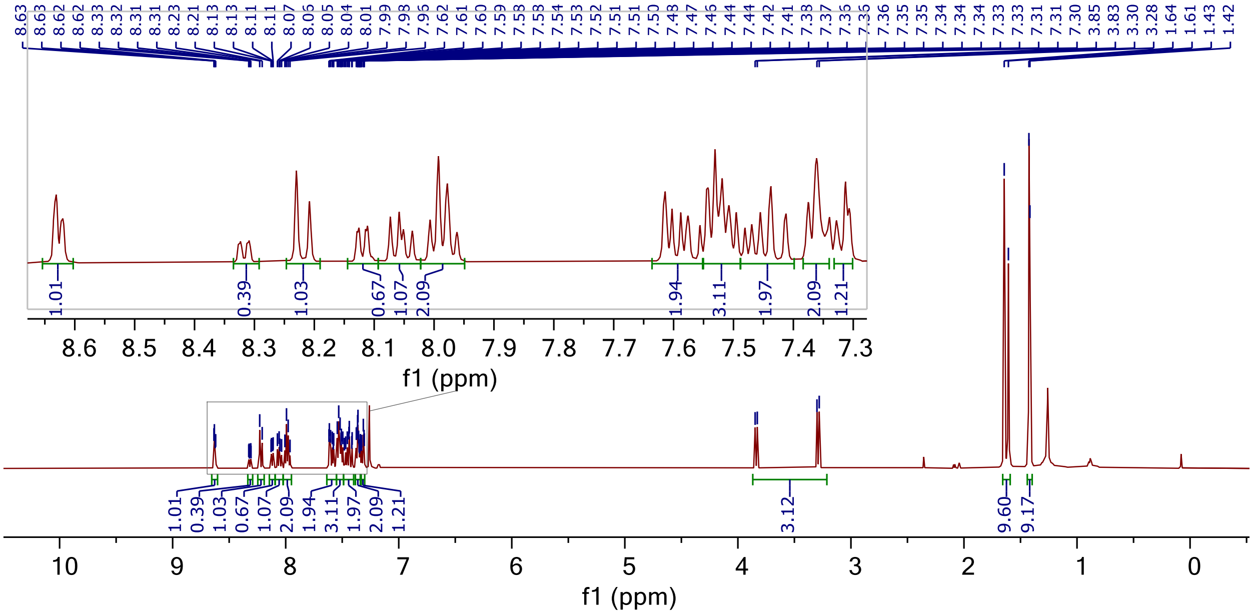

^{13}C NMR (151 MHz, $CDCl_3$)

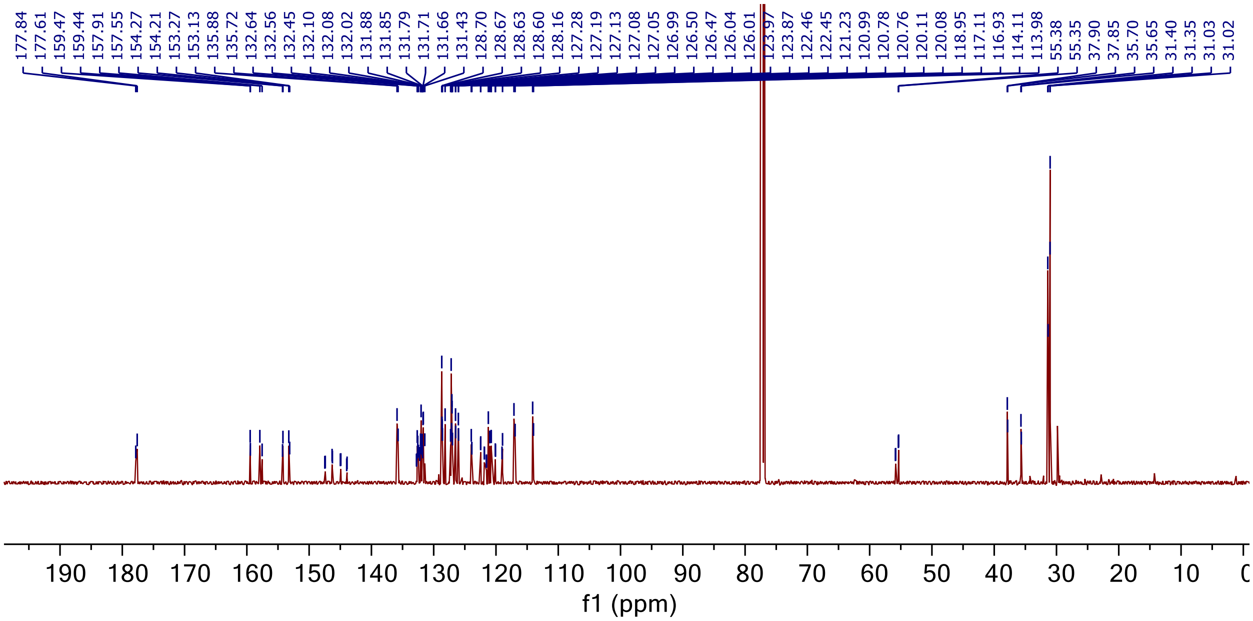

^{31}P NMR (162 MHz, $CDCl_3$)

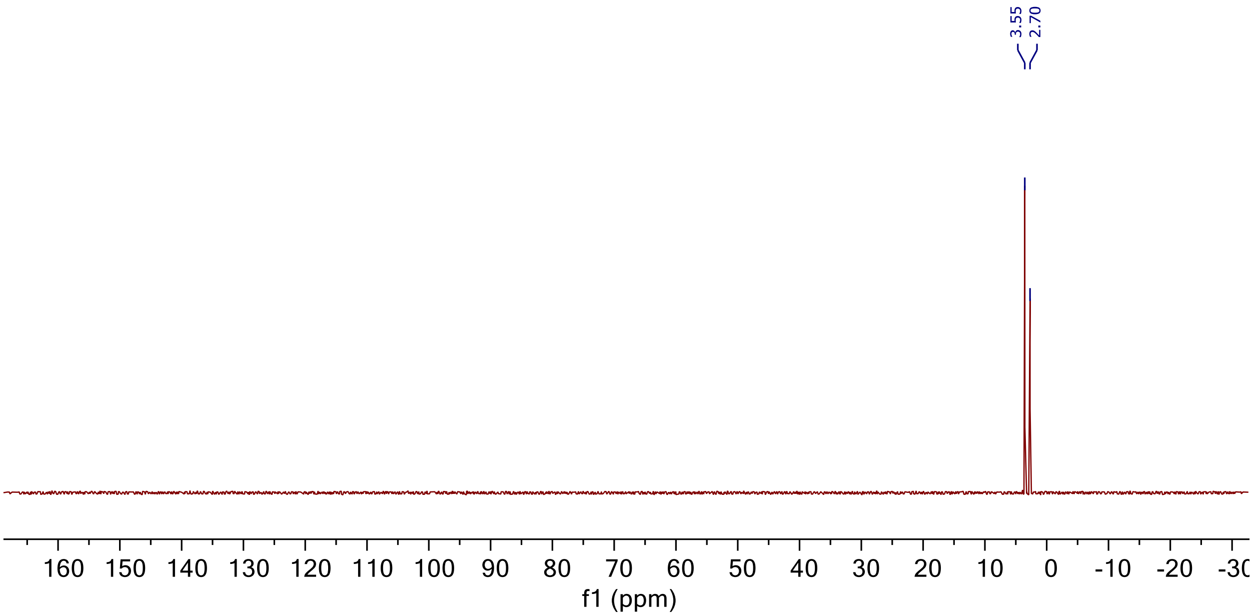

iii) Physical Characterization of Catalysts

UV-Vis data was collected in $PhCF_3$ using a Hitachi U-3000 spectrophotometer utilizing the UV solutions software (program #: 1344331-15). All samples were measured in absorbance mode with a wavelength range from 250 – 700 nm and a slit width of 2.0 nm.

Samples were prepared from serial dilutions of 1 mg of each respective catalyst in 10 mL $PhCF_3$ until reaching between 0.25 and 0.60 Abs.

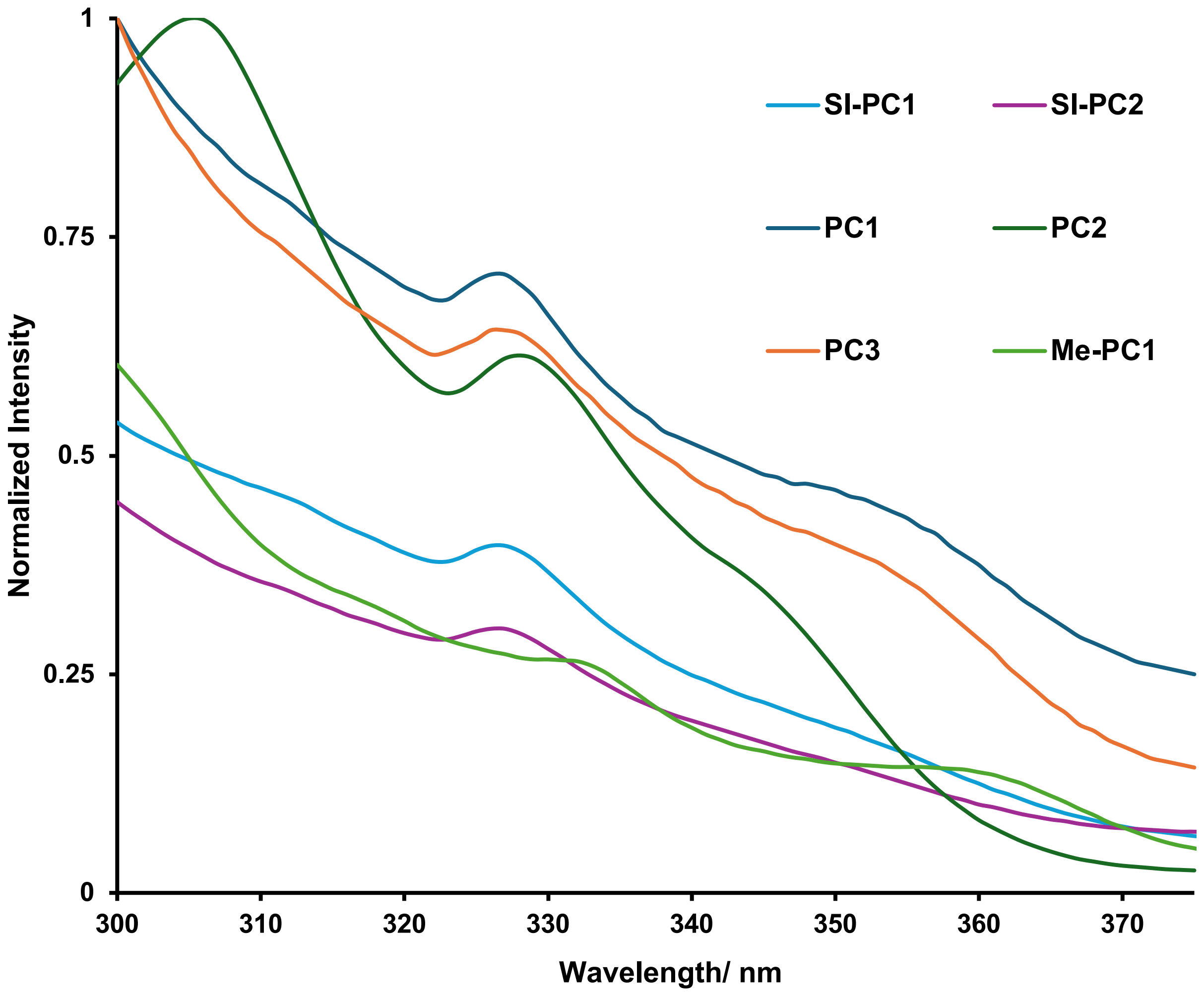

Figure SI-2 - UV-Vis spectra of photocatalysts from 300 – 400nm

D. Synthesis and Characterization of Intramolecular Substrates

i) General Procedures

General procedure D1: Synthesis of diol substrates via S_N2 and reduction

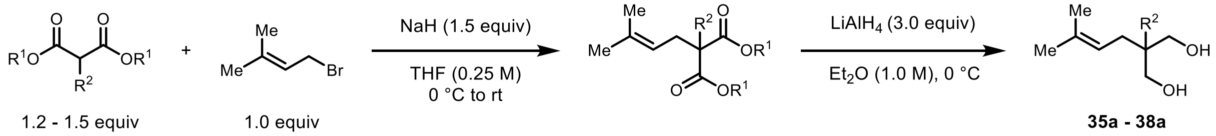

A flame-dried 25-mL round-bottomed flask was charged with NaH (3.5 mmol, 1.5 equiv), sealed with a rubber septum, and then evacuated and backfilled with nitrogen three times, after which anhydrous THF (4.7 mL) was added via syringe. The flask was then placed in an ice-water bath to cool to 0 °C. Following a 5-minute temperature equilibration period, the respective malonate (2.8 - 3.5 mmol, 1.2 - 1.5 equiv) was added to the reaction flask in anhydrous THF (2.3 mL) in a dropwise fashion. After stirring the reaction at 0 °C for 30 minutes, a solution of prenyl bromide (2.3 mmol, 1.0 equiv) in anhydrous THF (2.3 mL) was added dropwise. The reaction was then stirred at room temperature until complete consumption of the malonate was observed by TLC. At this point, the vial was placed in an ice-water bath and cooled to 0°C. After stirring for 5 minutes, the reaction was quenched with 3 M HCl (1.0 mL), diluted with Et_2O (5.0 mL) and washed with brine (3 × 10 mL). The organic phase was collected, dried over anhydrous Na_2SO_4, and concentrated *in vacuo*. The crude mixture then carried through to the next step.

A flame-dried 25-mL round-bottomed flask was charged with $LiAlH_4$ (4.5 mmol, 3.0 equiv), sealed with a rubber septum, and then evacuated and backfilled with nitrogen three times, after which anhydrous Et_2O (0.73 mL) was added. The flask was then placed in an ice-water bath and cooled to 0°C. Following a 5-minute equilibration period, a solution of the respective ester derivative (1.5 mmol, 1.0 equiv) in anhydrous Et_2O (0.73 mL) was added in a dropwise fashion. The reaction was then warmed to room temperature and stirred until completion (assessed by TLC).

The round-bottomed flask was cooled to 0°C and quenched with H_2O (0.30 mL), 2 M NaOH (0.30 mL), and H_2O (0.90 mL) were added sequentially in a dropwise fashion waiting for gas evolution to cease. After stirring at 0°C for 15 minutes, anhydrous $MgSO_4$ was added to the reaction flask under vigorous stirring and allowed to warm to room temperature. After 15 minutes the reaction was diluted with Et_2O (7 mL), filtered through a fritted funnel, and rinsed with additional Et_2O (7 mL × 3). The filtrate was then concentrated under vacuum to obtain the respective crude of **35a – 38a**, which was purified by silica column chromatography.

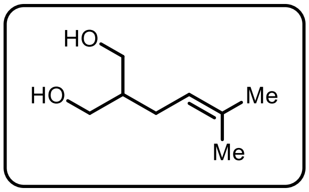

2-(3-methylbut-2-en-1-yl)propane-1,3-diol (35a). The reaction was set up using the general procedure **D1** with dimethyl malonate (1.33 g, 10.1 mmol, 1.5 equiv) for 18 hours. Following this, the crude product was purified using silica column chromatography (hexanes/acetone 100/0 to hexanes/acetone 50/50) to yield the indicated product in 56% yield (500 mg) as a colorless liquid. The resulting product has been previously reported and matches the obtained spectroscopic data tabulated below.[2]

¹H NMR (400 MHz, $CDCl_3$): δ 5.13 (tp, *J* = 7.4, 1.2 Hz, 1H), 3.79 (d, *J* = 10.3 Hz, 2H), 3.66 (dd, *J* = 10.8, 7.3 Hz, 2H), 2.20 (s, 2H), 1.97 (t, *J* = 7.3 Hz, 2H), 1.85 – 1.79 (m, 1H), 1.70 (s, 3H), 1.61 (s, 3H).

¹³C NMR (151 MHz, $CDCl_3$): δ 133.5, 121.9, 66.4, 66.4, 42.9, 26.7, 25.9, 17.8.

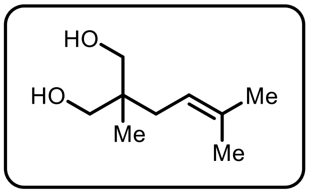

2-methyl-2-(3-methylbut-2-en-1-yl)propane-1,3-diol (36a). The reaction was set up using the general procedure **D1** with diethyl-2-methylmalonate (614 mg, 3.5 mmol, 1.5 equiv) for 18 hours. Following this, the crude product was purified using silica column chromatography (hexanes/EtOAc 100/0 to hexanes/EtOAc 60/40) to yield the indicated product in 62% yield (254 mg) as a white solid. The resulting product has been previously reported and matches the obtained spectroscopic data tabulated below.[3]

¹H NMR (600 MHz, $CDCl_3$): δ 5.21 – 5.16 (m, 1H), 3.56 (dd, *J* = 10.7, 3.6 Hz, 2H), 3.52 (dd, *J* = 10.7, 3.4 Hz, 2H), 2.33 (s, 2H), 2.03 (d, *J* = 7.8 Hz, 2H), 1.72 (s, 3H), 1.63 (s, 3H), 0.83 (s, 3H).

¹³C NMR (151 MHz, $CDCl_3$): δ 134.4, 118.6, 70.4, 40.2, 32.6, 26.2, 18.7, 18.0.

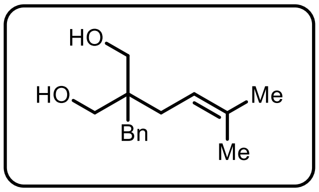

2-benzyl-2-(3-methylbut-2-en-1-yl)propane-1,3-diol (37a). The reaction was set up using the general procedure **D1** with diethyl-2-benzylmalonate (2.24 g, 10.1 mmol, 1.5 equiv) for 18 hours. Following this, the crude product was purified using silica column chromatography (hexanes/EtOAc 100/0 to hexanes/EtOAc 90/10) to yield the indicated product in 61% yield (929 mg) as a white solid. The resulting product has been previously reported and matches the obtained spectroscopic data tabulated below.[3]

¹H NMR (600 MHz, $CDCl_3$): δ 7.28 – 7.26 (m, 2H), 7.22 – 7.19 (m, 3H), 5.27 (att, *J* = 7.4, 1.3 Hz, 1H), 3.60 (dd, *J* = 10.9, 4.7 Hz, 2H), 3.54 (dd, *J* = 10.7, 5.4 Hz, 2H) 2.70 (s, 2H), 2.23 (m, 2H), 1.94 (d, *J* = 7.5 Hz, 2H), 1.75 (s, 3H), 1.60 (s, 3H).

¹³C NMR (151 MHz, $CDCl_3$): δ 137.9, 134.7, 130.6, 128.2, 126.3, 119.3, 68.2, 43.6, 37.8, 30.3, 26.3, 18.1.

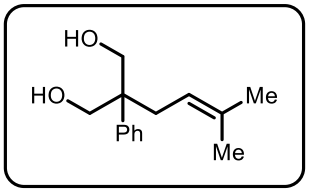

2-(3-methylbut-2-en-1-yl)-2-phenylpropane-1,3-diol (38a). The reaction was set up using the general procedure **D1** with diethyl-2-phenylmalonate (1.00 g, 6.7 mmol, 1.2 equiv) for 18 hours. Following this, the crude product was purified using silica column chromatography (hexanes/EtOAc 100/0 to hexanes/EtOAc 90/10) to yield the indicated product in 46% yield (704 mg) as a colorless liquid. The resulting product has been previously reported and matches the obtained spectroscopic data tabulated below.[4]

^{1}**_H NMR_** (600 MHz, $CDCl_3$): δ 7.37 – 7.34 (m, 4H), 7.26 – 7.23 (m, 1H), 4.86 (att, *J* = 7.4, 1.4 Hz, 1H), 4.03 (dd, *J* = 11.0, 4.3 Hz, 2H), 3.89 (dd, *J* = 11.1, 4.6 Hz, 2H), 2.46 (s, 2H), 2.36 (d, *J* = 7.6 Hz, 2H), 1.61 (s, 3H), 1.55 (s, 3H).

^{13}**_C NMR_** (151 MHz, $CDCl_3$): δ 141.6, 134.6, 128.7, 127.3, 126.6, 119.0, 68.5, 47.9, 33.1, 26.0, 18.0.

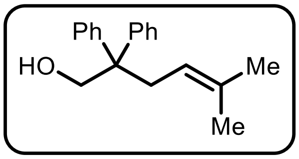

5-methyl-2,2-diphenylhex-4-en-1-ol (39a). The substrate was synthesized according to a known procedure.[5] The resulting product has been previously reported and matches the obtained spectroscopic data tabulated below.[5]

^{1}**_H NMR_** (600 MHz, $CDCl_3$): δ 7.30 (at, *J* = 7.6 Hz, 4H), 7.23 – 7.19 (m, 6H), 4.85 (att, *J* = 7.2, 1.3 Hz, 1H), 4.13 (s, 2H), 2.89 (d, *J* = 7.6 Hz, 2H), 1.60 (s, 3H), 1.54 (s, 3H), 1.17 (br s, 1H).

^{13}**_C NMR_** (151 MHz, $CDCl_3$): δ 145.7, 134.7, 128.4, 128.3, 126.4, 119.9, 68.4, 52.4, 35.2, 26.1, 18.0.

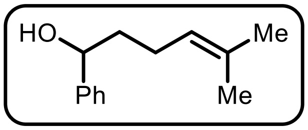

5-methyl-1-phenylhex-4-en-1-ol (40a). The substrate was synthesized according to a known procedure.[6] The resulting product has been previously reported and matches the obtained spectroscopic data tabulated below.[6]

^{1}**_H NMR_** (600 MHz, $CDCl_3$): δ 7.35 (d, J = 4.4 Hz, 4H), 7.29 – 7.26 (m, 1H), 5.15 (dt, J = 7.2, 7.1 Hz, 1H), 4.70 – 4.67 (m, 1H), 2.12 – 2.02 (m, 2H), 1.87 – 1.81 (m, 2H), 1.78 – 1.73 (m, 1H), 1.69 (s, 3H), 1.58 (s, 3H).

^{13}**_C NMR_** (151 MHz, $CDCl_3$): 144.9, 132.5, 128.6, 127.6, 126.1, 123.9, 74.4, 39.2, 25.9, 24.6, 17.9.

General Procedure D2: Synthesis of vinyl bromide linkers via Wittig reaction

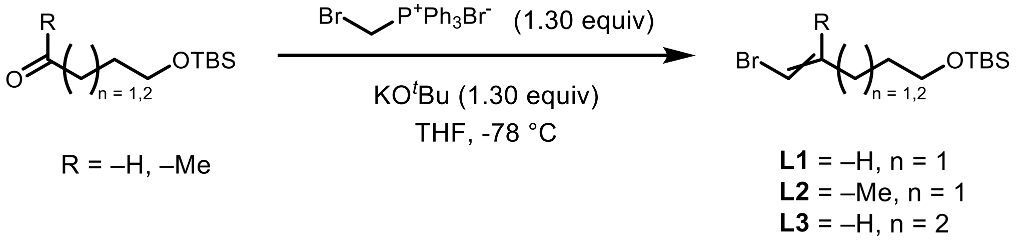

A 250-mL flame-dried round-bottomed flask was charged with a magnetic stir bar and (bromomethyl)triphenyl phosphonium bromide (32.2 mmol, 1.30 equiv), sealed with a rubber septum and evacuated and backfilled with nitrogen three times. Following this, anhydrous THF (100 mL, 0.25 M) was added via syringe, and the reaction flask was placed in a dry ice-acetone bath to cool to –78 °C. Following a five-minute equilibration period, solid KO^tBu (32.2 mmol, 1.30 equiv) was added to the flask in a single portion. The resulting mixture was stirred for 1 hour at –78 °C, after which the requisite aldehyde/ketone (24.7 mmol, 1.00 equiv) was added in a solution of anhydrous THF (10.0 mL). The mixture was then removed from the cooling bath and allowed to warm to room temperature, stirring continuously for 16 hours. After observing the full consumption of the aldehyde/ketone by TLC, the reaction mixture was diluted with H_2O (150 mL)

and extracted with Et_2O (2 × 100 mL). The combined organic layers were dried over anhydrous Na_2SO_4, filtered and concentrated *in vacuo*. The crude mixture was dissolved in a minimal amount of Et_2O to induce precipitation of the triphenylphosphine oxide byproduct. The precipitated solid was filtered and washed with hexanes, after which the filtrate was concentrated down to access the crude mixture. Residual impurities were removed by silica gel column chromatography (hexanes 100%).

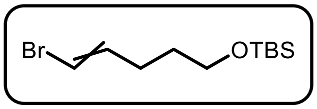

((5-bromopent-4-en-1-yl)oxy)(tert-butyl)dimethylsilane (L1). The reaction was set up using general procedure **D2** with 4-((*tert*-butyldimethylsilyl)oxy)butanal[7] (5.00 g, 24.7 mmol) for 16 hours. Following this, the crude product was purified using silica column chromatography (hexanes 100%) to yield the indicated product as an inseparable mixture of isomers in 61% yield (4.22 mg, 15.1 mmol) as a colorless liquid (*E*:*Z* = 27:33).

IR (Diamond-ATR, neat) ṽ (cm^{-1}): 2932, 2857, 1254, 1102, 833, 773, 661.

^{1}H NMR (600 MHz, $CDCl_3$): For *Z*-isomer: δ 6.15 (dt, *J* = 7.0, 1.2 Hz, 1H), 6.12 (aq, *J* = 6.9 Hz, 1H), 3.63 (t, *J* = 6.5 Hz, 2H), 2.26 (td, *J* = 7.6, 6.2 Hz, 2H), 1.68 – 1.62 (m, 2H), 0.90 (s, 9H), 0.06 (s, 6H). For *E*-isomer: δ 6.19 (add, *J* = 14.0, 6.9 Hz, 1H), 6.06 – 6.00 (m, 1H), 3.62 – 3.59 (m, 2H), 2.12 (aqd, *J* = 7.3, 1.4 Hz, 2H), 1.60 (dd, *J* = 14.1, 6.7 Hz, 2H), 0.89 (s, 9H), 0.04 (s, 6H).

^{13}C NMR (151 MHz, $CDCl_3$): For *Z*-isomer: δ 134.7, 108.0, 62.6, 31.4, 26.5, 26.1, 18.5, -5.2. For *E*-isomer: δ 137.9, 104.5, 62.1, 31.7, 29.5, 26.5, 18.5, -5.2.

HRMS (ESI): m/z: $[M+H]^+$ calc'd for $C_{11}H_{24}BrOSi^+$: 279.0779. Found: 279.0774.

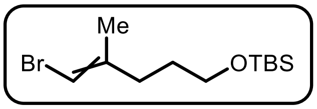

((5-bromo-4-methylpent-4-en-1-yl)oxy)(tert-butyl)dimethyl silane (L2). The reaction was set up using general procedure **D2** with 5-((*tert*-butyldimethylsilyl)oxy)pentan-2-one[8] (1.16 g, 5.4 mmol) for 16 hours. Following this, the crude product was purified using silica column chromatography (hexanes 100%) to yield the indicated product in 45% yield (702 mg, 2.4 mmol) as a colorless liquid (*E:Z*=43:57).

IR (Diamond-ATR, neat) ṽ (cm^{-1}): 2952, 2932, 2857, 1472, 1253, 1102, 833, 774, 660.

^{1}H NMR (600 MHz, $CDCl_3$): For *Z*-isomer: δ 5.90 (s, 1H), 3.59 (t, *J* = 6.2 Hz, 2H), 2.17 (t, *J* = 7.7 Hz, 2H), 1.79 (s, 3H), 1.68 – 1.61 (m, 2H), 0.89 (s, 9H), 0.04 (s, 6H). For *E*-isomer: δ 5.86 (s, 1H), 3.63 (t, *J* = 6.5 Hz, 2H), 2.26 (dd, *J* = 9.0, 7.0 Hz, 2H), 1.79 (s, 3H), 1.69 – 1.61 (m, 2H), 0.90 (s, 9H), 0.06 (s, 6H).

^{13}C NMR (151 MHz, $CDCl_3$): For *Z*-isomer: δ 141.6, 101.3, 62.3, 34.8, 30.8, 26.1, 22.3, 19.2, -5.2. For *E*-isomer: δ 141.7, 100.8, 62.9, 31.1, 30.2, 26.1, 22.3, 18.5, -5.1.

HRMS (ESI): m/z: $[M+H]^+$ calc'd for $C_{12}H_{26}BrOSi^+$: 293.0936. Found: 293.0941.

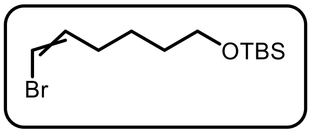

((6-bromohex-5-en-1-yl)oxy)(tert-butyl)dimethylsilane (L3). The reaction was set up using general procedure **D2** with 5-((*tert*-butyldimethylsilyl)oxy)pentanal[9] (1.00 g, 4.62 mmol) for 16 hours. Following this, the crude product was purified using silica column chromatography (hexanes 100%) to yield the indicated product in 62% yield (840 mg, 2.86 mmol, *Z*-isomer) as a colorless liquid.

IR (Diamond-ATR, neat) ṽ (cm^{-1}): 2928, 2857, 1252, 1095, 833, 812, 773.

^{1}H NMR (600 MHz, $CDCl_3$): δ 6.22 – 6.00 (m, 2H), 3.62 (t, *J* = 6.3 Hz, 2H), 2.22 (aq, *J* = 7.1 Hz, 2H), 1.58 – 1.52 (m, 2H), 1.51 – 1.44 (m, 2H), 0.89 (s, 9H), 0.05 (s, 6H).

^{13}C NMR (151 MHz, $CDCl_3$): δ 135.0, 107.9, 63.0, 32.4, 29.6, 26.1, 24.6, 18.5, -5.1.

HRMS (ESI): m/z: $[M+H]^+$ calc'd for $C_{12}H_{26}BrOSi^+$: 293.0936. Found: 293.0941.

General procedure D3: Synthesis of enamide substrate precursors via cross-coupling

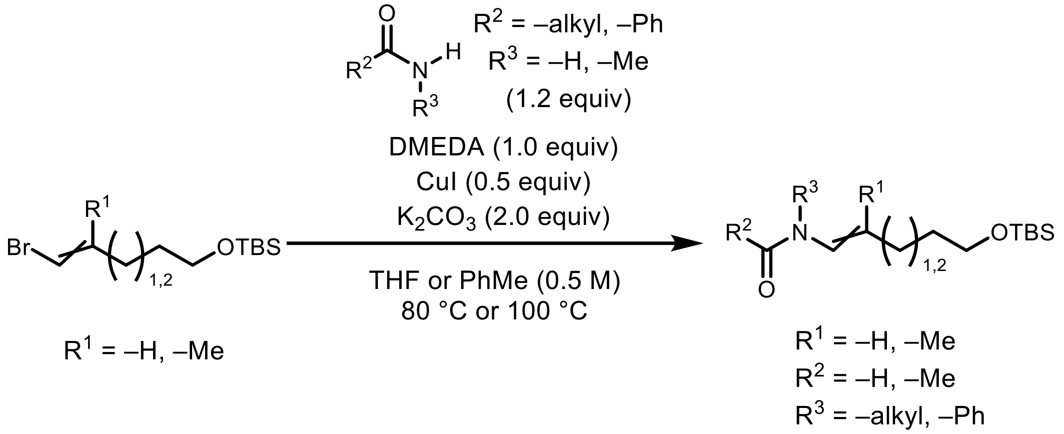

A flame-dried crimp cap vial was charged with magnetic stir bar, CuI (0.5 mmol, 0.5 equiv), K_2CO_3 (2 mmol, 2.0 equiv), and amide (1.2 mmol, 1.2 equiv), sealed with a crimp cap and evacuated and backfilled with nitrogen three times. A separate flame-dried flask was charged with vinyl bromide (1.0 mmol, 1.0 equiv), sealed with rubber septum, and evacuated and backfilled with nitrogen three times. Anhydrous *N*,*N*-dimethylethylenediamine (DMEDA) (1.0 mmol, 1.0 equiv) was added to the flask in a single portion via syringe, followed by the addition of anhydrous THF or PhMe (2.0 mL, 0.5 M) solvent. The resulting solution was degassed for 2 minutes by sparging nitrogen through the solution during sonication. This solution was then transferred into the sealed crimp vial via syringe. The cap of the vial was further reinforced with Teflon tape along with electrical tape. The reaction was subsequently transferred to a pre-heated oil bath and stirred at 80 °C (110 °C if in PhMe). After the reaction was judged to be complete by TLC, it was allowed to cool down to room temperature and filtered through a short plug of silica gel eluting with EtOAc. The filtrate was concentrated, and the residue was purified by column chromatography on silica gel to access the desired product.

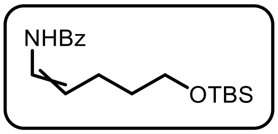

N-(5-((tert-butyldimethylsilyl)oxy)pent-1-en-1-yl)benzamide (43c). The reaction was set up using general procedure **D3** with ((5-bromopent-4-en-1-yl)oxy)(*tert*-butyl)dimethylsilane (838 mg, 3.00 mmol) and benzamide (436 mg, 3.60 mmol) in THF (6 mL, 0.5 M) for 4 hours. Following this, the crude product was purified using silica column chromatography (hexanes/EtOAc 17/83) to yield the indicated product in 71% yield (684 mg) as a white solid (*E:Z*=33:67).

IR (Diamond-ATR, neat) ṽ (cm^{-1}): 3342, 2927, 1652, 1512, 1472.

^{1}H NMR (600 MHz, $CDCl_3$): For *Z*-isomer: δ 7.97 (d, *J* = 10.5 Hz, 1H), 7.79 (dt, *J* = 7.2, 1.5 Hz, 2H), 7.54 – 7.47 (m, 1H), 7.46 – 7.39 (m, 2H), 7.03 – 6.94 (m, 1H), 4.87 (aq, *J* = 8.2 Hz, 1H), 3.67 (t, *J* = 5.8 Hz, 2H), 2.21 (td, *J* = 7.6, 6.2 Hz, 2H), 1.67 – 1.58 (m, 2H), 0.84 (s, 9H), 0.03 (s, 6H). For *E*-isomer: δ 7.81 – 7.45 (m, 3H), 7.54 – 7.47 (m, 1H), 7.47 – 7.40 (m, 2H), 6.97 – 6.94 (m, 1H), 5.32 (dt. *J* = 14.2, 7.1, 1H), 3.63 (t, *J* = 6.3 Hz, 2H), 2.14 (aqd, *J* = 7.2, 1.4 Hz, 2H), 1.67 – 1.58 (m, 2H), 0.90 (s, 9H), 0.05 (s, 6H).

^{13}C NMR (151 MHz, $CDCl_3$): For *Z*-isomer: δ 164.9, 134.3, 132.0, 128.8, 127.3, 122.7, 111.4, 61.7, 32.0, 26.1, 21.6, 18.6, -5.0. For *E*-isomer: δ 164.4, 134.0, 131.9, 128.8, 127.1, 123.2, 113.8, 62.6, 33.1, 26.4, 21.6, 18.5, -5.1.

HRMS (ESI): m/z: $[M+H]^+$ calc'd for $C_{18}H_{30}NO_2Si^+$: 320.2046. Found: 320.2038.

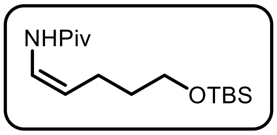

<u>(Z)-N-(5-((tert-butyldimethylsilyl)oxy)pent-1-en-1-yl)pivalamide (44c)</u>. The reaction was set up using general procedure **D3** with ((5-bromopent-4-en-1-yl)oxy)(*tert*-butyl)dimethylsilane (279 mg, 1.00 mmol) and pivalamide (121 mg, 1.20 mmol) in THF (2 mL, 0.5 M) for 8 hours. Following this, the crude product was purified using silica column chromatography (hexanes/EtOAc 10/90) to yield the indicated product in 40% yield (120 mg) as a colorless white solid (*Z*-isomer).

IR (Diamond-ATR, neat) ṽ (cm^{-1}): 2955, 2932, 2857, 2357, 1653, 1490, 1472, 1255, 1180, 1097, 833, 812, 774.

^{1}H NMR (600 MHz, $CDCl_3$): δ 7.23 (t, *J* = 7.5 Hz, 1H), 6.69 (dd, *J* = 10.9, 8.9 Hz, 1H), 4.68 (aq, *J* = 8.0 Hz, 1H), 3.58 (t, *J* = 6.0 Hz, 2H), 2.03 (aq, *J* = 7.0 Hz, 2H), 1.55 (ap, *J* = 6.6 Hz, 2H), 1.18 (s, 9H), 0.84 (s, 9H), 0.00 (s, 6H).

^{13}C NMR (151 MHz, $CDCl_3$): δ 175.6, 122.1, 110.4, 62.0, 39.0, 32.3, 27.5, 26.1, 21.8, 18.5, -5.1.

HRMS (ESI): m/z: $[M+H]^+$ calc'd for $C_{16}H_{34}NO_2Si^+$: 300.2359. Found: 300.2354.

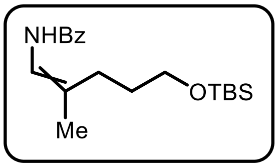

<u>N-(5-((tert-butyldimethylsilyl)oxy)-2-methylpent-1-en-1-yl) benzamide (45c)</u>. The reaction was set up using general procedure **D3** with ((5-bromo-4-methylpent-4-en-1-yl)oxy)(*tert*-butyl)dimethyl silane (293 mg, 1.00 mmol) and benzamide (145 mg, 1.20 mmol) in THF (2 mL, 0.5 M) for 16 hours. Following this, the crude product was purified using silica column chromatography (hexanes/EtOAc 17/83) to yield the indicated product in 81% yield (271 mg) as a colorless sticky liquid (*E:Z*=29:71).

IR (Diamond-ATR, neat) ṽ (cm^{-1}): 2931, 2858, 1646, 1512, 1485, 1255.

^{1}H NMR (600 MHz, $CDCl_3$): For *Z*-isomer: δ 7.80 – 7.77 (m, 2H), 7.53 (d, *J* = 10.4 Hz, 1H), 7.51 – 7.45 (m, 1H), 7.42 (t, *J* = 7.7 Hz, 2H), 6.77 (d, *J* = 10.3 Hz, 1H), 3.61 (t, *J* = 6.5 Hz, 2H), 2.11 (t, *J* = 7.8 Hz, 2H), 1.70 (s, 3H), 1.68 – 1.64 (m, 2H), 0.89 (s, 9H), 0.04 (s, 6H). For *E*-isomer: δ 8.00 (d, *J* = 10.1 Hz, 1H), 7.76 – 7.74 (m, 2H), 7.51 – 7.45 (m, 1H), 7.39 (d, *J* = 7.5 Hz, 2H), 6.80 (d, *J* = 10.0 Hz, 1H), 3.64 – 3.62 (m, 2H), 2.20 (t, *J* = 6.9 Hz, 2H), 1.72 (s, 3H), 1.62 (aq, *J* = 5.5 Hz, 2H), 0.79 (s, 9H), 0.00 (s, 6H).

^{13}C NMR (151 MHz, $CDCl_3$): For *Z*-isomer: δ 164.2, 134.3, 131.7, 128.7, 127.0, 119.7, 117.6, 62.8, 33.2, 31.3, 26.0, 18.4, 15.0, -5.2. For *E*-isomer: δ 164.9, 134.6, 131.6, 128.7, 127.3, 119.5, 118.7, 61.6, 29.7, 26.4, 20.5, 18.6, -5.0.

HRMS (ESI): m/z: $[M+H]^+$ calc'd for $C_{19}H_{32}NO_2Si^+$: 334.2202. Found: 334.2194.

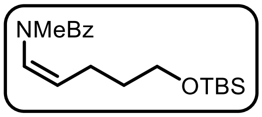

(Z)-N-(5-((tert-butyldimethylsilyl)oxy)pent-1-en-1-yl)-N-methyl benzamide (46c). The reaction was set up using general procedure **D3** with ((5-bromopent-4-en-1-yl)oxy)(*tert*-butyl)dimethylsilane (279 mg, 1.00 mmol) and *N*-methylbenzamide (162 mg, 1.20 mmol) in PhMe (2 mL, 0.5 M) for 16 hours. Following this, the crude product was purified using silica column chromatography (hexanes/EtOAc 17/83) to yield the indicated product in 40% yield (132 mg) as a colorless sticky liquid (*Z*-isomer).

IR (Diamond-ATR, neat) ṽ (cm^{-1}): 2931, 2857, 1639, 1472, 1383, 1332, 1252, 1066, 1027, 941, 833, 774, 698, 659.

^{1}H NMR (500 MHz, DMSO-d_6): δ 7.48 – 7.43 (m, 3H), 7.40 – 7.35 (m, 2H), 6.17 (d, *J* = 8.2 Hz, 1H), 5.04 (q, *J* = 7.6 Hz, 1H), 3.54 (t, *J* = 6.2 Hz, 2H), 3.09 (s, 3H), 1.94 (aq, *J* = 7.2 Hz, 2H), 1.41 (ap, *J* = 6.7 Hz, 2H), 0.87 (s, 9H), 0.03 (s, 6H).

^{13}C NMR (151 MHz, DMSO-d_6): δ 171.1, 136.2, 134.3, 130.2, 128.5, 127.9, 125.0, 62.5, 35.9, 32.0, 26.0, 23.5, 18.4, -5.2.

HRMS (ESI): m/z: $[M+H]^+$ calc'd for $C_{19}H_{32}NO_2Si^+$: 334.2202. Found: 334.2197.

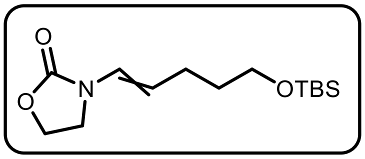

3-(5-((tert-butyldimethylsilyl)oxy)pent-1-en-1-yl)oxazolidin-2-one (47c). The reaction was set up using general procedure **D3** with ((5-bromopent-4-en-1-yl)oxy)(*tert*-butyl)dimethylsilane (279 mg, 1.00 mmol) and oxazolidin-2-one (105 mg, 1.20 mmol) in PhMe (2 mL, 0.5 M) for 16 hours. Following this, the crude product was purified using silica column chromatography (hexanes/EtOAc 25/75) to yield the indicated product in 66% yield (188 mg) as a colorless sticky liquid (*E:Z*=24:76).

IR (Diamond-ATR, neat) ṽ (cm^{-1}): 2932, 2856, 1750, 1669, 1416, 1075, 1039.

^{1}H NMR (600 MHz, $CDCl_3$): For *Z*-isomer: δ 6.22 (d, *J* = 9.5 Hz, 1H), 4.76 (dd, *J* = 9.3, 7.5 Hz, 1H), 4.35 – 4.27 (m, 2H), 4.02 – 3.90 (m, 2H), 3.58 (t, *J* = 6.1 Hz, 2H), 2.21 (aq, *J* = 7.6 Hz, 2H), 1.56 (ap, *J* = 6.0 Hz, 2H), 0.84 (s, 9H), 0.00 (s, 6H). For *E*-isomer: δ 6.60 (d, *J* = 14.3 Hz, 1H), 4.81 – 4.78 (m, 1H), 4.41 – 4.35 (m, 2H), 3.67 – 3.61 (m, 2H), 3.57 (d, *J* = 4.8 Hz, 2H), 2.09 (aq, *J* = 7.2 Hz, 2H), 1.56 (ap, *J* = 6.0 Hz, 2H), 0.85 (s, 9H), 0.00 (s, 6H).

^{13}C NMR (151 MHz, $CDCl_3$): For *Z*-isomer: δ 156.9, 122.8, 114.6, 62.2, 62.0, 45.5, 33.2, 26.2, 25.9, 22.8, -5.3. For *E*-isomer: δ 155.4, 124.0, 110.8, 62.4, 62.2, 42.6, 33.1, 26.2, 26.0, 18.3, -5.3.

HRMS (ESI): m/z: $[M+H]^+$ calc'd for $C_{14}H_{28}NO_3Si^+$: 286.1839. Found: 286.1823.

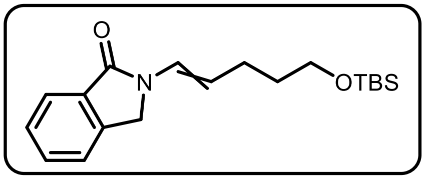

2-(5-((tert-butyldimethylsilyl)oxy)pent-1-en-1-yl)isoindolin-1-one (48c). The reaction was set up using general procedure **D3** with ((5-bromopent-4-en-1-yl)oxy)(*tert*-butyl)dimethylsilane (419 mg, 1.50 mmol) and isoindolin-1-one (500 mg, 1.80 mmol) in PhMe (2 mL, 0.5 M) for 18 hours. Following this, the crude product was purified using silica column chromatography (hexanes/EtOAc 100/0 to hexanes/EtOAc 95/5) to yield the indicated product in 82% yield (449 mg) as a yellow solid (*E:Z*=21:79).

IR (Diamond-ATR, neat) ṽ (cm^{-1}): 2927, 2855, 1691, 1663, 1469, 1393, 1341, 1251.

^{1}H-NMR (400 MHz, $CDCl_3$): For *Z*-isomer: 7.88 (d, *J* = 7.6 Hz, 1H), 7.57 (atd, *J* = 7.4, 1.3 Hz, 1H), 7.50 – 7.45 (m, 2H), 6.80 (d, *J* = 9.8 Hz, 1H), 4.91 (dt, *J* = 9.8, 7.8 Hz, 1H), 4.83 (s, 2H), 3.67 (t, *J* = 6.0 Hz, 2H), 2.44 (aqd, *J* = 7.6, 1.6 Hz, 2H), 1.73 – 1.63 (m, 2H), 1.24 – 1.17 (m, 2H), 0.90 (s, 9H), 0.05 (s, 6H). For *E*-isomer: δ 7.86 (d, *J* = 6.9 Hz, 1H), 7.56 – 7.52 (m, 1H), 7.51 – 7.42 (m, 2H), 7.17 – 7.07 (m, 1H), 5.20 (dt, *J* = 14.3, 7.1 Hz, 1H), 4.49 (s, 2H), 3.67 (t, *J* = 6.0 Hz, 2H), 2.22 (aq, *J* = 7.3 Hz, 2H), 1.70 – 1.61 (m, 2H), 1.24 – 1.17 (m, 2H), 0.91 (s, 9H), 0.06 (s, 6H).

^{13}C-NMR (151 MHz, $CDCl_3$): For *Z*-isomer: δ 166.1, 140.8, 132.6, 132.1, 128.4, 124.1, 123.8, 122.8, 111.4, 62.6, 48.3, 33.3, 26.7, 26.1, 18.5, -5.1. For *E*-isomer: δ 167.8, 141.6, 132.1, 131.5, 128.4, 124.2, 122.7, 122.3, 114.1, 62.1, 51.3, 26.7, 26.1, 23.6, -5.2.

HRMS (ESI): m/z: $[M+H]^+$ calc'd for $C_{19}H_{30}NO_2Si^+$: 332.2046. Found: 332.2004.

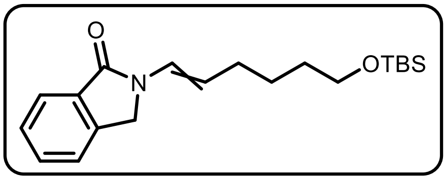

2-(6-((tert-butyldimethylsilyl)oxy)hex-1-en-1-yl)isoindolin-1-one (49c). The reaction was set up using general procedure **D3** with ((6-bromohex-4-en-1-yl)oxy)(*tert*-butyl)dimethylsilane (750 mg, 2.56 mmol) and isoindolin-1-one (409 mg, 3.07 mmol) in PhMe (5.12 mL, 0.5 M) for 24 hours. Following this, the crude product was purified using silica column chromatography (hexanes/EtOAc 100/0 to hexanes/EtOAc 50/50) to yield the indicated product in 37% yield (323 mg) as a yellow solid (*E:Z*=17:83).

IR (Diamond-ATR, neat) ṽ (cm^{-1}): 2953, 2925, 2854, 2258, 1683, 1467, 1258, 1095, 799.

^{1}H NMR (400 MHz, $CDCl_3$): For *Z*-isomer: δ 7.88 (d, *J* = 7.7 Hz, 1H), 7.57 (at, *J* = 7.5 Hz, 1H), 7.49 – 7.45 (m, 2H), 6.80 (d, *J* = 9.9 Hz, 1H), 4.91 (dt, *J* = 8.2, 8.0 Hz, 1H), 4.83 (s, 2H), 3.67 (t, *J* = 6.0 Hz, 2H), 2.43 (aq, *J* = 7.5 Hz, 2H), 1.72 – 1.61 (m, 2H), 0.89 (s, 9H), 0.04 (s, 6H). For *E*-

isomer: δ 7.86 (d, *J* = 7.7 Hz, 1H), 7.58 – 7.54 (m, 1H), 7.49 – 7.45 (m, 2H), 7.14 (d, *J* = 14.4 Hz, 1H), 5.20 (dt, *J* =14.4, 7.2 Hz, 1H), 4.50 (s, 2H), 3.65 (at, *J* = 6.6 Hz, 2H), 2.44 (aq, *J* = 7.3, 2H), 1.72 – 1.61 (m, 2H), 0.90 (s, 9H), 0.06 (s, 6H).

^{13}C NMR (151 MHz, $CDCl_3$): For *Z*-isomer: δ 167.8, 141.6, 132.2, 132.1, 128.4, 124.2, 122.7, 122.3 114.2, 62.2, 51.3, 33.4, 29.8, 26.7, 26.1, 18.5, -5.2. For *E-isomer:* δ 166.1, 140.8, 132.7, 131.6, 128.4, 124.1, 123.8, 122.9, 114.1, 111.4, 62.6, 48.4, 33.4, 29.9, 27.2, 26.1, 23.6, -5.1.

HRMS (ESI): m/z: $[M+H]^+$ calc'd for $C_{20}H_{32}NO_2Si^+$: 346.2202. Found: 346.2199.

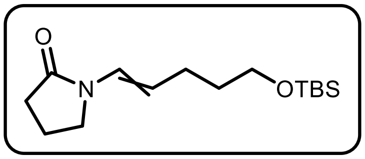

1-(5-((tert-butyldimethylsilyl)oxy)pent-1-en-1-yl)pyrrolidin-2-one (50c). The reaction was set up using general procedure **D3** with ((5-bromopent-4-en-1-yl)oxy)(*tert*-butyl)dimethylsilane (279 mg, 1.00 mmol) and pyrrolidin-2-one (102 mg, 1.2 mmol) in PhMe (2 mL, 0.5 M) for 16 hours. Following this, the crude product was purified using silica column chromatography (hexanes/EtOAc 25/75) to yield the indicated product in 68% yield (192 mg) as a colorless sticky liquid (*E*:*Z*=23:77).

IR (Diamond-ATR, neat) ṽ (cm^{-1}): 2930, 2856, 1699, 1412, 1255, 1098, 833, 774.

^{1}H NMR (600 MHz, $CDCl_3$): For *Z*-isomer: δ 6.40 (d, *J* = 9.7 Hz, 1H), 4.83 (dt, *J* = 9.9, 7.7 Hz, 1H), 3.78 (t, *J* = 7.1 Hz, 2H), 3.61 (at, *J* = 6.2 Hz, 2H), 2.40 (at, *J* = 8.2 Hz, 2H), 2.25 (aqd, *J* = 7.6, 1.6 Hz, 2H), 2.09 – 2.02 (m, 2H), 1.64 – 1.55 (m, 2H), 0.88 (s, 9H), 0.03 (s, 6H). For *E*-isomer: δ 6.87 (d, *J* = 14.4 Hz, 1H), 4.94 (adt, *J* = 14.3, 7.1 Hz, 1H), 3.60 (t, *J* = 6.5 Hz, 2H), 3.48 (at, *J* = 7.2 Hz, 2H), 2.47 (at, *J* = 8.1 Hz, 2H), 2.12 (aq, *J* = 6.8 Hz, 2H), 2.09 – 2.05 (m, 2H), 1.62 – 1.55 (m, 2H), 0.88 (s, 9H), 0.03 (s, 6H).

^{13}C NMR (151 MHz, $CDCl_3$): For *Z*-isomer: δ 174.8, 122.7, 116.0, 62.3, 48.7, 33.4, 30.4, 26.0, 23.6, 18.7, 18.4, -5.2. For *E*-isomer: δ 173.0, 123.9, 112.1, 62.6, 45.4, 33.3, 31.4, 26.6, 26.1, 18.4, 17.6, -5.2.

HRMS (ESI): m/z: $[M+H]^+$ calc'd for $C_{15}H_{30}NO_2Si^+$: 284.2046. Found: 284.2049.

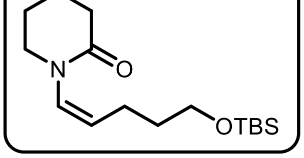

(Z)-1-(5-((tert-butyldimethylsilyl)oxy)pent-1-en-1-yl)piperidin-2-one (51c). The reaction was set up using general procedure **D3** with ((5-bromopent-4-en-1-yl)oxy)(*tert*-butyl)dimethylsilane (279 mg, 1.00 mmol) and piperidin-2-one (119 mg, 1.20 mmol) in PhMe (2 mL, 0.5 M) for 16 hours. Following this, the crude product was purified using silica column chromatography (hexanes/EtOAc 25/75) to yield the indicated product in 45% yield (134 mg) as a colorless sticky liquid (*Z*-isomer).

IR (Diamond-ATR, neat) ṽ (cm^{-1}): 2932, 2857, 1641, 1412, 1250, 1099, 834, 752, 666.

1H NMR (600 MHz, $CDCl_3$): δ 6.23 (d, *J* = 8.6 Hz, 1H), 5.16 (aq, *J* = 7.7 Hz, 1H), 3.58 (t, *J* = 6.2 Hz, 2H), 3.42 (aq, *J* = 4.1 Hz, 2H), 2.41 (aq, *J* = 4.6 Hz, 2H), 2.09 (aqd, *J* = 7.4, 1.8 Hz, 2H), 1.87 – 1.74 (m, 4H), 1.57 (ap, *J* = 6.5 Hz, 2H), 0.85 (s, 9H), 0.01 (s, 6H).

13C NMR (151 MHz, $CDCl_3$): δ 169.8, 128.3, 124.5, 62.5, 49.9, 32.4, 32.4, 26.0, 23.9, 23.3, 21.2, 18.4, -5.2.

HRMS (ESI): m/z: $[M+H]^+$ calc'd for $C_{16}H_{32}NO_2Si^+$: 298.2202. Found: 298.2200.

Synthesis of Intramolecular PMP Styrene substrate (41a)

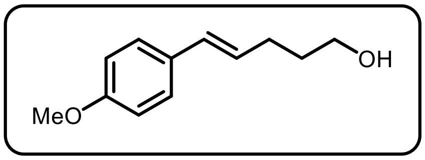

(E)-5-(4-methoxyphenyl)pent-4-en-1-ol (41a). The substrate was synthesized according to a known procedure.[5] The resulting product has been previously reported and matches the obtained spectroscopic data tabulated below.[5]

1H NMR (600 MHz, $CDCl_3$): δ 7.27 (d, *J* = 8.7 Hz, 2H), 6.84 (d, *J* = 8.7 Hz, 2H), 6.36 (d, *J* = 15.7 Hz, 1H), 6.08 (dt, *J* = 15.7, 7.9 Hz, 1H), 3.80 (s, 3H), 3.70 (s, 3H), 2.28 (aqd, *J* = 7.3 Hz, 2H), 1.74 (aqt, *J* = 7.1 Hz, 2H).

13C NMR (151 MHz, $CDCl_3$): 158.9, 130.6, 129.9, 128.0, 127.2, 114.1, 62.6, 55.4, 32.5, 29.4.

Synthesis of Intramolecular silyl enol ether substrate (42a)

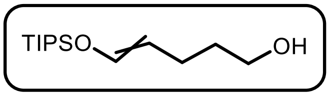

5-((triisopropylsilyl)oxy)pent-4-en-1-ol (42a). 5-oxopentenyl acetate was synthesized according to a known procedure.[10] Spectral properties are consistent with those previously reported. A flame-dried, 25-mL round-bottomed flask was equipped with a magnetic stir bar and sealed with a rubber septum, evacuated and backfilled with nitrogen three times and placed under a nitrogen atmosphere. Anhydrous CH_2Cl_2 (14.0 mL, 0.25 M) was transferred into the flask via syringe. 5-oxopentenyl acetate (500 mg, 3.47 mmol. 1.0 equiv) was added to the flask in a single portion via syringe, and the resulting solution was cooled to 0 °C by transferring the reaction apparatus to an ice-water bath. After stirring at this temperature for 5 minutes, NEt_3 (870 µL, 6.2 mmol, 1.8 equiv) and TIPSOTf (1.00 mL, 3.8 mmol, 1.1 equiv) (in this order) were added dropwise via syringe transfer over the course of 2 minutes.

After stirring at 0 °C for 30 minutes, the reaction was warmed up to room temperature by transferring the reaction apparatus out of the ice-water bath. The reaction solution was concentrated under pressure by rotary evaporation to provide crude oil. The crude was purified by column chromatography on basic alumina (hexanes 100%) to access 5-((triisopropylsilyl)oxy)pent-4-en-1-yl-acetate 73% yield (561 mg) as a colorless liquid (*E:Z*=9:91). A flame-dried, 10-mL round-bottomed flask charged with 5-((triisopropylsilyl)oxy)pent-4-en-1-yl-acetate (441 mg, 1.5 mmol, 1.0 equiv) was equipped with a magnetic stir bar and sealed with a rubber septum, evacuated and backfilled with nitrogen three times and placed under a nitrogen atmosphere. Anhydrous MeOH (6.3 mL, 0.23 M) was transferred into the flask via syringe. The septum was removed and K_2CO_3 (1.05 g, 7.6 mmol, 5.2 equiv) was quickly added to the flask in a single portion and the septum was replaced. The reaction was then stirred until judged to be

complete by TLC. Following this, the crude reaction mixture was concentrated under reduced pressure by rotary evaporation to provide a crude oil. The crude was purified by column chromatography on basic alumina (hexanes 100%) to access 5-((triisopropylsilyl)oxy)pent-4-en-1-ol (**42a**) in 75% (220 mg) yield. The resulting product has been previously reported and matches the obtained spectroscopic data tabulated below.[11]

¹H NMR (600 MHz, $CDCl_3$): For *Z*-isomer: δ 6.33 (d, *J* = 11.8 Hz, 1H), 5.02 – 4.92 (m, 1H), 4.07 (t, *J* = 6.7 Hz, 2H), 2.17 (aq, *J* = 7.9 Hz, 2H), 2.03 (s, 3H), 1.68 (ap, *J* = 6.8 Hz, 2H), 1.20 – 1.03 (m, 21H). For *E*-isomer: 6.30 (d, *J* = 5.7 Hz, 1H), 4.39 (aq, *J* = 7.3 Hz, 1H), 4.07 (t, *J* = 6.7 Hz, 2H), 2.17 (aq, *J* = 7.9 Hz, 2H), 2.03 (s, 3H), 1.68 (ap, *J* = 6.8 Hz, 2H), 1.20 – 1.03 (m, 21H).

¹³C NMR (151 MHz, $CDCl_3$): For *Z*-isomer: δ 171.4, 141.5, 109.7, 64.1, 29.5, 23.9, 21.2, 18.2, 17.9, 17.9, 17.9, 12.1. For *E*-isomer: 171.4, 140.0, 108.3, 64.5, 28.8, 21.2, 20.2, 17.9, 12.0.

General procedure D4: Synthesis of enamide substrates via TBS deprotection

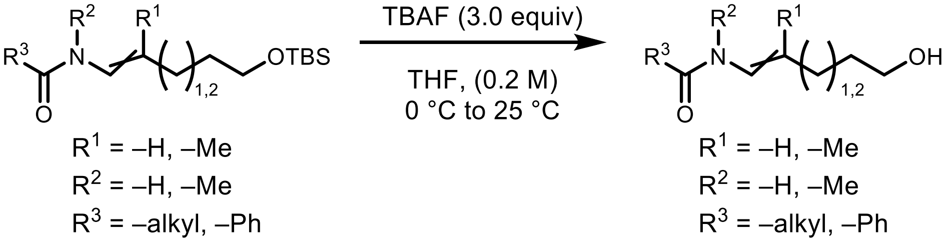

A flame-dried screw cap vial charged with TBS-protected enamide (1.0 equiv), sealed with a screw cap, and was evacuated and backfilled with nitrogen three times. Anhydrous THF (0.20 M) was added to the solid via syringe, and the resulting solution was cooled to 0 °C through transfer of the reaction vial into an ice-water bath. TBAF (1.0 M solution in THF, 3.0 equiv) was added dropwise into the reaction mixture and allowed to stir at room temperature following the addition. After 3 hours, full consumption of starting material was observed by TLC. At this juncture, the reaction was diluted with EtOAc (20 mL), washed with brine (2 × 20 mL), and H_2O (20 mL). The organic phase was collected, dried over anhydrous Na_2SO_4, and concentrated *in vacuo*. The crude mixture was purified by column chromatography to afford the intramolecular enamide substrate.

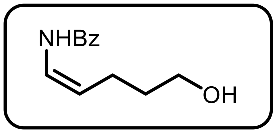

(Z)-N-(5-hydroxypent-1-en-1-yl)benzamide (43a). The reaction was set up using general procedure **D4** with *N*-(5-((*tert*-butyldimethylsilyl)oxy)pent-1-en-1-yl)benzamide (408 mg, 1.99 mmol) for 3 hours. Following this, the crude product was purified using silica column chromatography (hexanes/EtOAc 50/50) to yield the indicated product in 80% yield (324 mg) as a white solid (*Z*-isomer).

IR (Diamond-ATR, neat) ṽ (cm^{-1}): 3408, 3287, 2938, 2868, 1822, 1411, 1306, 1174, 1057, 719.

¹H NMR (600 MHz, $CDCl_3$): δ 8.89 (d, *J* = 10.4 Hz, 1H), 7.91 – 7.84 (m, 2H), 7.50 – 7.45 (m, 1H), 7.40 (at, *J* = 7.8 Hz, 2H), 6.99 (at, *J* = 9.5 Hz, 1H), 4.87 (aq, *J* = 8.5 Hz, 1H), 3.70 (t, *J* = 5.7 Hz, 2H), 2.32 – 2.26 (m, 2H), 2.29 (br s, 1H), 1.68 (ap, *J* = 5.7 Hz, 2H).

13C NMR (151 MHz, $CDCl_3$): δ 165.0, 133.9, 131.9, 128.7, 127.4, 124.0, 110.9, 60.2, 30.8, 21.2.

HRMS (ESI): m/z: $[M+H]^+$ calc'd for $C_{12}H_{16}NO_2^+$: 184.1338. Found: 184.1329.

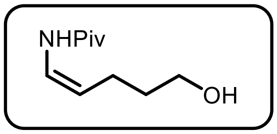

(Z)-N-(5-hydroxypent-1-en-1-yl)pivalamide (44a). The reaction was set up using general procedure **D4** with *(Z)*-*N*-(5-((*tert*-butyldimethylsilyl)oxy)pent-1-en-1-yl)pivalamide (369 mg, 1.99 mmol) for 3 hours. Following this, the crude product was purified using silica column chromatography (hexanes/EtOAc 50/50) to yield the indicated product in 72% yield (80 mg) as a white solid (*Z*-isomer).

IR (Diamond-ATR, neat) ṽ (cm^{-1}): 3335, 2938, 2868, 1822, 1411, 1306, 1174, 1057, 719.

1H NMR (600 MHz, $CDCl_3$): δ 7.96 (d, *J* = 10.0 Hz, 1H), 6.78 (at, *J* = 9.6 Hz, 1H), 4.73 (aq, *J* = 8.4 Hz, 1H), 3.66 (t, *J* = 5.9 Hz, 2H), 2.18 (aq, *J* = 7.4 Hz, 2H), 1.73 (br s, 1H), 1.65 (ap, *J* = 6.0 Hz, 2H), 1.22 (s, 9H).

13C NMR (151 MHz, $CDCl_3$): δ 176.4, 123.7, 109.8, 60.5, 39.1, 31.1, 27.5, 21.2.

HRMS (ESI): m/z: $[M+H]^+$ calc'd for $C_{10}H_{20}NO_2^+$: 186.1694. Found: 186.1489.

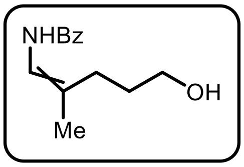

N-(5-hydroxy-2-methylpent-1-en-1-yl)benzamide (45a). The reaction was set up using general procedure **D4** with *N*-(5-((*tert*-butyldimethylsilyl)oxy)-2-methylpent-1-en-1-yl)benzamide (271 mg, 0.81 mmol) for 3 hours. Following this, the crude product was purified using silica column chromatography (hexanes/EtOAc 50/50) to yield the indicated product in 91% yield (162 mg) as a white solid (*E:Z*=40:60).

IR (Diamond-ATR, neat) ṽ (cm^{-1}): 3376, 2970, 2930, 1635, 1533, 1490, 1288, 1285.

1H NMR (600 MHz, $CDCl_3$): For *Z*-isomer: δ 8.98 (d, *J* = 9.5 Hz, 1H), 7.83 – 7.75 (m, 2H), 7.52 (at, *J* = 7.3 Hz, 1H), 7.45 (at, *J* = 7.6 Hz, 2H), 6.79 (d, *J* = 10.8 Hz, 1H), 3.66 (t, *J* = 6.4 Hz, 2H), 2.16 (t, *J* = 7.6 Hz, 2H), 2.12 (br s, 1H), 1.76 – 1.69 (m, 5H). For *E*-isomer: δ 7.91 – 7.86 (m, 2H), 7.48 (s, 1H), 7.47 (d, *J* = 7.1 Hz, 1H), 7.39 (at, *J* = 7.6 Hz, 2H), 6.82 (d, *J* = 9.4 Hz, 1H), 3.67 (t, *J* = 5.5 Hz, 2H), 2.33 – 2.28 (m, 2H), 2.12 (br s, 1H), 1.77 – 1.68 (m, 5H).

13C NMR (151 MHz, $CDCl_3$): For *Z*-isomer: δ 164.4, 134.3, 131.9, 128.9, 127.1, 119.8, 117.8, 62.5, 33.1, 30.9, 14.9. For *E*-isomer: δ 164.7, 134.3, 131.6, 128.6, 127.4, 119.6, 119.4, 60.0, 28.5, 26.2, 20.5.

HRMS (ESI): m/z: $[M+H]^+$ calc'd for $C_{13}H_{18}NO_2^+$: 220.13376. Found: 220.1329.

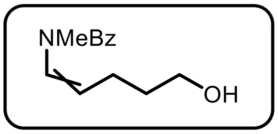

N-(5-hydroxypent-1-en-1-yl)-N-methylbenzamide (46a). The reaction was set up using general procedure **D4** with *N*-(5-((*tert*-butyldimethylsilyl)oxy)pent-1-en-1-yl)-*N*-methylbenzamide (132 mg, 0.40 mmol) for 3 hours. Following this, the crude product was purified using silica column chromatography (hexanes/EtOAc 50/50) to yield the indicated product in 83% yield (72.0 mg) as a white solid (*E:Z*=36:64).

IR (Diamond-ATR, neat) ṽ (cm^{-1}): 3422, 2938, 3868, 1822, 1411, 1306, 1174, 1057, 719.

^{1}H NMR (500 MHz, DMSO-d_6): For *Z*-isomer: δ 7.50 – 7.34 (m, 5H), 6.15 (d, *J* = 8.1 Hz, 1H), 5.03 (aq, *J* = 7.6 Hz, 1H), 4.28 (t, *J* = 5.1 Hz, 1H), 3.38 – 3.32 (m, 2H), 3.09 (s, 3H), 1.98 – 1.89 (m, 2H), 1.36 (ap, *J* = 6.8 Hz, 2H). For *E*-isomer: δ 7.73 – 7.33 (m, 6H), 5.14 (dt, *J* = 14.2, 7.1 Hz, 1H), 4.23 (t, *J* = 5.1 Hz, 1H), 3.39 (d, *J* = 5.9 Hz, 2H), 3.09 (s, 3H), 2.00 (br s, 2H), 1.47 (br s, 2H).

^{13}C NMR (151 MHz, DMSO-d_6): For *Z*-isomer: δ 169.5, 136.1, 129.7, 129.6, 128.2, 127.6, 127.2, 60.0, 32.7, 31.4, 22.8. For *E*-isomer: δ 169.7, 135.6, 131.5, 130.6, 128.7, 127.5, 125.5, 60.0, 35.2, 30.0, 26.2.

HRMS (ESI): m/z: $[M+H]^+$ calc'd for $C_{13}H_{18}NO_2^+$: 220.1338. Found: 220.1327.

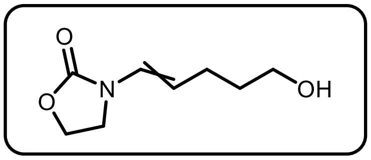

3-(5-hydroxypent-1-en-1-yl)oxazolidin-2-one (47a). The reaction was set up using general procedure **D4** with 3-(5-((*tert*-butyldimethylsilyl)oxy)pent-1-en-1-yl)oxazolidin-2-one (184.4 mg, 0.65 mmol) for 3 hours. Following this, the crude product was purified using silica column chromatography (EtOAc/MeOH 100/0 to EtOAc/MeOH 90/10) to yield the indicated product in 93% yield (103 mg) as a colorless liquid.

IR (Diamond-ATR, neat) ṽ (cm^{-1}): 3408, 2931, 2362, 2341, 1730, 1418, 1243, 1073, 1036, 724.

^{1}H NMR (600 MHz, $CDCl_3$): For *Z*-isomer: δ 6.20 (d, *J* = 9.4 Hz, 1H), 4.87 (dt, *J* = 9.6, 7.7 Hz, 1H), 4.40 – 4.35 (m, 2H), 3.96 (dd, *J* = 8.6, 7.4 Hz, 2H), 3.66 (t, *J* = 5.4 Hz, 2H), 2.34 – 2.24 (m, 2H), 1.89 (br s, 1H), 1.69 – 1.63 (m, 2H). For *E*-isomer: δ 6.66 (dt, *J* = 14.3, 1.4 Hz, 1H), 4.82 (dt, *J* = 14.4, 7.2 Hz, 1H), 4.45 – 4.40 (m, 2H), 3.96 (ddd, *J* = 9.1, 7.2, 1.1 Hz, 2H), 3.70 – 3.67 (m, 2H), 2.16 (aqd, *J* = 7.3, 1.4 Hz, 2H), 1.76 (br s, 1H), 1.70 – 1.62 (m, 2H).

^{13}C NMR (151 MHz, $CDCl_3$): For *Z*-isomer: δ 157.1, 123.0, 116.5, 62.4, 61.7, 45.8, 32.8, 23.0. For *E*-isomer: 155.6, 124.4, 110.6, 62.3, 62.2, 42.7, 33.0, 26.2.

HRMS (ESI): m/z: $[M+H]^+$ calc'd for $C_8H_{14}NO_3^+$: 172.0974. Found: 172.0966.

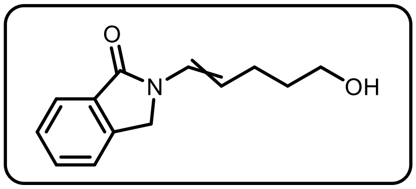

2-(5-hydroxypent-1-en-1-yl)isoindolin-1-one (48a). The reaction was set up using general procedure **D4** with 2-(5-((*tert*-butyldimethylsilyl)oxy)pent-1-en-1-yl)isoindolin-1-one (448.7 mg, 1.35 mmol) for 2 hours. Following this, the crude product was purified using silica column chromatography (hexanes/EtOAc 100/0 to hexanes/EtOAc 50/50) to yield the indicated product in 71% yield (210 mg) as a white solid (*E:Z*=17:83).

IR (Diamond-ATR, neat) ṽ (cm^{-1}): 3386, 2953, 2925, 2854, 2358, 1683, 1470, 1258.

^{1}H NMR (400 MHz, $CDCl_3$): For *Z*-isomer: δ 7.88 (dd, *J* = 7.3, 1.6 Hz, 1H), 7.57 (td, *J* = 7.6, 1.1 Hz, 1H), 7.47 (at, *J* = 7.5 Hz, 2H), 6.65 (dt, *J* = 9.5, 1.8 Hz, 1H), 5.01 (dt, *J* = 9.6, 7.6 Hz, 1H), 4.77 (s, 2H), 3.73 (t, *J* = 6.1 Hz, 2H), 2.45 (aqd, *J* = 7.3, 1.8 Hz, 2H), 2.07 (s, 1H), 1.78 – 1.73 (m, 2H). For *E*-isomer: δ 7.90 – 7.83 (m, 1H), 7.57 (atd, *J* = 7.6, 1.1 Hz, 1H), 7.47 (at, *J* = 7.5 Hz, 2H), 7.16 (d, *J* = 14.4 Hz, 1H), 5.21 (dt, *J* = 14.4, 7.1 Hz, 1H), 4.50 (s, 2H), 3.73 (t, *J* = 6.1 Hz, 2H), 2.26 (aqd, *J* = 7.3, 1.4 Hz, 2H), 1.80 – 1.68 (m, 2H), 1.57 (s, 1H).

^{13}C NMR (151 MHz, $CDCl_3$): For *Z*-isomer: δ 167.8, 141.6, 132.2, 131.6 128.4, 124.2, 122.8, 122.6, 117.1, 61.8, 51.6, 32.9, 23.7. For *E*-isomer: δ 166.3, 140.8, 132.5, 132.2, 128.4, 124.1, 124.0, 122.9, 111.0, 62.3, 48.4, 33.1, 26.6.

HRMS (ESI): m/z: $[M+H]^+$ calc'd for $C_{13}H_{16}NO_2^+$: 218.1181. Found: 218.1178.

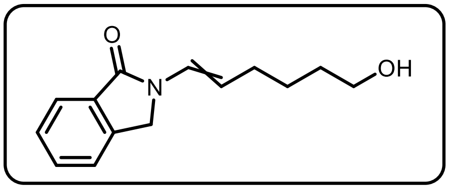

2-(6-hydroxyhex-1-en-1-yl)isoindolin-1-one (49a). The reaction was set up using general procedure **D4** with 2-(5-((*tert*-butyldimethylsilyl)oxy)hex-1-en-1-yl)isoindolin-1-one (128.7 mg, 0.39 mmol) for 1.5 hours. Following this, the crude product was purified using silica column chromatography (hexanes/EtOAc 100/0 to hexanes/EtOAc 50/50) to yield the indicated product in 94% yield (78 mg) as a white solid (*E:Z*=30:70).

IR (Diamond-ATR, neat) ṽ (cm^{-1}): 3393, 2928, 2859, 2359, 1670, 1469, 1152.

^{1}H NMR (400 MHz, $CDCl_3$): For *Z*-isomer: δ 7.92 – 7.84 (m, 1H), 7.57 (aqd, *J* = 6.9, 1.2 Hz, 1H), 7.49 (at, *J* = 7.1 Hz, 2H), 6.72 (d, *J* = 9.8 Hz, 1H), 4.97 (dt, *J* = 9.7, 7.5 Hz, 1H), 4.77 (s, 2H), 3.69 (t, *J* = 6.2 Hz, 2H), 2.39 (aqd, *J* = 7.4, 1.7 Hz, 2H), 1.71 – 1.60 (m, 5H). For *E*-isomer: δ 7.89 – 7.84 (m, 1H), 7.70 – 7.63 (m, 1H), 7.51 – 7.43 (m, 2H), 7.15 (d, *J* = 14.4 Hz, 1H), 5.19 (dt, *J* = 14.4, 7.1 Hz, 1H), 4.51 (s, 2H), 3.68 (t, *J* = 6.3 Hz, 2H), 2.24 – 2.14 (m, 2H), 1.60 – 1.47 (m, 5H).

^{13}C NMR (151 MHz, $CDCl_3$): For *Z*-isomer: δ 167.8, 141.5, 132.2, 128.5, 128.4, 124.3, 122.8, 122.3, 116.0, 62.8, 51.5, 32.4, 27.1, 26.5. For *E*-isomer: δ 166.2, 140.8, 131.6, 128.7, 128.6, 124.2, 123.9, 122.9, 111.4, 62.9, 48.4, 32.3, 30.3, 26.4.

HRMS (ESI): m/z: $[M+H]^+$ calc'd for $C_{14}H_{18}NO_2^+$: 232.1337. Found: 232.1328.

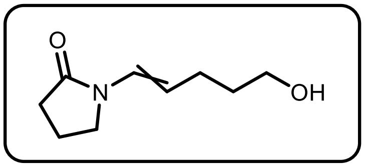

1-(5-hydroxypent-1-en-1-yl)pyrrolidin-2-one (50a). The reaction was set up using general procedure **D4** with 1-(5-((*tert*-butyldimethylsilyl)oxy)pent-1-en-1-yl)pyrrolidin-2-one (187 mg, 0.66 mmol) for 3 hours. Following this, the crude product was purified using silica column chromatography (EtOAc/MeOH 100/0 to EtOAc/MeOH 90/10) to yield the indicated product in 99% yield (112 mg) as a colorless liquid (*E:Z*=23:77).

IR (Diamond-ATR, neat) ṽ (cm^{-1}): 3385, 2937, 1660, 1414, 1269, 1056.

^{1}H NMR (600 MHz, $CDCl_3$): For *Z*-isomer: δ 6.23 (dt, *J* = 9.5, 1.7 Hz, 1H), 4.95 – 4.90 (m, 1H), 3.74 – 3.68 (m, 2H), 3.64 (t, *J* = 6.0 Hz, 2H), 2.41 (t, *J* = 8.2 Hz, 2H), 2.30 (br s, 1H), 2.27 (aqd, *J* = 7.3, 1.7 Hz, 2H), 2.07 (t, *J* = 7.2 Hz, 2H), 1.65 (ddd, *J* = 13.2, 7.2, 6.0 Hz, 2H). For *E*-isomer: δ 6.88 (d, *J* = 14.4 Hz, 1H), 4.94 (add, *J* = 13.2, 5.9 Hz, 2H), 3.63 (ad, *J* = 5.7 Hz, 2H), 3.48 (t, *J* = 7.2 Hz, 2H), 2.46 (t, *J* = 8.1 Hz, 2H), 2.18 – 2.13 (m, 2H), 2.11 – 2.09 (m, 1H), 1.89 (br s, 1H), 1.65 – 1.61 (m, 2H).

^{13}C NMR (151 MHz, $CDCl_3$): For *Z*-isomer: δ 174.7, 123.0, 118.8, 61.8, 49.0, 32.8, 30.6, 23.7, 18.6. For *E*-isomer: δ 173.0, 124.1, 111.7, 62.2, 45.4, 33.0, 31.4, 26.5, 17.5.

HRMS (ESI): m/z: $[M+H]^+$ calc'd for $C_9H_{16}NO_2^+$: 170.1181. Found: 170.1177.

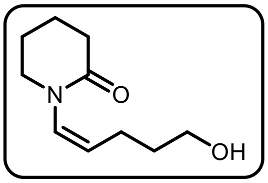

(Z)-1-(5-hydroxypent-1-en-1-yl)piperidin-2-one (51a). The reaction was set up using general procedure **D4** with *(Z)*-1-(5-((*tert*-butyldimethylsilyl)oxy)pent-1-en-1-yl)piperidin-2-one (142 mg, 0.48 mmol) for 3 hours. Following this, the crude product was purified using silica column chromatography (EtOAc/MeOH 100/0 to EtOAc/MeOH 90/10) to yield the indicated product in 85% yield (74.6 mg) as a colorless liquid.

IR (Diamond-ATR, neat) ṽ (cm^{-1}): 3404, 2938, 3868, 1822, 1411, 1306, 1174, 1057, 719.

^{1}H NMR (600 MHz, $CDCl_3$): δ 6.02 (dt, *J* = 8.4, 1.8 Hz, 1H), 5.30 (dt, *J* = 8.7, 7.3 Hz, 1H), 3.62 (t, *J* = 6.0 Hz, 2H), 3.40 – 3.33 (m, 2H), 2.85 (br s, 1H), 2.43 (aq, *J* = 4.5 Hz, 2H), 2.12 (aqd, *J* = 7.1, 1.8 Hz, 2H), 1.82 (ap, *J* = 3.2 Hz, 4H), 1.65 (ddd, *J* = 12.9, 7.0, 5.9 Hz, 2H).

^{13}C NMR (151 MHz, $CDCl_3$): δ 170.1, 128.2, 127.5, 61.7, 50.0, 32.4, 31.8, 23.7, 23.1, 21.2.

HRMS (ESI): m/z: $[M+H]^+$ calc'd for $C_{10}H_{18}NO_2^+$: 184.1338. Found: 184.1329.

ii) NMR Spectra of Intramolecular Substrates

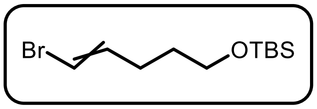

((5-bromopent-4-en-1-yl)oxy)(tert-butyl)dimethylsilane (L1)

1H NMR (600 MHz, $CDCl_3$):

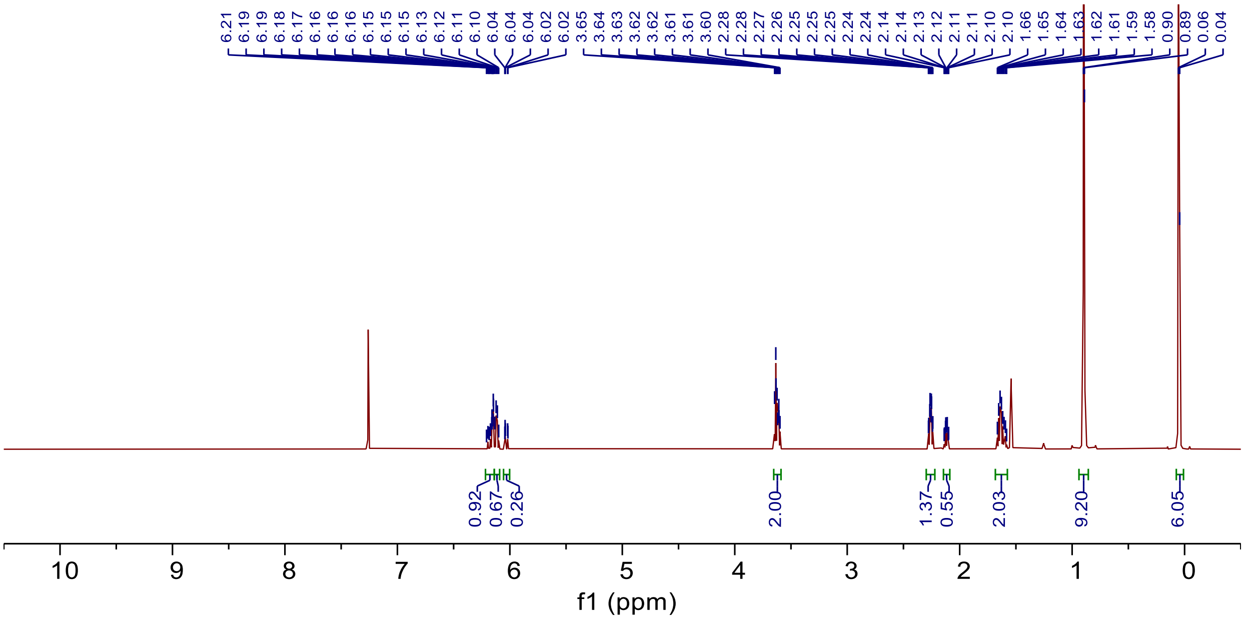

13C NMR (151 MHz, $CDCl_3$):

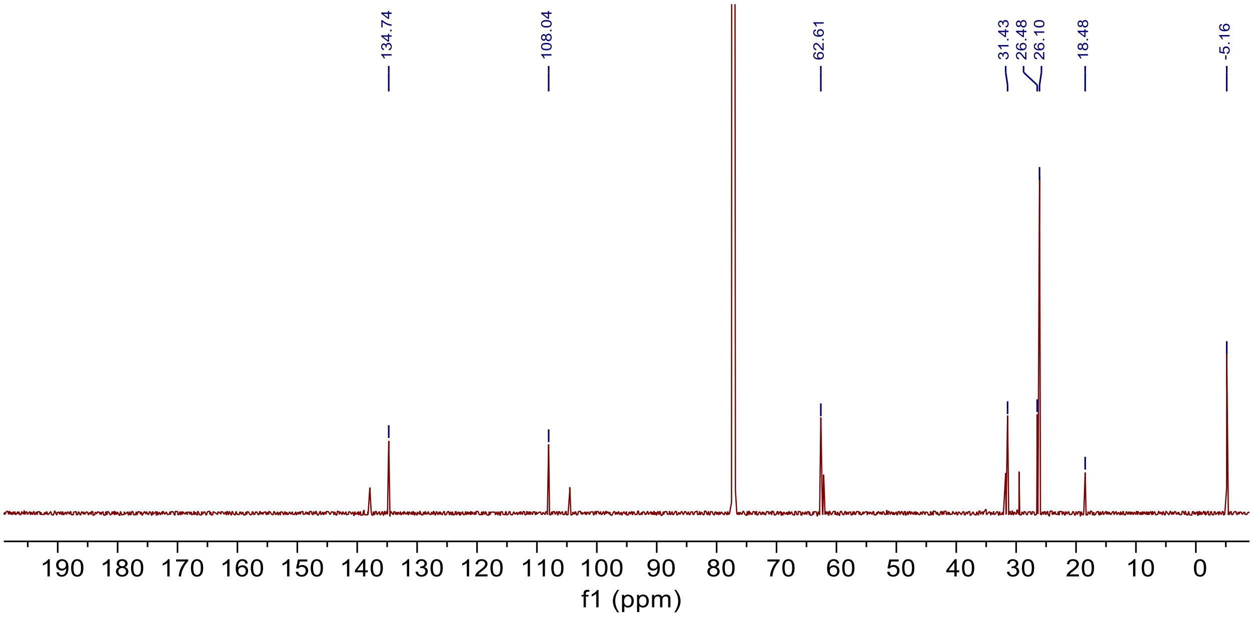

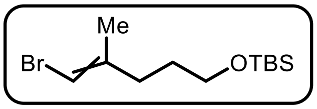

((5-bromo-4-methylpent-4-en-1-yl)oxy)(tert-butyl)dimethyl silane (L2)

¹H NMR (600 MHz, $CDCl_3$):

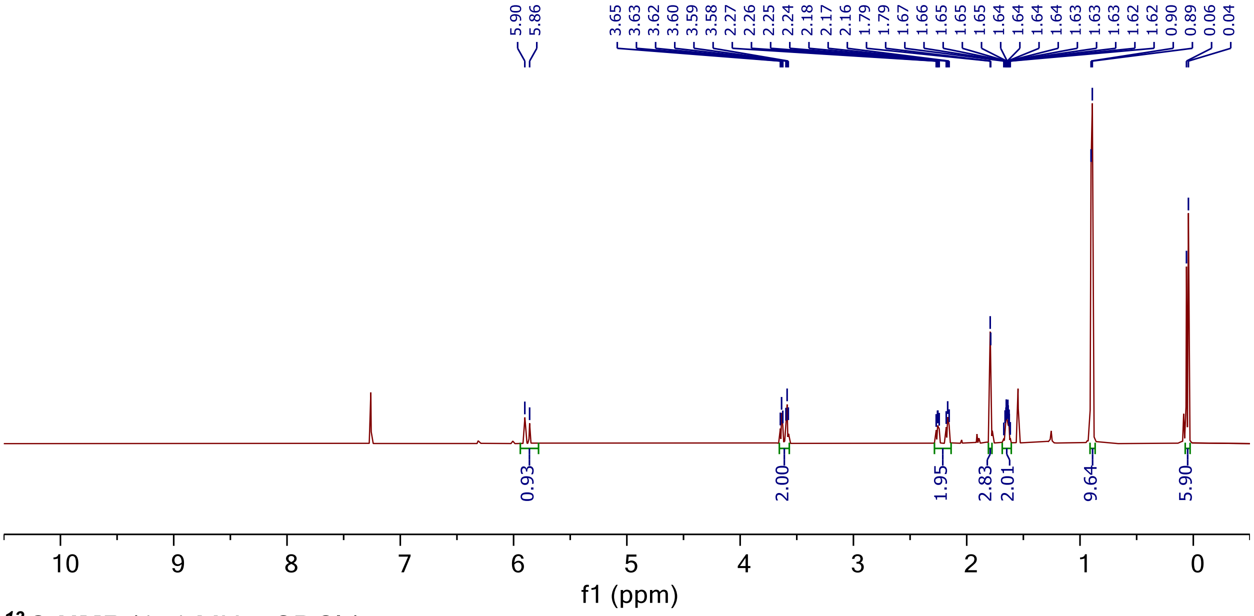

¹³C NMR (151 MHz, $CDCl_3$):

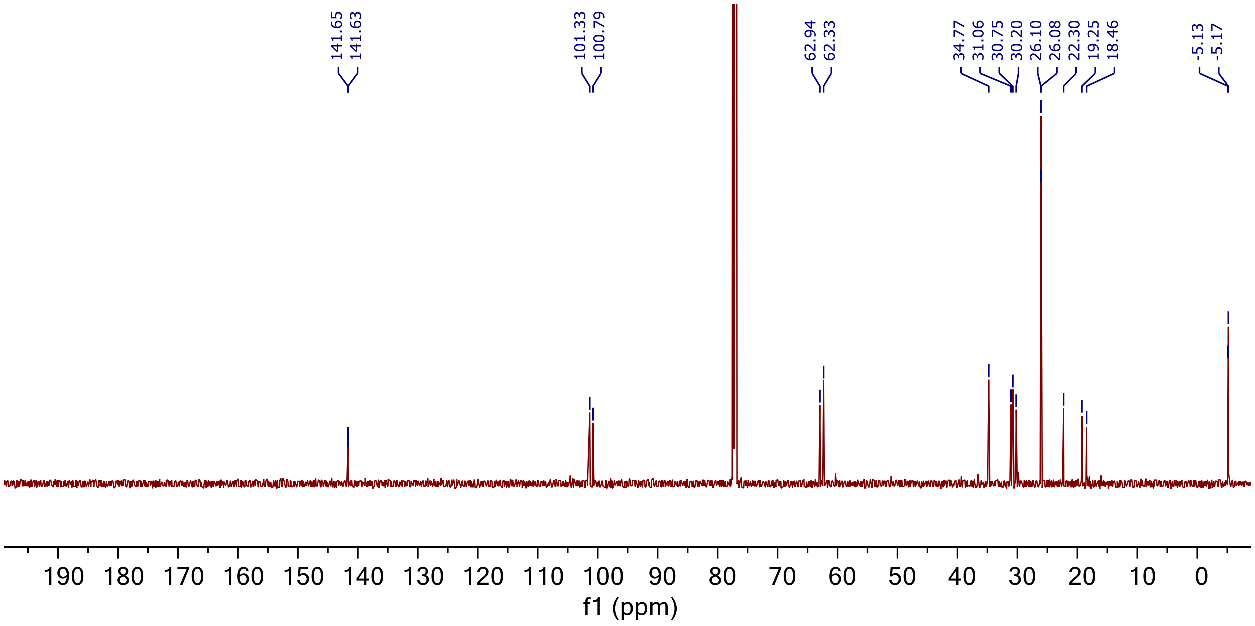

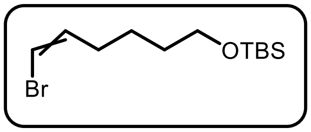

((6-bromohex-5-en-1-yl)oxy)(tert-butyl)dimethylsilane (L3)

^{1}H NMR (600 MHz, $CDCl_3$):

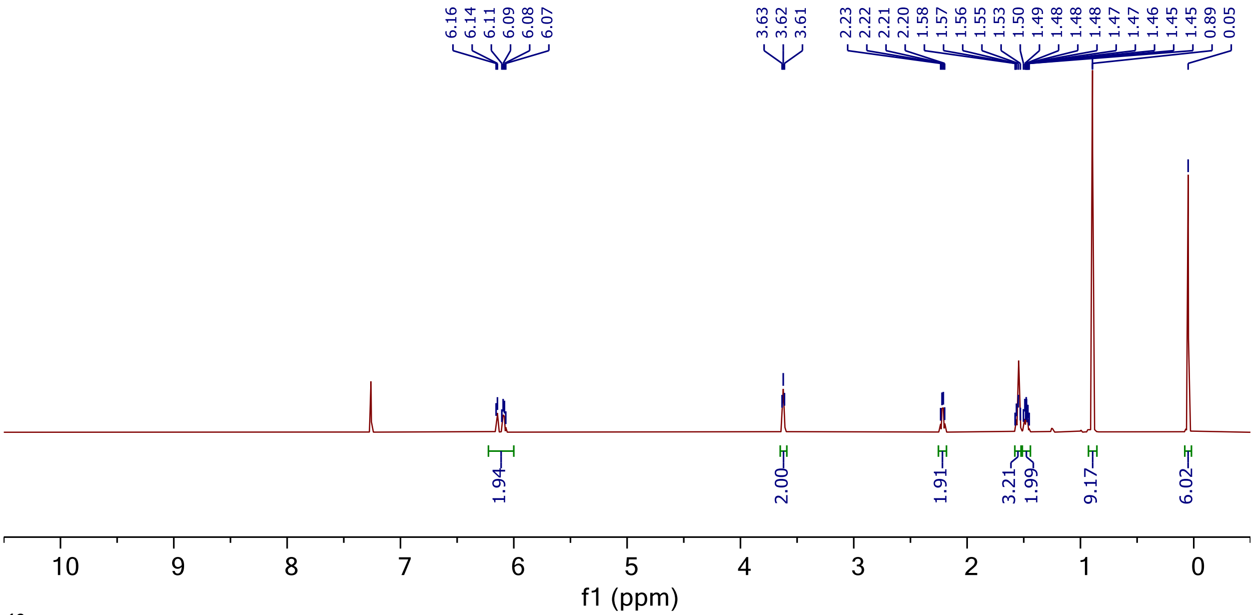

^{13}C NMR (151 MHz, $CDCl_3$):

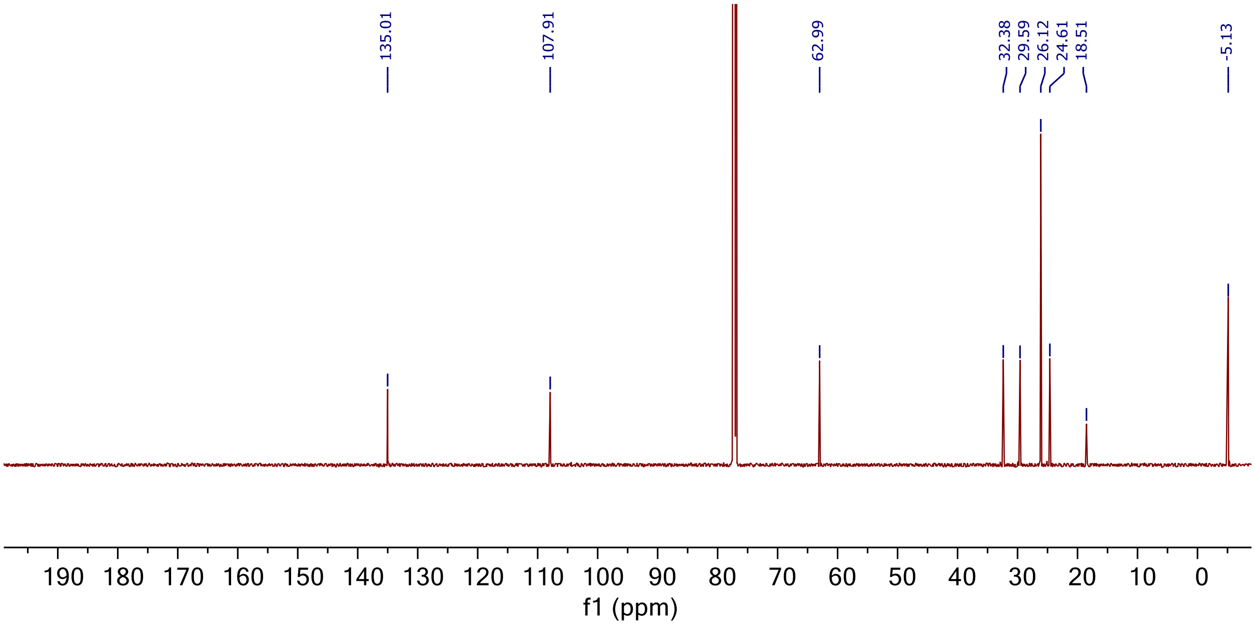

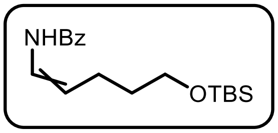

N-(5-((tert-butyldimethylsilyl)oxy)pent-1-en-1-yl)benzamide (43d).

^{1}H NMR (600 MHz, $CDCl_3$):

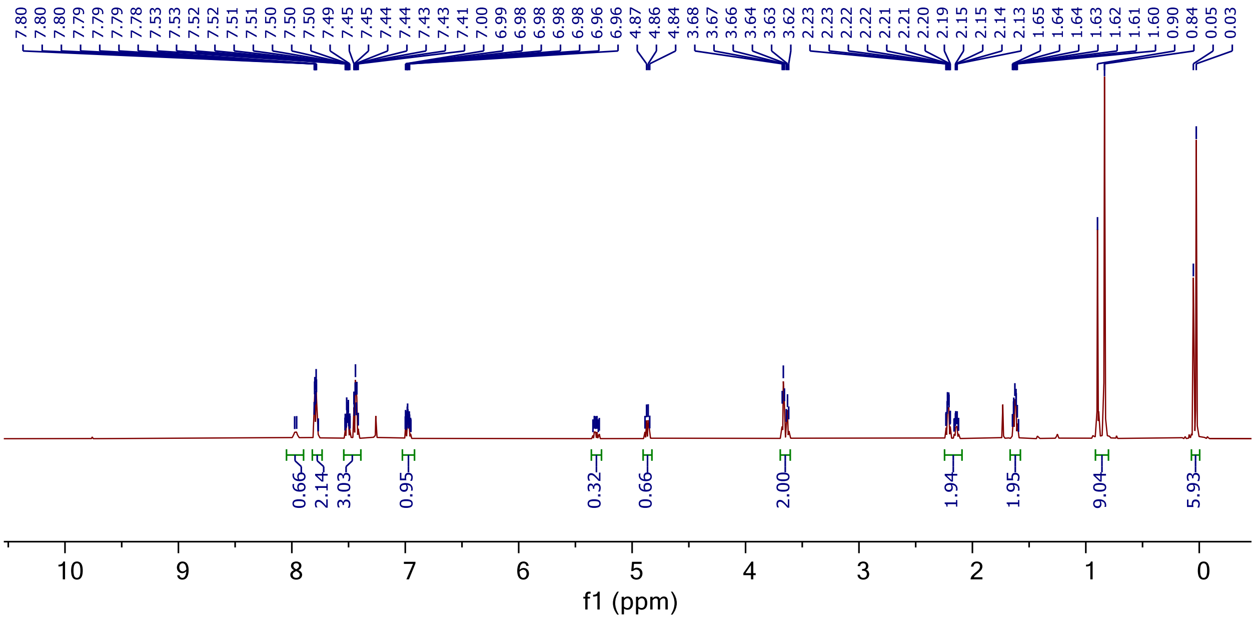

^{13}C NMR (151 MHz, $CDCl_3$):

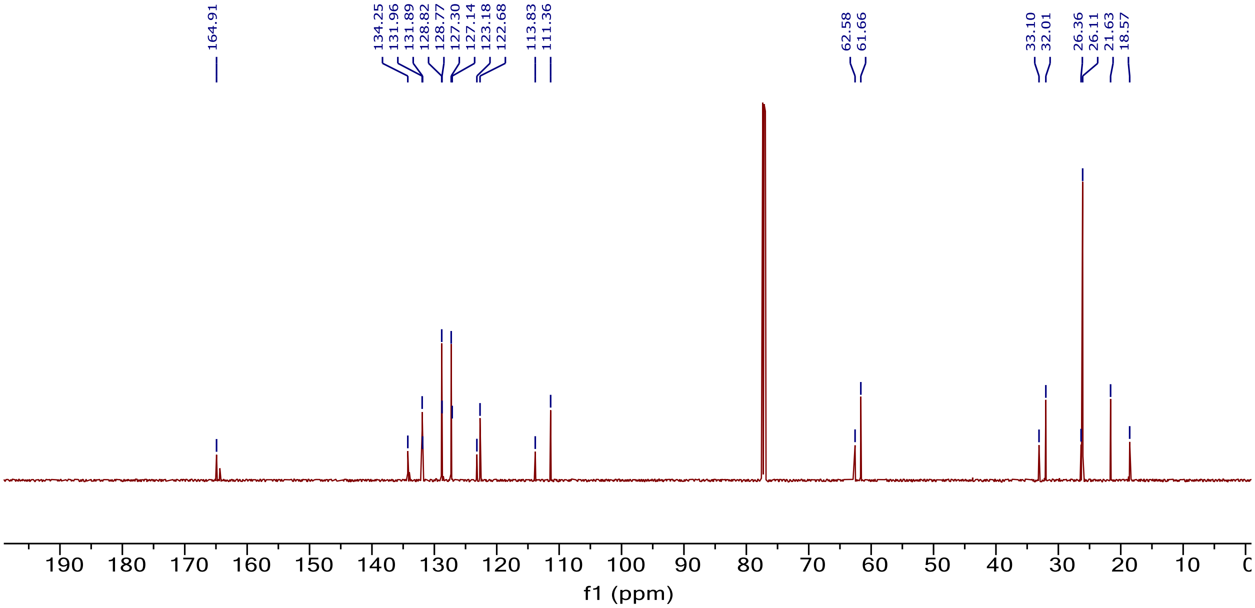

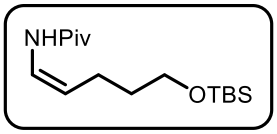

(Z)-N-(5-((tert-butyldimethylsilyl)oxy)pent-1-en-1-yl)pivalamide (44c).

1H NMR (600 MHz, $CDCl_3$):

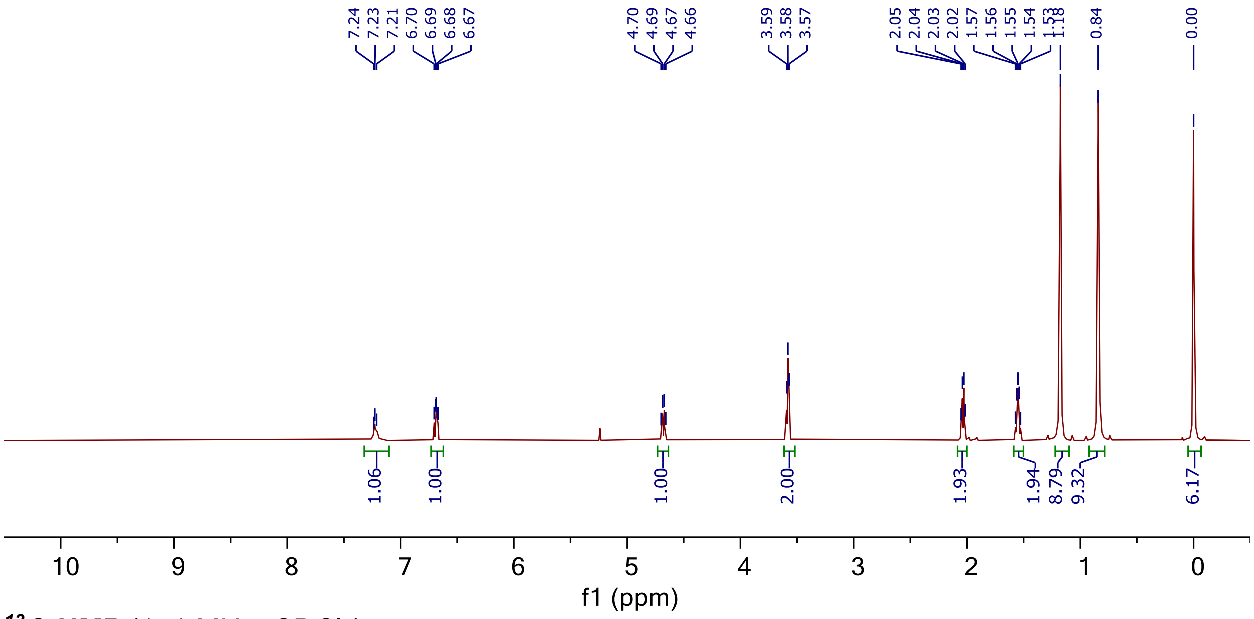

13C NMR (151 MHz, $CDCl_3$):

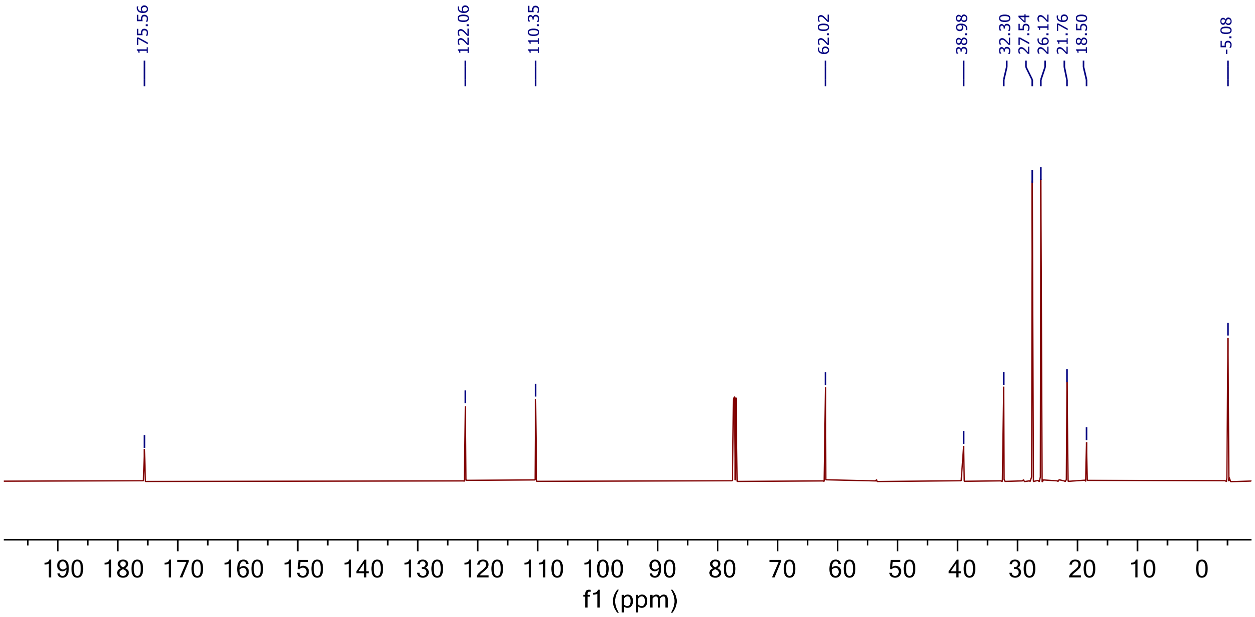

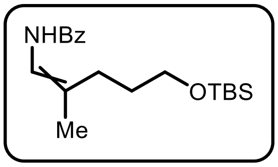

N-(5-((tert-butyldimethylsilyl)oxy)-2-methylpent-1-en-1-yl)benzamide (45c).

^{1}H NMR (600 MHz, $CDCl_3$):

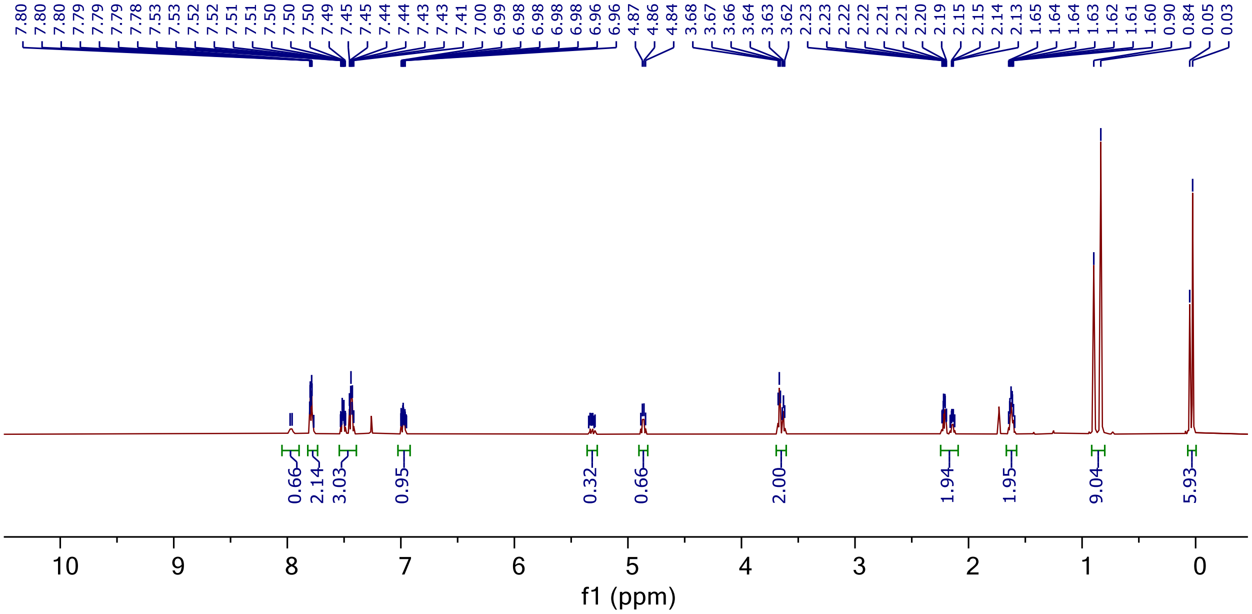

^{13}C NMR (151 MHz, $CDCl_3$):

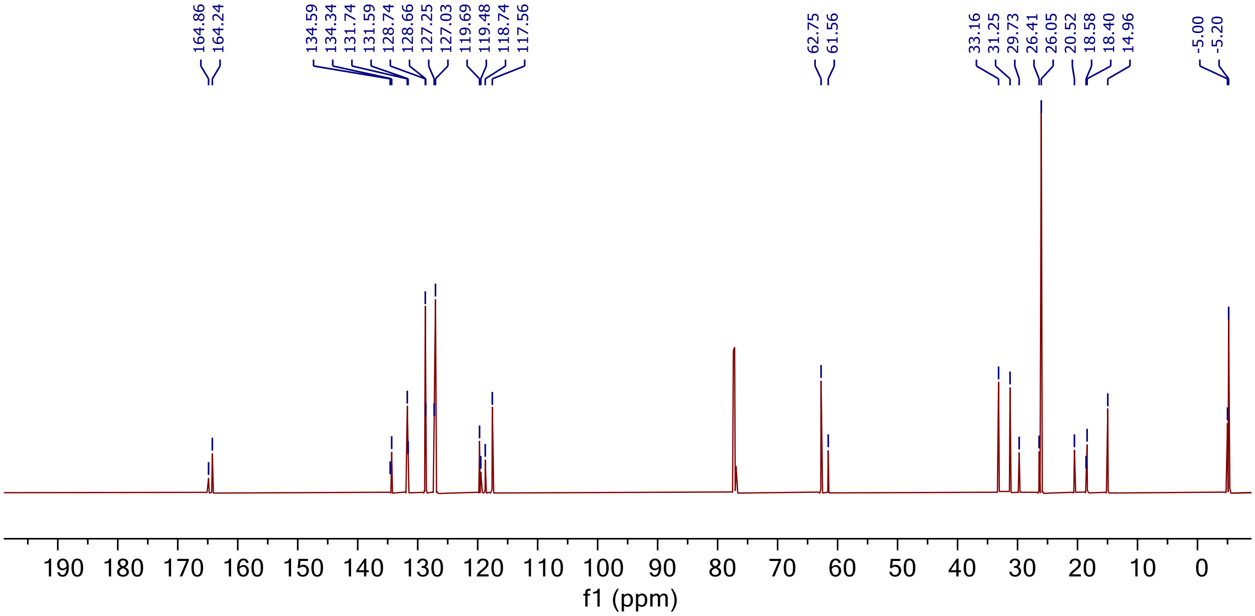

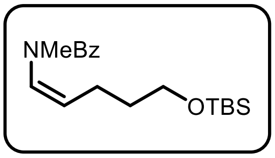

(Z)-N-(5-((tert-butyldimethylsilyl)oxy)pent-1-en-1-yl)-N methylbenzamide (46c).

^{1}H NMR (600 MHz, DMSO-*d_6*):

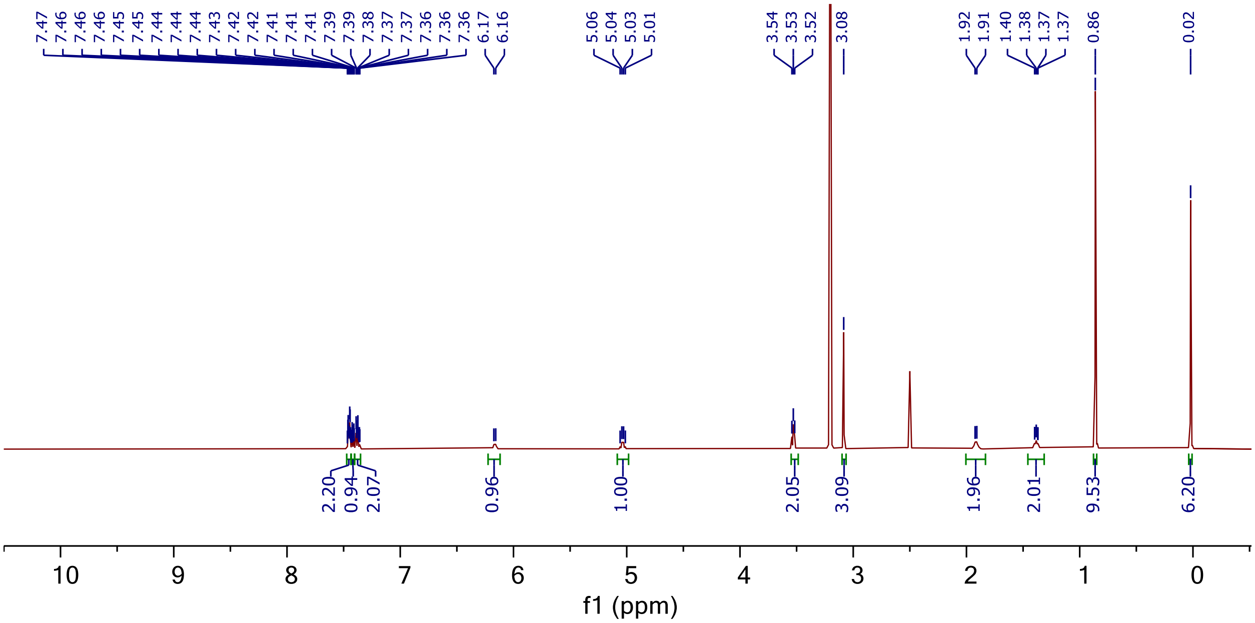

^{13}C NMR (151 MHz, DMSO-*d_6*):

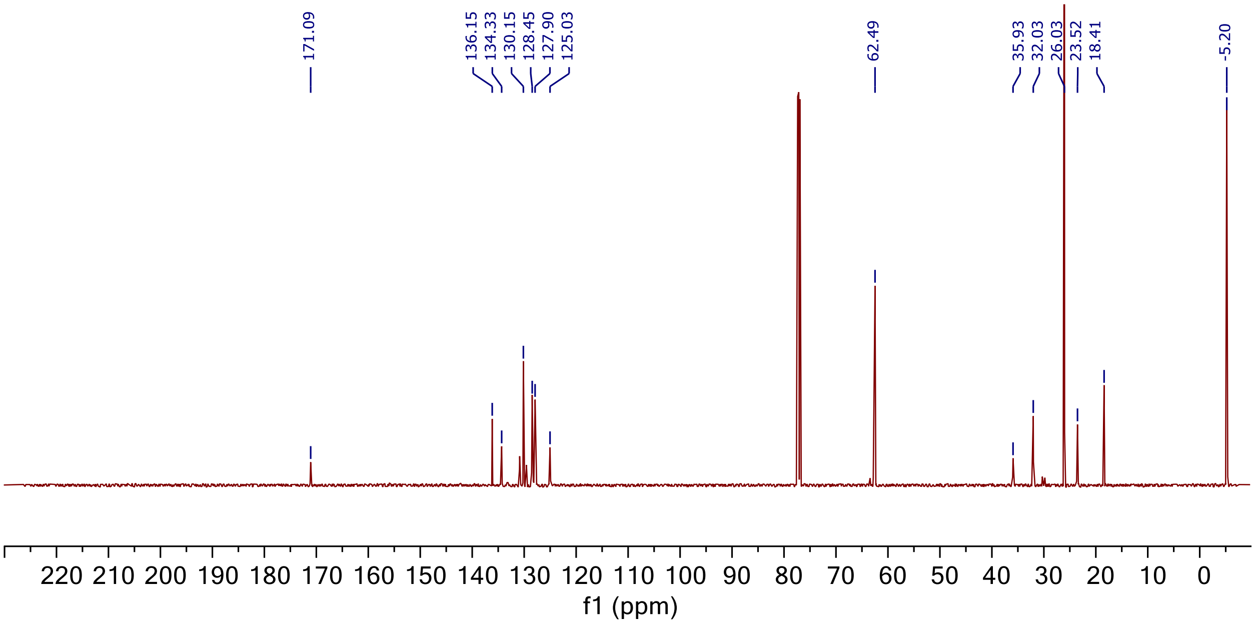

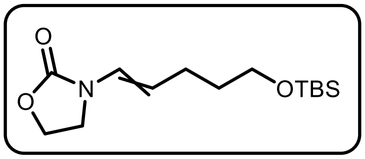

3-(5-((tert-butyldimethylsilyl)oxy)pent-1-en-1-yl)oxazolidin-2-one (47c).

^{1}H NMR (600 MHz, $CDCl_3$):

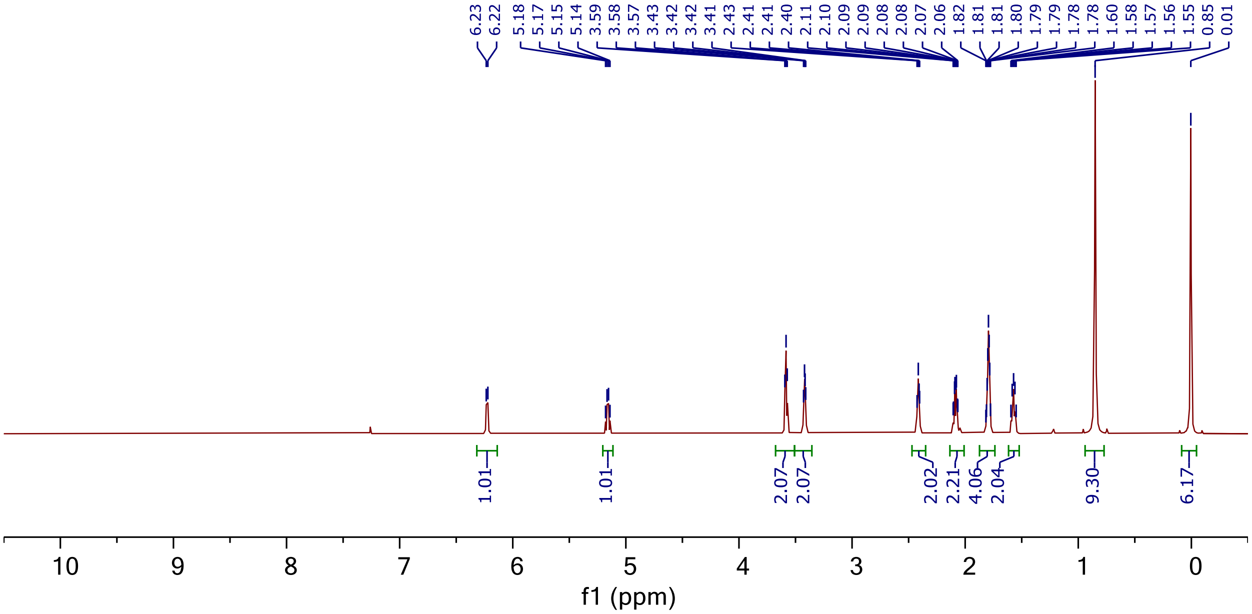

^{13}C NMR (151 MHz, $CDCl_3$):

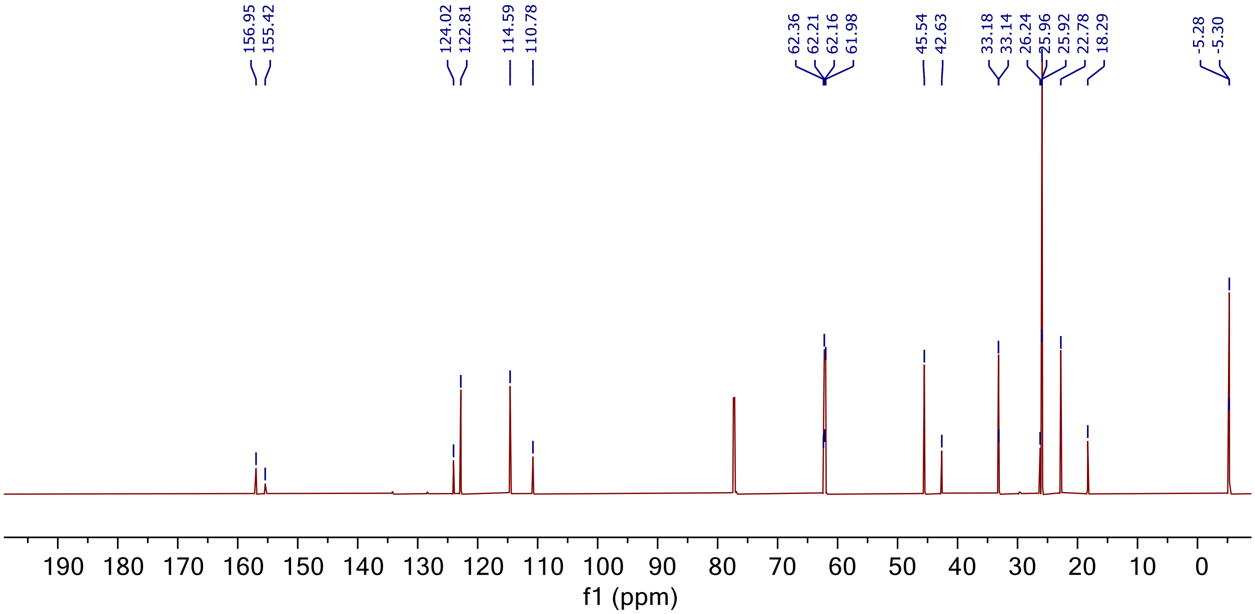

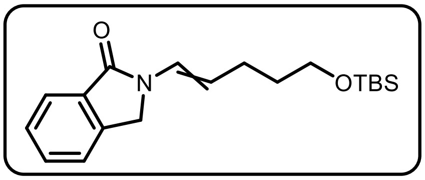

2-(5-((tert-butyldimethylsilyl)oxy)pent-1-en-1-yl)isoindolin-1-one (48c)

^{1}H NMR (600 MHz, $CDCl_3$):

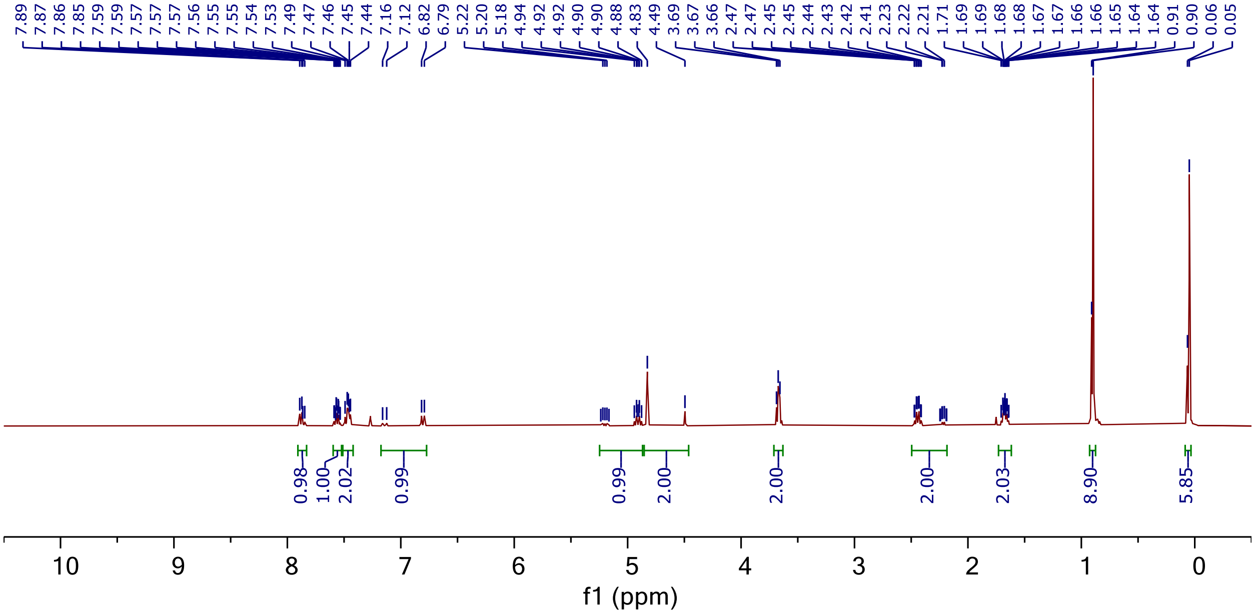

^{13}C NMR (151 MHz, $CDCl_3$):

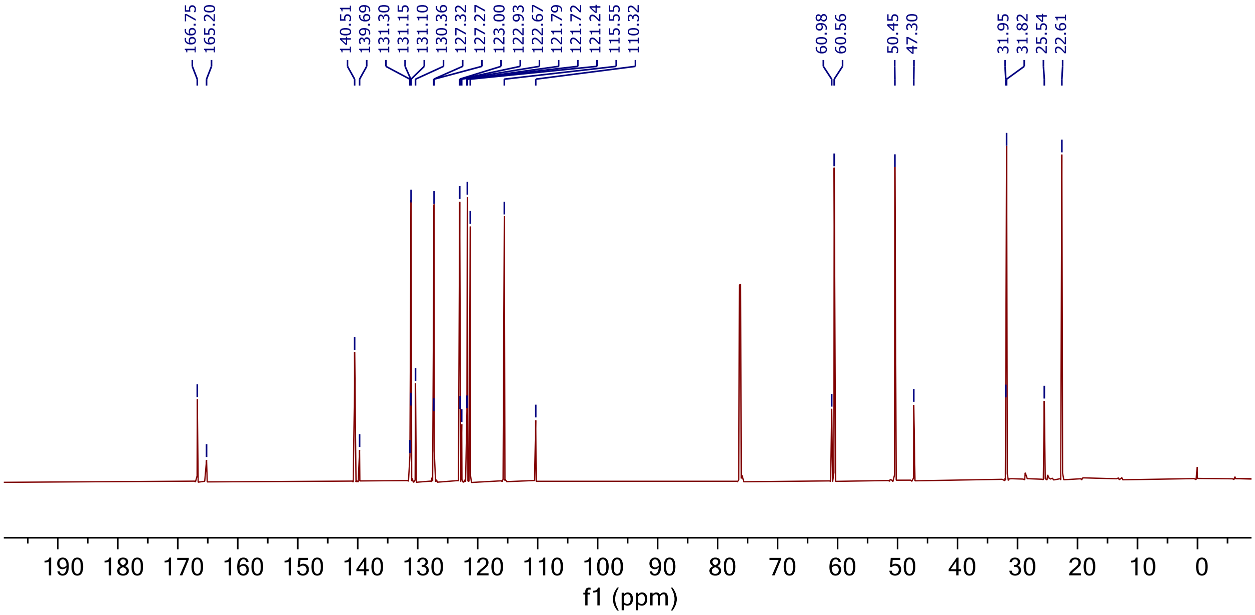

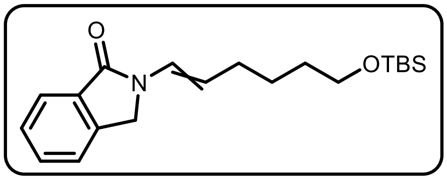

2-(6-((tert-butyldimethylsilyl)oxy)hex-1-en-1-yl) isoindolin-1-one (49c).

^{1}H NMR (600 MHz, $CDCl_3$):

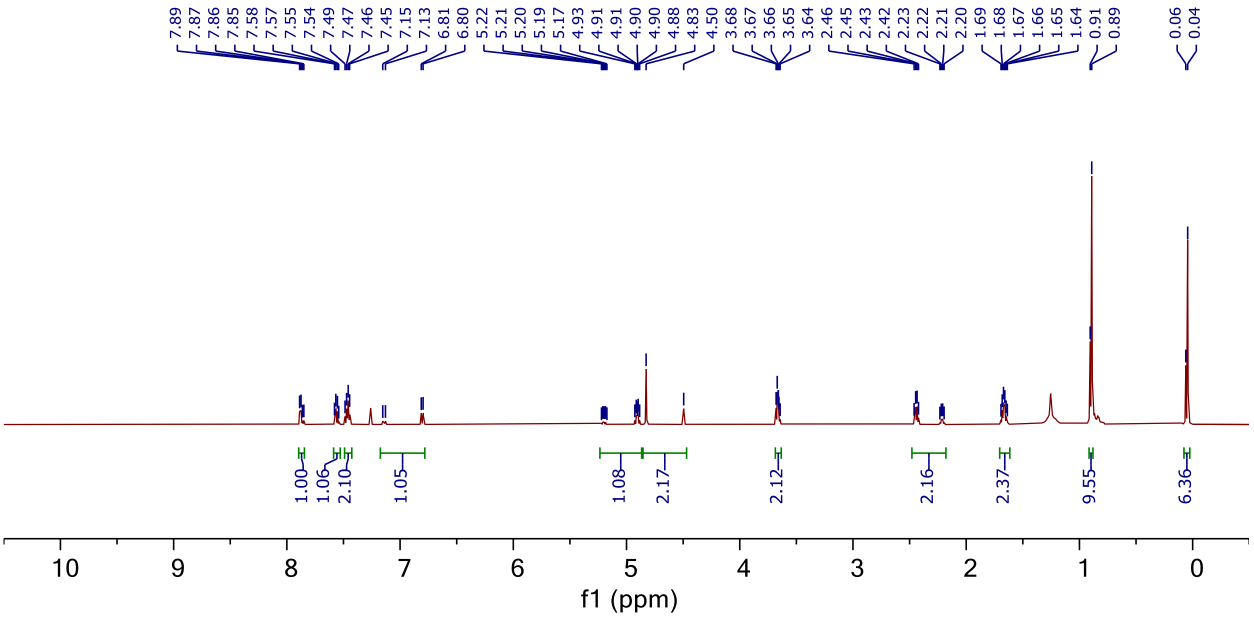

^{13}C NMR (151 MHz, $CDCl_3$):

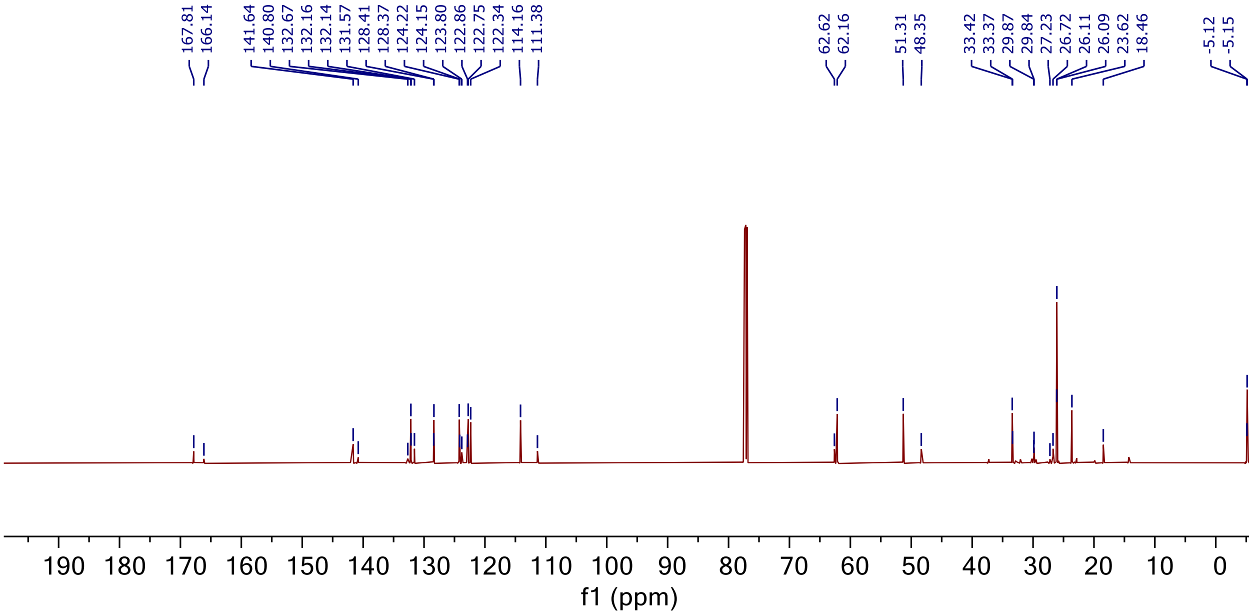

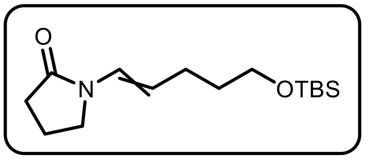

1-(5-((tert-butyldimethylsilyl)oxy)pent-1-en-1-yl)pyrrolidin-2-one (50c).

^{1}H NMR (600 MHz, $CDCl_3$):

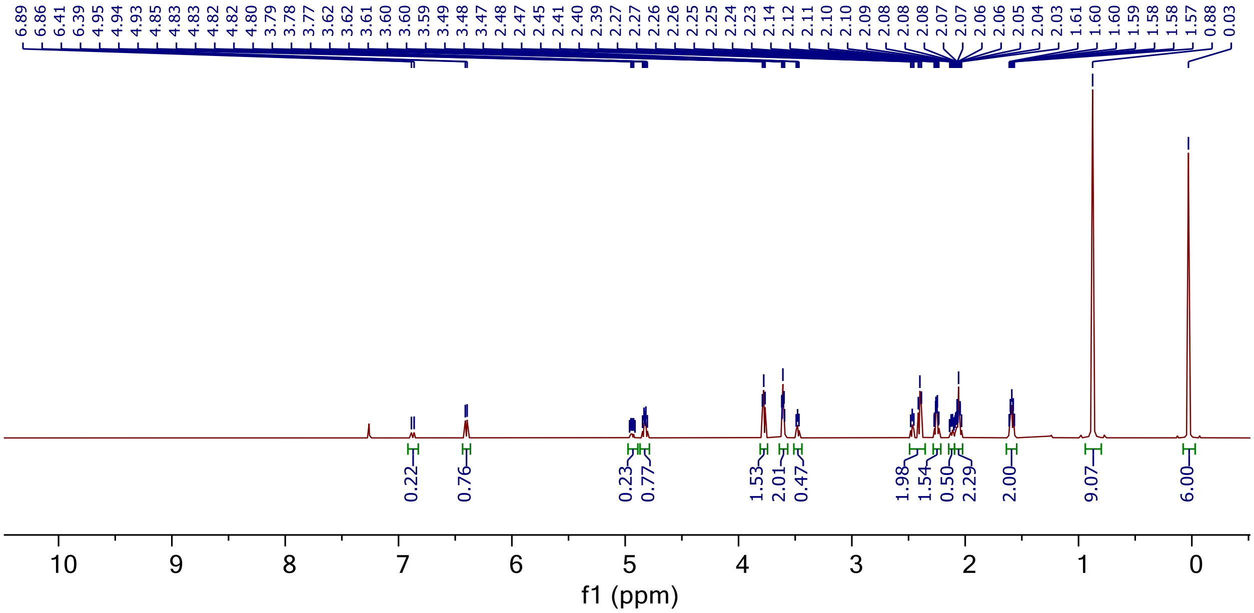

^{13}C NMR (151 MHz, $CDCl_3$):

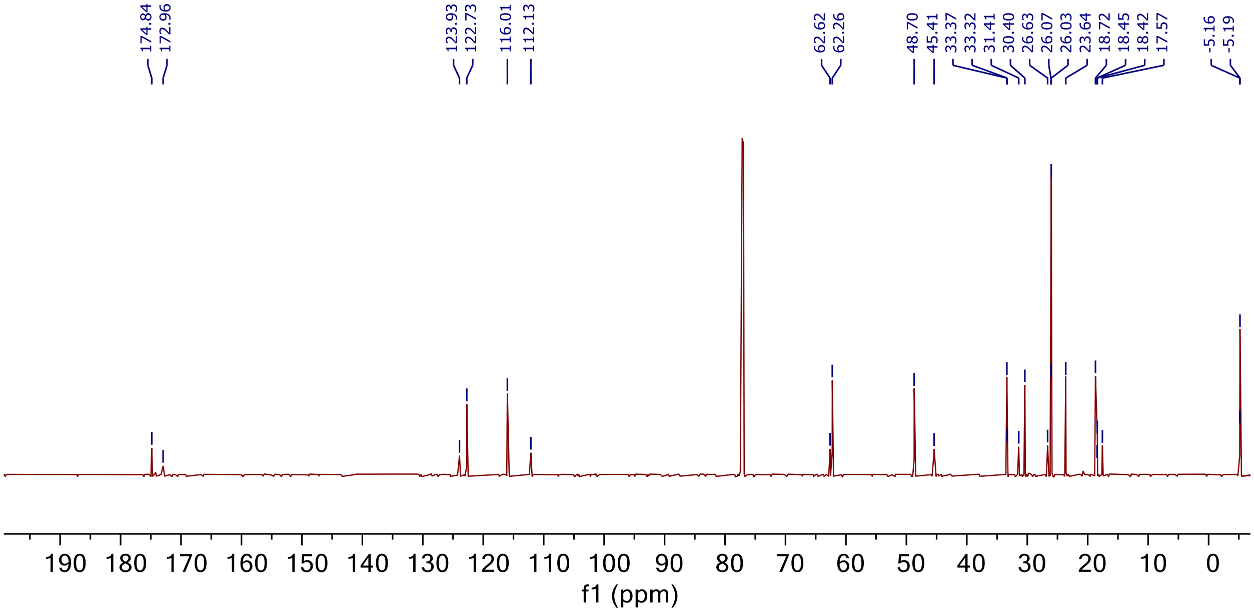

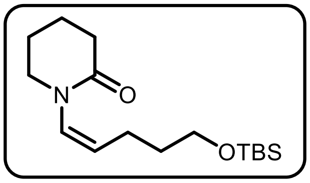

(Z)-1-(5-((tert-butyldimethylsilyl)oxy)pent-1-en-1-yl)piperidin-2-one (51c).

^{1}H NMR (600 MHz, $CDCl_3$):

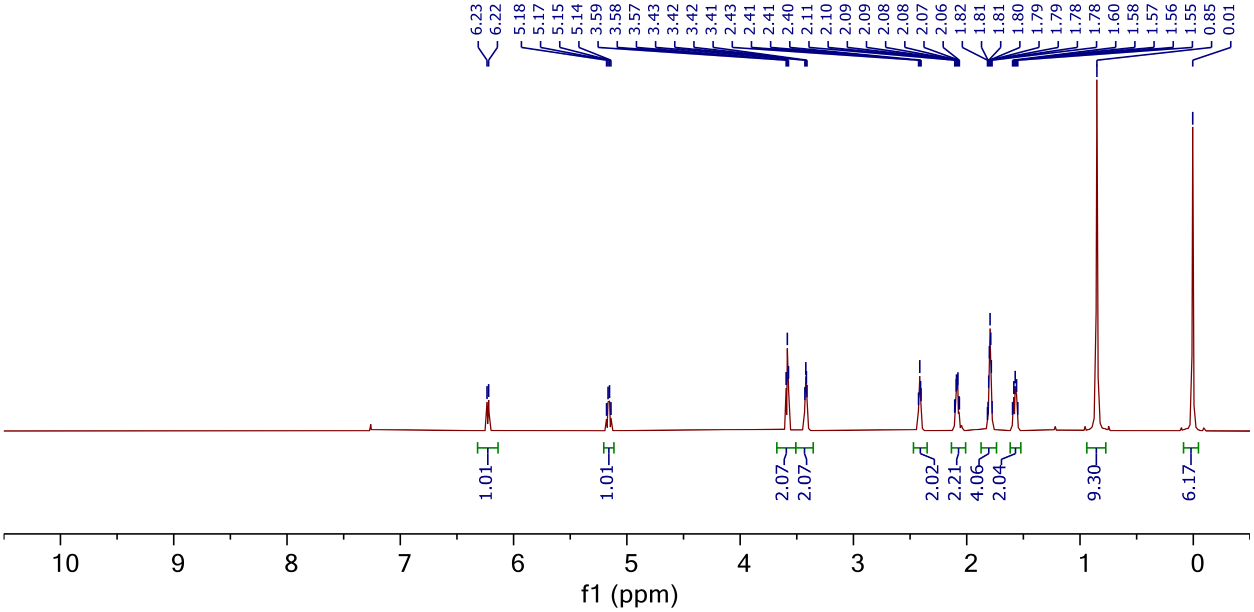

^{13}C NMR (151 MHz, $CDCl_3$):

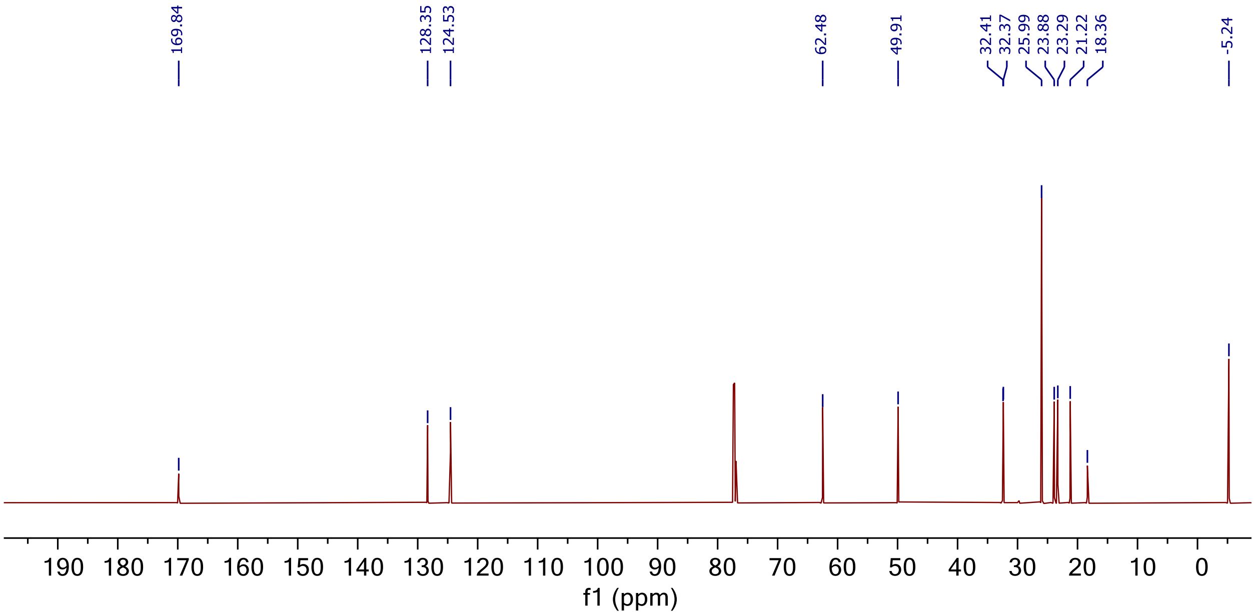

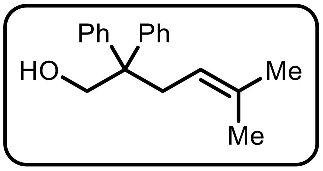

5-methyl-2,2-diphenylhex-4-en-1-ol (39a)

^{1}H NMR (600 MHz, $CDCl_3$):

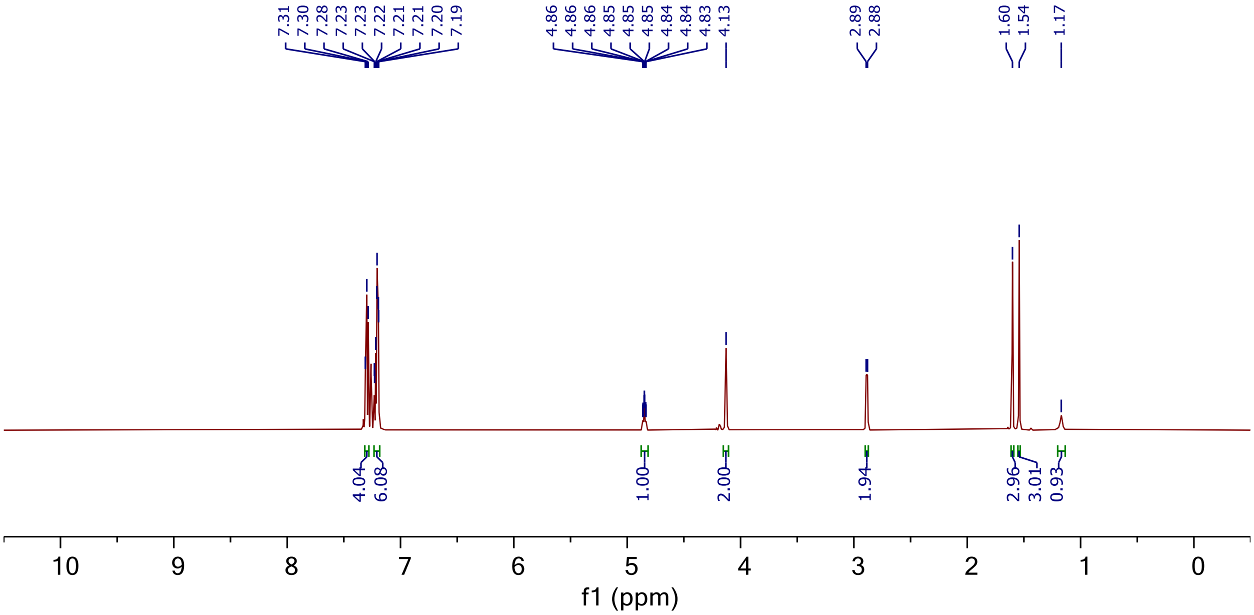

^{13}C NMR (151 MHz, $CDCl_3$):

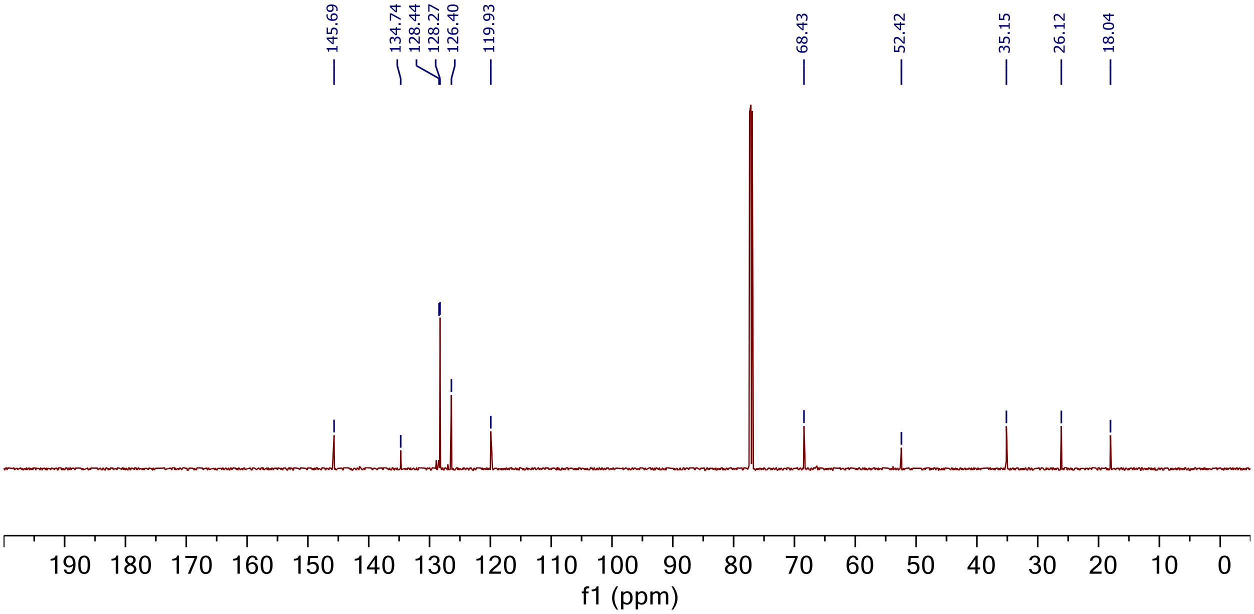

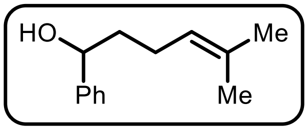

5-methyl-1-phenylhex-4-en-1-ol (40a).

^{1}H NMR (600 MHz, $CDCl_3$):

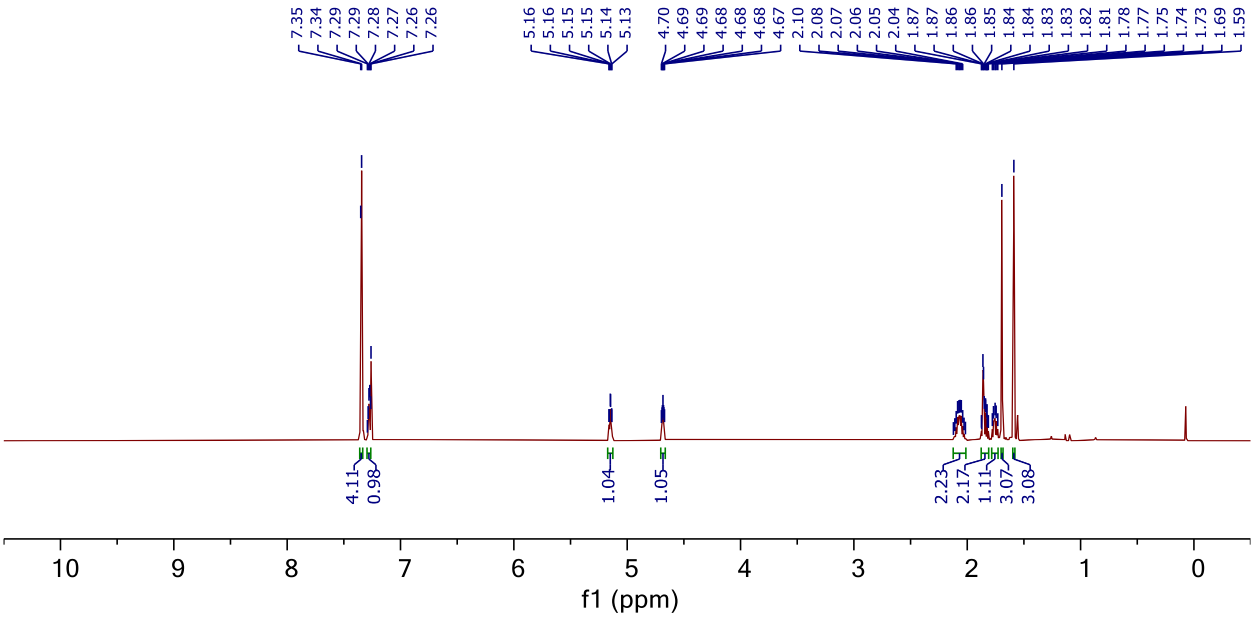

^{13}C NMR (151 MHz, $CDCl_3$):

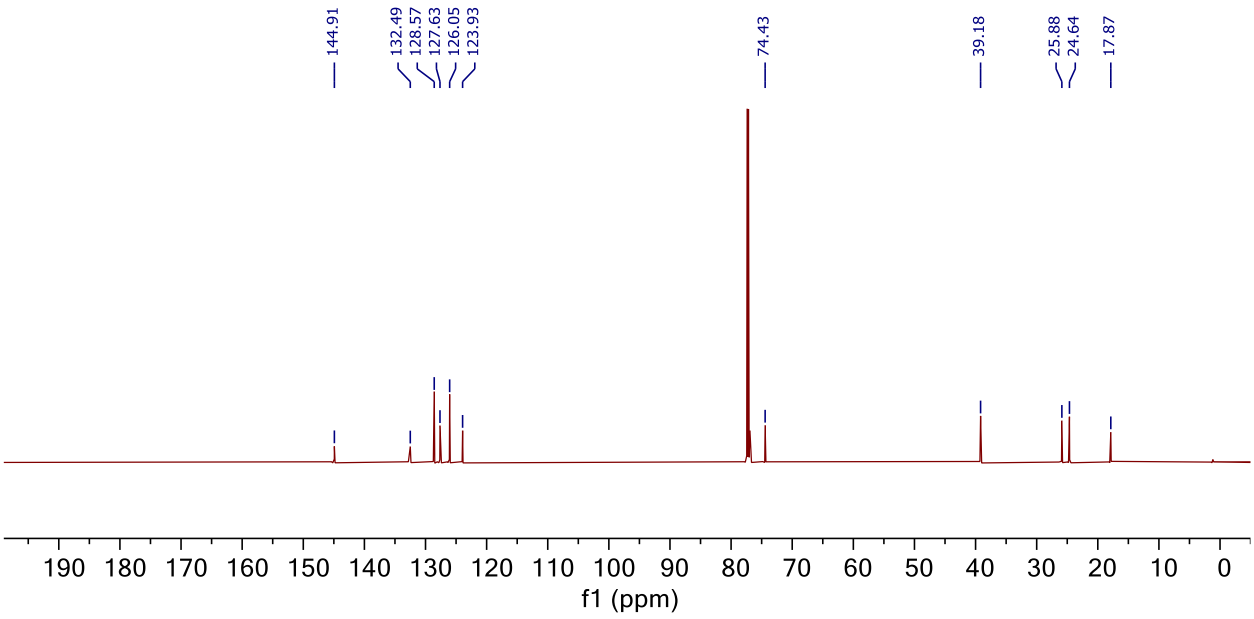

5-((triisopropylsilyl)oxy)pent-4-en-1-ol (42a)

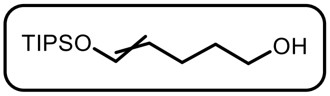

¹H NMR (600 MHz, $CDCl_3$):

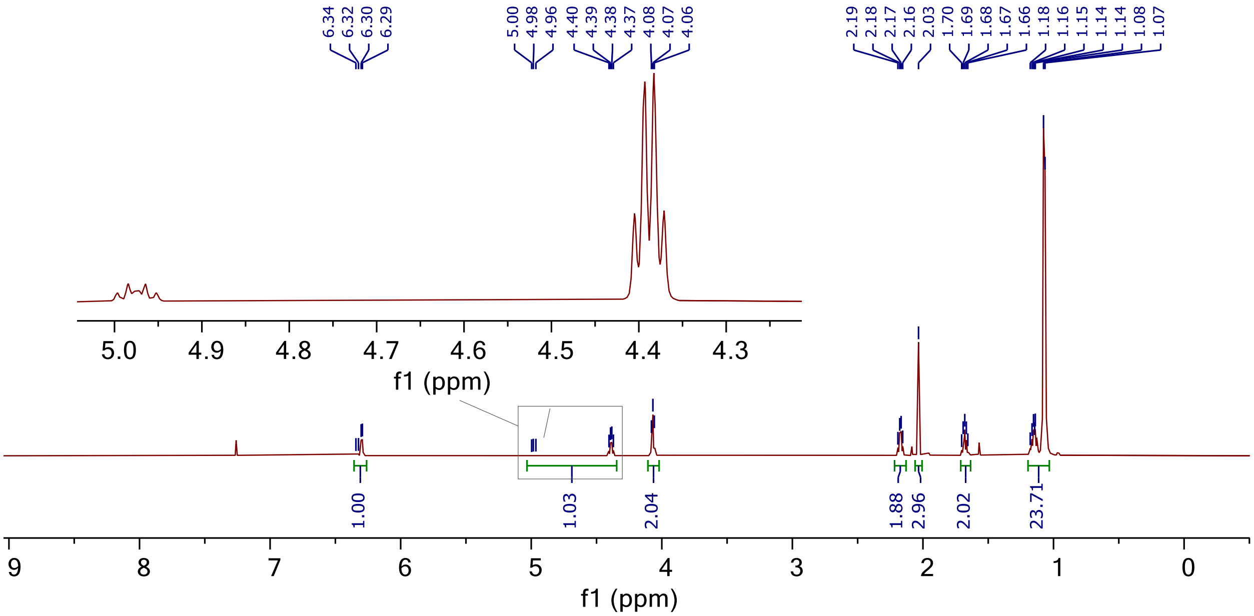

¹³C NMR (151 MHz, $CDCl_3$) :

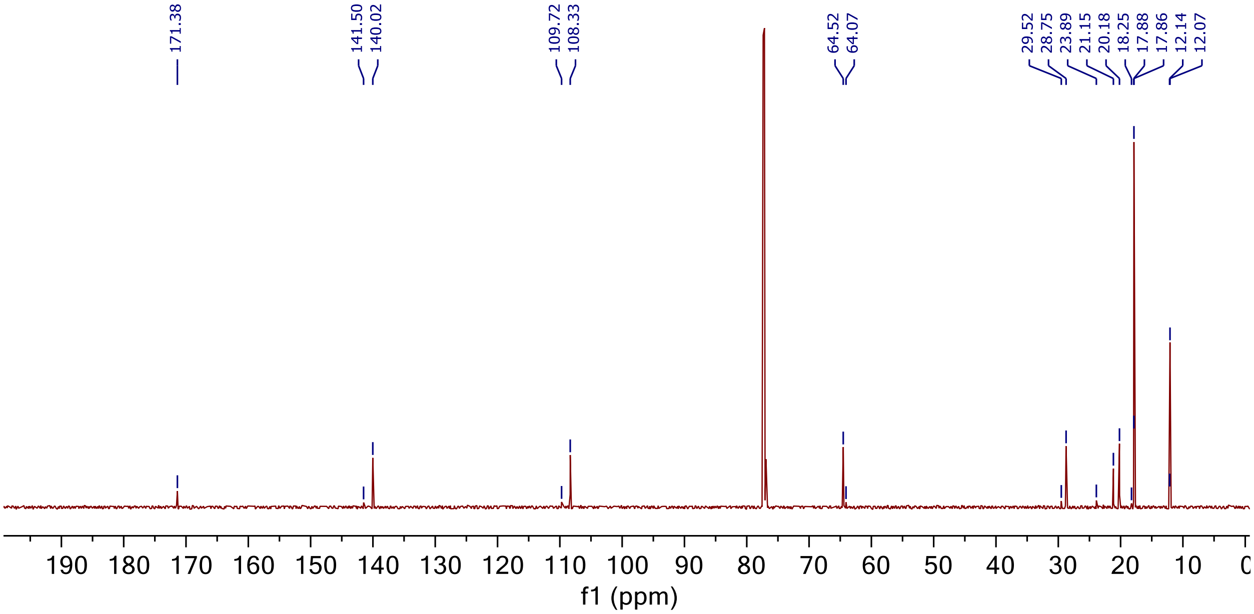

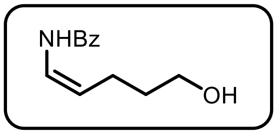

(Z)-N-(5-hydroxypent-1-en-1-yl)benzamide (43a)

^{1}H NMR (600 MHz, $CDCl_3$):

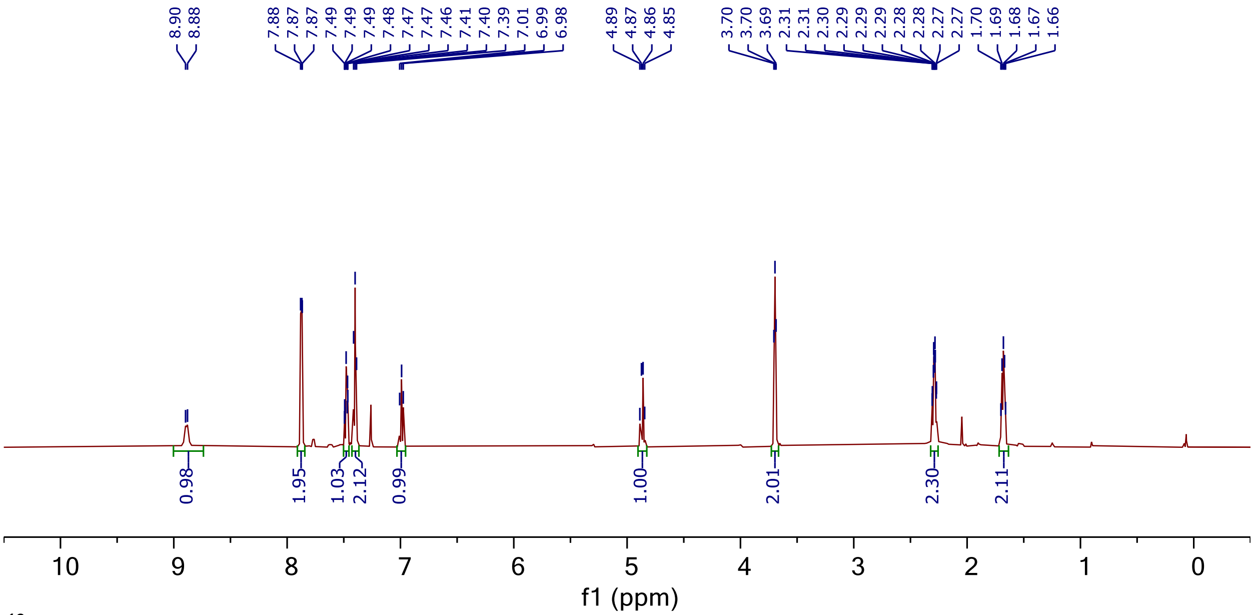

^{13}C NMR (151 MHz, $CDCl_3$):

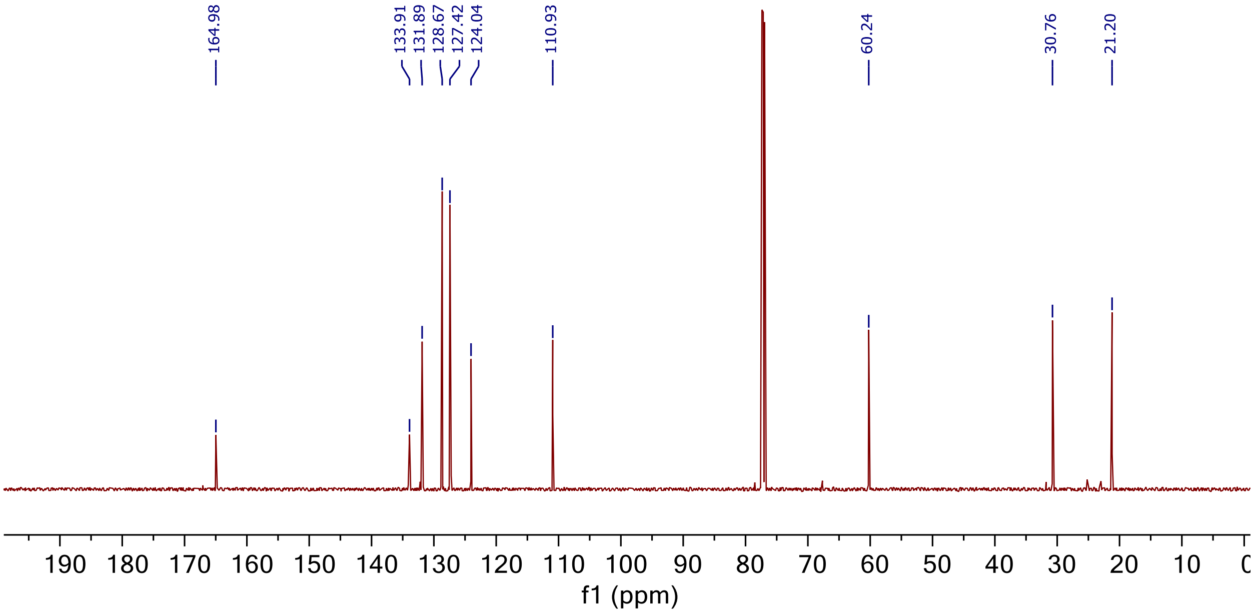

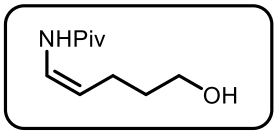

(Z)-N-(5-hydroxypent-1-en-1-yl)pivalamide (44a).

^{1}H NMR (600 MHz, $CDCl_3$):

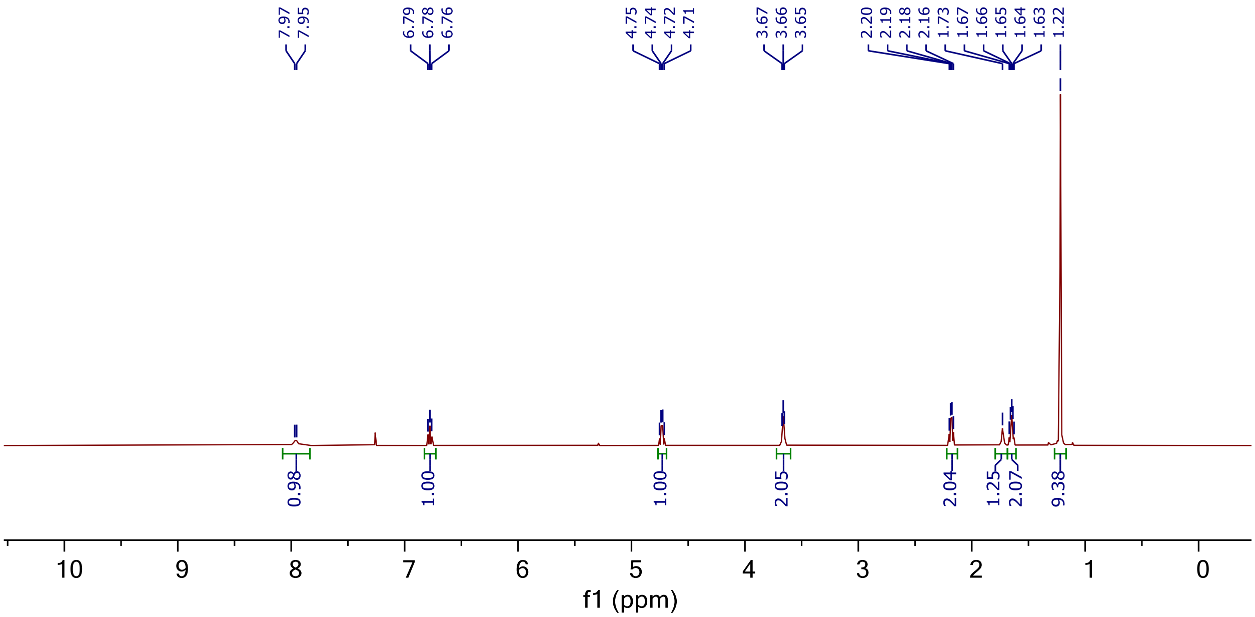

^{13}C NMR (151 MHz, $CDCl_3$):

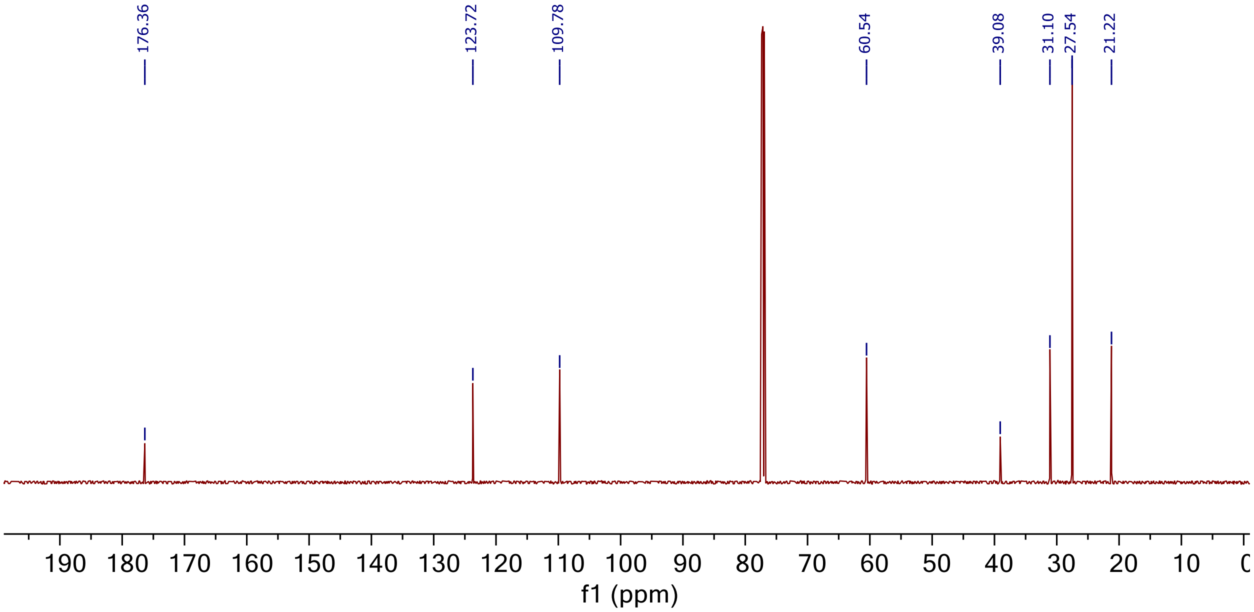

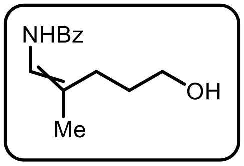

N-(5-hydroxy-2-methylpent-1-en-1-yl)benzamide (45a).

^{1}H NMR (600 MHz, $CDCl_3$):

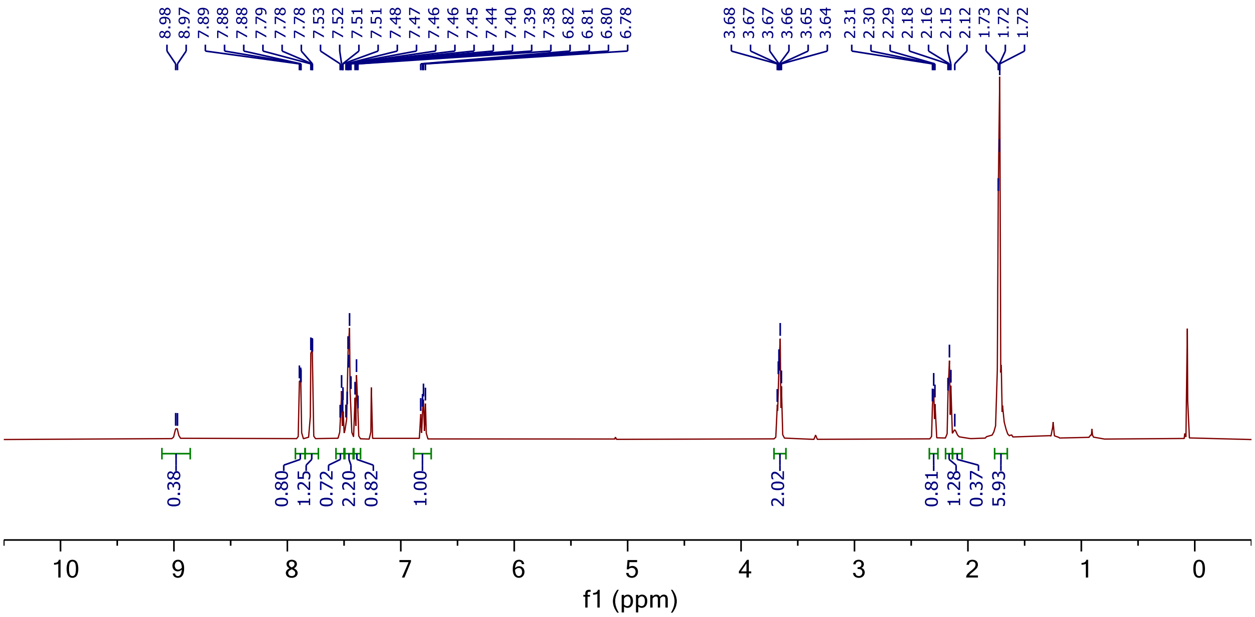

^{13}C NMR (151 MHz, $CDCl_3$):

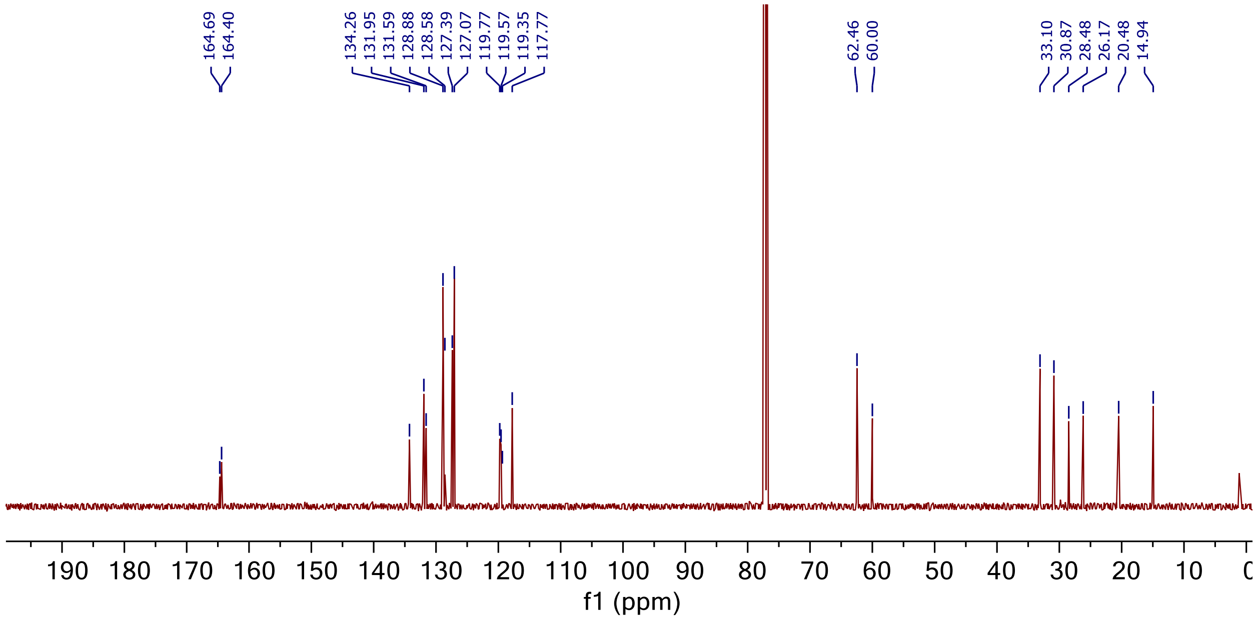

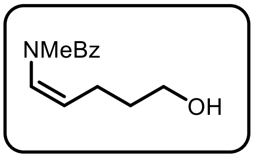

(Z)-N-(5-hydroxypent-1-en-1-yl)-N-methylbenzamide (46a).

¹H NMR (600 MHz, $CDCl_3$):

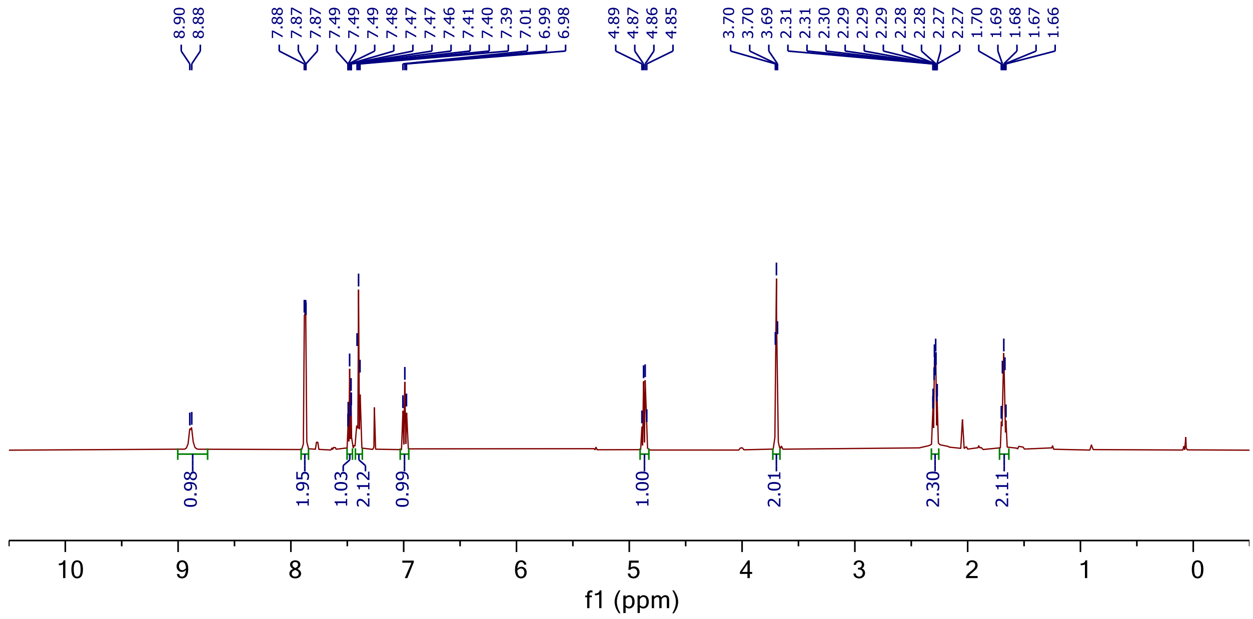

¹³C NMR (151 MHz, DMSO-d_6):

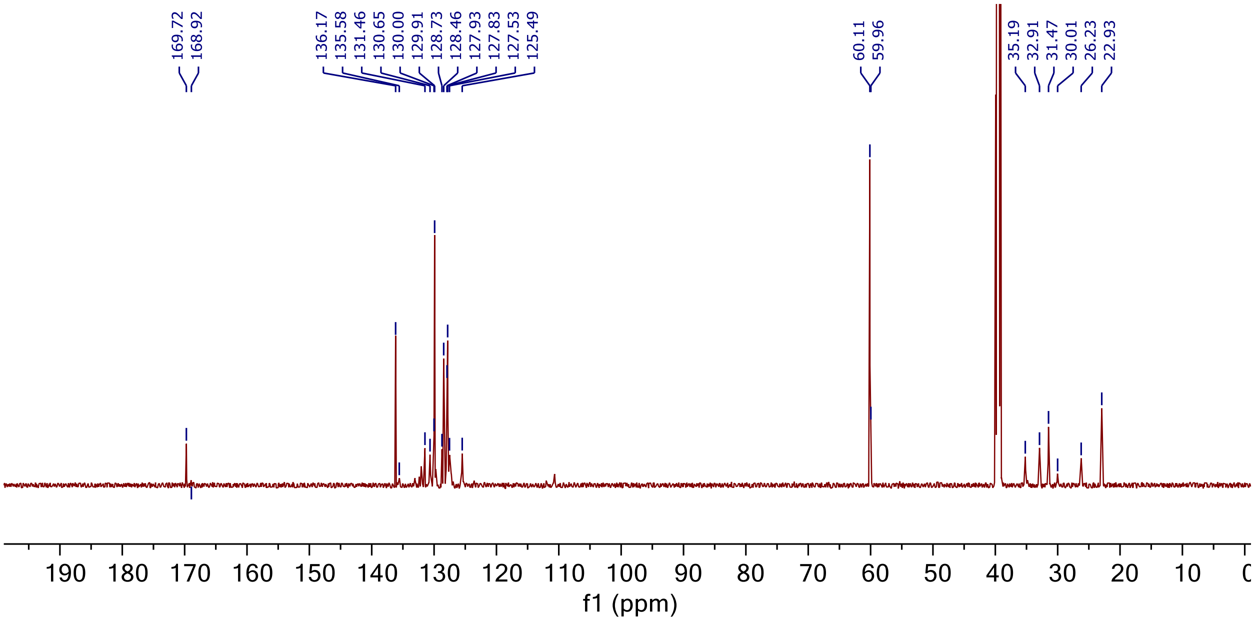

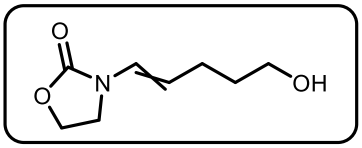

3-(5-hydroxypent-1-en-1-yl)oxazolidin-2-one (47a).

^{1}H NMR (600 MHz, $CDCl_3$):

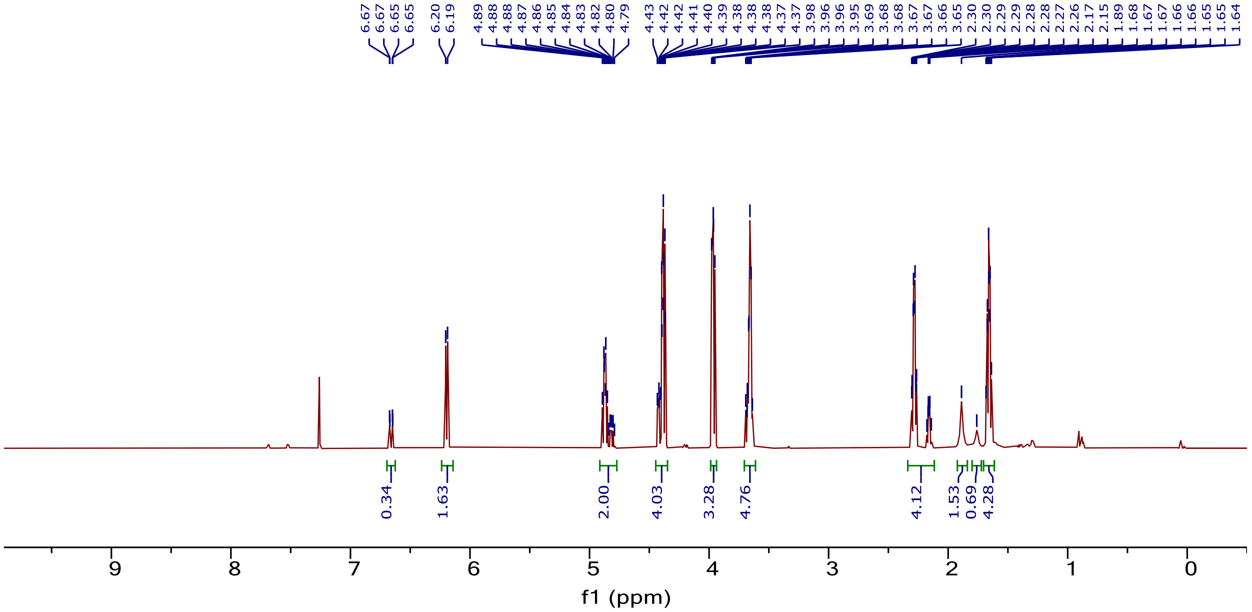

^{13}C NMR (151 MHz, $CDCl_3$):

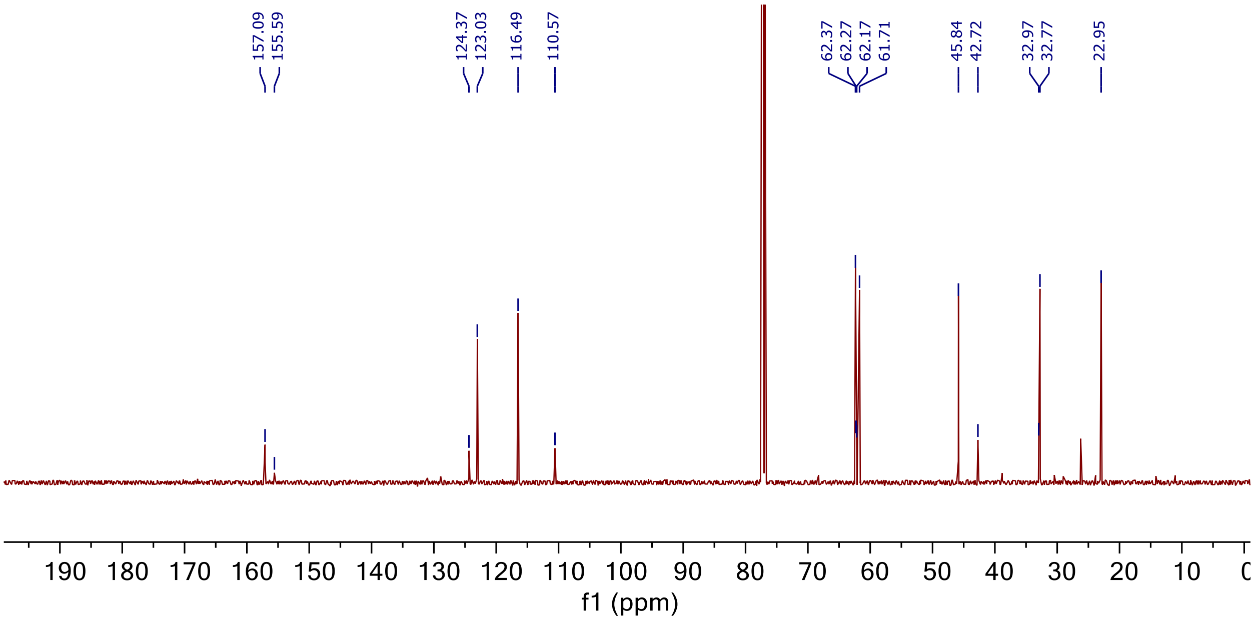

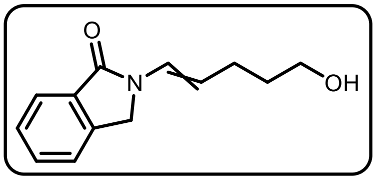

2-(5-hydroxypent-1-en-1-yl)isoindolin-1-one (48a).

^{1}H NMR (600 MHz, $CDCl_3$):

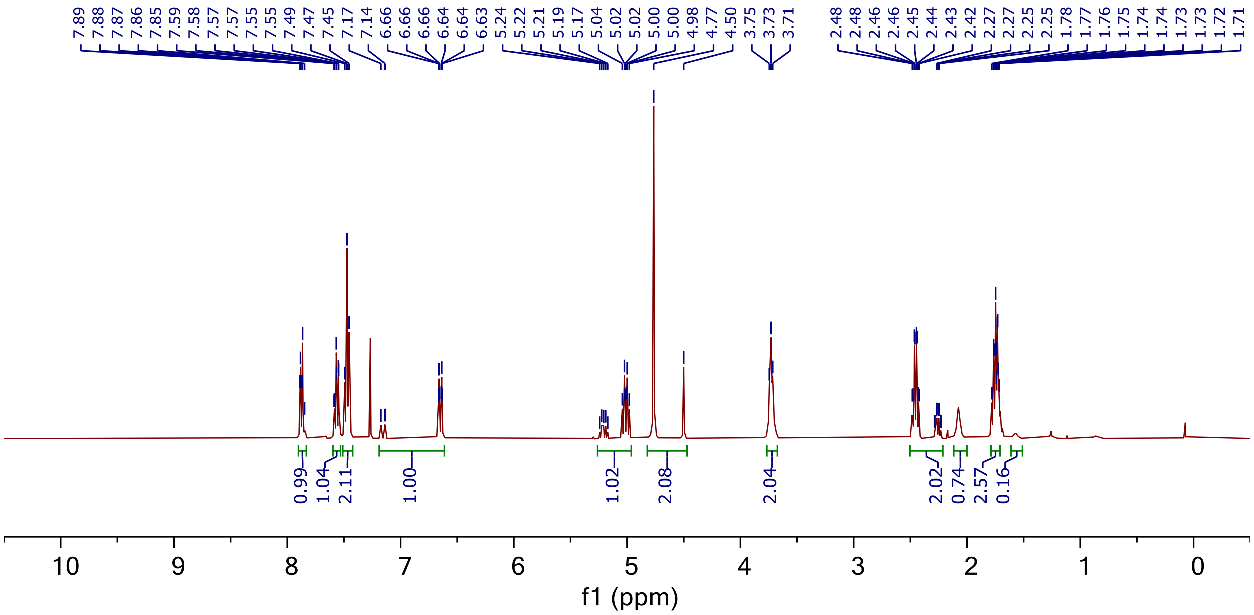

^{13}C NMR (151 MHz, $CDCl_3$) :

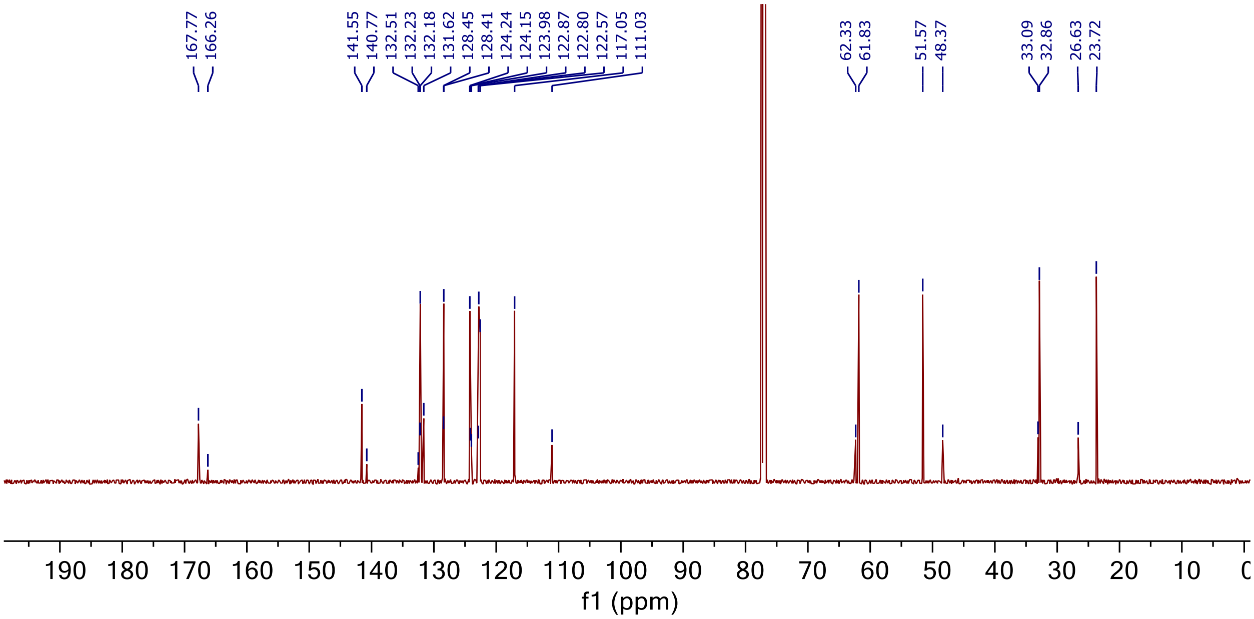

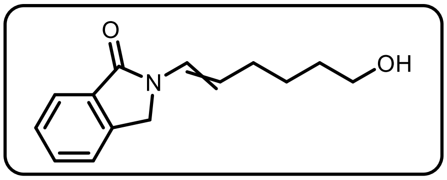

2-(6-hydroxyhex-1-en-1-yl)isoindolin-1-one (49a)

^{1}H NMR (600 MHz, $CDCl_3$):

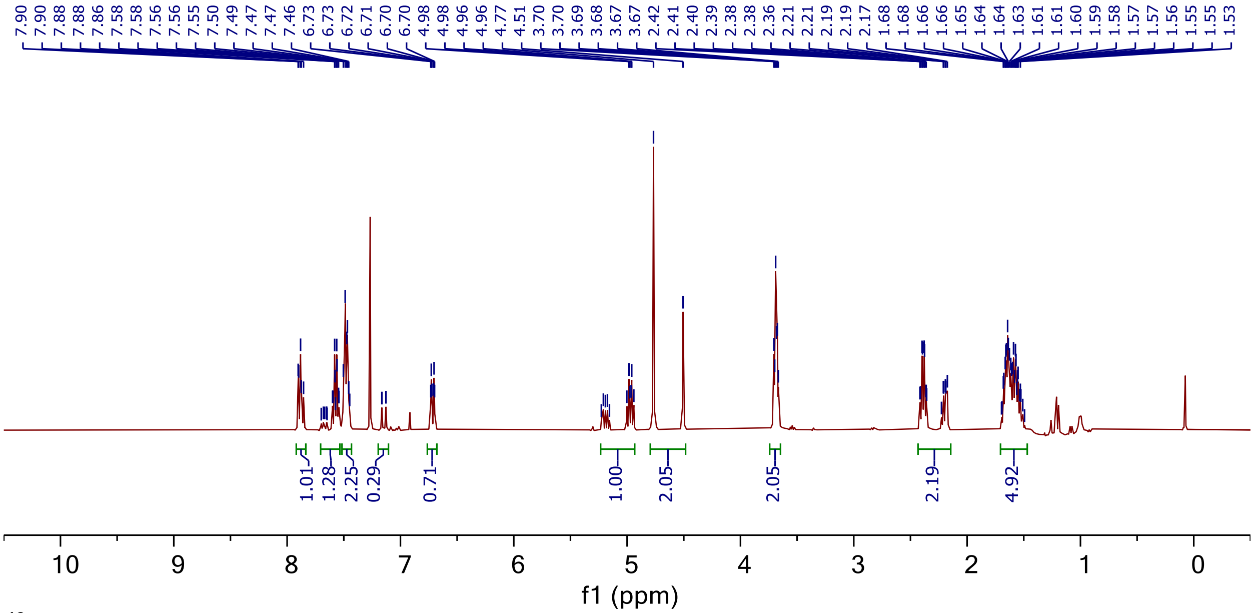

^{13}C NMR (151 MHz, $CDCl_3$):

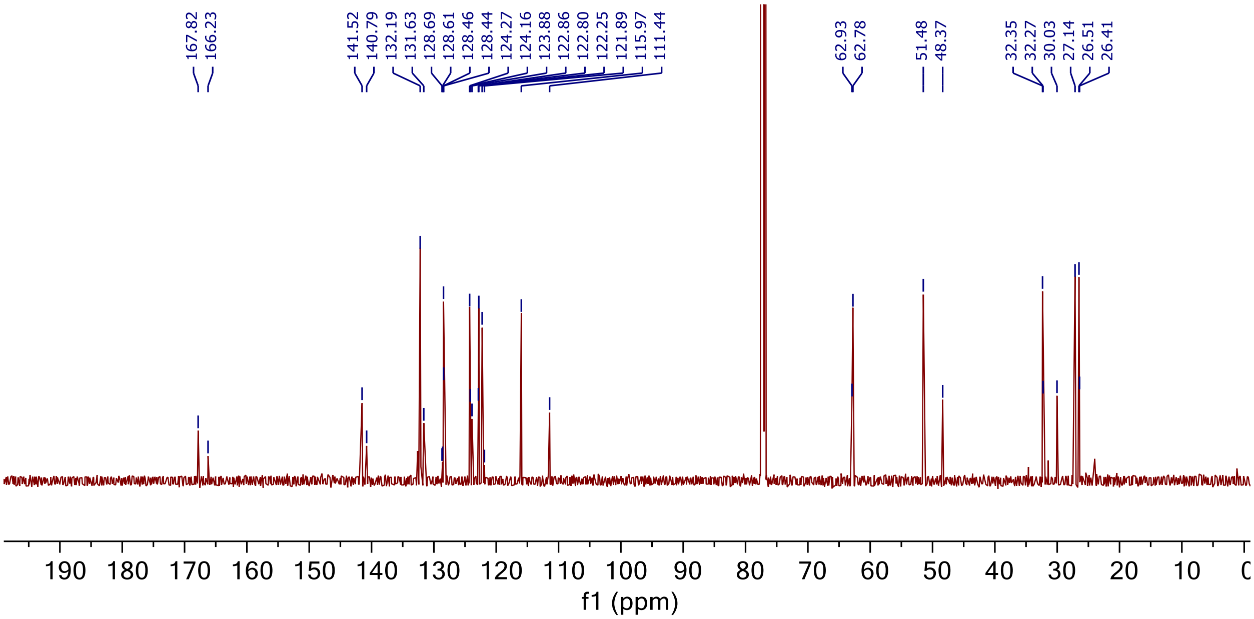

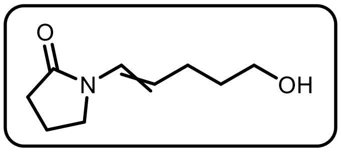

1-(5-hydroxypent-1-en-1-yl)pyrrolidin-2-one (50a).

^{1}H NMR (600 MHz, $CDCl_3$):

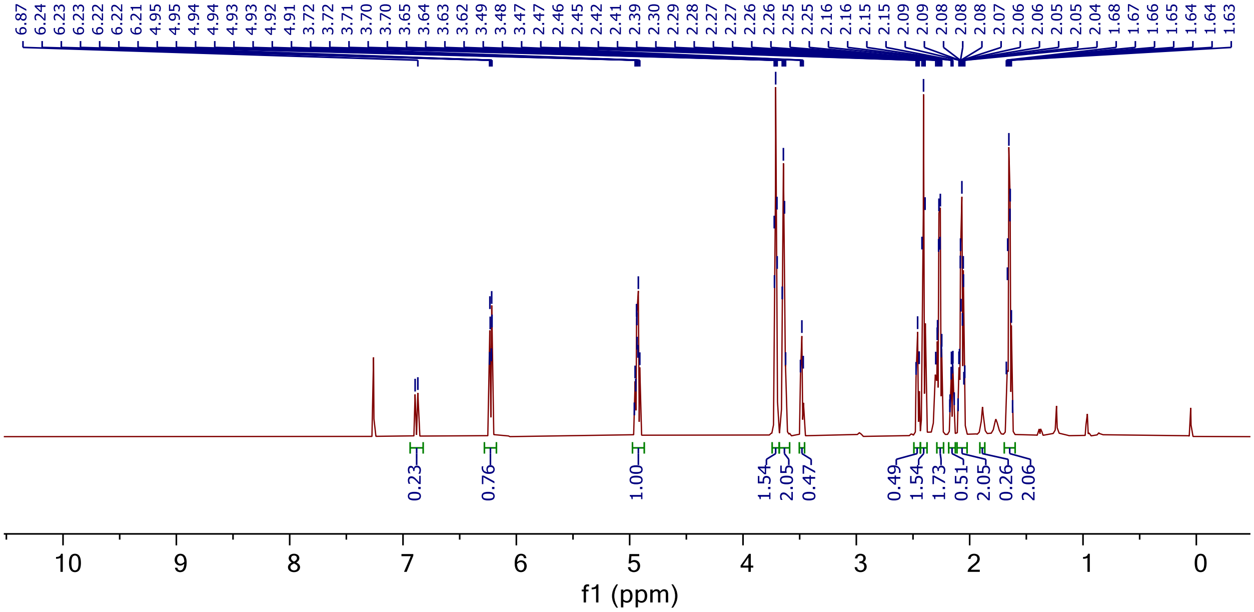

^{13}C NMR (151 MHz, $CDCl_3$):

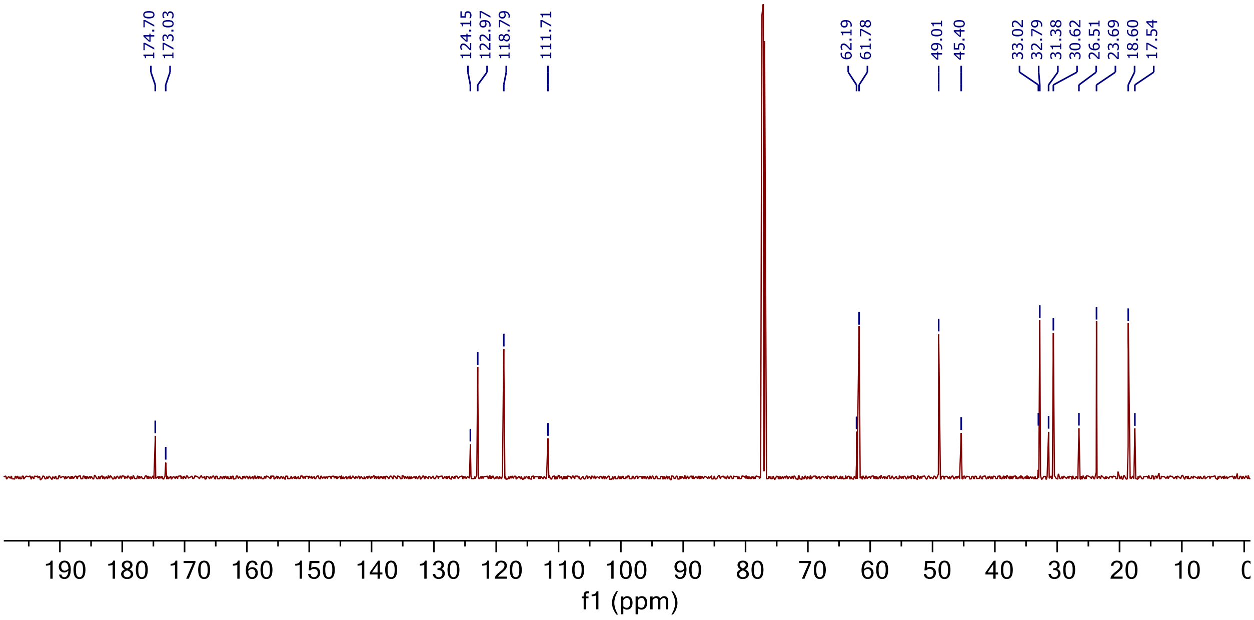

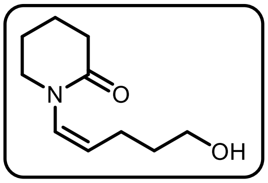

(Z)-1-(5-hydroxypent-1-en-1-yl)piperidin-2-one (51a)

^{1}H NMR (600 MHz, $CDCl_3$):

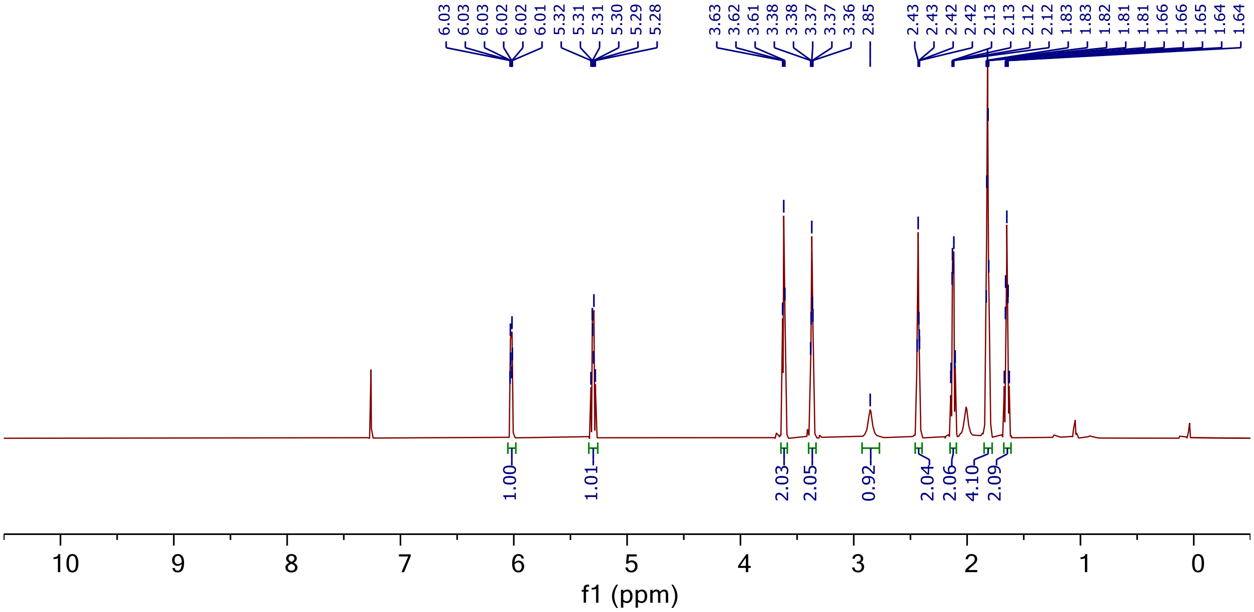

^{13}C NMR (151 MHz, $CDCl_3$):

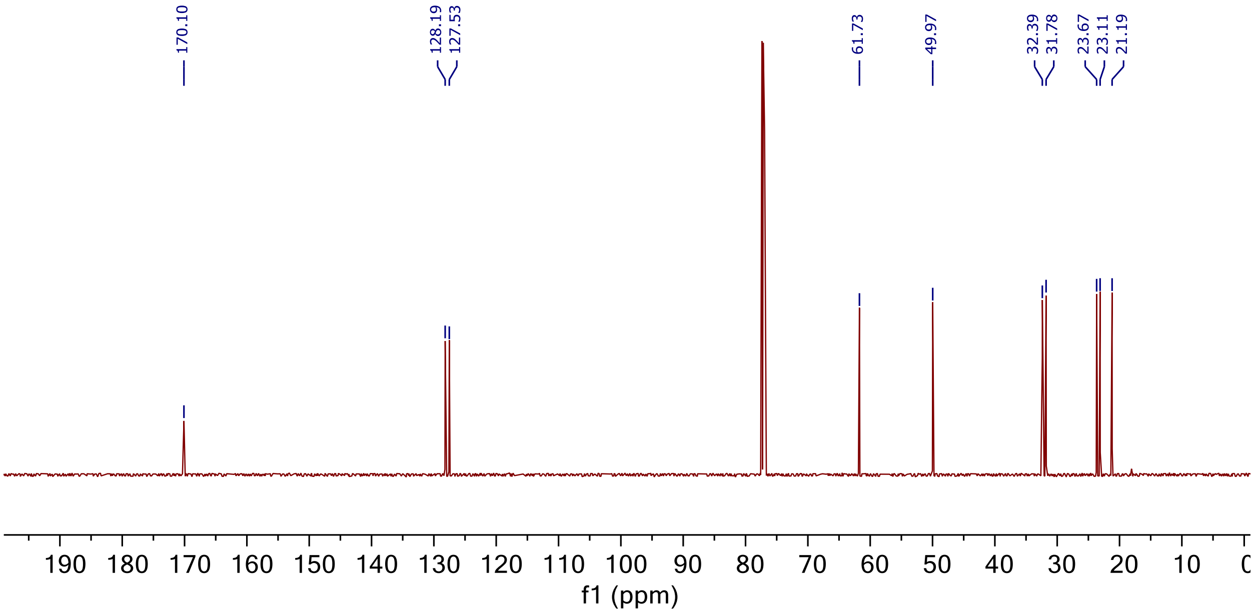

E. Synthesis and Characterization of Intermolecular Substrates

i) General Procedures

General procedure E1: Preparation of enamide substrates via cross-coupling

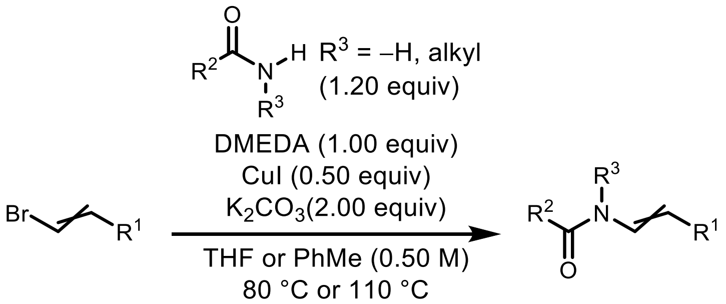

A flame-dried crimp cap vial was charged with CuI (0.5 mmol, 0.5 equiv), K_2CO_3 (2.0 mmol, 2.0 equiv), and amide (1.2 mmol, 1.2 equiv), sealed with a crimp cap and evacuated and backfilled with nitrogen three times. A separate flame-dried vial was charged with vinyl bromide (1.0 mmol) and evacuated and backfilled with N_2 three times. Anhydrous *N*,*N*-dimethylethylenediamine (DMEDA) (1.0 mmol, 1.0 equiv) was added to the vial in a single portion via syringe, followed by the addition of anhydrous THF or PhMe (2.0 mL, 0.5 M) solvent. The resulting solution was degassed for 2 minutes by sparging nitrogen through the solution during sonication. This solution was then transferred into the sealed crimp vial via syringe. The vial was further reinforced with Teflon tape along with electrical tape. The reaction was subsequently transferred to a pre-heated oil bath and stirred at 80 °C (110 °C if in PhMe). After the reaction was judged to be complete by TLC, it was allowed to cool down to room temperature and filtered through a short plug of silica gel eluting with EtOAc. The filtrate was concentrated, and the residue was purified by column chromatography on silica gel to access the desired product.

General procedure E1: Preparation of enoxysilane substrates via soft enolization

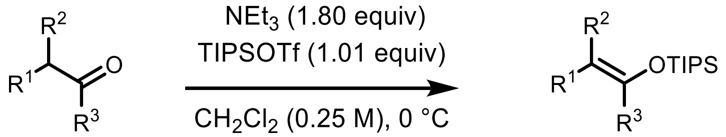

A flame-dried, 25 mL round-bottomed flask was equipped with a magnetic stir bar, sealed with a rubber septum, evacuated and backfilled with nitrogen three times and placed under a nitrogen atmosphere. Anhydrous CH_2Cl_2 (0.25 M) was transferred into the flask via syringe. The carbonyl (1.0 mmol, 1.0 equiv) was added to the flask in a single portion via syringe and the resulting solution was cooled to 0 °C by transferring the reaction apparatus to an ice-water bath. After stirring at this temperature for 5 minutes, NEt_3 (1.8 mmol, 1.8 equiv) and freshly distilled TIPSOTf (1.0 mmol, 1.01 equiv) (in this order) were added dropwise via syringes over the course of 2 minutes. After stirring at 0 °C for 30 minutes, the reaction was warmed up to room temperature by removing the reaction apparatus from the ice-water bath and concentrated under pressure by rotary evaporation to provide a crude oil. The crude mixture was purified by column chromatography on basic alumina or NEt_3 treated silica gel to access the desired product.

Synthesis of intermolecular enamide substrates:

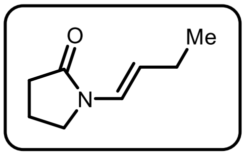

(E)-1-(prop-1-en-1-yl)pyrrolidin-2-one (1a). The reaction was set up using the general procedure **E1** with pyrrolidin-2-one (458 μL, 6.0 mmol) and (*E*)-1-bromoprop-1-ene (428 μL, 5.00 mmol) in PhMe (0.5 M, 12.0 mL) for 3 hours. Following this, the crude product was purified using silica column chromatography (hexanes/EtOAc 100/0 to hexanes/EtOAc 50/50) to yield the indicated product in 70% yield (435 mg) as a white solid. The resulting product has been previously reported and matches the obtained spectroscopic data tabulated below.[12]

^{1}H NMR (600 MHz, $CDCl_3$): δ 6.87 (d, *J* = 14.4 Hz, 1H), 4.93 (dq, *J* = 13.5, 6.6 Hz, 1H), 3.48 (t, *J* = 7.2 Hz, 2H), 2.46 (t, *J* = 8.2 Hz, 2H), 2.07 (ap, *J* = 7.7 Hz, 2H), 1.71 (d, *J* = 6.7 Hz, 3H).

^{13}C NMR (151 MHz, $CDCl_3$): δ 172.6, 124.4, 106.8, 45.2, 31.2, 17.4, 15.2.

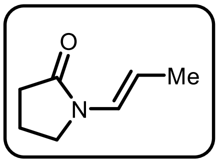

(E)-1-(but-1-en-1-yl)pyrrolidin-2-one (2a). The reaction was set up using the procedure in the reported literature.[13] Following this, the crude product was purified using silica column chromatography (hexanes/EtOAc 100/0 to hexanes/EtOAc 50/50) to yield the indicated product in 48% yield (666 mg) as a slightly yellow liquid. The resulting product has been previously reported and matches the obtained spectroscopic data tabulated below.[13]

^{1}H NMR (600 MHz, $CDCl_3$): δ 6.84 (dt, *J* = 14.4, 1.6 Hz, 1H), 4.95 (adt, *J* = 14.4, 6.7 Hz, 1H), 3.51 – 3.43 (m, 2H), 2.44 (dd, *J* = 8.7, 7.6 Hz, 2H), 2.10 – 2.02 (m, 4H), 0.99 (t, *J* = 7.4 Hz, 3H).

^{13}C NMR (151 MHz, $CDCl_3$): δ 172.9, 123.1, 114.2, 45.4, 31.4, 23.4, 17.5, 14.6.

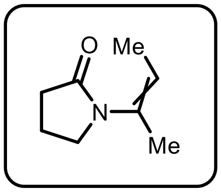

1-(but-2-en-2-yl)pyrrolidin-2-one (3a). The reaction was set up using the general procedure **E1** with pyrrolidin-2-one (458 μL, 6.0 mmol) and 2-bromobut-2-ene (508 μL, 5.0 mmol) in PhMe (0.5 M, 12.0 mL) for 3 hours. Following this, the crude product was purified using silica column chromatography (hexanes/EtOAc 100/0 to hexanes/EtOAc 50/50) to yield the indicated product in 52% yield (360 mg) as a colorless liquid (*E:Z* =1:1). The resulting product has been previously reported and matches the obtained spectroscopic data tabulated below.[12]

^{1}H NMR (600 MHz, $CDCl_3$): For *E*-isomer: δ 5.41 (aq, *J* = 6.8 Hz, 1H), 3.47 (t, *J* = 7.0 Hz, 2H), 2.45 – 2.40 (m, 2H), 2.10 (ap, *J* = 7.5 Hz, 2H), 1.81 (s, 3H), 1.51 (ad, *J* = 6.7 Hz, 3H). For *Z*-isomer: δ 5.31 (aq, *J* = 7.5 Hz, 1H), 3.53 (t, *J* = 7.0 Hz, 2H), 2.45 – 2.40 (m, 2H), 2.02 (ap, *J* = 7.5 Hz, 2H), 1.94 (s, 3H), 1.66 (ad, *J* = 6.9 Hz, 3H).

^{13}C NMR (151 MHz, $CDCl_3$): For *E*-isomer: δ 173.4, 132.1, 121.7, 47.6, 31.1, 19.3, 18.8, 13.1. For *Z*-isomer: δ 173.9, 134.0, 115.9, 49.3, 32.2, 18.3, 14.2, 12.5.

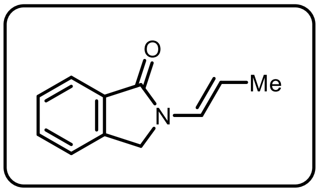

(E)-2-(prop-1-en-1-yl)isoindolin-1-one (4a). The reaction was set up using the general procedure **E1** with isoindolin-1-one (399 mg, 3.0 mmol) and 1-bromoprop-1-ene (213 μL, 2.5 mmol) in PhMe (0.5 M, 6.0 mL) for 5 hours. Following this, the crude product was purified using silica column chromatography (hexanes/EtOAc 100/0 to hexanes/EtOAc 50/50) to yield the indicated product in 35% yield (153 mg) as a white solid.

IR (Diamond-ATR, neat) ṽ (cm^{-1}): 3041, 2853, 2916, 1683, 1464, 1397, 1373.

¹H NMR (600 MHz, $CDCl_3$): δ 7.86 (ad, *J* = 8.0 Hz, 1H), 7.55 (at, *J* = 7.5 Hz, 1H), 7.48 – 7.44 (m, 2H), 7.13 (d, *J* = 14.3 Hz, 1H), 5.20 (dq, *J* = 13.4, 6.7 Hz, 1H), 4.49 (s, 2H), 1.81 (d, *J* = 6.7 Hz, 3H).
¹³C NMR (151 MHz, $CDCl_3$): δ 165.9, 140.6, 132.5, 131.9, 128.3, 124.2, 124.0, 122.7, 106.2, 48.2, 15.3.
HRMS (ESI): m/z: $[M+H]^+$ calc'd for $C_{11}H_{12}NO^+$: 174.0919. Found: 174.0910.

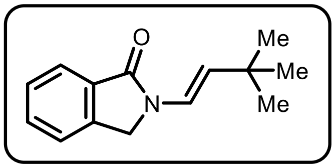

(E)-2-(3,3-dimethylbut-1-en-1-yl)isoindolin-1-one (5a). The reaction was set up using a slight modification of the general procedure **E1**. *(E)*-1-iodo-3,3-dimethylbut-1-ene[14] (210 mg, 1.00 mmol) and isoindolin-1-one (160 mg, 1.20 mmol) were stirred in PhMe (0.5 M, 2 mL) at 110 °C for 16 hours. Following this, the crude mixture was purified using silica gel column chromatography (hexanes/EtOAc 100/0 to hexanes/EtOAc 75/25) to yield the indicated product in 81% yield (175 mg) as off-white solid.
IR (Diamond-ATR, neat) ṽ (cm^{-1}): 2947, 2863, 1694, 1664, 1462, 1144.
¹H NMR (600 MHz, $CDCl_3$): δ 7.86 (d, *J* = 7.5 Hz, 1H), 7.55 (at, *J* = 7.4 Hz, 1H), 7.45 (d, *J* = 7.0 Hz, 2H), 7.09 (d, *J* = 14.9 Hz, 1H), 5.25 (d, *J* = 14.7 Hz, 1H), 4.49 (s, 2H), 1.12 (s, 9H).
¹³C NMR (151 MHz, $CDCl_3$): δ 166.4, 140.8, 132.8, 132.1, 128.3, 124.1, 123.5, 122.8, 120.3, 48.4, 32.3, 30.4.
HRMS (ESI): m/z: $[M+H]^+$ calc'd for $C_{14}H_{18}NO^+$: 216.1388. Found: 216.1387.

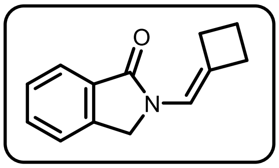

2-(cyclobutylidenemethyl)isoindolin-1-one (6a). The reaction was set up using a slight modification of the general procedure **E1**. (bromomethylene)cyclobutane[15] (55.5 mg, 0.377 mmol) and isoindolin-1-one (60.3 mg, 0.453 mmol) was stirred in PhMe (0.5 M, 0.76 mL) at 110 °C for 16 hours. Following this, the crude mixture was purified using silica column chromatography (hexanes/EtOAc 100/0 to hexanes/EtOAc 75/25) to yield the indicated product in 52% yield (39.3 mg) as off-white solid.
IR (Diamond-ATR, neat) ṽ (cm^{-1}): 2938, 2251, 1669, 1470, 1152.
¹H NMR (600 MHz, $CDCl_3$): δ 7.86 (d, *J* = 7.6 Hz, 1H), 7.54 (atd, *J* = 7.5, 1.3 Hz, 1H), 7.50 – 7.42 (m, 2H), 6.73 (at, *J* = 2.4 Hz, 1H), 4.63 (s, 2H), 3.10 – 3.02 (m, 2H), 2.86 – 2.80 (m, 2H), 2.10 (p, *J* = 7.9 Hz, 2H).
¹³C NMR (151 MHz, $CDCl_3$): δ 166.3, 141.3, 132.1, 131.8, 128.3, 124.3, 124.0, 122.7, 115.5, 49.5, 30.1, 29.6, 18.0.
HRMS (ESI): m/z: $[M+H]^+$ calc'd for $C_{13}H_{14}NO^+$: 200.1075. Found: 200.1064.

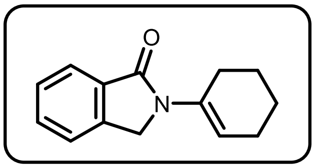

2-(cyclohex-1-en-1-yl)isoindolin-1-one (7a). The reaction was set up using a slight modification of the general procedure **E1**. 1-bromocyclohex-1-ene[16] (242 mg, 1.50 mmol) and isoindolin-1-one (240 mg, 1.80 mmol) was stirred in PhMe (0.5 M, 3.0 mL) at 110 °C for 16 hours. Following this, the crude mixture was purified using silica column chromatography (hexanes/EtOAc 100/0 to hexanes/EtOAc 75/25) to yield the indicated product in 90% yield (289 mg) as off-white solid.
IR (Diamond-ATR, neat) ṽ (cm^{-1}): 2925, 2851, 1673, 1654, 1239.
¹H NMR (600 MHz, $CDCl_3$): δ 7.82 (dd, *J* = 7.4, 1.4 Hz, 1H), 7.52 (atd, *J* = 7.4, 1.3 Hz, 1H), 7.49 – 7.39 (m, 2H), 5.78 (td, *J* = 4.1, 1.9 Hz, 1H), 4.55 (s, 2H), 2.60 (tdd, *J* = 6.4, 3.3, 1.8 Hz, 2H), 2.20 (tdt, *J* = 6.5, 5.0, 2.5 Hz, 2H), 1.81 – 1.73 (m, 2H), 1.64 (apd, *J* = 5.7, 2.9 Hz, 2H).

13C NMR (151 MHz, $CDCl_3$): δ 167.2, 140.5, 136.2, 133.7, 131.6, 128.2, 123.9, 122.5, 115.6, 50.9, 26.9, 24.6, 22.9, 22.0.
HRMS (ESI): m/z: $[M+H]^+$ calc'd for $C_{14}H_{16}NO^+$: 214.1231. Found: 214.1228.

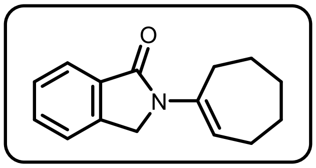

2-(cyclohept-1-en-1-yl)isoindolin-1-one (8a). The reaction was set up using a slight modification of the general procedure **E1**. 1-bromocyclohept-1-ene[16] (263 mg, 1.50 mmol) and isoindolin-1-one (240 mg, 1.80 mmol) was stirred in PhMe (0.5 M, 3.0 mL) at 110 °C for 16 hours. Following this, the crude mixture was purified using silica column chromatography (hexanes/EtOAc 100/0 to hexanes/EtOAc 75/25) to yield the indicated product in 74% yield (252 mg) as off-white solid.
IR (Diamond-ATR, neat) ṽ (cm^{-1}): 2942, 2292, 2252, 1691, 1653, 1039.
1H NMR (600 MHz, $CDCl_3$): δ 7.82 (d, *J* = 7.6 Hz, 1H), 7.52 (t, *J* = 7.5 Hz, 1H), 7.47 – 7.39 (m, 2H), 5.85 (t, *J* = 6.8 Hz, 1H), 4.55 (s, 2H), 2.66 (dd, *J* = 8.2, 3.8 Hz, 2H), 2.24 (aq, *J* = 6.2 Hz, 2H), 1.80 (t, *J* = 6.1 Hz, 2H), 1.71 (t, *J* = 5.6 Hz, 2H), 1.58 (d, *J* = 7.0 Hz, 2H).
13C NMR (151 MHz, $CDCl_3$): δ 167.2, 142.6, 140.8, 133.5, 131.5, 128.2, 124.1, 123.9, 122.6, 52.0, 32.2, 31.5, 27.0, 27.0, 26.2.
HRMS (ESI): m/z: $[M+H]^+$ calc'd for $C_{15}H_{18}NO^+$: 228.1388. Found: 228.1380.

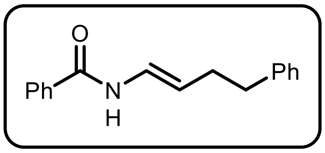

(E)-N-(4-phenylbut-1-en-1-yl)benzamide (9a). The reaction was set up using the general procedure **E1** with *(E)*-(4-bromobut-3-en-1-yl)benzene[17] (211 mg, 1.0 mmol) and benzamide (145 mg, 1.2 mmol) in THF (0.5 M, 2.0 mL) for 16 hours. Following this, the crude product was purified using silica column chromatography (hexanes/EtOAc 100/0 to hexanes/EtOAc 83/17) to yield the indicated product in 78% yield (197 mg) as a white solid. The resulting product has been previously reported and matches the obtained spectroscopic data tabulated below.[18]
1H NMR (600 MHz, $CDCl_3$): δ 7.79 (d, *J* = 7.8 Hz, 2H), 7.72 (d, *J* = 10.2 Hz, 1H), 7.52 (at, *J* = 7.4 Hz, 1H), 7.44 (at, *J* = 7.t Hz, 2H), 7.29 (at, *J* = 7.5 Hz, 2H), 7.22 – 7.17 (m, 3H), 7.02 (dd, *J* = 10.9, 3.0 Hz, 1H), 5.33 (dt, *J* = 14.3, 7.1 Hz, 1H), 2.73 (t, *J* = 7.6 Hz, 2H), 2.43 (q, *J* = 7.6 Hz, 2H).
13C NMR (151 MHz, $CDCl_3$): δ 164.4, 141.6, 133.9, 132.0, 128.8, 128.6, 128.5, 127.1, 126.1, 123.5, 113.2, 36.5, 31.7.

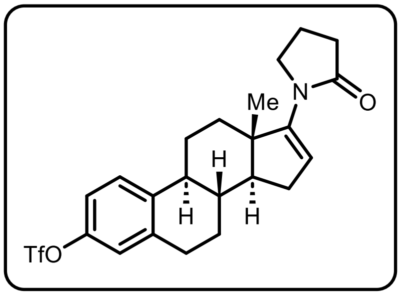

(8R,9S,13S,14S)-13-methyl-17-(2-oxopyrrolidin-1-yl)-7,8,9,11,12,13,14,15-octahydro-6H-cyclopenta[a]phenanthren-3-yl trifluoromethanesulfonate (10a). The reaction was set up using a slight modification of the general procedure **E1**. (8*R*,9*S*,13*S*,14*S*)-17-bromo-13-methyl-7,8,9,11,12,13,14,15-octahydro-6H-cyclopenta[a]phenanthren-3-yl trifluoromethanesulfonate[19] (579 mg, 1.24 mmol) and pyrrolidinone (127 mg, 1.49 mmol) was stirred in PhMe (0.5 M, 2.5 mL) at 110 °C for 16 hours. Following this, the crude mixture was purified using silica column chromatography (hexanes/EtOAc 100/0 to hexanes/EtOAc 75/25) to yield the indicated product in 60% yield (350 mg) as slightly sticky yellow solid.
IR (Diamond-ATR, neat) ṽ (cm^{-1}): 2932, 2260, 2343, 1695, 1417, 1206, 1140, 917, 734.
1H NMR (600 MHz, $CDCl_3$): δ 7.31 (d, *J* = 8.7 Hz, 1H), 7.01 (dd, *J* = 8.6, 2.7 Hz, 1H), 6.97 (d, *J* = 2.7 Hz, 1H), 5.62 (s, 1H), 3.64 (adtd, *J* = 16.5, 9.5, 6.8 Hz, 2H), 2.96 – 2.88 (m, 2H), 2.46 (at, *J* =

8.1 Hz, 2H), 2.36 – 2.25 (m, 3H), 2.22 (adt, *J* = 12.4, 2.8 Hz, 1H), 2.07 (atd, *J* = 13.4, 5.8 Hz, 3H), 1.99 – 1.92 (m, 1H), 1.74 (adtd, *J* = 23.5, 12.0, 5.1 Hz, 2H), 1.67 – 1.56 (m, 2H), 1.45 (aqd, *J* = 11.9, 8.4 Hz, 1H), 1.05 (s, 3H).

^{13}C NMR (151 MHz, $CDCl_3$): δ 174.1, 150.3, 147.7, 141.2, 139.7, 127.1, 121.4, 119.0 ($^1J_{C-F}$ = 257.7 Hz), 118.3, 117.5, 55.9, 50.0, 47.3, 44.4, 36.8, 35.4, 32.1, 29.8, 29.6, 27.0, 26.4, 18.9, 16.1.

^{19}F NMR (471 MHz, $CDCl_3$) δ -74.0.

HRMS (ESI): m/z: $[M+H]^+$ calc'd for $C_{23}H_{27}F_3NO_4S^+$: 470.1613. Found: 470.1623.

Synthesis of intermolecular silyl enol ether substrates:

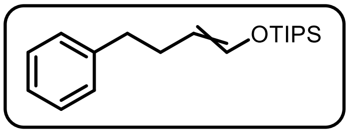

triisopropyl((4-phenylbut-1-en-1-yl)oxy)silane (11a). The reaction was set up using general procedure **E2** with 4-phenylbutanal (103 µL, 0.68 mmol) for 30 minutes. Following this, the crude product was purified using alumina column chromatography (hexanes 100%) to yield the indicated product in 88% yield (180 mg) as a colorless liquid. The resulting product has been previously reported and matches the obtained spectroscopic data tabulated below (*E:Z* =20:80).[11]

^{1}H NMR (600 MHz, $CDCl_3$): For *Z*-isomer: 7.29 – 7.24 (m, 2H), 7.22 – 7.14 (m, 3H), 6.33 (dt, *J* = 5.6 Hz, *J* = 1.1 Hz, 1H), 4.45 (td, *J* = 7.2 Hz, 5.6 Hz, 1H), 2.67 – 2.62 (m, 2H), 2.42 – 2.35 (m, 2H), 1.22 – 1.11 (m, 3H), 1.08 – 1.04 (m, 18H).

^{13}C NMR (151 MHz, $CDCl_3$): 143.5, 140.5, 129.3, 129.2, 126.6, 109.7, 36.4, 26.4, 18.1, 12.7.

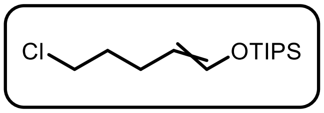

((5-chloropent-1-en-1-yl)oxy)triisopropylsilane (12a). The reaction was set up using general procedure **E2** with 5-chloropentanal (99.4 µL, 0.83 mmol) for 30 minutes. Following this, the crude product was purified using alumina column chromatography (hexanes 100%) to yield the indicated product in 91% yield (210 mg) as a colorless liquid (*E:Z*=8:92).

IR (Diamond-ATR, neat) ṽ (cm^{-1}): 2865, 1653, 1271, 743.

^{1}H NMR (600 MHz, $CDCl_3$): For *Z*-isomer: δ 6.32 (dd, *J* = 5.8, 1.3 Hz, 1H), 4.37 (td, *J* = 7.3, 5.8 Hz, 1H), 3.54 (t, *J* = 6.9 Hz, 2H), 2.24 (aqd, *J* = 7.2, 1.0 Hz, 2H), 1.83 (ap, *J* = 7.1 Hz, 2H), 1.18 – 1.12 (m, 3H), 1.07 (d, *J* = 7.3 Hz, 18H). For *E*-isomer: δ 6.36 (dt, *J* = 11.8, 1.3 Hz, 1H), 4.94 (dt, *J* = 11.7, 7.6 Hz, 1H), 3.54 (t, *J* = 6.9 Hz, 2H), 2.05 (aqd, *J* = 7.3, 1.00 Hz, 2H), 1.81 – 1.76 (m, 2H), 1.18 – 1.12 (m, 3H), 1.07 (d, *J* = 7.3 Hz, 18H).

^{13}C NMR (151 MHz, $CDCl_3$): For *Z*-isomer: δ 140.4, 107.7, 45.0, 32.9, 21.2, 17.9, 12.1. For *E*-isomer: δ 142.0, 108.9, 44.5, 33.1, 24.5, 17.9, 12.1.

HRMS (ESI): m/z: $[M+H]^+$ calc'd for $C_{14}H_{30}ClOSi^+$: 277.1754. Found: 277.1732.

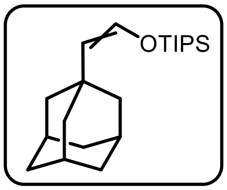

((2-adamantan-1-yl)vinyl)oxy)triisopropylsilane (13a). The reaction was set up using the general procedure **E2** with (2-adamantan-1-yl)acetaldehyde[20] (150 mg, 0.84 mmol) for 30 minutes. Following this, the crude product was purified using alumina column chromatography (hexanes 100%) to yield the indicated product in 77% yield (218 mg) as a colorless liquid (*E:Z* =23:77).

IR (Diamond-ATR, neat) ṽ (cm^{-1}): 2901, 2867, 2848, 1458, 1062.

^{1}H NMR (600 MHz, $CDCl_3$): For *Z*-isomer: δ 6.08 (d, *J* = 6.6 Hz, 1H), 3.99 (d, *J* = 6.5 Hz, 1H), 1.95 – 1.91 (m, 3H), 1.85 (br s, 4H), 1.71 – 1.67 (m, 6H), 1.55 (s, 2H), 1.20 – 1.13 (m, 3H), 1.10 (d, *J* = 6.8 Hz, 18H). For *E*-isomer: δ 6.21 (d, *J* = 12.1 Hz, 1H), 4.96 (d, *J* = 12.2 Hz, 1H), 1.97 –

1.95 (m, 3H), 1.86 – 1.84 (m, 4H), 1.73 – 1.62 (m, 6H), 1.56 – 1.54 (m, 2H), 1.20 – 1.13 (m, 3H), 1.10 (d, *J* = 6.8 Hz, 18H).
^{13}C NMR (151 MHz, $CDCl_3$): For *Z*-isomer δ 138.0, 118.8, 42.6, 37.3, 33.8, 29.1, 18.0, 12.0. For *E*-isomer: δ 138.4, 124.1, 43.3, 37.0, 33.8, 28.7, 18.3, 12.2.
HRMS (ESI): m/z: $[M+H]^+$ calc'd for $C_{21}H_{39}OSi^+$: 335.2770. Found: 335.2765.

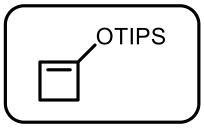

(cyclobut-1-en-1-yloxy)triisopropylsilane (14a). The reaction was set up using the general procedure **E2** with cyclobutanone (74.7 µL, 1.00 mmol) for 30 minutes. Following this, the crude product was purified using alumina column chromatography (hexanes 100%) to yield the indicated product in 71% yield (160 mg) as a colorless liquid. The resulting product has been previously reported and matches the obtained spectroscopic data tabulated below.[11]
^{1}H NMR (600 MHz, $CDCl_3$): δ 4.58 (d, *J* = 1.1 Hz, 1H), 2.61 – 2.57 (m, 2H), 1.97 (td, *J* = 3.2, 1.0 Hz, 2H), 1.20 – 1.14 (m, 3H), 1.09 (d, *J* = 7.4 Hz, 18H).
^{13}C NMR (151 MHz, $CDCl_3$): δ 148.7, 102.8, 34.3, 19.2, 17.8, 12.3.

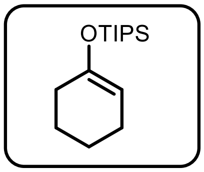

(cyclohex-1-en-1-yloxy)triisopropylsilane (15a). The reaction was set up using the general procedure **E2** with cyclohexanone (104 µL, 1.00 mmol) for 30 minutes. Following this, the crude product was purified using alumina column chromatography (hexanes 100%) to yield the indicated product in 95% yield (241 mg) as a colorless liquid. The resulting product has been previously reported and matches the obtained spectroscopic data tabulated below.[11]
^{1}H NMR (600 MHz, $CDCl_3$): 4.89 – 4.85 (m, 1H), 2.06 – 1.96 (m, 4H), 1.69 – 1.62 (m, 2H), 1.53 – 1.46 (m, 2H), 1.20 – 1.11 (m, 3H), 1.08 – 1.05 (m, 18H).
^{13}C NMR (151 MHz, $CDCl_3$): 151.6, 104.2, 30.7, 24.5, 24.0, 23.1, 18.4, 13.4.

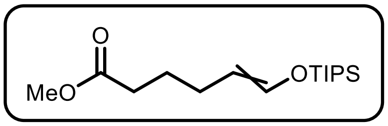

methyl-6-((triisopropylsilyl)oxy)hex-5-enoate (16a). The reaction was set up using general procedure **E2** with methyl-6-oxohexanoate (100 mg, 0.69 mmol) for 30 minutes. Following this, the crude product was purified using alumina column chromatography (hexanes 100%) to yield the indicated product in 79% yield (165 mg) as a colorless liquid (*E:Z*=10:90).
IR (Diamond-ATR, neat) ṽ (cm^{-1}): 2954, 2667, 2162, 1653, 1458, 1114.
^{1}H NMR (600 MHz, $CDCl_3$): For *Z*-isomer: δ 6.23 (d, *J* = 6.0 Hz, 1H), 4.29 (dt, *J* = 7.2, 7.1 Hz, 1H), 3.59 (s, 3H), 2.26 (t, *J* = 7.7 Hz, 2H), 2.06 (at, *J* = 7.3 Hz, 2H), 1.62 (ap, *J* = 7.6 Hz, 2H), 1.08 (adq, *J* = 13.0, 7.2 Hz, 3H), 1.00 (d, *J* = 6.7 Hz, 18H). For *E*-isomer: δ 6.24 (d, 1H), 4.88 (dt, *J* = 11.7, 7.5 Hz, 1H), 3.59 (s, 3H), 2.24 (d, *J* = 7.7 Hz, 2H), 2.10 (aq, *J* = 8.4 Hz, 2H), 1.59 (ap, *J* = 7.4 Hz, 2H), 1.08 (adq, *J* = 13.0, 7.2 Hz, 3H), 1.00 (d, *J* = 6.7 Hz, 18H).
^{13}C NMR (151 MHz, $CDCl_3$): For *Z*-isomer: δ 174.6, 140.0, 108.5, 51.6, 33.8, 25.1, 23.2, 17.9, 12.1. For *E*-isomer: δ 177.1, 141.6, 109.9, 53.7, 33.4, 26.9, 25.8, 18.3, 13.5.
HRMS (ESI): m/z: $[M+H]^+$ calc'd for $C_{16}H_{33}O_3Si^+$: 301.2199. Found: 301.2198.

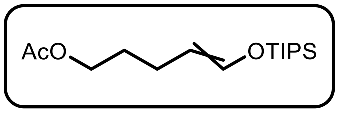

5-((triisopropylsilyl)oxy)pent-4-en-1-yl-acetate (17a). The reaction was set up using general procedure **E2** with 5-oxopentyl acetate (369 mg, 2.60 mmol) for 30 minutes. Following this, the crude product was purified using alumina column chromatography (hexanes 100%) to yield the indicated product in 73% yield (561 mg) as a colorless liquid (*E:Z*=9:91).
IR (Diamond-ATR, neat) ṽ (cm^{-1}): 2934, 2865, 1623, 1428, 1224.

^{1}H NMR (600 MHz, $CDCl_3$): For *Z*-isomer: δ 6.30 (d, *J* = 5.8 Hz, 1H), 4.39 (td, *J* = 7.1, 5.6 Hz, 1H), 4.07 (t, *J* = 6.7 Hz, 2H), 2.17 (q, *J* = 7.4 Hz, 2H), 2.03 (s, 3H), 1.68 (ap, *J* = 7.1 Hz, 2H), 1.15 (dt, *J* = 14.7, 7.4 Hz, 3H), 1.07 (d, *J* = 6.8 Hz, 18H). For *E*-isomer: δ 6.33 (d, *J* = 11.7 Hz, 1H), 4.98 (dt, *J* = 11.3, 7.4 Hz, 1H), 4.06 (t, *J* = 6.7 Hz, 2H), 2.04 (s, 3H), 1.96 (aq, *J* = 7.3 Hz, 2H), 1.68 (ap, *J* = 7.0 Hz, 2H), 1.15 (dt, *J* = 14.7, 7.4 Hz, 3H), 1.07 (d, *J* = 6.8 Hz, 18H).
^{13}C NMR (151 MHz, $CDCl_3$): For *Z*-isomer: δ 171.4, 140.0, 108.3, 64.5, 28.8, 20.2, 18.3, 17.8, 12.1. For *E*-isomer: δ 171.4, 141.5, 109.7, 64.1, 29.5, 23.9, 17.9, 13.5, 12.4.
HRMS (ESI): m/z: $[M+H]^+$ calc'd for $C_{16}H_{33}O_3Si^+$: 301.2199. Found: 301.2185.

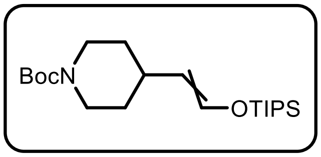

tert-butyl (E)-4-(2-((triisopropylsilyl)oxy)vinyl)piperidine-1-carboxylate (18a). The reaction was set up using the general procedure **E2** with *tert*-butyl 4-(2-oxoethyl)piperidine-1-carboxylate (150 mg, 0.66 mmol) for 30 minutes. Following this, the crude product was purified using alumina column chromatography (hexanes/EtOAc 100/0 to hexanes/EtOAc 90/10) to yield the indicated product in 54% yield (137 mg) as a colorless liquid (*E:Z* =15:85).
IR (Diamond-ATR, neat) ṽ (cm^{-1}): 2948, 2865, 1678, 1464, 1424, 1216, 1058.
^{1}H NMR (600 MHz, $CDCl_3$): For *Z*-isomer: δ 6.24 (dd, *J* = 5.8, 1.2 Hz, 1H), 4.26 (dd, *J* = 8.4, 5.9 Hz, 1H), 4.13 – 3.99 (m, 2H), 2.97 – 2.88 (m, 1H), 2.88 – 2.77 (m, 1H), 2.72 – 2.63 (m, 1H), 1.67 (dd, *J* = 12.8, 3.3 Hz, 2H), 1.34 – 1.27 (m, 2H), 1.19 – 1.10 (m, 3H), 1.09 (d, *J* = 7.2 Hz, 18H), 1.06 (s, 9H). For *E*-isomer: δ 6.35 (dd, *J* = 12.0, 1.0 Hz, 1H), 4.96 (dd, *J* = 11.9, 7.9 Hz, 1H), 4.13 – 3.99 (m, 2H), 2.97 – 2.88 (m, 1H), 2.88 – 2.77 (m, 1H), 2.72 – 2.63 (m, 1H), 1.67 (dd, *J* = 12.8, 3.3 Hz, 2H), 1.34 – 1.27 (m, 2H), 1.19 – 1.10 (m, 3H), 1.09 (d, *J* = 7.2 Hz, 18H), 1.06 (s, 9H).
^{13}C NMR (126 MHz, $CDCl_3$, 333K) For *Z*-isomer: δ 154.2, 139.1, 113.6, 31.7, 18.1, 17.9, 12.5, 12.3. For *E*-isomer: δ 154.2, 140.7, 115.7, 35.1, 18.3, 17.9, 13.8, 12.4.
HRMS (ESI): m/z: $[M+H]^+$ calc'd for $C_{21}H_{42}NO_3Si^+$: 384.2934. Found: 384.2919.

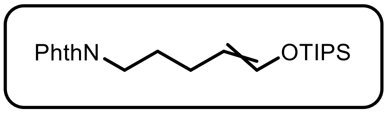

2-(5-((triisopropylsilyl)oxy)pent-4-en-1-yl)isoindolin-E-1,3-dione (19a)

To a solution of 5-((triisopropylsilyl)oxy)pent-4-en-1-yl 4-methylbenzenesulfonate[21] (413 mg, 1.00 mmol) in DMF (5.0 mL, 0.20 M) was added potassium phthalimide (370 mg, 2.00 mmol). The mixture was heated at 50 °C for 16 h, then taken up in EtOAc (15 mL). The organic phase was washed with brine (2 × 20 mL) and H_2O (20 mL). The organic phase was dried over anhydrous Na_2SO_4, filtered and concentrated by rotary evaporator to provide crude mixture. The crude mixture was purified using silica column chromatography (hexanes/EtOAc 95/5) to yield the indicated product in 64% yield (248 mg) as colorless liquid (*E:Z*=8:92).
IR (Diamond-ATR, neat) ṽ (cm^{-1}): 2943, 2866, 1711, 1393, 1037.
^{1}H NMR (600 MHz, $CDCl_3$): For *Z*-isomer: δ 7.83 (dd, *J* = 5.4, 3.1 Hz, 2H), 7.70 (dd, *J* = 5.4, 3.1 Hz, 2H), 6.29 (d, *J* = 5.7 Hz, 1H), 4.44 (td, *J* = 7.2, 5.7 Hz, 1H), 3.70 (t, *J* = 7.4 Hz, 2H), 2.16 (aq, *J* = 7.4 Hz, 2H), 1.73 (ap, *J* = 7.5 Hz, 2H), 1.15 – 1.09 (m, 3H), 1.05 – 1.02 (m, 18H). For *E*-isomer: δ 7.83 (dd, *J* = 5.4, 3.1 Hz, 2H), 7.70 (dd, *J* = 5.4, 3.1 Hz, 2H), 6.36 (d, *J* = 11.8 Hz, 1H), 5.00 (dt, *J* = 11.8, 7.4 Hz, 1H), 3.67 (at, *J* = 7.2 Hz, 2H), 1.95 (aq, *J* = 7.5 Hz, 2H), 1.71 – 1.67 (m, 2H), 1.16 – 1.11 (m, 18H), 1.06 – 1.05 (m, 3H).

^{13}C NMR (151 MHz, $CDCl_3$): For *Z*-isomer: δ 168.6, 140.0, 133.9, 132.4, 123.3, 108.2, 38.0, 28.6, 21.2, 17.9, 12.1. For *E*-isomer: δ 168.6, 141.5, 134.0, 132.3, 123.3, 109.7, 37.8, 29.5, 25.0, 17.9, 12.4.
HRMS (ESI): m/z: $[M+H]^+$ calc'd for $C_{22}H_{34}NO_3Si^+$: 388.2308. Found: 388.2303.

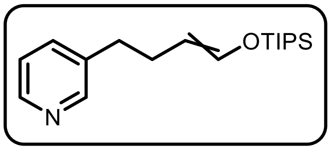

3-(4-((triisopropylsilyl)oxy)but-3-en-1-yl)pyridine (20a). The reaction was set up using the general procedure **E2** with 4-(pyridin-3-yl)butanal[22] (100 mg, 0.67 mmol) for 30 minutes. Following this, the crude product was purified using silica column chromatography (hexanes/NEt_3 90/10) to yield the indicated product in 25% yield (52 mg) as a yellow liquid (*E:Z* =18:82).
IR (Diamond-ATR, neat) ṽ (cm^{-1}): 2941, 2864, 1463, 1112.
^{1}H NMR (600 MHz, $CDCl_3$): For *Z*-isomer: δ 8.47 (d, *J* = 4.5 Hz, 2H), 7.12 (d, *J* = 4.7 Hz, 2H), 6.29 (d, *J* = 5.7 Hz, 1H), 4.38 (q, *J* = 7.2 Hz, 1H), 2.66 (t, *J* = 7.7 Hz, 2H), 2.44 (aq, *J* = 7.7 Hz, 2H), 1.14 (ahept, *J* = 7.2 Hz, 3H), 1.06 (d, *J* = 5.6 Hz, 18H). For *E*-isomer: δ 8.47 (d, *J* = 4.5 Hz, 2H), 7.12 (d, *J* = 4.7 Hz, 2H), 6.27 (s, 1H), 4.97 (dt, *J* = 11.7, 7.5 Hz, 1H), 2.63 (ad, *J* = 7.5 Hz, 2H), 2.37 (adt, *J* = 19.1, 7.3 Hz, 2H), 1.13 (ahept, *J* = 7.2 Hz, 3H), 1.06 (d, *J* = 5.6 Hz, 18H).
^{13}C NMR (151 MHz, $CDCl_3$):): For *Z*-isomer δ 149.6, 140.1, 124.0, 107.7, 35.1, 24.3, 18.1, 17.7, 13.4, 11.9.
The low abundance of the *E*-isomer prevents full characterization from the *E:Z* mixture used for the reaction.
HRMS (ESI): m/z: $[M+H]^+$ calc'd for $C_{18}H_{32}NOSi^+$: 306.2253. Found: 306.2249.

ii) NMR Spectra of Intermolecular Substrates

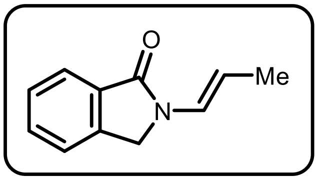

(E)-2-(prop-1-en-1-yl)isoindolin-1-one(4a).

^{1}H NMR (600 MHz, $CDCl_3$):

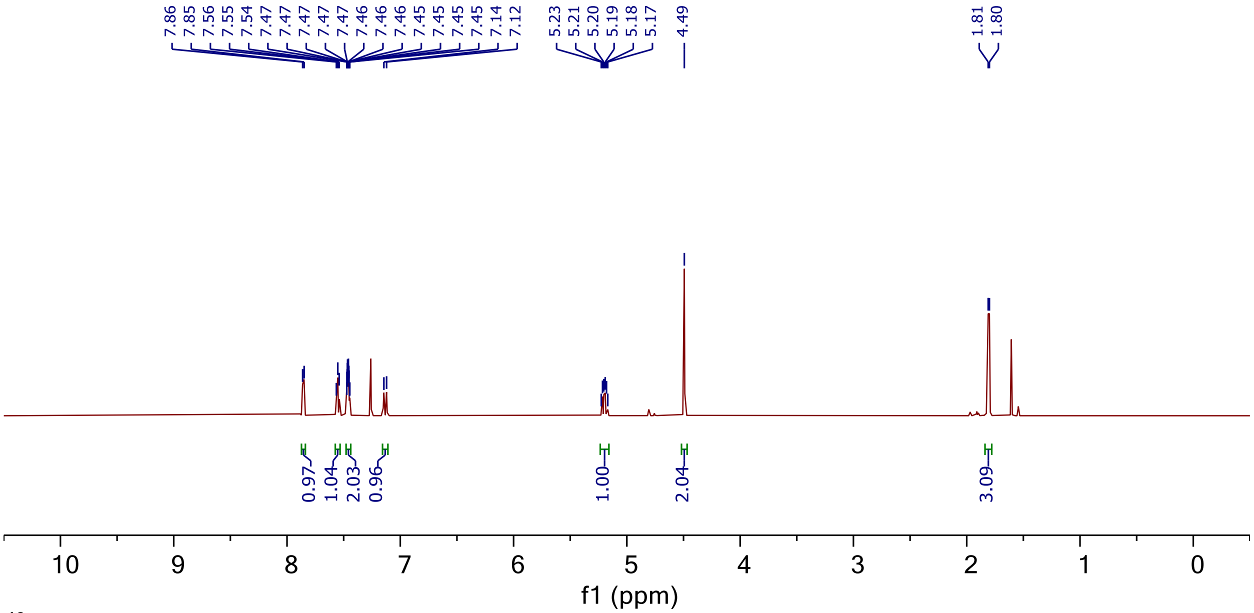

^{13}C NMR (151 MHz, $CDCl_3$):

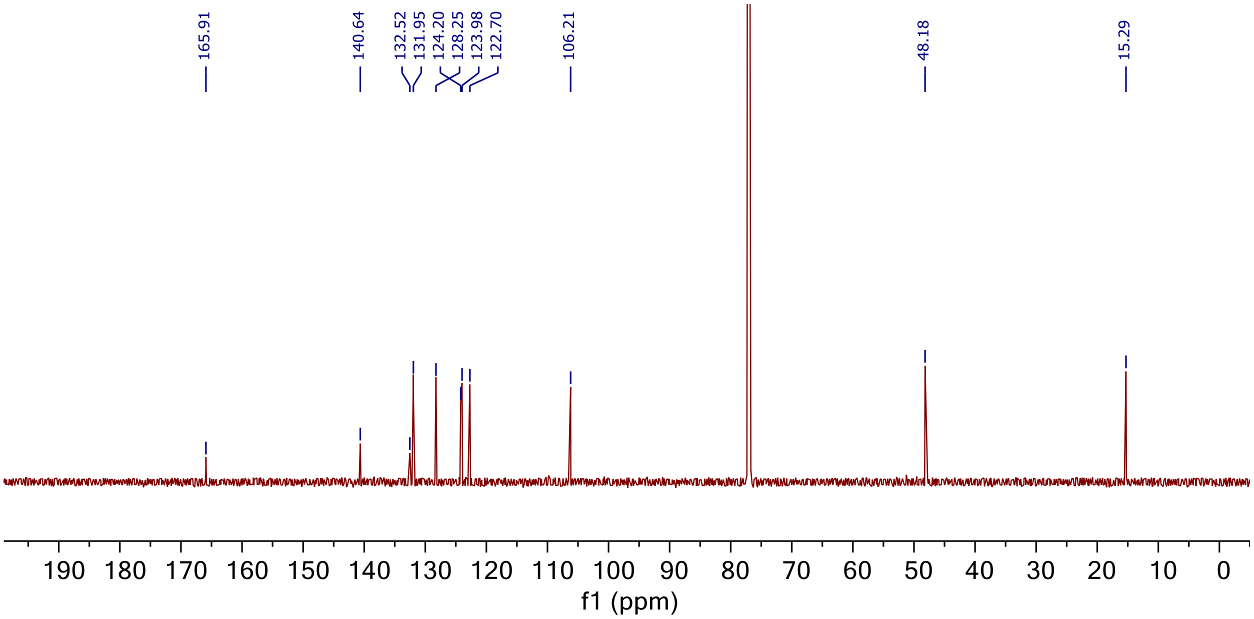

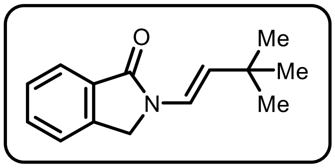

(E)-2-(3,3-dimethylbut-1-en-1-yl)isoindolin-1-one (5a)

^{1}H NMR (600 MHz, $CDCl_3$):

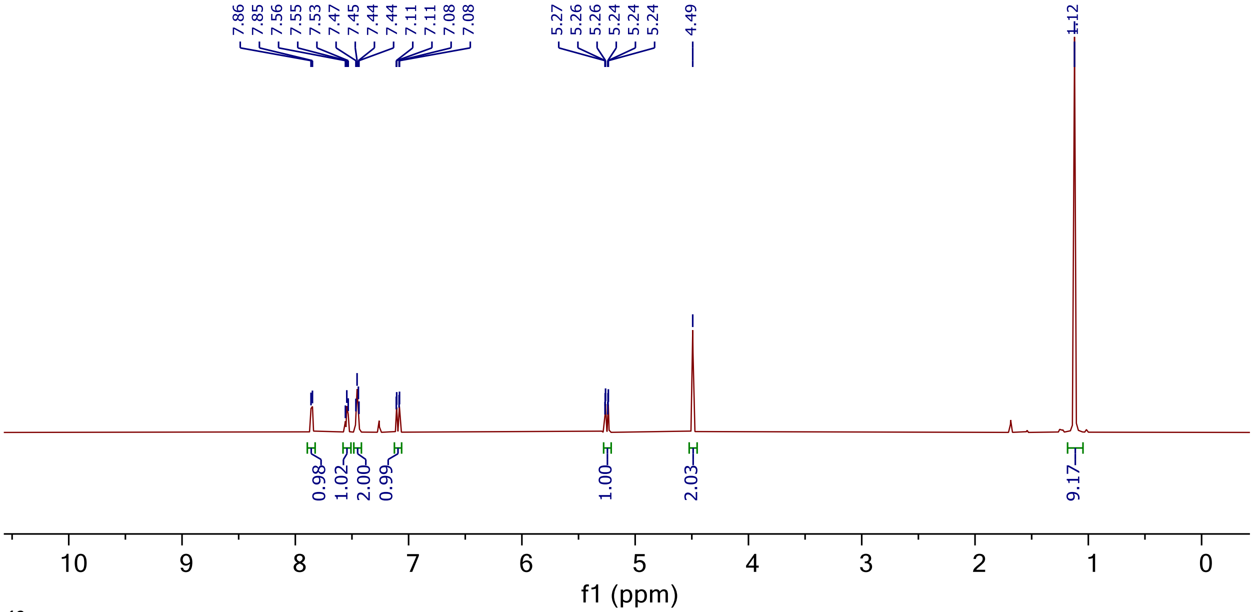

^{13}C NMR (151 MHz, $CDCl_3$):

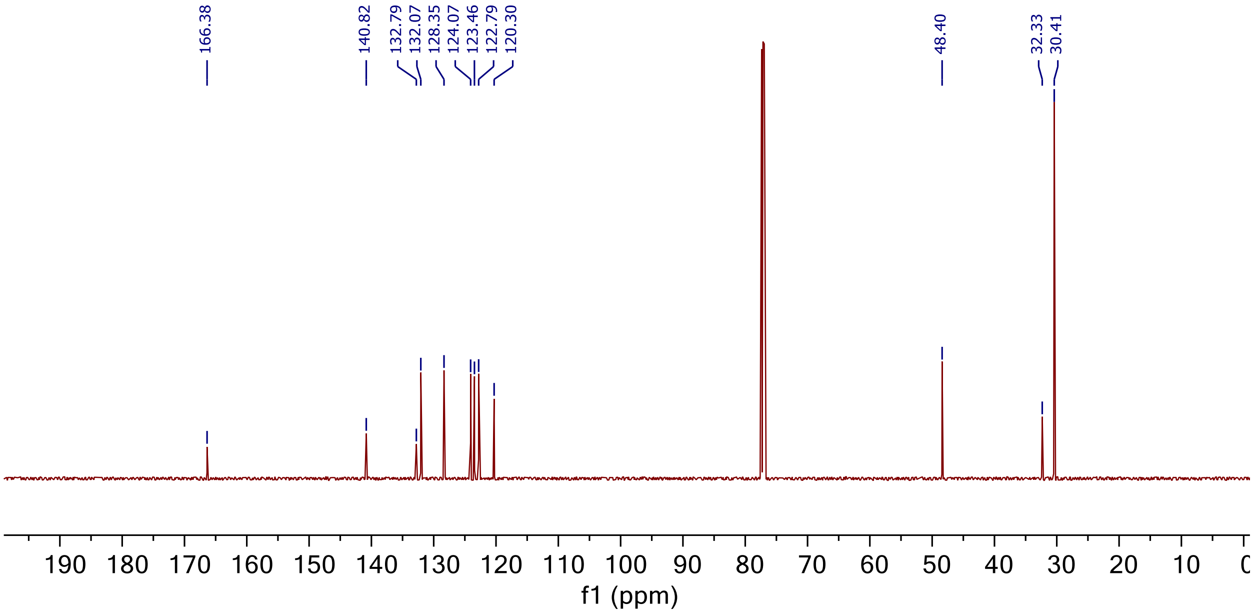

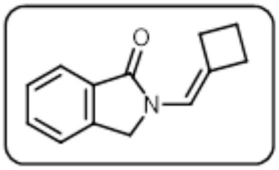

2-(cyclobutylidenemethyl)isoindolin-1-one (6a).

^1H NMR (600 MHz, $CDCl_3$):

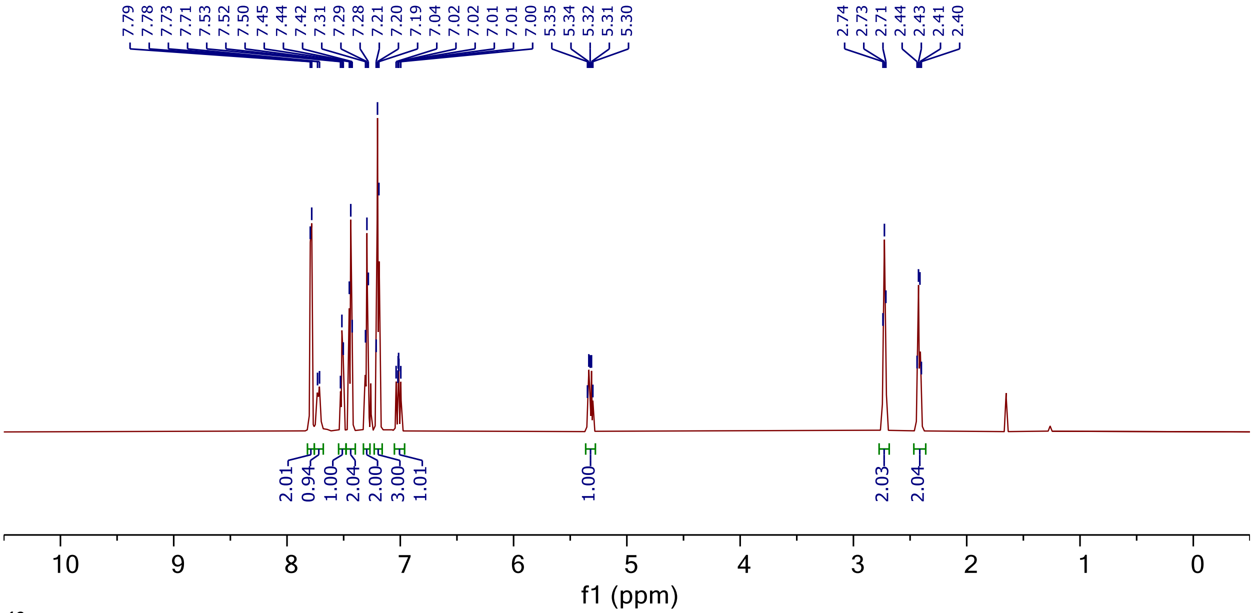

^{13}C NMR (151 MHz, $CDCl_3$):

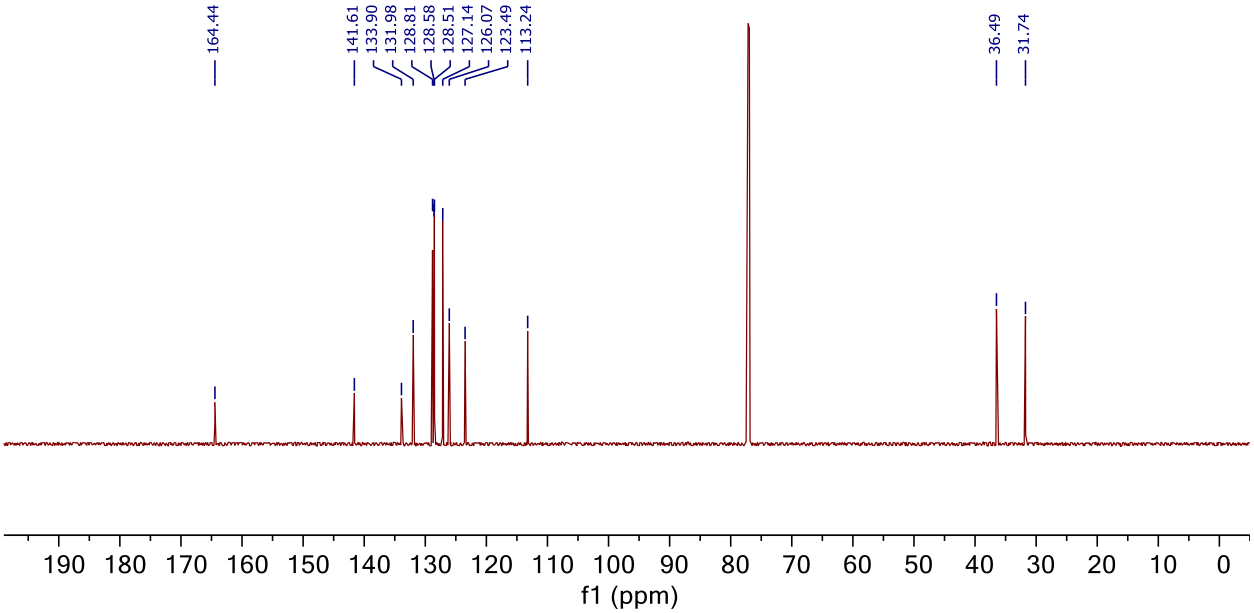

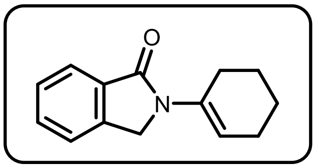

2-(cyclohex-1-en-1-yl)isoindolin-1-one (7a)

^{1}H NMR (600 MHz, $CDCl_3$):

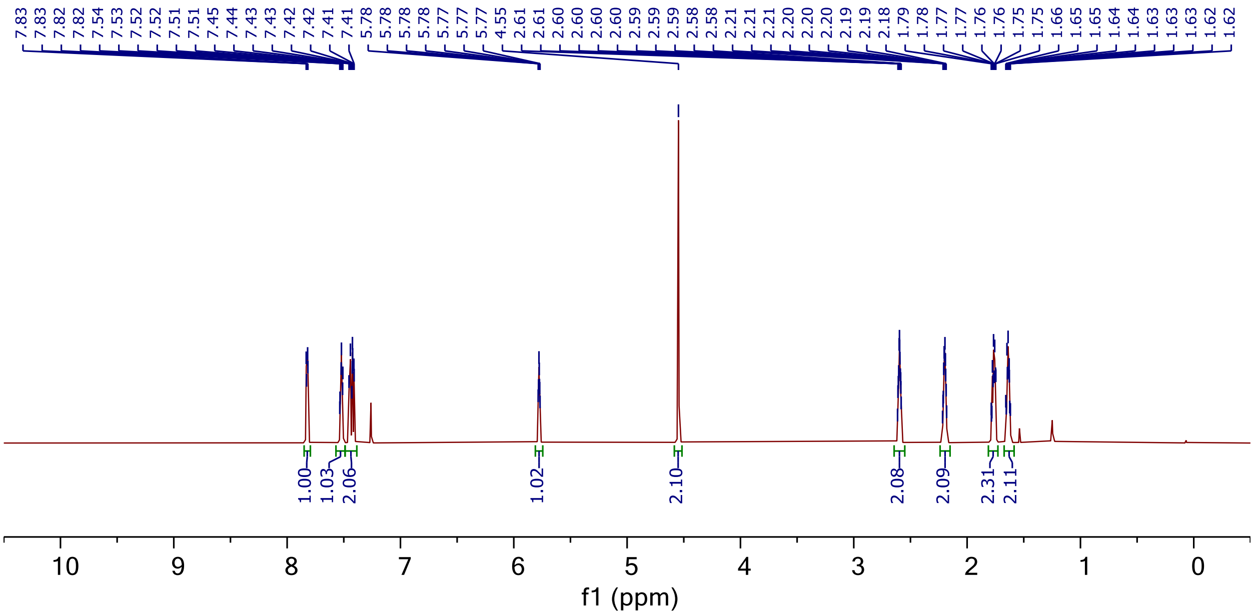

^{13}C NMR (151 MHz, $CDCl_3$):

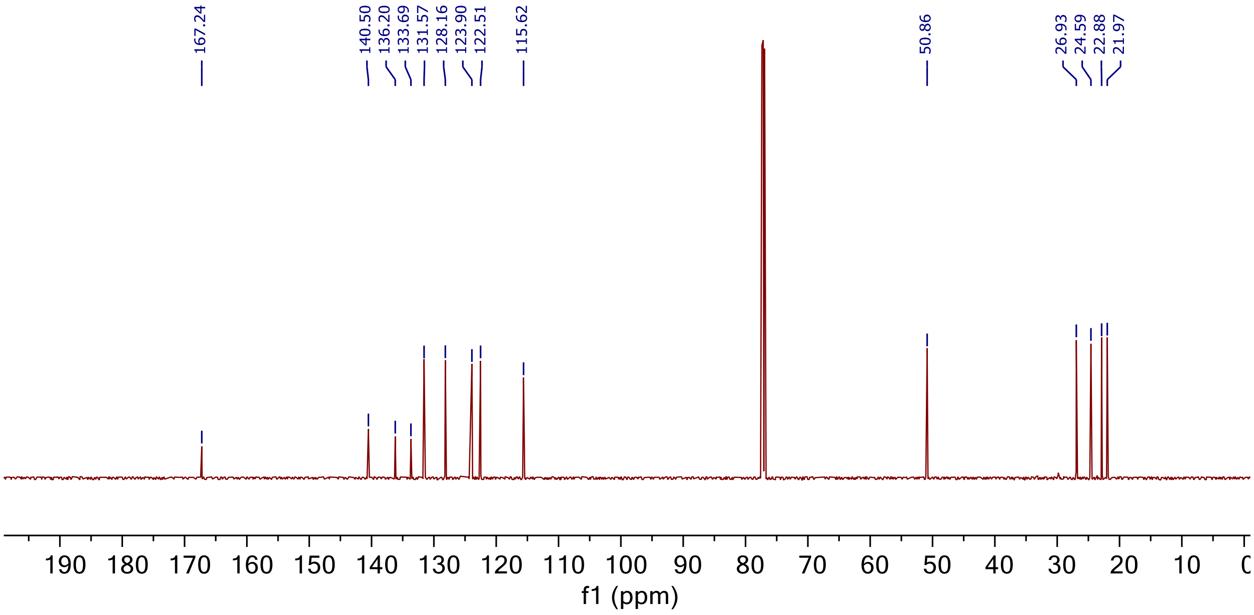

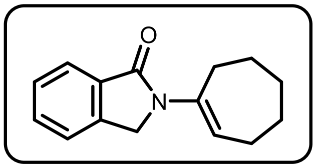

2-(cyclohept-1-en-1-yl)isoindolin-1-one (8a).

^{1}H NMR (600 MHz, $CDCl_3$):

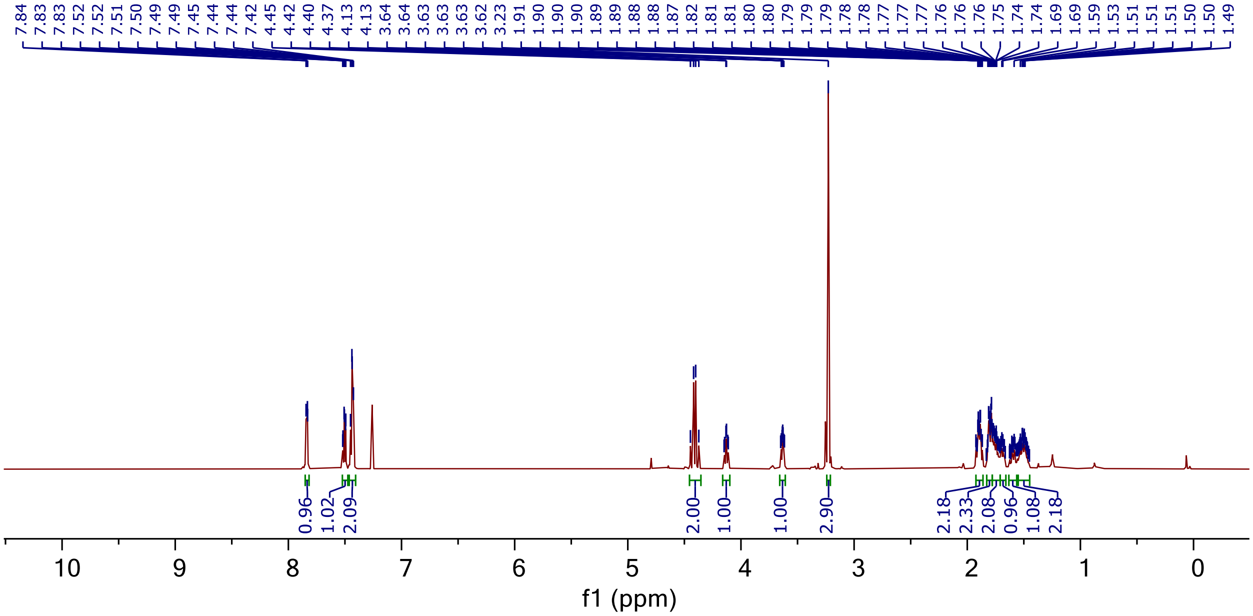

^{13}C NMR (151 MHz, $CDCl_3$):

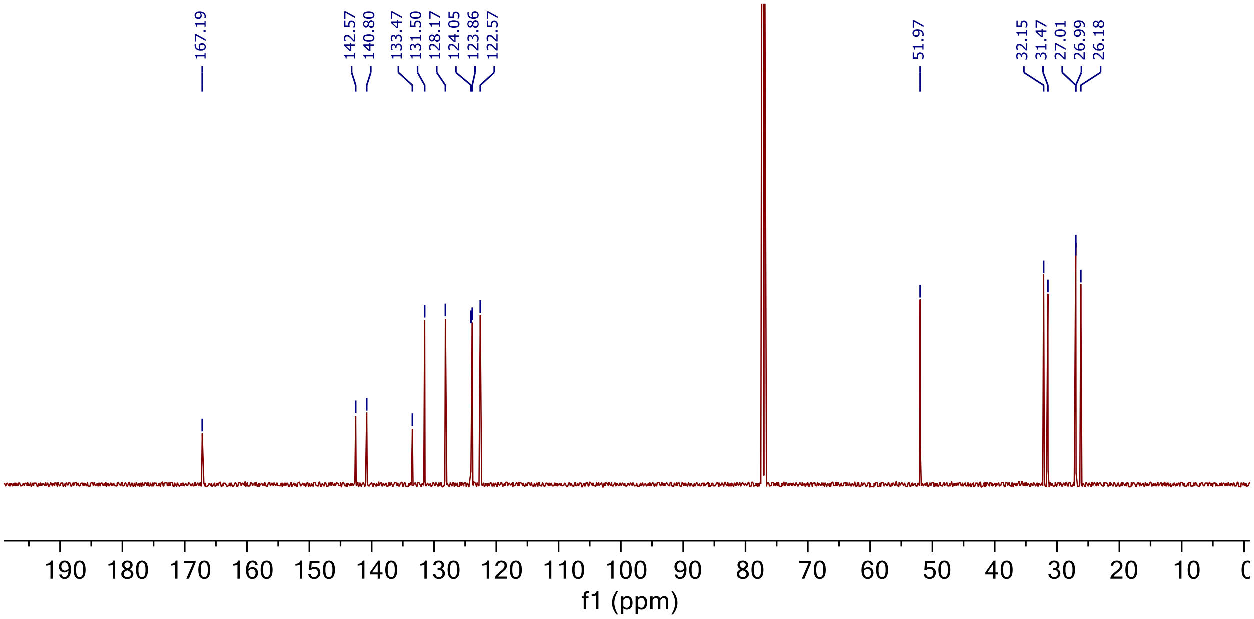

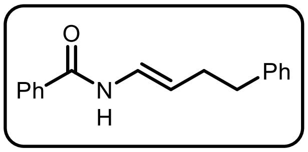

(E)-N-(4-phenylbut-1-en-1-yl)benzamide (9a).

^{1}H NMR (600 MHz, $CDCl_3$):

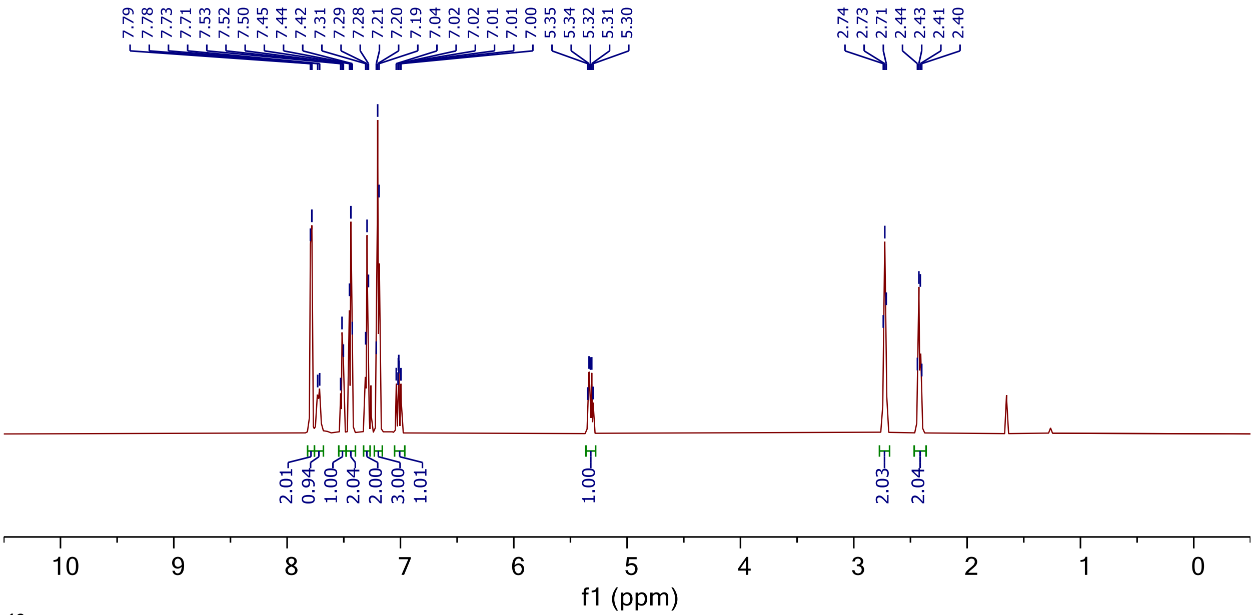

^{13}C NMR (600 MHz, $CDCl_3$):

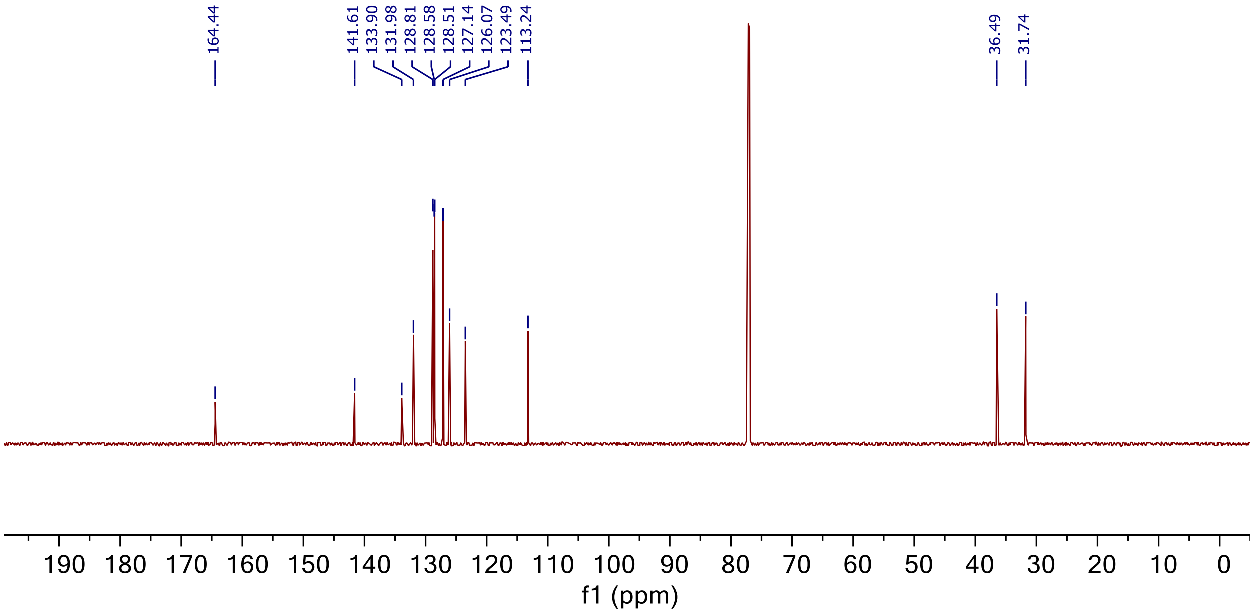

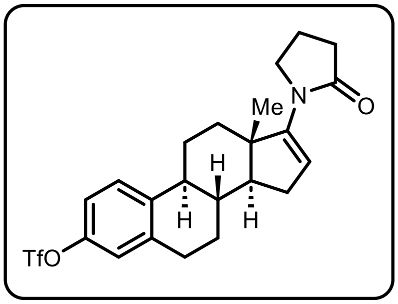

(8R,9S,13S,14S)-13-methyl-17-(2-oxopyrrolidin-1-yl)-7,8,9,11,12,13,14,15-octahydro-6H-cyclopenta[a]phenanthren-3-yl trifluoromethanesulfonate (10a).

^{1}H NMR (600 MHz, $CDCl_3$):

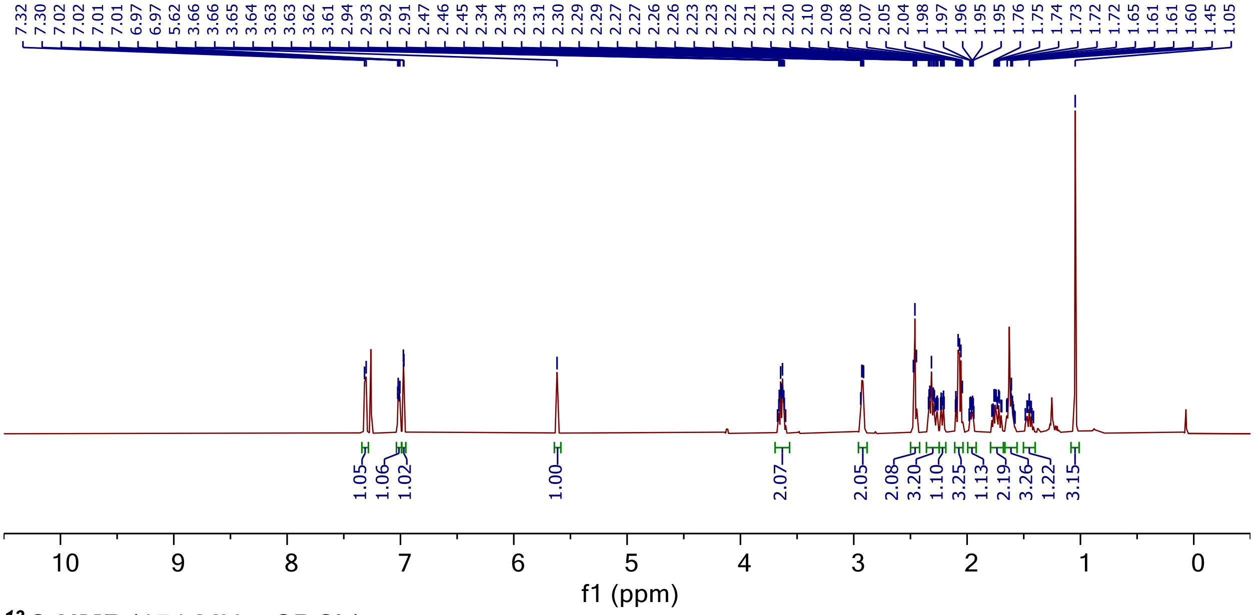

^{13}C NMR (151 MHz, $CDCl_3$):

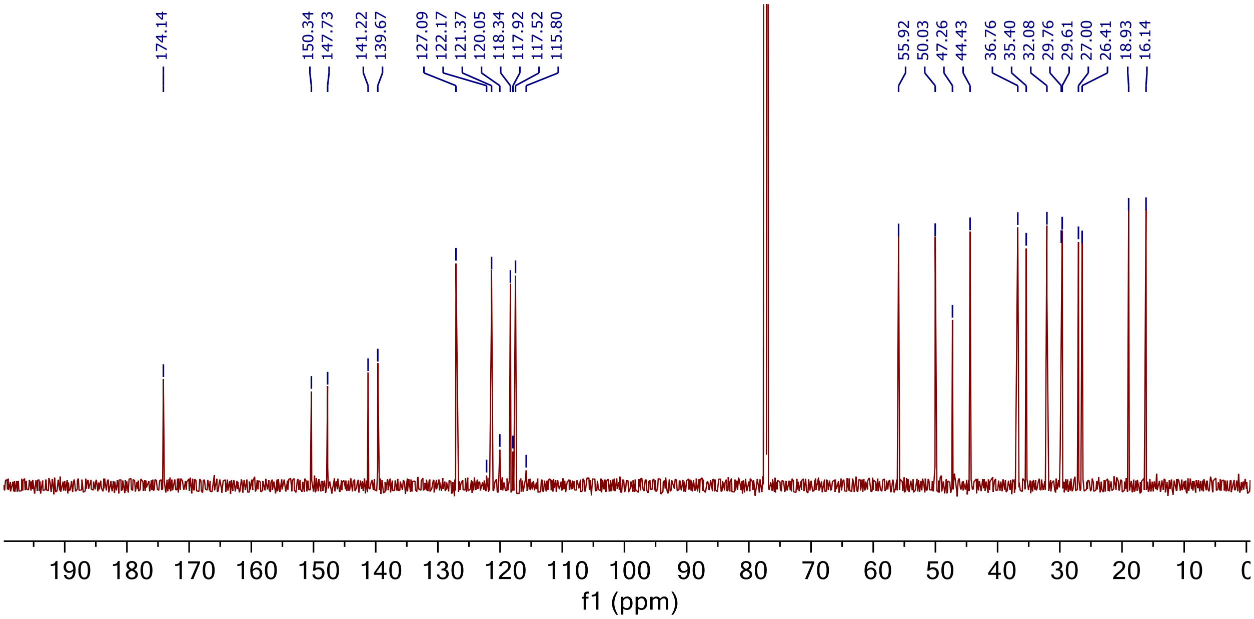

^{19}***F NMR*** (471 MHz, $CDCl_3$):

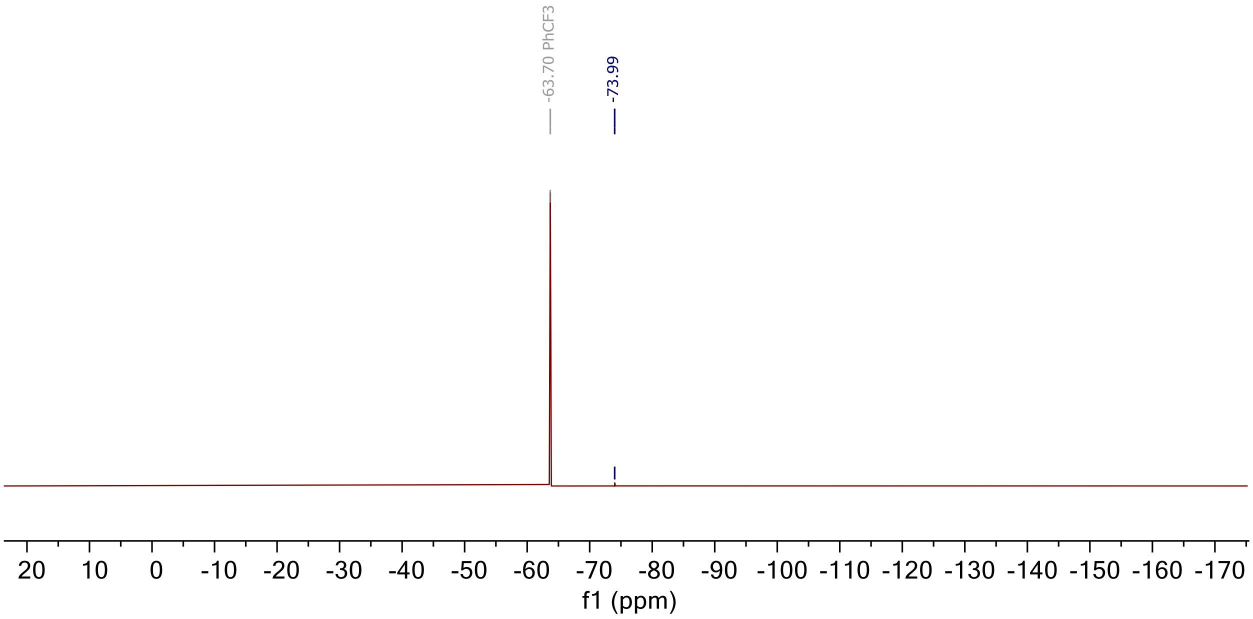

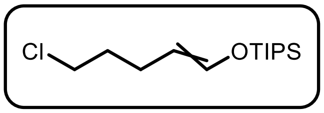

((5-chloropent-1-en-1-yl)oxy)triisopropylsilane (12a).

^{1}H NMR (600 MHz, $CDCl_3$)

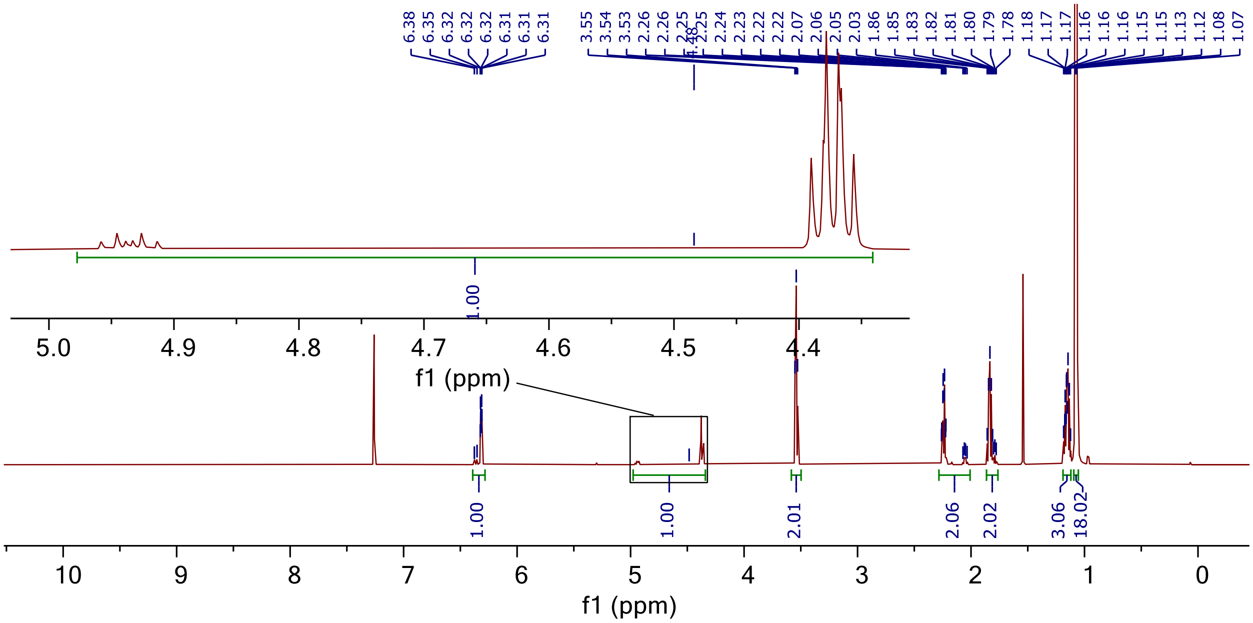

^{13}C NMR (151 MHz, $CDCl_3$):

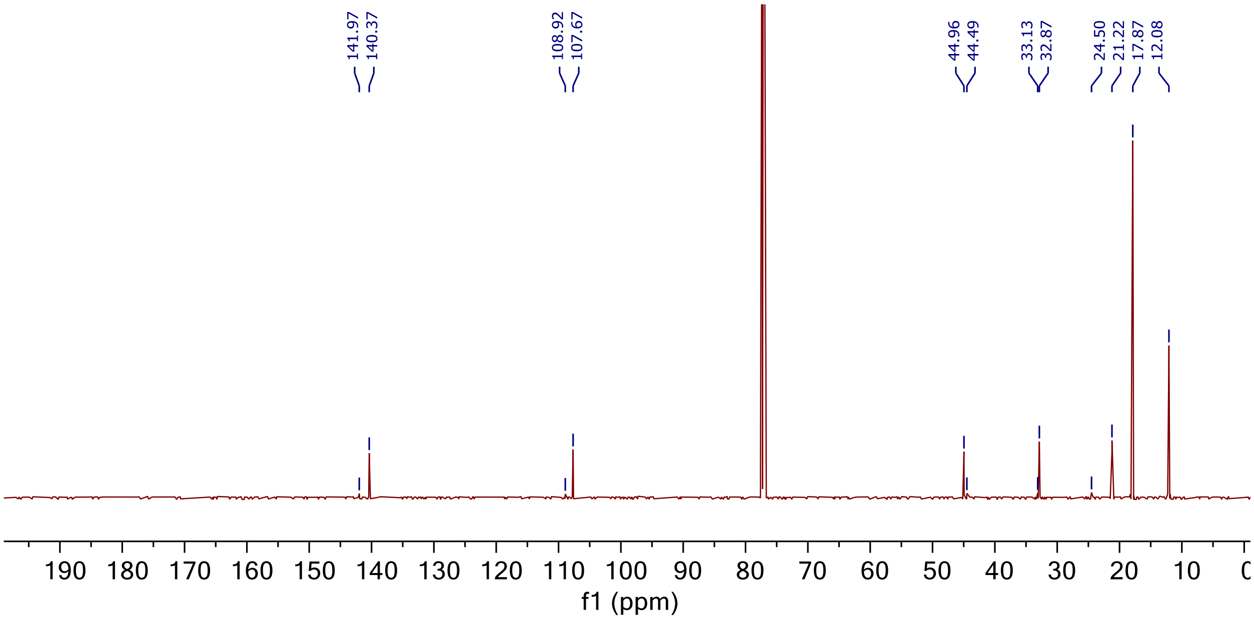

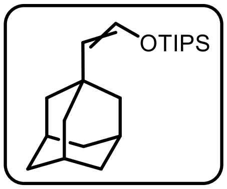

((2-((3r,5r,7r)-adamantan-1-yl)vinyl)oxy)triisopropylsilane (13a).

^{1}H NMR (600 MHz, $CDCl_3$):

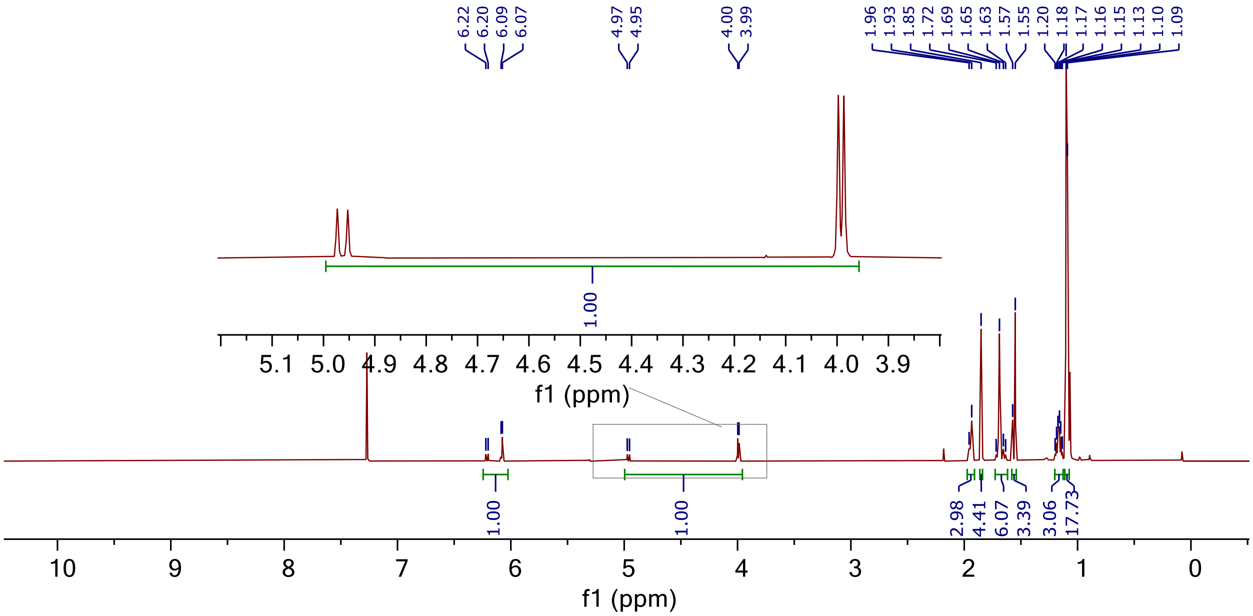

^{13}C NMR (151 MHz, $CDCl_3$):

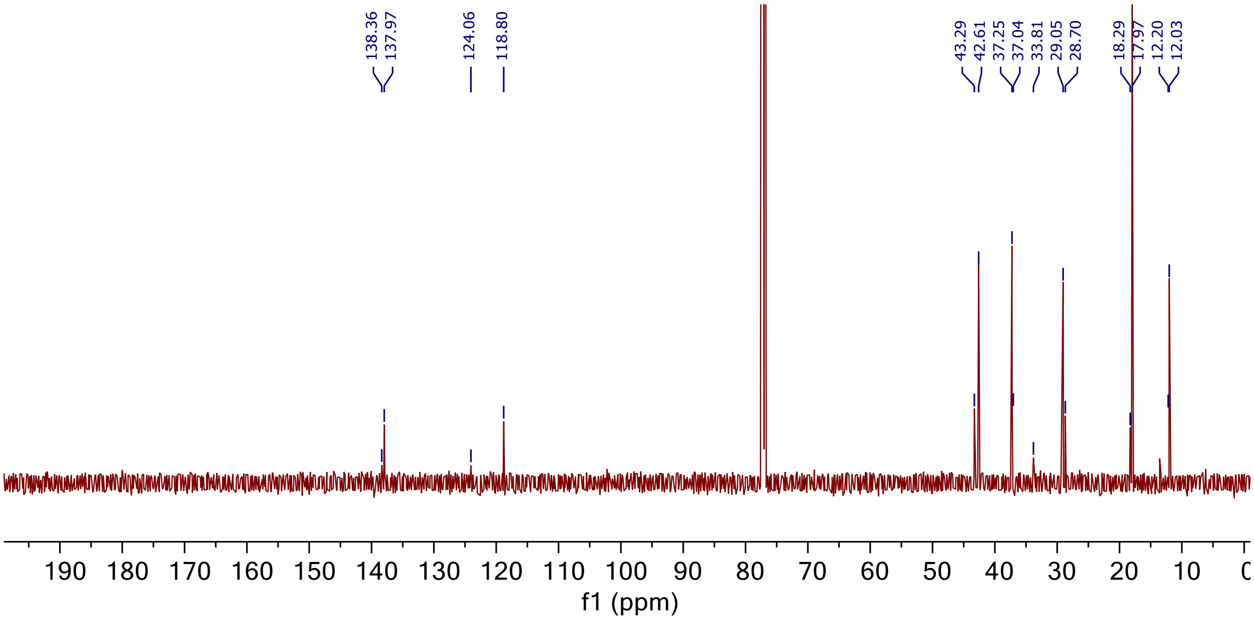

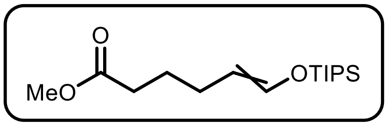

methyl-6-((triisopropylsilyl)oxy)hex-5-enoate (16a)

^{1}H NMR (600 MHz, $CDCl_3$):

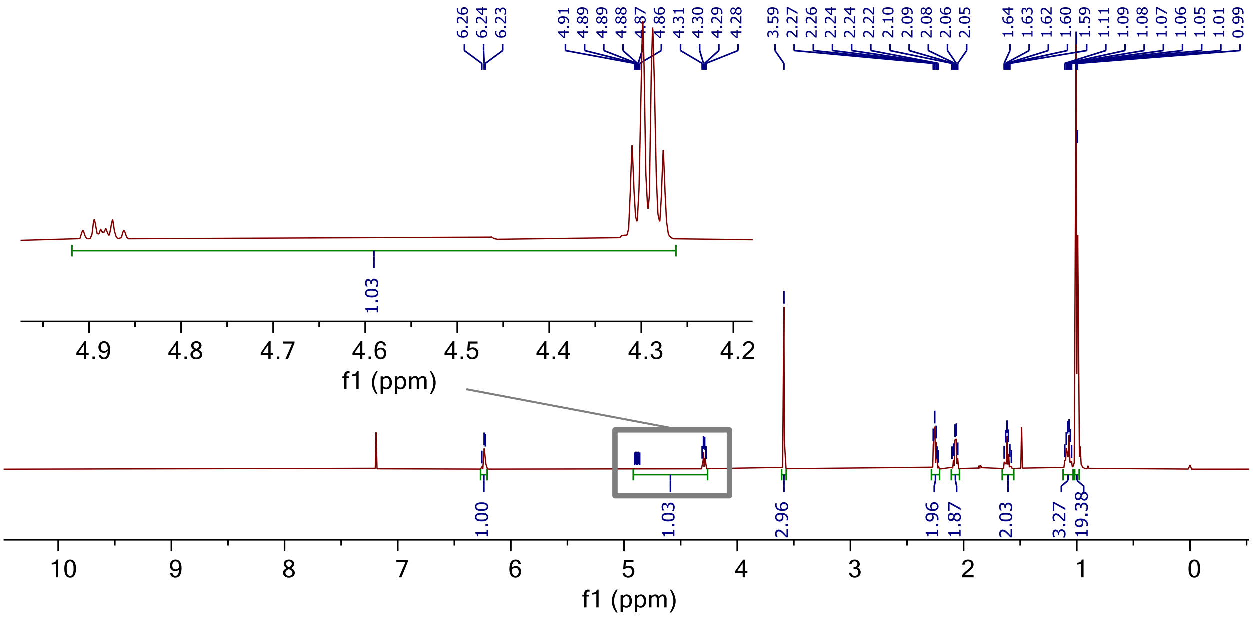

^{13}C NMR (151 MHz, $CDCl_3$):

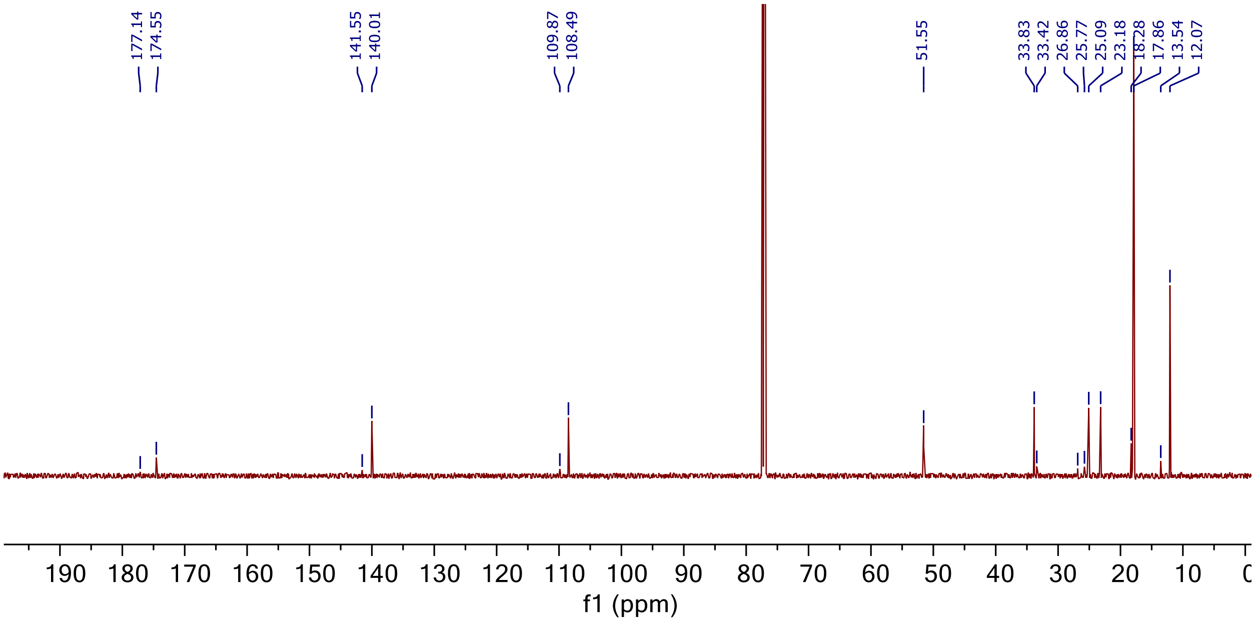

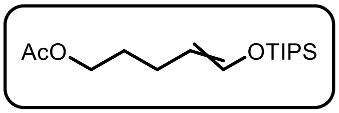

5-((triisopropylsilyl)oxy)pent-4-en-1-yl-acetate (17a)

^{1}H NMR (600 MHz, $CDCl_3$):

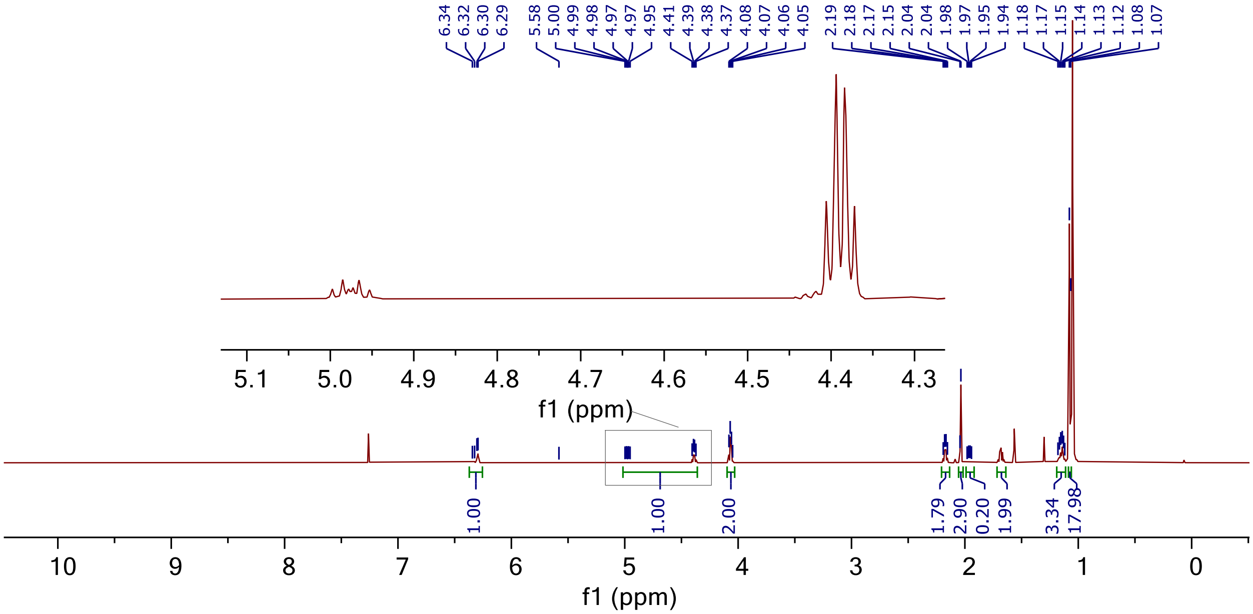

^{13}C NMR (151 MHz, $CDCl_3$):

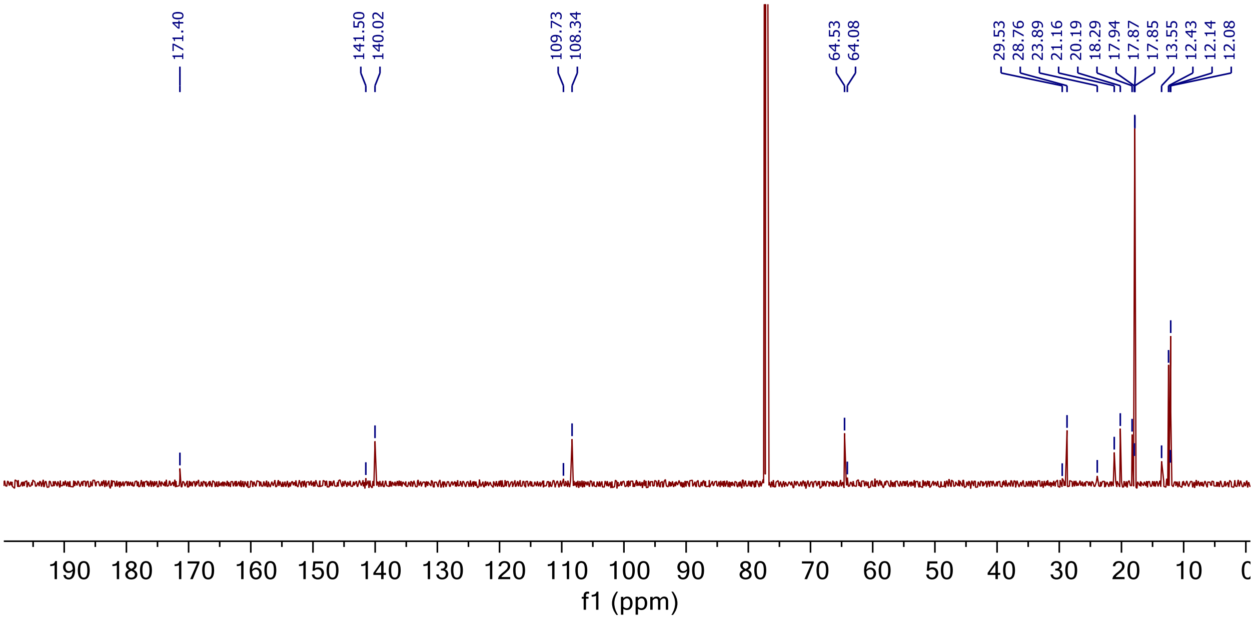

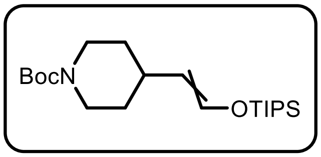

tert-butyl (E)-4-(2-((triisopropylsilyl)oxy)vinyl)piperidine-1-carboxylate (18a).

^{1}H NMR (600 MHz, $CDCl_3$):

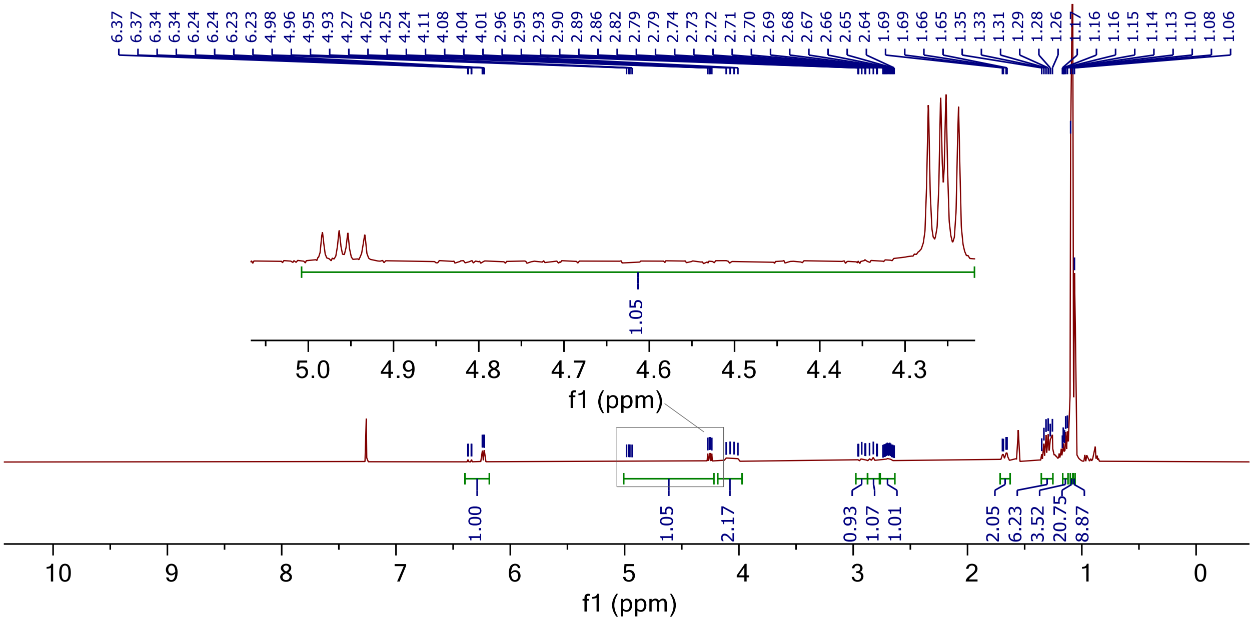

^{13}C NMR (151 MHz, $CDCl_3$):

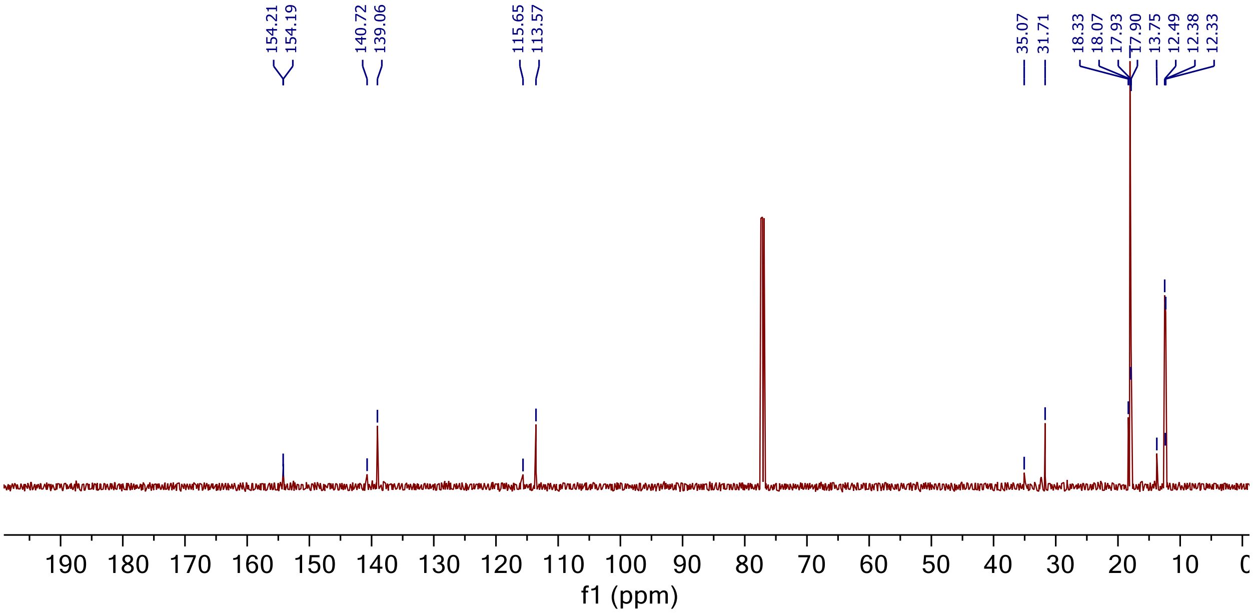

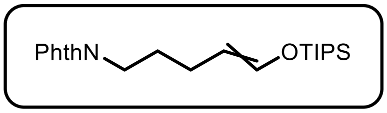

2-(5-((triisopropylsilyl)oxy)pent-4-en-1-yl)isoindoline-1,3-dione (19a).

^{1}H NMR (600 MHz, $CDCl_3$):

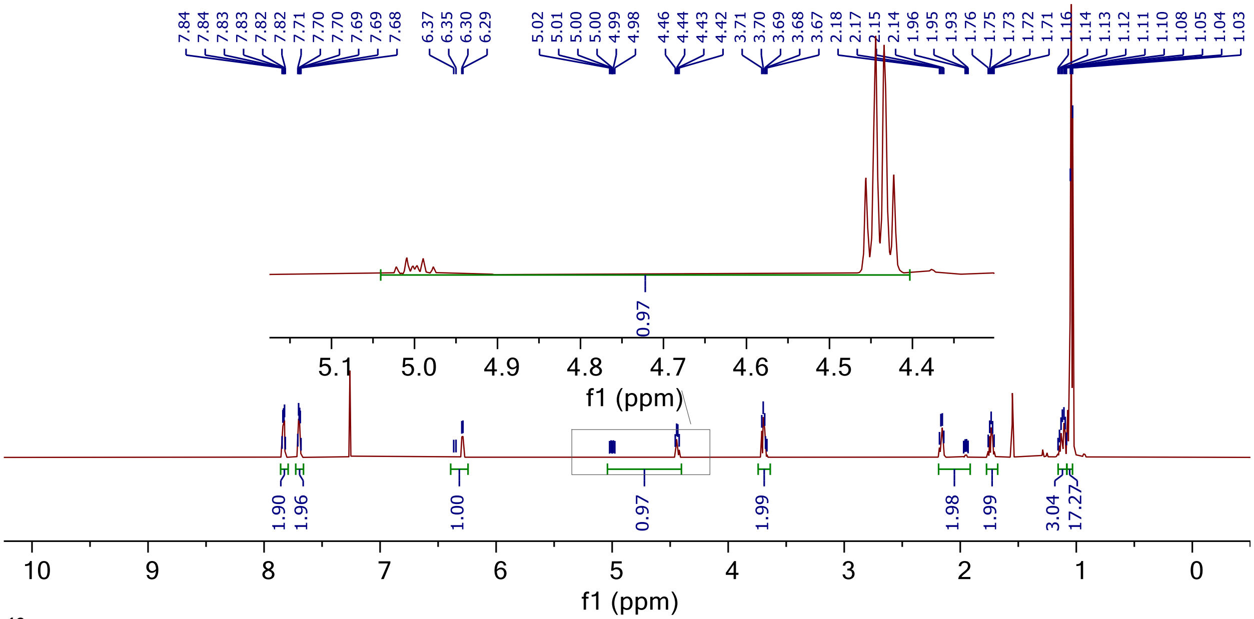

^{13}C NMR (151 MHz, $CDCl_3$):

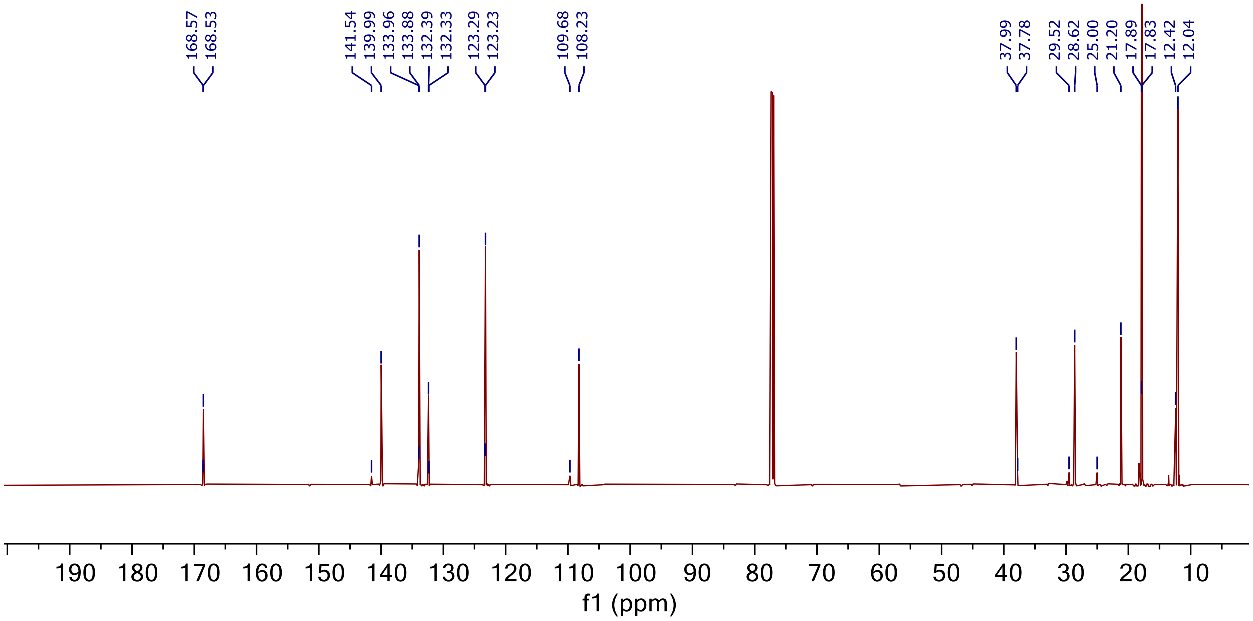

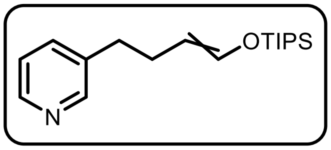

3-(4-((triisopropylsilyl)oxy)but-3-en-1-yl)pyridine (20a).

¹H NMR (600 MHz, $CDCl_3$):

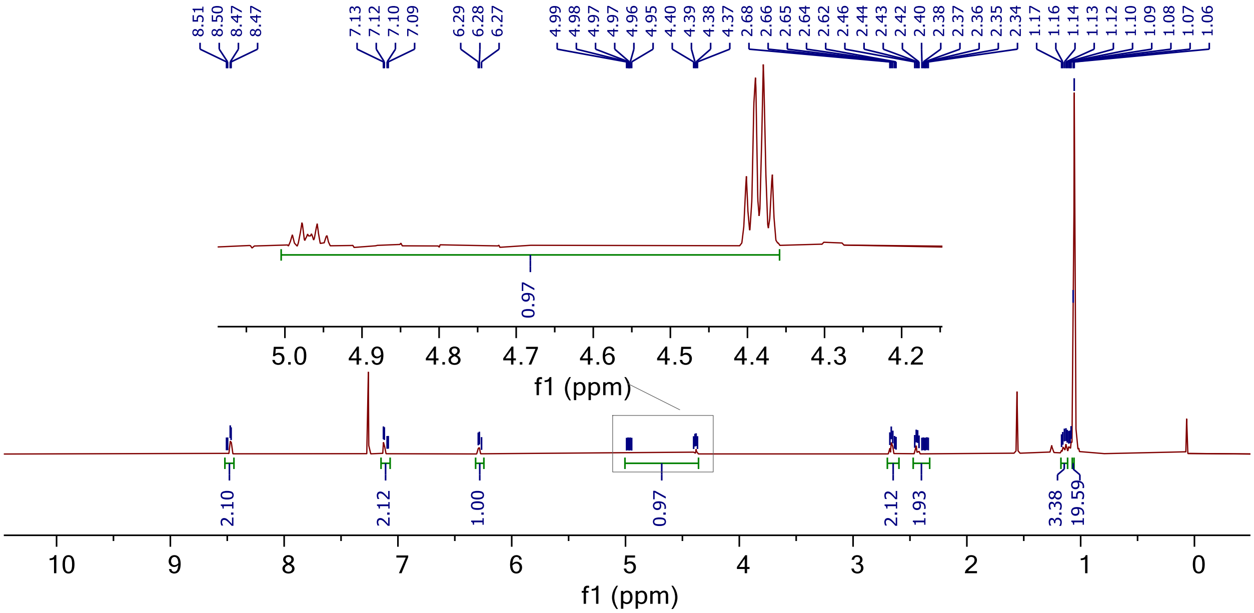

¹³C NMR (151 MHz, $CDCl_3$):

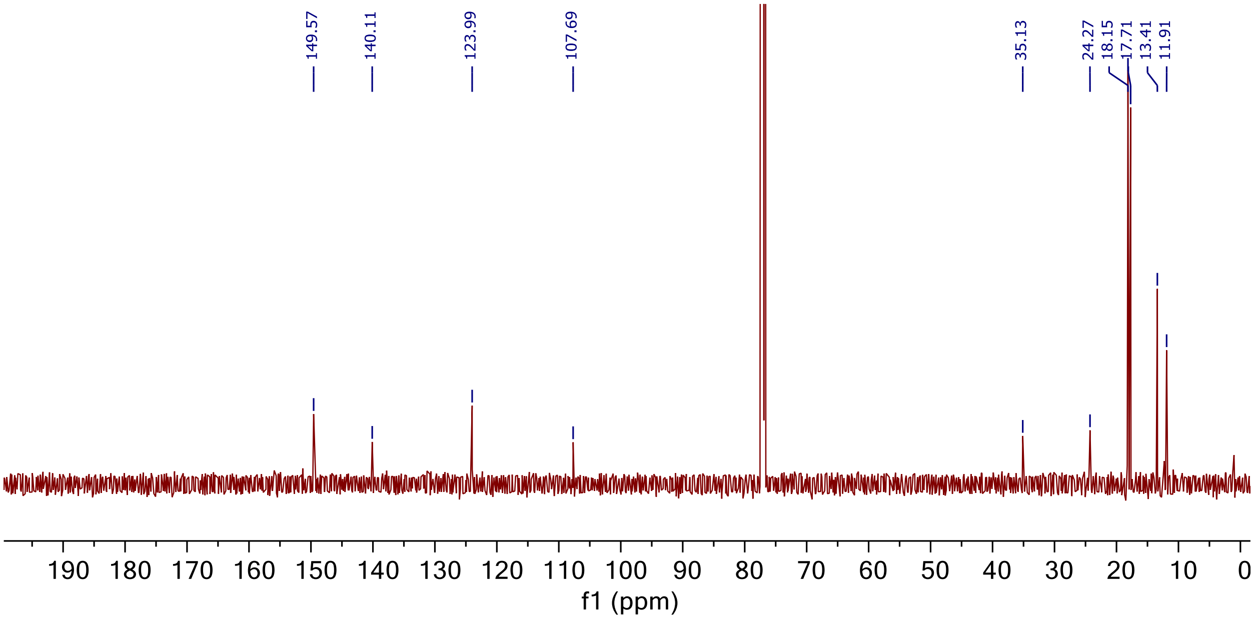

F. Mechanistic Studies

i) Crude NMR Quantification

General information: The crude ^{1}H NMR was acquired following substrate treatment with slightly modified general procedure **H2**: ***PC1*** (0.0025 mmol, 2.5 mol%), $TRIP_2S_2$ (0.0050 mmol, 5.0 mol%) in $PhCF_3$ (0.2 mL) under nitrogen atmosphere, 25 °C, with 365 nm light irradiation. The crude reaction mixture was concentrated down *in vacuo*, dissolved in $CDCl_3$ and 1,1,2,2-tetrachloroethane was added as an internal standard. The spectrum was acquired on a Bruker Avance III HD 400 MHz spectrometer.

a) **Product 21b, quantification of remaining alcohol starting material**[a]

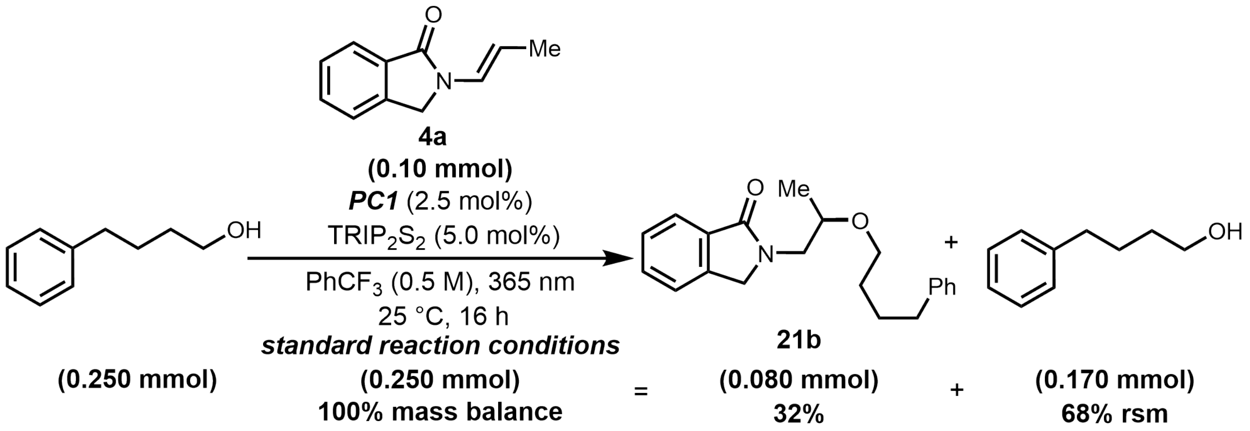

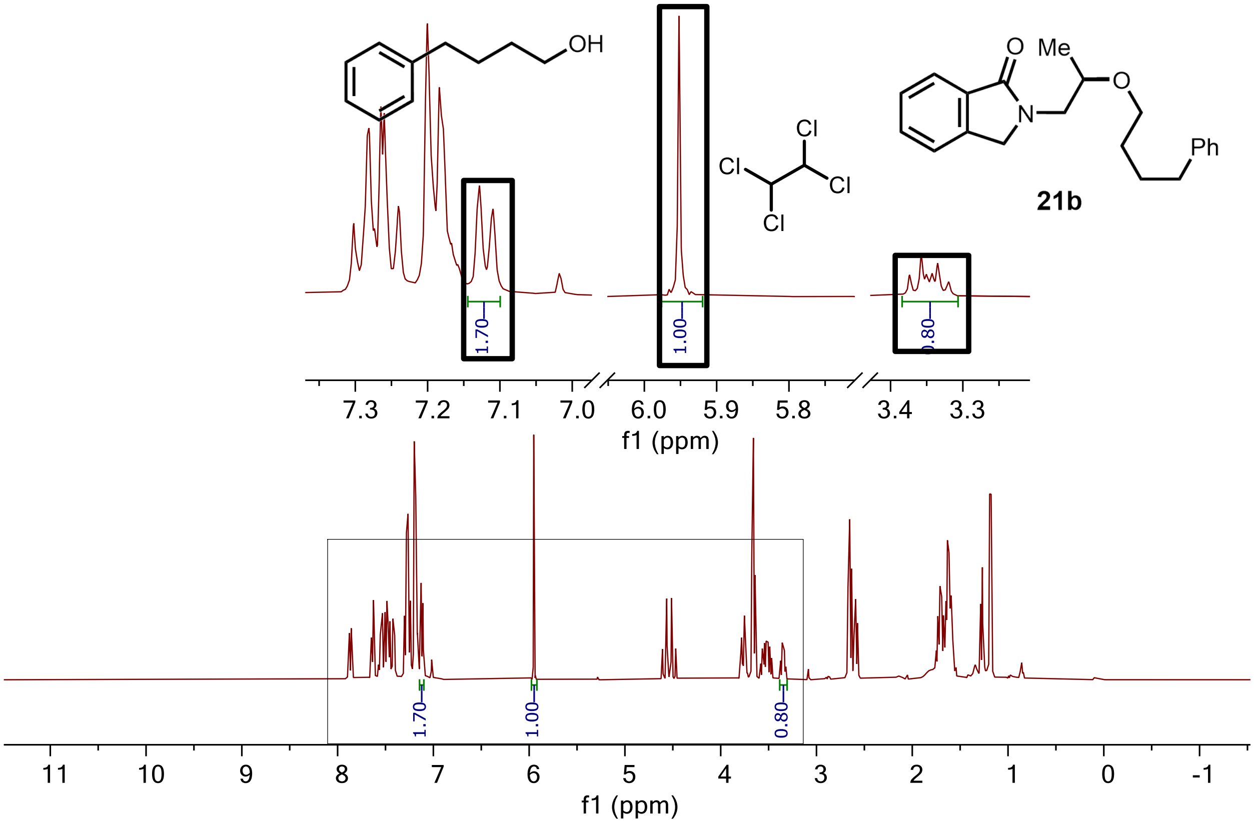

[a] Crude ^{1}H NMR was taken using 1,1,2,2-tetrachloroethane (0.05 mmol) as internal standard.

Figure SI-4 – Crude NMR of **21b**.

b) Product 25b, quantification of remaining alcohol starting material[a]

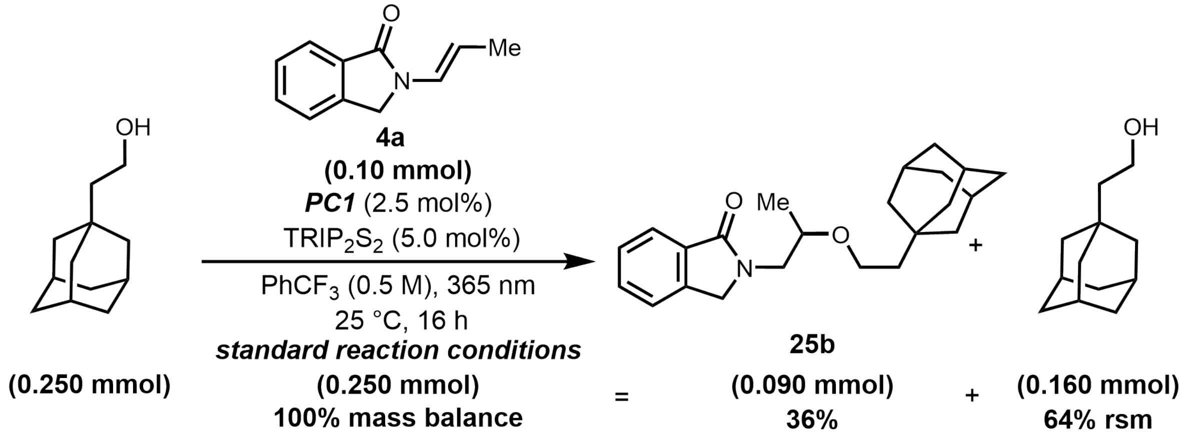

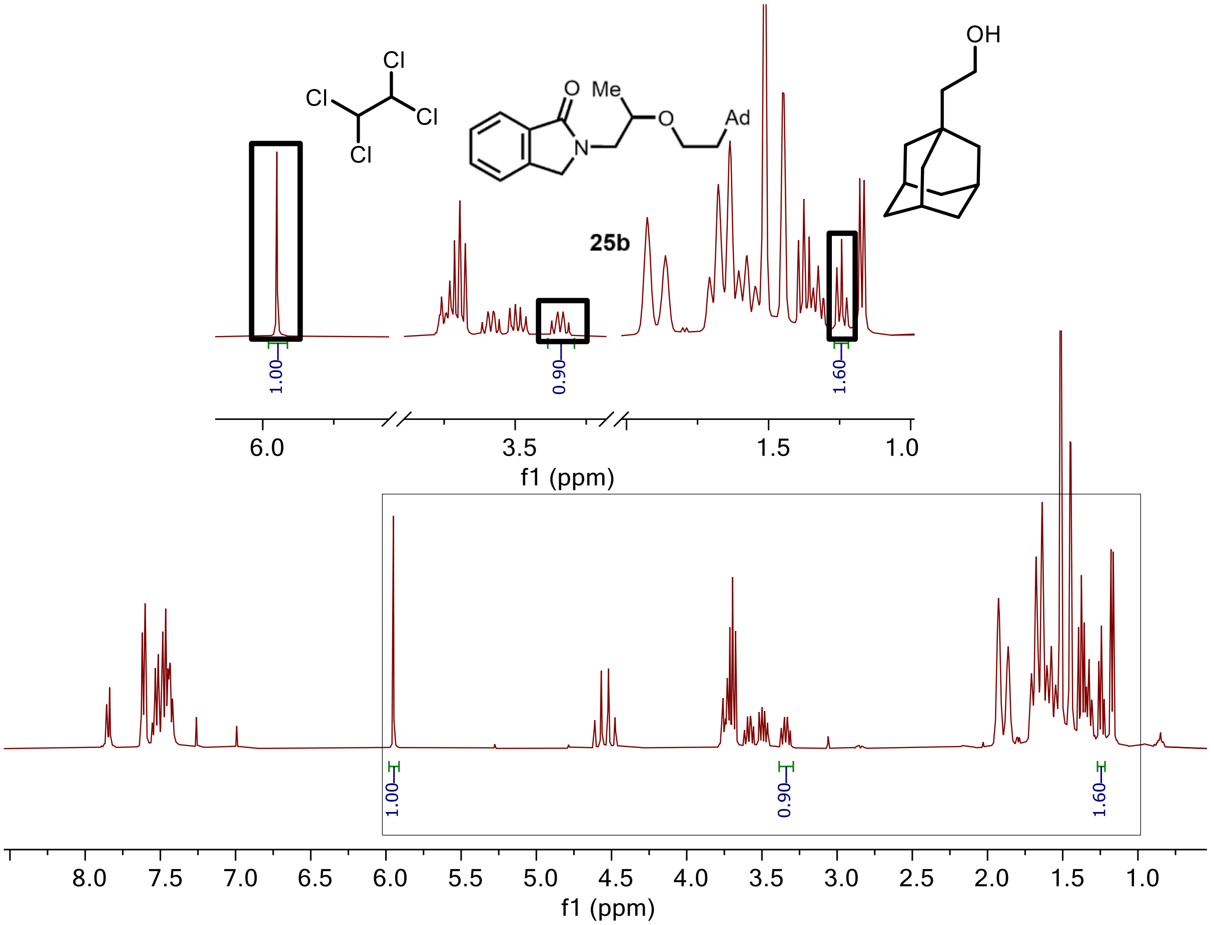

[a] Crude ^{1}H NMR was taken using 1,1,2,2-tetrachloroethane (0.05 mmol) as internal standard.

Figure SI-5 – Crude NMR of **25b**.

c) Product 28b, quantification of remaining alcohol starting material[a]

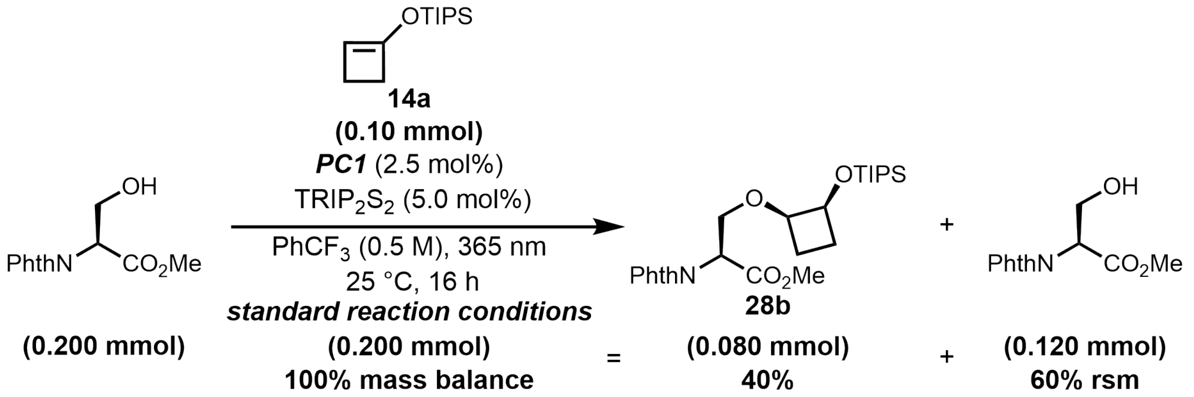

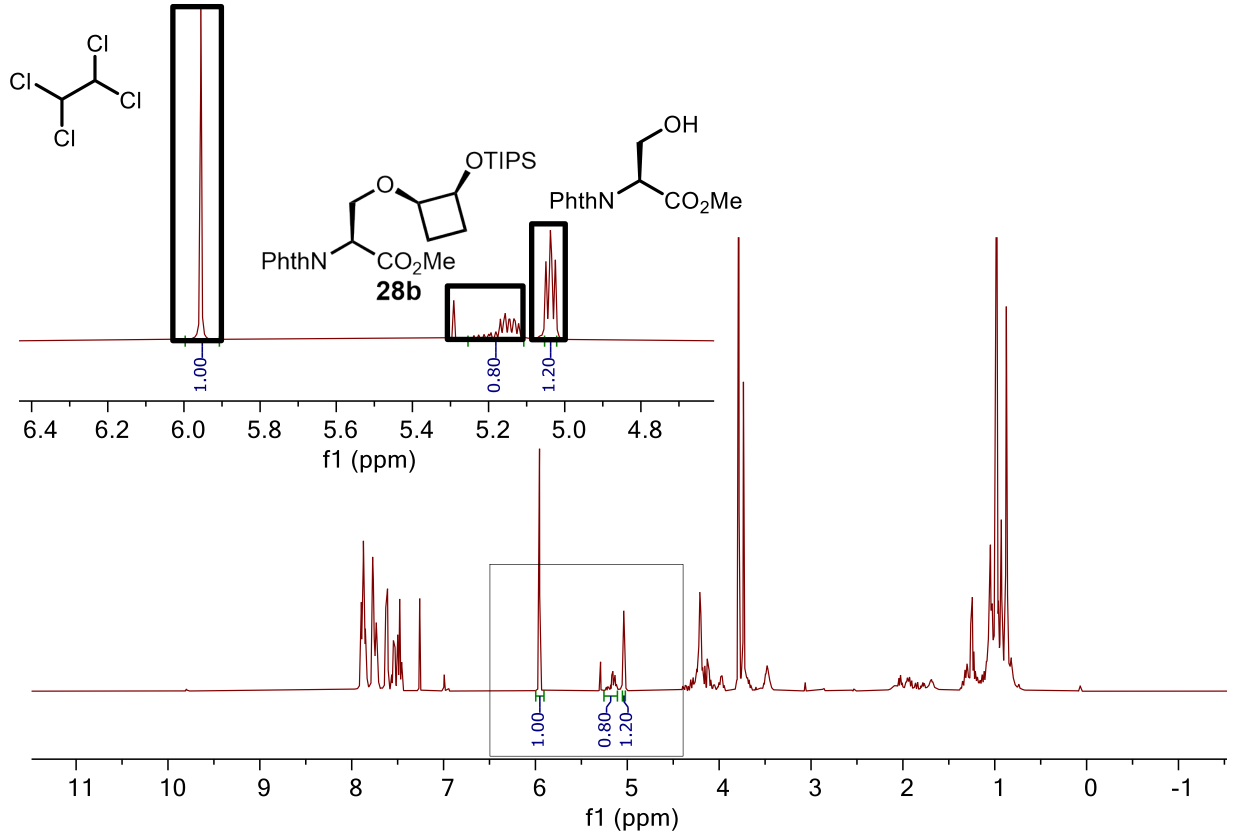

[a] crude ^{1}H NMR was taken using 1,1,2,2-tetrachloroethane (0.05 mmol) as internal standard.

Figure SI-6 – Crude NMR of **28b**.

d) Product 31b, quantification of remaining alcohol starting material[a]

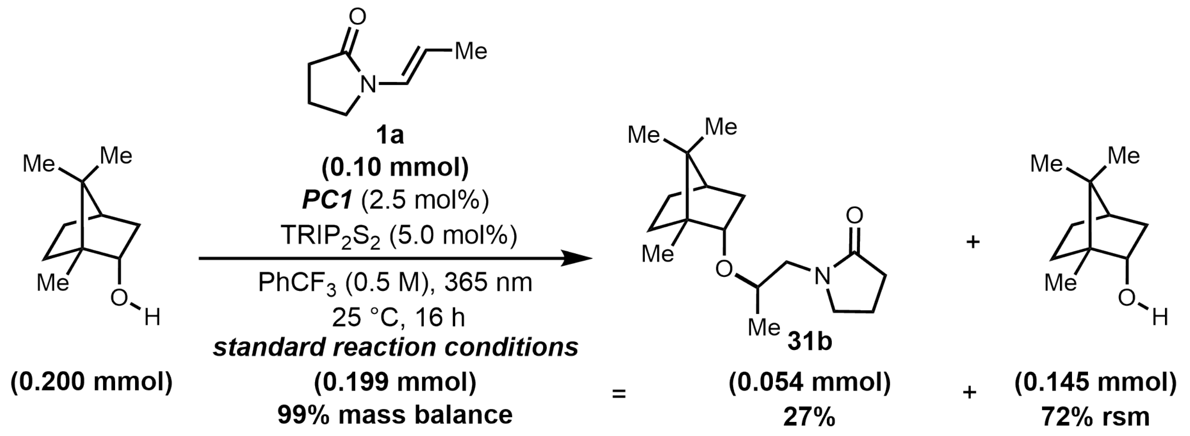

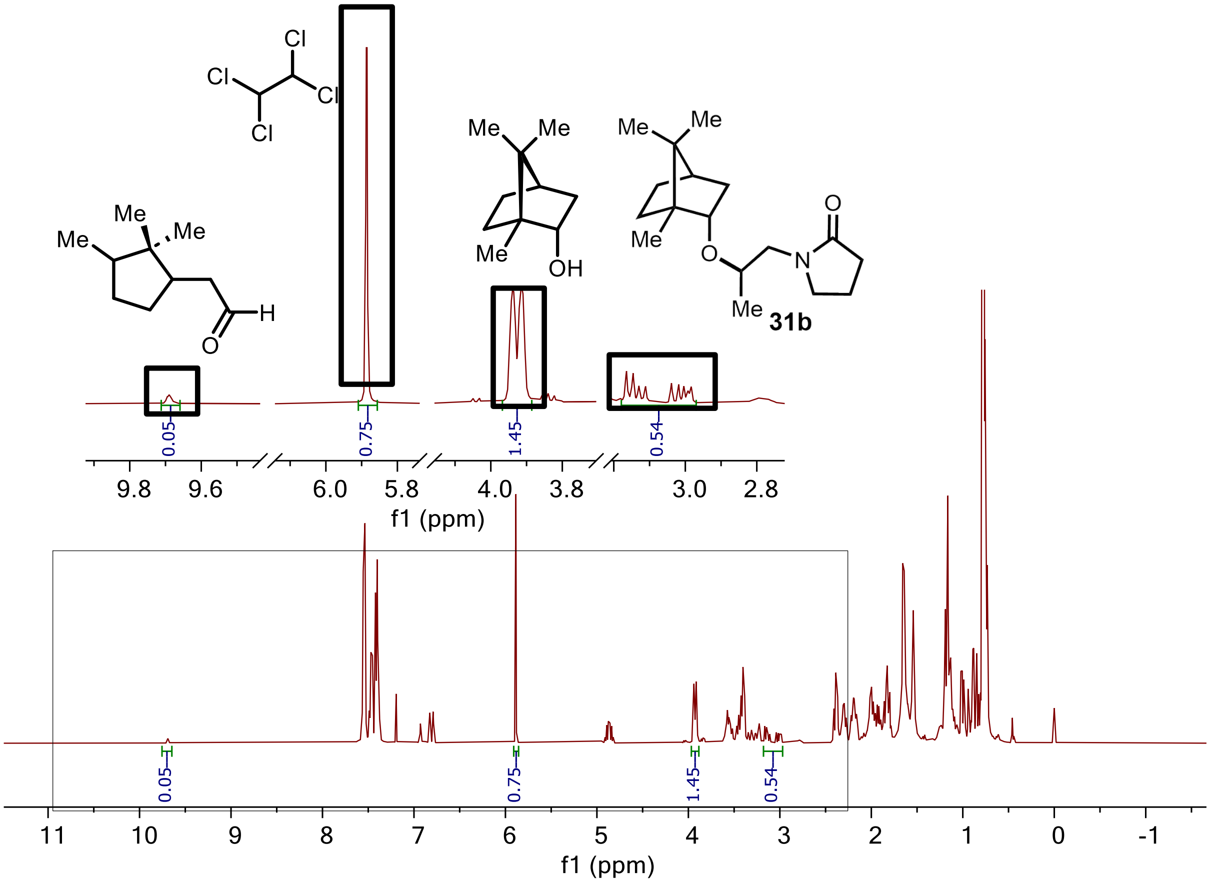

[a] Crude ^{1}H NMR was taken using 1,1,2,2-tetrachloroethane (0.0375 mmol) as internal standard.

Figure SI-7 – Crude NMR of **31b**.

e) Alkoxy Radical Scission Probe

Table SI-4 – crude NMR analysis of bornol scission experiments with **PC1, PC2, & PC3**

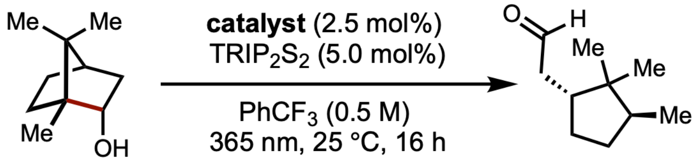

catalyst	*rsm*	*scission*
PC2	45%	45%
PC1	64%	22%
PC3	83%	6%

General information: A flame-dried 1 dram vial equipped with a magnetic stir bar was transferred to a nitrogen-filled glovebox. The vial was charged with (–)-borneol (0.1 mmol, 1.0 equiv) and **PC1, 2** or **3** (0.0025 mmol, 2.5 mol%). Following these additions, freeze-pump-thaw degassed anhydrous $PhCF_3$ (0.2 mL, 0.5 M) was added to the reaction vial, after which the vial was sealed with a threaded cap. The resulting solution was allowed to stir for 1 minute. The cap was then removed, and $TRIP_2S_2$ (0.005 mmol, 5 mol%) was quickly added to the vial as a solid in a single portion. The vial was sealed with a threaded cap, removed from the glovebox, and further reinforced with parafilm. The reaction vial was then transferred to a Hepatochem photoreactor attached to a recirculating chiller set to maintain a temperature of 25 °C. The reaction was set to stir under 365 nm light irradiation for 16 hours.

PC2 :

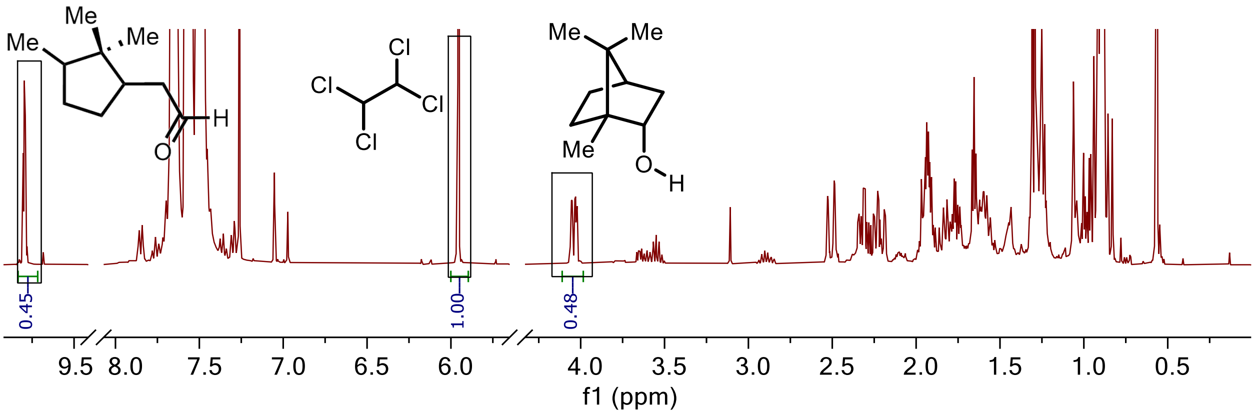

Figure SI-8 – Crude NMR of borneol scission with **PC2**

PC1 :

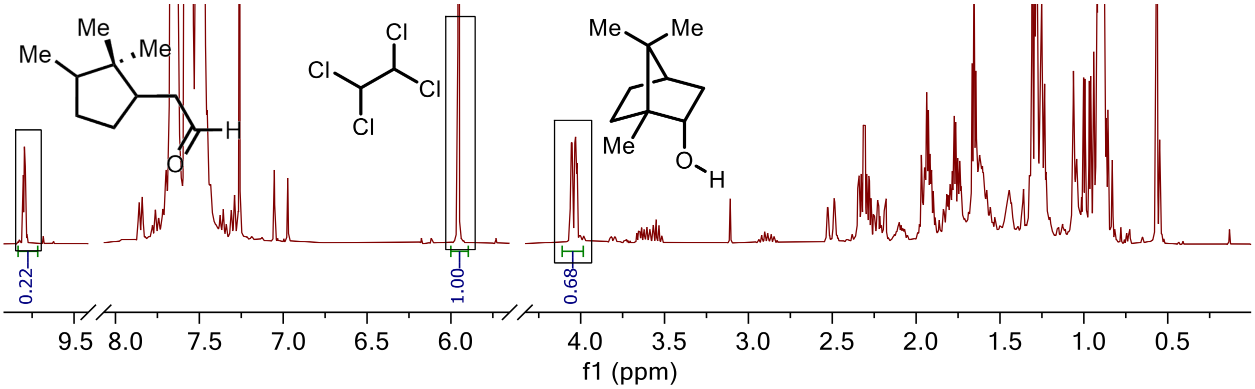

Figure SI-9 – Crude NMR of borneol scission with **PC1**

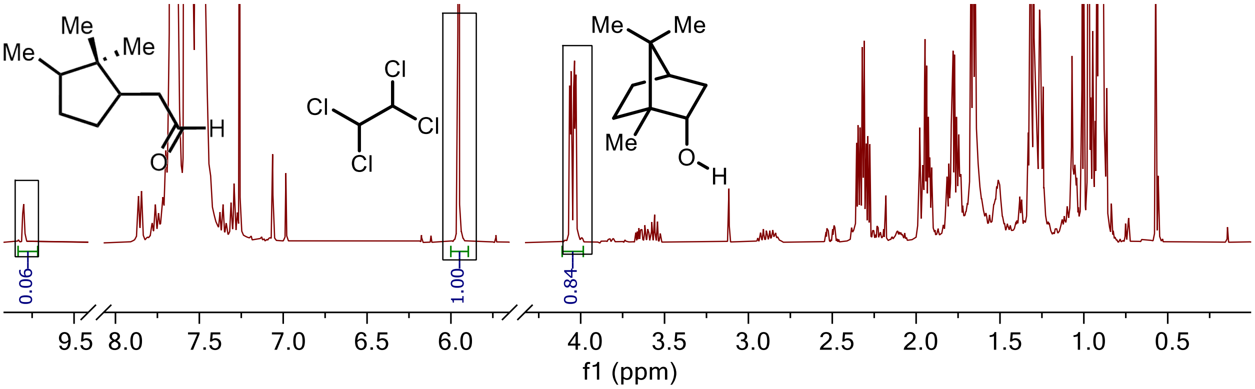

Figure SI-10 – Crude NMR of borneol scission with **PC3**

ii) Cyclic Voltammetry Measurements

General Procedure for acquiring cyclic voltammograms of selected alkenes:
To a flame-dried 25 mL three-neck round-bottomed flask equipped with a magnetic stir bar was added tetra-*n*-butylammonium hexafluorophosphate (387 mg, 1.0 mmol, 20.0 equiv), ferrocene (465 µg, 5 mol%) and analyte (0.05 mmol). The flask was sealed with three rubber septa, evacuated, and backfilled with nitrogen three times using a dual-manifold Schlenk line. Then degassed MeCN (10 mL) was added via syringe. The resulting mixture was stirred vigorously for 5 min before the septa were removed. A glassy carbon working electrode, a platinum wire counter electrode, and an $Ag/AgNO_3$ reference electrode were attached through the three necks of the flask. The electrodes were connected to a Pine Research WaveDriver 40 DC Bipotentiostat through steel alligator clips, and the stirring was stopped and the acquisition of the cyclic voltammogram was initiated.
Cyclic voltammogram acquisition was performed at a rate of 75 mV/s, starting at –200 mV, with an initial rising segment to +1800 mV, followed by a falling segment to −200 mV.
Results show an irreversible wave due to the reaction of the generated radical cation with another equivalent of alkene.[23–25] To estimate the upper bounds of the $E_{1/2}$ for the respective alkenes, the inflexion point was calculated via the first derivative of the cyclic voltammogram, assuming a symmetric wave. This results in an estimated $E_{1/2}$ of 1.02 V for **1a**, 1.08 V for **9a,** and 0.92 V for **15a**.

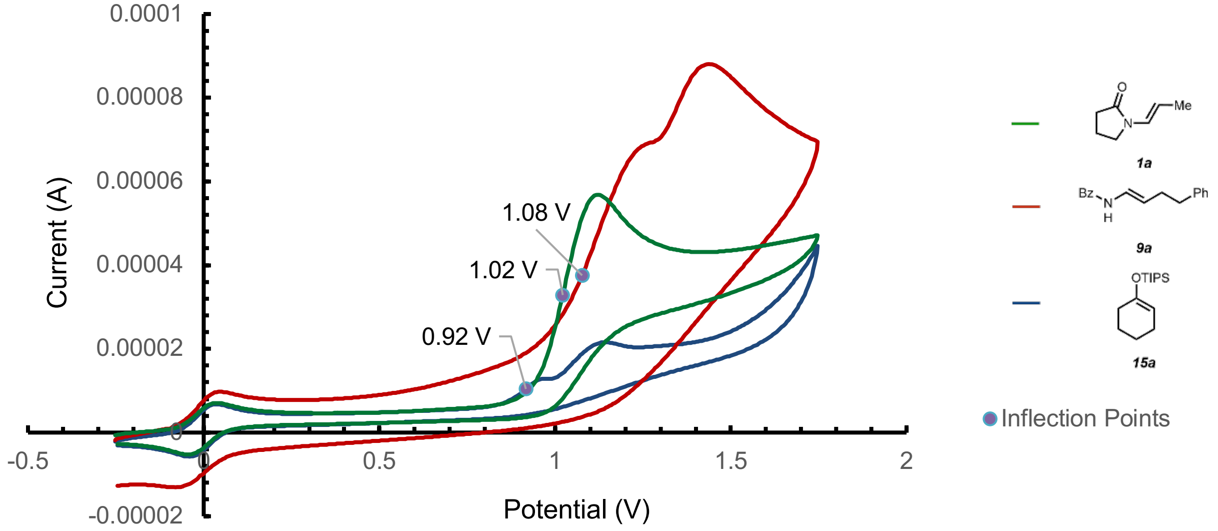

Figure SI-11 – Cyclic voltammograms of selected alkenes in V vs. F_c^+/F_c

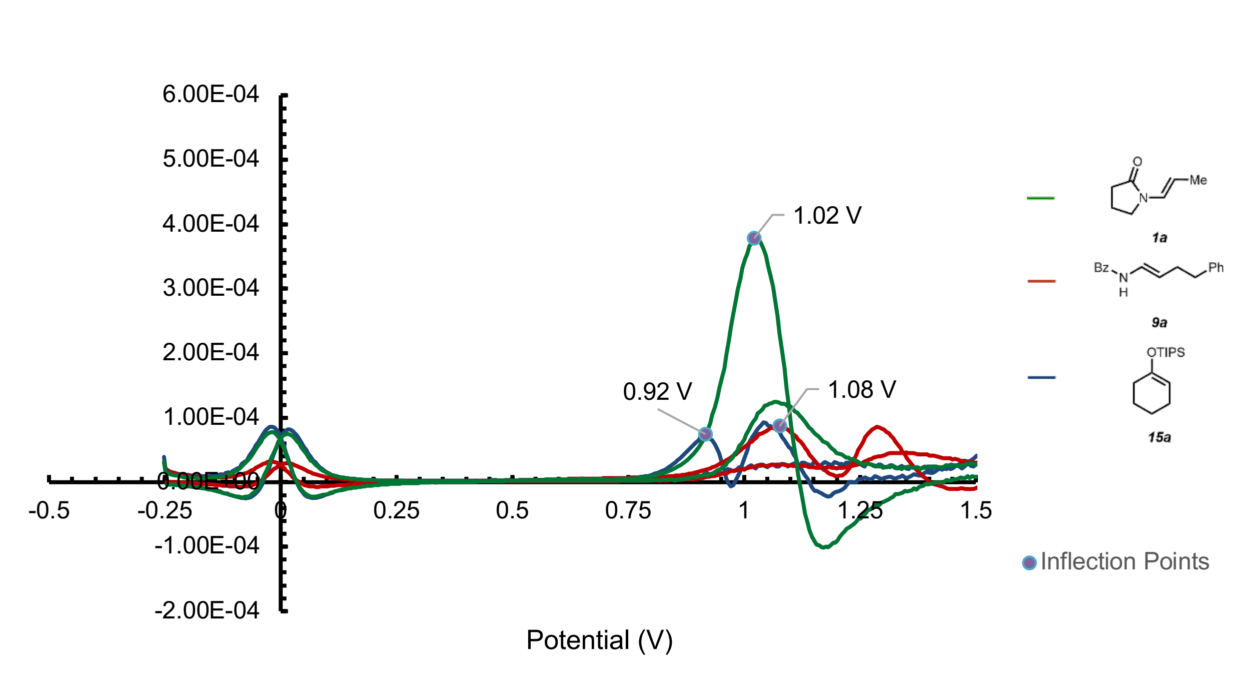

Figure SI-12 – First derivative of cyclic voltammograms of selected alkenes in V vs. F_c^+/F_c

General Procedure for acquiring cyclic voltammograms of xanthone derivatives:

To a flame-dried 25 mL three-neck round-bottomed flask equipped with a magnetic stir bar was added tetra-*n*-butylammonium hexafluorophosphate (387 mg, 1.0 mmol, 20.0 equiv), ferrocene (465 µg, 5 mol%) and analyte (0.05 mmol). The flask was sealed with three rubber septa, evacuated, and backfilled with nitrogen three times using a dual-manifold Schlenk line. Then degassed THF (10 mL) was added via syringe. The resulting mixture was stirred vigorously for 5 min before the septa were removed. A glassy carbon working electrode, a platinum wire counter electrode, and an $Ag/AgNO_3$ reference electrode were attached through the three necks of the flask. The electrodes were connected to a Pine Research WaveDriver 40 DC Bipotentiostat through steel alligator clips, and the stirring was stopped and the acquisition of the cyclic voltammogram was initiated.

Cyclic voltammogram acquisition was performed at a rate of 200 mV/s, starting at 500 mV, with an initial falling segment to -2000 mV, followed by a rising segment to 500 mV. Raw data was plotted after correction based on the $E_{1/2}$ of ferrocene.

The observed irreversible wave is likely due to rapid follow-up chemical reactivity of the electrogenerated xanthone radical anion (EC-type behavior), which prevents its re-oxidation on the reverse scan. To estimate the upper bounds of the $E_{1/2}$ for the respective xanthones, the inflexion point was calculated via the first derivative of the cyclic voltammogram, assuming a symmetric wave.

This results in an estimated $E_{1/2}$ = -1.93 V for **XO2**, of -1.69 V for **S9**, and -1.84 V for **S10**.

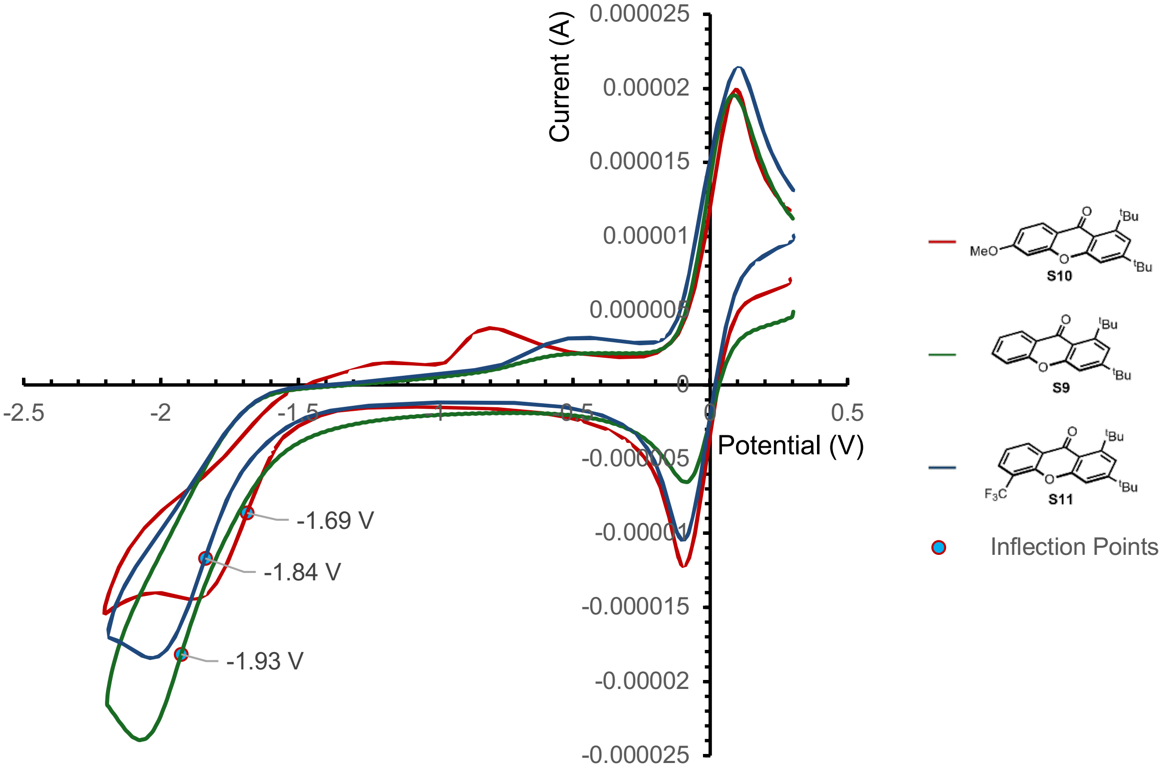

Figure SI-13 – Cyclic voltammograms of xanthone derivatives in V vs. F_c^+/F_c

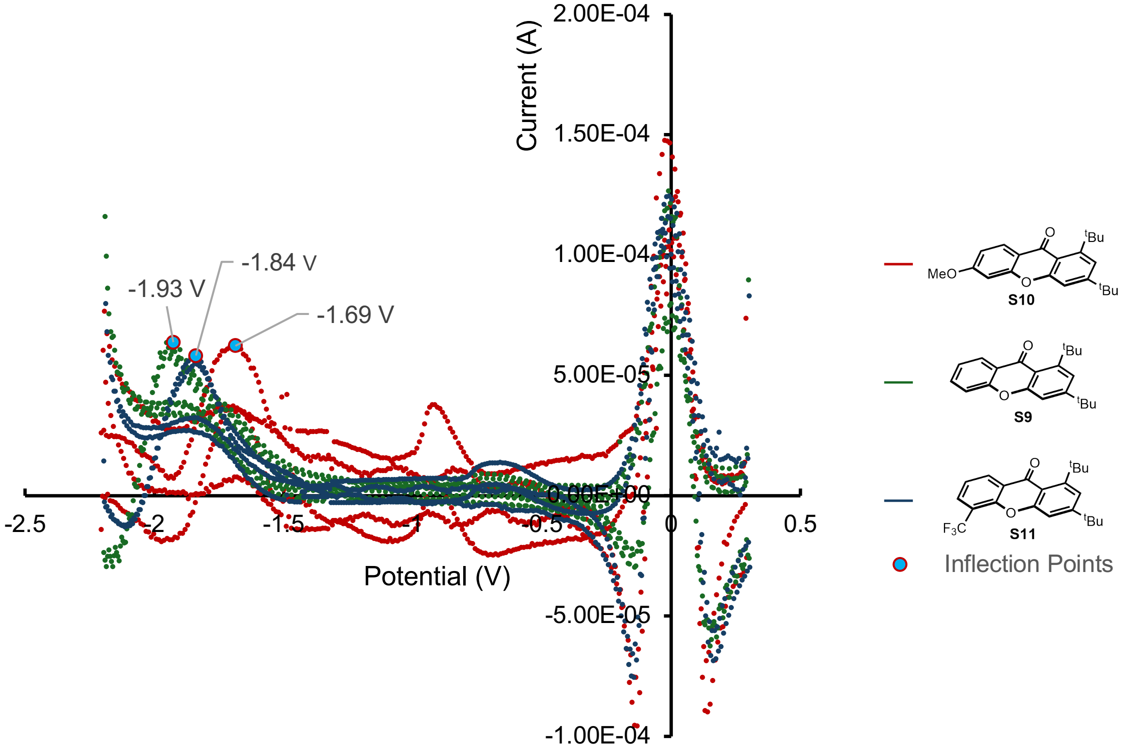

Figure SI-14 – First derivative of cyclic voltammograms of xanthone derivatives in V vs. F_c^+/F_c

iii) Luminescence Experiments

Luminescence Titration Experiment

General Information:

The luminescence measurements were carried out using a Varian Cary Eclipse Fluorescence Spectrophotometer and a 10 mm cuvette (VWR, Cat. No.: 414004-064, VWR Cell Fluoro Flat 10 mm). The luminescence was measured utilizing fluorescence mode (*Scan* program provided by Varian Cary) with an excitation wavelength at 365 nm, 10.0 nm excitation slit width, and 10.0 nm emission slit width. $PhCF_3$, MeCN, MeOH, EtOH, 2,2,2-trifluoroethanol (TFE) and ethylene glycol used in this study were degassed through a freeze-pump-thaw cycle.

Preparation of quencher stock solutions:

Inside a nitrogen-filled glovebox, a 10 mL scintillation vial was charged with **quencher** (200.0 μmol) and $PhCF_3$ (200.0 μL). The resulting solution was shaken to ensure full homogeneity to yield a stock solution (1.0 M) of quencher.

Preparation of photocatalyst stock solution:

Inside a nitrogen-filled glovebox, a 2 mL scintillation vial was charged with ***PC*** (5.0 μmol) and $PhCF_3$ (200.0 μL). The resulting solution was shaken to ensure full homogeneity to yield a stock solution (25 mM) of the photocatalyst.

Preparation of quenching samples:

Into a 10 mm cuvette, ***PC*** stock solution (40 μL) and $PhCF_3$ (3.4 mL) were added. The resulting solution was shaken to ensure full homogeneity. Then, the cuvette was placed inside the fluorescence spectrophotometer. The fluorescence was measured. This process was repeated in its entirety with increasing amounts of quencher stock solution added (10, 20, 30, 40 μL) to make (2.5, 5.0, 7.5, 10.0mM) solutions of quencher, respectively.

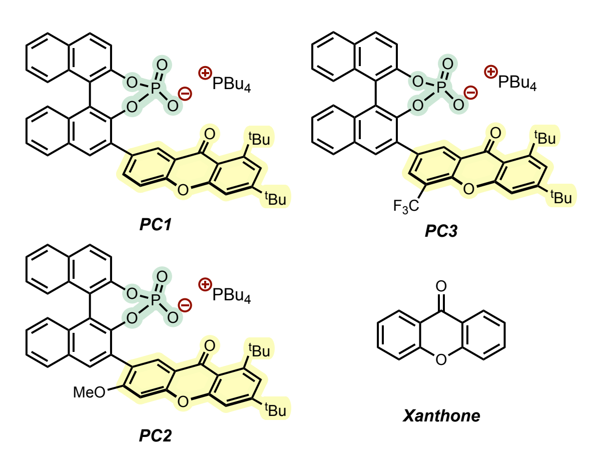

Catalysts used in this study

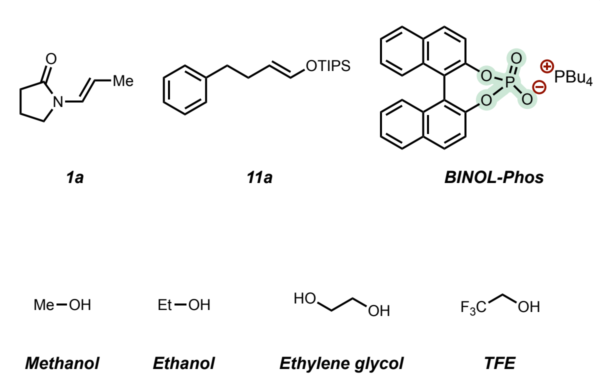

Substrates used in this study

Luminescence Comparison

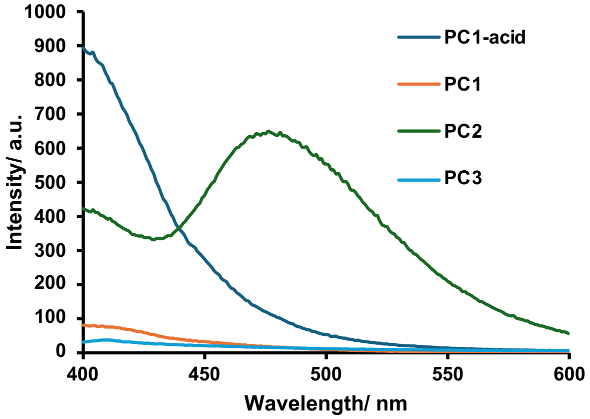

Figure SI-15 – Fluorescence emission spectra of photocatalysts

Determination of excited state redox potential

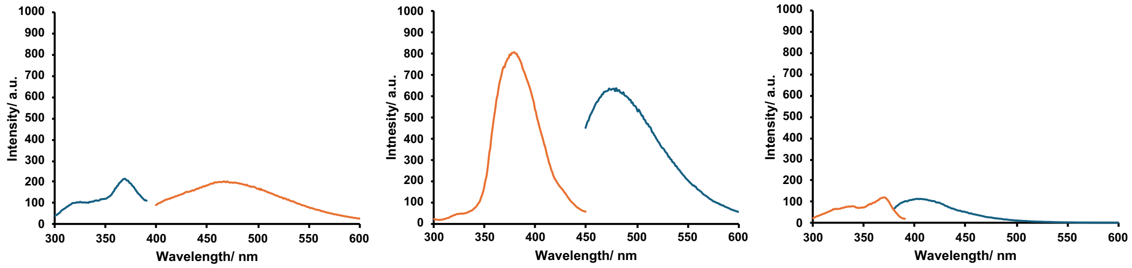

S9$_{0\text{–}0}$: 390 nm
E_{red}^{*} = 3.18-1.93 = 1.25 V

S10$_{0\text{–}0}$: 427 nm
E_{red}^{*} = 2.90-1.69 = 1.21 V

S11$_{0\text{–}0}$: 379 nm
E_{red}^{*} = 3.27-1.84 = 1.43 V

Figure SI-16 – Overlap of fluorescence emission and absorption spectra of photocatalysts

The 0–0 energy gap of **S9** and **S11** was calculated using the intersection point of the fluorescence excitation and emission spectra; The 0–0 energy gap of **S10** was calculated using the average of λ_{max} of the fluorescence excitation and emission spectra. We estimated the excited state redox potential of the catalyst using the excited redox potential of the xanthones.

Fluorescence Recovery of *PC1*: (tabulated as I/I_0)

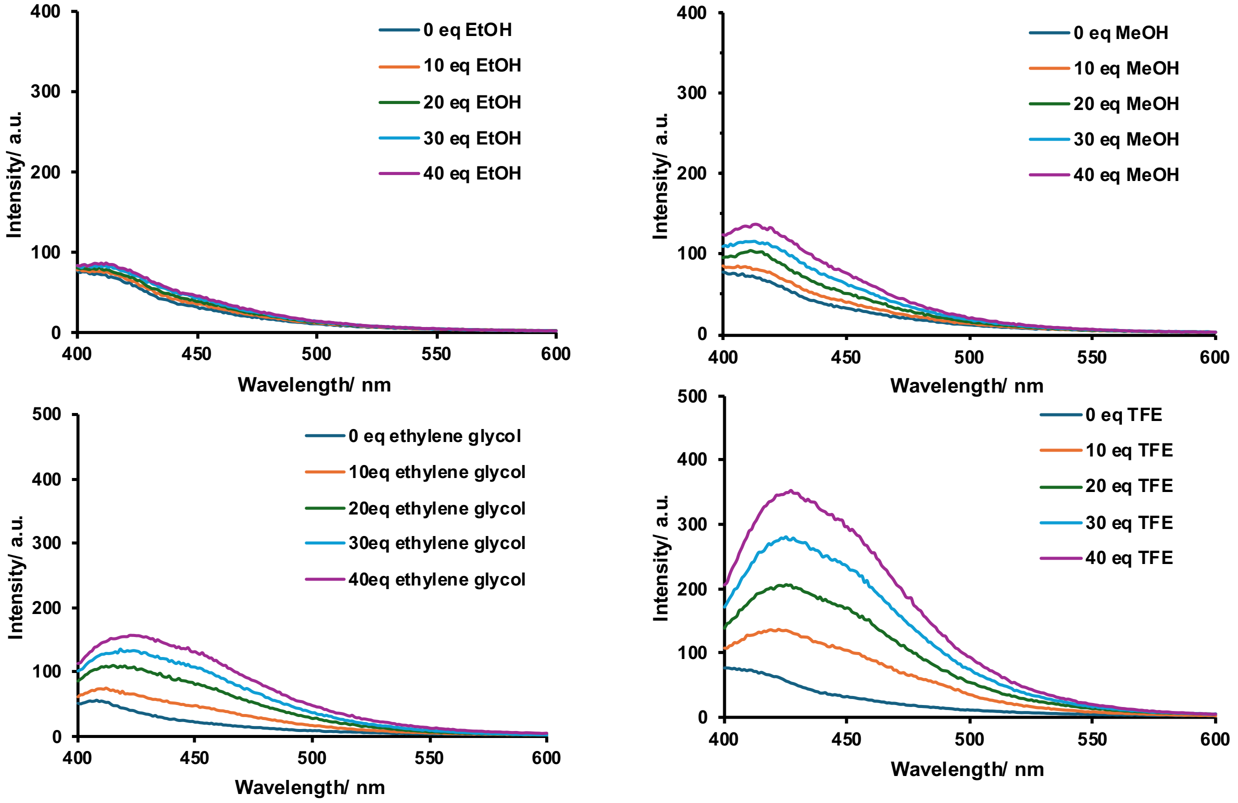

Figure SI-16 – Fluorescence spectra of ***PC1*** titration

Table SI-5 – Emission intensity of ***PC1*** at 425nm

	EtOH		MeOH		Ethylene glycol		TFE	
[Q]/[F]	Intensity/a.u.	*I/I o*	Intensity/a.u.	*I/I o*	Intensity/a.u.	*I/I o*	Intensity/a.u.	*I/I o*
0	57.0717	1.0000	57.2875	1.0000	37.7820	1.0000	55.8907	1.0000
10	61.0820	1.0703	68.0391	1.1877	64.6359	1.7108	134.4267	2.4052
20	64.7448	1.1344	86.2959	1.5064	104.5335	2.7668	205.7505	3.6813
30	70.4508	1.2344	104.1321	1.8177	132.4921	3.5068	279.8449	5.0070
40	73.3653	1.2855	120.9100	2.1106	155.9666	4.1281	349.3234	6.2501

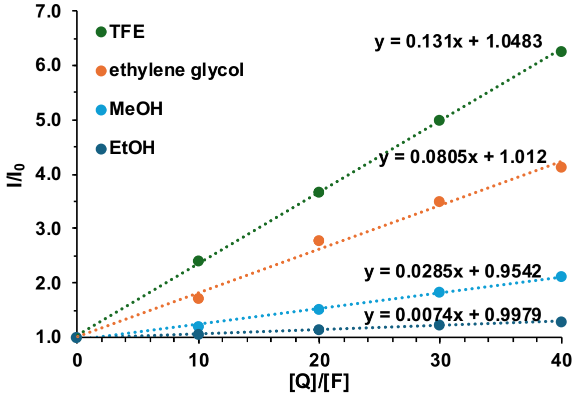

Fluorescence Recovery of ***PC2***: (tabulated as I/I_0)

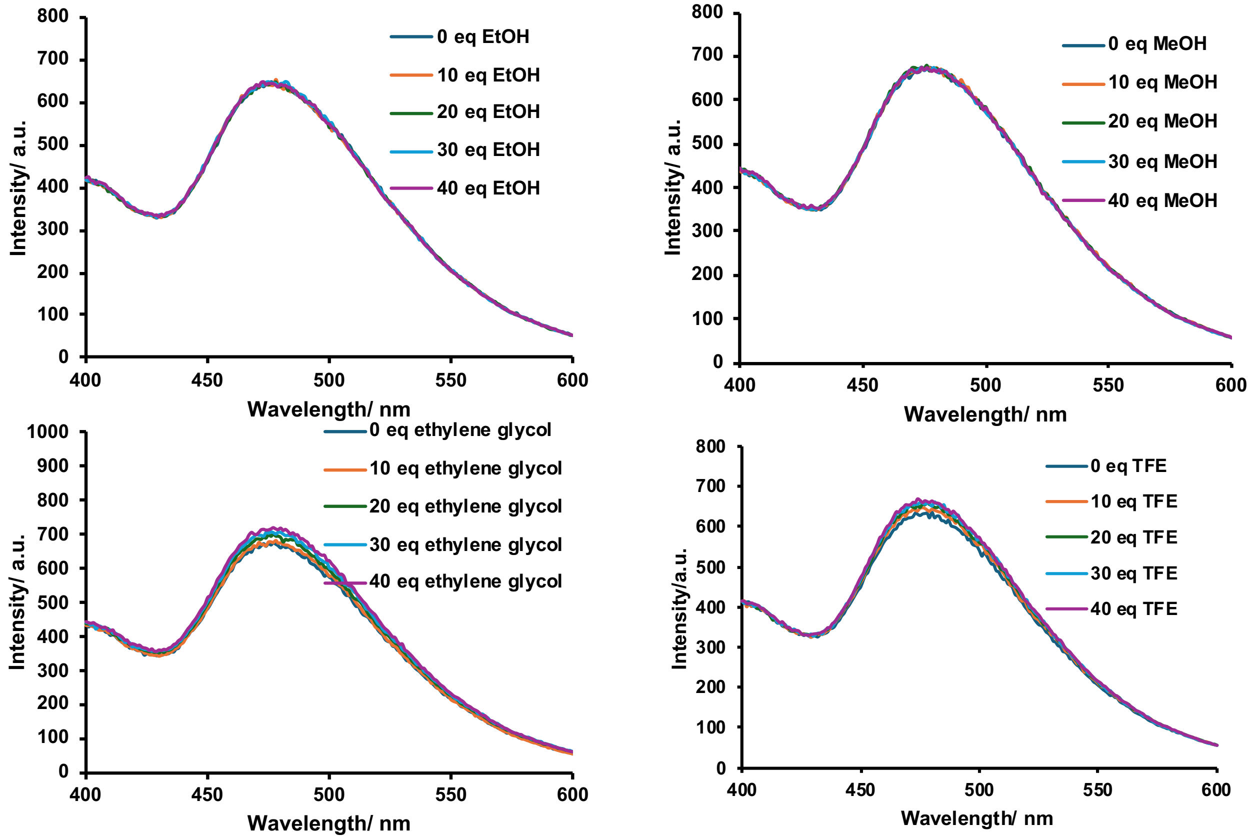

Figure SI-17 – Fluorescence spectra of ***PC2*** titration

Table SI-6 – Emission intensity of ***PC2*** at 475nm

	EtOH		MeOH		Ethylene glycol		TFE	
[Q]/[F]	Intensity/a.u.	*I/I₀*	Intensity/a.u.	*I/I₀*	Intensity/a.u.	*I/I₀*	Intensity/a.u.	*I/I₀*
0	642.9036	1.0000	670.9684	1.0000	675.2126	1.0000	629.6196	1.0000
10	642.4658	0.9993	671.6296	1.0010	679.8997	1.0069	646.3456	1.0266
20	642.7898	0.9998	669.5065	0.9978	698.5272	1.0345	649.0309	1.0308
30	642.5062	0.9994	667.8722	0.9954	704.6359	1.0436	661.8403	1.0512
40	641.8298	0.9983	668.4191	0.9962	710.4479	1.0522	667.4414	1.0601

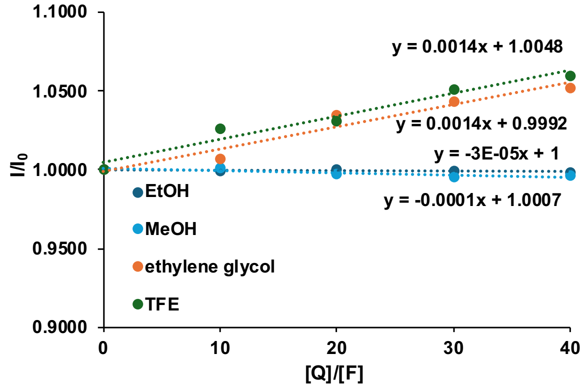

Fluorescence Recovery of ***PC3*** (tabulated as I/I_0):

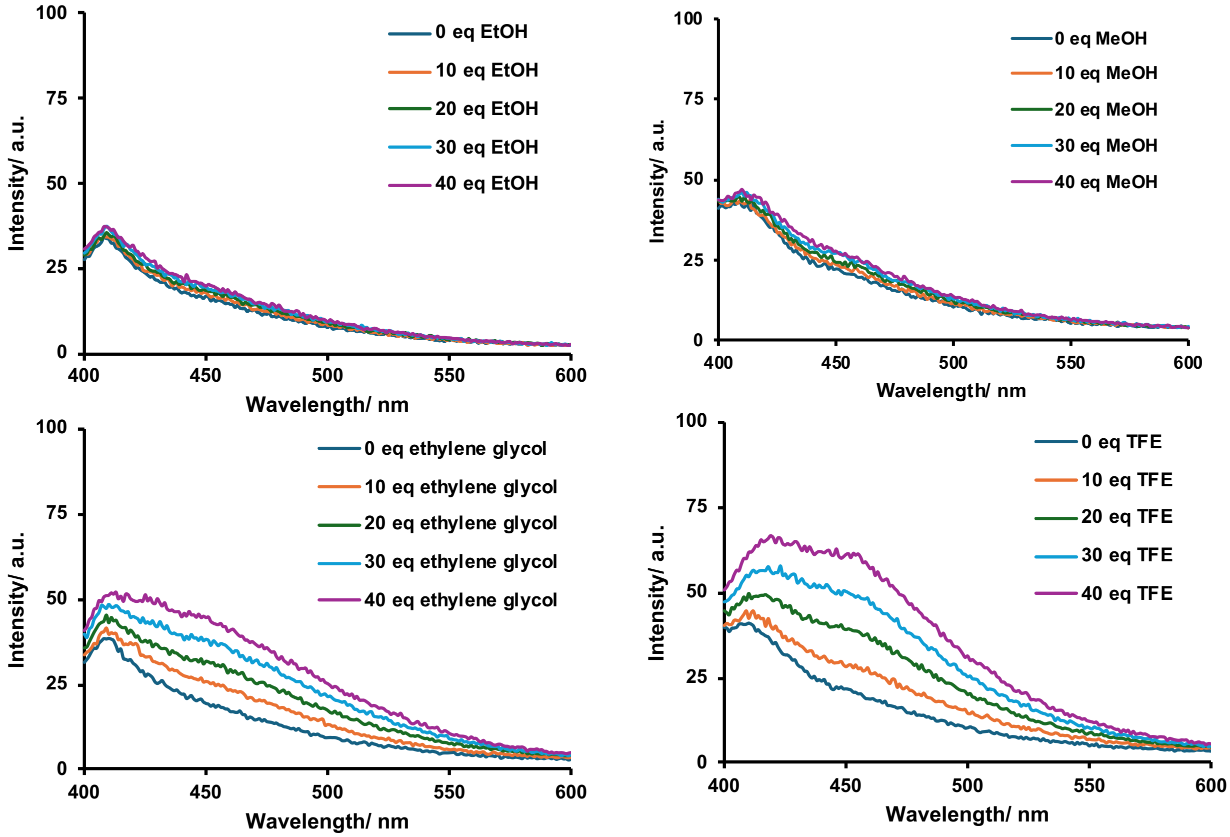

Figure SI-18 – Fluorescence spectra of ***PC3*** titration

Table SI-7 – Emission intensity of ***PC3*** at 409nm

	EtOH		MeOH		Ethylene glycol		TFE	
[Q]/[F]	Intensity/a.u.	I/I_0	Intensity/a.u.	I/I_0	Intensity/a.u.	I/I_0	Intensity/a.u.	I/I_0
0	33.9899	1.0000	42.9251	1.0000	38.5493	1.0000	40.4446	1.0000
10	34.3387	1.0103	43.6386	1.0166	41.1885	1.0685	44.4396	1.0988
20	35.7696	1.0524	44.4806	1.0362	45.4050	1.1778	47.9323	1.1851
30	37.5381	1.1044	45.5709	1.0616	47.0384	1.2202	55.2621	1.3664
40	37.2437	1.0957	46.2904	1.0784	51.0731	1.3249	59.7455	1.4772

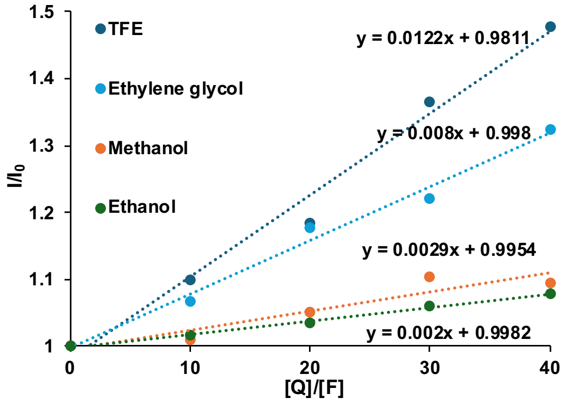

Fluorescence titration with added MeOH while varying the amount of substrates (tabulated as I_0/I):

Table SI-8 – Emission intensity of ***PC1*** at 425nm, quencher = **1a**

	0 eq MeOH		40 eq MeOH		200 eq MeOH		300 eq MeOH	
[Q]/[F]	Intensity/a.u.	I_0/I	Intensity/a.u.	I_0/I	Intensity/a.u.	I_0/I	Intensity/a.u.	I_0/I
0	79.6793	1.0000	85.3792	1.0000	380.0982	1.0000	417.5241	1.0000
10	104.6130	0.7617	99.6065	0.8572	382.4037	0.9940	416.0002	1.0037
20	138.8619	0.5738	111.0923	0.7685	386.7926	0.9827	417.5502	0.9999
30	178.3407	0.4468	121.2716	0.7040	379.7374	1.0010	417.4725	1.0001
40	206.8661	0.3852	132.5499	0.6441	381.6055	0.9961	417.5905	0.9998

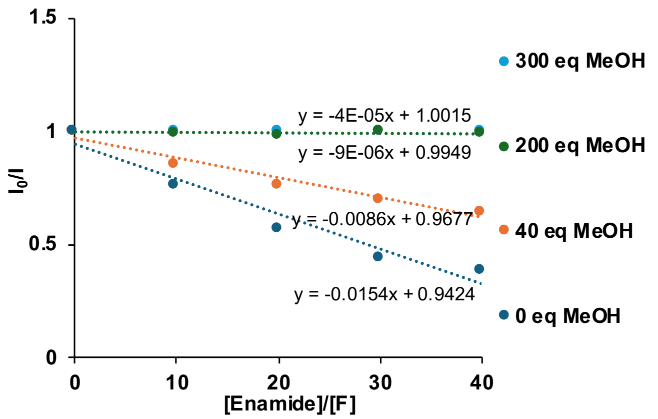

Table SI-9 – Emission intensity of ***PC1*** at 425nm, quencher = **11a**

	0 eq MeOH		40 eq MeOH		100 eq MeOH		200 eq MeOH	
[Q]/[F]	Intensity/a.u.	I_0/I	Intensity/a.u.	I_0/I	Intensity/a.u.	I_0/I	Intensity/a.u.	I_0/I
0	53.2739	1.0000	126.9873	1.0000	132.6406	1.0000	356.3448	1.0000
10	55.3510	0.9625	126.1566	1.0066	130.7691	1.0143	347.7593	1.0247
20	57.9582	0.9192	124.3509	1.0212	128.0049	1.0362	335.3328	1.0627
30	59.9403	0.8888	125.1918	1.0143	126.1847	1.0512	319.8390	1.1141
40	60.0138	0.8877	124.3704	1.0210	124.9333	1.0617	304.3407	1.1709

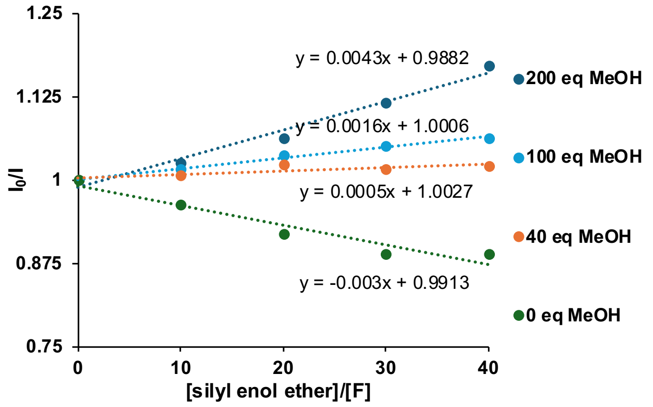

Explanation for fluorescence recovery:

We assume the adduct (A) formation requires a 1:1 stoichiometry of the fluorophore (F) and quencher (Q).

$$F + Q \rightleftharpoons A$$

Thus, the thermodynamic binding constant (K_a) can be expressed as a function of adduct concentration [A], uncomplexed fluorophore $[F]_u$ and quencher $[Q]_u$.

$$K_a = \frac{[A]}{[F]_u \times [Q]_u}$$

Thus,

$$[F]_u = [F]_t - [A]$$

$[F]_t$ is the total fluorophore concentration. Since total quencher concentration $[Q]_t$ >> [A] in the solution, we could assume that:

$$[Q]_u \approx [Q]_t$$

Then the fluorescence intensity is proportion to the emission intensity of uncomplexed fluorophore and adduct.

$$I = \varphi_F[F]_u + \varphi_A[A] = \varphi_F([F]_t - [A]) + \varphi_A[A] = \varphi_F[F]_t + (\varphi_A - \varphi_F)[A]$$

φ_F and φ_A are proportion to the emission intensity.

Then the initial intensity:

$$I_0 = \varphi_F[F]_t$$

Thus:

$$\frac{I}{I_0} = \frac{\varphi_F[F]_t + (\varphi_A - \varphi_F)[A]}{\varphi_F[F]_t} = 1 + \frac{\varphi_A - \varphi_F}{\varphi_F} \times \frac{[A]}{[F]_t} = 1 + \frac{\varphi_A - \varphi_F}{\varphi_F} \times \frac{K_a\,[F]_u[Q]_u}{[F]_t}$$

The fluorescence enhancement slope we observed is a function of K_a and $\frac{\varphi_A}{\varphi_F}$.

In the case of ***PC1*** and ***PC3***, any interaction that forms an adduct that has $\varphi_A > \varphi_F$:

$$= 1 + \left(\frac{\varphi_A - \varphi_F}{\varphi_F}\right)\frac{K_a[F]_u}{[F]_t}[Q]_t = 1 + \left(\frac{\varphi_A - \varphi_F}{\varphi_F}\right)\frac{K_a[Q]_t}{K_a[Q]_t + 1} > 1$$

We would observe fluorescence enhancement.

Fluorescence titration of XO:
With phosphate:

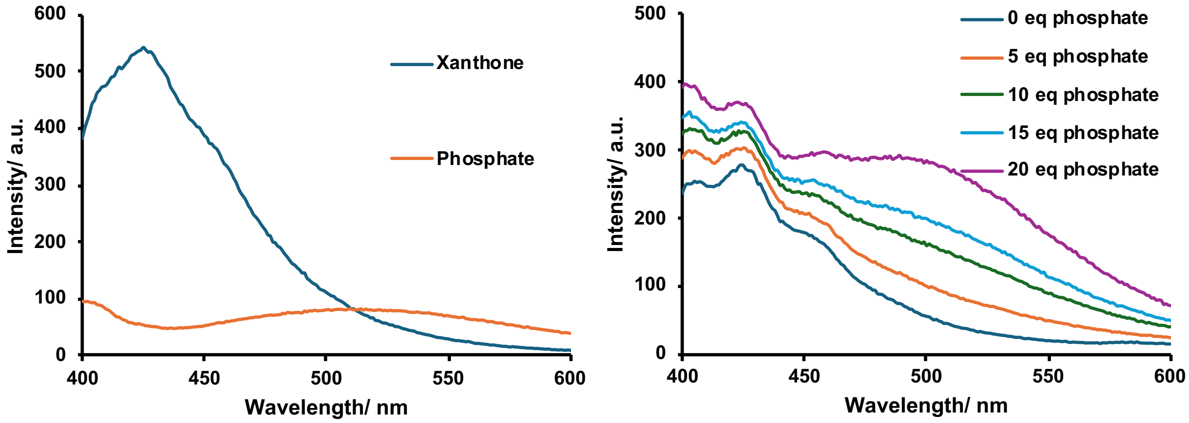

Figure SI-19 – Fluorescence spectra of **XO** with **BINOL-Phos**

The fluorescence spectra of **XO** and **BINOL-Phos** overlap at 400nm-450nm, causing interference between the two signals, making it challenging to obtain a reliable quenching signal.

With Methanol (tabulated as I/I_0):

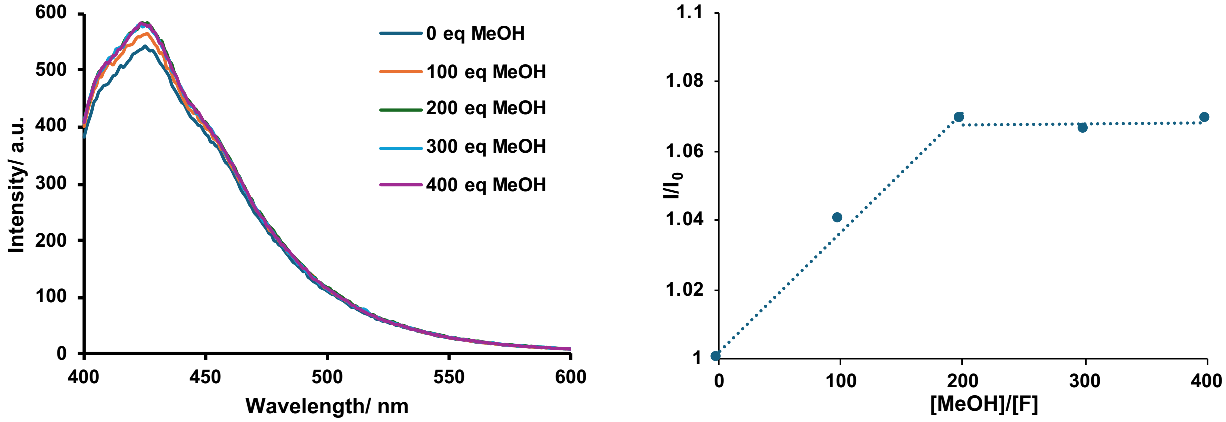

Figure SI-20 – Fluorescence spectra of **XO** with MeOH

With Methanol and added 1.0 equiv of phosphate (tabulated as I_0/I):

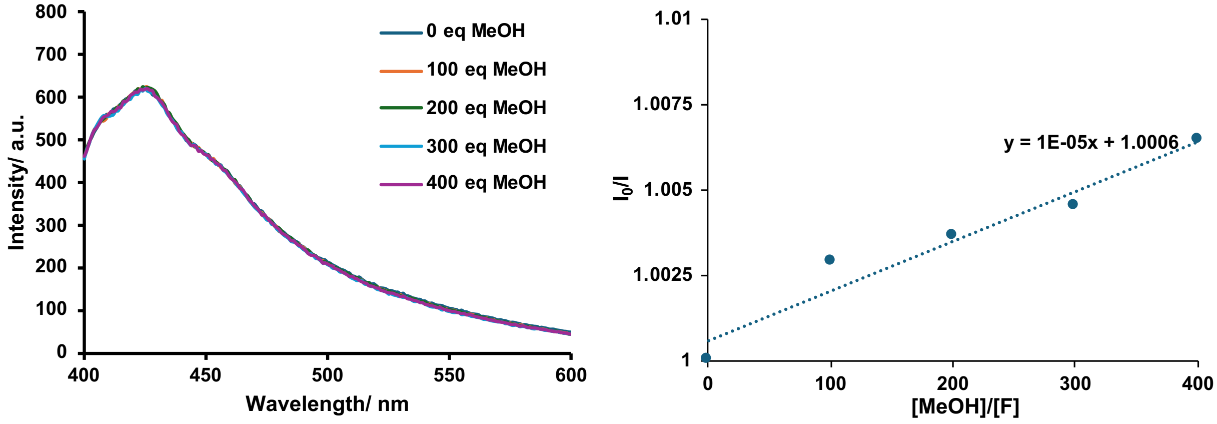

Figure SI-21 – Fluorescence spectra of **XO** with MeOH and **BINOL-Phos**

Slight fluorescence quenching was observed.

With 1a (tabulated as I/I_0):

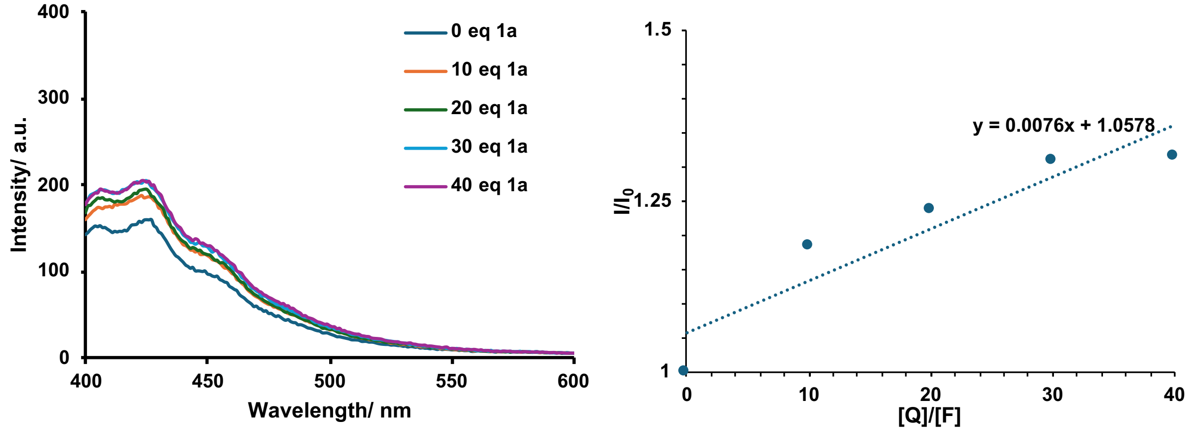

Figure SI-22 – Fluorescence spectra of **XO** with **1a**

With 11a (tabulated as I/I_0):

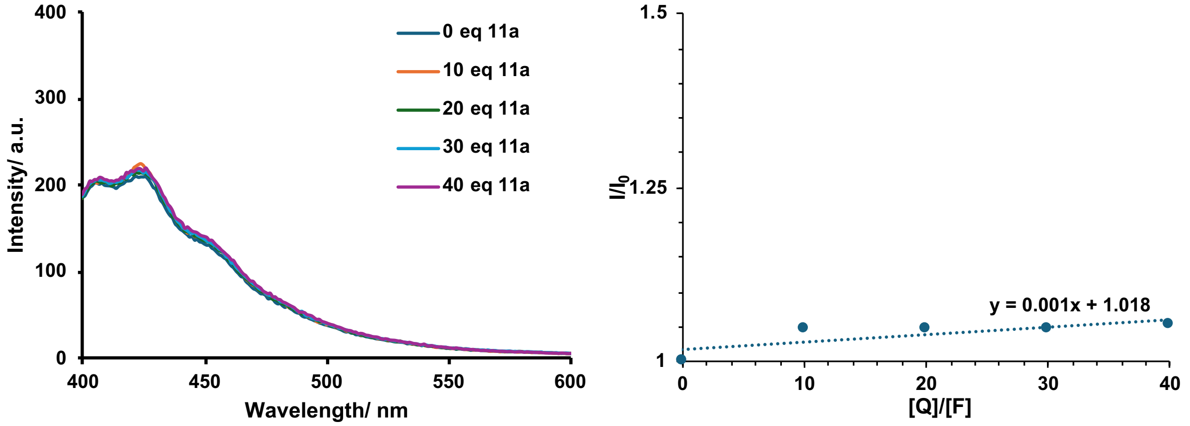

Figure SI-23 – Fluorescence spectra of **XO** with **11a**

Slight fluorescence enhancement was observed.

iv) Competition Experiments

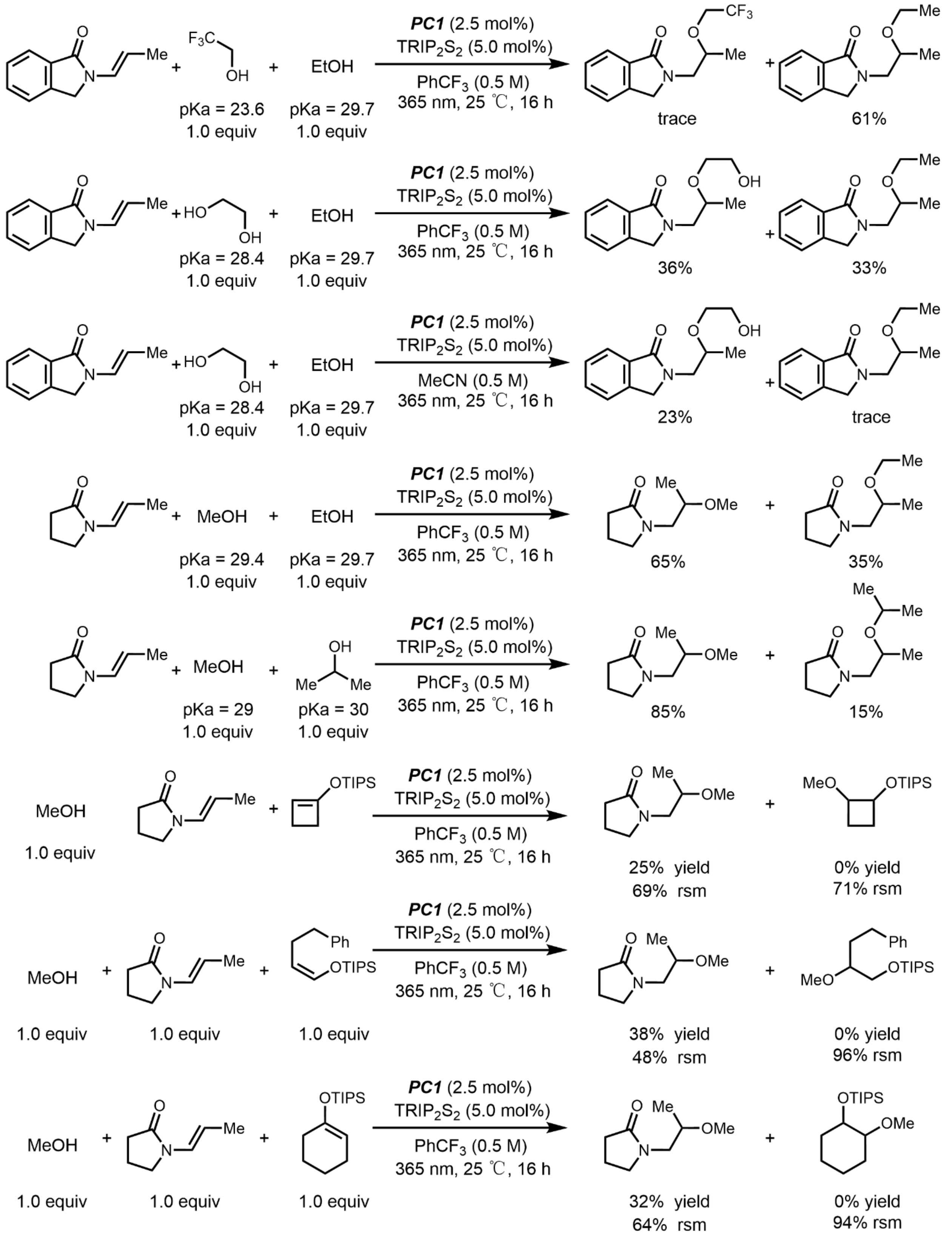

Figure SI-24 – Competition experiments

v) Comparison Experiments

Comparison experiment was conducted with selected substrates to gain mechanistic insight. Condition A, and B were based on the condition from reported literature, intended to represent PCET[26] and LMCT[11] mechanism respectively. Yield was assessed by ^{1}H-NMR yields of the crude mixture using 1,1,2,2-tetrachloroethane as an internal standard.

Table SI-10 – Comparison with known hydroetherification conditions

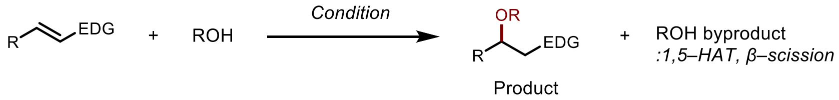

Our method	Condition A	Condition B
**PC1** (2.5 mol%)<br>$TRIP_2S_2$ (5.0 mol%)<br>TFT (0.5 M)<br>365 nm, 25 °C, 24 h	$[Ir(dF(CF_3)ppy)_2(5,5'\text{-}d(CF_3)bpy)]PF_6$ (2 mol%)<br>$Bu_4P^+(PhO)_2P(O)O^-$ (20 mol%)<br>bis(2-fluorophenyl)disulfide (30 mol%)<br>TFT (0.1 M), 440 nm, 48 h	$(Bu_4N)_2CeCl_6$ (5 mol%)<br>2,4,6-(^{i}Pr)$_3$PhCO$_2^-$K$^+$ (10 mol%)<br>$TRIP_2S_2$ (10 mol%), DPA (5 mol%)<br>MeCN (0.05 M), 390 nm, 16 h

Substrate	Condition	ROH (n equiv)	SM retention	Product	ROH retention	ROH byproduct	Mass balance for ROH
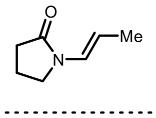	**Our method**	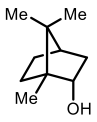 2.0 equiv	-	54%	145%	5%	177%
	**Condition A**		-	67%	82%	50%	199%
	**Condition B**		88%	-	35%	163%	198%
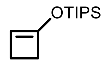	**Our method**	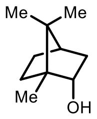 2.0 equiv	36%	45%	143%	5%	193%
	**Condition A**		-	43%	87%	63%	193%
	**Condition B**		35%	-	11%	172%	183%
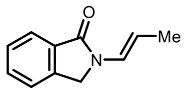	**Our method**	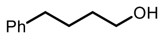 2.5 equiv	-	80%	170%	-	250%
	**Condition A**		-	80%	170%	-	250%
	**Condition B**		90%	-	249%	-	249%
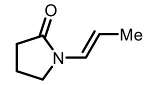	**Our method**	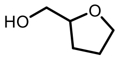 7.5 equiv	17%	83%	667%	-	750%
	**Condition A**		-	79%	670%	-	749%
	**Condition B**		92%	-	735%	-	735%

G. Catalytic Intramolecular Reaction Procedure

i) General Procedure

General Procedure G1: Intramolecular Hydroetherification

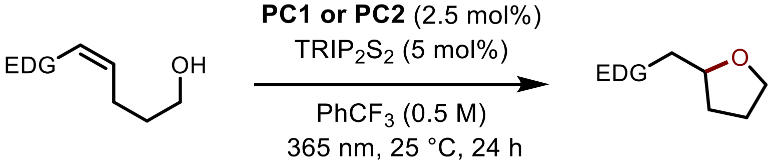

A flame-dried 1 dram vial equipped with a magnetic stir bar was transferred to a nitrogen-filled glovebox. The vial was charged with substrate (0.1 mmol, 1.0 equiv) and **PC1** or **PC2** (0.0025 mmol, 2.5 mol%). Following these additions, freeze-pump-thaw degassed anhydrous $PhCF_3$ (0.2 mL, 0.5 M) was added to the vial, after which the vial was sealed with a threaded cap. The resulting solution was allowed to stir for 1 minute. The cap was removed and $TRIP_2S_2$ (0.005 mmol, 5 mol%) was quickly added to the vial as a solid in a single portion. The vial was then sealed with a threaded cap, removed from the glovebox, and further reinforced with parafilm. The reaction vial was then transferred to a Hepatochem photoreactor attached to a recirculating chiller set to maintain a temperature of 25 °C.

The reaction was set to stir under 365 nm light irradiation for 24 hours. At the designated time, the vial was removed from the photoreactor and the solvent was removed *in vacuo*. The resultant crude was purified using flash column chromatography on silica gel to access the target intramolecular hydroetherification product.

General Procedure G2: Intramolecular Hydroetherification

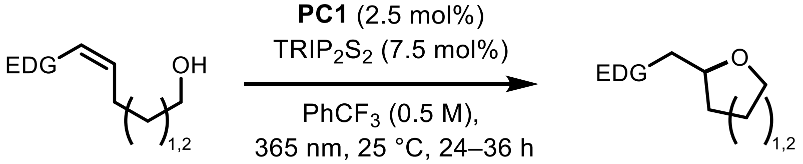

A flame-dried 1 dram vial equipped with a magnetic stir bar was transferred to a nitrogen-filled glovebox. The vial was charged with substrate (0.1 mmol, 1.0 equiv) and **PC1** (0.0025 mmol, 2.5 mol%). Following these additions, freeze-pump-thaw degassed anhydrous $PhCF_3$ (0.2 mL, 0.5 M) was added to the vial, after which the vial was sealed with a threaded cap. The resulting solution was allowed to stir for 1 minute. The cap was removed and $TRIP_2S_2$ (0.0075 mmol, 7.5 mol%) was quickly added to the vial as a solid in a single portion. The vial was then sealed with a threaded cap, removed from the glovebox, and further reinforced with parafilm. The reaction vial was then transferred to a Hepatochem photoreactor attached to a recirculating chiller set to maintain a temperature of 25 °C.

The reaction was set to stir under 365 nm light irradiation for 24–36 hours. At the designated time, the vial was removed from the photoreactor and the solvent was removed *in vacuo*. The resultant crude was purified using flash column chromatography on silica gel to access the target intramolecular hydroetherification product.

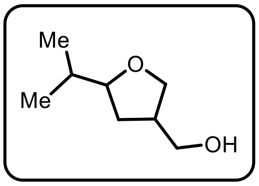

(5-isopropyltetrahydrofuran-3-yl)methanol (35b) The reaction was set up using the general procedure **G1** with **PC1** (2.3 mg, 0.0025 mmol, 2.5 mol%) and 2-(3-methylbut-2-en-1-yl)propane-1,3-diol (14.4 mg, 0.1 mmol) for 24 hours. Following this, the crude product was purified using silica column chromatography (hexanes/acetone 100/0 to hexanes/acetone 50/50) to yield the indicated product as an inseparable mixture of diastereomers in 74% yield (10.4 mg, *dr* = 2.6:1) as a colorless liquid.

IR (Diamond-ATR, neat) ṽ (cm^{-1}): 3408, 2957, 2926, 2870, 1079, 1020.

^{1}H NMR (600 MHz, $CDCl_3$): For major diastereomer: δ 3.80 (dd, *J* = 8.8, 7.5 Hz, 1H), 3.70 (dd, *J* = 8.9, 5.3 Hz, 1H), 3.61 – 3.51 (m, 2H), 3.48 (adt, *J* = 13.1, 9.5 Hz, 1H), 2.51 – 2.44 (m, 1H), 2.05 (ddd, *J* = 12.3, 8.1, 6.1 Hz, 1H), 1.76 – 1.63 (m, 2H), 1.23 – 1.19 (m, 1H), 0.96 (d, *J* = 6.7 Hz, 3H), 0.88 (d, *J* = 6.5 Hz, 3H). For minor diastereomer: δ 3.98 (dd, *J* = 8.8, 6.9 Hz, 1H), 3.63 (dd, *J* = 10.5, 6.7 Hz, 1H), 3.55 – 3.52 (m, 3H), 2.51 – 2.44 (m, 1H), 1.76 – 1.63 (m, 3H), 1.23 – 1.19 (m, 1H), 0.97 (d, *J* = 6.6 Hz, 3H), 0.86 (d, *J* = 6.7 Hz, 3H).

^{13}C NMR (151 MHz, $CDCl_3$): For major diastereomer: δ 85.7, 70.5, 65.6, 42.0, 33.3, 33.8, 19.6, 18.7. For minor diastereomer: δ 84.4, 70.6, 65.0, 41.9, 33.3, 32.2, 19.4, 18.6.

HRMS (ESI): m/z: $[M+H]^+$ calc'd for $C_8H_{17}O_2^+$: 145.1229. Found: 145.1222.

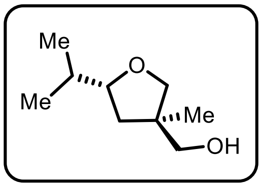

(5-isopropyl-3-methyltetrahydrofuran-3-yl)methanol (36b) The reaction was set up using the general procedure **G1** with **PC1** (2.3 mg, 0.0025 mmol, 2.5 mol%) and 2-methyl-2-(3-methylbut-2-en-1-yl)propane-1,3-diol (15.8 mg, 0.1 mmol) for 24 hours. Following this, the crude product was purified using silica column chromatography (hexanes/EtOAc 100/0 to hexanes/EtOAc 60/40) to yield the indicated product as an inseparable mixture of diastereomers in 80% yield (12.6 mg, *dr* = 1.6:1) as a colorless liquid.

IR (Diamond-ATR, neat) ṽ (cm^{-1}): 3411, 2958, 2930, 2872, 1387, 1081.

^{1}H NMR (600 MHz, $CDCl_3$): For major diastereomer: δ 3.67 (d, *J* = 8.8 Hz, 1H), 3.63 – 3.55 (m, 1H), 3.50 – 3.47 (m, 3H), 1.92 (dd, *J* = 6.7, 6.0 Hz, 1H), 1.72 – 1.65 (m, 2H), 1.32 (dd, *J* = 9.2, 3.3 Hz, 1H), 1.07 (s, 3H), 0.95 (d, *J* = 6.9 Hz, 3H), 0.85 (d, *J* = 6.6 Hz, 3H). For minor diastereomer: δ 3.78 (d, *J* = 8.8 Hz, 1H), 3.63 – 3.55 (m, 1H), 3.53 – 3.44 (m, 2H), 3.38 (d, *J* = 8.8 Hz, 1H), 1.72 – 1.65 (m, 1H), 1.64 – 1.61 (m, 2H), 1.48 (dd, *J* = 9.4, 2.8 Hz, 1H), 1.11 (s, 3H), 0.97 (d, *J* = 7.3 Hz, 3H), 0.86 (d, *J* = 6.2 Hz, 3H).

^{13}C NMR (151 MHz, $CDCl_3$): For major diastereomer: δ 85.3, 76.1, 69.1, 45.3, 40.1, 33.7, 22.2, 19.4, 18.5. For minor diastereomer: δ 85.3, 76.5, 70.2, 44.9, 40.1, 33.4, 21.9, 19.6, 18.7.

HRMS (ESI): m/z: $[M+H]^+$ calc'd for $C_9H_{19}O_2^+$: 159.1385. Found: 159.1377.

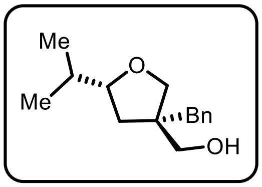

(3-benzyl-5-isopropyltetrahydrofuran-3-yl)methanol (37b) The reaction was set up using the general procedure **G1** with **PC1** (2.3 mg, 0.0025 mmol, 2.5 mol%) and 2-benzyl-2-(3-methylbut-2-en-1-yl)propane-1,3-diol (23.4 mg, 0.1 mmol) for 24 hours. Following this, the crude product was purified using silica column chromatography (hexanes/EtOAc 100/0 to hexanes/EtOAc 80/20) to yield the indicated product in 77% yield (18.0 mg, *dr* = 1.6:1) as a white solid.

Major Diastereomer

IR (Diamond-ATR, neat) ṽ (cm^{-1}): 3439, 2959, 2926, 2855, 2259, 1070.

^{1}H NMR (600 MHz, $CDCl_3$): δ 7.29 (at, *J* = 7.3 Hz, 2H), 7.24 (d, *J* = 7.1 Hz, 1H), 7.21 (d, *J* = 7.1 Hz, 2H), 3.70 (d, *J* = 8.4 Hz, 1H), 3.65 – 3.60 (m, 2H), 3.47 (d, *J* = 4.9, 2H), 2.85 (d, *J* = 13.4 Hz,

1H), 2.76 (d, J = 13.4 Hz, 1H), 1.81 (dd, J = 12.6, 6.5 Hz, 1H), 1.68 (asxt, J = 6.8 Hz, 1H), 1.52 (dd, J = 12.9, 9.4 Hz, 1H), 1.45 (at, J = 5.0 Hz, 1H), 0.96 (d, J = 6.8 Hz, 3H), 0.86 (d, J = 6.9 Hz, 3H).

^{13}C NMR (151 MHz, $CDCl_3$): δ 138.6, 130.1, 128.5, 126.5, 84.9, 74.6, 65.6, 49.7, 41.0, 38.2, 33.8, 19.4, 18.6.

HRMS (ESI): m/z: $[M+H]^+$ calc'd for $C_{15}H_{23}O_2^+$: 235.1698. Found: 235.1692.

Minor Diastereomer

IR (Diamond-ATR, neat) ṽ (cm^{-1}): 3425, 2957, 2924, 2870, 2357, 1033.

^{1}H NMR (600 MHz, $CDCl_3$): δ 7.29 (at, J = 7.3 Hz, 2H), 7.25 – 7.20 (m, 3H), 3.74 (d, J = 8.9 Hz, 1H), 3.64 – 3.57 (m, 2H), 3.47 (dd, J = 5.3, 5.1 Hz, 1H), 3.42 (dd, J = 5.8, 4.4 Hz, 2H), 2.86 (d, J = 13.4 Hz, 1H), 2.78 (d, J = 13.4 Hz, 1H), 1.87 (dd, J = 5.8, 6.9 Hz, 1H), 1.70 – 1.65 (m, 1H), 1.49 (at, J = 4.9 Hz, 1H), 1.33 (dd, J = 9.1, 3.6 Hz, 1H), 0.97 (d, J = 6.6 Hz, 3H), 0.86 (d, J = 6.7 Hz, 3H).

^{13}C NMR (151 MHz, $CDCl_3$): δ 138.5, 130.3, 128.5, 126.5, 85.1, 75.1, 66.9, 49.3, 40.1, 37.7, 33.5, 19.5, 18.7.

HRMS (ESI): m/z: $[M+H]^+$ calc'd for $C_{15}H_{23}O_2^+$: 235.1698. Found: 235.1690.

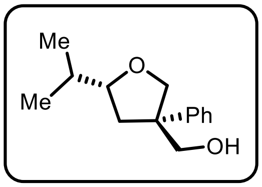

<u>(5-isopropyl-3-phenyltetrahydrofuran-3-yl)methanol (38b)</u> The reaction was set up using the general procedure **G1** with **PC1** (2.3 mg, 0.0025 mmol, 2.5 mol%) and 2-(3-methylbut-2-en-1-yl)-2-phenylpropane-1,3-diol (22.0 mg, 0.1 mmol) for 24 hours. Following this, the crude product was purified using silica column chromatography (hexanes/EtOAc 100/0 to hexanes/EtOAc 90/10) to yield the indicated product in 48% yield (10.6 mg, *dr* = 1.3:1) as a colorless liquid.

Major Diastereomer

IR (Diamond-ATR, neat) ṽ (cm^{-1}): 3397, 2957, 2927, 2873, 2360, 2339, 1035, 700.

^{1}H NMR (600 MHz, $CDCl_3$): δ 7.36 (at, J = 7.6 Hz, 2H), 7.26 – 7.25 (m, 1H), 7.22 (ad, J = 7.7 Hz, 2H), 4.28 (d, J = 8.8 Hz, 1H), 3.89 (d, J = 8.9 Hz, 1H), 3.68 (dd, J = 7.8, 2.3 Hz, 1H), 3.63 – 3.56 (m, 2H), 2.26 (dd, J = 7.8, 5.0 Hz, 1H), 1.88 (dd, J = 8.3, 4.4 Hz, 1H), 1.83 – 1.75 (m, 1H), 1.43 (dd, J = 5.4, 2.0 Hz, 1H), 1.01 (d, J = 6.6 Hz, 3H), 0.93 (d, J = 6.8 Hz, 3H).

^{13}C NMR (151 MHz, $CDCl_3$): δ 143.7, 128.8, 127.4, 126.9, 85.3, 74.3, 70.8, 53.5, 38.6, 33.5, 19.5, 18.8.

HRMS (ESI): m/z: $[M+H]^+$ calc'd for $C_{14}H_{21}O_2^+$: 221.1541. Found: 221.1532.

Minor Diastereomer

IR (Diamond-ATR, neat) ṽ (cm^{-1}): 3438, 2957, 2927, 2869, 2359, 2331, 1070, 700.

^{1}H NMR (600 MHz, $CDCl_3$): δ 7.36 (at, J = 7.6 Hz, 2H), 7.27 (at, J = 7.5 Hz, 2H), 7.18 (ad, J = 7.5 Hz, 2H), 4.21 (d, J = 8.4 Hz, 1H), 3.92 (d, J = 8.4 Hz, 1H), 3.86 (adt, J = 9.2, 6.9 Hz, 1H), 3.74 – 3.68 (m, 2H), 2.46 (dd, J = 6.3, 6.2 Hz, 1H), 1.85 (dd, J = 9.4, 2.3 Hz, 1H), 1.74 – 1.66 (m, 1H), 1.38 (at, J = 6.7 Hz, 1H), 0.98 (d, J = 6.6 Hz, 3H), 0.88 (d, J = 6.8 Hz, 3H).

^{13}C NMR (151 MHz, $CDCl_3$): δ 143.5, 128.8, 127.2, 127.1, 84.5, 73.9, 69.3, 54.2, 37.6, 33.8, 19.3, 18.4.

HRMS (ESI): m/z: $[M+H]^+$ calc'd for $C_{14}H_{21}O_2^+$: 221.1541. Found: 221.1535.

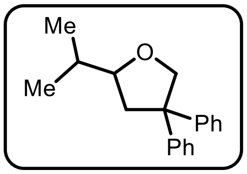

2-isopropyl-4,4-diphenyltetrahydrofuran (39b) The reaction was set up using a modified general procedure **G1** with **PC2** (2.4 mg, 0.0025 mmol, 2.5 mol%) and 5-methyl-2,2-diphenylhex-4-en-1-ol (26.6 mg, 0.1 mmol) for 24 hours. Following this, the crude product was purified using silica column chromatography (MeCN/H_2O 0/100 to MeCN/H_2O 100/0) and isolated using silica preparatory thin layer chromatography to yield the indicated product as a colorless oil. The yield reported is an NMR yield (61%) using 1,1,2,2-tetrachloroethane (0.05 mmol) as an internal standard due to co-polar byproduct.

IR (Diamond-ATR, neat) ṽ (cm^{-1}): 2957, 2868, 1494, 1468, 1447, 1078, 774.

^{1}H NMR (600 MHz, $CDCl_3$): δ 7.33 – 7.27 (m, 6H), 7.23 – 7.17 (m, 4H), 4.65 (dd, *J* = 8.7, 0.8 Hz, 1H), 4.05 (d, *J* = 8.7 Hz, 1H), 3.70 (ddd, *J* = 10.0, 8.7, 6.0 Hz, 1H), 2.53 (dd, *J* = 12.1, 5.6 Hz, 1H), 2.34 (dd, *J* = 12.6, 10.3, Hz, 1H), 1.80 – 1.72 (m, 1H), 0.98 (d, *J* = 6.6 Hz, 3H), 0.88 (d, *J* = 6.8 Hz, 3H).

^{13}C NMR (151 MHz, $CDCl_3$): δ 146.6, 146.2, 128.5, 128.4, 127.3, 127.3, 126.5, 126.3, 84.3, 77.0, 56.1, 42.6, 33.7, 19.4, 18.5.

HRMS (ESI): m/z: $[M+H]^+$ calc'd for $C_{19}H_{23}O^+$: 267.1749. Found: 267.1745

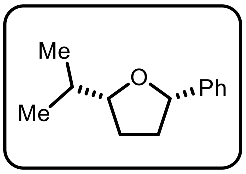

2-isopropyl-5-phenyltetrahydrofuran (40b) The reaction was set up using a modified general procedure **G1** with **PC2** (2.4 mg, 0.0025 mmol, 2.5 mol%) and 5-methyl-1-phenylhex-4-en-1-ol (19.0 mg, 0.1 mmol) for 24 hours. Following this, the crude product was purified using silica column chromatography (pentane/Et_2O 100/0 to pentane/Et_2O 95/5) to yield the indicated product as a colorless oil. The yield reported is an NMR yield (36%, *dr* = 2:1) using 1,1,2,2-tetrachloroethane (0.05 mmol) as an internal standard due to volatility.

IR (Diamond-ATR, neat) ṽ (cm^{-1}): 2959, 2924, 2854, 2364, 2346, 2323, 1261, 1098, 1018, 800.

^{1}H NMR (600 MHz, $CDCl_3$): For major diastereomer: 7.37 – 7.31 (m, 4H), 7.25 – 7.22 (m, 1H), 4.86 (at, *J* = 7.2 Hz, 1H), 3.74 – 3.70 (m, 1H), 2.31 – 2.25 (m, 1H), 2.00 – 1.96 (m, 1H), 1.87 – 1.67 (m, 3H), 1.06 (d, *J* = 6.7 Hz, 3H), 0.95 (d, *J* = 6.8 Hz, 3H) . For minor diastereomer: 7.37 – 7.31 (m, 4H), 7.25 – 7.22 (m, 1H), 4.96 (dd, *J* = 6.2, 2.2 Hz, 1H), 3.90 – 3.87 (m, 1H), 2.37 – 2.32 (m, 1H), 2.07 – 2.02 (m, 1H), 1.87 – 1.67 (m, 3H), 1.02 (d, *J* = 6.7 Hz, 3H), 0.92 (d, *J* = 6.8 Hz, 3H).

^{13}C NMR (151 MHz, $CDCl_3$): For major diastereomer: 143.7, 128.3, 127.2, 125.9, 85.5, 80.8, 34.8, 33.4, 28.9, 19.6, 18.8. For minor diastereomer: 143.7, 128.4, 127.1, 125.7, 85.6, 80.6, 35.9, 33.5, 30.5, 19.5, 18.4.

HRMS (ESI): m/z: $[M+H]^+$ calc'd for $C_{13}H_{19}O^+$: 191.1436. Found: 191.1428.

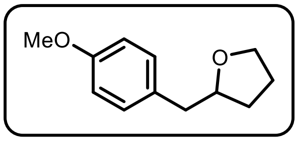

2-(4-methoxybenzyl)tetrahydrofuran (41b). The reaction was set up using the general procedure **G2** with **PC1** (2.3 mg, 0.0025 mmol, 2.5 mol%) and 5-(4-methoxyphenyl)pent-4-en-1-ol (19.2 mg, 0.1 mmol) for 24 hours. Following this, the crude product was purified using silica column chromatography (hexanes/EtOAc 100/0 to hexanes/EtOAc 0/100) to yield the indicated product in 68% yield (13.0 mg) as a white solid. The resulting product has been previously reported and matches the obtained spectroscopic data tabulated below.[5]

IR (Diamond-ATR, neat) ṽ (cm^{-1}): 2921, 2852, 2361, 2342, 1462, 1259, 1019.

^{1}H NMR (600 MHz, $CDCl_3$): δ 7.14 (d, *J* = 8.7 Hz, 2H), 6.83 (d, *J* = 8.6 Hz, 2H), 4.02 (ap, *J* = 6.8 Hz, 1H), 3.89 (aq, *J* = 6.8 Hz, 1H), 3.78 (s, 3H), 3.73 (atd, *J* = 7.9, 6.2 Hz, 1H) 2.85 (dd, *J* = 13.7, 6.4 Hz, 1H), 2.69 (dd, *J* = 13.7, 6.5 Hz, 1H), 1.94 – 1.79 (m, 3H), 1.58 – 1.51 (m, 1H).

13C NMR (151 MHz, $CDCl_3$): δ 157.0, 130.1, 129.1, 112.7, 79.2, 66.9, 54.2, 39.9, 29.9, 24.6.
HRMS (ESI): m/z: $[M+H]^+$ calc'd for $C_{12}H_{17}O_2^+$: 193.1229. Found: 193.1220.

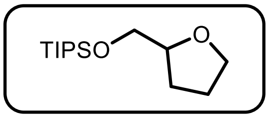

triisopropyl((tetrahydrofuran-2-yl)methoxy)silane (42b). The reaction was set up using the general procedure **G2** with 5-((triisopropylsilyl)oxy)pent-4-en-1-ol (25.8 mg, 0.1 mmol) for 24 hours. Following this, the crude product was purified using silica column chromatography (hexanes/Et_2O 100/0 to hexanes/Et_2O 90/10) to yield the indicated product in 70% yield (18.1 mg) as a colorless oil. The resulting product has been previously reported and matches the obtained spectroscopic data tabulated below.[11]
IR (Diamond-ATR, neat) ṽ (cm^{-1}): 2942, 2865, 2361, 2342, 1071.
1H NMR (400 MHz, $CDCl_3$): δ 3.99 (ap, *J* = 6.0 Hz, 1H), 3.85 (aq, *J* = 7.1 Hz, 1H), 3.79 – 3.70 (m, 2H), 3.63 (dd, *J* = 10.2, 5.3 Hz, 1H), 1.98 – 1.82 (m, 3H), 1.80 – 1.73 (m, 1H), 1.06 (ad, *J* = 5.6 Hz, 21H).
13C NMR (151 MHz, $CDCl_3$): δ 79.7, 68.6, 66.3, 28.1, 25.9, 18.1, 12.1.
HRMS (ESI): m/z: $[M+H]^+$ calc'd for $C_{14}H_{31}O_2Si^+$: 259.2094. Found: 259.2084.

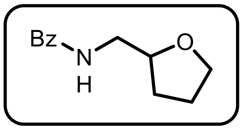

N-((tetrahydrofuran-2-yl)methyl)benzamide (43b). The reaction was set up using the general procedure **G2** with *N*-(5-hydroxypent-1-en-1-yl)benzamide (20.5 mg, 0.1 mmol) for 36 hours. Following this, the crude product was purified using silica column chromatography (hexanes/EtOAc 100/0 to hexanes/EtOAc 50/50) to yield the indicated product in 69% yield (14.1 mg) as a white solid.
IR (Diamond-ATR, neat) ṽ (cm^{-1}): 3292, 2932, 2870, 1634, 1576, 1458, 1492, 1314, 1066, 698, 680, 665.
1H NMR (600 MHz, $CDCl_3$): δ 7.79 – 7.75 (m, 2H), 7.50 – 7.45 (m, 1H), 7.41 (at, *J* = 7.7 Hz, 2H), 6.59 (s, 1H), 4.06 (aqd, *J* = 7.2, 3.3 Hz, 1H), 3.87 (adt, *J* = 8.5, 6.7 Hz, 1H), 3.77 (addt, *J* = 15.3, 8.5, 5.1 Hz, 2H), 3.34 (ddd, *J* = 13.8, 7.5, 4.9 Hz, 1H), 2.01 (adt, *J* = 12.6, 6.7 Hz, 1H), 1.96 – 1.86 (m, 2H), 1.60 (adq, *J* = 12.3, 7.7 Hz, 1H).
13C NMR (151 MHz, $CDCl_3$): δ 167.6, 134.6, 131.5, 128.6, 127.0, 77.9, 68.2, 43.7, 28.7, 26.0.
HRMS (ESI): m/z: $[M+H]^+$ calc'd for $C_{12}H_{16}NO_2^+$: 206.1181. Found: 206.1178.

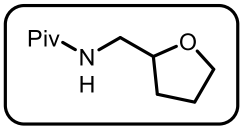

N-((tetrahydrofuran-2-yl)methyl)pivalamide (44b). The reaction was set up using the general procedure **G2** with *N*-(5-hydroxypent-1-en-1-yl)pivalamide (18.5 mg, 0.1 mmol) for 36 hours. Following this, the crude product was purified using silica column chromatography (hexanes/EtOAc 100/0 to hexanes/EtOAc 50/50) to yield the indicated product in 72% yield (13.4 mg) as a white solid.
IR (Diamond-ATR, neat) ṽ (cm^{-1}): 3362, 2967, 2927, 1526, 1366, 1210, 1117.
1H NMR (400 MHz, $CDCl_3$): δ 6.00 (brs, 1H), 3.94 (aqd, *J* = 7.1, 3.3 Hz, 1H), 3.85 (aq, *J* = 7.9 Hz, 1H), 3.74 (aq, *J* = 7.6 Hz, 1H), 3.55 (ddd, *J* = 13.8, 6.4, 3.3 Hz, 1H), 3.15 (ddd, *J* = 13.8, 7.1, 5.0 Hz, 1H), 2.01 – 1.92 (m, 1H), 1.88 (adt, *J* = 13.8, 6.7 Hz, 2H), 1.52 (adq, *J* = 11.0, 7.4 Hz, 1H), 1.19 (s, 9H).
13C NMR (151 MHz, $CDCl_3$): δ 178.8, 78.1, 68.4, 43.3, 39.0, 28.7, 27.8, 26.2.
HRMS (ESI): m/z: $[M+H]^+$ calc'd for $C_{10}H_{20}NO_2^+$: 186.1494. Found: 186.1486.

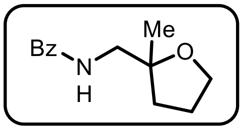

N-((2-methyltetrahydrofuran-2-yl)methyl)benzamide (45b). The reaction was set up using the general procedure **G2** with *N*-(5-hydroxy-2-methylpent-1-en-1-yl)benzamide (21.9 mg, 0.1 mmol) for 36 hours. Following this, the crude product was purified using silica column chromatography (hexanes/EtOAc 100/0 to hexanes/EtOAc 50/50) to yield the indicated product in 77% yield (16.8 mg) as a white solid.

IR (Diamond-ATR, neat) ṽ (cm^{-1}): 2967, 2827, 2868, 2359, 1639, 1534, 1489, 1292.

^{1}H NMR (600 MHz, $CDCl_3$): δ 7.80 – 7.74 (m, 2H), 7.51 – 7.46 (m, 1H), 7.46 – 7.38 (m, 2H), 6.52 (s, 1H), 3.88 (atd, *J* = 7.9, 5.9 Hz, 1H), 3.86 – 3.81 (m, 1H), 3.56 – 3.48 (m, 2H), 2.00 – 1.95 (m, 1H), 1.94 – 1.88 (m, 1H), 1.87 – 1.82 (m, 1H), 1.72 (ddd, *J* = 12.6, 8.5, 5.7 Hz, 1H), 1.24 (s, 3H).

^{13}C NMR (151 MHz, $CDCl_3$): δ 167.7, 134.7, 131.5, 128.7, 127.0, 82.3, 67.9, 47.5, 34.8, 26.4, 24.5.

HRMS (ESI): m/z: $[M+H]^+$ calc'd for $C_{13}H_{18}NO_2^+$: 220.1337. Found: 220.1335.

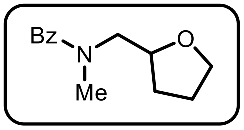

N-methyl-N-((tetrahydrofuran-2-yl)methyl)benzamide (46b). The reaction was set up using the general procedure **G2** with *N*-(5-hydroxypent-1-en-1-yl)-*N*-methylbenzamide (21.9 mg, 0.1 mmol) for 24 hours. Following this, the crude product was purified using silica column chromatography (hexanes/EtOAc 100/0 to hexanes/EtOAc 50/50) to yield the indicated product in 71% yield (15.5 mg) as a colorless liquid.

IR (Diamond-ATR, neat) ṽ (cm^{-1}): 2926, 2869, 2358, 1630, 1400, 1070.

^{1}H NMR (500 MHz, DMSO-d_6): δ 7.44 – 7.40 (m, 3H), 7.38 – 7.34 (m, 2H), 4.06 (brs, 1H), 3.64 (aq, *J* = 7.2 Hz, 2H), 3.36 (brs, 2H), 2.98 (brs, 3H), 1.91 (brs, 1H), 1.79 (brs, 2H), 1.45 (brs, 1H).

^{13}C NMR (151 MHz, DMSO-d_6): δ 170.3, 136.9, 129.3, 128.3, 126.7, 77.0, 67.1, 50.6, 33.0, 28.8, 25.1.

HRMS (ESI): m/z: $[M+H]^+$ calc'd for $C_{13}H_{18}NO_2{}^+$: 220.1337. Found: 220.1339.

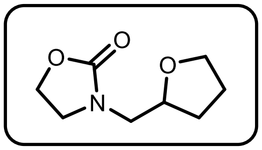

3-((tetrahydrofuran-2-yl)methyl)oxazolidin-2-one (47b). The reaction was set up using the general procedure **G2** with 3-(5-hydroxypent-1-en-1-yl)oxazolidin-2-one (17.1 mg, 0.1 mmol) for 36 hours. Following this, the crude product was purified using silica column chromatography (EtOAc/MeOH 100/0 to EtOAc/MeOH 90/10) to yield the indicated product in 65% yield (11.1 mg) as a colorless liquid.

IR (Diamond-ATR, neat) ṽ (cm^{-1}): 2874, 1729, 1425, 1263, 1053, 864, 762, 697.

^{1}H NMR (600 MHz, $CDCl_3$): δ 4.31 (at, *J* = 8.1 Hz, 2H), 4.04 (aqd, *J* = 7.3, 3.2 Hz, 1H), 3.85 (aq, *J* = 7.6 Hz, 1H), 3.81 – 3.71 (m, 2H), 3.64 (aq, *J* = 8.3 Hz, 1H), 3.49 (dd, *J* = 14.4, 3.2 Hz, 1H), 3.16 (dd, *J* = 14.4, 7.4 Hz, 1H), 2.04 – 1.96 (m, 1H), 1.95 – 1.83 (m, 2H), 1.57 (adq, *J* = 12.3, 7.9 Hz, 1H).

^{13}C NMR (151 MHz, $CDCl_3$): δ 159.0, 78.1, 68.3, 62.1, 48.5, 46.2, 29.0, 25.7.

HRMS (ESI): m/z: $[M+H]^+$ calc'd for $C_8H_{14}NO_3^+$: 172.0973. Found: 172.0968.

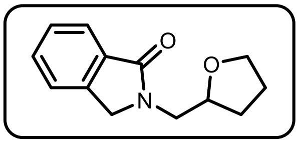

2-((tetrahydrofuran-2-yl)methyl)isoindolin-1-one (48b). The reaction was set up using the general procedure **G2** with 2-(5-hydroxypent-1-en-1-yl)isoindolin-1-one (21.7 mg, 0.1 mmol) for 24 hours. Following this, the crude product was purified using silica column chromatography (hexanes/EtOAc/MeOH 100/0/0 to hexanes/EtOAc/MeOH 0/100/0 to hexanes/EtOAc/MeOH 0/0/100) to yield the indicated product in 64% yield (13.9 mg) as a white solid.

IR (Diamond-ATR, neat) ṽ (cm^{-1}): 2858, 1716, 1684, 1471, 1395.
^{1}H NMR (600 MHz, $CDCl_3$): δ 7.85 (d, *J* = 7.4 Hz, 1H), 7.52 (at, *J* = 7.4 Hz, 1H), 7.44 (at, *J* = 7.8 Hz, 2H), 4.63 (d, *J* = 17.4 Hz, 1H), 4.49 (d, *J* = 17.4 Hz, 1H), 4.13 (aqd, *J* = 7.1, 3.3 Hz, 1H), 3.95 – 3.83 (m, 2H), 3.75 (aq, *J* = 8.3 Hz, 1H), 3.53 (dd, *J* = 14.3, 7.0 Hz, 1H), 2.11 – 1.98 (m, 1H), 1.89 (ap, *J* = 8.1 Hz, 2H), 1.71 – 1.58 (m, 1H).
^{13}C NMR (151 MHz, $CDCl_3$): δ 168.9, 141.9, 132.7, 131.3, 128.0, 123.8, 122.7, 78.7, 68.3, 51.9, 46.5, 29.1, 25.8.
HRMS (ESI): m/z: $[M+H]^+$ calc'd for $C_{13}H_{16}NO_2^+$: 218.1181. Found: 218.1179.

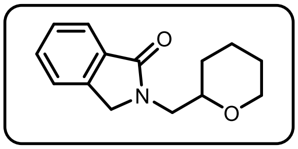

2-((tetrahydro-2H-pyran-2-yl)methyl)isoindolin-1-one (49b). The reaction was set up using the general procedure **G2** with 2-(6-hydroxyhex-1-en-1-yl)isoindolin-1-one (23.1 mg, 0.1 mmol) for 24 hours. Following this, the crude product was purified using silica column chromatography (hexanes/EtOAc 100/0 to hexanes/EtOAc 0/100) to yield the indicated product in 20% yield (4.0 mg) as a white solid.
IR (Diamond-ATR, neat) ṽ (cm^{-1}): 2860, 1676, 1468, 1450, 1399, 1323.
^{1}H NMR (600 MHz, $CDCl_3$): δ 7.85 (d, *J* = 7.4 Hz, 1H), 7.52 (atd, *J* = 7.4, 1.1 Hz, 1H), 7.44 (at, *J* = 7.8, 2H), 4.64 (d, *J* = 17.4 Hz, 1H), 4.48 (d, *J* = 17.4 Hz, 1H), 3.96 (adt, *J* = 11.4, 2.4 Hz, 1H), 3.81 (dd, *J* = 14.1, 2.9 Hz, 1H), 3.60 (addt, *J* = 10.7, 7.5, 2.5 Hz, 1H), 3.46 (dd, *J* = 14.2, 7.5 Hz, 1H), 3.38 (atd, *J* = 11.4, 3.1 Hz, 1H), 1.88 – 1.81 (m, 1H), 1.58 – 1.43 (m, 4H), 1.37 – 1.24 (m, 1H).
^{13}C NMR (151 MHz, $CDCl_3$): δ 168.8, 142.0, 132.9, 131.3, 127.9, 123.8, 122.7, 77.6, 68.5, 52.4, 47.8, 29.3, 26.0, 23.1.
HRMS (ESI): m/z: $[M+H]^+$ calc'd for $C_{14}H_{18}NO_2^+$: 232.1337. Found: 232.1333.

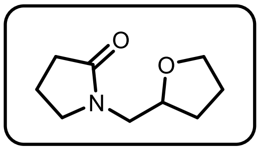

1-((tetrahydrofuran-2-yl)methyl)pyrrolidin-2-one (50b). The reaction was set up using the general procedure **G2** with 1-(5-hydroxypent-1-en-1-yl)pyrrolidin-2-one (16.9 mg, 0.1 mmol) for 24 hours. Following this, the crude product was purified using silica column chromatography (EtOAc/MeOH 100/0 to EtOAc/MeOH 90/10) to yield the indicated product as a colorless liquid. The yield reported is a ^{1}H NMR yield (96%) using 1,1,2,2-tetrachloroethane (0.05 mmol) as an internal standard due to poor visualization of product by standard detection methods during chromatography.
IR (Diamond-ATR, neat) ṽ (cm^{-1}): 2944, 2869, 1621, 1495, 1351, 1057, 727.
^{1}H NMR (600 MHz, $CDCl_3$): δ 4.00 (aqd, *J* = 7.2, 3.5 Hz, 1H), 3.83 (dt, *J* = 8.7, 6.7 Hz, 1H), 3.70 (dt, *J* = 8.0, 6.0 Hz, 1H), 3.54 (adt, *J* = 9.9, 7.3 Hz, 1H), 3.50 – 3.41 (m, 2H), 3.16 (dd, *J* = 14.0, 7.4 Hz, 1H), 2.35 (at, *J* = 8.2 Hz, 2H), 2.02 – 1.91 (m, 3H), 1.91 – 1.77 (m, 2H), 1.52 (addt, *J* = 12.4, 8.7, 7.3 Hz, 1H).
^{13}C NMR (151 MHz, $CDCl_3$): δ 175.5, 77.9, 68.1, 48.8, 46.8, 31.0, 29.2, 25.6, 18.2.
HRMS (ESI): m/z: $[M+H]^+$ calc'd for $C_9H_{16}NO_2^+$: 170.1181. Found: 170.1174.

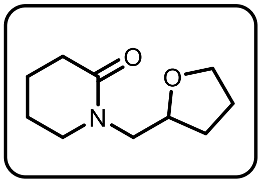

1-((tetrahydrofuran-2-yl)methyl)piperidin-2-one (51b). The reaction was set up using the general procedure **G2** with 1-(5-hydroxypent-1-en-1-yl)piperidin-2-one (18.3 mg, 0.1 mmol) for 24 hours. Following this, the crude product was purified using silica column chromatography (EtOAc/MeOH 100/0 to EtOAc/MeOH 90/10) to yield the indicated product in 88% yield (16.9 mg) as a colorless liquid.

IR (Diamond-ATR, neat) ṽ (cm^{-1}): 2867, 1622, 1494, 1447, 1350.
^{1}H NMR (600 MHz, $CDCl_3$): δ 4.09 (aqd, *J* = 7.3, 3.3 Hz, 1H), 3.83 (aq, *J* = 7.4 Hz, 1H), 3.80 (dd, *J* = 13.9, 3.5 Hz, 1H), 3.72 (atd, *J* = 8.0, 6.0 Hz, 1H), 3.55 – 3.49 (m, 1H), 3.35 – 3.29 (m, 1H), 3.06 (dd, *J* = 13.8, 7.7 Hz, 1H), 2.40 – 2.34 (m, 2H), 2.01 – 1.95 (m, 1H), 1.90 – 1.82 (m, 2H), 1.79 – 1.72 (m, 4H), 1.55 – 1.48 (m, 1H).
^{13}C NMR (151 MHz, $CDCl_3$): δ 170.3, 78.2, 68.1, 51.3, 49.7, 32.4, 29.4, 25.6, 23.4, 21.4.
HRMS (ESI): m/z: $[M+H]^+$ calc'd for $C_{10}H_{18}NO_2^+$: 184.1338. Found: 184.1337.

ii) NMR Spectra of Intramolecular Hydroetherification Products

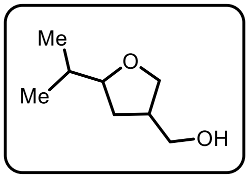

(5-isopropyltetrahydrofuran-3-yl)methanol (35b)

^{1}H NMR (600 MHz, $CDCl_3$)

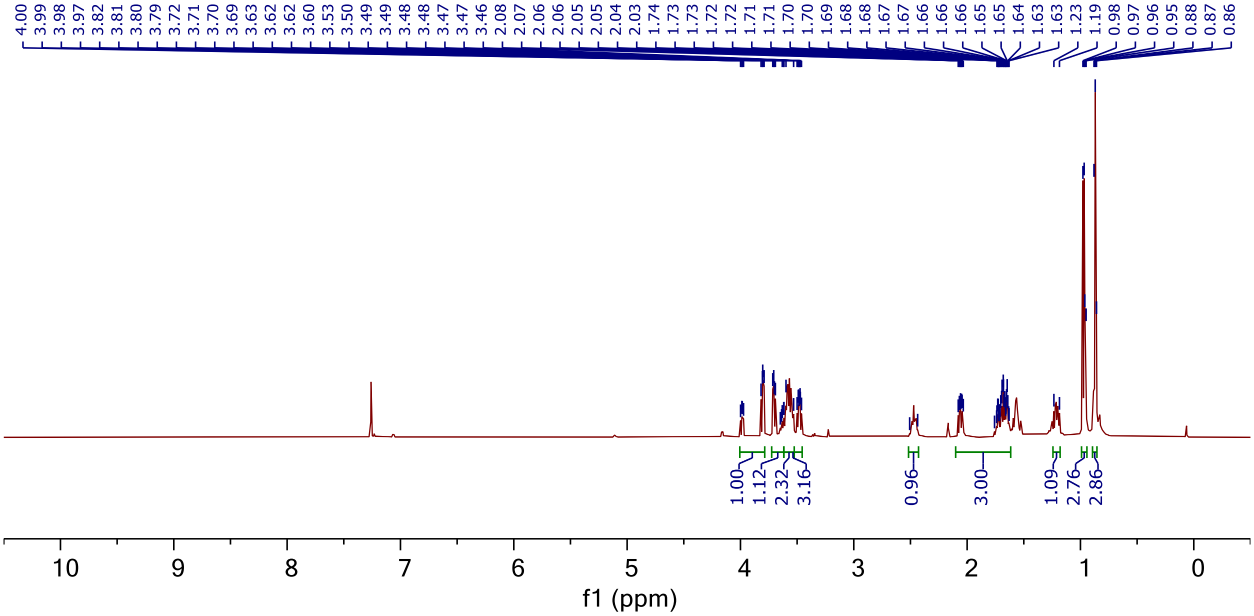

^{13}C NMR (151 MHz, $CDCl_3$)

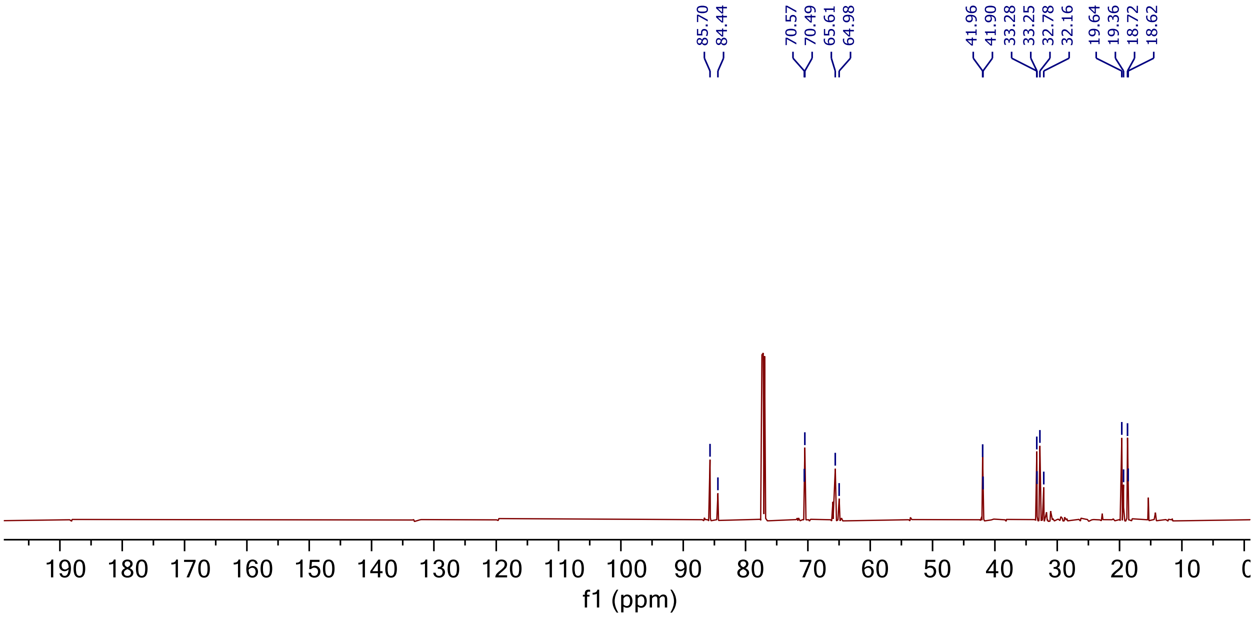

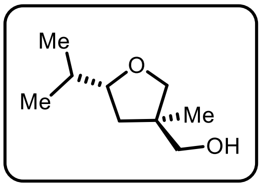

(5-isopropyl-3-methyltetrahydrofuran-3-yl)methanol (36b)

^{1}H NMR (600 MHz, $CDCl_3$)

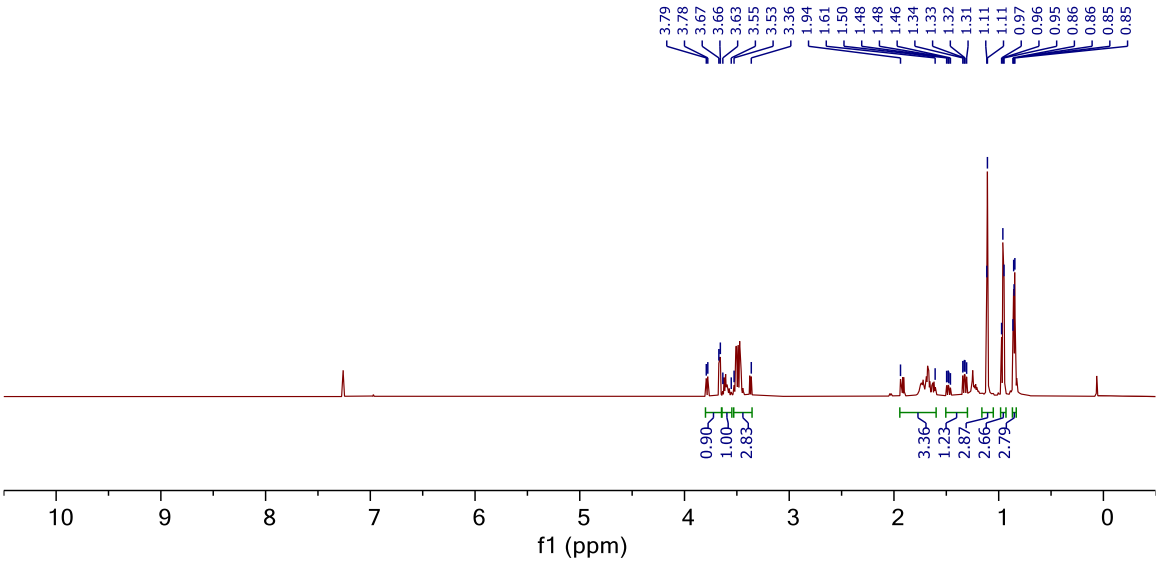

^{13}C NMR (151 MHz, $CDCl_3$)

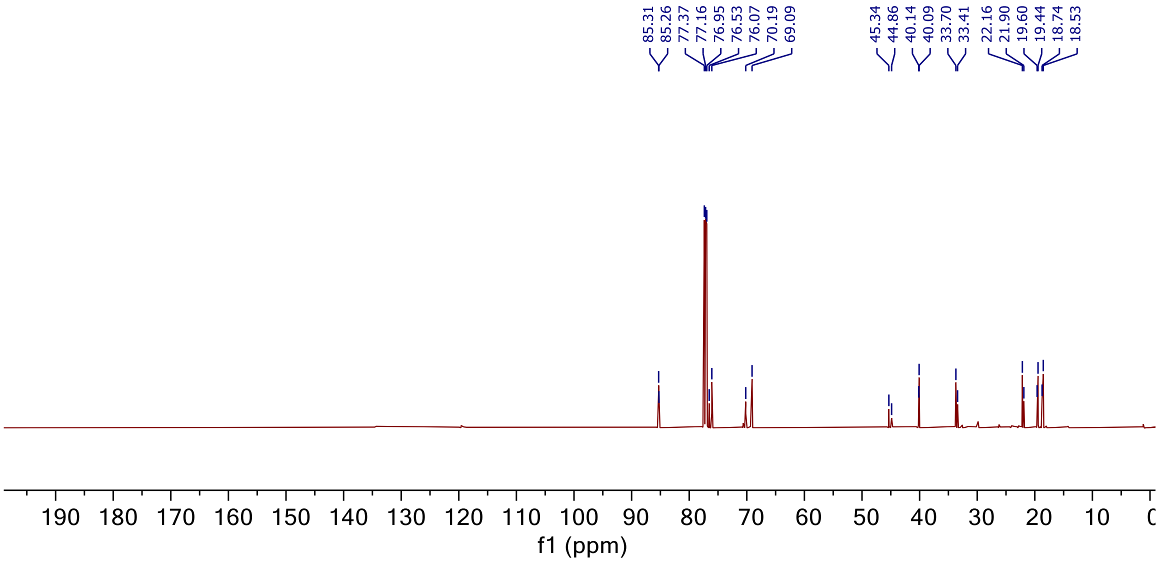

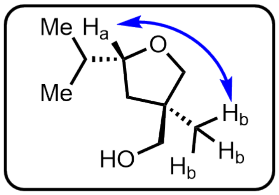

NOESY (600 MHz, $CDCl_3$):
Major Diastereomer

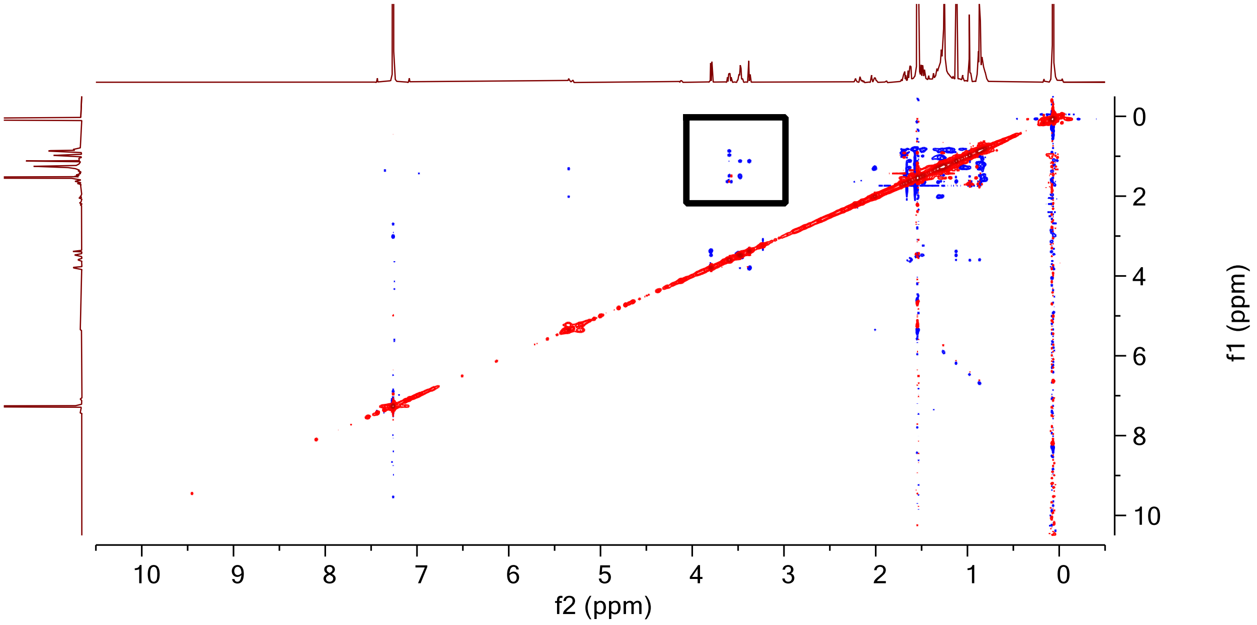

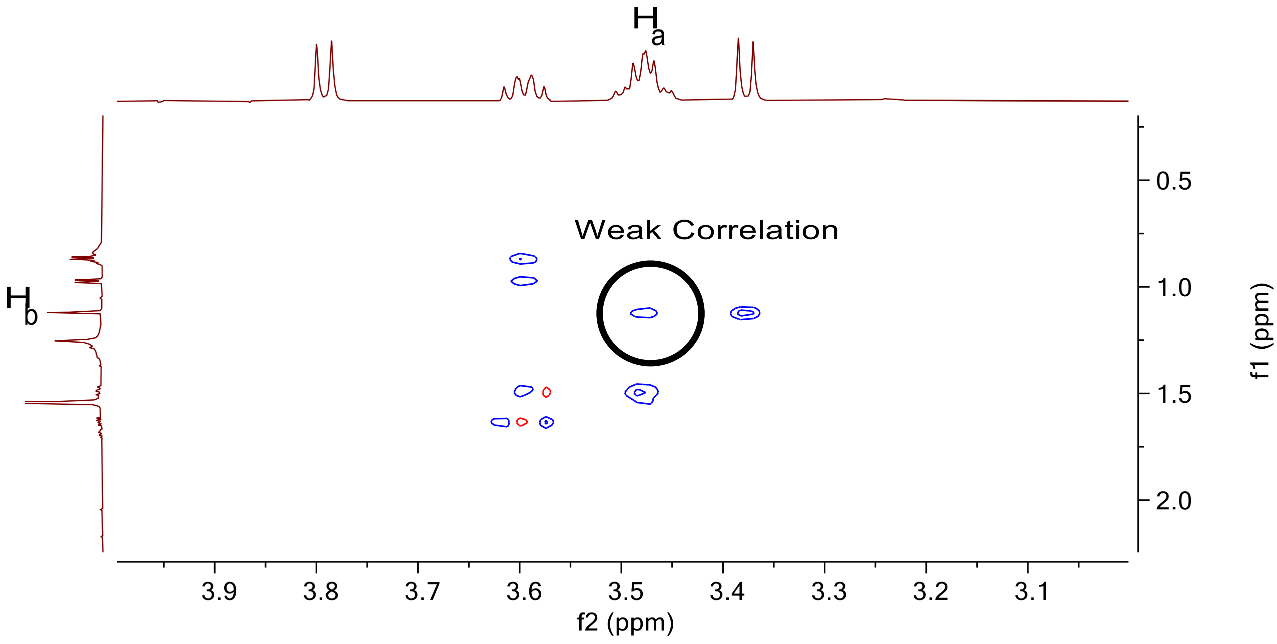

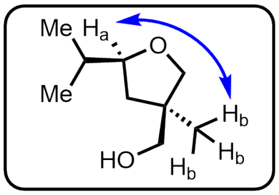

NOESY (600 MHz, $CDCl_3$):
Minor Diastereomer

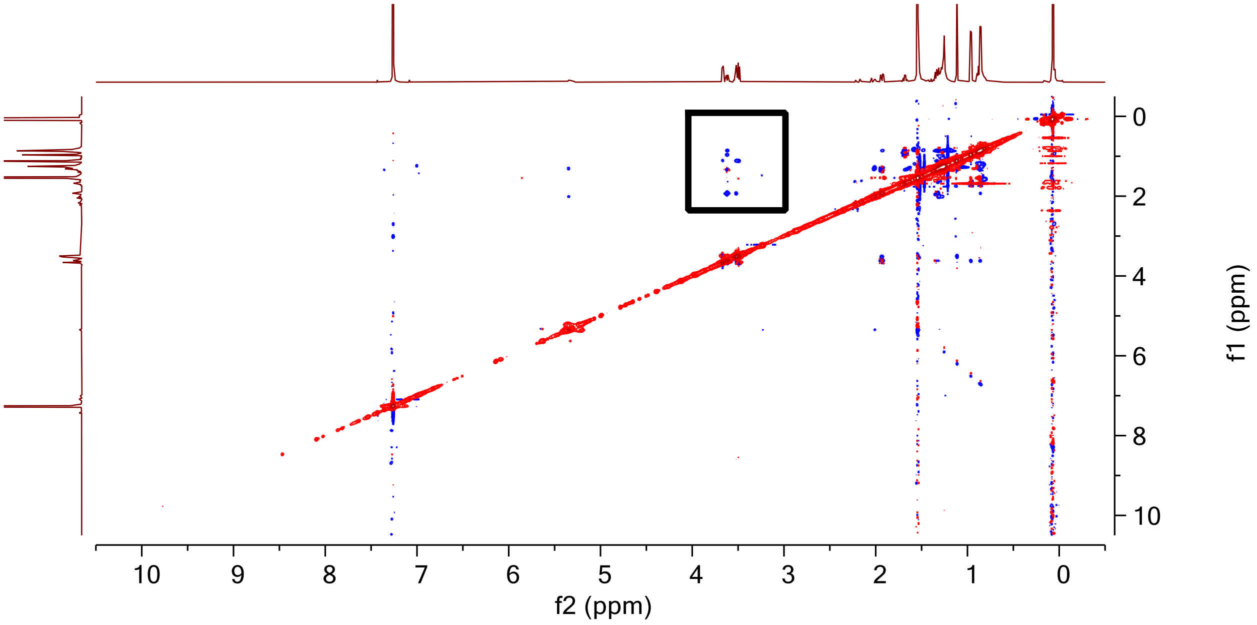

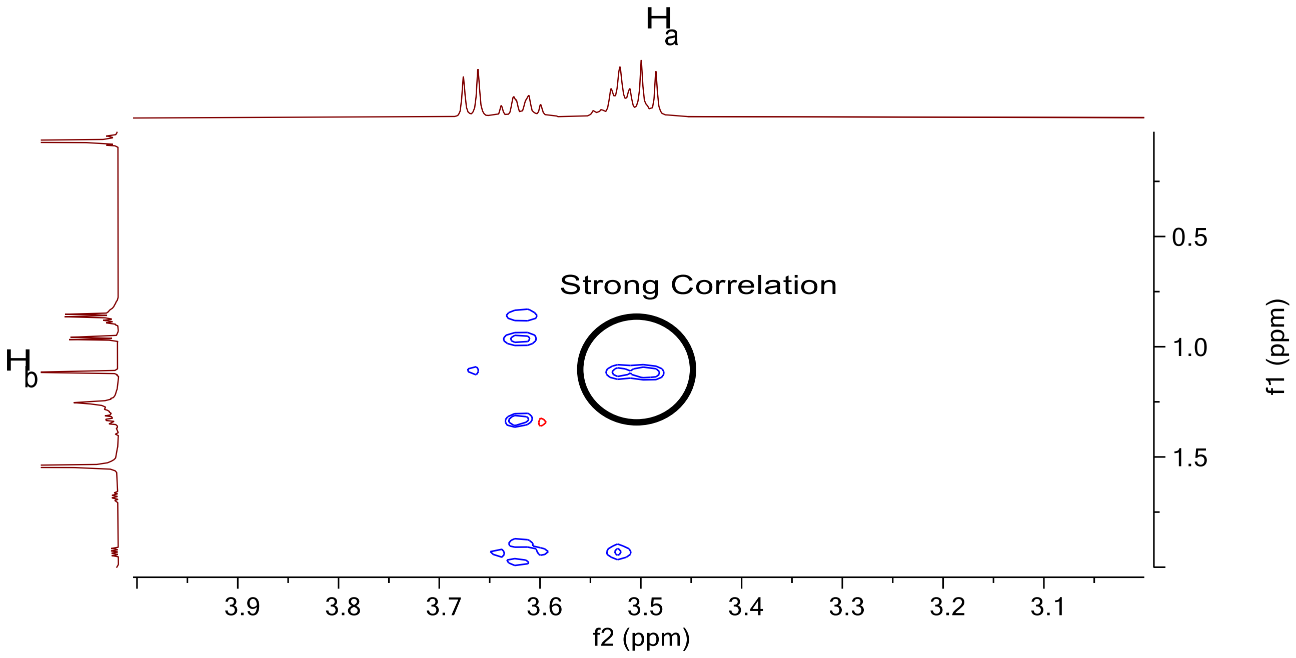

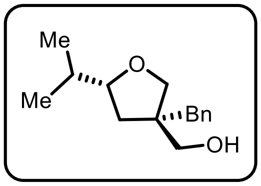

(3-benzyl-5-isopropyltetrahydrofuran-3-yl)methanol (37b) – Major Diastereomer

^{1}H NMR (600 MHz, $CDCl_3$)

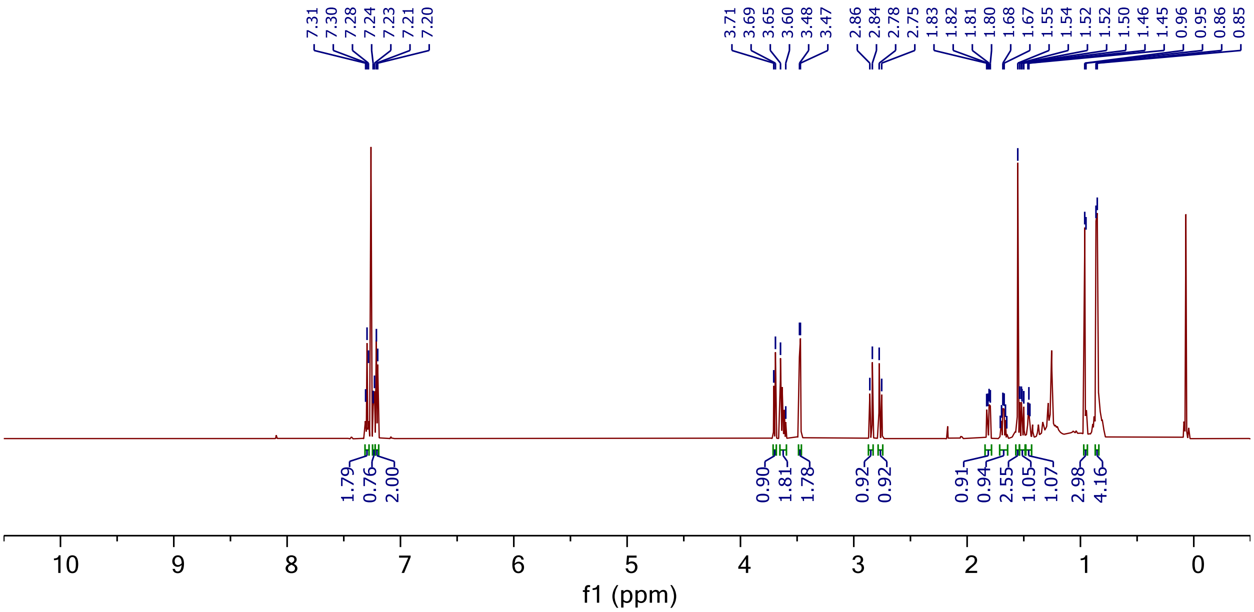

^{13}C NMR (151 MHz, $CDCl_3$)

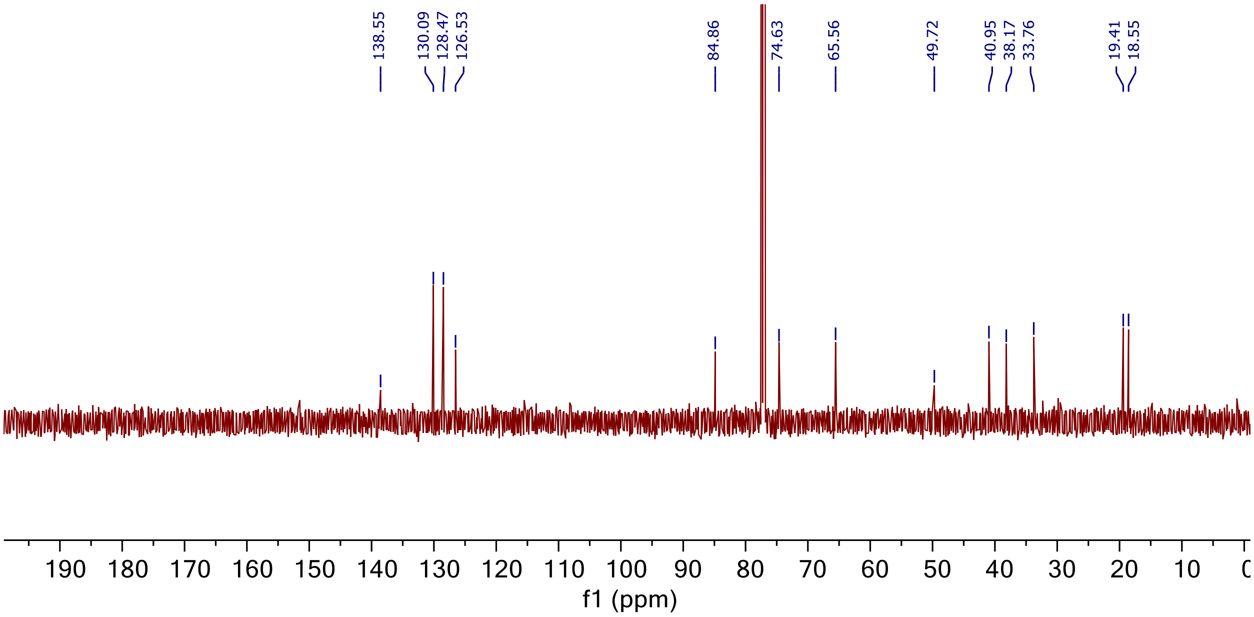

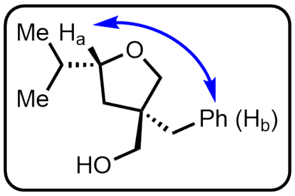

NOESY (600 MHz, $CDCl_3$):
Major Diastereomer

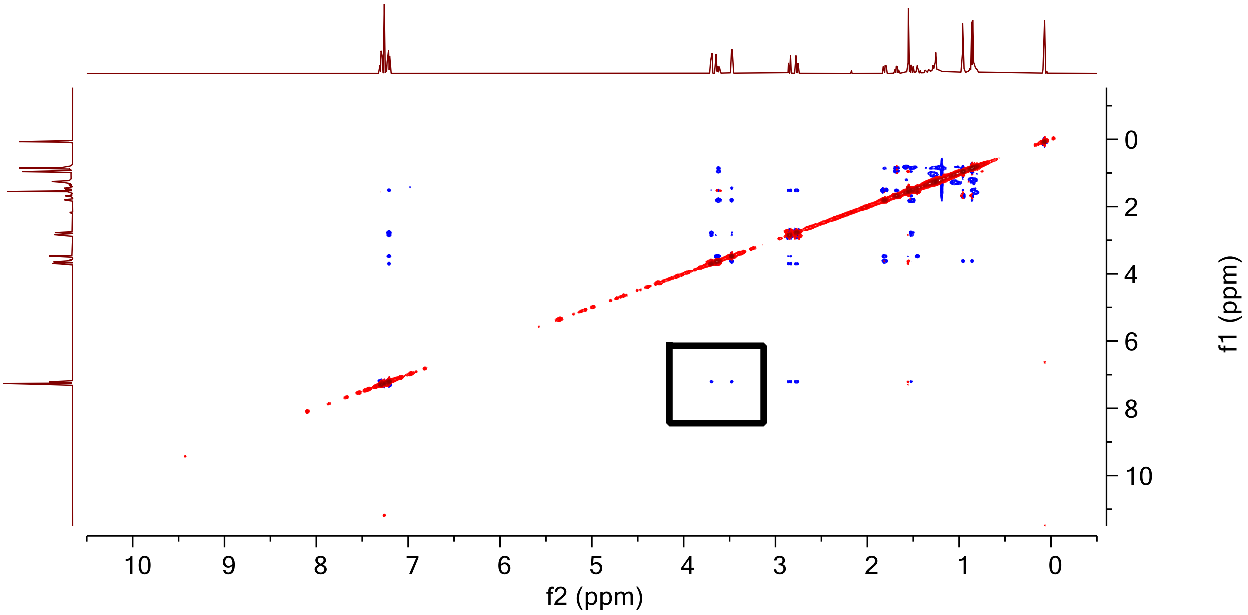

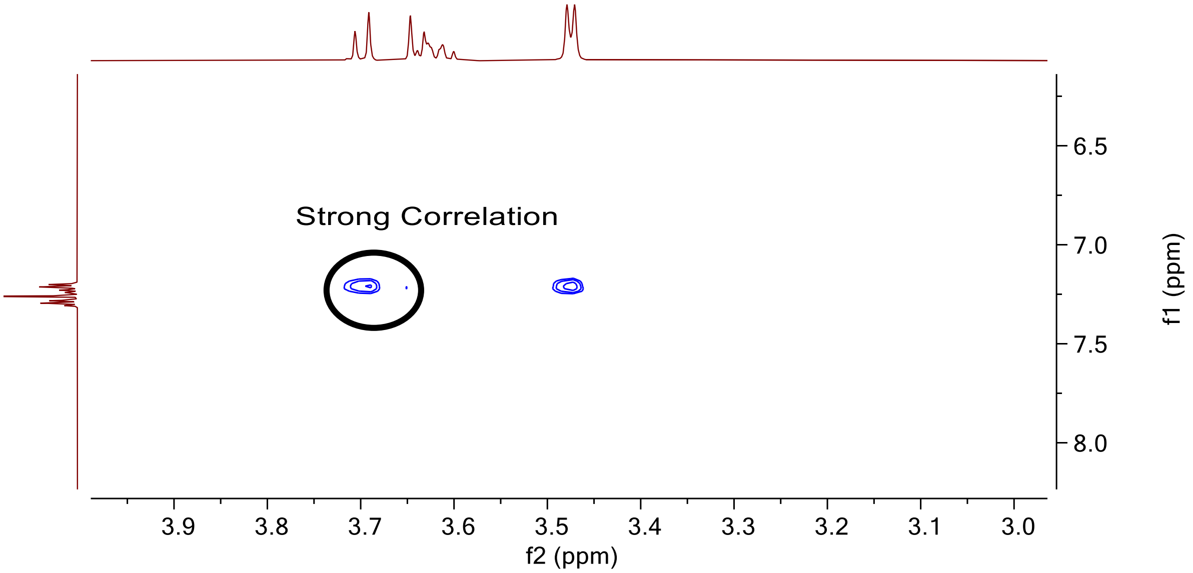

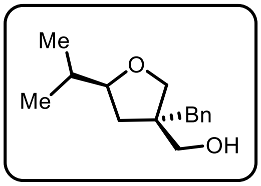

(3-benzyl-5-isopropyltetrahydrofuran-3-yl)methanol (37b)
Minor Diastereomer

^{1}H NMR (600 MHz, $CDCl_3$)

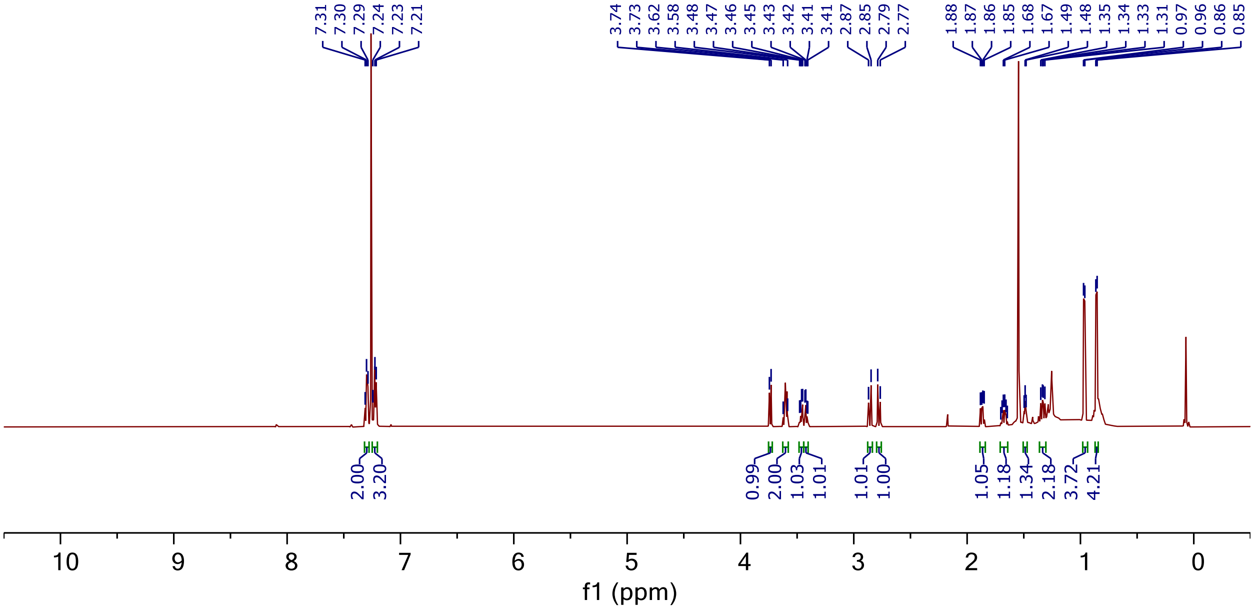

^{13}C NMR (151 MHz, $CDCl_3$)

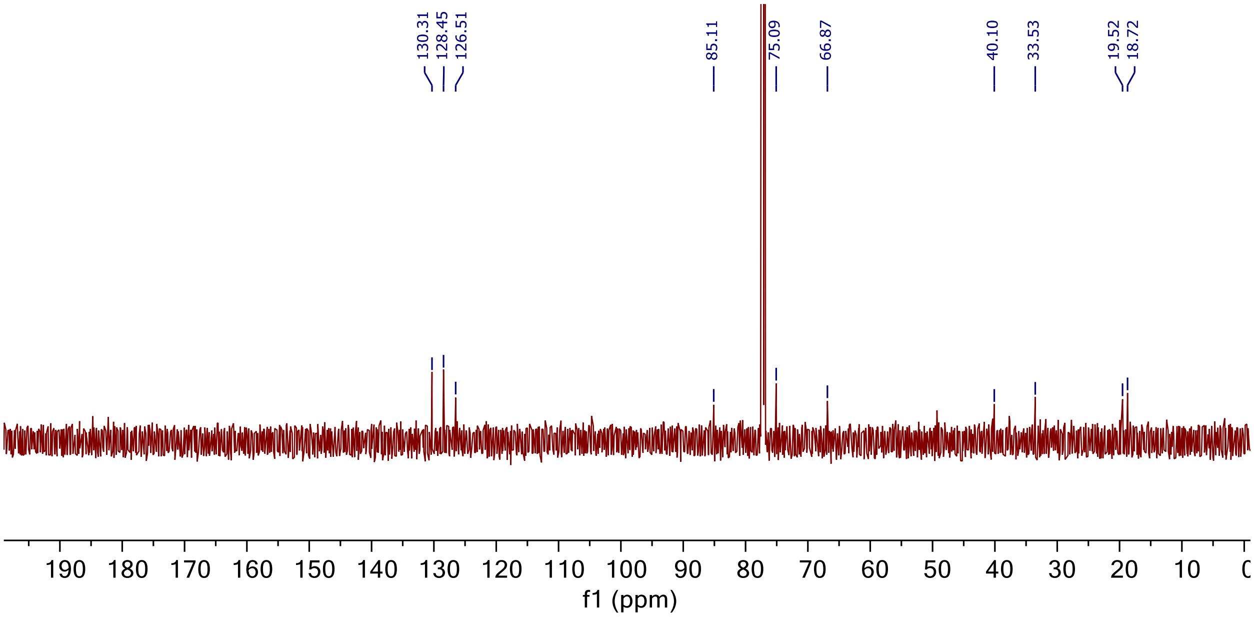

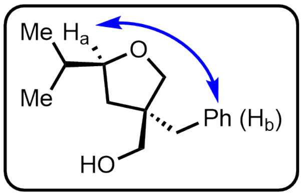

NOESY (600 MHz, $CDCl_3$):
Minor Diastereomer

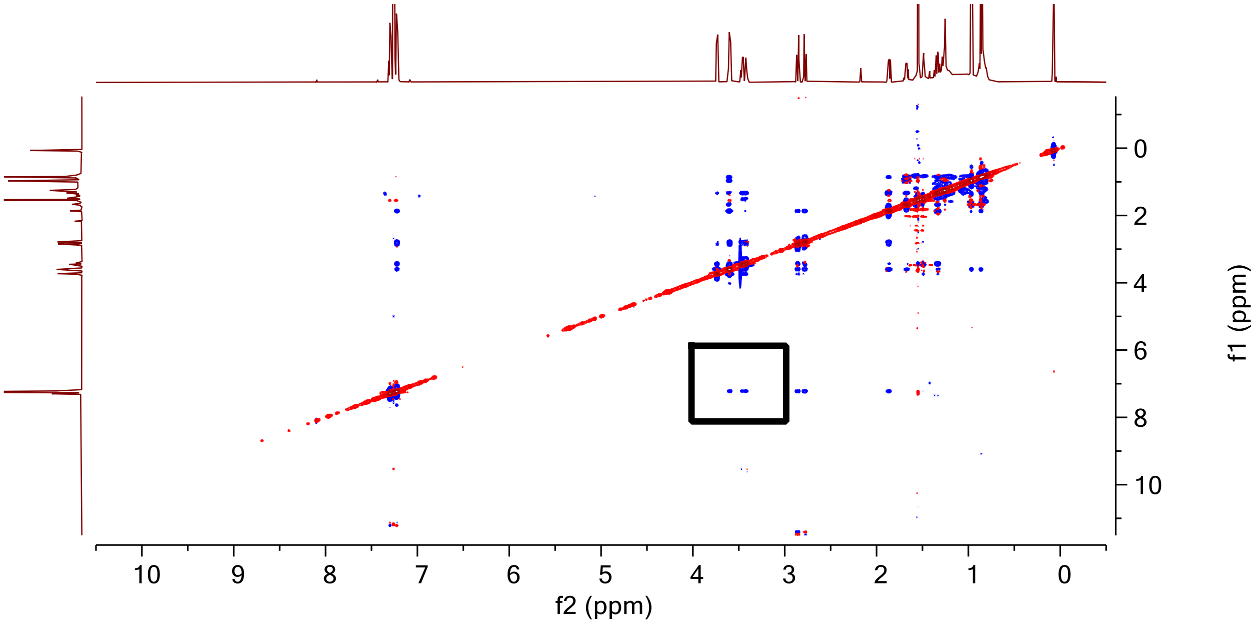

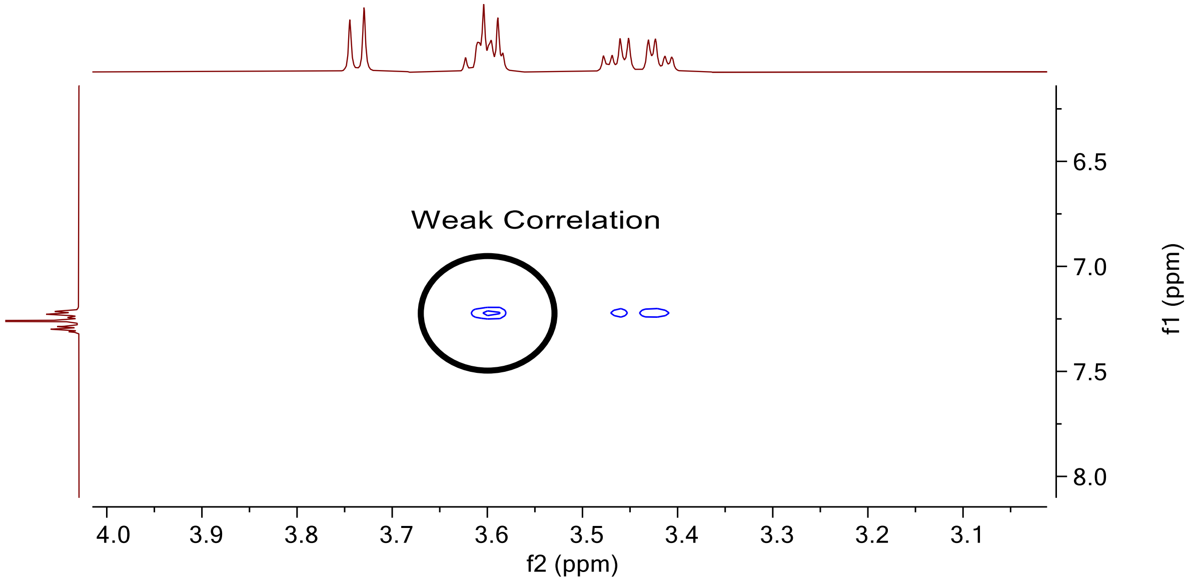

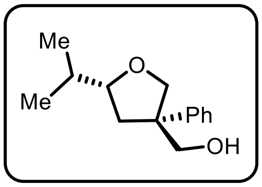

(5-isopropyl-3-phenyltetrahydrofuran-3-yl)methanol (38b)
Major Diastereomer

^{1}H NMR (600 MHz, $CDCl_3$)

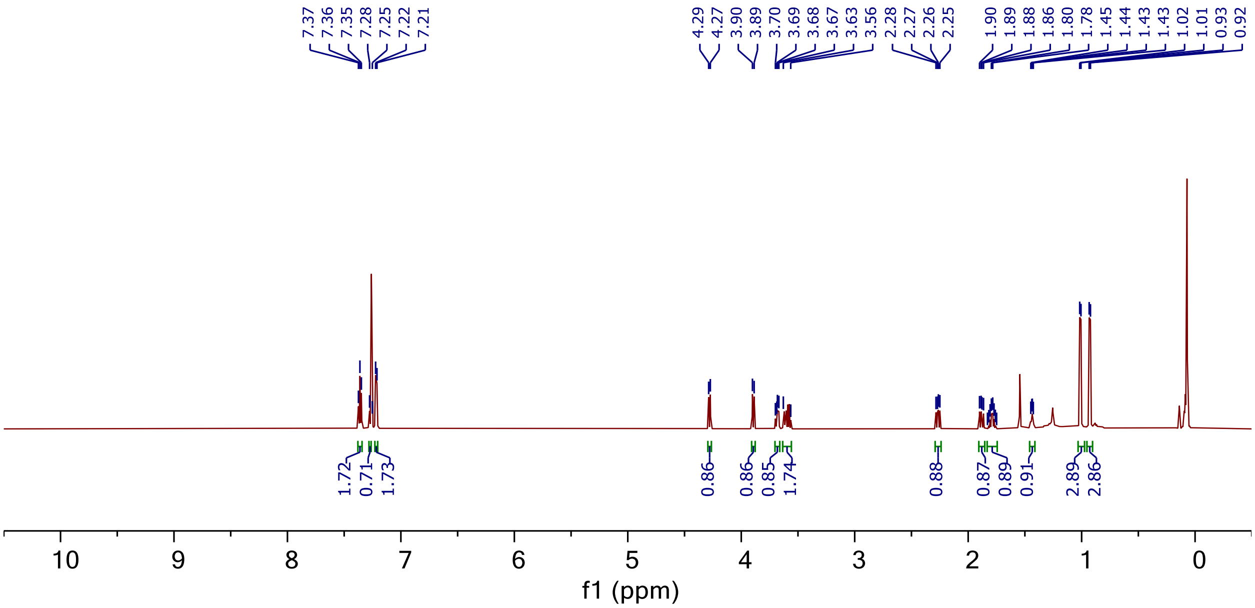

^{13}C NMR (151 MHz, $CDCl_3$)

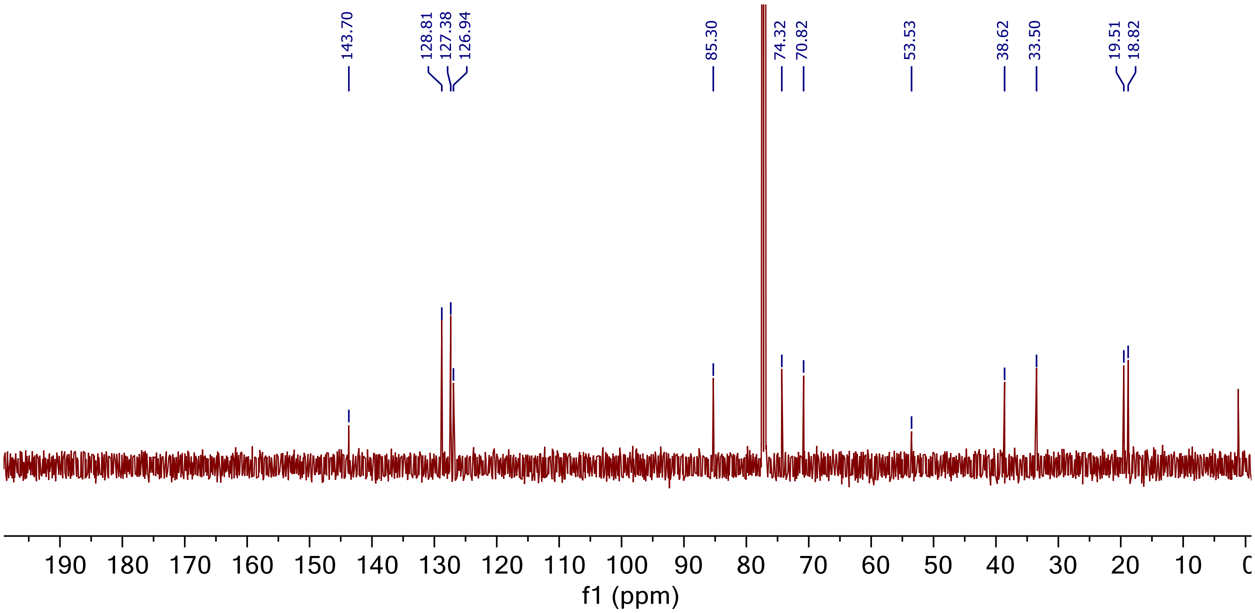

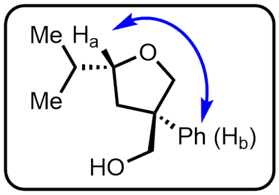

NOESY (600 MHz, $CDCl_3$):
Major Diastereomer

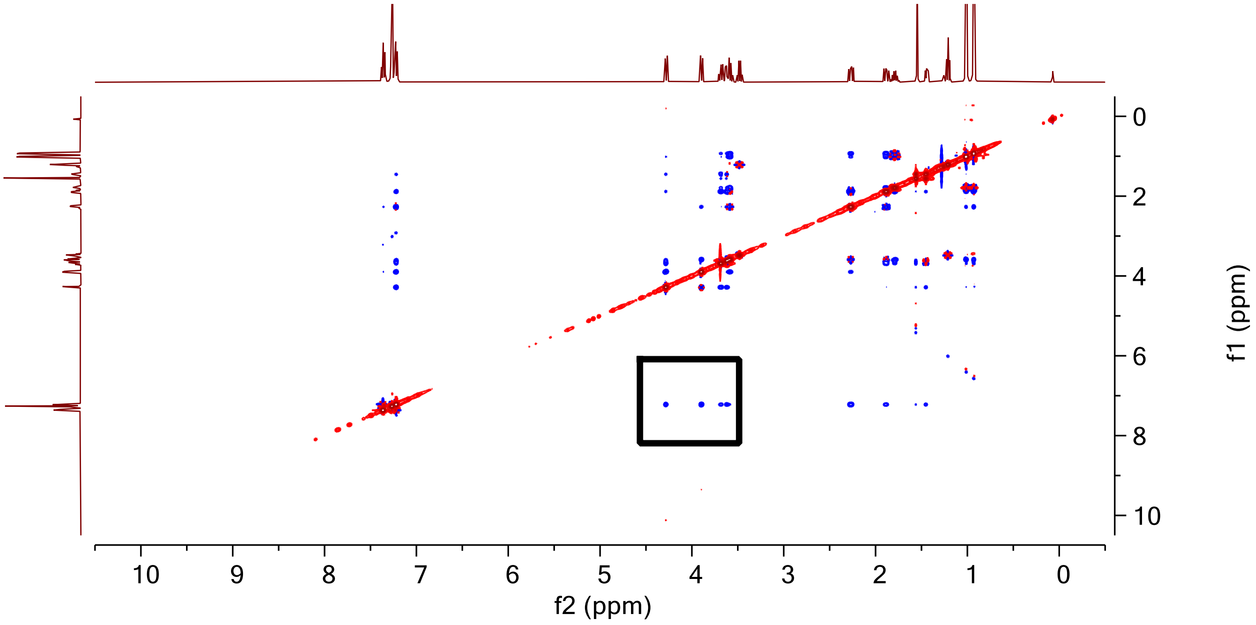

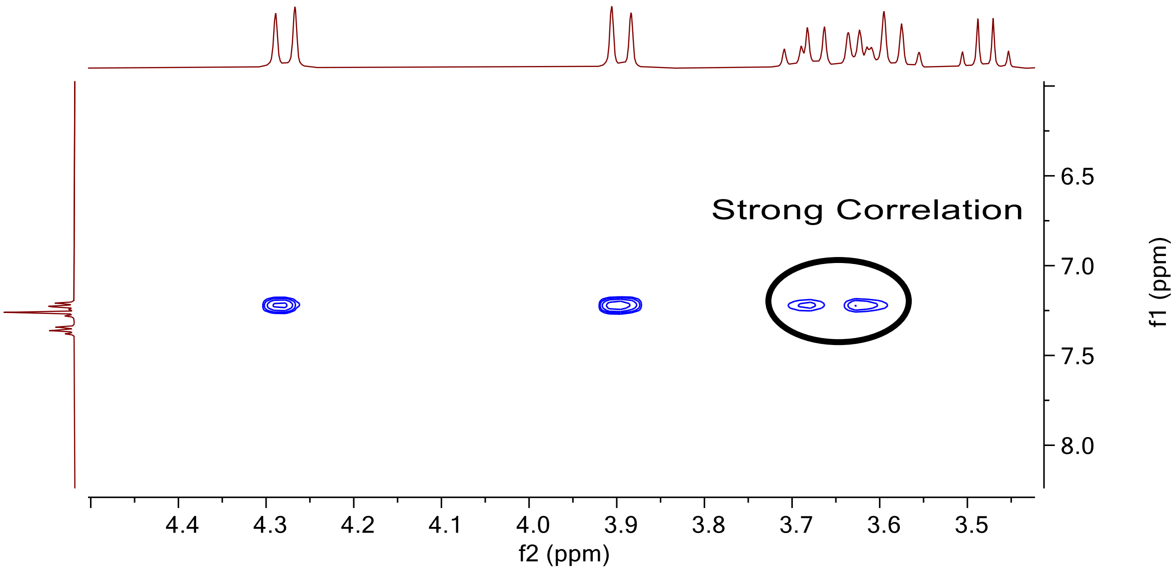

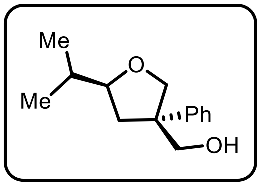

(5-isopropyl-3-phenyltetrahydrofuran-3-yl)methanol (38b)
Minor Diastereomer

^{1}H NMR (600 MHz, $CDCl_3$)

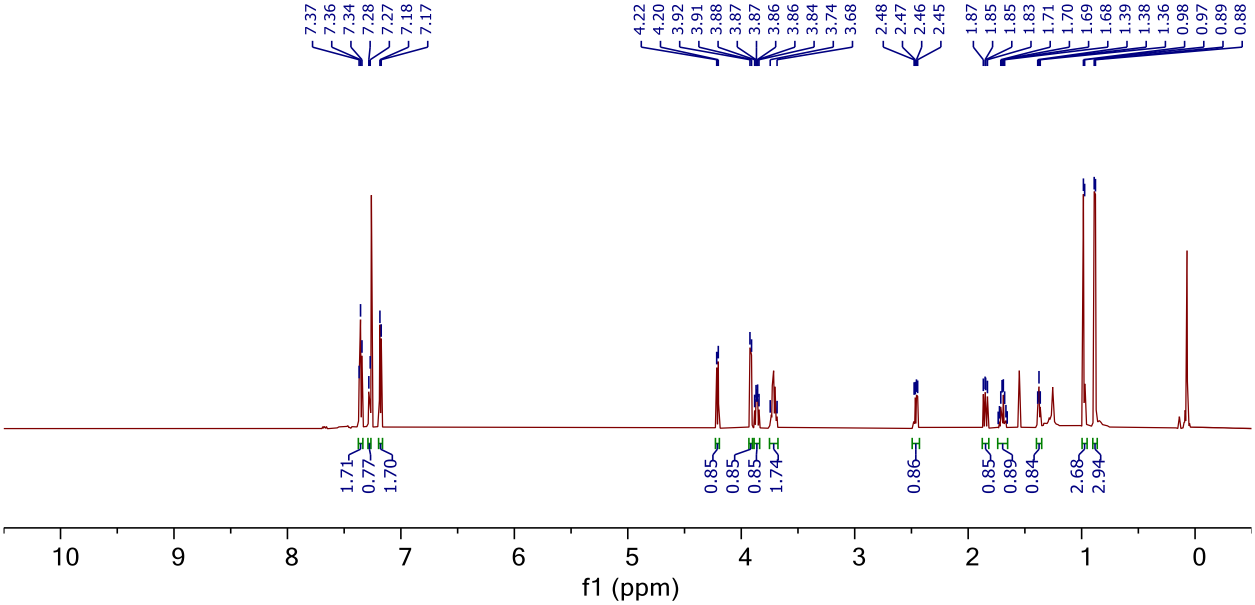

^{13}C NMR (151 MHz, $CDCl_3$)

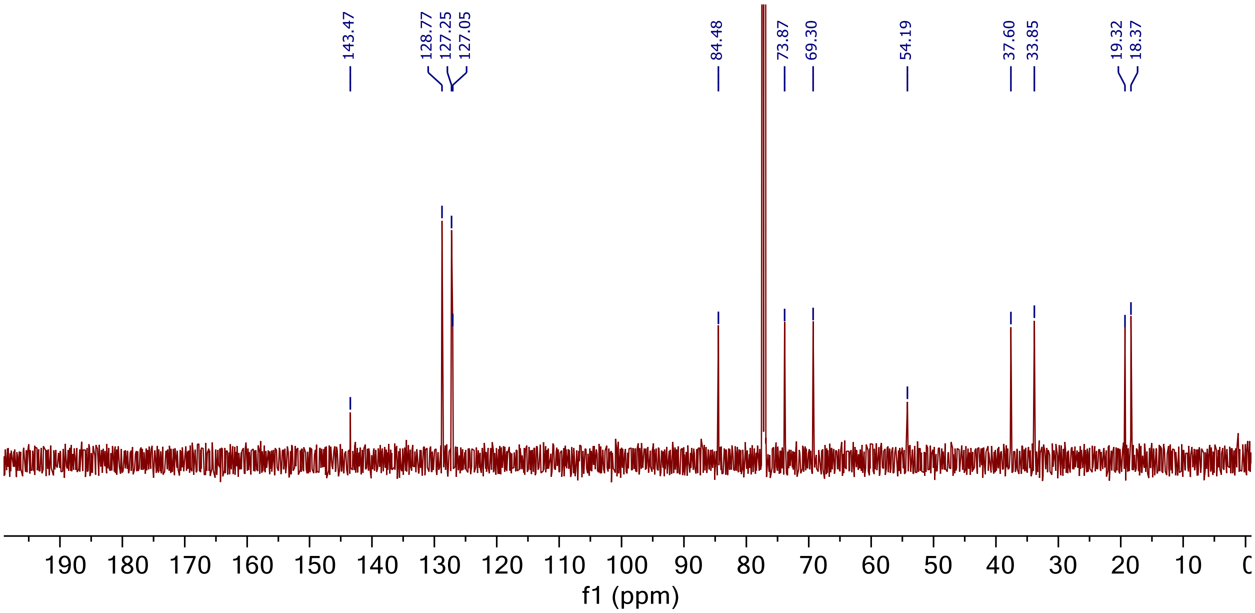

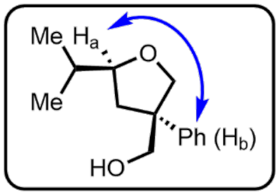

NOESY (600 MHz, $CDCl_3$):
Minor Diastereomer

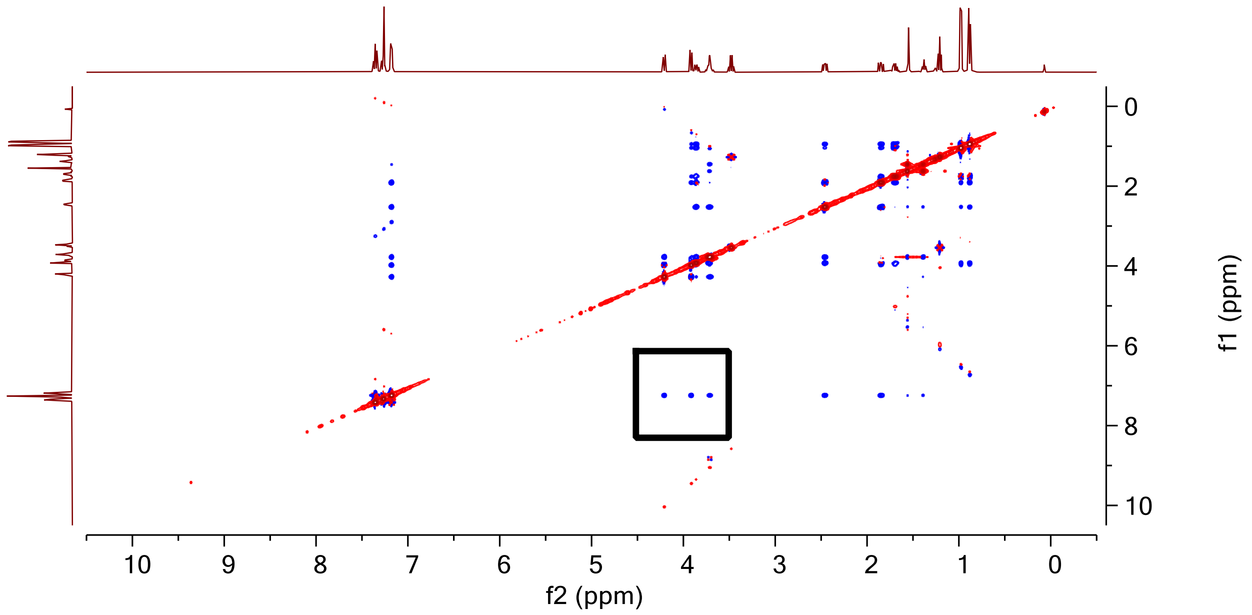

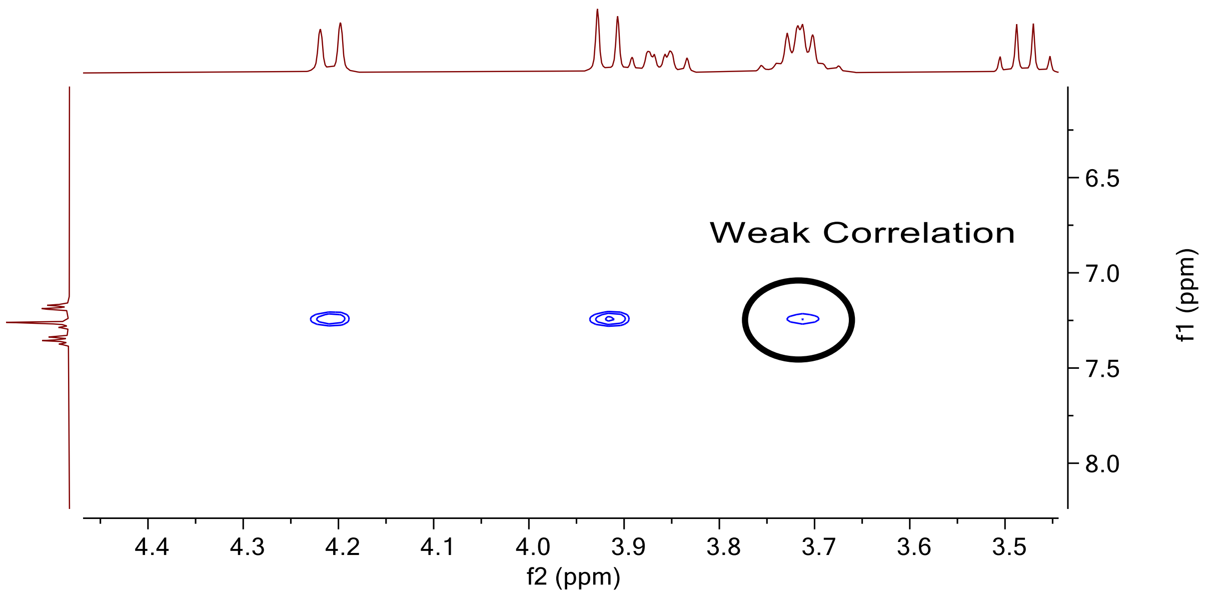

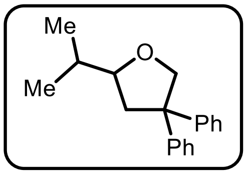

2-isopropyl-4,4-diphenyltetrahydrofuran (39b)

^{1}H NMR (600 MHz, $CDCl_3$)

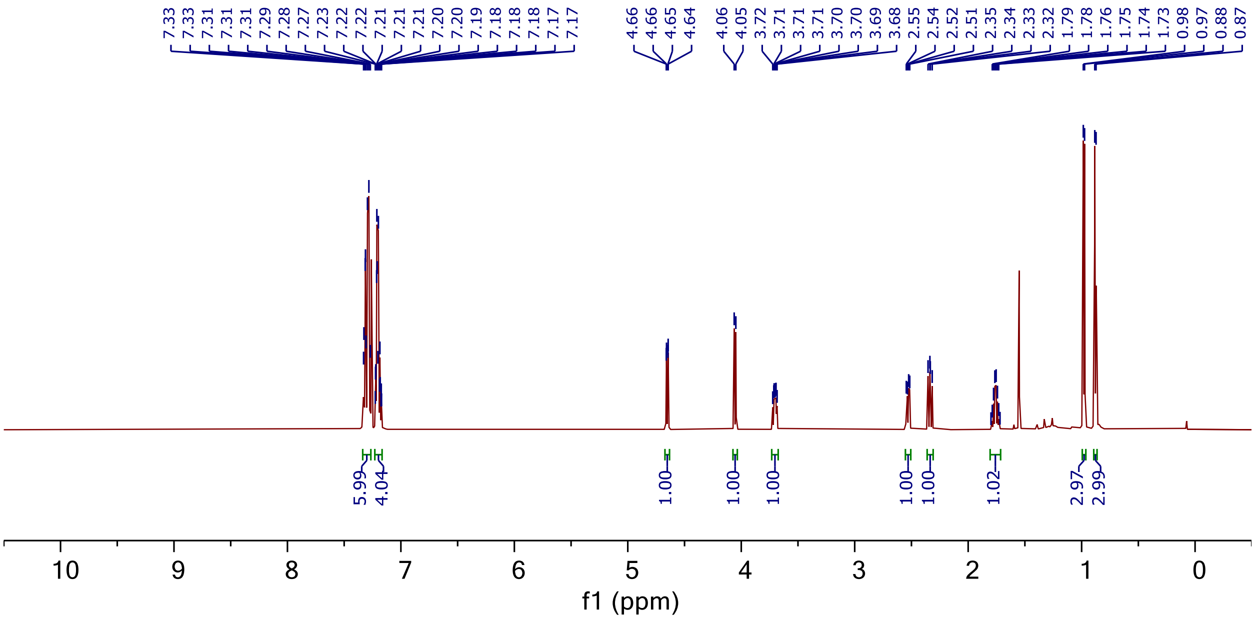

^{13}C NMR (151 MHz, $CDCl_3$)

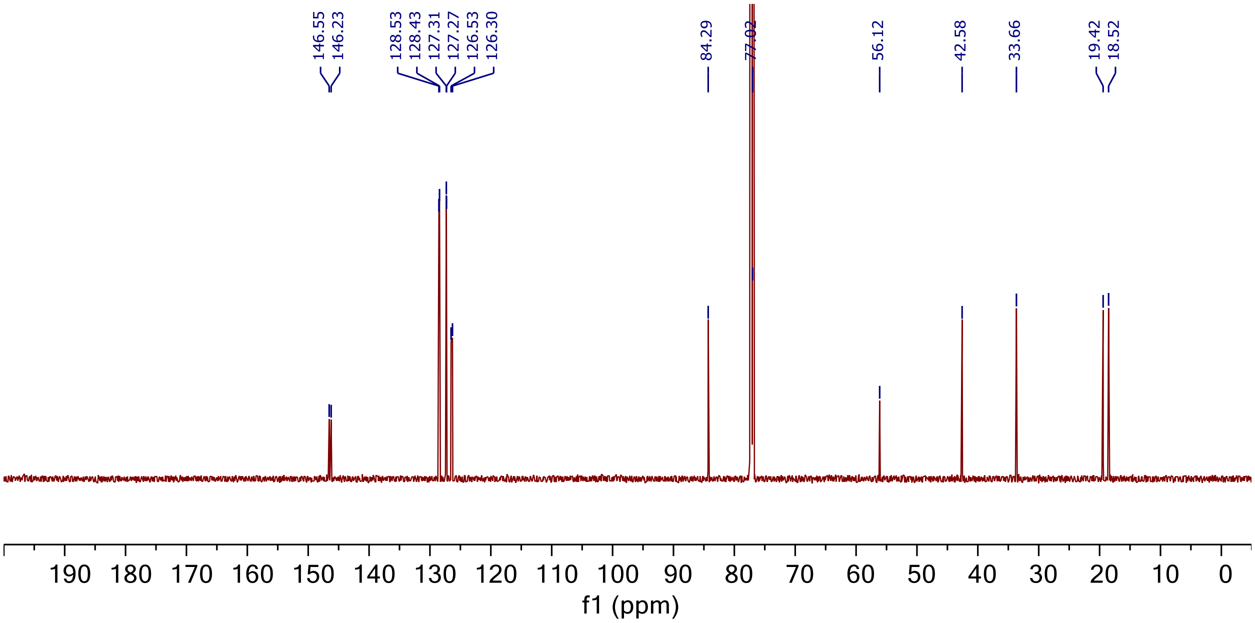

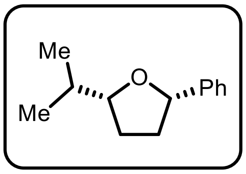

2-isopropyl-5-phenyltetrahydrofuran (40b).

^{1}H NMR (600 MHz, $CDCl_3$)

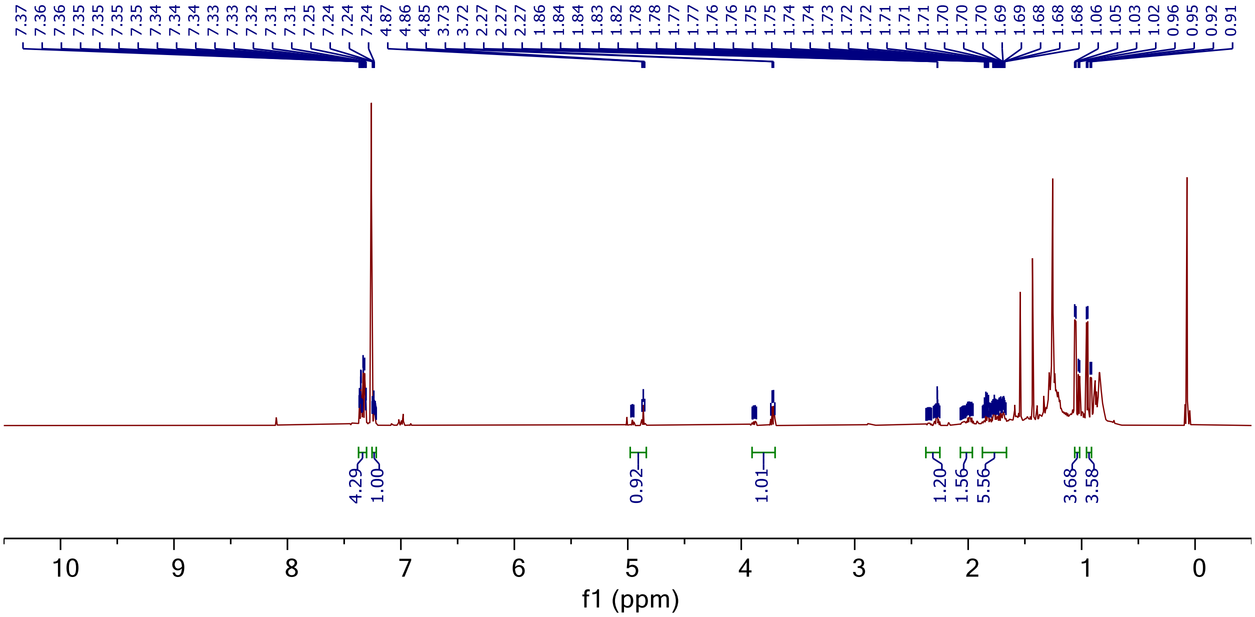

^{13}C NMR (151 MHz, $CDCl_3$)

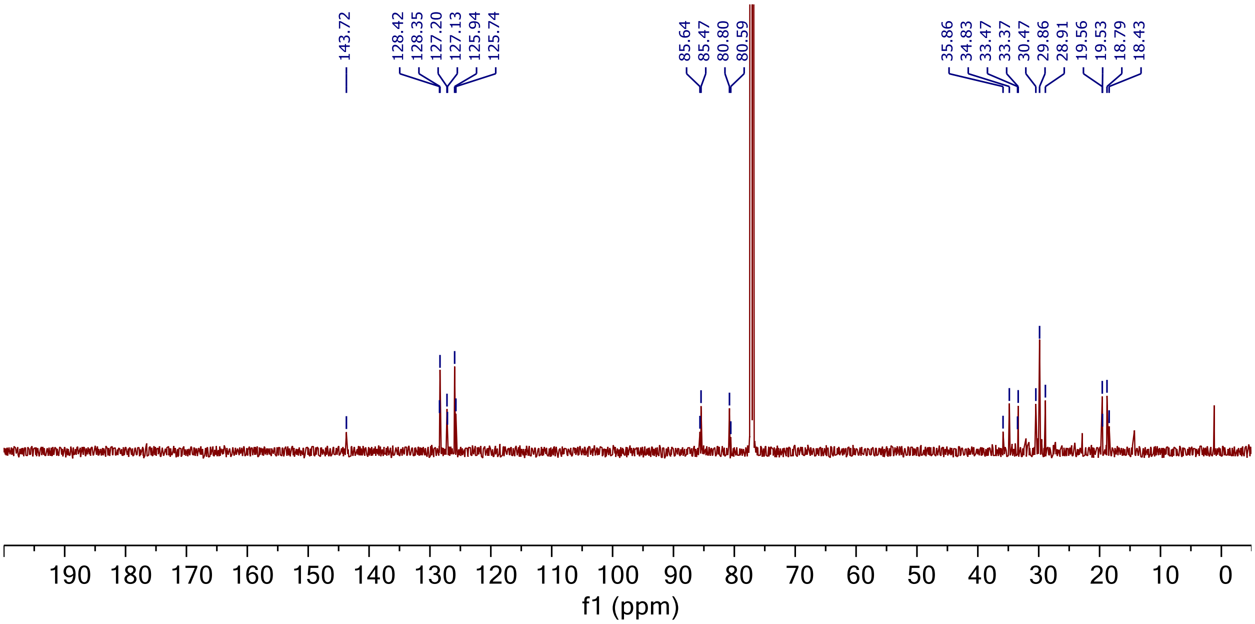

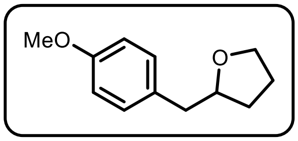

2-(4-methoxybenzyl)tetrahydrofuran (41b)

^{1}H NMR (600 MHz, $CDCl_3$)

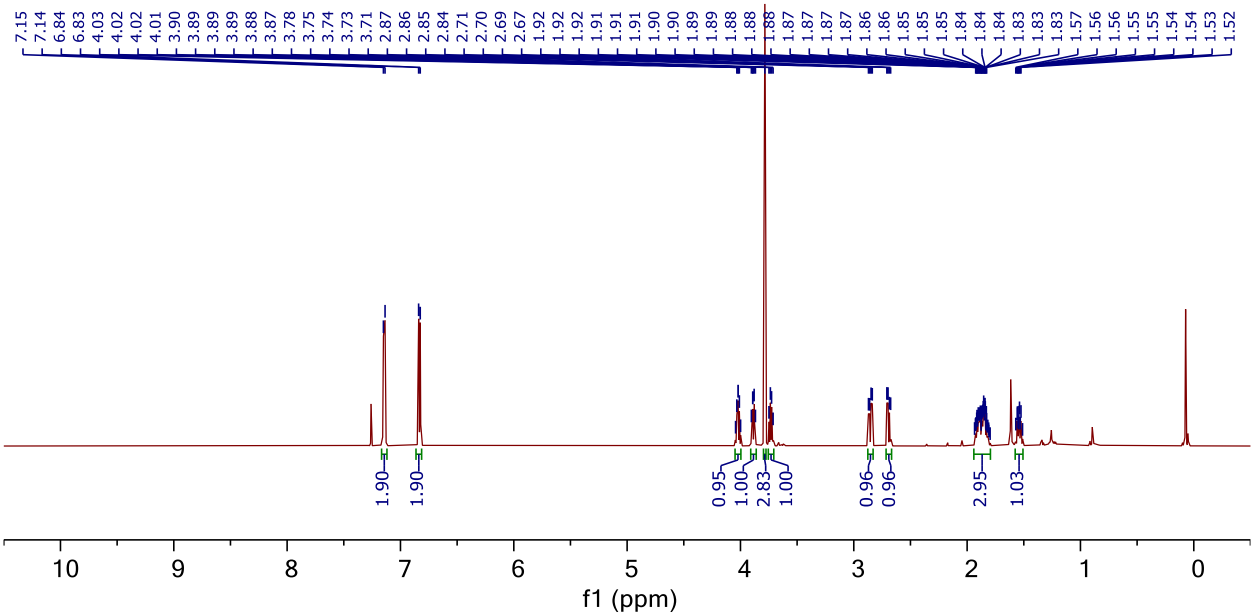

^{13}C NMR (151 MHz, $CDCl_3$)

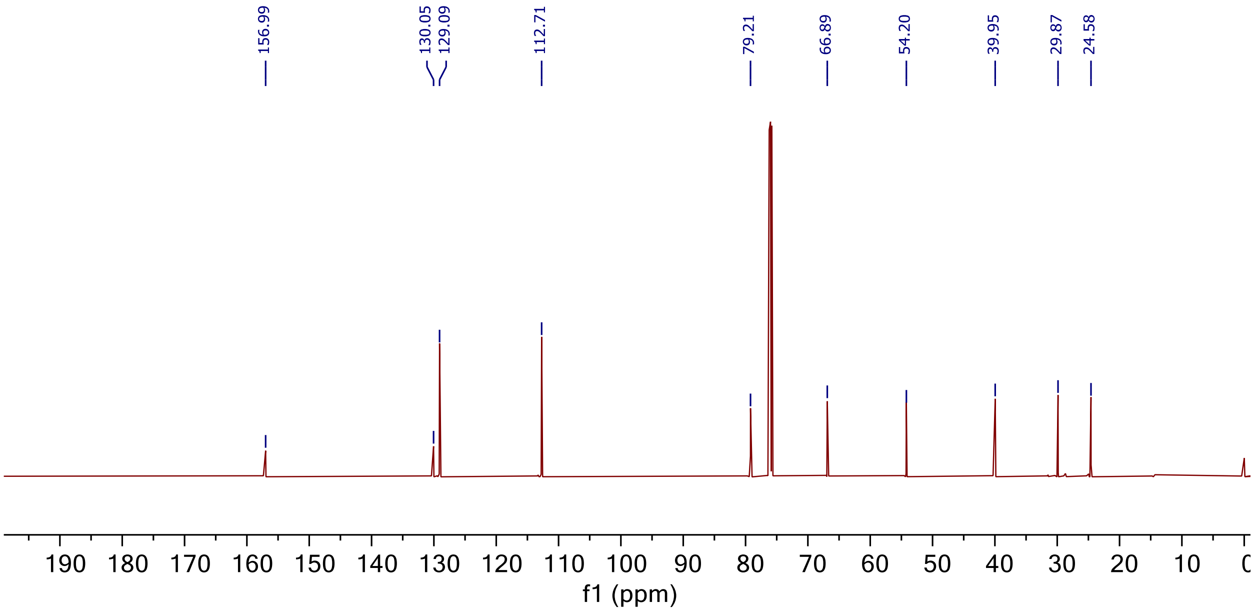

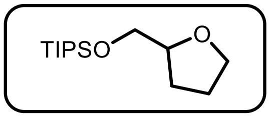

triisopropyl((tetrahydrofuran-2-yl)methoxy)silane (42b)

^{1}H NMR (600 MHz, $CDCl_3$)

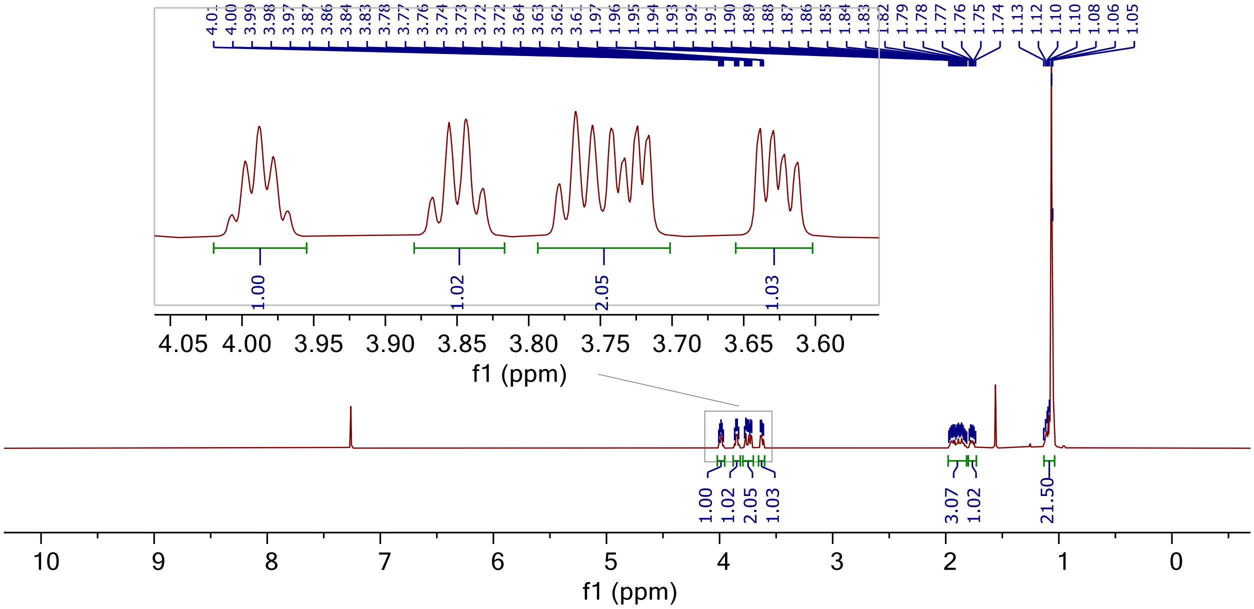

^{13}C NMR (151 MHz, $CDCl_3$)

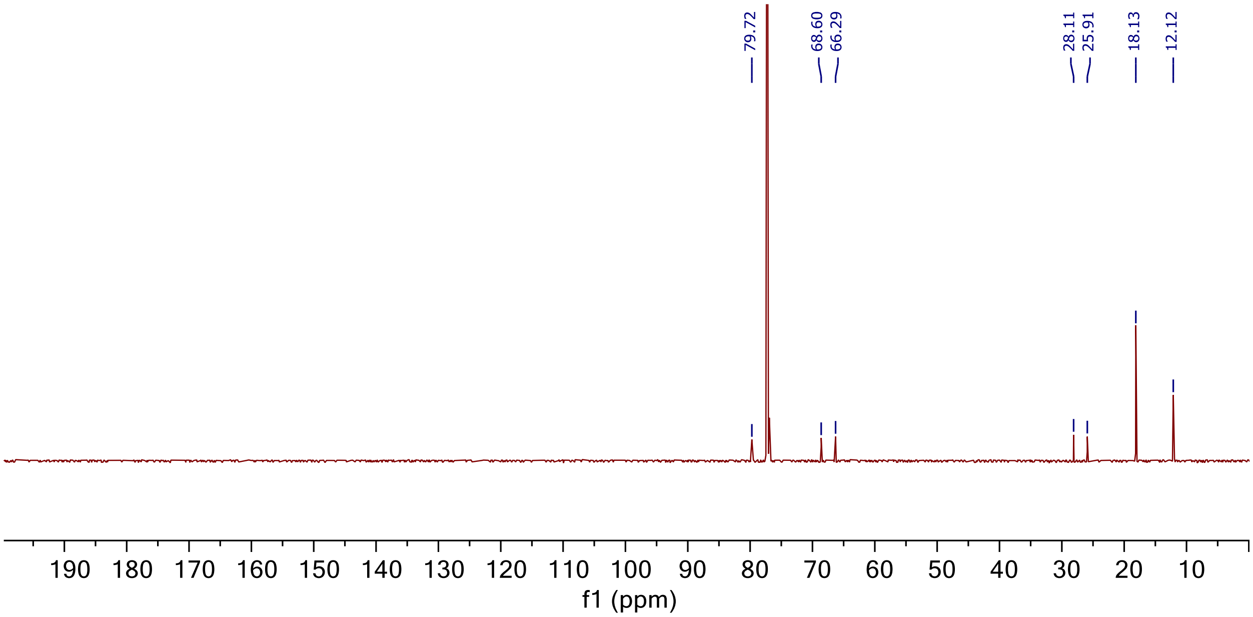

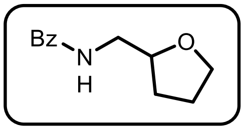

N-((tetrahydrofuran-2-yl)methyl)benzamide (43b)

^{1}H NMR (600 MHz, $CDCl_3$)

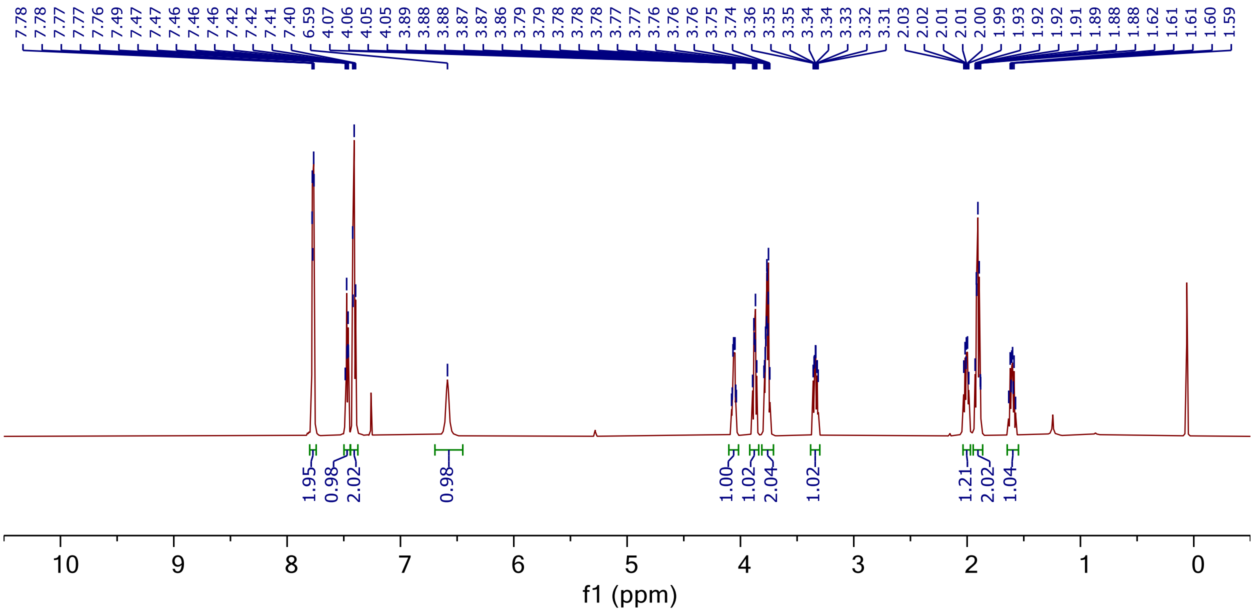

^{13}C NMR (151 MHz, $CDCl_3$):

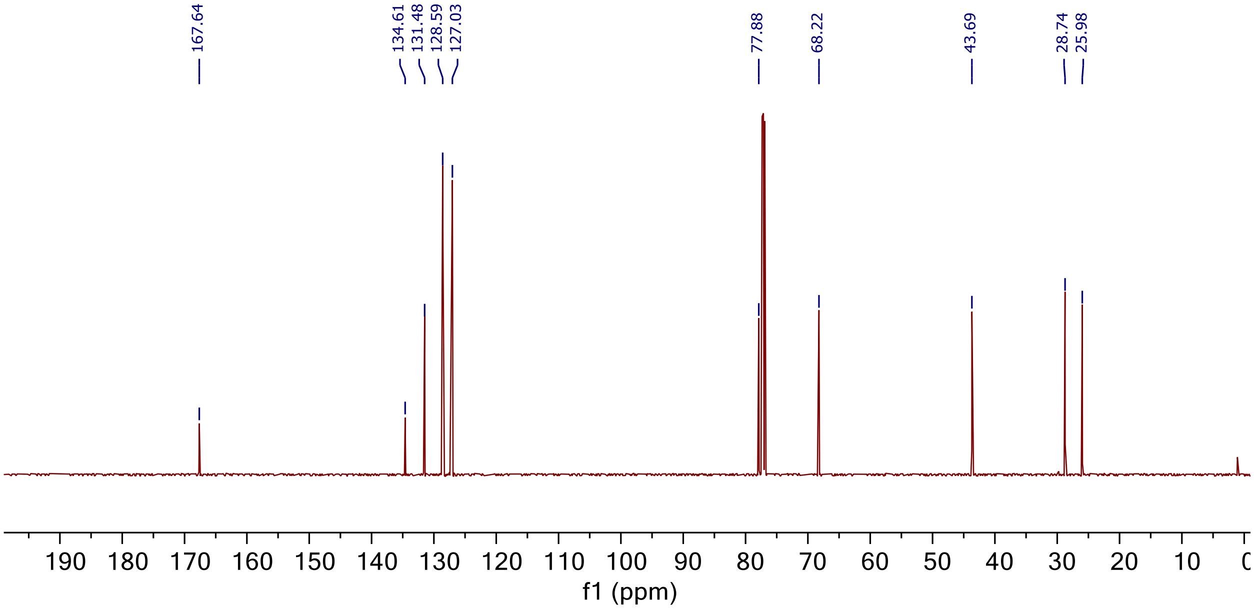

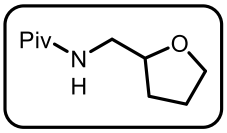

tert-butyl((tetrahydrofuran-2-yl)methyl)carbamate (44b)

^{1}H NMR (600 MHz, $CDCl_3$)

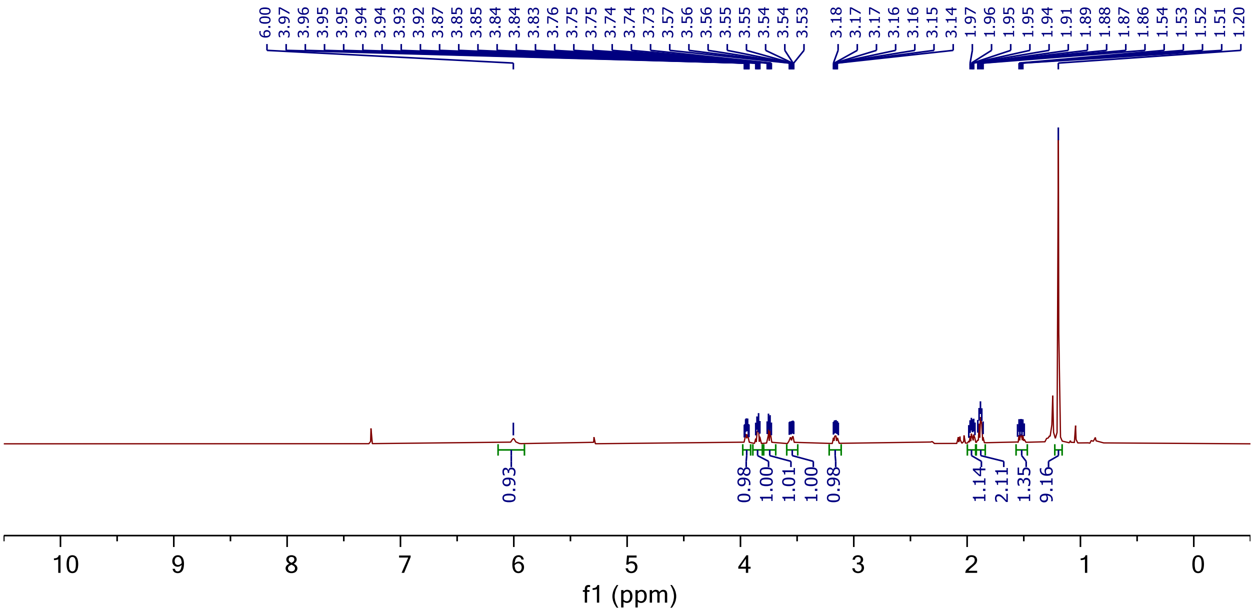

^{13}C NMR (151 MHz, $CDCl_3$)

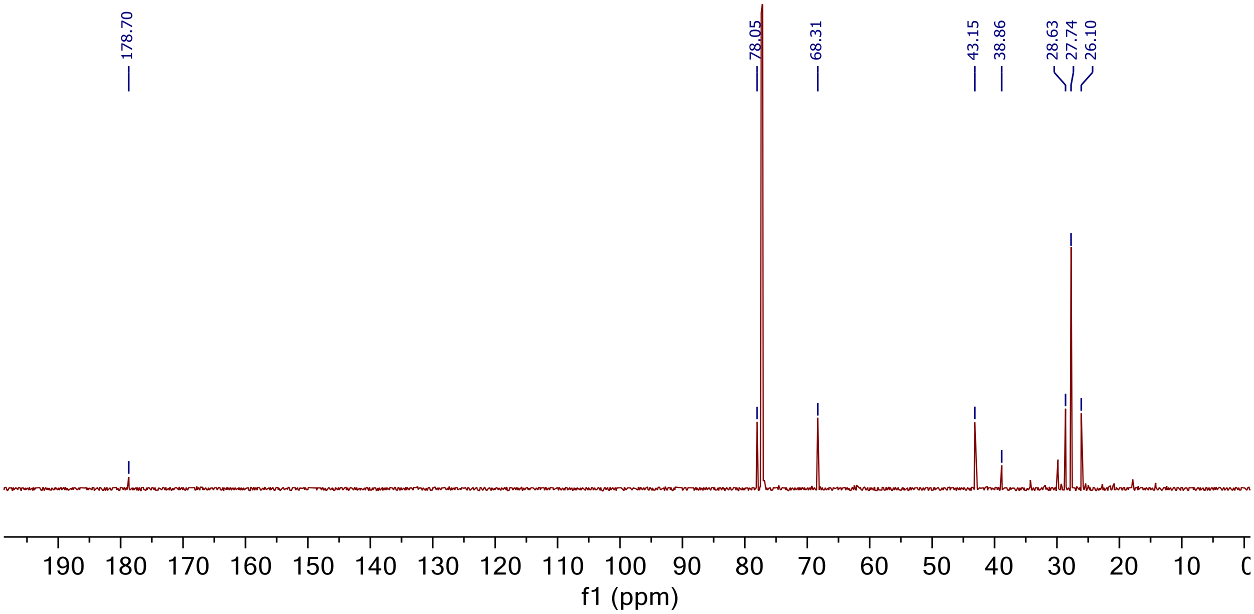

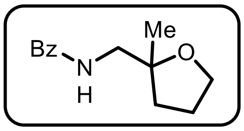

N-((2-methyltetrahydrofuran-2-yl)methyl)benzamide (45b)

1H NMR (600 MHz, $CDCl_3$)

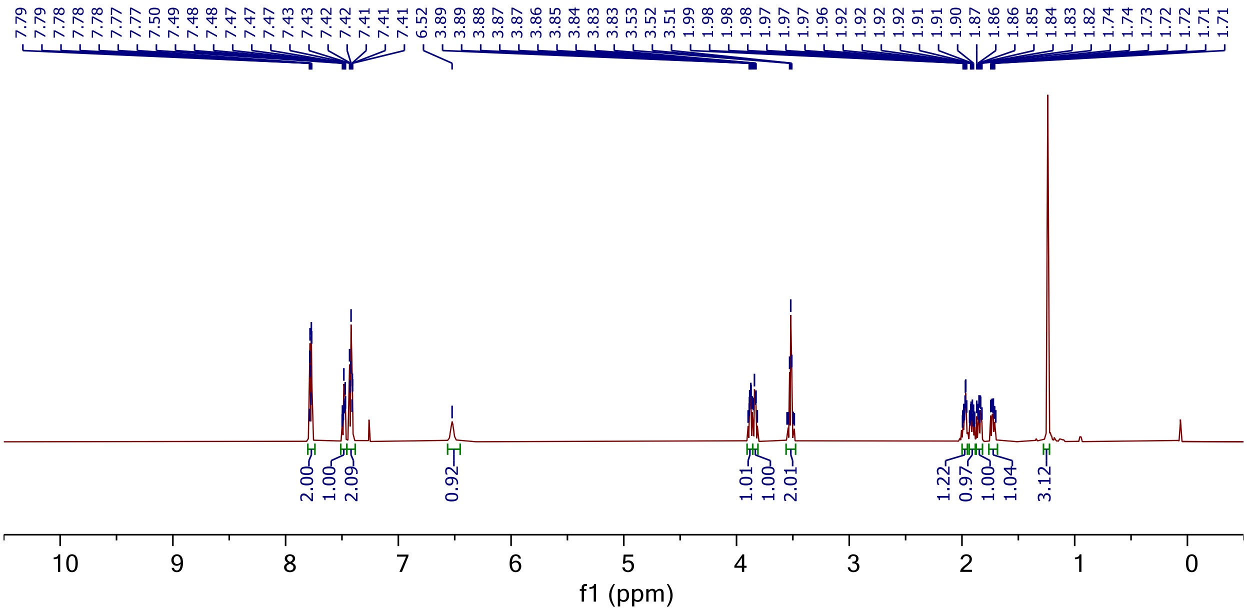

13C NMR (151 MHz, $CDCl_3$)

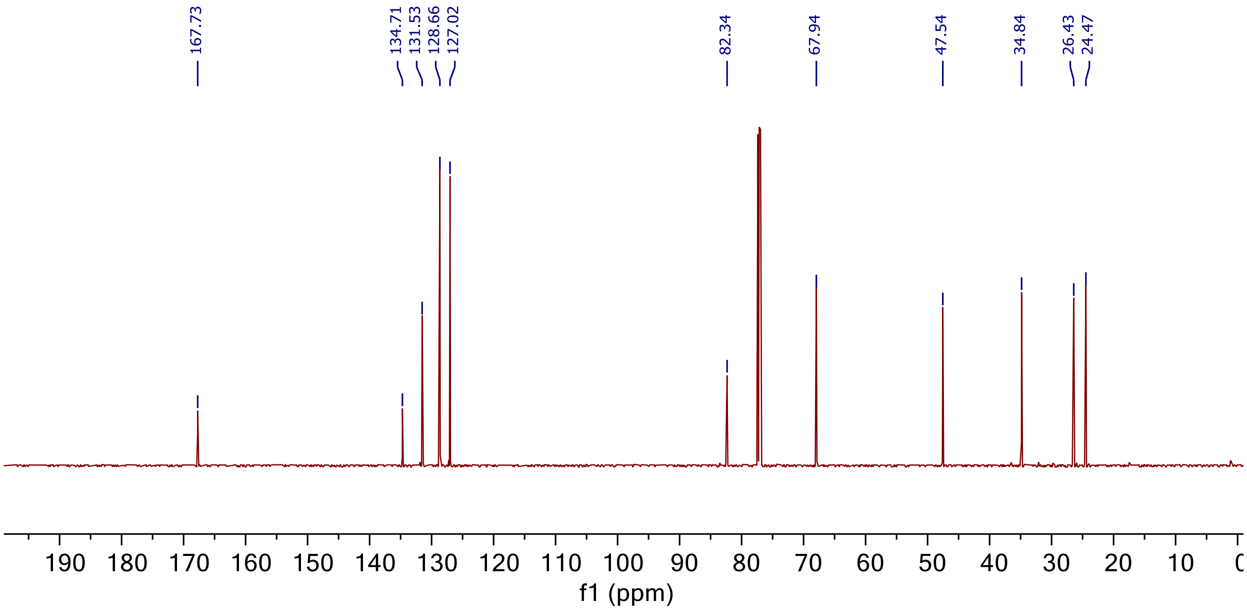

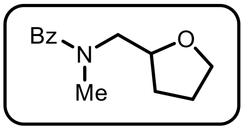

N-methyl-N-((tetrahydrofuran-2-yl)methyl)benzamide (46b)

^{1}H NMR (500 MHz, DMSO-d_6)

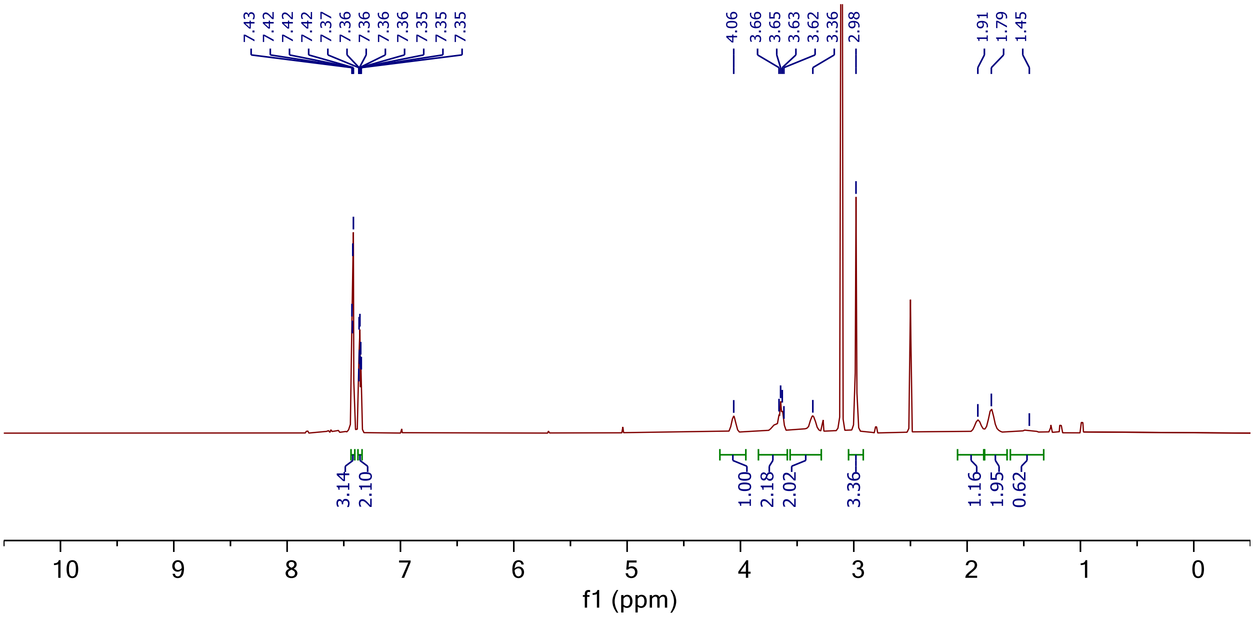

^{13}C NMR (151 MHz, DMSO-d_6)

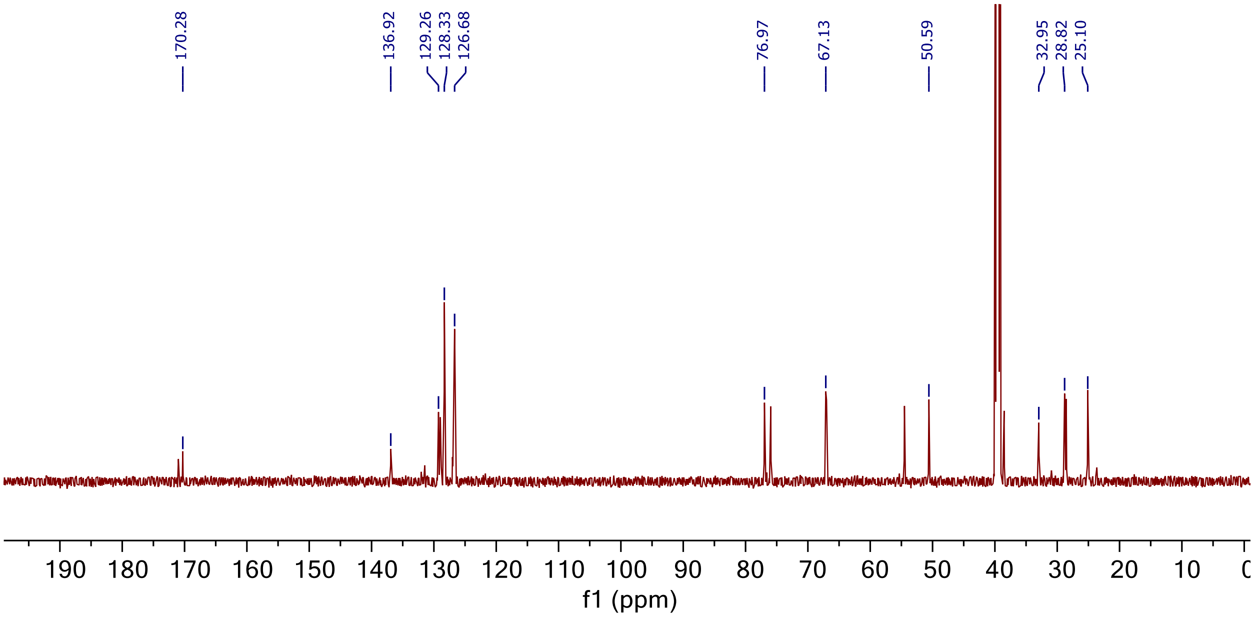

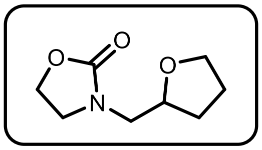

3-((tetrahydrofuran-2-yl)methyl)oxazolidin-2-one (47b)

^{1}H NMR (600 MHz, $CDCl_3$)

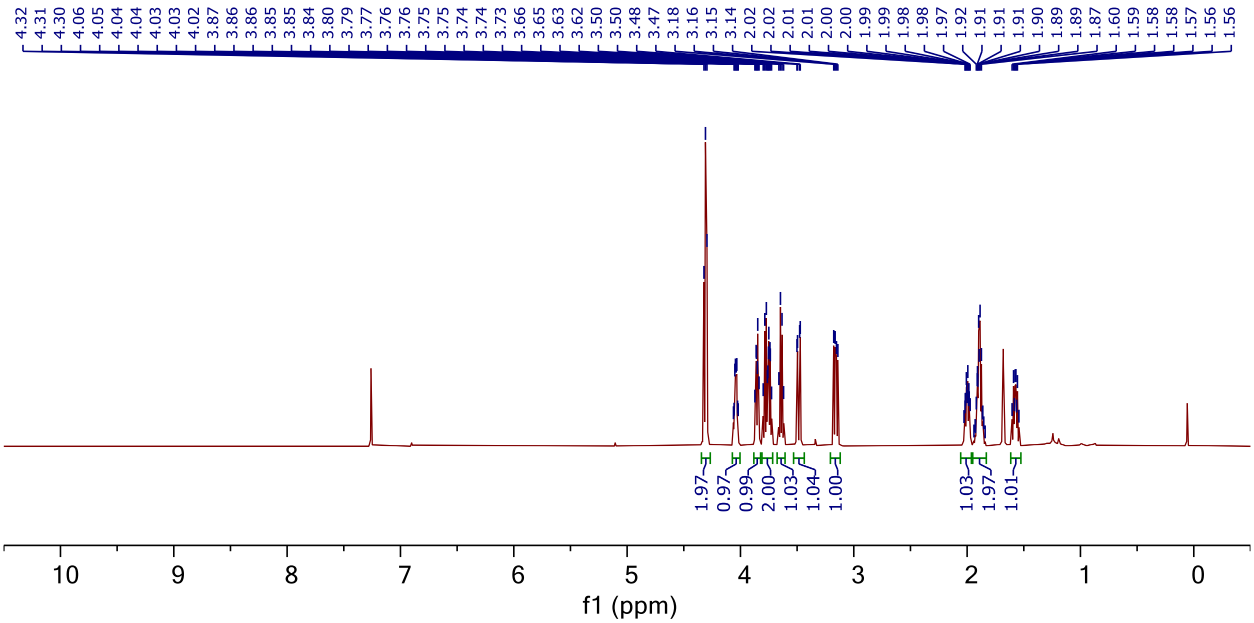

^{13}C NMR (151 MHz, $CDCl_3$)

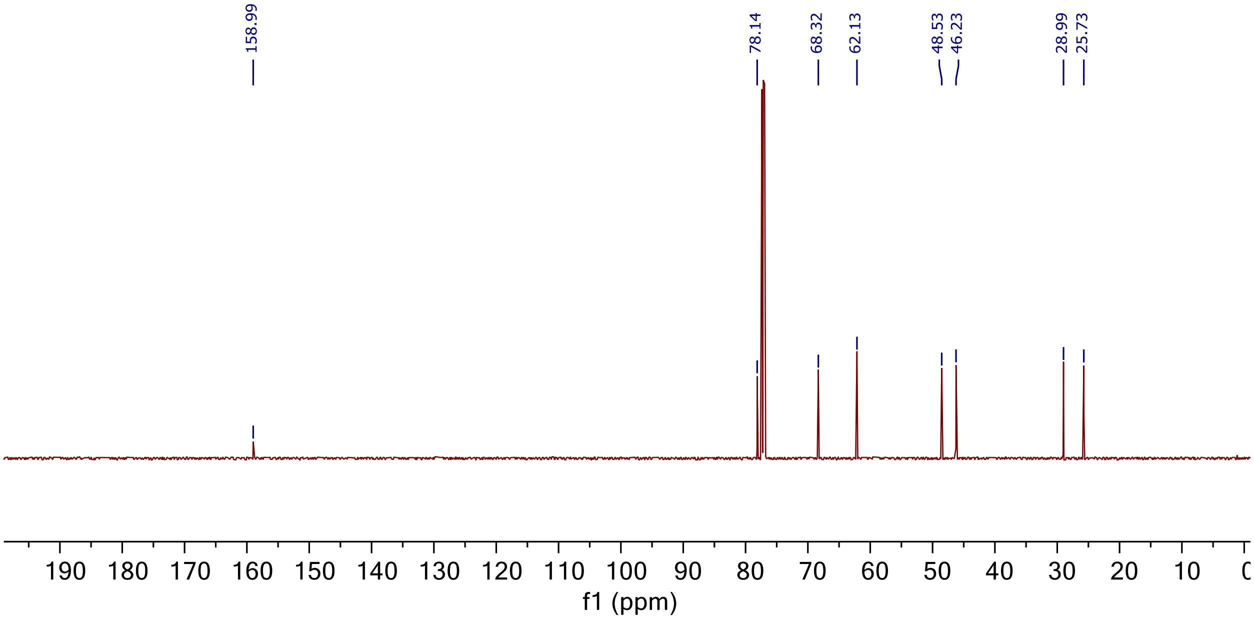

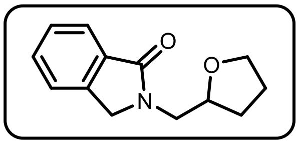

2-((tetrahydrofuran-2-yl)methyl)isoindolin-1-one (48b)

^{1}H NMR (600 MHz, $CDCl_3$)

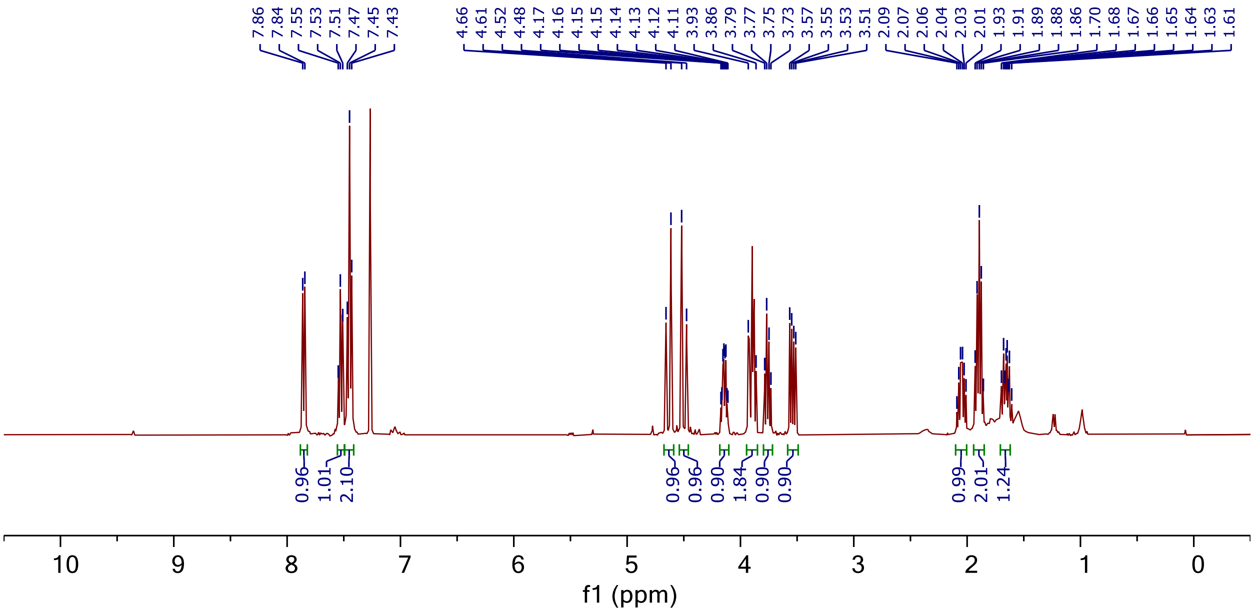

^{13}C NMR (151 MHz, $CDCl_3$)

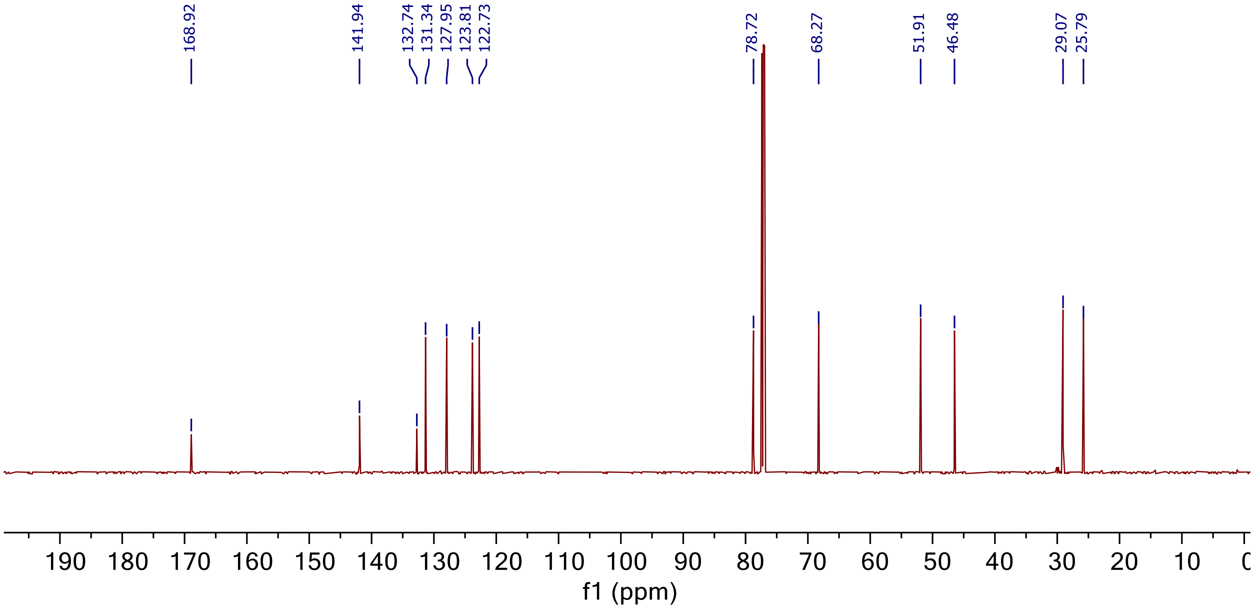

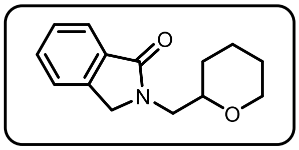

2-((tetrahydro-2H-pyran-2-yl)methyl)isoindolin-1-one (49b)

^{1}H NMR (600 MHz, $CDCl_3$)

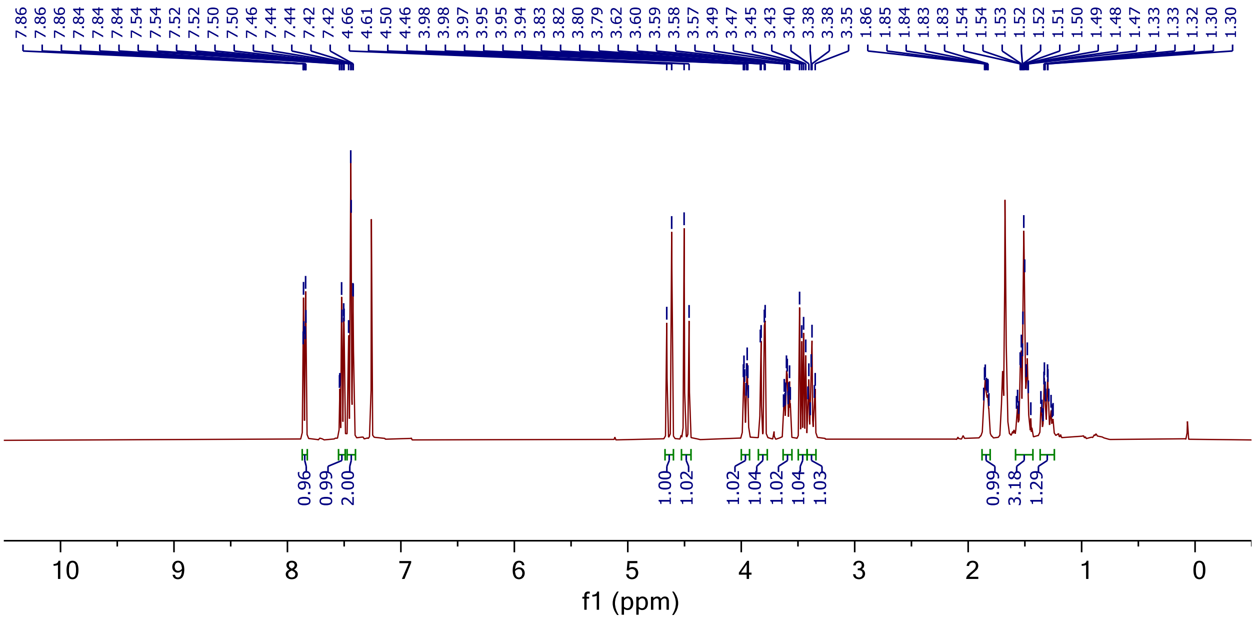

^{13}C NMR (151 MHz, $CDCl_3$)

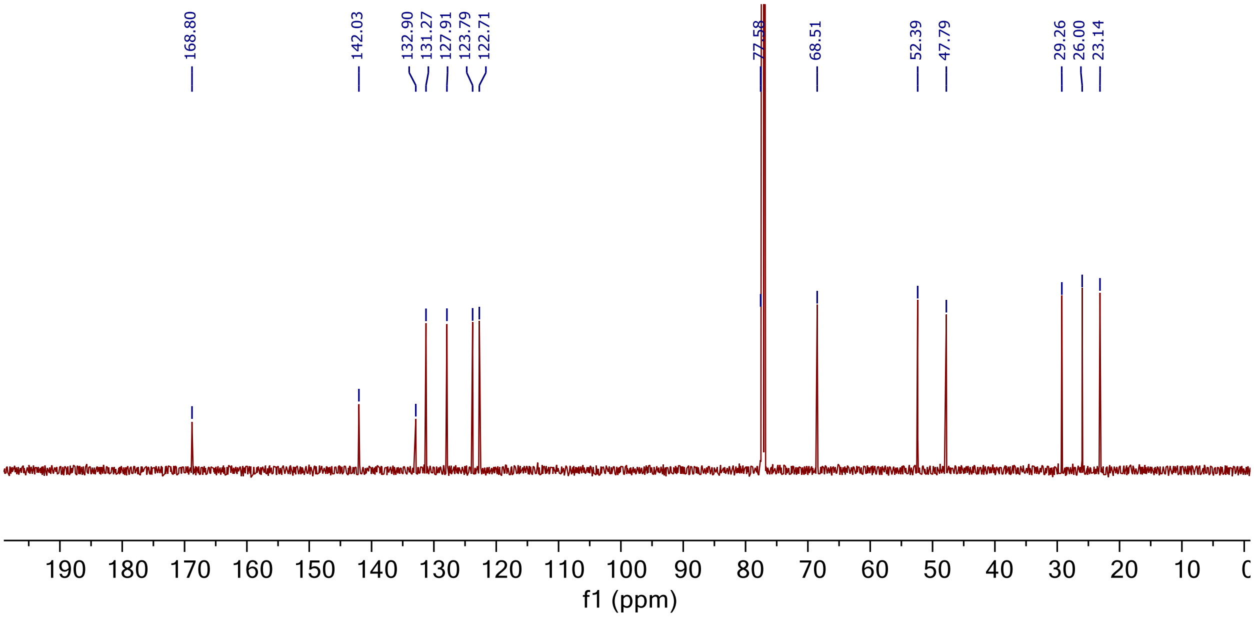

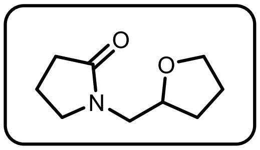

1-((tetrahydrofuran-2-yl)methyl)pyrrolidin-2-one (50b)

^{1}H NMR (600 MHz, $CDCl_3$)

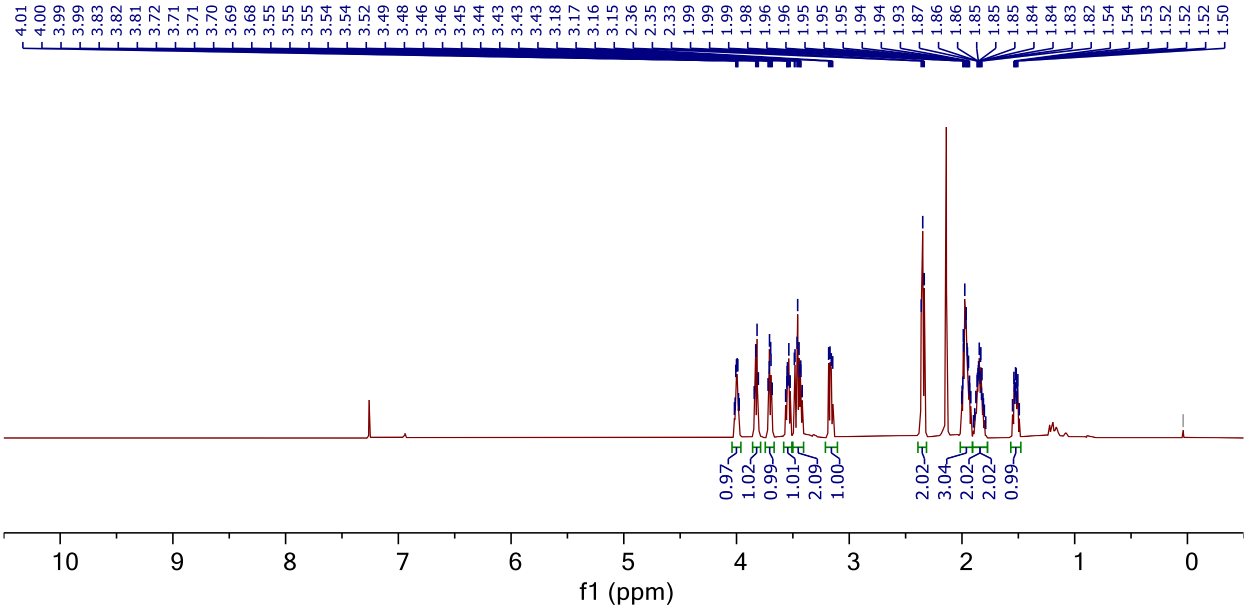

^{13}C NMR (151 MHz, $CDCl_3$)

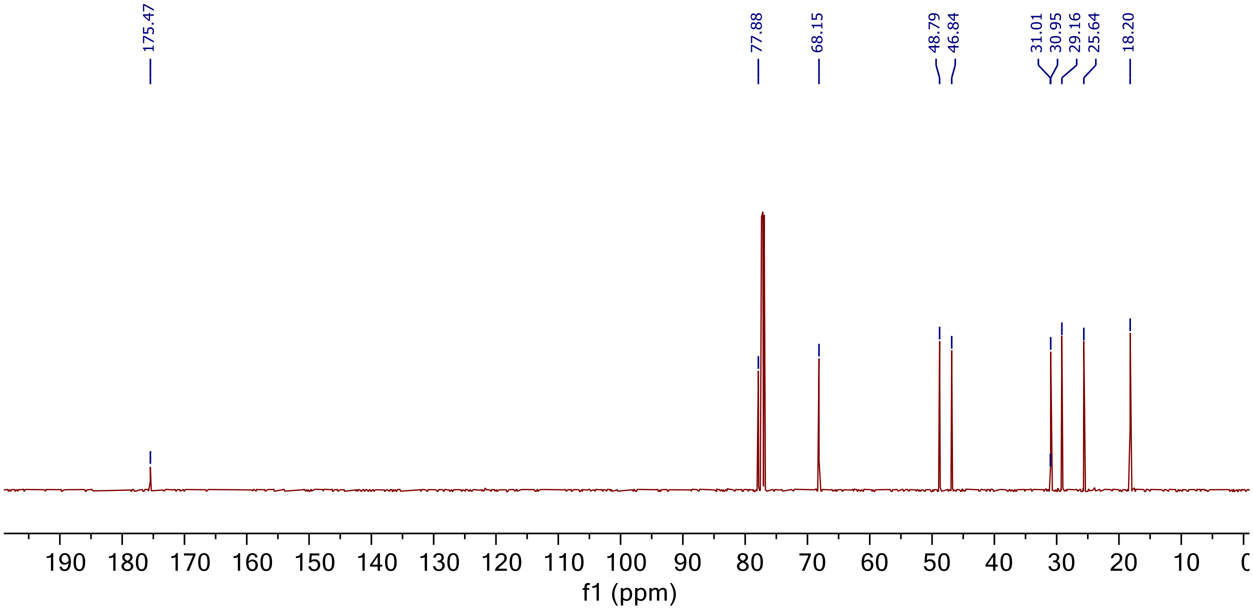

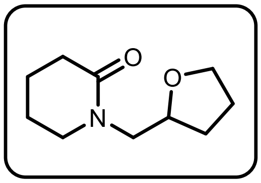

1-((tetrahydrofuran-2-yl)methyl)piperidin-2-one (51b)

^{1}H NMR (600 MHz, $CDCl_3$)

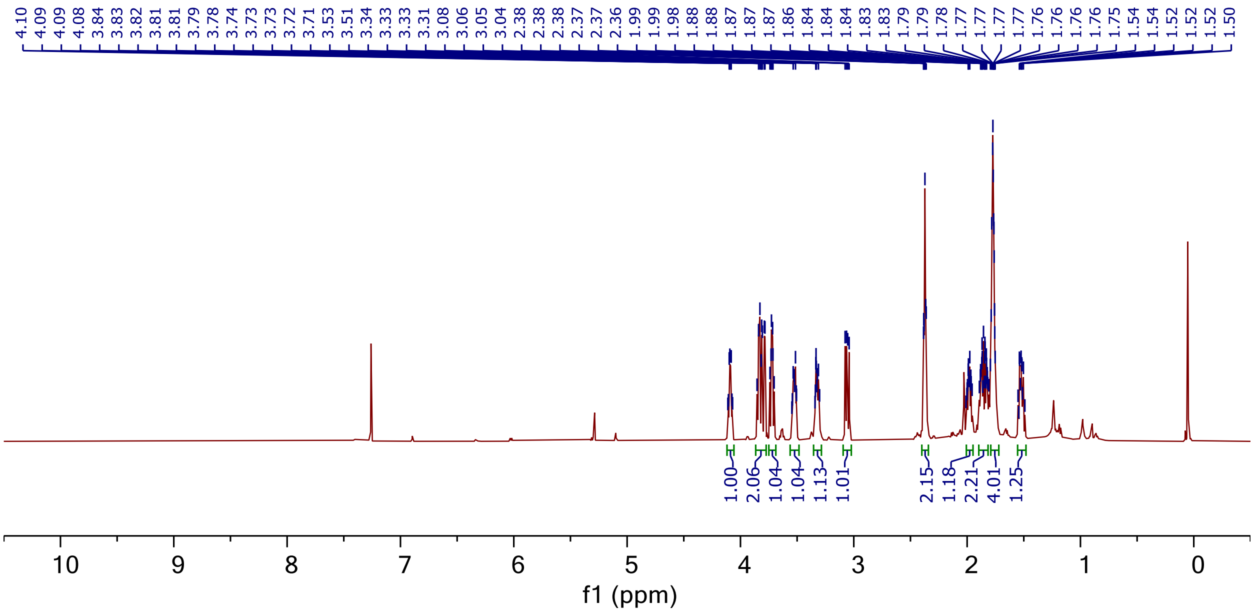

^{13}C NMR (151 MHz, $CDCl_3$)

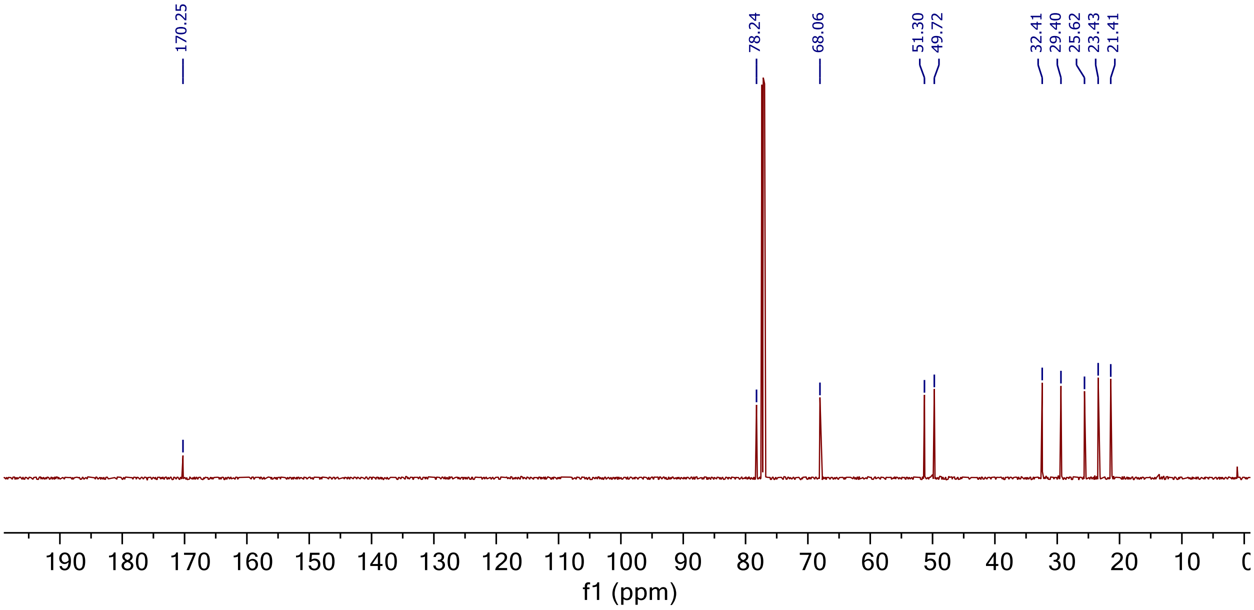

H. Catalytic Intermolecular Reaction Procedure

i) General Procedures

General Procedure H1: Intermolecular Hydroetherification A

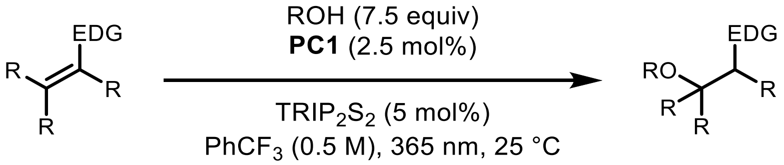

A flame-dried 1 dram vial equipped with a magnetic stir bar was transferred to a nitrogen-filled glovebox. The vial was charged with substrate (0.1 mmol, 1.0 equiv) and **PC1** (0.0025 mmol, 2.5 mol%). Following these additions, freeze-pump-thaw degassed anhydrous $PhCF_3$ (0.2 mL, 0.5 M) and freshly distilled freeze-pump-thaw degassed alcohol (0.75 mmol, 7.5 equiv) were added to the reaction vial, after which the vial was sealed with a threaded cap. The resulting solution was allowed to stir for 1 minute. The cap was then removed, and $TRIP_2S_2$ (0.005 mmol, 5 mol%) was quickly added to the vial as a solid in a single portion. The vial was sealed with a threaded cap, removed from the glovebox, and further reinforced with parafilm. The reaction vial was then transferred to a Hepatochem photoreactor attached to a recirculating chiller set to maintain a temperature of 25 °C. The reaction was set to stir under 365 nm light irradiation. At the designated time, the vial was removed from the photoreactor and the solvent was removed *in vacuo*. The resultant crude mixture was loaded directly onto silica gel for purification via flash column chromatography to access the intermolecular hydrotherification product.

General Procedure H2: Intermolecular Hydroetherification B

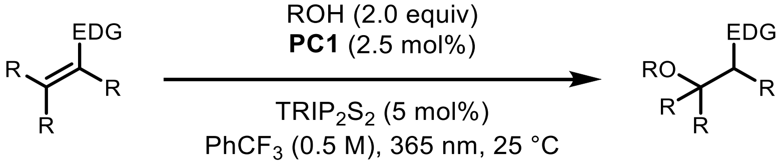

A flame-dried 1 dram vial equipped with a magnetic stir bar was transferred to a nitrogen-filled glovebox. The vial was charged with substrate (0.1 mmol, 1.0 equiv) and **PC1** (0.0025 mmol, 2.5 mol%). Following these additions, freeze-pump-thaw degassed anhydrous $PhCF_3$ (0.2 mL, 0.5 M) and freshly distilled freeze-pump-thaw degassed alcohol (0.2 mmol, 2.0-2.5 equiv) were added to the reaction vial, after which the vial was sealed with a threaded cap. The resulting solution was allowed to stir for 1 minute. The cap was then removed, and $TRIP_2S_2$ (0.005 mmol, 5 mol%) was quickly added to the vial as a solid in a single portion. The vial was sealed with a threaded cap, removed from the glovebox, and further reinforced with parafilm. The reaction vial was then transferred to a Hepatochem photoreactor attached to a recirculating chiller set to maintain a temperature of 25 °C. The reaction was set to stir under 365 nm light irradiation. At the designated time, the vial was removed from the photoreactor and the solvent was removed *in vacuo*. The resultant crude was loaded directly onto silica gel for purification via flash column chromatography to access the intermolecular hydrotherification product.

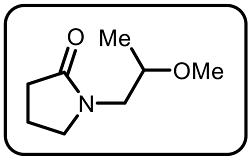

1-(2-methoxypropyl)pyrrolidin-2-one (1b). The reaction was set up using general procedure **H1** with 1-(prop-1-en-1-yl)pyrrolidin-2-one (12.5 mg, 0.1 mmol) and MeOH (30 µL, 0.75 mmol) for 16 hours. Following this, the crude product was purified using silica column chromatography (EtOAc/MeOH 100/0 to EtOAc/MeOH 90/10) to yield the indicated product in 99% yield (15.6 mg) as a colorless oil.

IR (Diamond-ATR, neat) ṽ (cm^{-1}): 2969, 2928, 1668, 1422, 1284.

^{1}H NMR (600 MHz, $CDCl_3$): δ 3.57 – 3.45 (m, 3H), 3.37 (dd, *J* = 14.2, 3.7 Hz, 1H), 3.32 (s, 3H), 3.23 (dd, *J* = 14.1, 7.1 Hz, 1H), 2.38 (at, *J* = 8.1 Hz, 2H), 2.00 (ap, *J* = 7.6 Hz, 2H), 1.12 (d, *J* = 6.3 Hz, 3H).

^{13}C NMR (151 MHz, $CDCl_3$): δ 175.3, 75.9, 56.2, 49.1, 47.7, 30.9, 18.2, 16.8.

HRMS (ESI): m/z: $[M+H]^+$ calc'd for $C_8H_{16}NO_2^+$: 158.1181. Found: 158.1178.

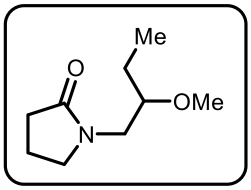

1-(2-methoxybutyl)pyrrolidin-2-one (2b). The reaction was set up using general procedure **H1** with 1-(but-1-en-1-yl)pyrrolidin-2-one (13.9 mg, 0.1 mmol) and MeOH (30 µL, 0.75 mmol) for 36 hours. Following this, the crude product was purified using silica column chromatography (EtOAc/MeOH 100/0 to EtOAc/MeOH 90/10) to yield the indicated product in 57% yield (9.7 mg) as a colorless oil.

IR (Diamond-ATR, neat) ṽ (cm^{-1}): 2944, 1653, 1112, 1015.

^{1}H NMR (600 MHz, $CDCl_3$): δ 3.50 (atd, *J* = 7.2, 1.7 Hz, 2H), 3.39 (dd, *J* = 13.5, 3.1 Hz, 1H), 3.35 (s, 3H), 3.36 – 3.24 (m, 2H), 2.37 (at, *J* = 8.1 Hz, 2H), 2.00 (ap, *J* = 7.8 Hz, 2H), 1.55 – 1.45 (m, 2H), 0.94 (at, *J* = 7.5 Hz, 3H).

^{13}C NMR (151 MHz, $CDCl_3$): δ 175.5, 81.3, 57.0, 49.2, 45.5, 31.0, 24.6, 18.4, 9.5.

HRMS (ESI): m/z: $[M+H]^+$ calc'd for $C_9H_{18}NO_2{}^+$: 172.1337. Found:172.1336.

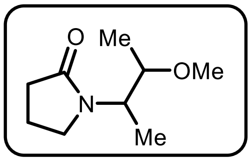

1-(3-methoxybutan-2-yl)pyrrolidin-2-one (3b). The reaction was set up using general procedure **H1** with 1-(but-2-en-2-yl)pyrrolidin-2-one (13.9 mg, 0.1 mmol) and MeOH (30 µL, 0.75 mmol) for 16 hours. Following this, the crude product was purified using silica column chromatography (EtOAc/MeOH 100/0 to EtOAc/MeOH 90/10) to yield a inseparable mixture of stereoisomers in 88% yield (15.1 mg, *dr* = 1:1) as a colorless oil.

IR (Diamond-ATR, neat) ṽ (cm^{-1}): 2959, 2925, 2358, 2342, 1683, 1260.

^{1}H NMR (600 MHz, $CDCl_3$): δ 4.16 – 4.02 (m, 1H), 3.45 – 3.31 (m, 3H), 3.30 – 3.27 (m, 3H), 2.41 – 2.32 (m, 2H), 2.01 – 1.91 (m, 2H), 1.16 – 1.13 (m, 3H), 1.11 – 1.07 (m, 3H).

^{13}C NMR (151 MHz, $CDCl_3$): δ 175.2, 78.6, 56.5, 50.7, 44.2, 31.3, 18.4, 15.9, 14.5.

HRMS (ESI): m/z: $[M+H]^+$ calc'd for $C_9H_{18}NO_2^+$: 172.1338. Found: 172.1332.

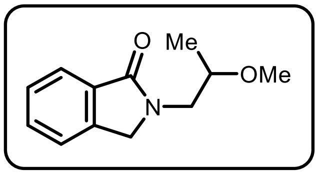

2-(2-methoxypropyl)isoindolin-1-one (4b). The reaction was set up using general procedure **H1** with 2-(prop-1-en-1-yl)isoindolin-1-one (17.3 mg, 0.1 mmol) and MeOH (30 µL, 0.75 mmol) for 16 hours. Following this, the crude product was purified using silica column chromatography (EtOAc/MeOH 100/0 to EtOAc/MeOH 90/10) to yield the indicated product in 98% yield (20.2 mg) as a colorless oil.

IR (Diamond-ATR, neat) ṽ (cm^{-1}): 3485, 2974, 2927, 2827, 1669, 1619, 1455.

^{1}H NMR (600 MHz, $CDCl_3$): δ 7.85 (d, *J* = 7.5 Hz, 1H), 7.53 (atd, *J* = 7.4, 1.2 Hz, 1H), 7.45 (dd, *J* = 11.5, 7.5 Hz, 2H), 4.59 (d, *J* = 17.3 Hz, 1H), 4.51 (d, *J* = 17.3 Hz, 1H), 3.77 (dd, *J* = 14.3, 3.2

Hz, 1H), 3.67 (apd, J = 6.1, 3.2 Hz, 1H), 3.52 (dd, J = 14.2, 7.0 Hz, 1H), 3.34 (s, 3H), 1.19 (d, J = 6.2 Hz, 3H).
^{13}C NMR (151 MHz, $CDCl_3$): δ 168.8, 141.8, 132.7, 131.2, 127.8, 123.7, 122.6, 76.6, 56.2, 52.1, 47.6, 16.7.
HRMS (ESI): m/z: $[M+H]^+$ calc'd for $C_{12}H_{16}NO_2^+$: 206.1181. Found: 206.1176.

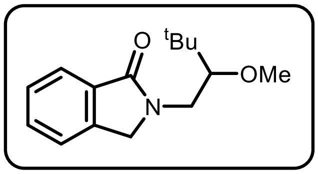

2-(2-methoxy-3,3-dimethylbutyl)isoindolin-1-one (5b). The reaction was set up using general procedure **H1** with (*E*)-2-(3,3-dimethylbut-1-en-1-yl)isoindolin-1-one (21.5 mg, 0.1 mmol) and MeOH (30 µL, 0.75 mmol) for 16 hours. Following this, the crude product was purified using silica gel column chromatography (hexanes/EtOAc 100/0 to hexanes/EtOAc 75/25) to yield the indicated product in 89% yield (22.0 mg) as a white solid.
IR (Diamond-ATR, neat) ṽ (cm^{-1}): 2947, 2877, 1682, 1103.
^{1}H NMR (600 MHz, $CDCl_3$): δ 7.88 – 7.81 (m, 1H), 7.52 (atd, J = 7.4, 1.2 Hz, 1H), 7.48 – 7.40 (m, 2H), 4.63 (d, J = 17.3 Hz, 1H), 4.45 (d, J = 17.3 Hz, 1H), 4.03 (dd, J = 14.0, 2.6 Hz, 1H), 3.31 (s, 3H), 3.28 (dd, J = 14.0, 9.0 Hz, 1H), 3.20 (dd, J = 9.0, 2.6 Hz, 1H), 0.98 (s, 9H).
^{13}C NMR (151 MHz, $CDCl_3$): δ 168.8, 141.9, 132.9, 131.3, 128.0, 123.7, 122.7, 88.7, 61.1, 52.4, 44.3, 35.5, 26.2.
HRMS (ESI): m/z: $[M+H]^+$ calc'd for $C_{15}H_{22}NO_2^+$: 248.1651. Found: 248.1652.

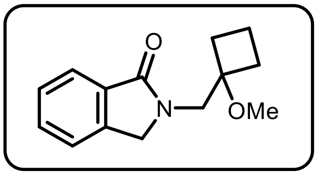

2-((1-methoxycyclobutyl)methyl)isoindolin-1-one (6b). The reaction was set up using general procedure **H1** with 2-(cyclobutylidenemethyl)isoindolin-1-one (19.9 mg, 0.1 mmol) and MeOH (30 µL, 0.75 mmol) for 16 hours. Following this, the crude product was purified using silica gel column chromatography (hexanes/EtOAc 75/25 to hexanes/EtOAc 67/33) to yield the indicated product in 65% yield (15.1 mg) as a colorless liquid.
IR (Diamond-ATR, neat) ṽ (cm^{-1}): 2932, 1718, 1684, 1470, 1411, 1324.
^{1}H NMR (600 MHz, $CDCl_3$): δ 7.85 (d, J = 7.5 Hz, 1H), 7.52 (atd, J = 7.4, 1.2 Hz, 1H), 7.47 – 7.39 (m, 2H), 4.53 (s, 2H), 3.84 (s, 2H), 3.27 (s, 3H), 2.20 – 2.12 (m, 2H), 2.00 – 1.92 (m, 2H), 1.82 – 1.75 (m, 2H).
^{13}C NMR (151 MHz, $CDCl_3$): δ 169.4, 142.1, 132.5, 131.3, 127.9, 123.8, 122.7, 80.2, 51.6, 49.7, 45.4, 29.3, 12.3.
HRMS (ESI): m/z: $[M+H]^+$ calc'd for $C_{14}H_{18}NO_2^+$:232.1337. Found: 232.1330.

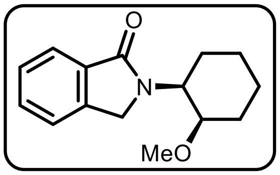

cis-2-(2-methoxycyclohexyl)isoindolin-1-one (7b). The reaction was set up using a modified version of general procedure **H1**. **PC1** (0.005 mmol, 5 mol%) $TRIP_2S_2$ (0.010 mmol, 10 mol%) was used with 1-(cyclohex-1-en-1-yl)indolin-2-one (21.3 mg, 0.1 mmol) and MeOH (30 µL, 0.75 mmol) for 36 hours. Following this, the crude product was purified using silica gel column chromatography (hexanes/EtOAc 75/25 to hexanes/EtOAc 67/33) to yield the indicated product in 63% yield (15.5 mg, *dr* = 6:1) as a white solid.

Major diastereomer:

IR (Diamond-ATR, neat) ṽ (cm^{-1}): 2928, 2857, 1676, 1407.
^{1}H NMR (600 MHz, $CDCl_3$): δ 7.86 (d, J = 7.6 Hz, 1H), 7.52 (atd, J = 7.4, 1.2 Hz, 1H), 7.44 (adt, J = 7.6, 3.5 Hz, 2H), 4.75 (d, J = 17.7 Hz, 1H), 4.42 (d, J = 17.7 Hz, 1H), 4.32 (adt, J = 12.9, 3.5

Hz, 1H), 3.67 (aq, *J* = 2.6 Hz, 1H), 3.24 (s, 3H), 2.10 – 2.01 (m, 2H), 1.88 – 1.82 (m, 1H), 1.59 (adt, *J* = 12.5, 3.8 Hz, 1H), 1.54 – 1.41 (m, 4H).
^{13}C NMR (151 MHz, $CDCl_3$): δ 168.3, 142.5, 133.1, 131.1, 127.8, 123.7, 122.7, 79.0, 56.1, 53.2, 49.1, 27.4, 25.5, 25.4, 19.1.
HRMS (ESI): m/z: $[M+H]^+$ calc'd for $C_{15}H_{20}NO_2^+$: 246.1494. Found: 246.1489.
The relative configuration of the diastereomer was assigned based on analysis of the ^{1}H NMR splitting patterns of diastereotopic protons, in comparison with the reported data for the opposite diastereomer.[27]

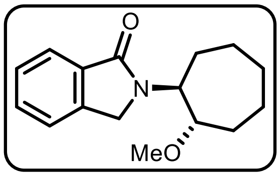

trans-2-(2-methoxycycloheptyl)isoindolin-1-one (8b). The reaction was set up using general procedure **H1** with 1-(cyclohept-1-en-1-yl)indolin-2-one (22.7 mg, 0.1 mmol) and MeOH (30 µL, 0.75 mmol) for 36 hours. Following this, the crude product was purified using silica gel column chromatography (hexanes/EtOAc 75/25 to hexanes/EtOAc 67/33) to yield the indicated product in 72% yield (18.7 mg, *dr* = 2:1) as an off-white solid.
Major diastereomer:
IR (Diamond-ATR, neat) ṽ (cm^{-1}): 2925, 2859, 1679, 1452, 1381.
^{1}H NMR (600 MHz, $CDCl_3$): δ 7.85 – 7.82 (m, 1H), 7.51 (atd, *J* = 7.3, 1.2 Hz, 1H), 7.46 – 7.41 (m, 2H), 4.43 (d, *J* = 16.7 Hz, 1H), 4.39 (d, *J* = 16.6 Hz, 1H), 4.13 (atd, *J* = 9.7, 3.0 Hz, 1H), 3.63 (ddd, *J* = 9.1, 7.4, 3.3 Hz, 1H), 3.23 (s, 3H), 1.93 –1.85 (m, 2H), 1.83 – 1.78 (m, 2H), 1.78 – 1.71 (m, 2H), 1.71 – 1.66 (m, 1H), 1.63 – 1.56 (m, 1H), 1.55 – 1.45 (m, 2H).
^{13}C NMR (151 MHz, $CDCl_3$): δ 168.2, 141.5, 133.7, 131.0, 128.0, 123.7, 122.7, 82.4, 58.8, 55.9, 48.4, 30.8, 29.4, 28.3, 25.5, 22.3.
HRMS (ESI): m/z: $[M+H]^+$ calc'd for $C_{16}H_{22}NO_2^+$: 260.1650. Found: 260.1646.
The relative configuration of the diastereomer was assigned based on analogy of the ^{1}H NMR data to previously reported compounds.[27]

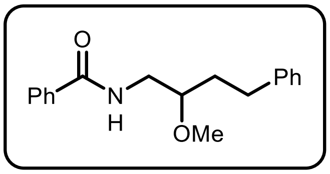

N-(2-methoxy-4-phenylbutyl)benzamide (9b). The reaction was set up using general procedure **H1** with *(E)-N*-(4-phenylbut-1-en-1-yl)benzamide (25.1 mg, 0.1 mmol) and MeOH (30 µL, 0.75 mmol) for 24 hours. Following this, the crude product was purified using silica gel column chromatography (hexanes/EtOAc 83/17 to hexanes/EtOAc 67/33) to yield the indicated product in 35% yield (9.8 mg) as a white solid.
IR (Diamond-ATR, neat) ṽ (cm^{-1}): 3309, 2922, 1632, 1602, 1578, 1539, 1301.
^{1}H NMR (600 MHz, $CDCl_3$): δ 7.78 (adt, *J* = 8.1, 1.1 Hz, 2H), 7.50 (atd, *J* = 7.2, 1.3 Hz, 1H), 7.44 (atd, *J* = 7.2, 1.2 Hz, 2H), 7.32 – 7.27 (m, 2H), 7.20 (dd, *J* = 8.0, 3.7 Hz, 3H), 6.45 (s, 1H), 3.75 (ddd, *J* = 14.0, 5.7, 3.7 Hz, 1H), 3.48 (adt, *J* = 13.9, 5.8 Hz, 1H), 3.44 – 3.42 (m, 1H), 3.41 (s, 3H), 2.73 (at, *J* = 8.0 Hz, 2H), 1.95 (addt, *J* = 14.1, 8.7, 6.6 Hz, 1H), 1.81 (atdd, *J* = 14.0, 7.7, 5.9 Hz, 1H).
^{13}C NMR (151 MHz, $CDCl_3$): δ 167.7, 141.8, 134.7, 131.6, 128.7, 128.6, 128.5, 127.1, 126.1, 79.0, 59.0, 42.0, 33.4, 31.5.
HRMS (ESI): m/z: $[M+H]^+$ calc'd for $C_{18}H_{22}NO_2^+$: 284.1650. Found: 284.1650.

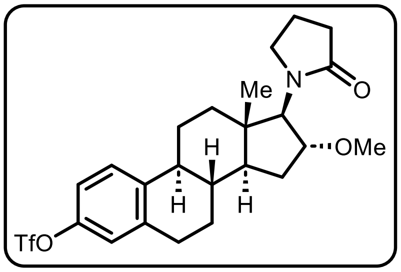

(8R,9S,13S,14S)-16-methoxy-13-methyl-17-(2-oxopyrrolidin-1-yl)- 7,8,9,11,12,13,14,15,16,17-decahydro-6H-cyclopenta[a] phenanthren-3-yl trifluoromethanesulfonate (10b). The reaction was set up using general procedure **H1** with (8*R*,9*S*,13*S*,14*S*)-13-methyl-17-(2-oxopyrrolidin-1-yl)-7,8,9,11,12,13,14,15-octahydro-6H-cyclopenta[a]phenanthren-3-yltrifluoromethanesulfonate (46.9 mg, 0.1 mmol) and MeOH (30 µL, 0.75 mmol) for 24 hours. Following this, the crude product was purified using silica gel column chromatography (hexanes/EtOAc 50/50 to hexanes/EtOAc 0/100) to yield the indicated product in 98% yield (48.9 mg, *dr* = 9:1) as a white solid.

IR (Diamond-ATR, neat) ṽ (cm^{-1}): 2926, 1678, 1417, 1209, 1140.

^{1}H NMR (600 MHz, $CDCl_3$): δ 7.31 (d, *J* = 8.7 Hz, 1H), 7.00 (dd, *J* = 8.7, 2.8 Hz, 1H), 6.95 (d, *J* = 2.7 Hz, 1H), 4.26 (at, *J* = 7.7 Hz, 1H), 3.90 (d, *J* = 7.1 Hz, 1H), 3.57 – 3.48 (m, 2H), 3.29 (s, 3H), 2.94 – 2.82 (m, 2H), 2.41 (asxt, *J* = 9.1 Hz, 2H), 2.34 – 2.22 (m, 2H), 2.04 (ap, *J* = 7.8 Hz, 2H), 1.93 – 1.88 (m, 1H), 1.87 – 1.81 (m, 2H), 1.69 (dddd, *J* = 19.1, 12.4, 9.3, 5.3 Hz, 3H), 1.49 – 1.34 (m, 3H), 0.77 (s, 3H).

^{13}C NMR (151 MHz, $CDCl_3$): δ 176.1, 147.6, 140.9, 139.4, 127.3, 121.2, 118.9 (q, $^{1}J_{C\text{-}F}$ = 321.6 Hz), 118.2, 80.1, 69.7, 56.8, 49.0, 47.8, 46.3, 44.0, 37.7, 37.4, 31.7, 30.7, 29.5, 26.9, 25.7, 19.0, 14.3.

^{19}F NMR (471 MHz, $CDCl_3$) δ -73.9.

HRMS (ESI): m/z: $[M+H]^+$ calc'd for $C_{24}H_{31}F_3NO_5S^+$: 502.18752. Found: 502.1860.

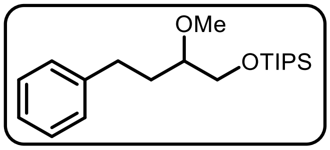

triisopropyl(2-methoxy-4-phenylbutoxy)silane (11b). The reaction was set up using general procedure **H1** with triisopropyl((4-phenylbut-1-en-1-yl)oxy)silane (30.4 mg, 0.1 mmol) and MeOH (30 µL, 0.75 mmol) for 24 hours. Following this, the crude product was purified using silica column chromatography (hexanes/Et_2O 100/0 to hexanes/Et_2O 90/10) to yield the indicated product in 51% yield (17.2 mg) as a colorless oil. The resulting product has been previously reported and matches the obtained spectroscopic data tabulated below.[11]

IR (Diamond-ATR, neat) ṽ (cm^{-1}): 2942, 2885, 1462, 1122.

^{1}H NMR (600 MHz, $CDCl_3$): δ 7.28 (at, *J* = 7.5 Hz, 2H), 7.23 – 7.15 (m, 3H), 3.76 (dd, *J* = 10.3, 5.4 Hz, 1H), 3.65 (dd, *J* = 10.3, 5.5 Hz, 1H), 3.45 (s, 3H), 3.28 – 3.21 (m, 1H), 2.79 (ddd, *J* = 15.3, 10.1, 5.3 Hz, 1H), 2.67 (ddd, *J* = 13.8, 9.9, 6.7 Hz, 1H), 1.92 – 1.87 (m, 1H), 1.82 – 1.75 (m, 1H), 1.16 – 1.03 (m, 21H).

^{13}C NMR (151 MHz, $CDCl_3$): δ 142.7, 128.7, 128.6, 125.9, 81.5, 65.5, 58.3, 33.5, 31.8, 18.2, 12.2.

HRMS (ESI): m/z: $[M+H]^+$ calc'd for $C_{20}H_{37}O_2Si^+$: 337.2563. Found: 337.2556.

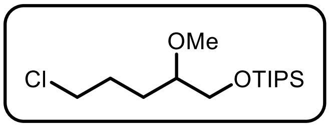

((5-chloro-2-methoxypentyl)oxy)triisopropylsilane (12b). The reaction was set up using general procedure **H1** with ((5-chloropent-1-en-1-yl)oxy)triisopropylsilane (27.6 mg, 0.1 mmol) and MeOH (30 µL, 0.75 mmol) for 24 hours. Following this, the crude product was purified using silica column chromatography (hexanes/Et_2O 100/0 to hexanes/Et_2O 95/5) to yield the indicated product in 65% yield (19.2 mg) as a colorless oil.

IR (Diamond-ATR, neat) ṽ (cm^{-1}): 2359, 1462, 1119, 1090, 881.

1H NMR (600 MHz, $CDCl_3$): δ 3.75 (dd, *J* = 10.2, 5.4 Hz, 1H), 3.62 (dd, *J* = 10.2, 5.4 Hz, 1H), 3.56 (aq, *J* = 6.6 Hz, 2H), 3.43 (s, 3H), 3.28 – 3.23 (m, 1H), 1.99 – 1.90 (m, 1H), 1.88 – 1.80 (m, 1H), 1.78 – 1.71 (m, 1H), 1.60 – 1.51 (m, 1H), 1.14 – 1.03 (m, 21H).
13C NMR (151 MHz, $CDCl_3$): δ 81.5, 65.4, 58.2, 45.5, 29.0, 28.7, 18.1, 12.1.
HRMS (ESI): m/z: $[M+H]^+$ calc'd for $C_{15}H_{34}ClO_2Si^+$: 309.2016. Found: 309.2015.

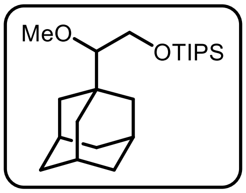

(2-adamantan-1-yl-2-methoxyethoxy)triisopropylsilane (13b). The reaction was set up using general procedure **H1** with (2-adamantan-1-yl)vinyloxy) triisopropylsilane (33.4 mg, 0.1 mmol) and MeOH (30 µL, 0.75 mmol) for 24 hours. Following this, the crude product was purified using silica column chromatography (hexanes/Et_2O 100/0 to hexanes/Et_2O 95/5) to yield the indicated product in 63% yield (23.0 mg) as a colorless oil.
IR (Diamond-ATR, neat) ṽ (cm^{-1}): 2900, 2864, 2847, 1466, 1124, 1104.
1H NMR (600 MHz, $CDCl_3$): δ 3.91 (dd, *J* = 10.6, 3.0 Hz, 1H), 3.67 (dd, *J* = 10.6, 7.5 Hz, 1H), 3.51 (s, 3H), 2.70 (dd, *J* = 7.5, 3.0 Hz, 1H), 1.95 (ap, *J* = 3.2 Hz, 3H), 1.72 – 1.60 (m, 12H), 1.10 – 1.06 (m, 21H).
13C NMR (151 MHz, $CDCl_3$): δ 91.2, 63.8, 61.1, 38.6, 37.3, 28.4, 18.1, 18.1, 11.9.
HRMS (ESI): m/z: $[M+H]^+$ calc'd for $C_{22}H_{43}O_2Si^+$: 367.3032. Found: 367.3030.

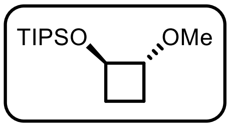

trans-triisopropyl(2-methoxycyclobutoxy)silane (14b). The reaction was set up using general procedure **H1** with (cyclobut-1-en-1-yloxy)triisopropylsilane (22.6 mg, 0.1 mmol) and MeOH (30 µL, 0.75 mmol) for 16 hours. Following this, the crude product was purified using silica column chromatography (hexanes/Et_2O 100/0 to hexanes/Et_2O 95/5) to yield the indicated product in 96% yield (24.9 mg, *dr* = 2.5:1) as a colorless oil. The resulting product has been previously reported and matches the obtained spectroscopic data tabulated below.[11]
IR (Diamond-ATR, neat) ṽ (cm^{-1}): 2942, 2891, 2865, 1463, 1383, 1175.
Major Diastereomer:
1H NMR (600 MHz, $CDCl_3$): δ 4.06 (aq, *J* = 7.5 Hz, 1H), 3.60 (aq, *J* = 7.6 Hz, 1H), 3.33 (s, 3H), 2.01 – 1.93 (m, 2H), 1.39 – 1.31 (m, 1H), 1.25 – 1.19 (m, 1H), 1.10 – 1.02 (m, 21H).
13C NMR (151 MHz, $CDCl_3$): δ 83.2, 73.4, 56.3, 23.4, 19.5, 17.9, 12.0.
Minor Diastereomer:
1H NMR (600 MHz, $CDCl_3$): δ 4.34 (aq, *J* = 6.3 Hz, 1H), 3.91– 3.87 (m, 1H), 3.42 (s, 3H), 2.15 – 2.09 (m, 2H), 1.83 – 1.75 (m, 2H), 1.10 – 1.02 (m, 21H).
13C NMR (151 MHz, $CDCl_3$): δ 80.5, 69.4, 57.3, 30.4, 22.3, 17.9, 12.1.
HRMS (ESI): m/z: $[M+H]^+$ calc'd for $C_{14}H_{31}O_2Si^+$: 259.2094. Found: 259.2089.

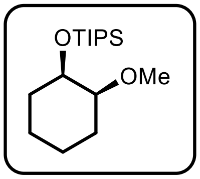

cis-triisopropyl-2-methoxycyclohexyl)oxy)silane (15b) The reaction was set up using general procedure **H1** with (cyclohex-1-en-1-yloxy)triisopropylsilane (25.4 mg, 0.1 mmol) and MeOH (30 µL, 0.75 mmol) for 16 hours. Following this, the crude product was purified using silica column chromatography (hexanes/Et_2O 100/0 to hexanes/Et_2O 95/5) to yield the indicated product in 38% yield (11.0 mg, *dr* = 3:1) as a colorless oil. The resulting product has been previously reported and matches the obtained spectroscopic data tabulated below.[11]
IR (Diamond-ATR, neat) ṽ (cm^{-1}): 2943, 2866, 1463, 1281, 1224, 994, 839.
Major diastereomer:

^{1}H NMR (600 MHz, $CDCl_3$): δ 3.95 (ad, *J* = 8.1 Hz, 1H), 3.39 (s, 3H), 3.23 (ad, *J* = 7.2 Hz, 1H), 1.87 – 1.76 (m, 2H), 1.72 – 1.62 (m, 2H), 1.50 – 1.39 (m, 2H), 1.30 – 1.22 (m, 2H), 1.03 – 0.97 (m, 21H).
^{13}C NMR (151 MHz, $CDCl_3$): δ 81.4, 71.1, 57.1, 31.6, 27.3, 22.3, 22.2, 18.3, 12.7.
Minor diastereomer:
^{1}H NMR (600 MHz, $CDCl_3$): δ 3.74 (aq, *J* = 6.4 Hz, 1H), 3.37 (s, 3H), 3.06 (adt, *J* = 3.9, 3.8 Hz, 1H), 1.87 – 1.76 (m, 2H), 1.72 – 1.62 (m, 2H), 1.50 – 1.39 (m, 2H), 1.30 – 1.22 (m, 2H), 1.03 – 0.97 (m, 21H).
^{13}C NMR (151 MHz, $CDCl_3$): δ 82.7, 72.0, 57.1, 32.3, 27.4, 22.4, 22.2, 18.3, 12.6.
HRMS (ESI): m/z: $[M+H]^+$ calc'd for $C_{16}H_{35}O_2Si^+$: 287.2406. Found: 287.2404.

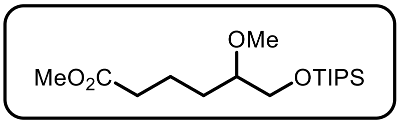

methyl 5-methoxy-6-((triisopropylsilyl)oxy)hexanoate (16b). The reaction was set up using general procedure **H1** with methyl 6-((triisopropylsilyl)oxy)hex-5-enoate (30.0 mg, 0.1 mmol) and MeOH (30 µL, 0.75 mmol) for 24 hours. Following this, the crude product was purified using silica column (hexanes/Et_2O 100/0 to hexanes/Et_2O 90/10) to yield the indicated product in 52% yield (17.3 mg) as a colorless oil.
IR (Diamond-ATR, neat) ṽ (cm^{-1}): 2941, 2865, 1740, 1462, 1090, 881, 680.
^{1}H NMR (600 MHz, $CDCl_3$): δ 3.73 (dd, *J* = 10.3, 5.5 Hz, 1H), 3.66 (s, 3H), 3.61 (dd, *J* = 10.3, 5.5 Hz, 1H), 3.43 (s, 3H), 3.25 – 3.20 (m, 1H), 2.33 (at, *J* = 7.6 Hz, 2H), 1.82 – 1.74 (m, 1H), 1.72 – 1.63 (m, 1H), 1.63 – 1.56 (m, 1H), 1.49 – 1.41 (m, 1H), 1.11 – 1.02 (m, 21H).
^{13}C NMR (151 MHz, $CDCl_3$): δ 174.1, 81.7, 65.3, 58.1, 51.5, 34.2, 31.0, 21.0, 18.0, 11.9.
HRMS (ESI): m/z: $[M+H]^+$ calc'd for $C_{17}H_{37}O_4Si^+$: 333.2461. Found: 333.2452.

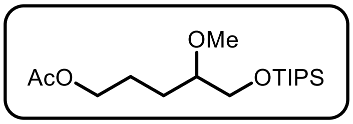

4-methoxy-5-((triisopropylsilyl)oxy)pentyl acetate (17b). The reaction was set up using general procedure **H1** with 5-((triisopropylsilyl)oxy)pent-4-en-1-yl acetate (30.0 mg, 0.1 mmol) and MeOH (30 µL, 0.75 mmol) for 24 hours. Following this, the crude product was purified using silica column chromatography (hexanes/Et_2O 100/0 to hexanes/Et_2O 90/10) to yield the indicated product in 70% yield (23.2 mg) as a colorless oil.
IR (Diamond-ATR, neat) ṽ (cm^{-1}): 2943, 2867, 1742, 1239, 1100.
^{1}H NMR (600 MHz, $CDCl_3$): δ 4.08 (at, *J* = 6.5 Hz, 2H), 3.75 (dd, *J* = 10.3, 5.5 Hz, 1H), 3.61 (dd, *J* = 10.2, 5.6 Hz, 1H), 3.43 (s, 3H), 3.28 – 3.21 (m, 1H), 2.04 (s, 3H), 1.84 – 1.75 (m, 1H), 1.72 – 1.61 (m, 2H), 1.55 – 1.46 (m, 1H), 1.14 – 1.03 (m, 21H).
^{13}C NMR (151 MHz, $CDCl_3$): δ 171.4, 81.7, 65.3, 64.8, 58.2, 28.1, 24.7, 21.1, 18.1, 12.1.
HRMS (ESI): m/z: $[M+H]^+$ calc'd for $C_{17}H_{37}O_4Si^+$: 333.24613. Found: 333.2456.

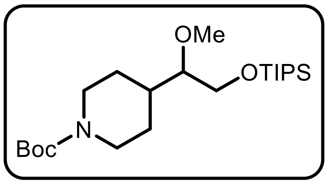

tert-butyl 4-(1-methoxy-2-((triisopropylsilyl)oxy) ethyl)piperidine-1-carboxylate (18b). The reaction was set up using general procedure **H1** with *tert*-butyl4-(2-((triisopropylsilyl)oxy)vinyl)piperidine-1-carboxylate (38.3 mg, 0.1 mmol) and MeOH (30 µL, 0.75 mmol) for 48 hours. Following this, the crude product was purified using silica column chromatography (PhMe/Et_2O/acetone 87.5/10/2.5) and isolated using silica preparatory thin layer chromatography to yield the indicated product as a white solid. The yield reported is an NMR yield (58%) using 1,1,2,2-tetrachloroethane (0.05 mmol) as an internal standard.
IR (Diamond-ATR, neat) ṽ (cm^{-1}): 2924, 2866, 1739, 1678, 1232, 1044.

^{1}H NMR (600 MHz, $CDCl_3$): δ 4.22 (d, *J* = 13.3 Hz, 1H), 4.13 (d, *J* = 11.3 Hz, 1H), 3.77 (d, *J* = 10.5 Hz, 1H), 3.72 (dd, *J* = 10.7, 5.3 Hz, 1H), 3.43 (s, 3H), 2.98 (d, *J* = 5.4 Hz, 1H), 2.79 (aq, *J* = 12.3 Hz, 1H), 2.67 (aq, *J* = 12.5 Hz, 1H), 1.79 (d, *J* = 13.4 Hz, 1H), 1.74 (adt, *J* = 16.7, 8.2 Hz, 1H), 1.63 (d, *J* = 13.1 Hz, 1H), 1.33 – 1.29 (m, 2H), 1.07 – 1.00 (m, 30H).
^{13}C NMR (151 MHz, $CDCl_3$): δ 154.1, 85.8, 63.2, 59.2, 45.3, 44.2, 38.2, 28.6, 27.8, 18.1, 12.3.
HRMS (ESI): m/z: $[M+K]^+$ calc'd for $C_{22}H_{46}NO_4SiK^+$: 454.2755. Found: 454.2840.

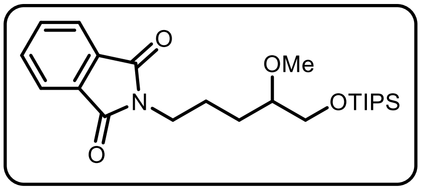

2-(4-methoxy-5-((triisopropylsilyl)oxy)pentyl)isoindoline-1,3-dione (19b).The reaction was set up using general procedure **H1** with 2-(5-((triisopropylsilyl)oxy)pent-4-en-1-yl)isoindoline-1,3-dione (38.8 mg, 0.1 mmol) and MeOH (30 µL, 0.75 mmol) for 48 hours. Following this, the crude product was purified using silica column chromatography ($PhMe/Et_2O$ 100/0 to $PhMe/Et_2O$ 90/10) to yield the indicated product in 38% yield (15.8 mg) as a white semisolid.
IR (Diamond-ATR, neat) ṽ (cm^{-1}): 2923, 2865, 1718, 1395, 1090.
^{1}H NMR (600 MHz, $CDCl_3$): δ 7.83 (dd, *J* = 5.4, 3.1 Hz, 2H), 7.70 (dd, *J* = 5.5, 3.0 Hz, 2H), 3.74 – 3.67 (m, 3H), 3.59 (dd, *J* = 10.3, 5.4 Hz, 1H), 3.42 (s, 3H), 3.27 – 3.22 (m, 1H), 1.89 – 1.80 (m, 1H), 1.78 – 1.69 (m, 1H), 1.64 – 1.57 (m, 1H), 1.52 – 1.44 (m, 1H), 1.10 – 0.99 (m, 21H).
^{13}C NMR (151 MHz, $CDCl_3$): δ 168.5, 134.0, 132.3, 123.3, 81.7, 65.4, 58.3, 38.2, 28.9, 24.7, 18.1, 12.0.
HRMS (ESI): m/z: $[M+H]^+$ calc'd for $C_{23}H_{38}NO_4Si^+$: 420.2570. Found: 420.2562.

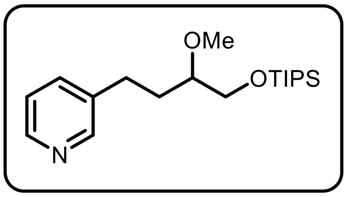

3-(3-methoxy-4-((triisopropylsilyl)oxy)butyl)pyridine (20b). The reaction was set up using general procedure **H1** with 3-(4-((triisopropylsilyl)oxy)but-3-en-1-yl)pyridine (30.6 mg, 0.1 mmol) and MeOH (30 µL, 0.75 mmol) for 48 hours. Following this, the crude product was purified using silica column chromatography (hexanes/EtOAc 100/0 to hexanes/EtOAc 75/25) to yield the indicated product in 25% yield (8.4 mg) as a white solid.
IR (Diamond-ATR, neat) ṽ (cm^{-1}): 2924, 2866, 2360, 2342, 1462, 1069.
^{1}H NMR (600 MHz, $CDCl_3$): δ 8.49 (br s, 2H), 7.13 (d, *J* = 4.1 Hz, 2H), 3.78 (dd, *J* = 10.3, 5.3 Hz, 1H), 3.63 (dd, *J* = 10.2, 5.8 Hz, 1H), 3.43 (s, 3H), 3.25 – 3.18 (m, 1H), 2.83 – 2.75 (m, 1H), 2.70 – 2.63 (m, 1H), 1.94 – 1.87 (m, 1H), 1.83 – 1.75 (m, 1H), 1.12-1.03 (m, 21H).
^{13}C NMR (151 MHz, $CDCl_3$): δ 151.6, 149.8, 124.1, 81.1, 65.1, 58.2, 32.3, 31.0, 29.9, 18.1, 12.6, 12.1.
HRMS (ESI): m/z: $[M+H]^+$ calc'd for $C_{19}H_{36}NO_2Si^+$: 338.2515. Found: 338.2504.

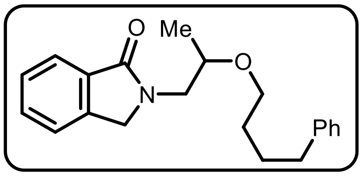

2-(2-(4-phenylbutoxy)propyl)isoindolin-1-one (21b). The reaction was set up using general procedure **H2** with 2-(prop-1-en-1-yl)isoindolin-1-one (17.3 mg, 0.1 mmol) and 4-phenylbutan-1-ol (30 mg, 0.2 mmol) for 24 hours. Following this, the crude product was purified using silica gel column chromatography (hexanes/EtOAc 80/20 to hexanes/EtOAc 25/75) to yield the indicated product in 88% yield (28.3 mg) as a colorless solid.
IR (Diamond-ATR, neat) ṽ (cm^{-1}): 2927, 2901, 2865, 1463, 1148, 1120.
^{1}H NMR (600 MHz, $CDCl_3$): δ 7.85 (d, *J* = 7.5 Hz, 1H), 7.52 (atd, *J* = 7.5, 1.2 Hz, 1H), 7.45 (at, *J* = 7.5 Hz, 1H), 7.41 (d, *J* = 7.5 Hz, 1H), 7.24 (d, *J* = 7.7 Hz, 2H), 7.19 – 7.15 (m, 1H), 7.12 – 7.09

(m, 2H), 4.58 (d, J = 17.4 Hz, 1H), 4.49 (d, J = 17.3 Hz, 1H), 3.79 – 3.72 (m, 2H), 3.55 (adt, J = 9.2, 6.3 Hz, 1H), 3.49 (aq, J = 7.1 Hz, 1H), 3.34 (adt, J = 9.1, 6.3 Hz, 1H), 2.60 – 2.55 (m, 2H), 1.66 – 1.61 (m, 2H), 1.60 – 1.54 (m, 2H), 1.18 (d, J = 6.1 Hz, 3H).
^{13}C NMR (151 MHz, $CDCl_3$): δ 168.7, 142.3, 141.8, 132.7, 131.1, 128.3, 128.2, 127.8, 125.7, 123.6, 122.5, 74.9, 68.5, 52.2, 47.9, 35.6, 29.7, 28.0, 17.3.
HRMS (ESI): m/z: $[M+H]^+$ calc'd for $C_{21}H_{26}NO_2^+$: 324.1964. Found: 324.1957.

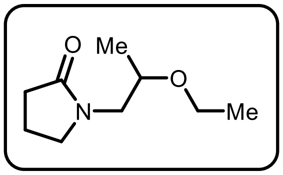

1-(2-ethoxypropyl)pyrrolidin-2-one (22b). The reaction was set up using general procedure **H1** with 1-(prop-1-en-1-yl)pyrrolidin-2-one (12.5 mg, 0.1 mmol) and EtOH (44 µL, 0.75 mmol) for 16 hours. Following this, the crude product was purified using silica column chromatography (EtOAc/MeOH 100/0 to EtOAc/MeOH 90/10) to yield the indicated product in 99% yield (33.9 mg) as a pale yellow oil.
IR (Diamond-ATR, neat) ṽ (cm^{-1}): 2972, 2973, 2872, 1667, 1464, 1443, 1424, 1093.
^{1}H NMR (600 MHz, $CDCl_3$): δ 3.66 – 3.60 (m, 1H), 3.59 – 3.53 (m, 2H), 3.52 – 3.45 (m, 1H), 3.42 – 3.36 (m, 2H), 3.16 (dd, J = 14.0, 7.3 Hz, 1H), 2.36 (t, J = 8.1 Hz, 2H), 2.03 – 1.96 (m, 2H), 1.16 (t, J = 7.0 Hz, 3H), 1.11 (d, J = 6.2 Hz, 3H).
^{13}C NMR (151 MHz, $CDCl_3$): δ 175.4, 74.3, 64.0, 49.3, 48.0, 30.9, 18.3, 17.6, 15.7.
HRMS (ESI): m/z: $[M+H]^+$ calc'd for $C_9H_{18}NO_2^+$: 172.1338. Found: 172.1333.

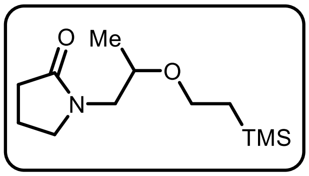

1-(2-(2-(trimethylsilyl)ethoxy)propyl)pyrrolidin-2-one (23b). The reaction was set up using general procedure **H2** with 1-(prop-1-en-1-yl)pyrrolidin-2-one (12.5 mg, 0.1 mmol) and 2-(trimethylsilyl)ethan-1-ol (28.7 µL, 0.2 mmol) for 24 hours. Following this, the crude product was purified using silica column chromatography (EtOAc/MeOH 100/0 to EtOAc/MeOH 90/10) to yield the indicated product in 99% yield (24.2 mg) as a colorless oil.
IR (Diamond-ATR, neat) ṽ (cm^{-1}): 2359, 1686, 1247, 1084, 858, 834.
^{1}H NMR (600 MHz, $CDCl_3$): δ 3.65 – 3.59 (m, 1H), 3.59 – 3.53 (m, 2H), 3.49 (adt, J = 10.0, 7.2 Hz, 1H), 3.44 – 3.34 (m, 2H), 3.18 (dd, J = 14.0, 7.1 Hz, 1H), 2.37 (t, J = 8.1 Hz, 2H), 1.99 (ap, J = 7.9 Hz, 2H), 1.11 (d, J = 6.3 Hz, 3H), 0.89 (t, J = 8.2 Hz, 2H), 0.01 (s, 9H).
^{13}C NMR (151 MHz, $CDCl_3$): δ 175.3, 73.9, 65.8, 49.3, 48.1, 30.9, 18.7, 18.3, 17.5, -1.4.
HRMS (ESI): m/z: $[M+H]^+$ calc'd for $C_{12}H_{26}NO_2Si^+$: 244.1733. Found: 244.1729.

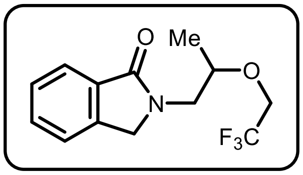

2-(2-(3,3,3-trifluoropropoxy)propyl)isoindolin-1-one (24b). The reaction was set up using general procedure **H1** with 2-(prop-1-en-1-yl)isoindolin-1-one (17.3 mg, 0.1 mmol) and 3,3,3-trifluoropropan-1-ol (55 µL, 0.75 mmol) for 16 hours. Following this, the crude product was purified using silica gel column chromatography (EtOAc/MeOH 100/0 to EtOAc/MeOH 90/10) to yield the indicated product in 63% yield (17.3 mg) as a colorless oil.
IR (Diamond-ATR, neat) ṽ (cm^{-1}): 2977, 2931, 2358, 1683, 1456, 1276.
^{1}H NMR (600 MHz, $CDCl_3$): δ 7.85 (d, J = 7.4 Hz, 1H), 7.54 (atd, J = 7.4, 1.2 Hz, 1H), 7.48 – 7.42 (m, 2H), 4.60 (d, J = 17.3 Hz, 1H), 4.49 (d, J = 17.2 Hz, 1H), 3.98 – 3.92 (m, 1H), 3.90 (adq, J = 12.0, 8.5 Hz, 1H), 3.83 (dd, J = 14.5, 3.1 Hz, 1H), 3.73 (adq, J = 12.0, 8.5 Hz, 1H), 3.53 (dd, J = 14.5, 7.3 Hz, 1H), 1.25 (d, J = 6.3 Hz, 3H).
^{13}C NMR (151 MHz, $CDCl_3$): δ 169.0, 141.8, 132.4, 131.4, 127.9, 123.9 (q, $^1J_{C-F}$ = 278.8 Hz) 123.7, 122.7, 77.5, 66.3 (q, $^2J_{C-F}$ = 34.2 Hz)., 52.2, 47.8, 16.9.
^{19}F NMR (471 MHz, $CDCl_3$) δ -75.6.

HRMS (ESI): m/z: $[M+H]^+$ calc'd for $C_{13}H_{15}F_3NO_2^+$: 274.1055. Found: 274.1000.

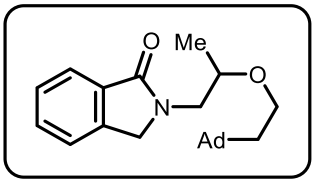

2-(2-(2-adamantan-1-yl)ethoxy)propyl)isoindolin-1-one (25b). The reaction was set up using the general procedure **H2** with 2-(prop-1-en-1-yl)isoindolin-1-one (17.3 mg, 0.1 mmol) and 2-(adamantan-1-yl)ethan-1-ol (36.1 mg, 0.2 mmol) for 24 hours. Following this, the crude product was purified using silica gel column chromatography (hexanes/EtOAc 80/20 to hexanes/EtOAc 25/75) to yield the indicated product in 92% yield (32.7 mg) as a colorless solid.

IR (Diamond-ATR, neat) ṽ (cm^{-1}): 2896, 2844, 1685, 1471, 1451.

^{1}H NMR (600 MHz, $CDCl_3$): δ 7.84 (d, *J* = 8.3 Hz, 1H), 7.51 (at, *J* = 6.9 Hz, 1H), 7.44 (at, *J* = 6.3 Hz, 2H), 4.62 – 4.47 (m, 2H), 3.77 – 3.71 (m, 2H), 3.61 – 3.55 (m, 1H), 3.49 (dd, *J* = 14.8, 7.8 Hz, 1H), 3.33 (td, *J* = 8.5, 6.9 Hz, 1H), 1.88 – 1.83 (m, 3H), 1.67 – 1.62 (m, 3H), 1.58 – 1.53 (m, 3H), 1.47 – 1,41 (m, 6H), 1.32 (ddd, *J* = 8.1, 6.4, 3.3 Hz, 2H), 1.17 (d, *J* = 6.2 Hz, 3H).

^{13}C NMR (151 MHz, $CDCl_3$): δ 168.7, 141.8, 132.7, 131.1, 127.7, 123.6, 122.5, 74.8, 64.4, 52.2, 47.9, 44.1, 42.6, 37.0, 31.6, 28.6, 17.2.

HRMS (ESI): m/z: $[M+H]^+$ calc'd for $C_{23}H_{32}NO_2^+$: 354.2433.1385. Found: 354.2424.

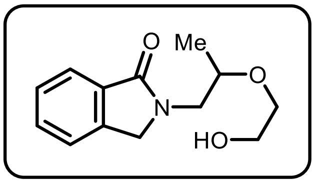

2-(2-(2-hydroxyethoxy)propyl)isoindolin-1-one (26b). The reaction was set up using general procedure **H1** with 2-(prop-1-en-1-yl)isoindolin-1-one (17.3 mg, 0.1 mmol) and ethylene glycol (41.9 µL, 0.75 mmol) for 16 hours. Following this, the crude product was purified using silica column chromatography (EtOAc/MeOH 100/0 to EtOAc/MeOH 90/10) to yield the indicated product in 70% yield (16.5 mg) as a white solid.

IR (Diamond-ATR, neat) ṽ (cm^{-1}): 3408, 2925, 1669, 1105, 1062.

^{1}H NMR (600 MHz, $CDCl_3$): δ 7.85 (d, *J* = 7.8 Hz, 1H), 7.53 (at, *J* = 7.4 Hz, 1H), 7.47 – 7.43 (m, 2H), 4.58 (d, *J* = 17.2 Hz, 1H), 4.51 (d, *J* = 17.2 Hz, 1H), 3.86 – 3.80 (m, 1H), 3.75 (dd, J = 14.4, 3.4 Hz, 1H), 3.72 – 3.69 (m, 2H), 3.68 – 3.64 (m, 1H), 3.59 (dd, *J* = 14.4, 7.0 Hz, 1H), 3.52 – 3.48 (m, 1H), 2.11 (at, *J* = 6.0 Hz, 1H), 1.23 (d, *J* = 6.3 Hz, 3H).

^{13}C NMR (151 MHz, $CDCl_3$): δ 169.0, 141.6, 132.6, 131.3, 127.9, 123.7, 122.6, 75.4, 69.9, 62.1, 52.1, 47.8, 17.5.

HRMS (ESI): m/z: $[M+H]^+$ calc'd for $C_{13}H_{18}NO_3^+$: 236.1287. Found: 236.1270.

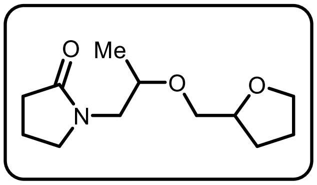

1-(2-((tetrahydrofuran-2-yl)methoxy)propyl)pyrrolidin-2-one (27b). The reaction was set up using general procedure **H1** with 1-(prop-1-en-1-yl)pyrrolidin-2-one (12.5 mg, 0.1 mmol) and (tetrahydrofuran-2-yl)methanol (73.0 µL, 0.75 mmol) for 16 hours. Following this, the crude product was purified using silica column chromatography (EtOAc/MeOH 100/0 to EtOAc/MeOH 90/10) to yield the indicated product in 84% yield (19.1 mg, *dr* = 1:1) as an inseparable mixture of diastereomers as a colorless oil.

IR (Diamond-ATR, neat) ṽ (cm^{-1}): 2970, 2866, 1667, 1285, 1071.

^{1}H NMR (600 MHz, $CDCl_3$): 3.99 (aq, *J* = 5.1 Hz, 1H), 3.85 (aq, *J* = 6.8 Hz, 1H), 3.79 – 3.73 (m, 1H), 3.72 – 3.64 (m, 1H), 3.63 – 3.56 (m, 1H), 3.55 – 3.46 (m, 2H), 3.44 – 3.32 (m, 2H), 3.21 (ddd, *J* = 28.0, 14.1, 7.4 Hz, 1H), 2.37 (at, *J* = 8.1 Hz, 2H), 1.99 (ap, *J* = 7.5 Hz, 2H), 1.97 – 1.91 (m, 1H), 1.87 (aq, *J* = 7.7 Hz, 2H), 1.61 – 1.54 (m, 1H), 1.14 (ddd, *J* = 5.3, 3.9, 2.0 Hz, 3H).

^{13}***C NMR*** (151 MHz, $CDCl_3$): δ 175.4, 78.0, 75.1, 71.4, 68.3, 49.2, 47.9, 30.9, 28.1, 25.6, 18.3, 17.3.
HRMS (ESI): m/z: $[M+H]^+$ calc'd for $C_{12}H_{22}NO_3^+$: 228.1600. Found: 228.1597.

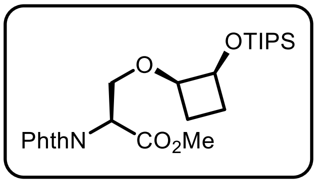

cis-methyl2-(1,3-dioxoisoindolin-2-yl)-3-(2-((triisopropylsilyl)oxy) cyclobutoxy)propanoate (28b). The reaction was set up using general procedure **H2** with (cyclobut-1-en-1-yloxy)triisopropylsilane (22.6 mg, 0.1 mmol) and *(S)*-methyl-2-(1,3-dioxoisoindolin-2-yl)-3-hydroxypropanoate (49.8 mg, 0.20 mmol) for 48 hours. Following this, the crude product was purified using silica gel column chromatography (hexanes/EtOAc 100/0 to hexanes/EtOAc 90/10) to yield the indicated product in 62% yield (29.7 mg, *dr* = 4:1) as a colorless sticky liquid.

Major diastereomer:

IR (Diamond-ATR, neat) ṽ (cm^{-1}): 2944, 2866, 2361, 2341, 1757, 1390, 1115.
^{1}***H NMR*** (600 MHz, $CDCl_3$): δ 7.86 (dd, *J* = 6.0, 2.8 Hz, 2H), 7.75 – 7.72 (m, 2H), 5.15 (adt, *J* = 10.6, 5.3 Hz, 1H), 4.24 – 4.18 (m, 1H), 4.13 – 4.08 (m, 1H), 3.97 (adtd, *J* = 8.5, 5.5, 3.5 Hz, 1H), 3.74 (s, 3H), 3.69 (addt, *J* = 16.6, 8.8, 4.5 Hz, 1H), 2.07 – 1.82 (m, 3H), 1.37 – 1.28 (m, 1H), 1.02 – 0.84 (m, 21H).
^{13}***C NMR*** (151 MHz, $CDCl_3$): δ 168.1, 167.6, 134.2, 132.1, 123.6, 81.9, 73.1, 64.7, 52.9, 51.9, 23.6, 20.0, 17.9, 12.1.
HRMS (ESI): m/z: $[M+H]^+$ calc'd for $C_{25}H_{38}NO_6Si^+$: 476.2468. Found: 476.2458.
The relative configuration of the diastereomer was assigned based on analysis of the ¹H NMR splitting patterns of diastereotopic protons, in comparison with the reported data.[11]

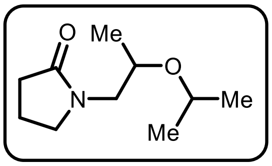

1-(2-isopropoxypropyl)pyrrolidin-2-one (29b). The reaction was set up using general procedure **H1** with 1-(prop-1-en-1-yl)pyrrolidin-2-one (12.5 mg, 0.1 mmol) and isopropanol (57 µL, 0.75 mmol) for 16 hours. Following this, the crude product was purified using silica column chromatography (EtOAc/MeOH 100/0 to EtOAc/MeOH 90/10) to yield the indicated product in 85% yield (15.8 mg) as a colorless oil.

IR (Diamond-ATR, neat) ṽ (cm^{-1}): 2970, 2925, 2360, 2341, 1682, 1270, 1123.
^{1}***H NMR*** (600 MHz, $CDCl_3$): δ 3.75 – 3.69 (m, 1H), 3.65 – 3.58 (m, 2H), 3.50 – 3.44 (m, 1H), 3.37 (dd, *J* = 14.0, 3.7 Hz, 1H), 3.08 (dd, *J* = 14.0, 7.6 Hz, 1H), 2.36 (at, *J* = 8.2 Hz, 2H), 1.99 (ap, *J* = 8.0 Hz, 2H), 1.13 (d, *J* = 6.1 Hz, 3H), 1.09 (d, *J* = 5.9 Hz, 6H).
^{13}***C NMR*** (151 MHz, $CDCl_3$): δ 175.2, 71.7, 69.6, 49.6, 48.6, 31.0, 23.2, 22.3, 18.5, 18.3.
HRMS (ESI): m/z: $[M+H]^+$ calc'd for $C_{10}H_{20}NO_2^+$: 186.1494. Found: 186.1485.

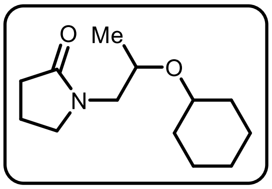

1-(2-isopropoxypropyl)pyrrolidin-2-one (30b). The reaction was set up using general procedure **H1** with 1-(prop-1-en-1-yl)pyrrolidin-2-one (12.5 mg, 0.1 mmol) and cyclohexanol (78 µL, 0.75 mmol) for 16 hours. Following this, the crude product was purified using silica column chromatography (EtOAc/MeOH 100/0 to EtOAc/MeOH 90/10) to yield the indicated product in 55% yield (12.3 mg) as a colorless oil.

IR (Diamond-ATR, neat) ṽ (cm^{-1}): 2927, 2854, 1673, 1115, 1074.
^{1}***H NMR*** (600 MHz, $CDCl_3$): δ 3.82 – 3.75 (m, 1H), 3.63 (adt, *J* = 9.9, 7.4 Hz, 1H), 3.49 (adt, *J* = 9.9, 7.4 Hz, 1H), 3.38 (dd, *J* = 13.9, 3.6 Hz, 1H), 3.27 (atq, *J* = 8.4, 3.6 Hz, 1H), 3.09 (dd, *J* = 13.9,

7.7 Hz, 1H), 2.37 (dd, *J* = 8.7, 7.5 Hz, 2H), 2.03 – 1.96 (m, 2H), 1.86 – 1.80 (m, 2H), 1.75 – 1.67 (m, 2H), 1.56 – 1.49 (m, 1H), 1.29 – 1.17 (m, 5H), 1.10 (d, *J* = 6.3 Hz, 3H).
^{13}C NMR (151 MHz, $CDCl_3$): δ 175.3, 75.8, 71.6, 49.7, 48.8, 33.6, 32.6, 31.0, 25.7, 24.3, 24.2, 18.6, 18.4.
HRMS (ESI): m/z: $[M+H]^+$ calc'd for $C_{13}H_{24}NO_2^+$: 226.1807. Found: 226.1811.

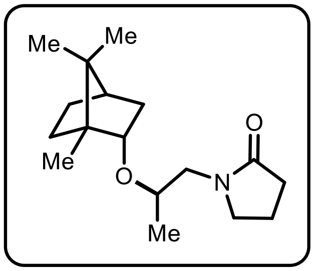

1-(2-(((1S,2S,4R)-1,7,7-trimethylbicyclo[2.2.1]heptan-2-yl)oxy)propyl) pyrrolidin -2-one (31b). The reaction was set up using general procedure **H1** with 1-(prop-1-en-1-yl)pyrrolidin-2-one (12.5 mg, 0.1 mmol) and (1*S*,2*S*,4*R*)-1,7,7-trimethylbicyclo[2.2.1]heptan-2-ol (116 mg, 0.75 mmol) for 16 hours. Following this, the crude product was purified using silica gel column chromatography (hexanes/EtOAc 80/20 to hexanes/EtOAc 50/50) to yield the indicated product in 61% yield (17.1 mg, *dr* = 1.1:1) as an inseparable mixture of diastereomers a colorless oil.
IR (Diamond-ATR, neat) ṽ (cm^{-1}): 2948, 2873, 1685, 1113, 1062.
Major diastereomer
^{1}H NMR (600 MHz, $CDCl_3$): δ 3.62 – 3.51 (m, 2H), 3.49 – 3.39 (m, 2H), 3.26 (dd, *J* = 13.9, 3.4 Hz, 1H), 3.15 (dd, *J* = 13.9, 7.5 Hz, 1H), 2.31 (aq, *J* = 7.8 Hz, 2H), 2.09 (addt, *J* = 13.2, 8.5, 4.0 Hz, 1H), 1.98 – 1.91 (m, 2H), 1.89 – 1.84 (m, 1H), 1.67 – 1.57 (m, 1H), 1.54 (at, *J* = 4.6 Hz, 1H), 1.21 – 1.04 (m, 2H), 1.02 (d, *J* = 6.3 Hz, 3H), 0.93 (dd, *J* = 13.0, 3.5 Hz, 1H), 0.80 – 0.73 (m, 9H).
^{13}C NMR (151 MHz, $CDCl_3$): δ 175.2, 84.1, 75.1, 49.7, 49.5, 48.5, 47.3, 45.1, 38.1, 31.0, 28.3, 26.7, 19.8, 19.0, 18.8, 18.3, 13.8.
Minor diastereomer
^{1}H NMR (600 MHz, $CDCl_3$): δ 3.62 – 3.51 (m, 2H), 3.49 – 3.39 (m, 2H), 3.33 (dd, *J* = 13.9, 3.2 Hz, 1H), 3.02 (dd, *J* = 13.9, 8.2 Hz, 1H), 2.31 (aq, *J* = 7.8 Hz, 2H), 2.02 (ddt, *J* = 13.2, 8.5, 4.0 Hz, 1H), 1.98 – 1.90 (m, 2H), 1.89 – 1.84 (m, 1H), 1.67 – 1.57 (m, 1H), 1.56 (at, *J* = 4.6 Hz, 1H), 1.17 – 1.03 (m, 3H), 0.99 (d, *J* = 6.1 Hz, 3H), 0.80 – 0.72 (m, 9H).
^{13}C NMR (151 MHz, $CDCl_3$): δ 175.2, 81.5, 72.4, 49.5, 49.4, 48.9, 47.7, 45.0, 36.6, 31.0, 28.4, 26.8, 19.8, 18.9, 18.4, 17.1, 13.6.
HRMS (ESI): m/z: $[M+H]^+$ calc'd for $C_{17}H_{30}NO_2^+$: 280.2277. Found: 280.2272.

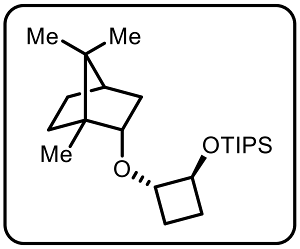

trans-triisopropyl(2-(((1S,2S,4R)-1,7,7-trimethylbicyclo[2.2.1]heptan-2-yl) oxy)cyclobutoxy)silane (32b). The reaction was set up using general procedure **H1** with (cyclobut-1-en-1-yloxy)triisopropylsilane (22.6 mg, 0.1 mmol) and (1*S*,2*S*,4*R*)-1,7,7-trimethylbicyclo[2.2.1]heptan-2-ol (116 mg, 0.75 mmol) for 16 hours. Following this, the crude product was purified using silica gel column chromatography (hexanes/Et_2O 100/0 to hexanes/Et_2O 90/10) to yield the indicated product in 45% yield (12.6 mg, *dr* = 3 :1) as a colorless oil.
IR (Diamond-ATR, neat) ṽ (cm^{-1}): 2958, 2866, 1259, 1091, 798.
Major diastereomer:
^{1}H NMR (600 MHz, $CDCl_3$): δ 4.05 (aq, *J* = 7.4 Hz, 1H), 3.71 (ddd, *J* = 9.5, 3.5, 1.9 Hz, 1H), 3.68 (aq, *J* = 7.4 Hz, 1H), 2.09 (ddt, *J* = 13.2, 8.5, 4.0 Hz, 1H), 2.00 – 1.94 (m, 2H), 1.93 – 1.87 (aq, *J* = 9.0 Hz, 1H), 1.69 – 1.62 (m, 1H), 1.59 (at, *J* = 4.7 Hz, 1H), 1.35 – 1.23 (m, 2H), 1.20 – 1.12 (m, 2H), 1.09 – 1.03 (m, 21H), 0.99 (dd, *J* = 13.0, 3.4 Hz, 1H), 0.85 (s, 3H), 0.86 (s, 3H), 0.82 (s, 3H).

^{13}C NMR (151 MHz, $CDCl_3$): δ 83.3, 81.4, 73.9, 45.1, 36.5, 28.3, 26.5, 24.0, 21.2, 19.8, 18.8, 17.9, 13.7, 12.0.

Minor diastereomer:

^{1}H NMR (600 MHz, $CDCl_3$): δ 4.33 (aq, *J* = 5.8 Hz, 1H), 3.97 (aq, *J* = 5.7 Hz, 1H), 3.64 – 3.60 (ddd, *J* = 9.5, 3.5, 1.9 Hz, 1H), 2.16 – 2.11 (m, 1H), 2.02 – 1.89 (m, 2H), 1.85 – 1.80 (m, 1H), 1.79 – 1.73 (m, 1H), 1.69 – 1.62 (m, 1H), 1.58 (at, *J* = 4.6 Hz, 1H), 1.25 – 1.19 (m, 2H), 1.15 – 1.05 (m, 2H), 1.06 – 1.05 (m, 21H), 0.86 (s, 3H), 0.82 (brs, 6H).

^{13}C NMR (151 MHz, $CDCl_3$): δ 82.4, 80.7, 73.5, 45.0, 36.3, 28.1, 26.6, 23.4, 20.4, 19.8, 18.9, 18.0, 17.9, 12.2.

HRMS (ESI): m/z: $[M+H]^+$ calc'd for $C_{23}H_{45}O_2Si^+$: 381.3189 Found: 381.3184.

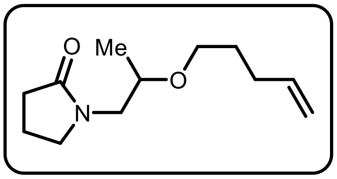

1-(2-(pent-4-en-1-yloxy)propyl)pyrrolidin-2-one (33b). The reaction was set up using general procedure **H1** with 1-(prop-1-en-1-yl)pyrrolidin-2-one (12.5 mg, 0.1 mmol) and pent-4-en-1-ol (155 uL, 0.75 mmol) for 16 hours. Following this, the crude product was purified using silica gel column chromatography (hexanes/EtOAc 100/0 to hexanes/EtOAc 50/50) to yield the indicated product in 99% yield (42.2 mg) as a pale yellow oil.

IR (Diamond-ATR, neat) ṽ (cm^{-1}): 2972, 2928, 1670, 1495, 1344.

^{1}H NMR (600 MHz, $CDCl_3$): δ 5.79 (addt, *J* = 16.9, 10.2, 6.6 Hz, 1H), 5.03 – 4.92 (m, 2H), 3.61 (att, *J* = 8.9, 4.5 Hz, 1H), 3.57 – 3.44 (m, 3H), 3.38 – 3.29 (m, 2H), 3.18 (dd, *J* = 14.0, 7.4 Hz, 1H), 2.36 (at, *J* = 8.1 Hz, 2H), 2.09 (aq, *J* = 6.4 Hz, 2H), 1.99 (ap, *J* = 7.9 Hz, 2H), 1.62 (ap, *J* = 6.6 Hz, 2H), 1.10 (d, *J* = 6.2 Hz, 3H).

^{13}C NMR (151 MHz, $CDCl_3$): δ 175.3, 138.2, 114.7, 74.4, 67.9, 49.3, 48.0, 30.9, 30.3, 29.3, 18.3, 17.4.

HRMS (ESI): m/z: $[M+H]^+$ calc'd for $C_{12}H_{22}NO_2^+$: 212.1650. Found: 212.1646.

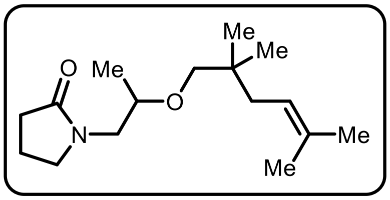

1-(2-((2,2,5-trimethylhex-4-en-1-yl)oxy)propyl)pyrrolidin-2-one (34b). The reaction was set up using general procedure **H2** with 1-(prop-1-en-1-yl)pyrrolidin-2-one (12.5 mg, 0.1 mmol) and 2,2,5-trimethylhex-4-en-1-ol (28.4 mg, 0.2 mmol) for 16 hours. Following this, the crude product was purified using silica column chromatography (hexanes/EtOAc 100/0 to hexanes/EtOAc 75/25) to yield the indicated product in 65% yield (17.3 mg) as a colorless oil.

IR (Diamond-ATR, neat) ṽ (cm^{-1}): 2967, 2924, 2871, 1687, 1424, 1284, 1085.

^{1}H NMR (600 MHz, $CDCl_3$): δ 5.16 – 5.11 (m, 1H), 3.62 – 3.53 (m, 2H), 3.53 – 3.47 (m, 1H), 3.35 (dd, *J* = 14.0, 3.6 Hz, 1H), 3.22 (dd, *J* = 14.0, 7.4 Hz, 1H), 3.18 (d, *J* = 8.6 Hz, 1H), 2.94 (d, *J* = 8.6 Hz, 1H), 2.37 (at, *J* = 8.3 Hz, 2H), 1.99 (ap, *J* = 7.3 Hz, 2H), 1.91 (at, *J* = 7.5 Hz, 2H), 1.71 (s, 3H), 1.59 (s, 3H), 1.09 (d, *J* = 6.2 Hz, 3H), 0.84 (s, 6H).

^{13}C NMR (151 MHz, $CDCl_3$): δ 175.2, 133.0, 120.8, 77.4, 74.7, 49.3, 48.2, 37.2, 35.5, 31.0, 26.0, 24.5, 24.4, 18.3, 17.8, 16.9.

HRMS (ESI): m/z: $[M+H]^+$ calc'd for $C_{16}H_{30}NO_2^+$: 268.2277. Found: 268.2269.

ii) NMR Spectra of Intermolecular Hydroetherification Products

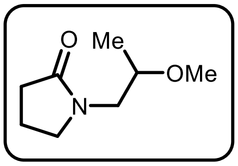

1-(2-methoxypropyl)pyrrolidin-2-one (1b)

^{1}H NMR (600 MHz, $CDCl_3$):

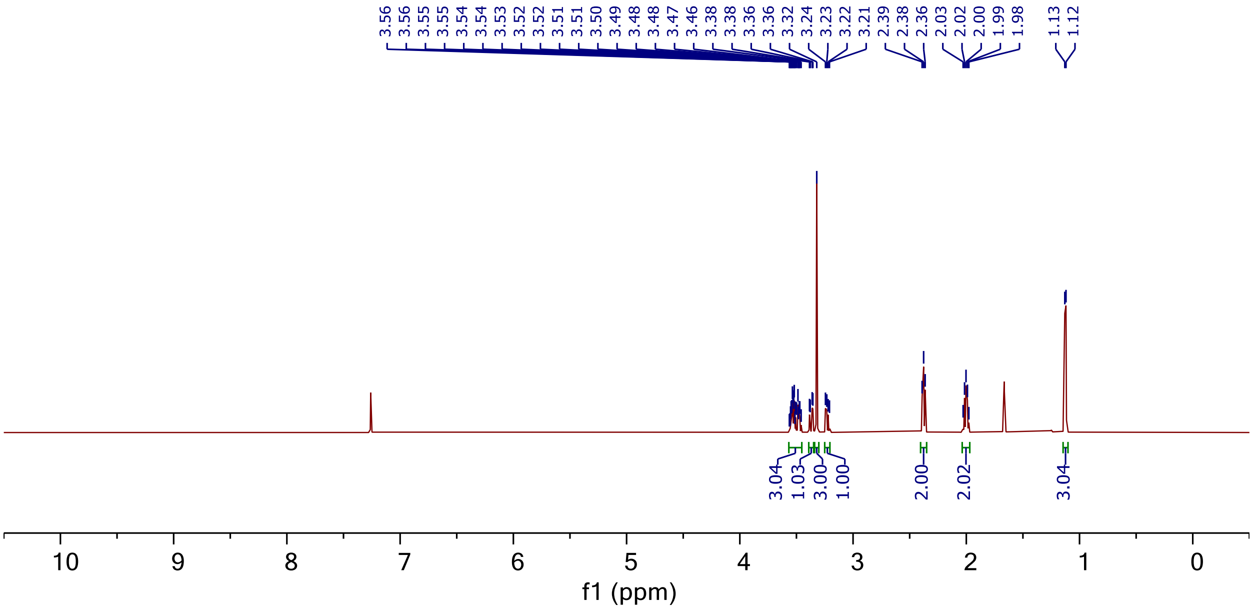

^{13}C NMR (151 MHz, $CDCl_3$):

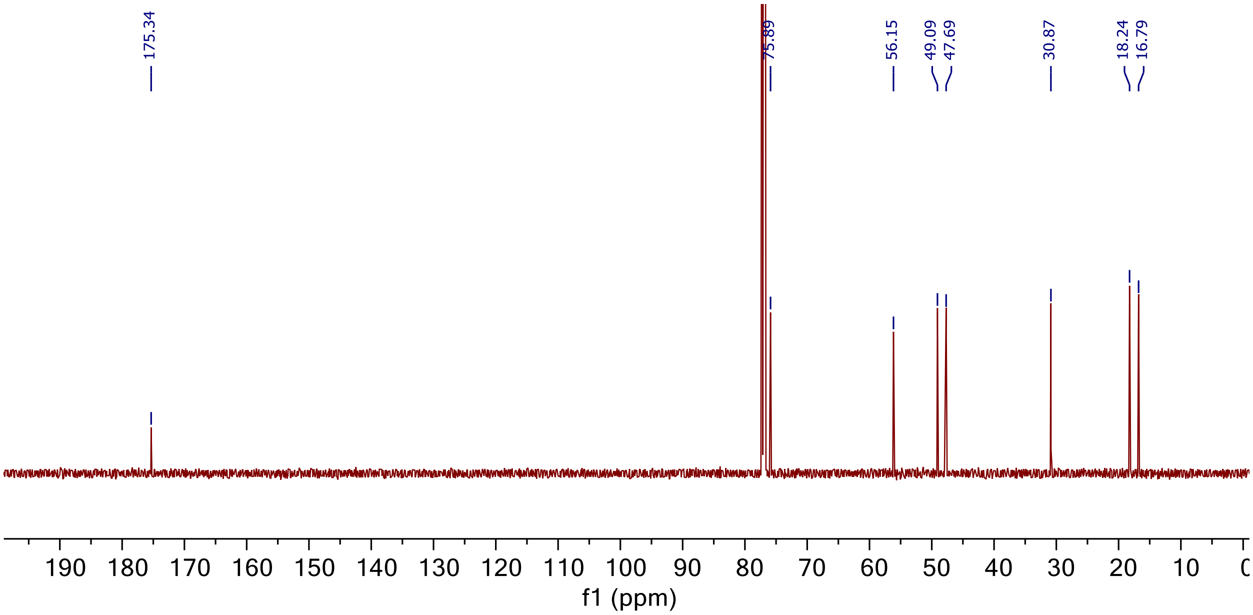

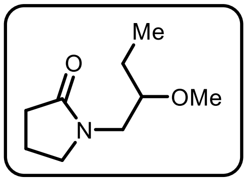

<u>1-(2-methoxybutyl)pyrrolidin-2-one (2b).</u>

^{1}H NMR (600 MHz, $CDCl_3$):

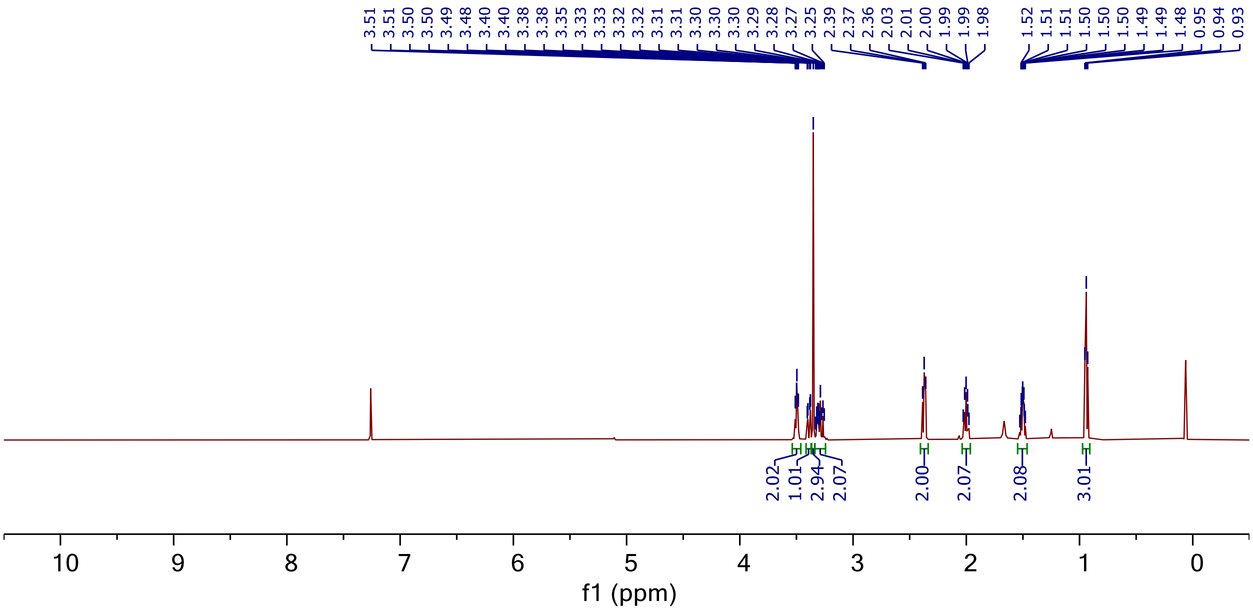

^{13}C NMR (151 MHz, $CDCl_3$):

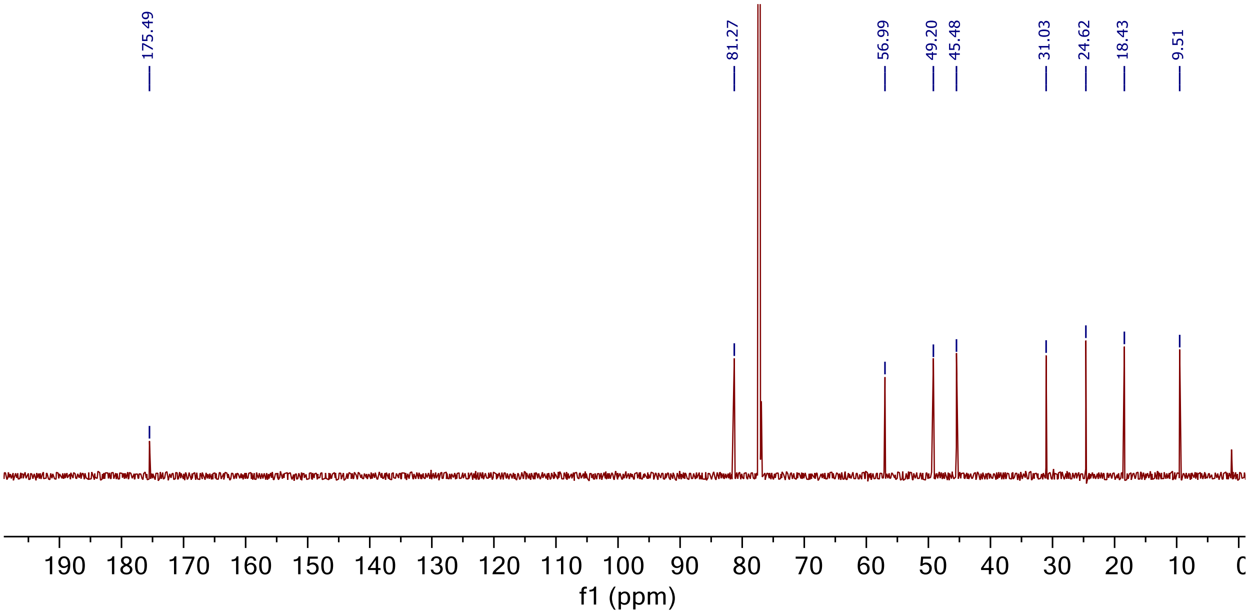

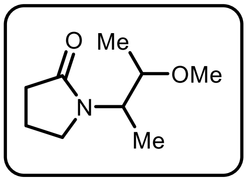

1-(3-methoxybutan-2-yl)pyrrolidin-2-one (3b).

^{1}H NMR (600 MHz, $CDCl_3$):

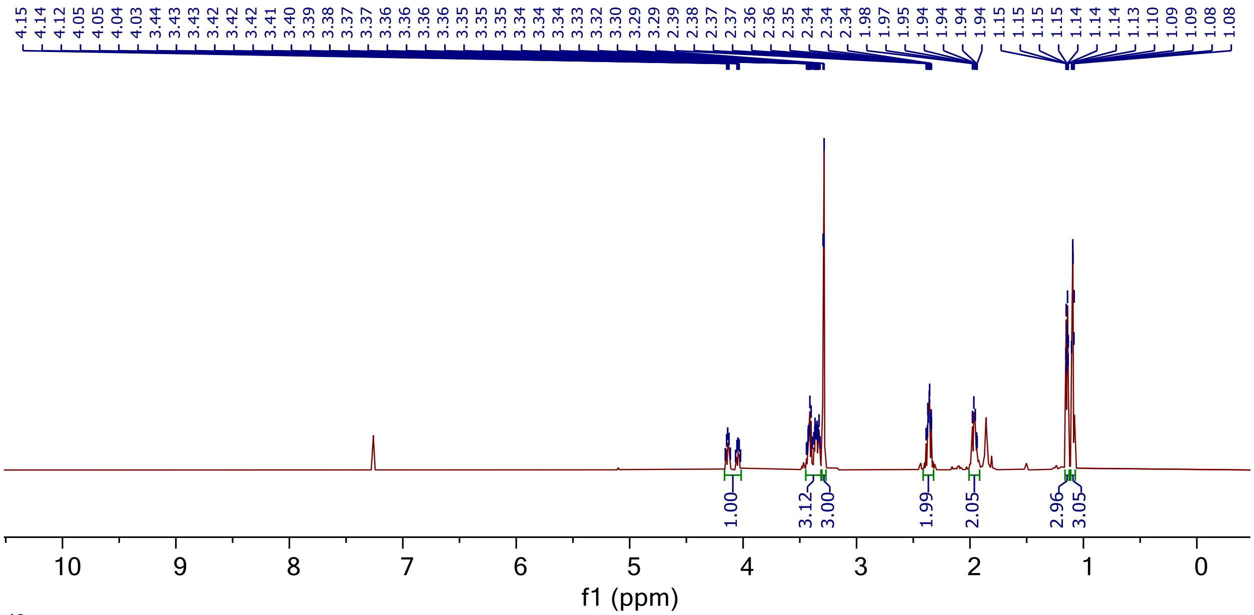

^{13}C NMR (151 MHz, $CDCl_3$):

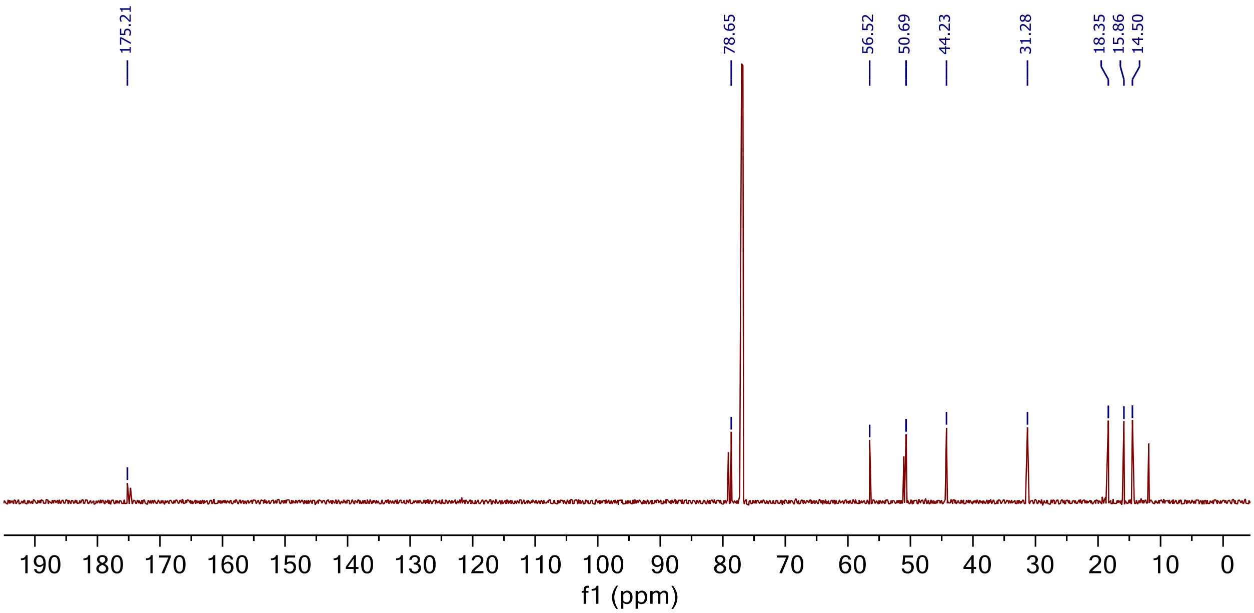

2-(2-methoxypropyl)isoindolin-1-one (4b).

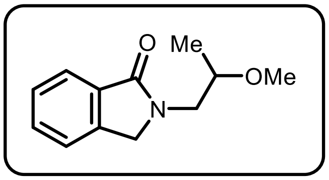

^{1}H NMR (600 MHz, $CDCl_3$):

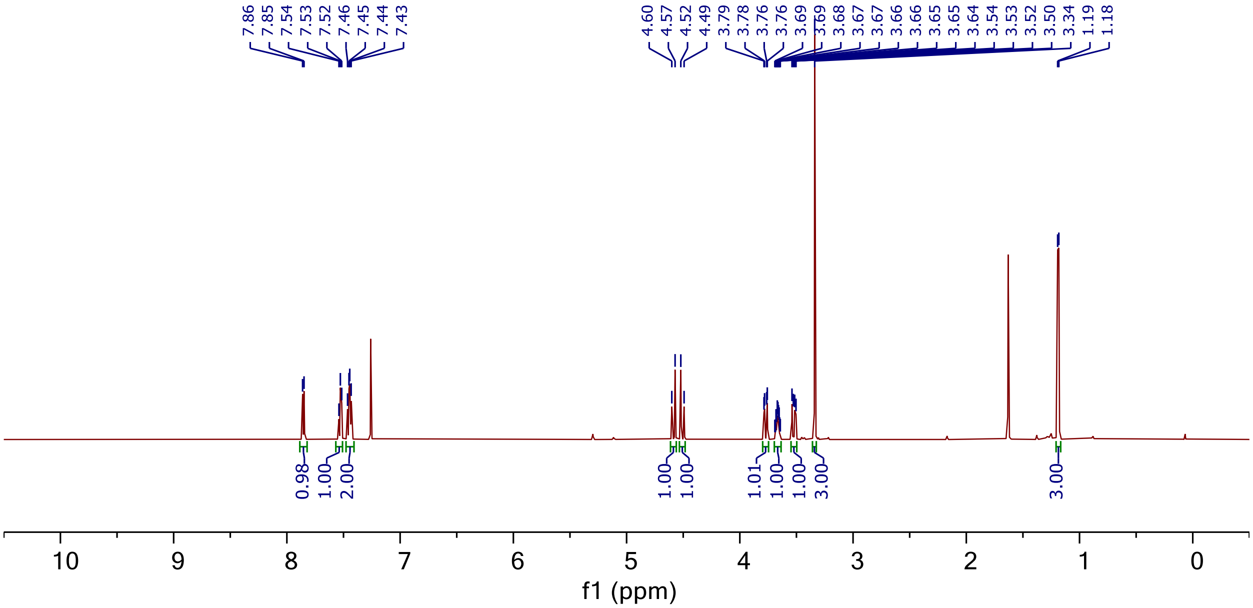

^{13}C NMR (151 MHz, $CDCl_3$):

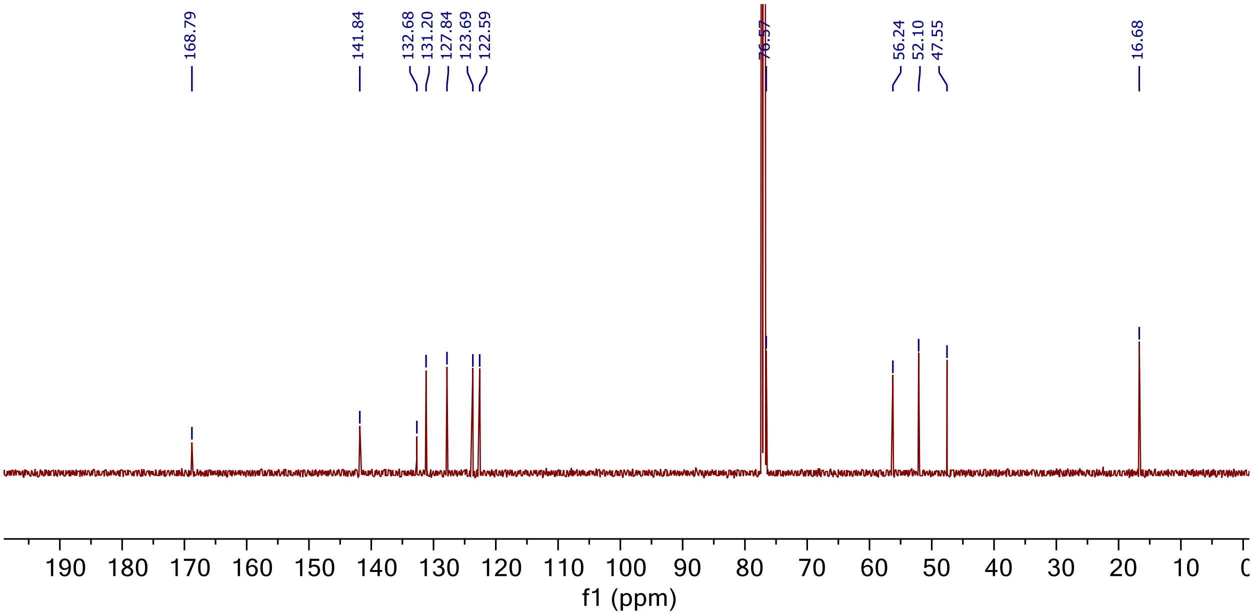

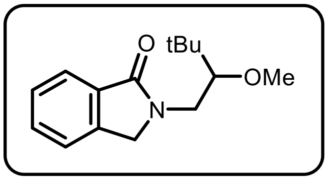

2-(2-methoxy-3,3-dimethylbutyl)isoindolin-1-one (5b).

^{1}H NMR (600 MHz, $CDCl_3$):

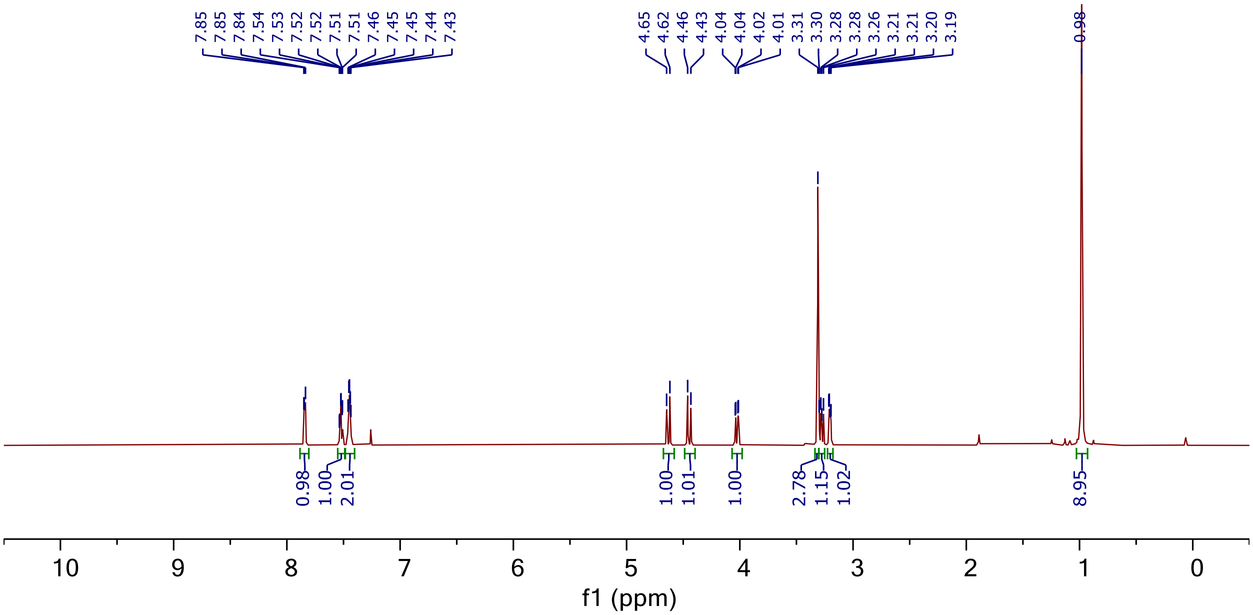

^{13}C NMR (151 MHz, $CDCl_3$):

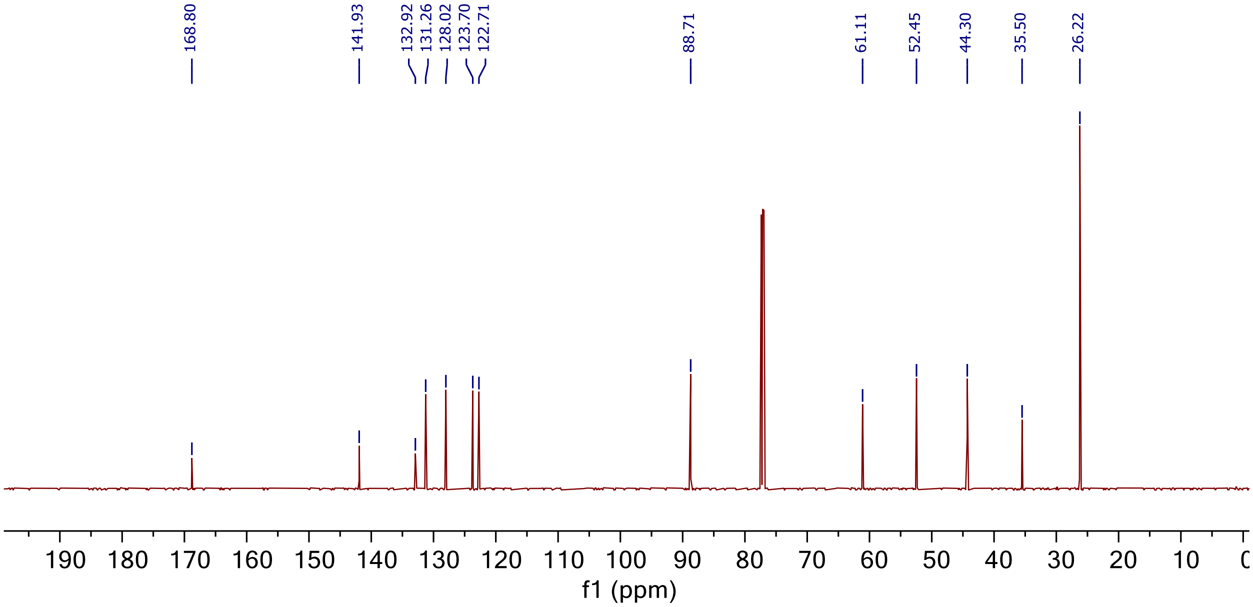

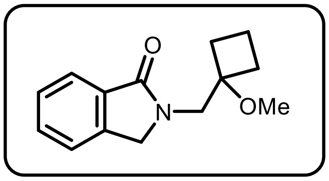

2-((1-methoxycyclobutyl)methyl)isoindolin-1-one (6b).

^{1}H NMR (600 MHz, $CDCl_3$):

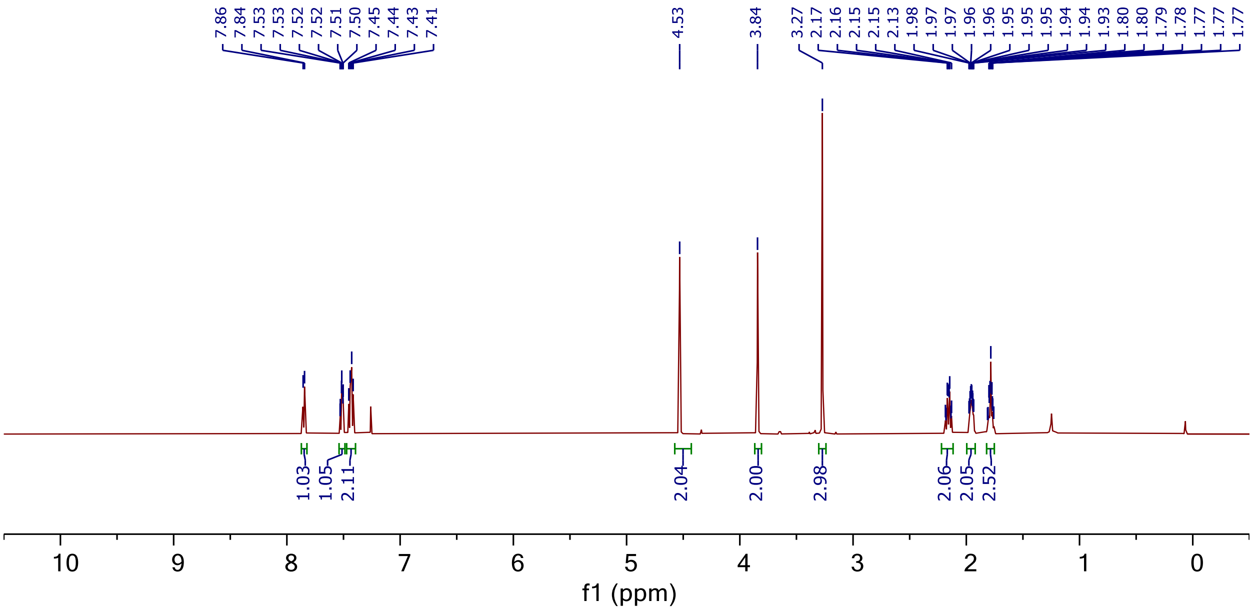

^{13}C NMR (151 MHz, $CDCl_3$):

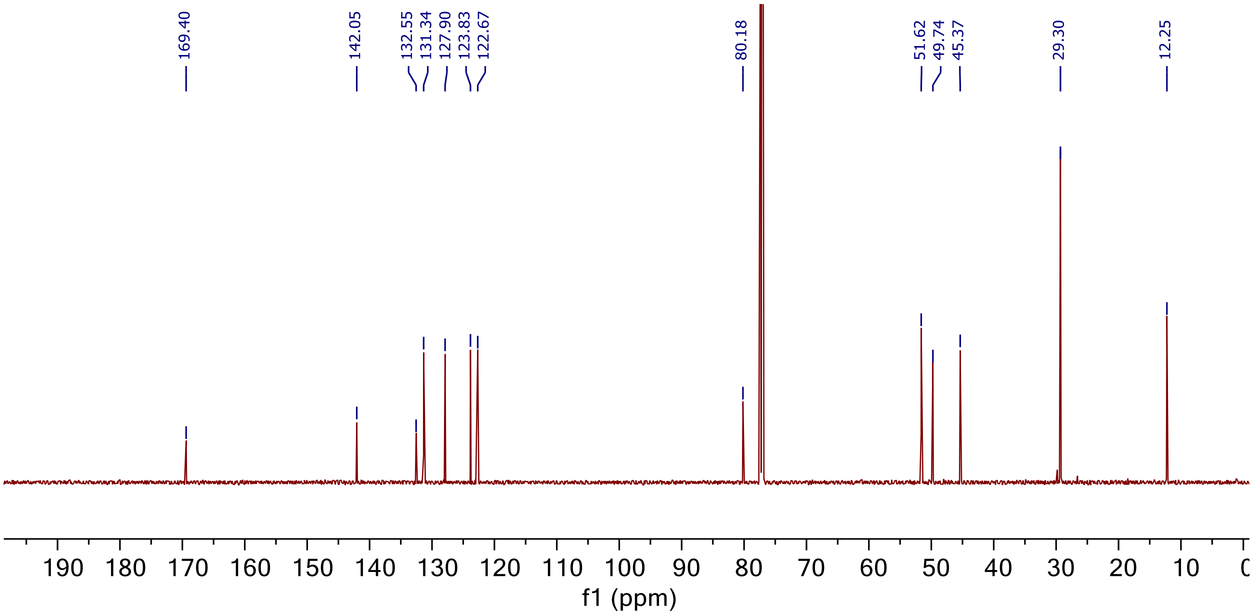

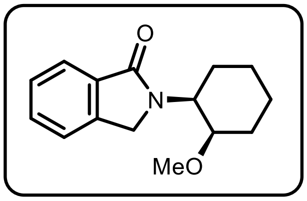

***cis*-2-(2-methoxycyclohexyl)isoindolin-1-one (7b)**.

¹H NMR (600 MHz, $CDCl_3$):

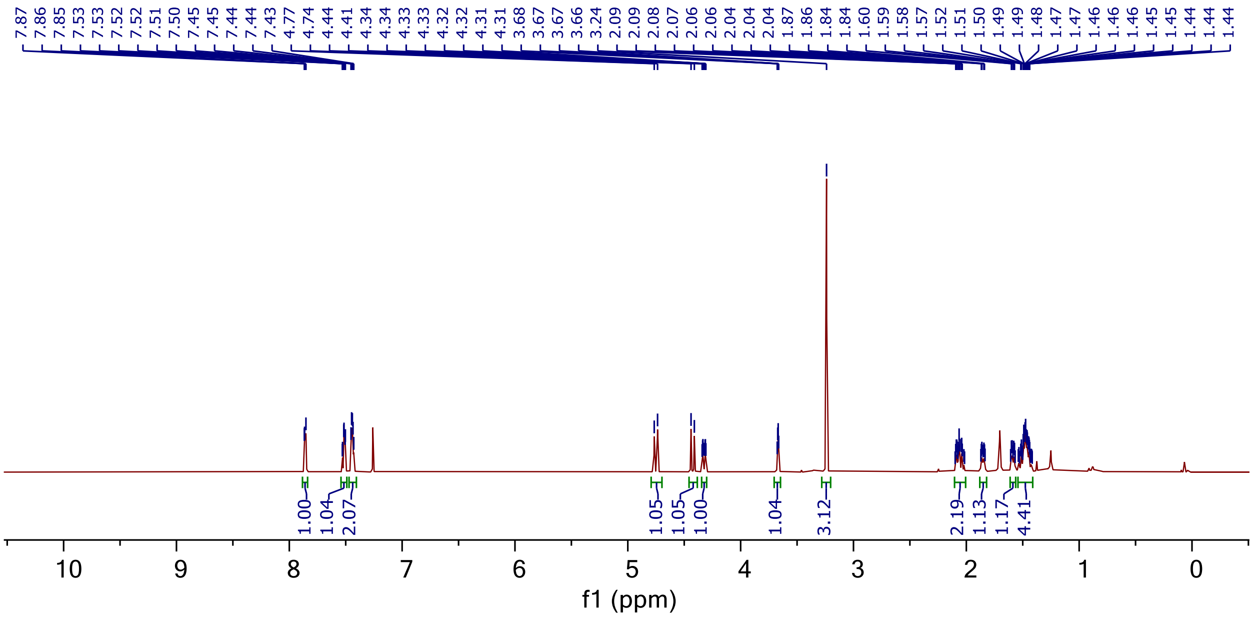

¹³C NMR (151 MHz, $CDCl_3$):

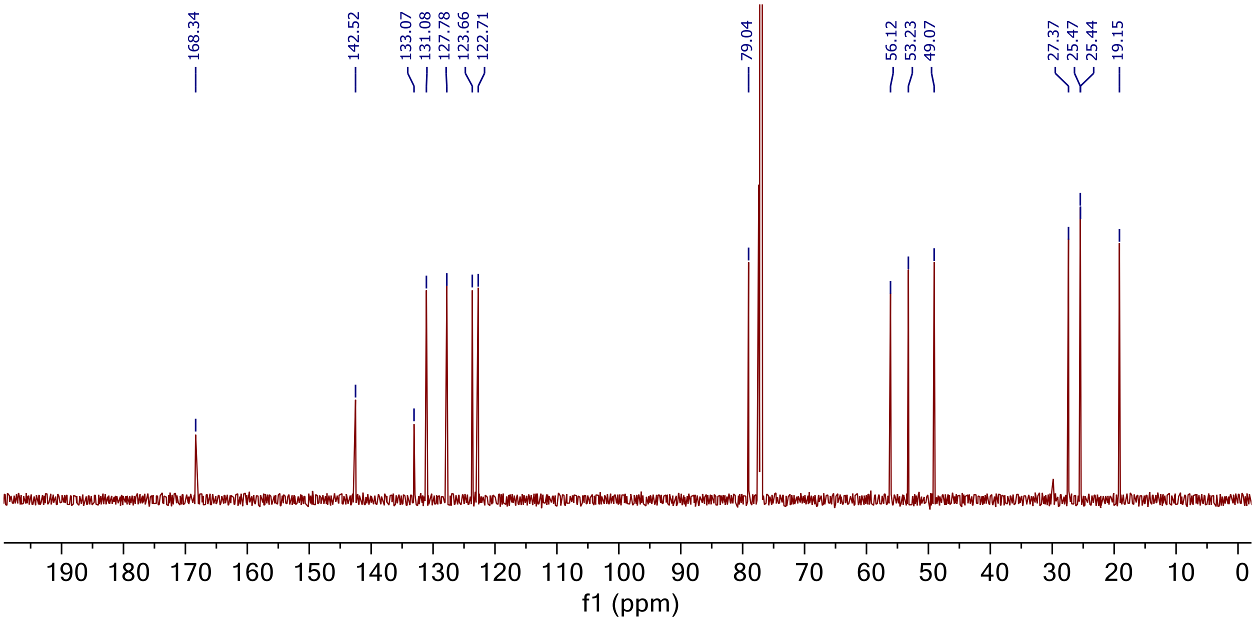

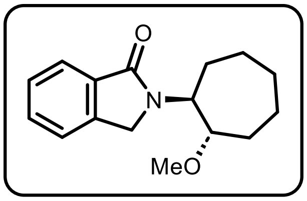

trans-2-(2-methoxycycloheptyl)isoindolin-1-one (8b).

^{1}H NMR (600 MHz, $CDCl_3$):

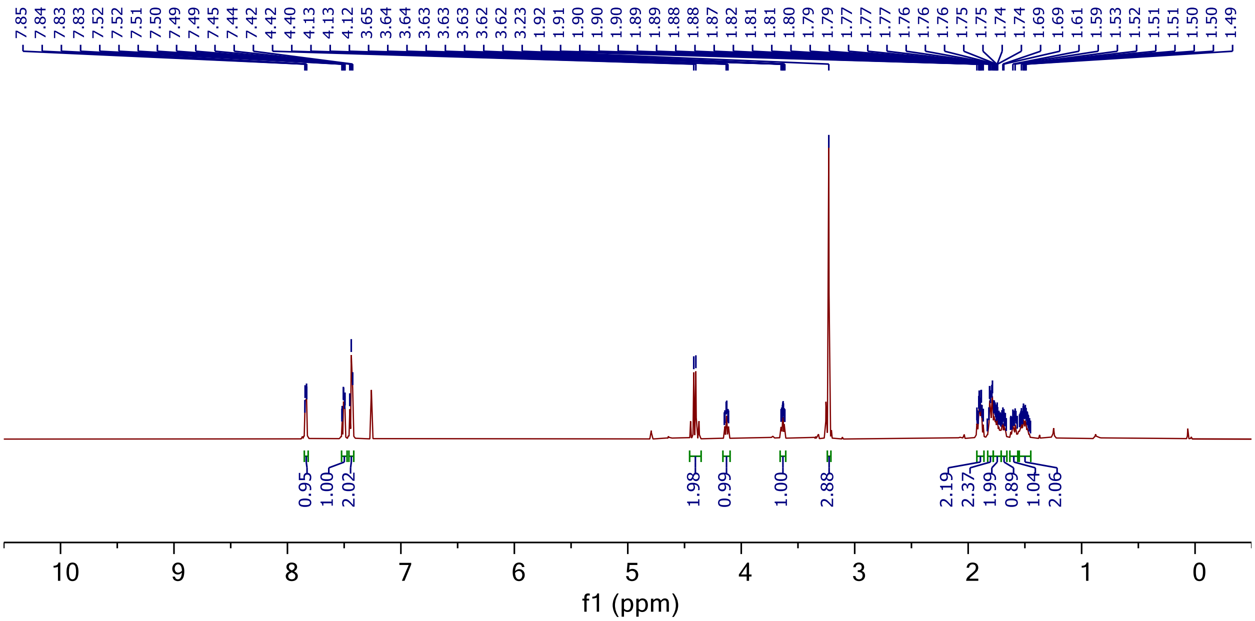

^{13}C NMR (151 MHz, $CDCl_3$):

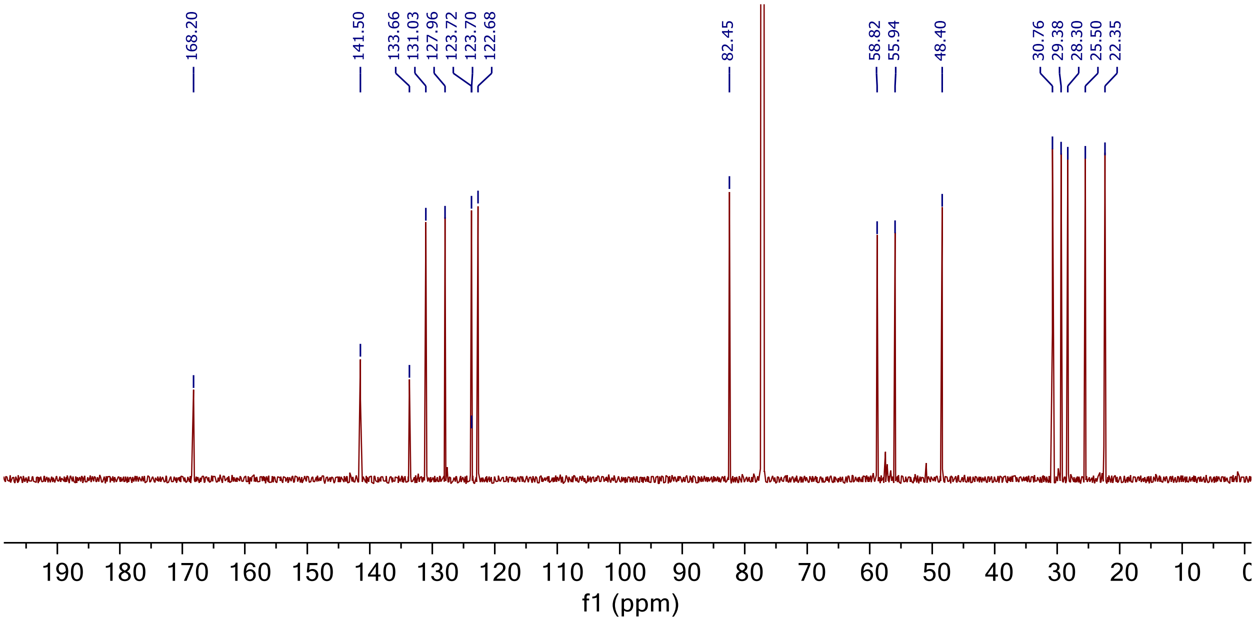

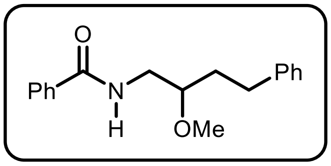

***N*-(2-methoxy-4-phenylbutyl)benzamide (9b)**.

^{1}H NMR (600 MHz, $CDCl_3$):

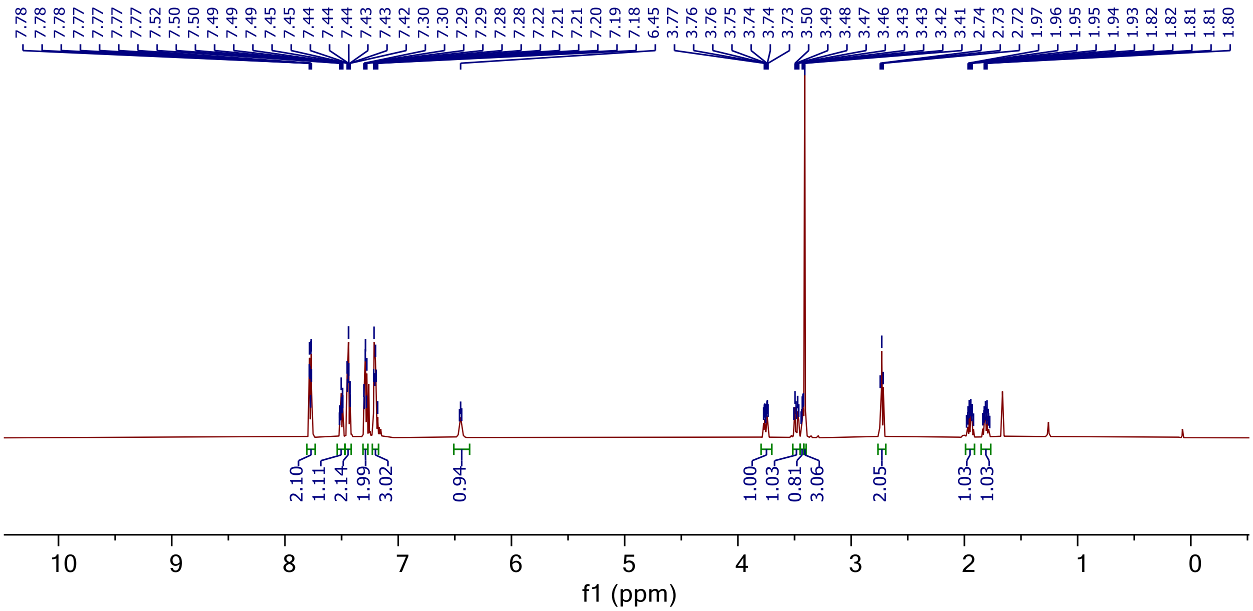

^{13}C NMR (151 MHz, $CDCl_3$):

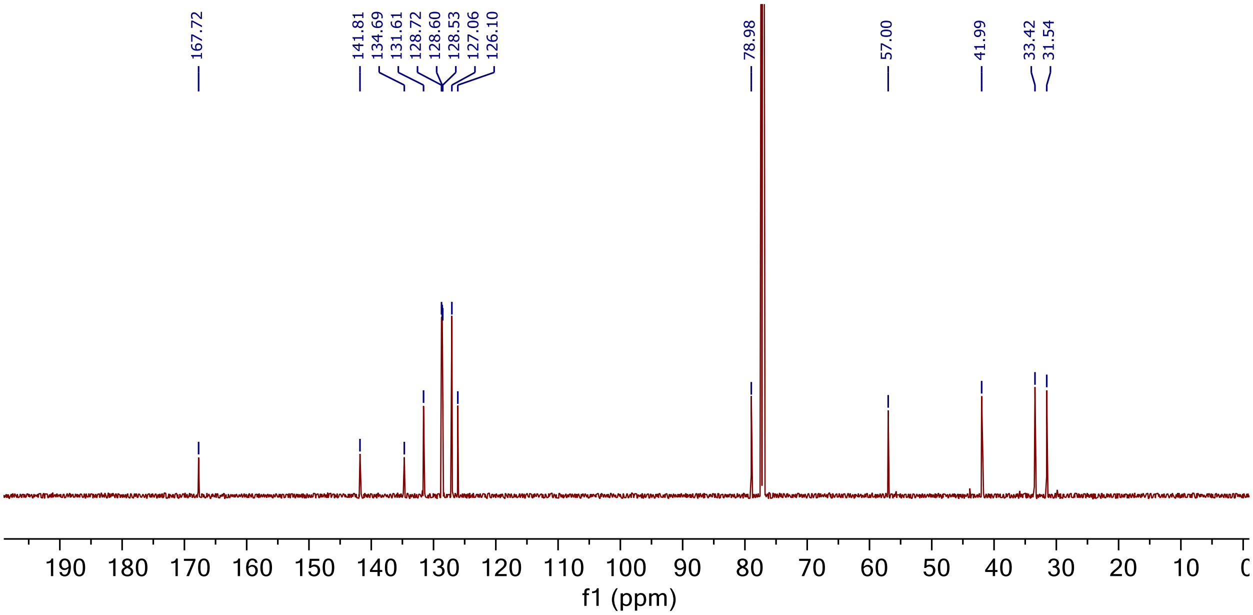

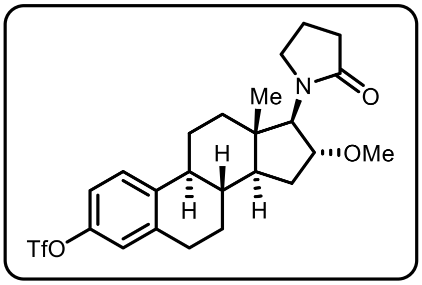

(8R,9S,13S,14S)-16-methoxy-13-methyl-17-(2-oxopyrrolidi n-1-yl)-7,8,9,11,12,13,14,15,16,17-decahydro-6H-cyclopen ta[a]phenanthren-3-yl trifluoromethanesulfonate (10b).

^{1}H NMR (600 MHz, $CDCl_3$):

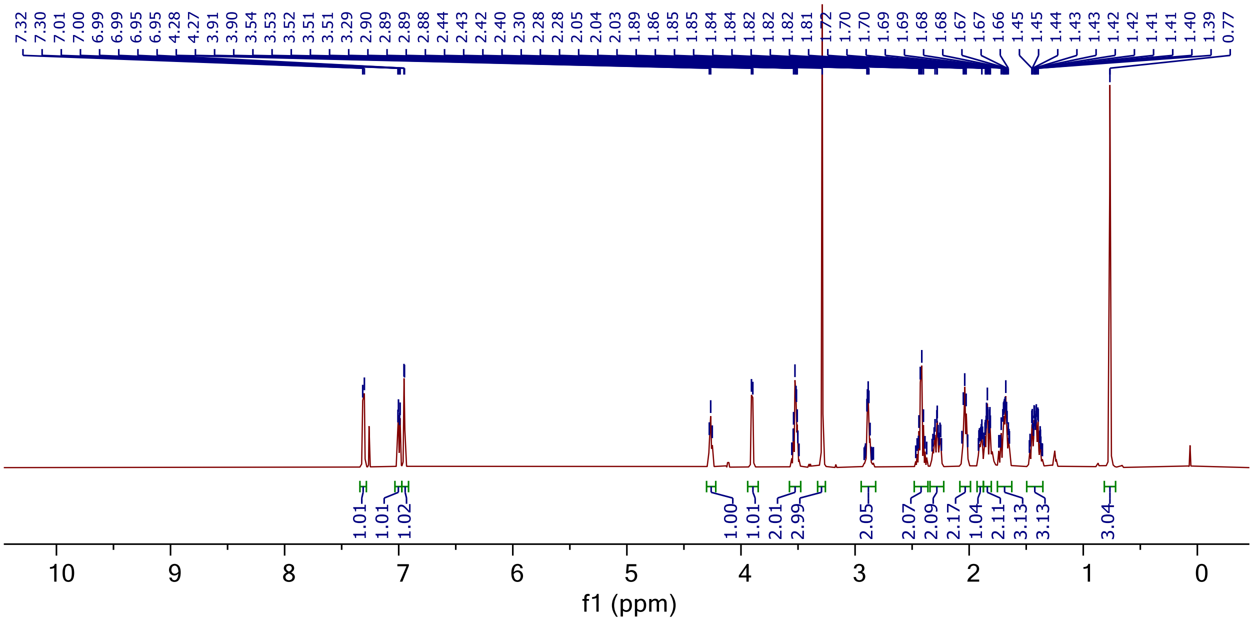

^{13}C NMR (151 MHz, $CDCl_3$):

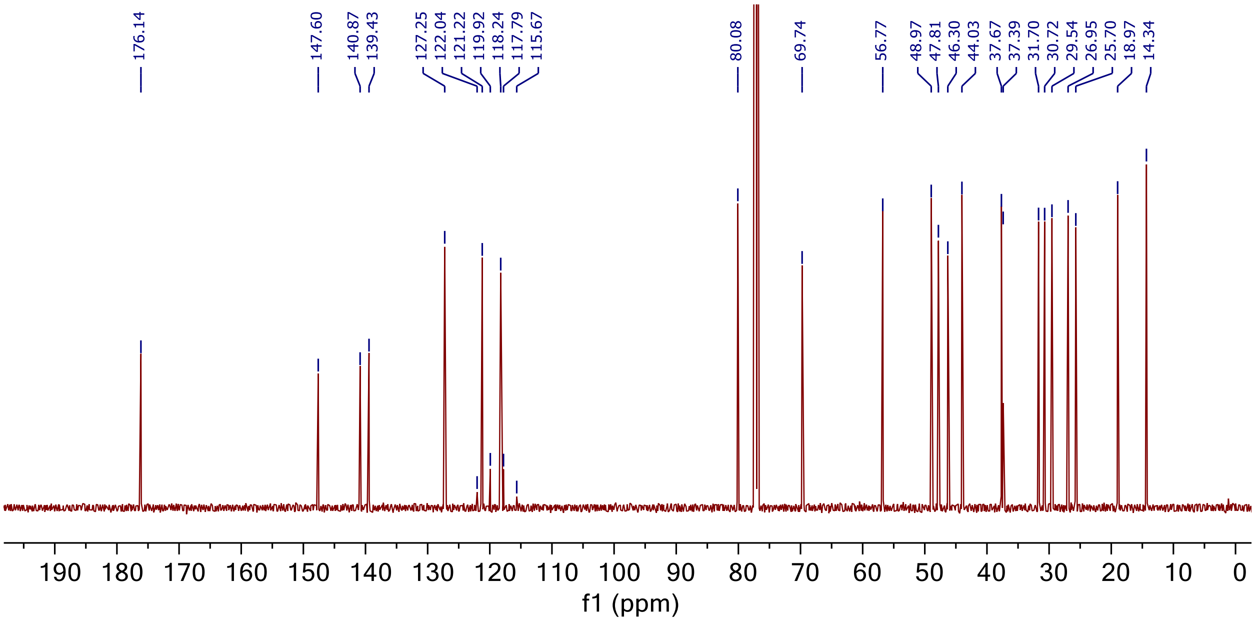

^{19}F NMR (471 MHz, $CDCl_3$):

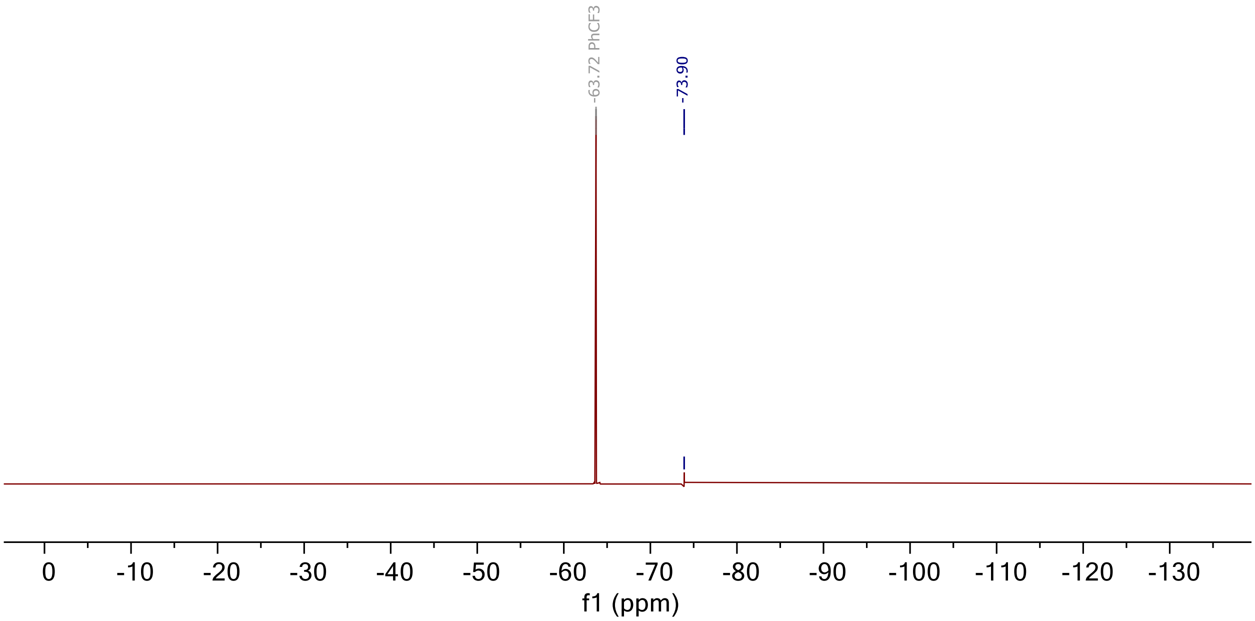

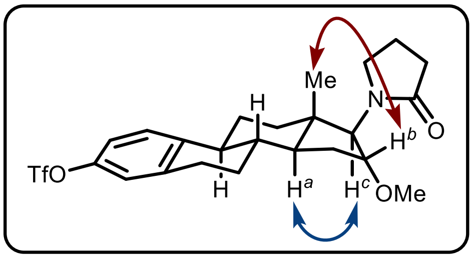

NOESY (600 MHz, $CDCl_3$):
Major diastereomer

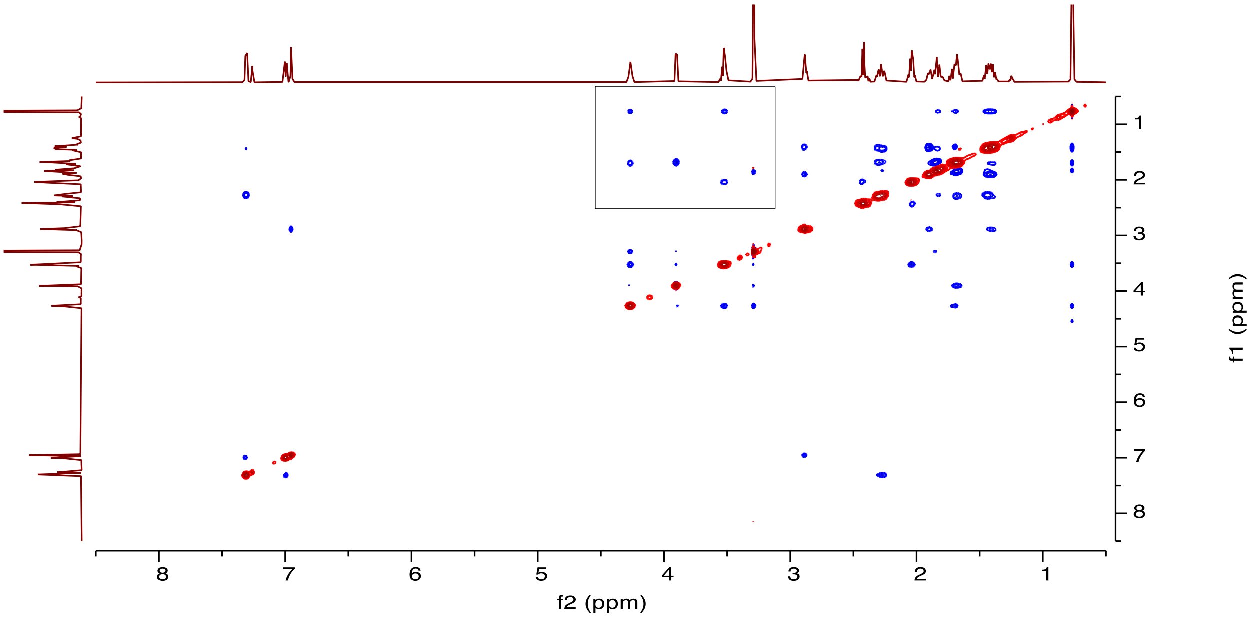

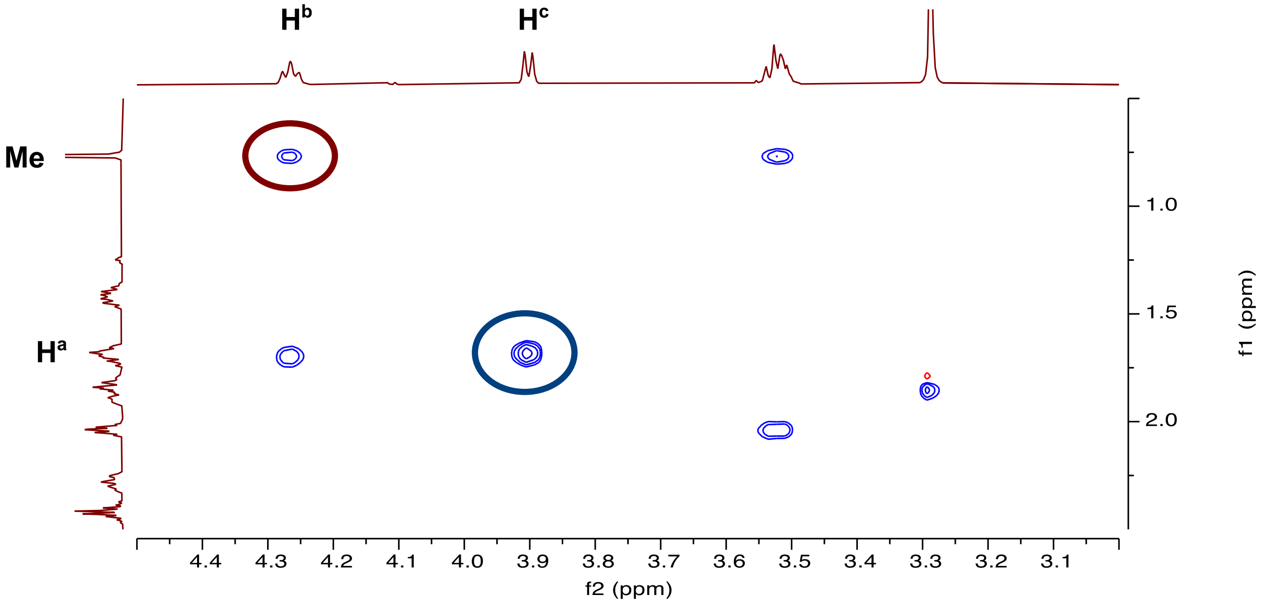

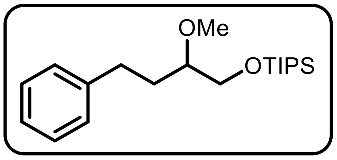

triisopropyl(2-methoxy-4-phenylbutoxy)silane (11b).

^{1}H NMR (600 MHz, $CDCl_3$):

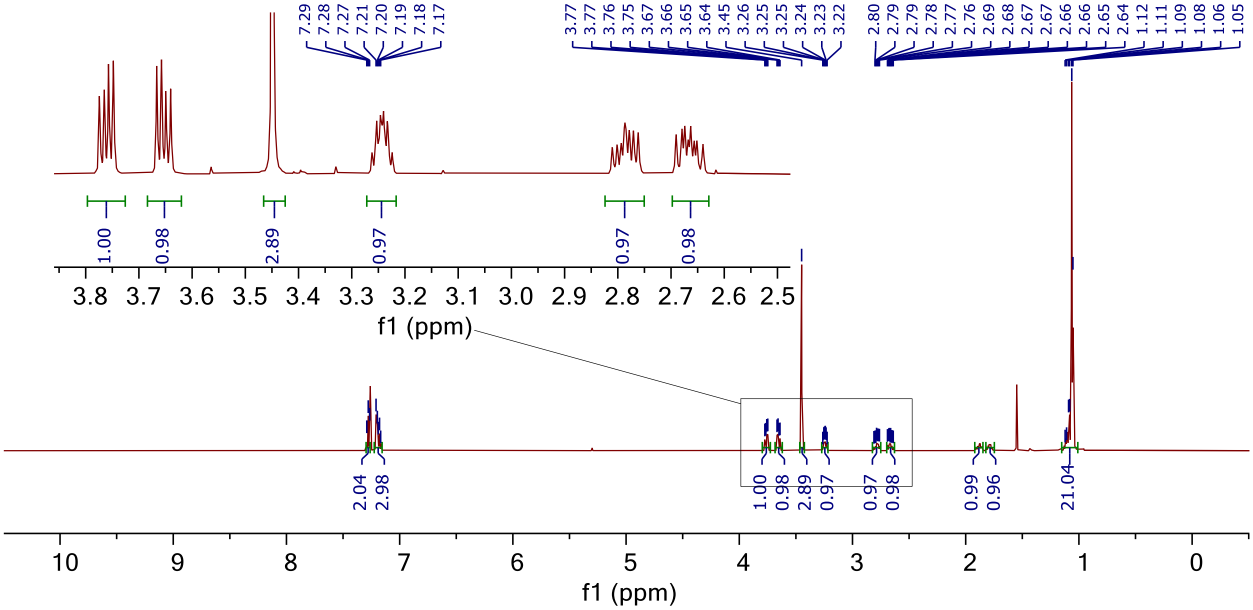

^{13}C NMR (151 MHz, $CDCl_3$):

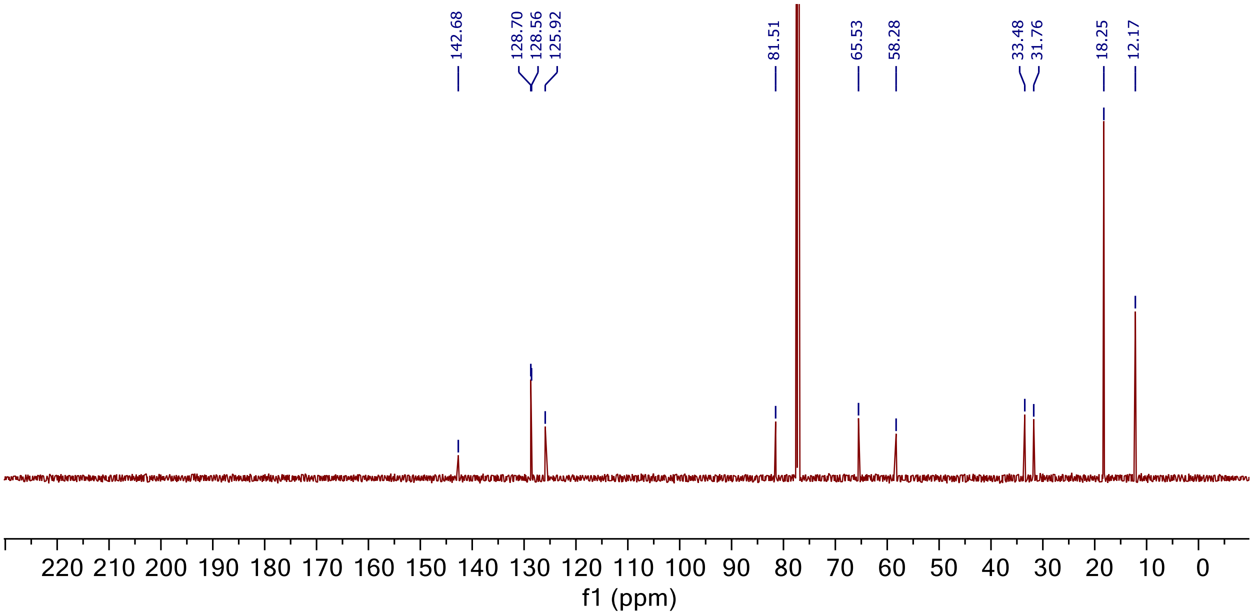

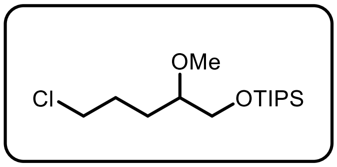

((5-chloro-2-methoxypentyl)oxy)triisopropylsilane (12b).

[1]***H NMR*** (600 MHz, $CDCl_3$):

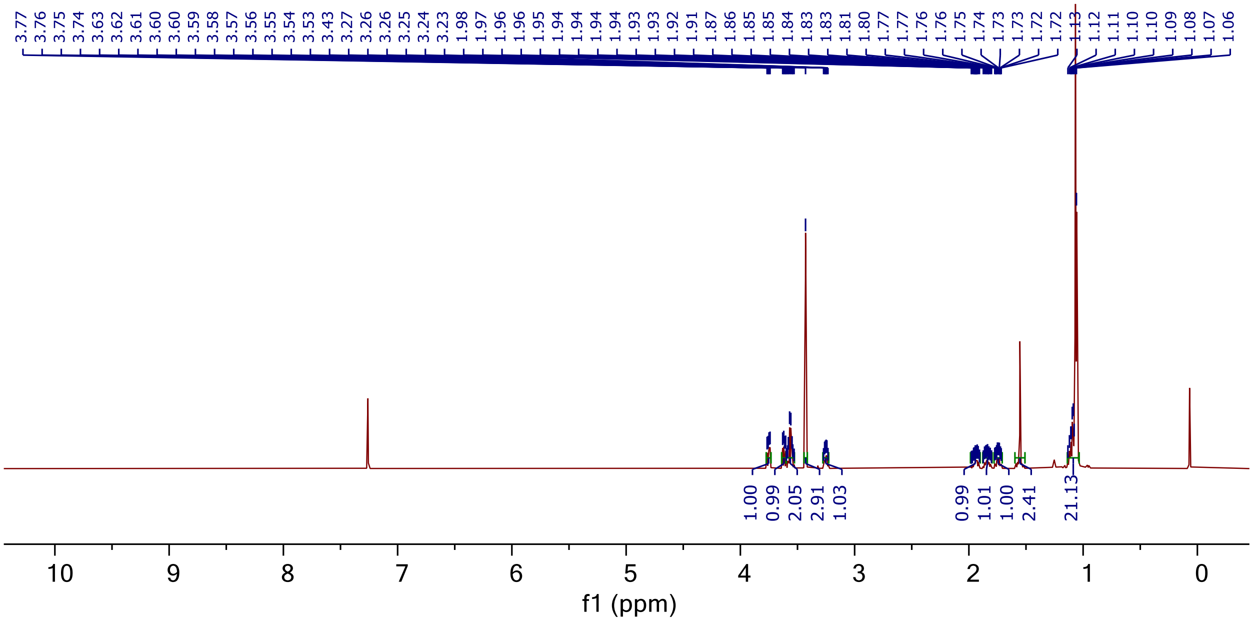

[13]***C NMR*** (151 MHz, $CDCl_3$):

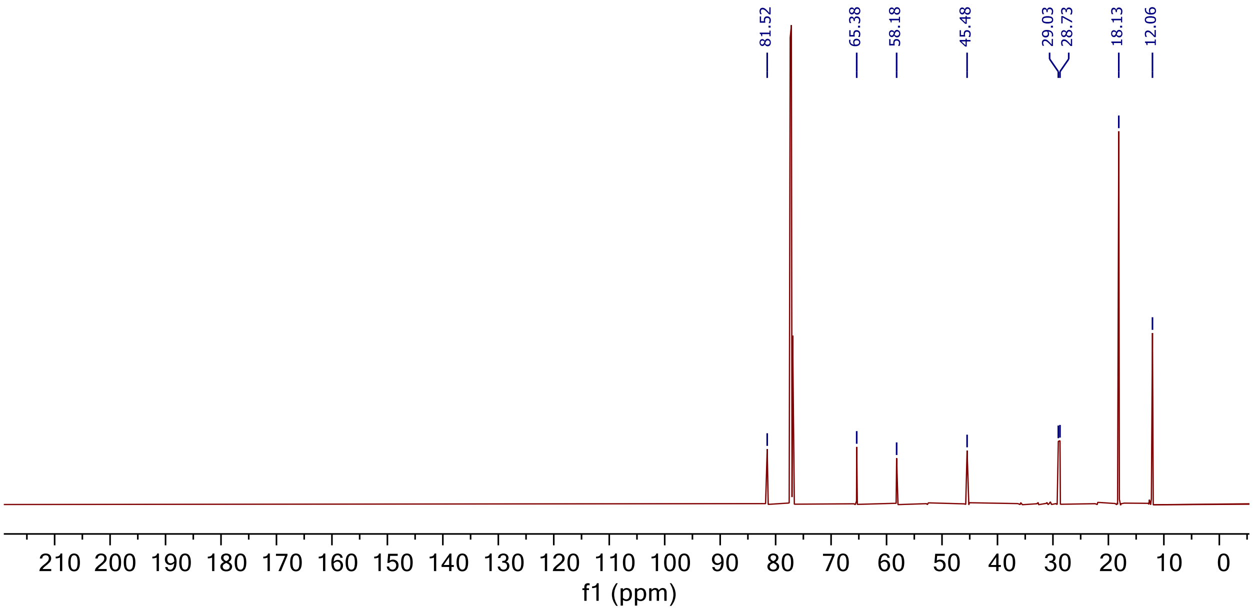

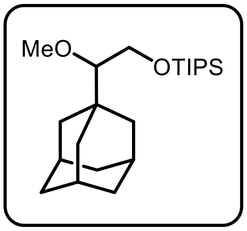

(2-adamantan-1-yl-2-methoxyethoxy)triisopropylsilane (13b)

¹H NMR (600 MHz, $CDCl_3$):

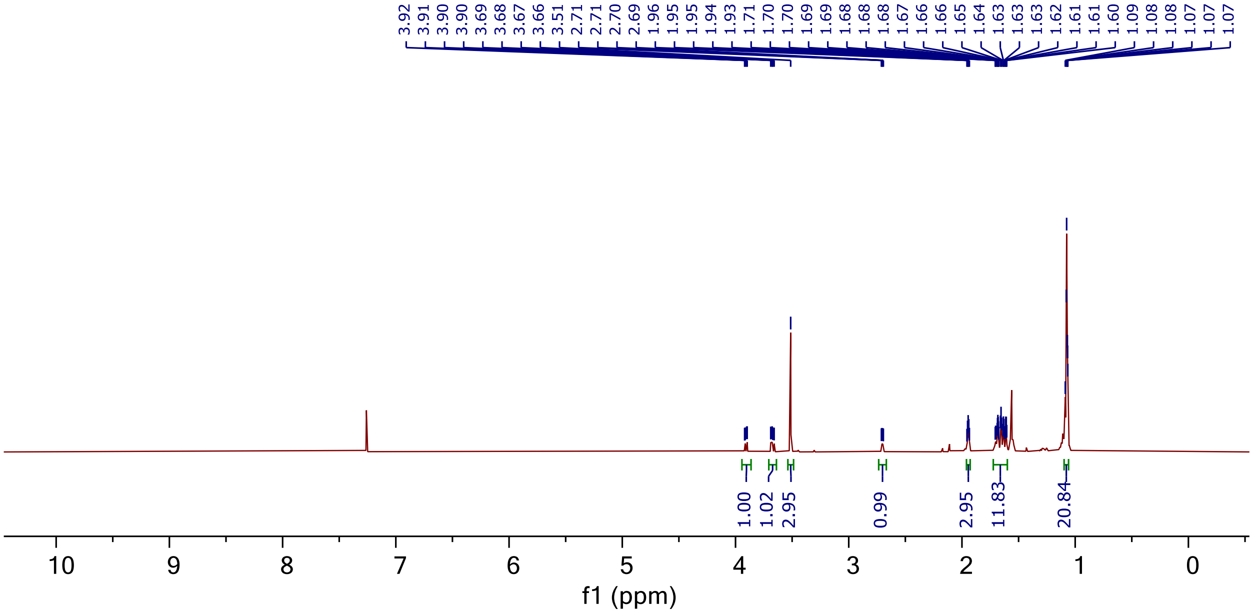

¹³C NMR (151 MHz, $CDCl_3$):

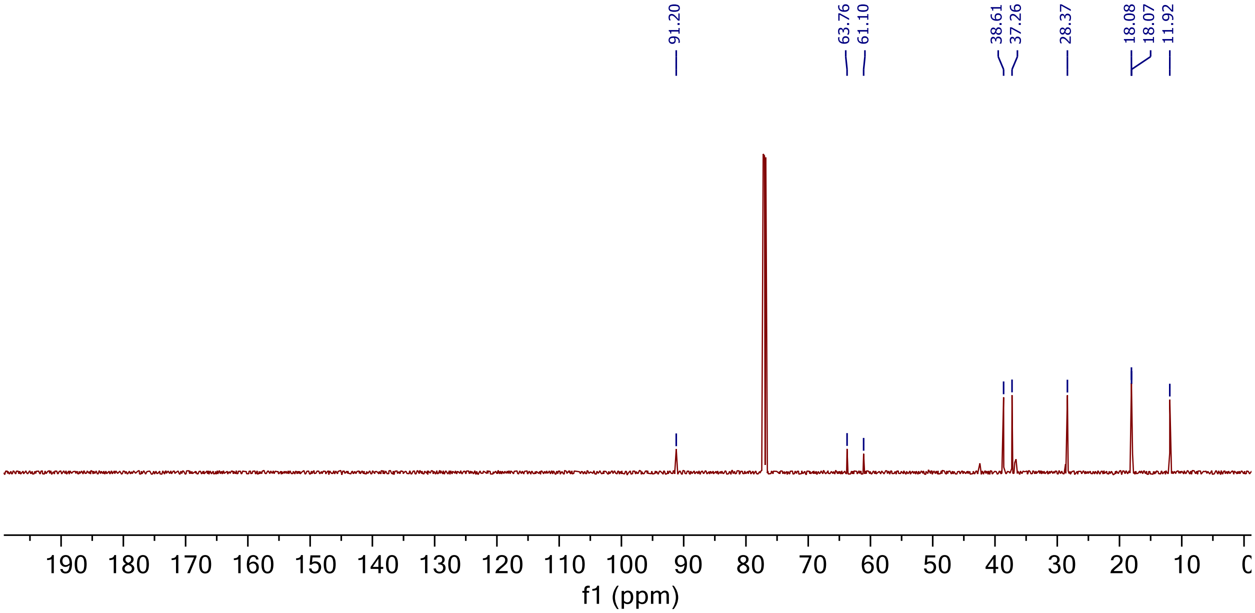

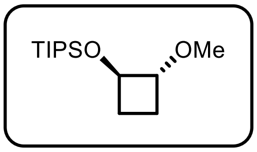

trans-triisopropyl(2-methoxycyclobutoxy)silane (14b).

^{1}H NMR (600 MHz, $CDCl_3$):

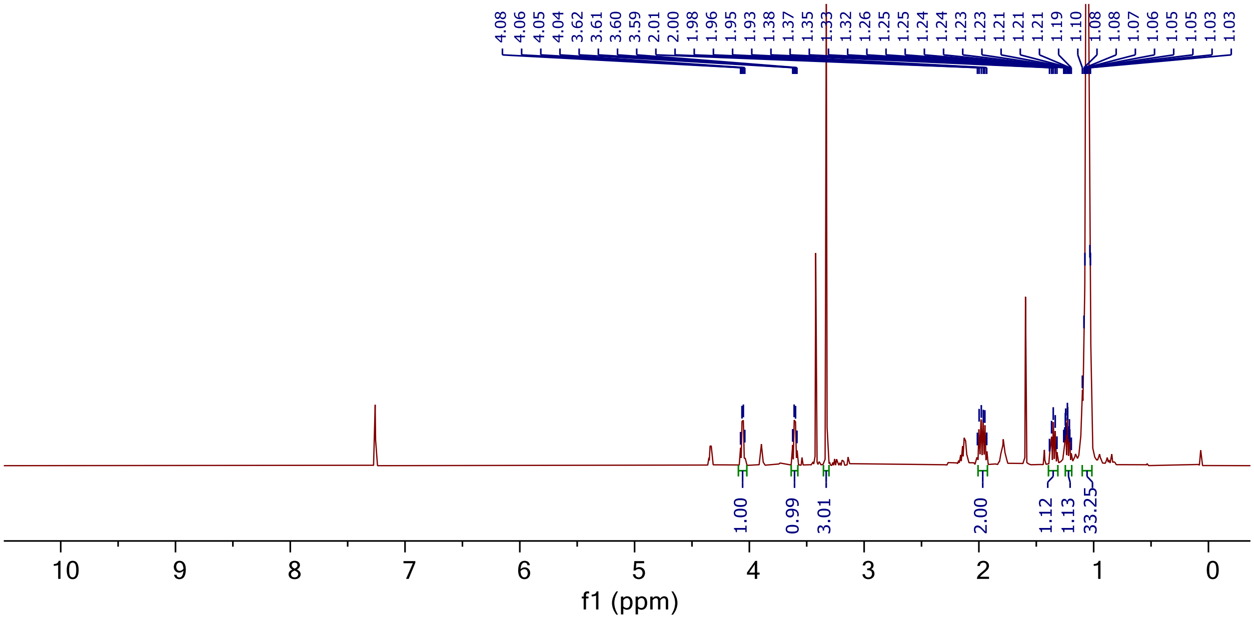

^{13}C NMR (151 MHz, $CDCl_3$):

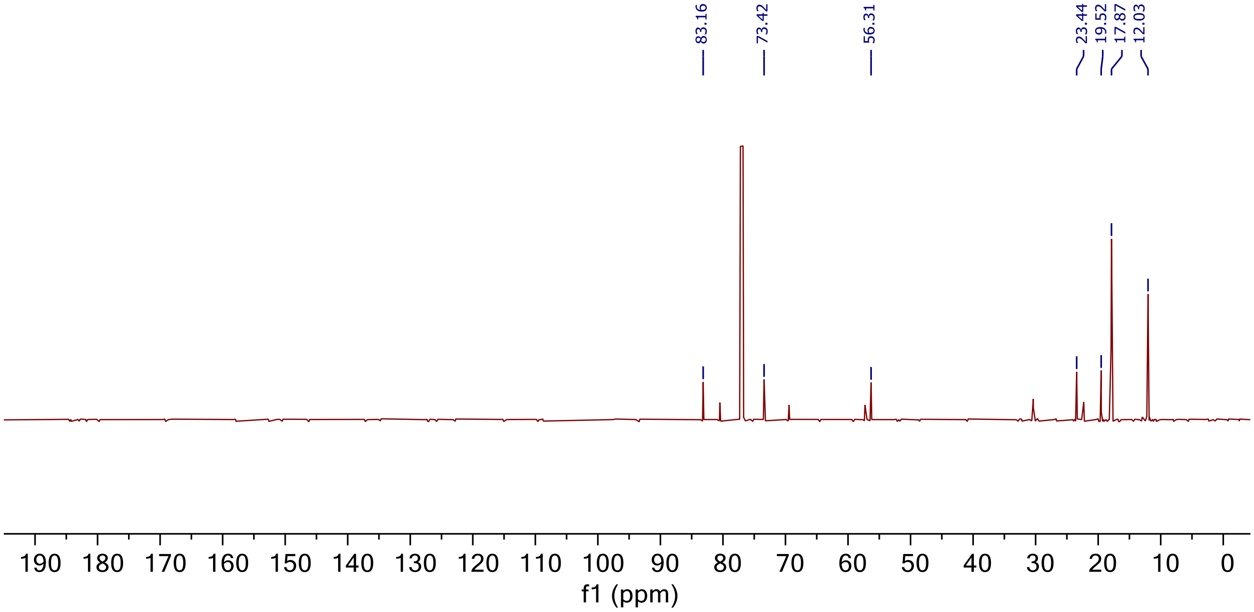

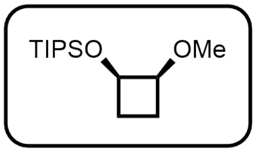

cis-triisopropyl(2-methoxycyclobutoxy)silane (14b).

^{1}H NMR (600 MHz, $CDCl_3$):

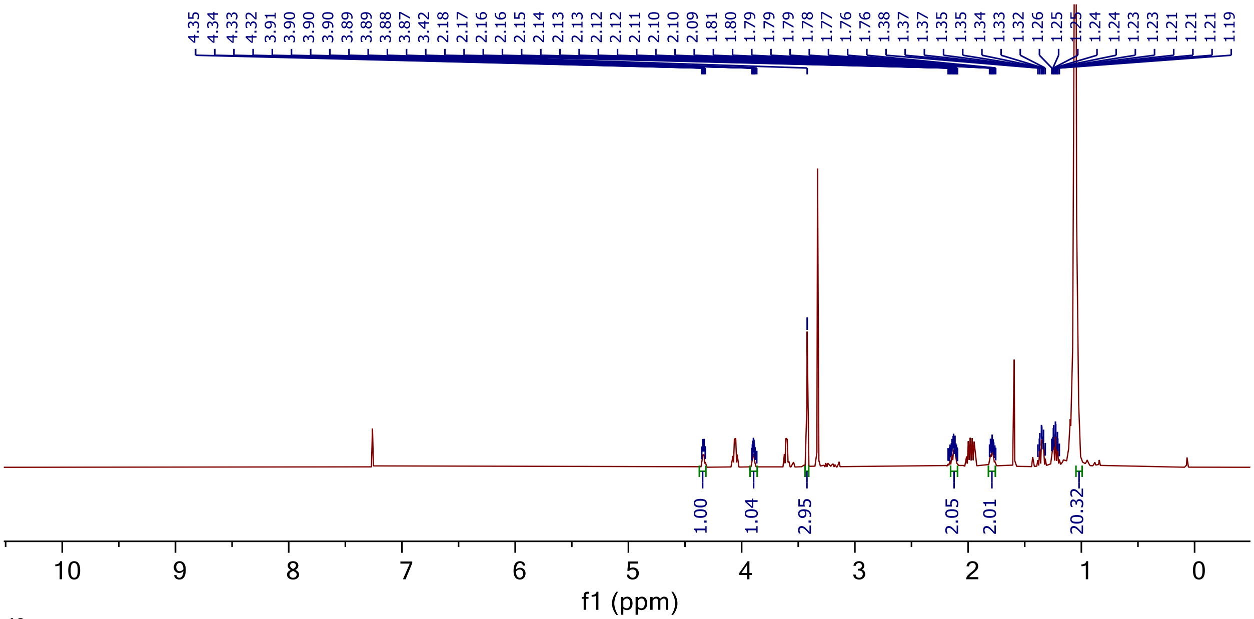

^{13}C NMR (151 MHz, $CDCl_3$):

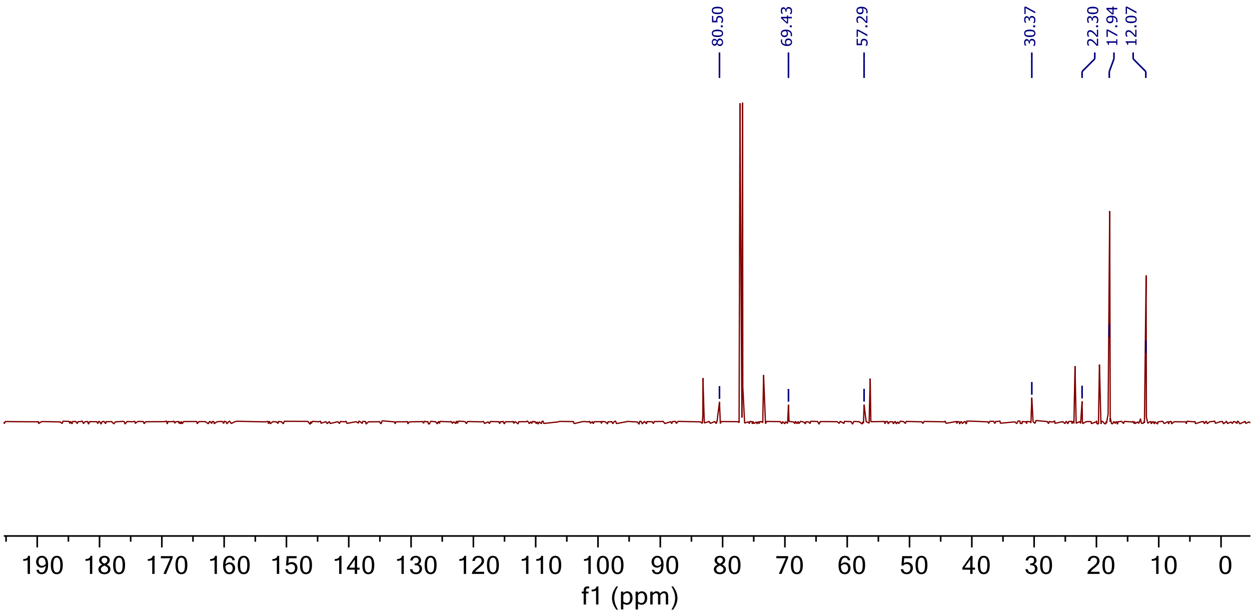

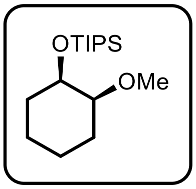

***cis*-triisopropyl-2-methoxycyclohexyl)oxy)silane (15b)**

^{1}H NMR (600 MHz, $CDCl_3$):

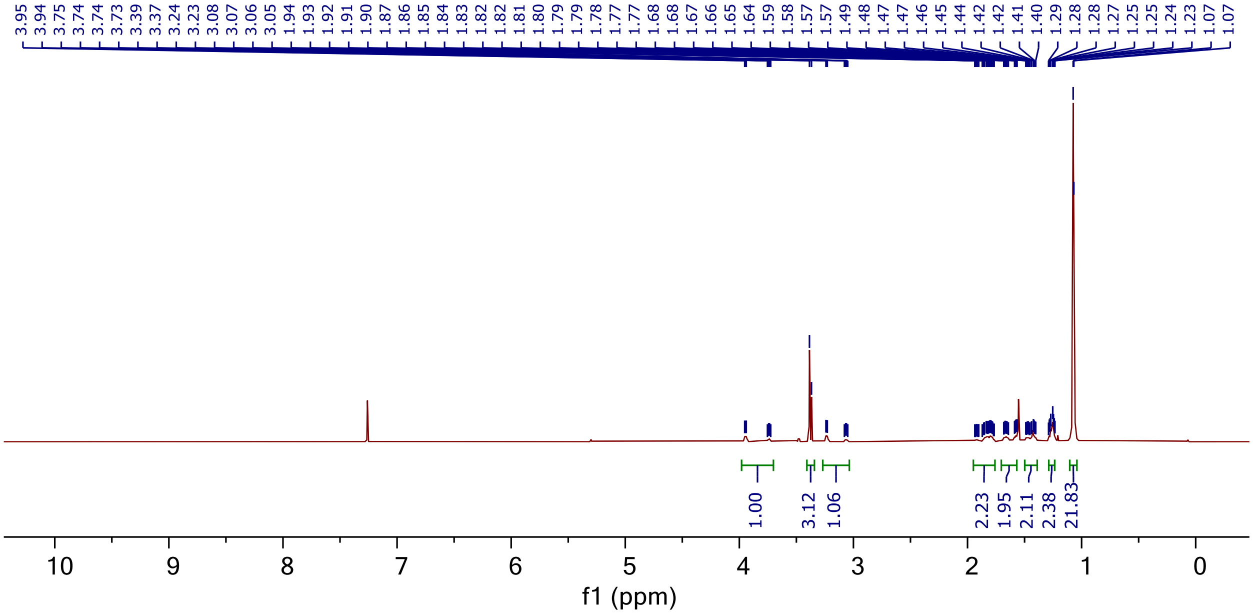

^{13}C NMR (151 MHz, $CDCl_3$):

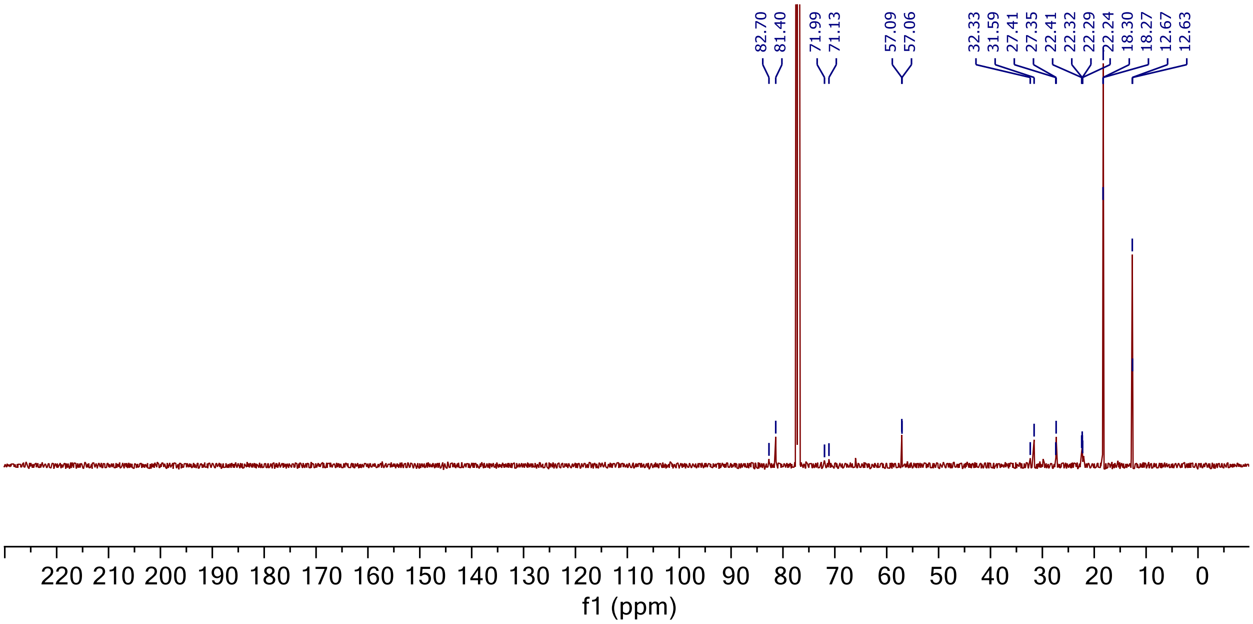

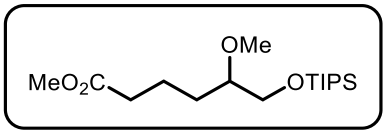

methyl 5-methoxy-6-((triisopropylsilyl)oxy)hexanoate (16b).

^{1}H NMR (600 MHz, $CDCl_3$):

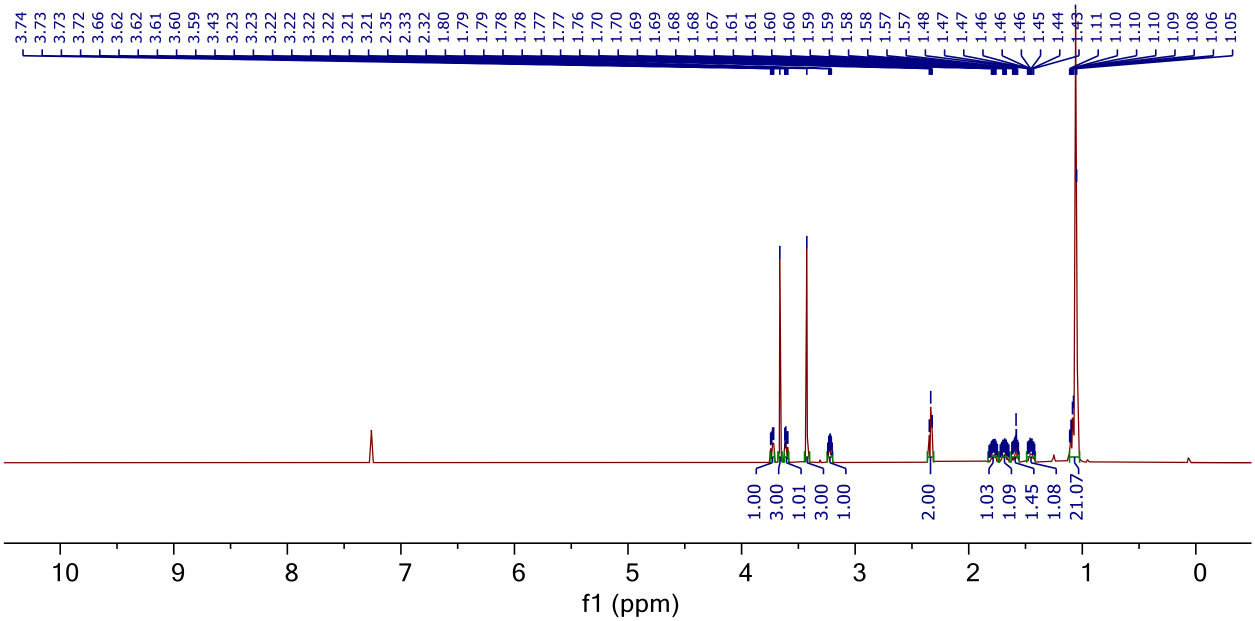

^{13}C NMR (151 MHz, $CDCl_3$):

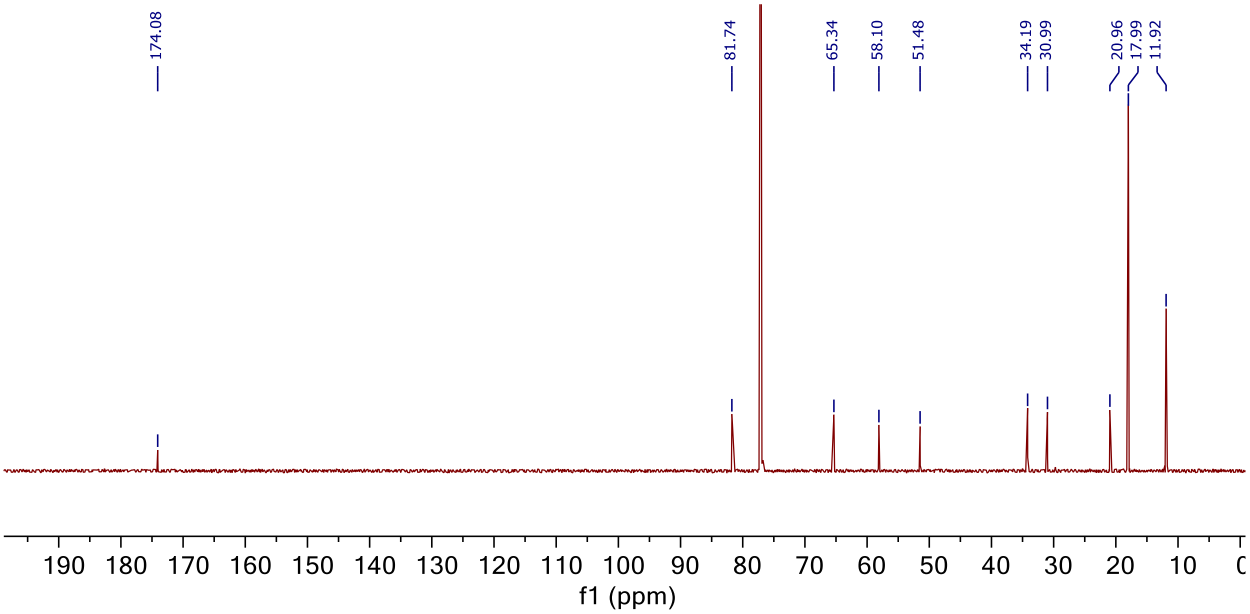

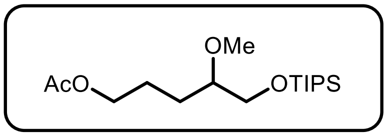

4-methoxy-5-((triisopropylsilyl)oxy)pentyl acetate (17b).

^{1}H NMR (600 MHz, $CDCl_3$):

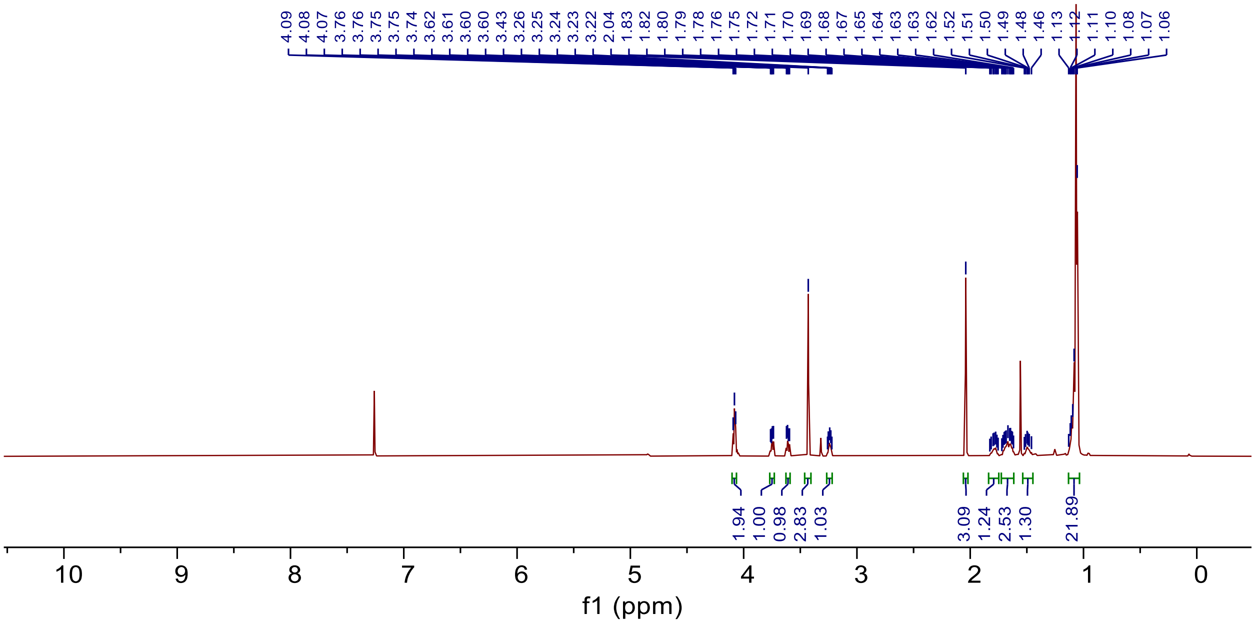

^{13}C NMR (151 MHz, $CDCl_3$):

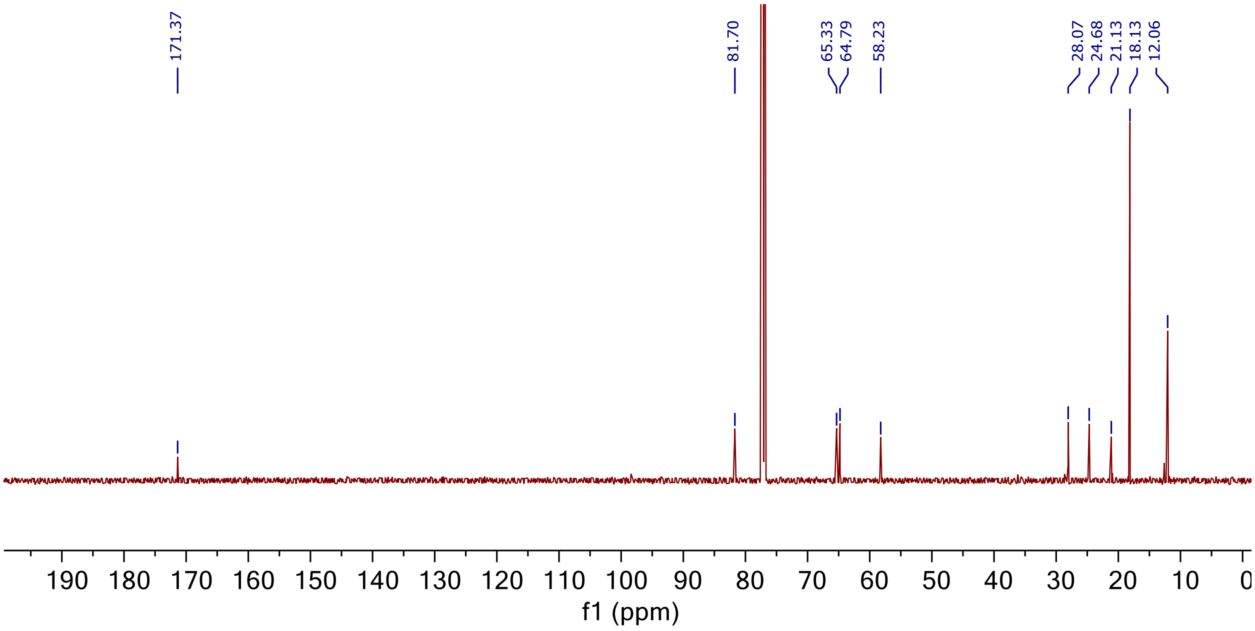

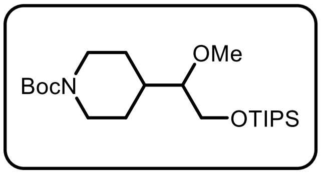

tert-butyl 4-(1-methoxy-2-((triisopropylsilyl)oxy) ethyl)piperidine-1-carboxylate (18b).

[1]***H NMR*** (600 MHz, $CDCl_3$):

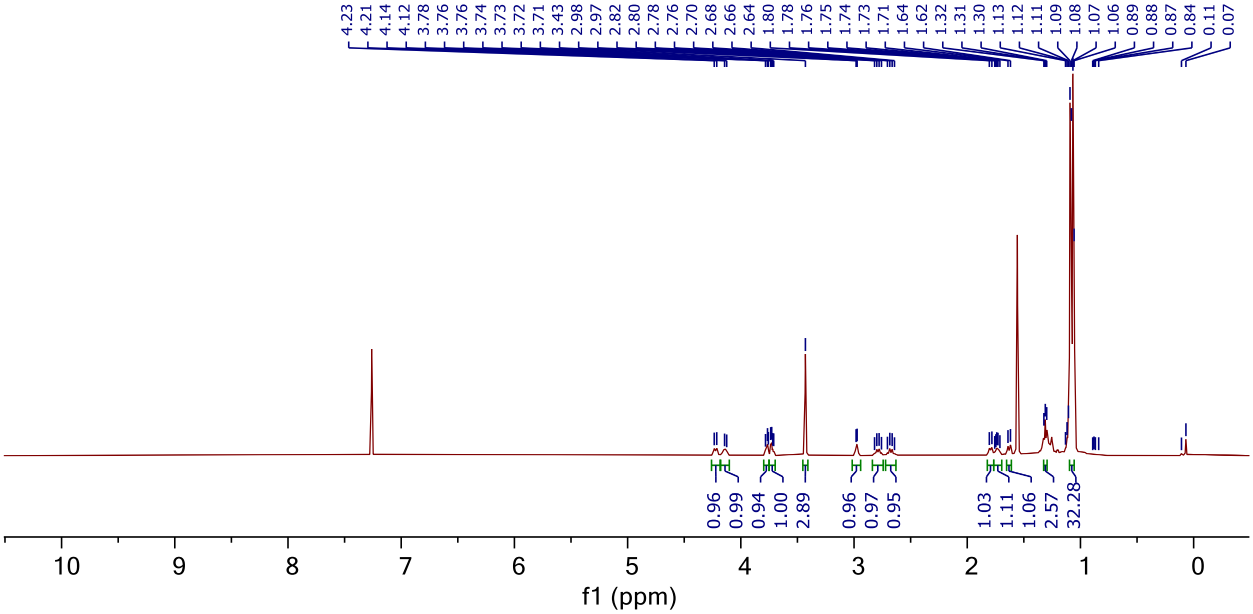

[13]***C NMR*** (151 MHz, $CDCl_3$):

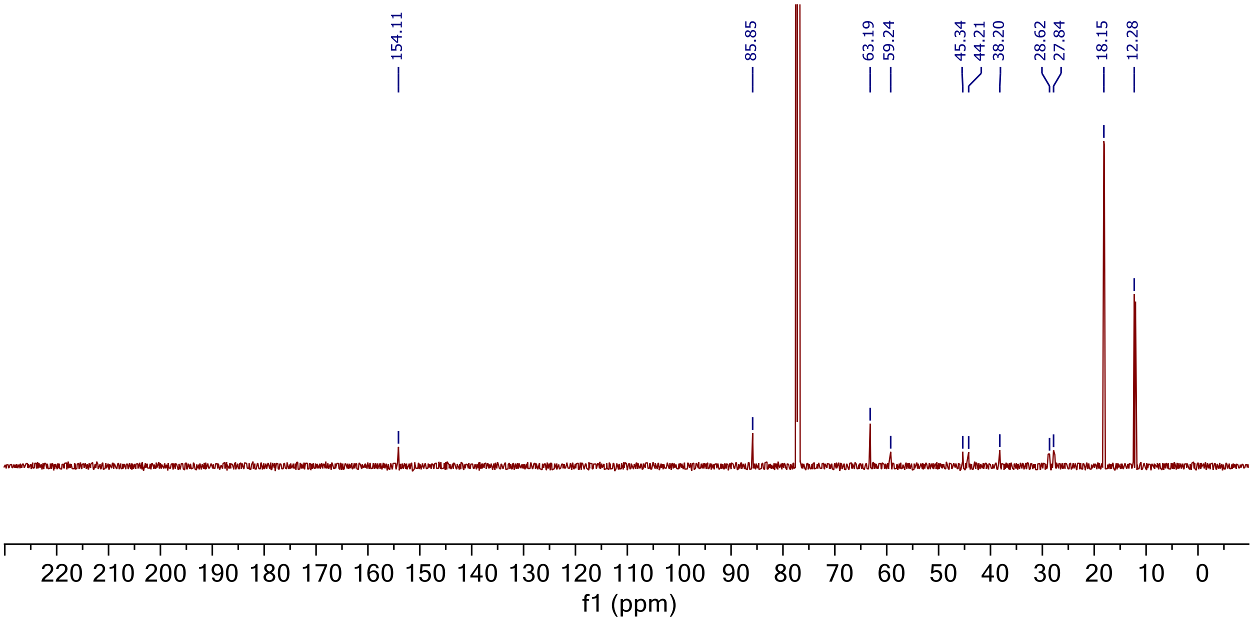

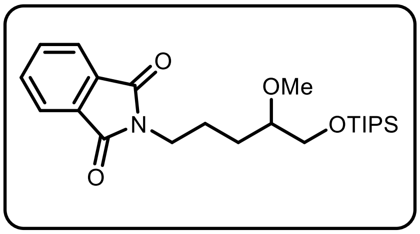

2-(4-methoxy-5-((triisopropylsilyl)oxy)pentyl)isoindo line-1,3-dione (19b)

^{1}H NMR (600 MHz, $CDCl_3$):

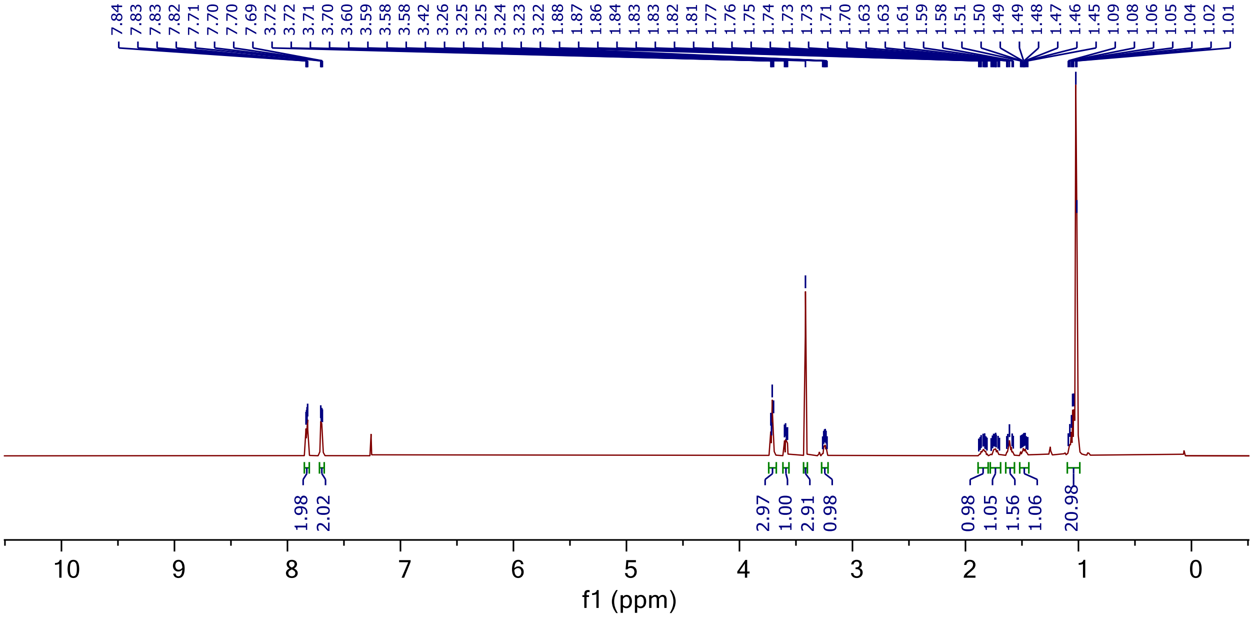

^{13}C NMR (151 MHz, $CDCl_3$):

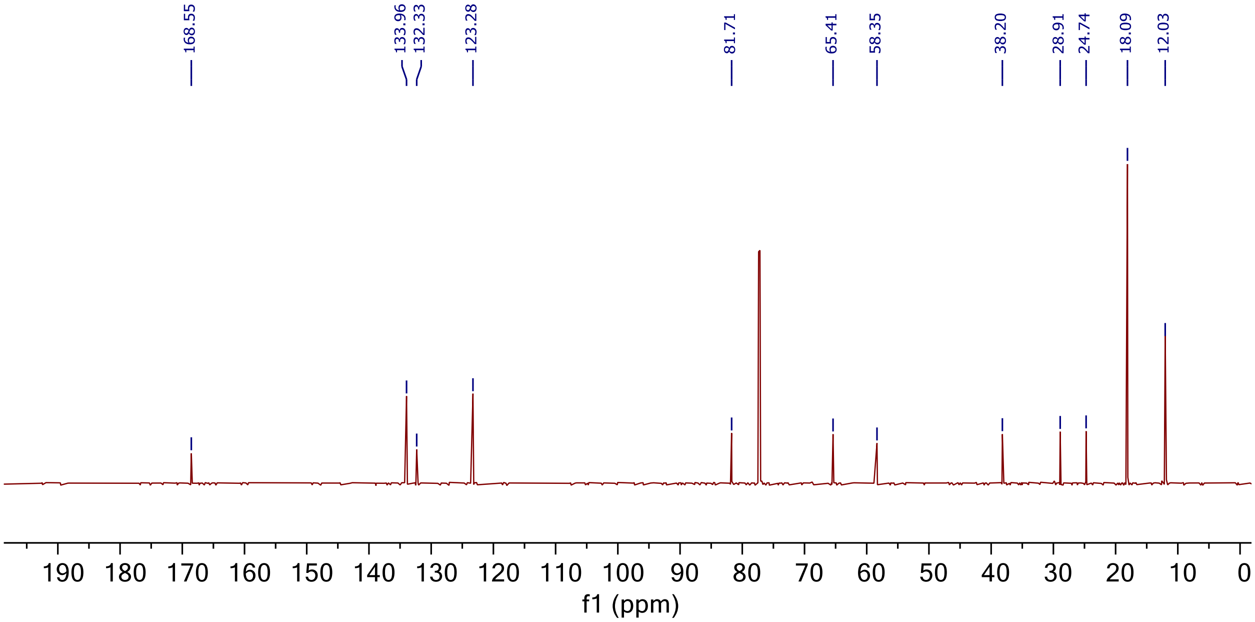

3-(3-methoxy-4-((triisopropylsilyl)oxy)butyl)pyridine (20b)

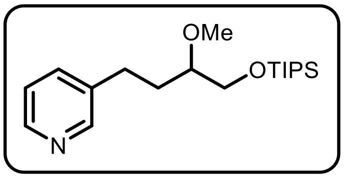

1H NMR (600 MHz, $CDCl_3$):

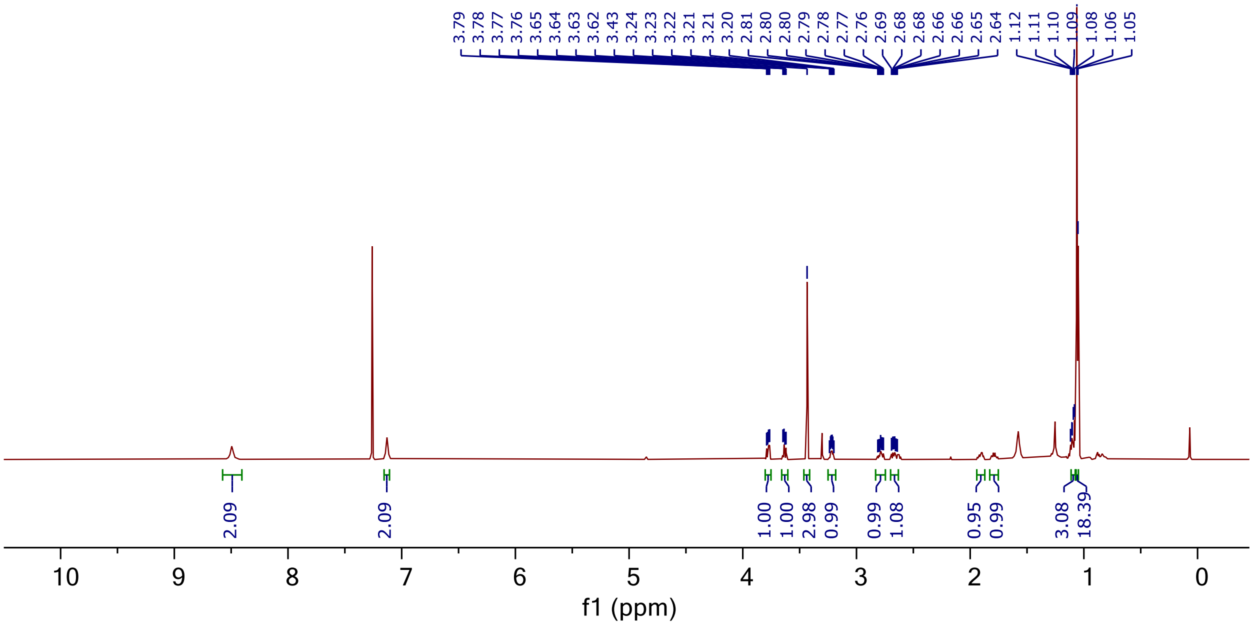

13C NMR (151 MHz, $CDCl_3$):

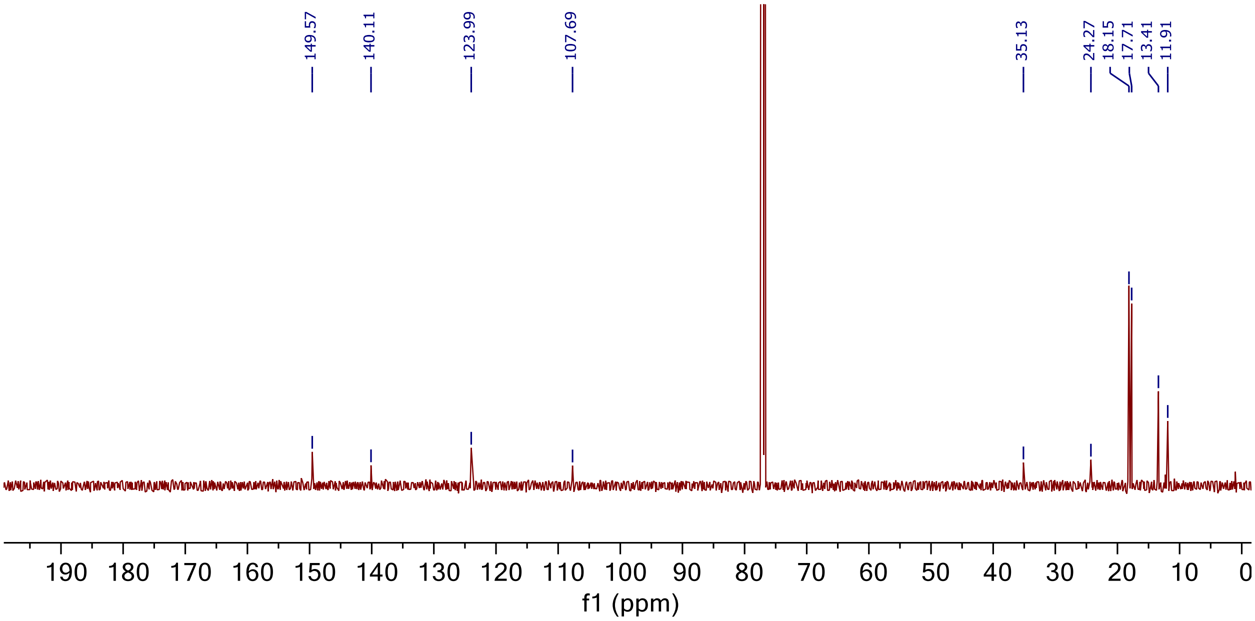

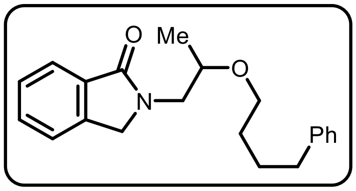

2-(2-(4-phenylbutoxy)propyl)isoindolin-1-one (21b).

1H NMR (600 MHz, $CDCl_3$):

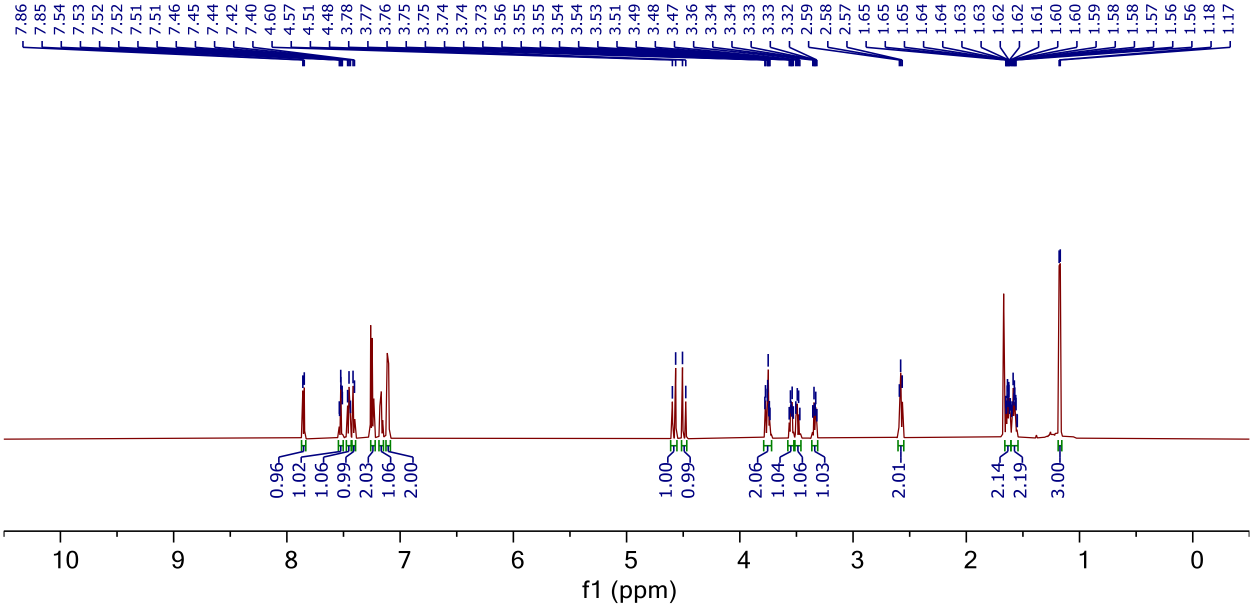

13C NMR (151 MHz, $CDCl_3$):

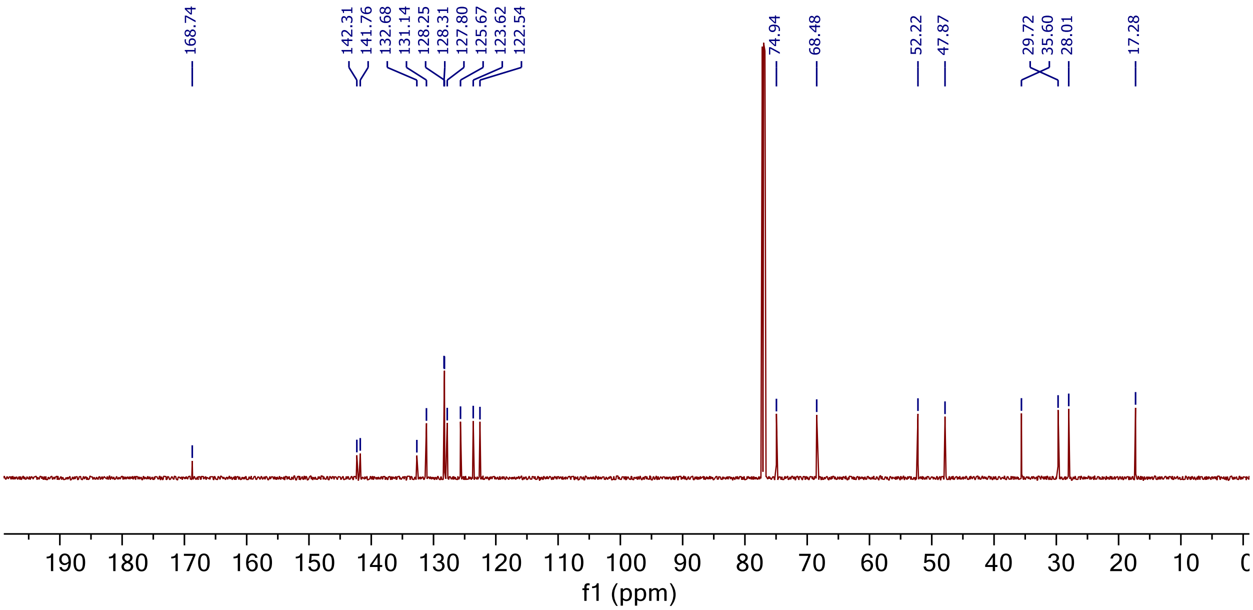

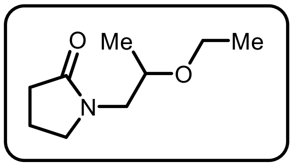

1-(2-ethoxypropyl)pyrrolidin-2-one (22b).

^{1}H NMR (600 MHz, $CDCl_3$):

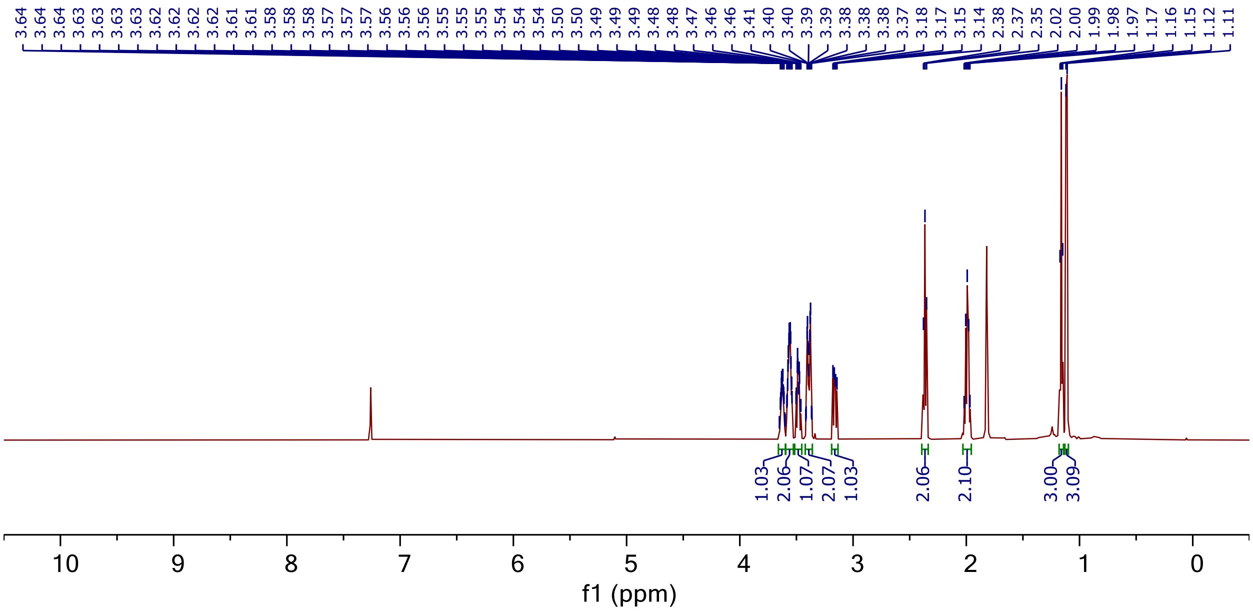

^{13}C NMR (151 MHz, $CDCl_3$):

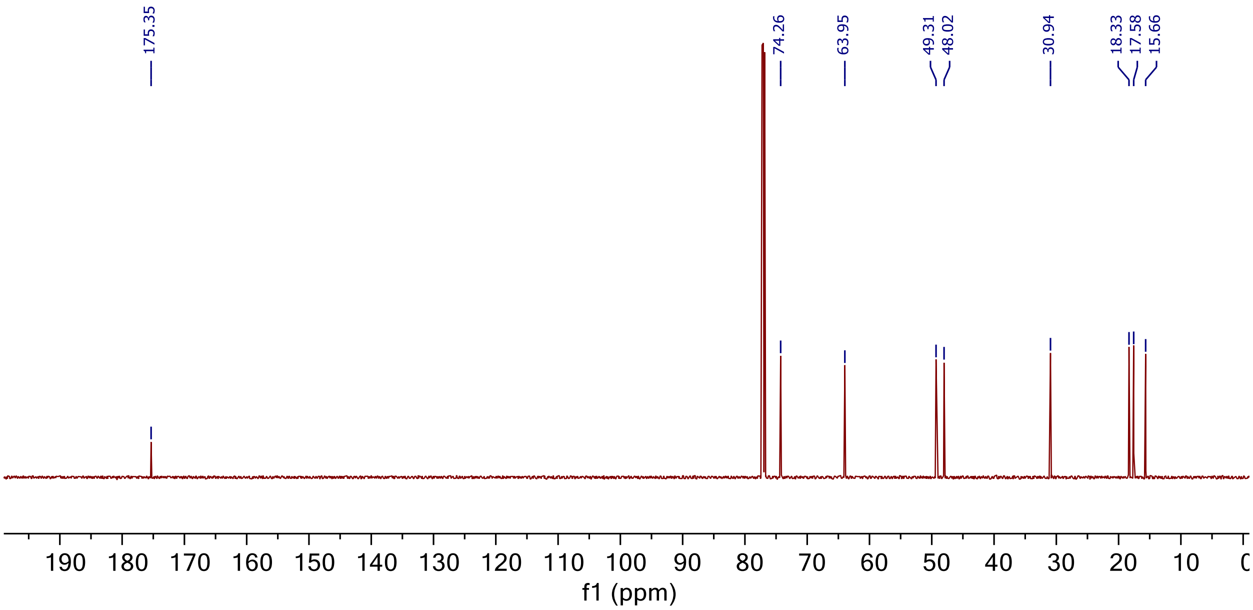

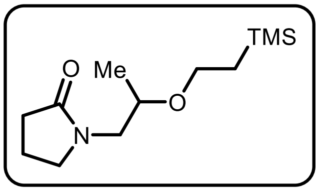

1-(2-(2-(trimethylsilyl)ethoxy)propyl)pyrrolidin-2-one (23b).

^{1}H NMR (600 MHz, $CDCl_3$):

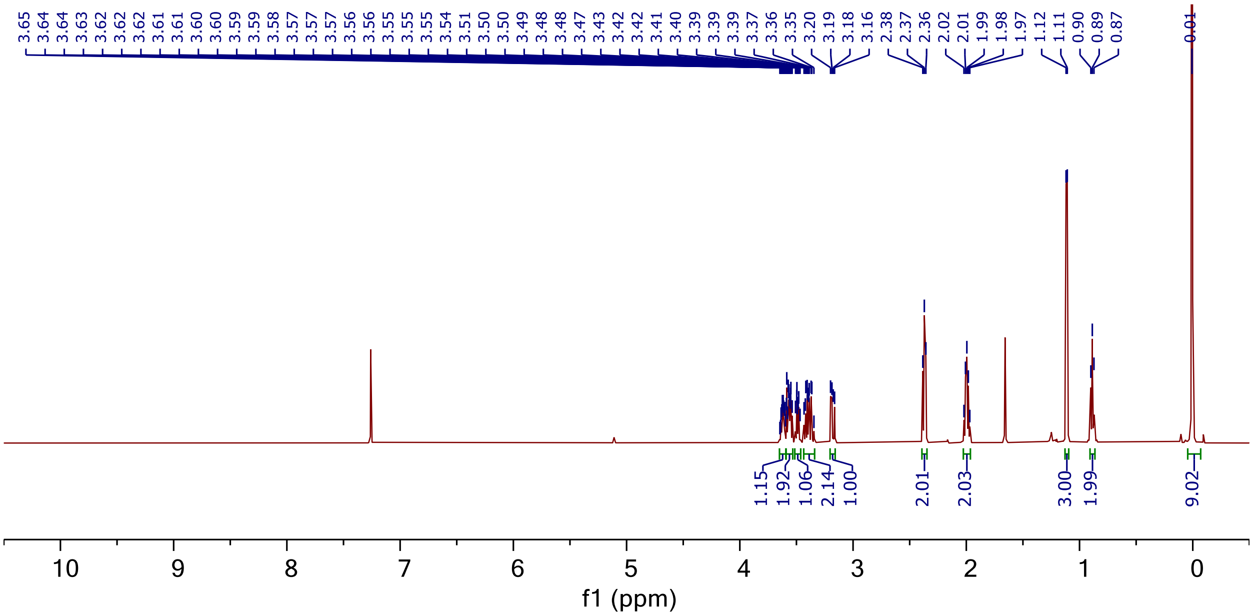

^{13}C NMR (151 MHz, $CDCl_3$):

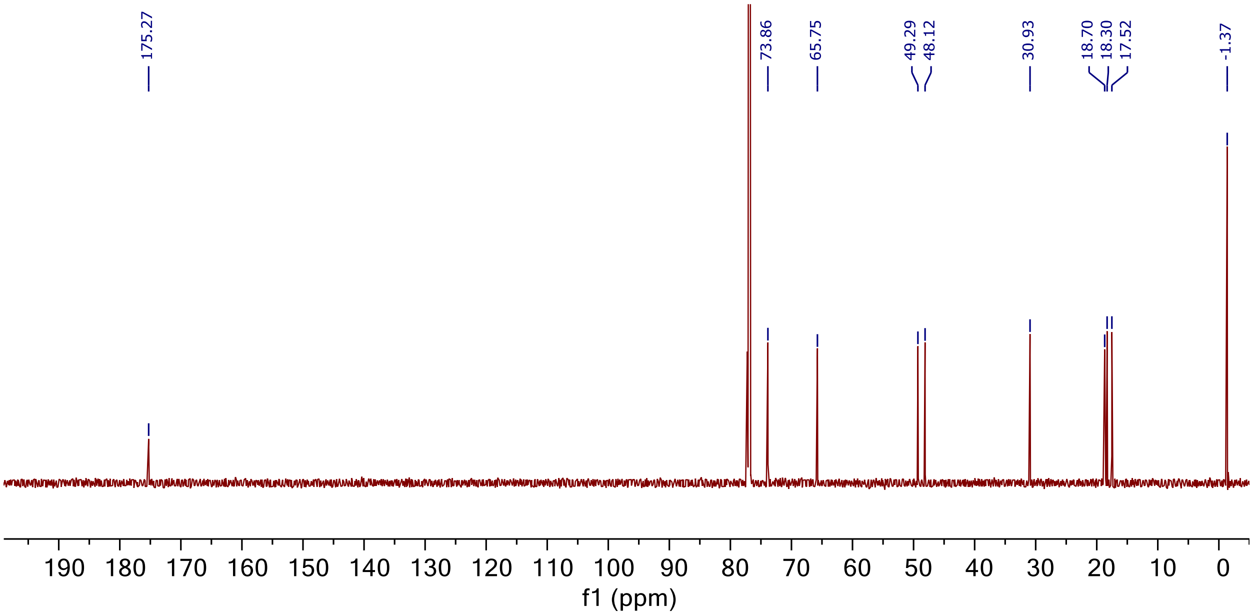

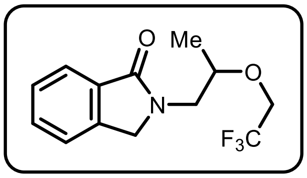

2-(2-(3,3,3-trifluoropropoxy)propyl)isoindolin-1-one (24b).

^{1}H NMR (600 MHz, CDCl3):

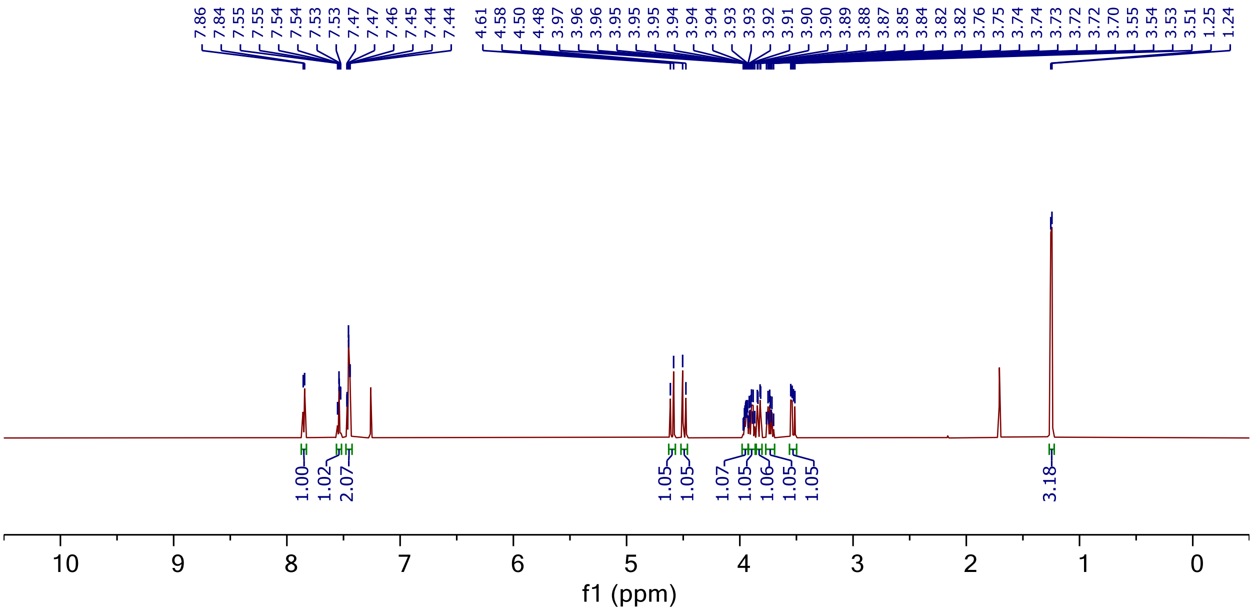

^{13}C NMR (151 MHz, CDCl3):

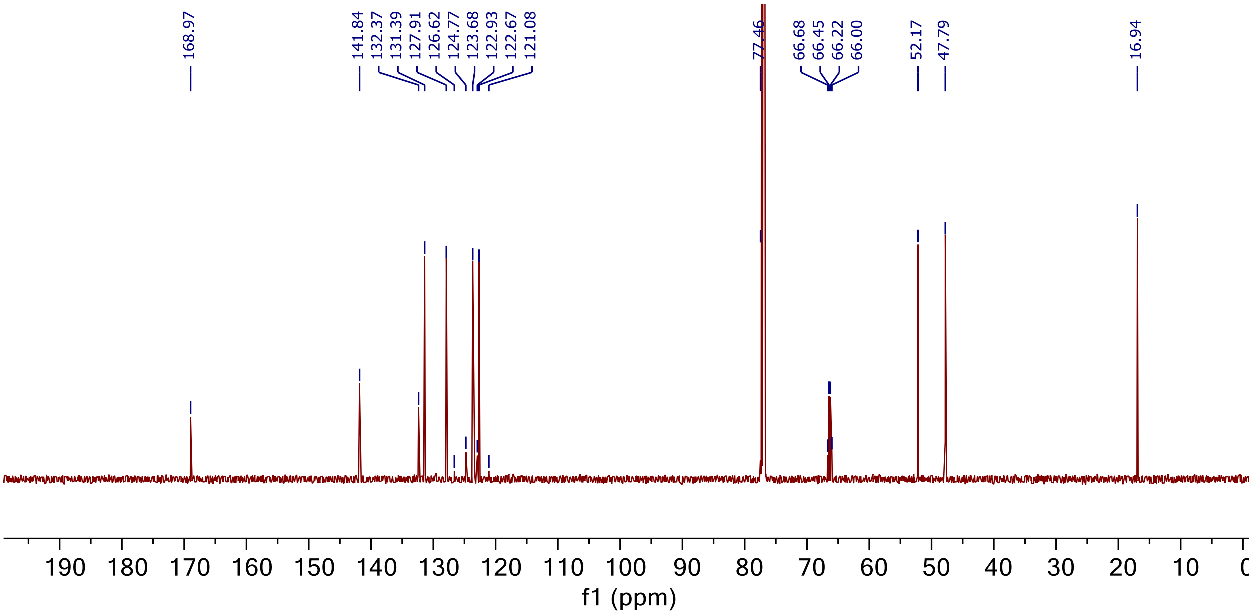

^{19}F NMR (471 MHz, $CDCl_3$)

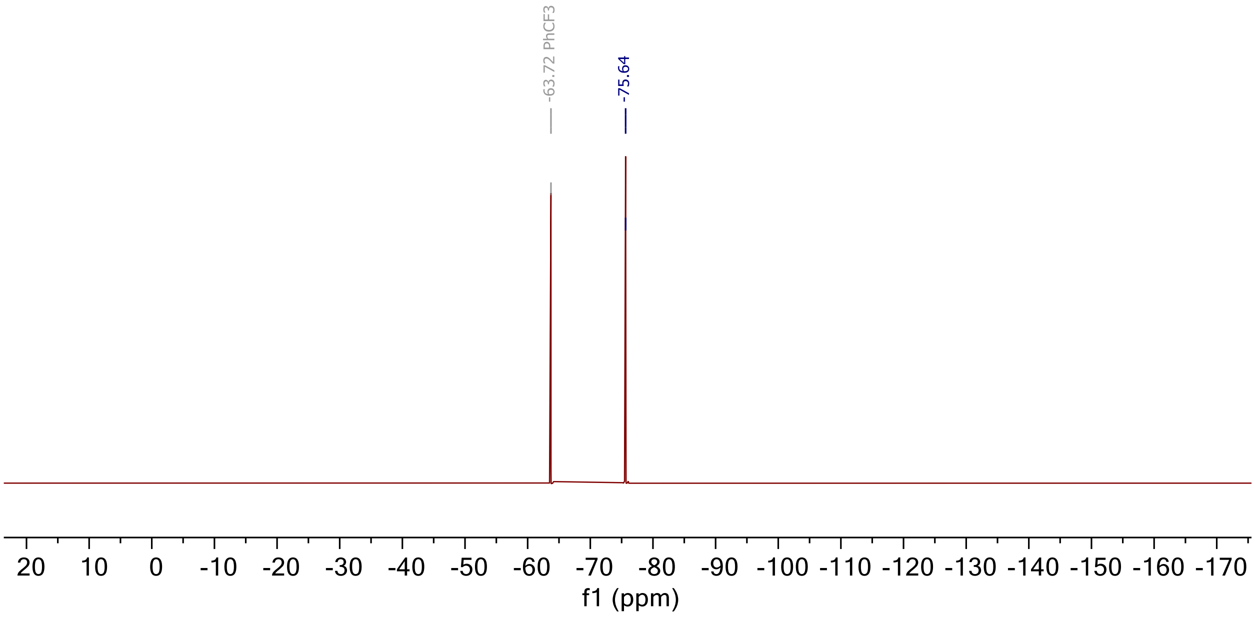

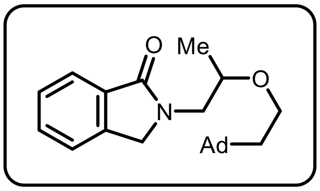

2-(2-(2-((3R,5R,7R)-adamantan-1-yl)ethoxy)propyl)isoindolin-1-one (25b).

¹H NMR (600 MHz, $CDCl_3$):

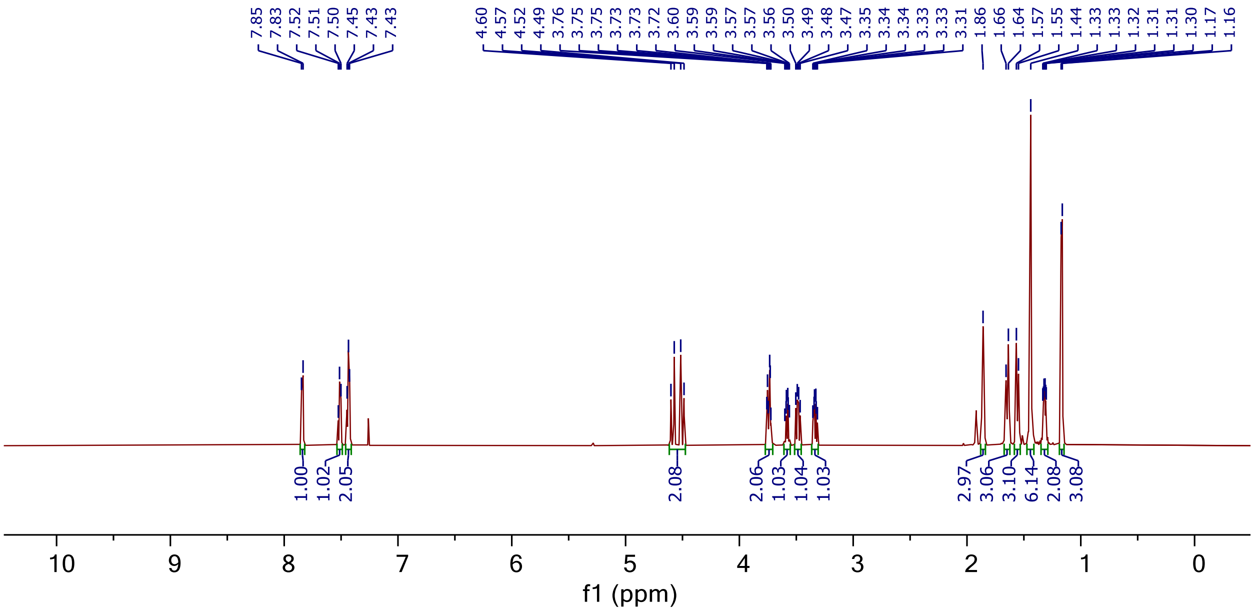

¹³C NMR (151 MHz, $CDCl_3$):

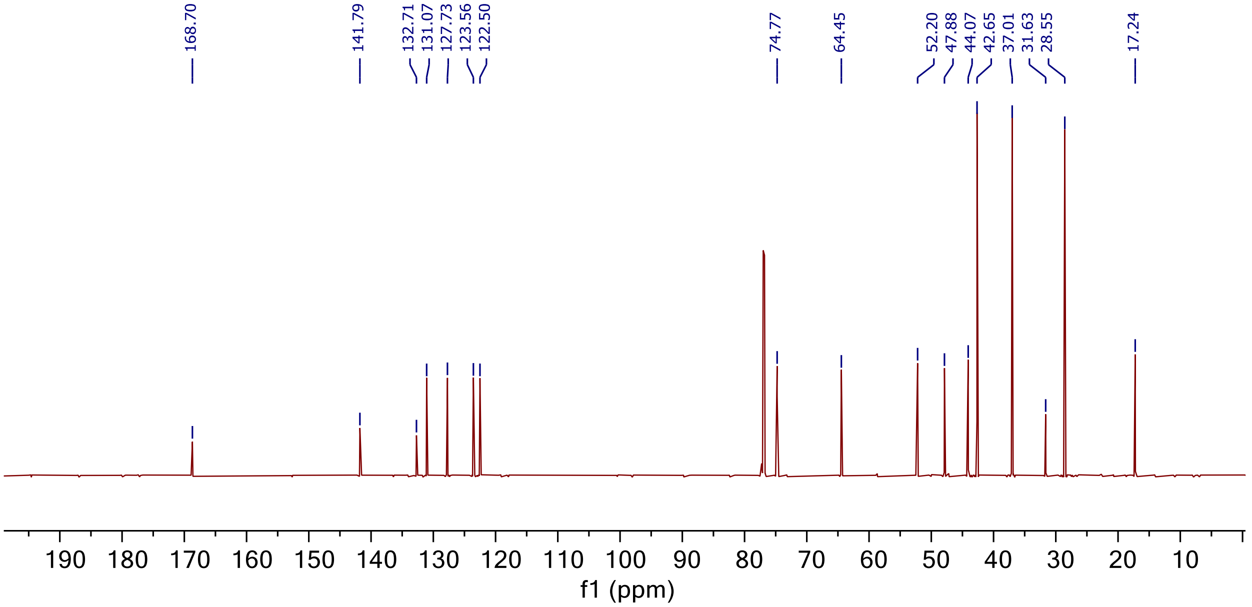

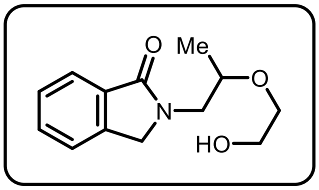

2-(2-(2-hydroxyethoxy)propyl)isoindolin-1-one (26b).

^{1}H NMR (600 MHz, $CDCl_3$):

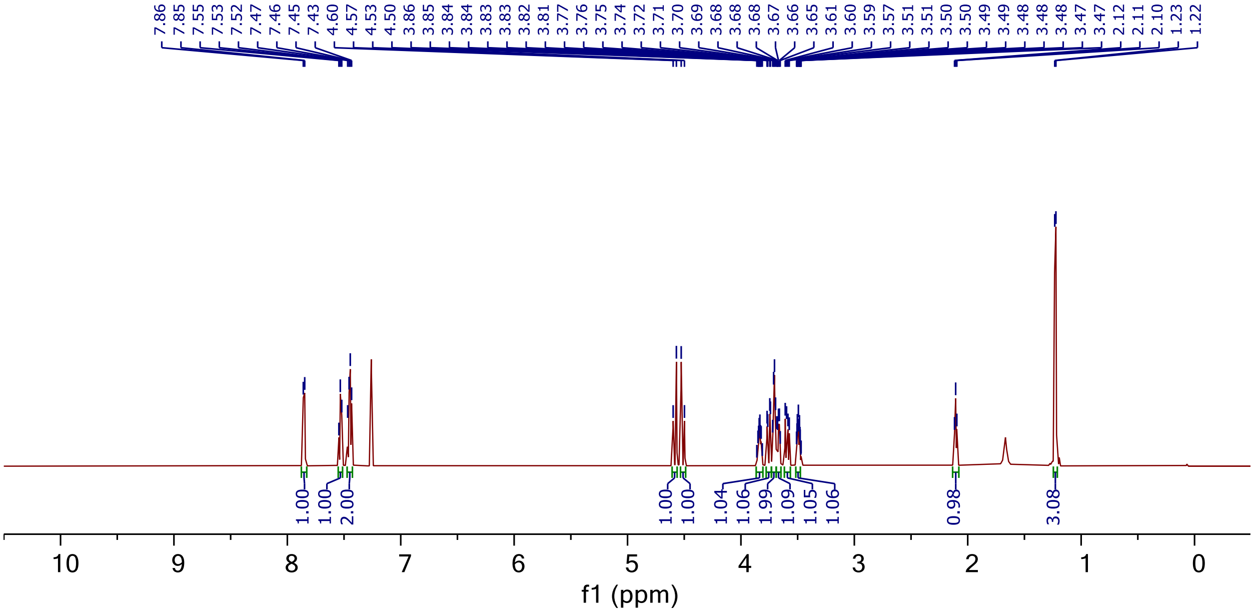

^{13}C NMR (151 MHz, $CDCl_3$):

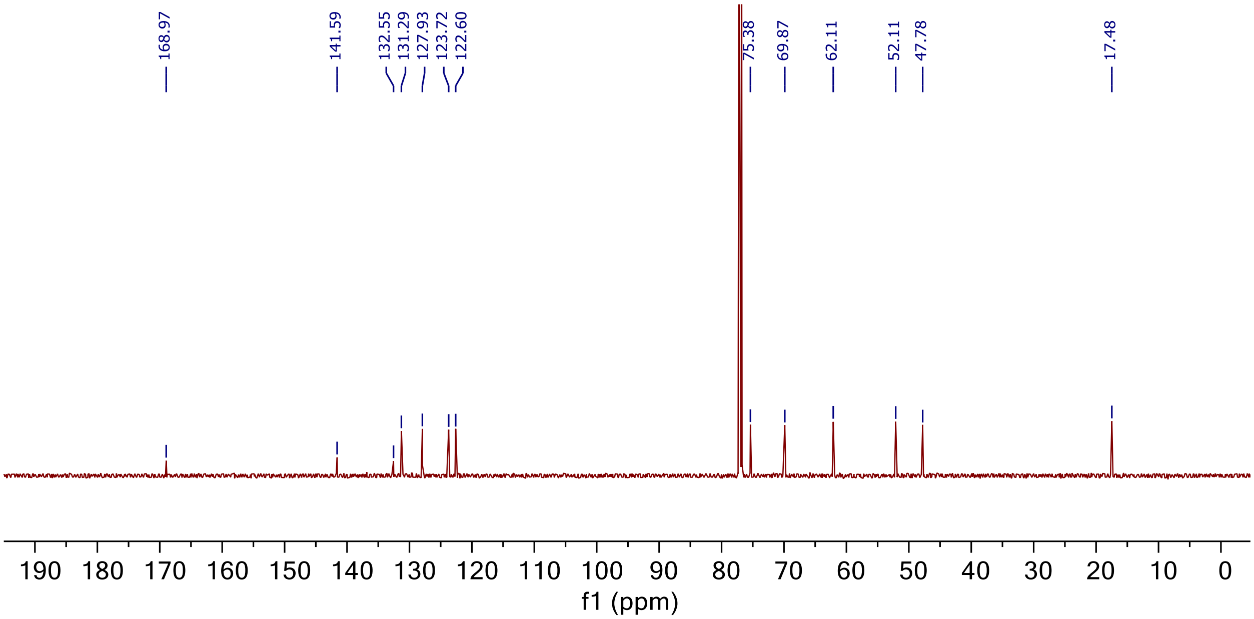

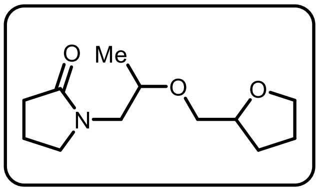

1-(2-((tetrahydrofuran-2-yl)methoxy)propyl)pyrrolidin-2-one (27b).

^{1}H NMR (600 MHz, $CDCl_3$):

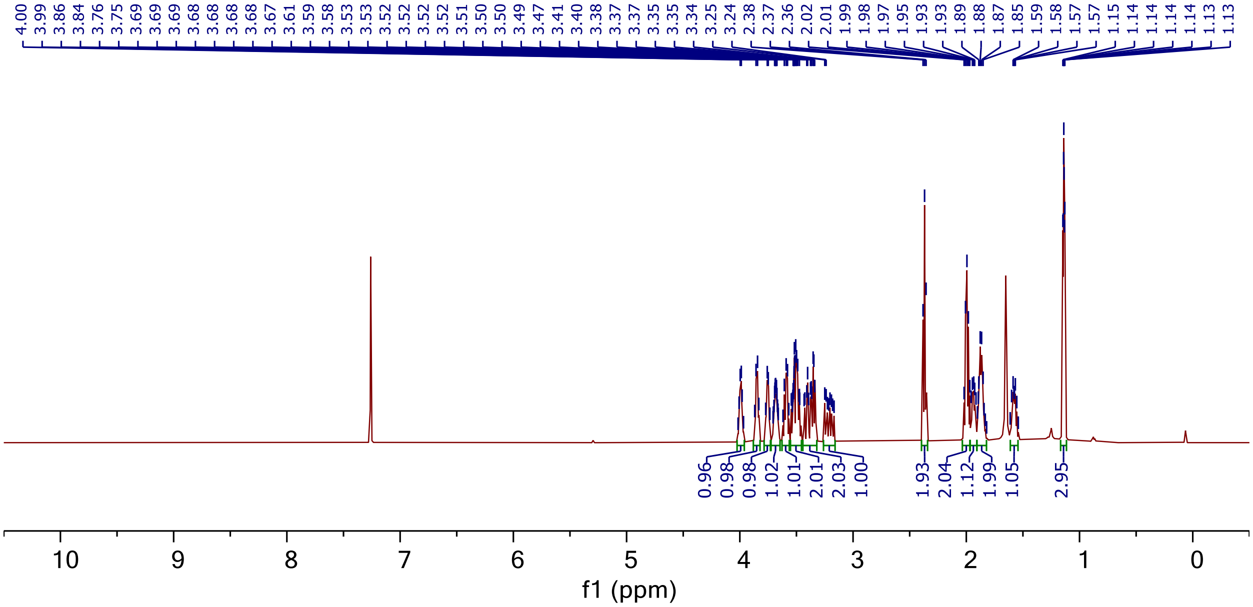

^{13}C NMR (151 MHz, $CDCl_3$):

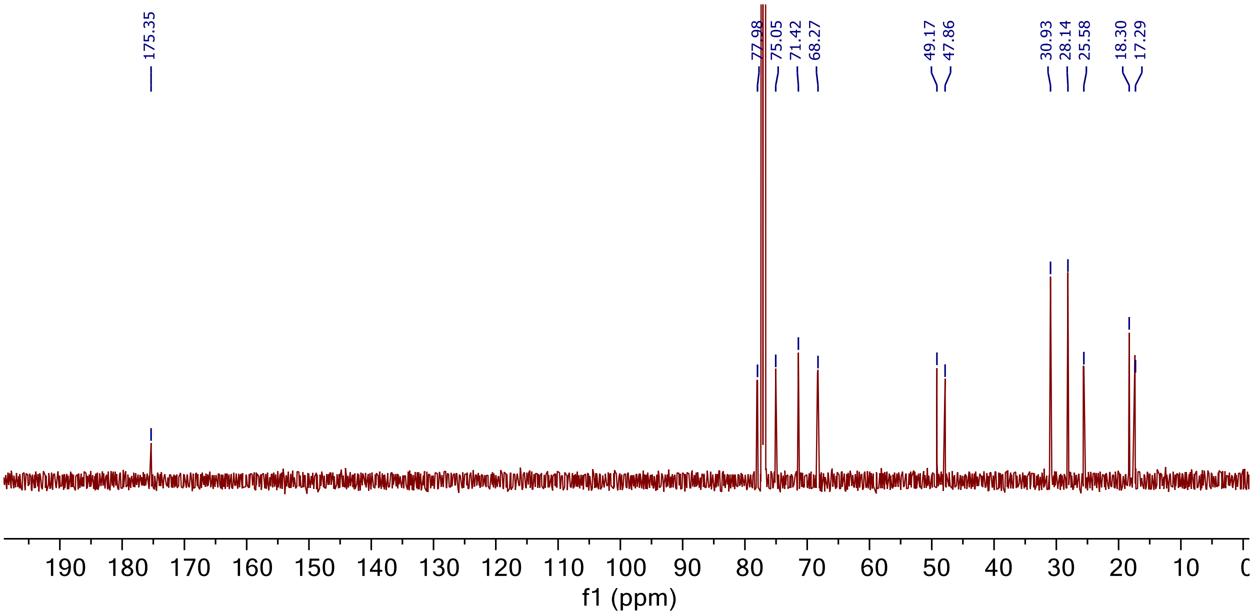

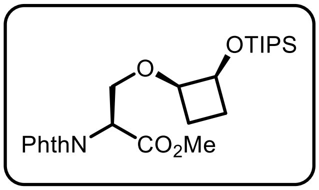

cis-methyl2-(1,3-dioxoisoindolin-2-yl)-3-(2-((triisopropylsilyl)oxy)cyclobutoxy)propanoate (28b).

^{1}H NMR (600 MHz, $CDCl_3$):

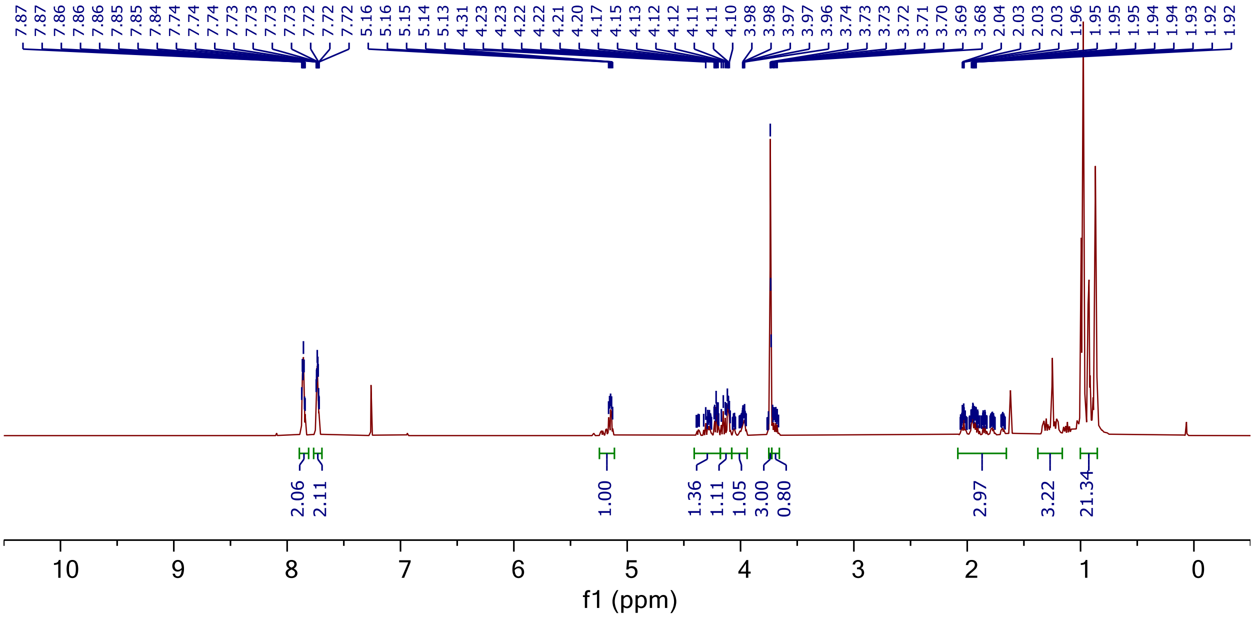

^{13}C NMR (151 MHz, $CDCl_3$):

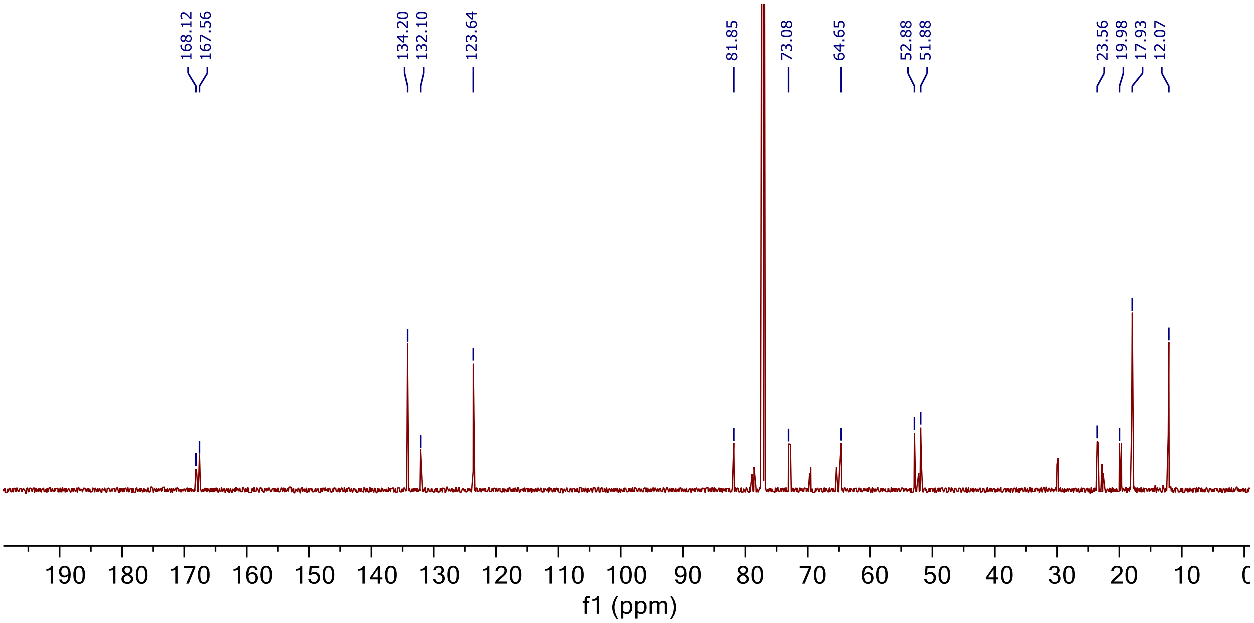

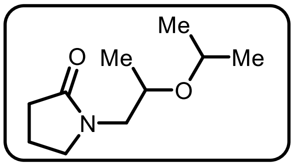

<u>1-(2-isopropoxypropyl)pyrrolidin-2-one (29b)</u>.

^{1}H NMR (600 MHz, $CDCl_3$):

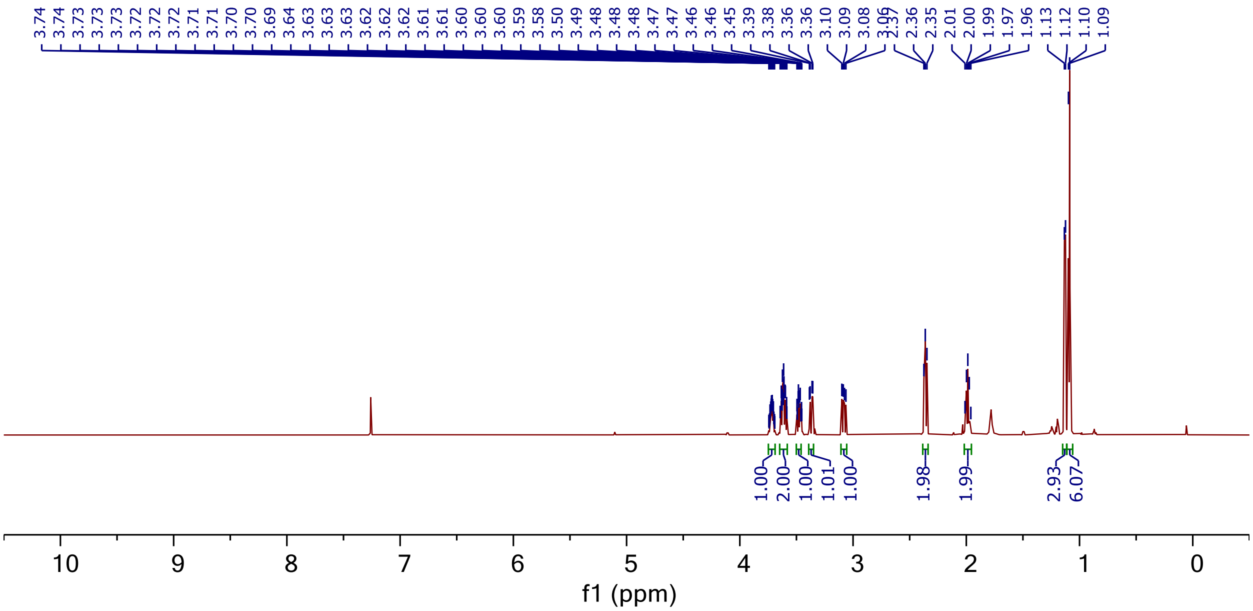

^{13}C NMR (151 MHz, $CDCl_3$):

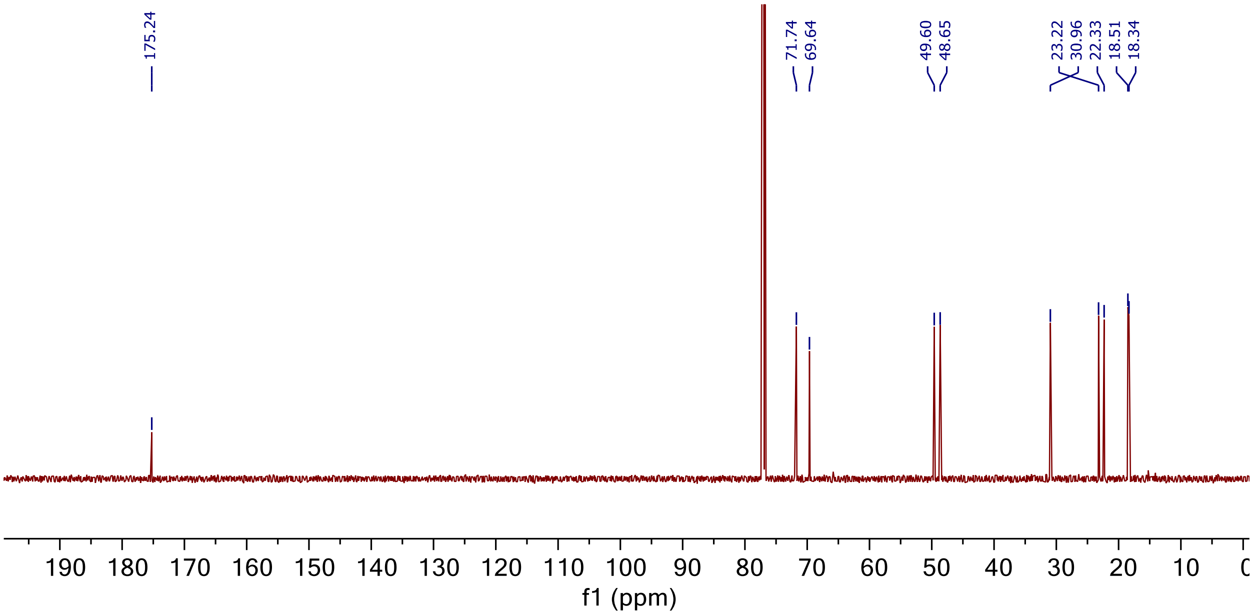

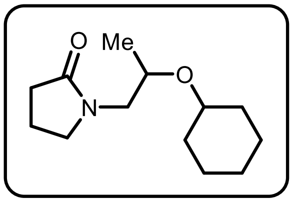

1-(2-isopropoxypropyl)pyrrolidin-2-one (30b).

^{1}H NMR (600 MHz, $CDCl_3$):

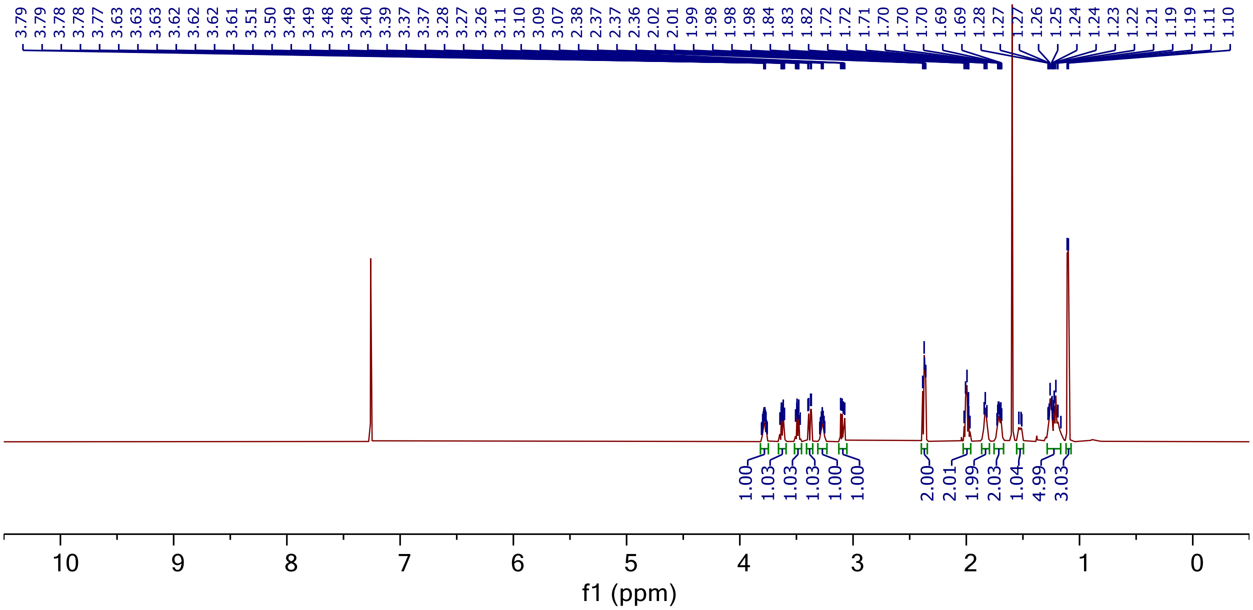

^{13}C NMR (151 MHz, $CDCl_3$):

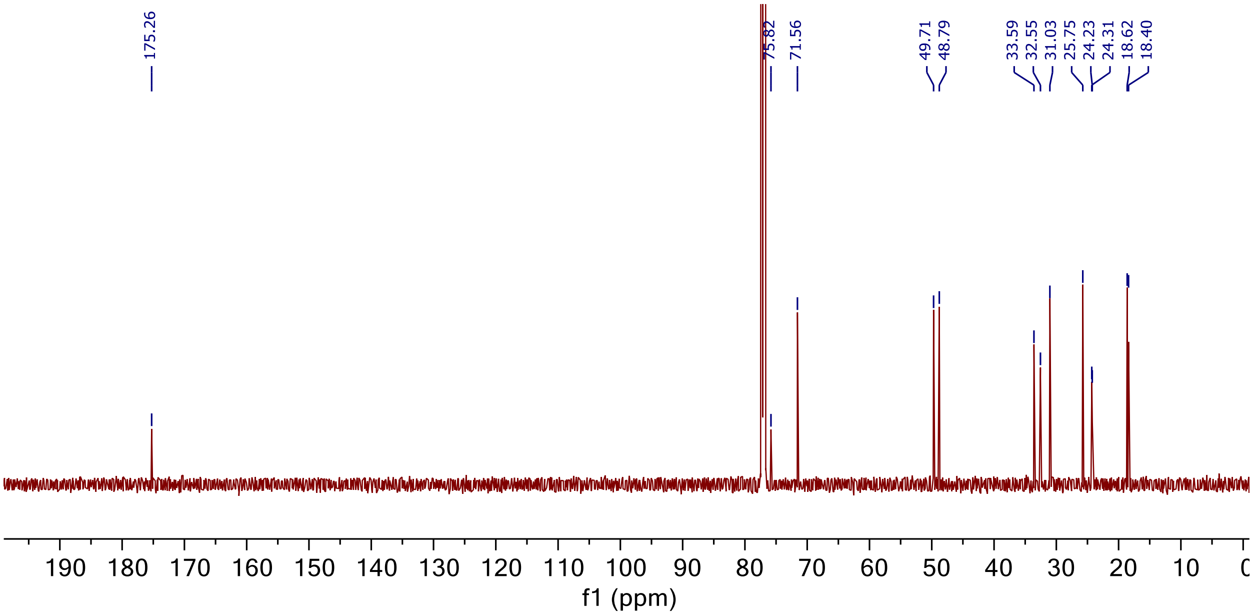

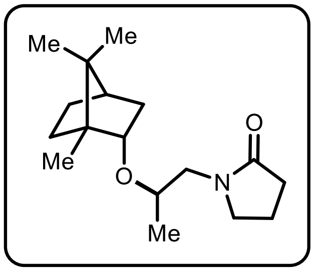

1-(2-(((1S,2S,4R)-1,7,7-trimethylbicyclo[2.2.1]heptan-2-yl)oxy)propyl)pyrrolidin-2-one (31b).

^{1}H NMR (600 MHz, $CDCl_3$):

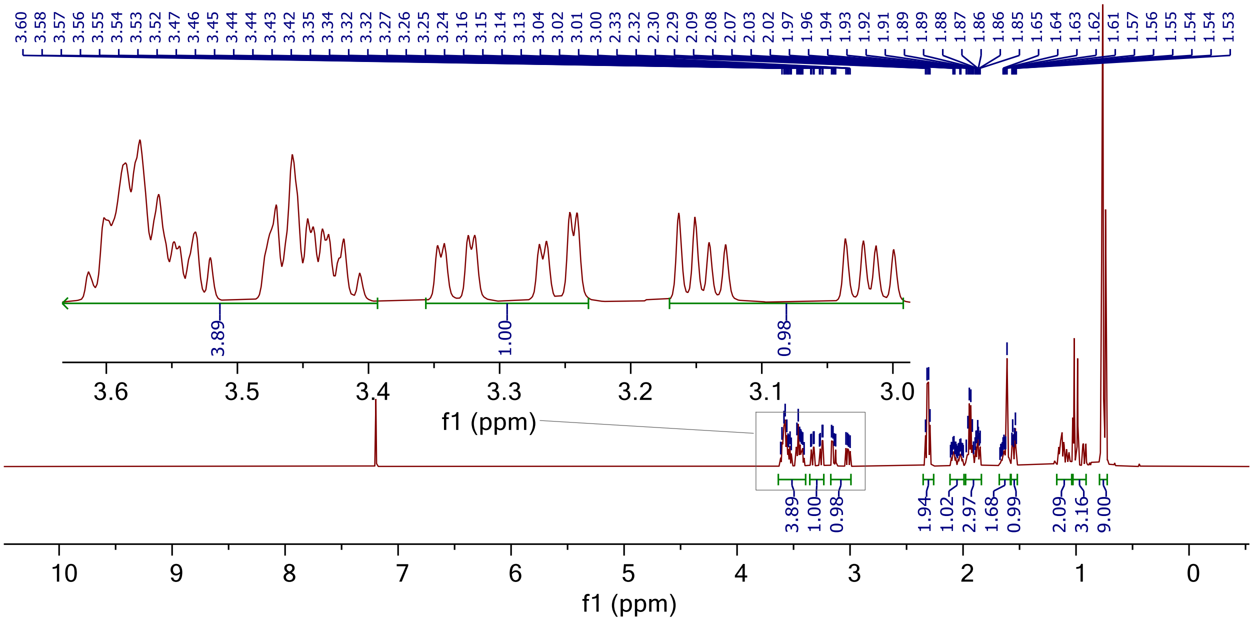

^{13}C NMR (151 MHz, $CDCl_3$):

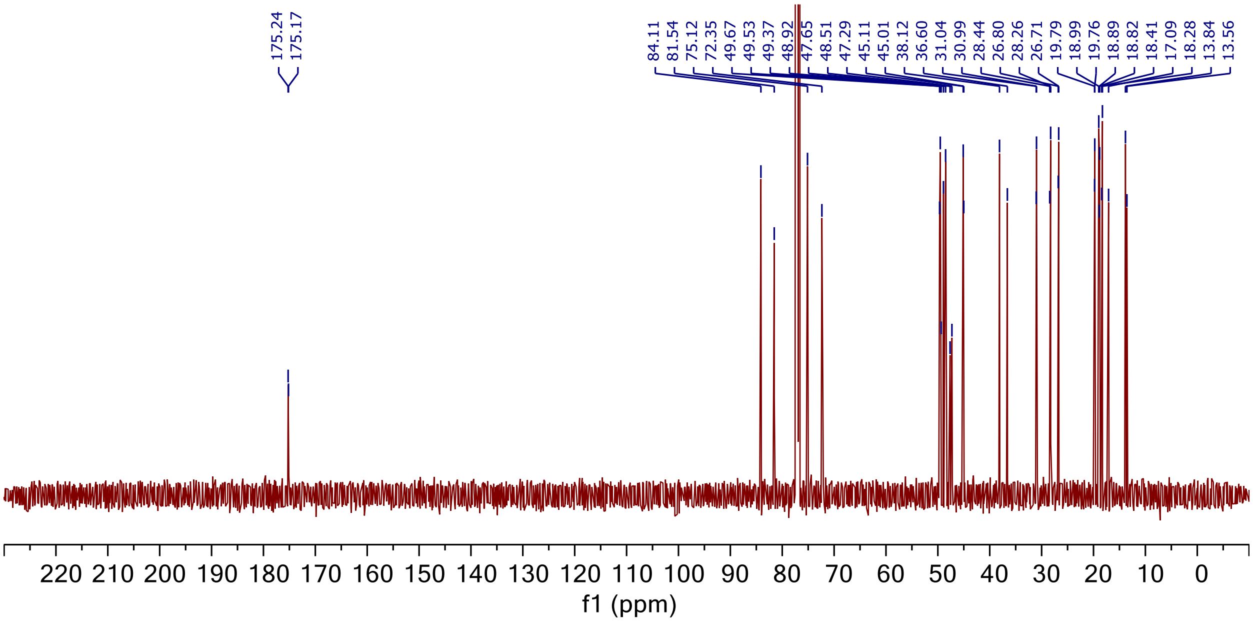

trans-triisopropyl((1S,2S)-2-(((1S,2S,4R)-1,7,7-trimethylbicyclo[2.2.1]heptan-2-yl)oxy)cyclobutoxy)silane (32b).

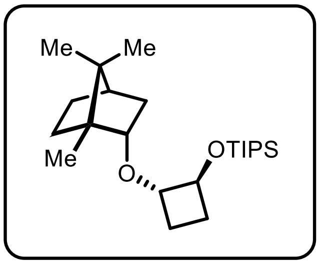

^{1}H NMR (600 MHz, $CDCl_3$):

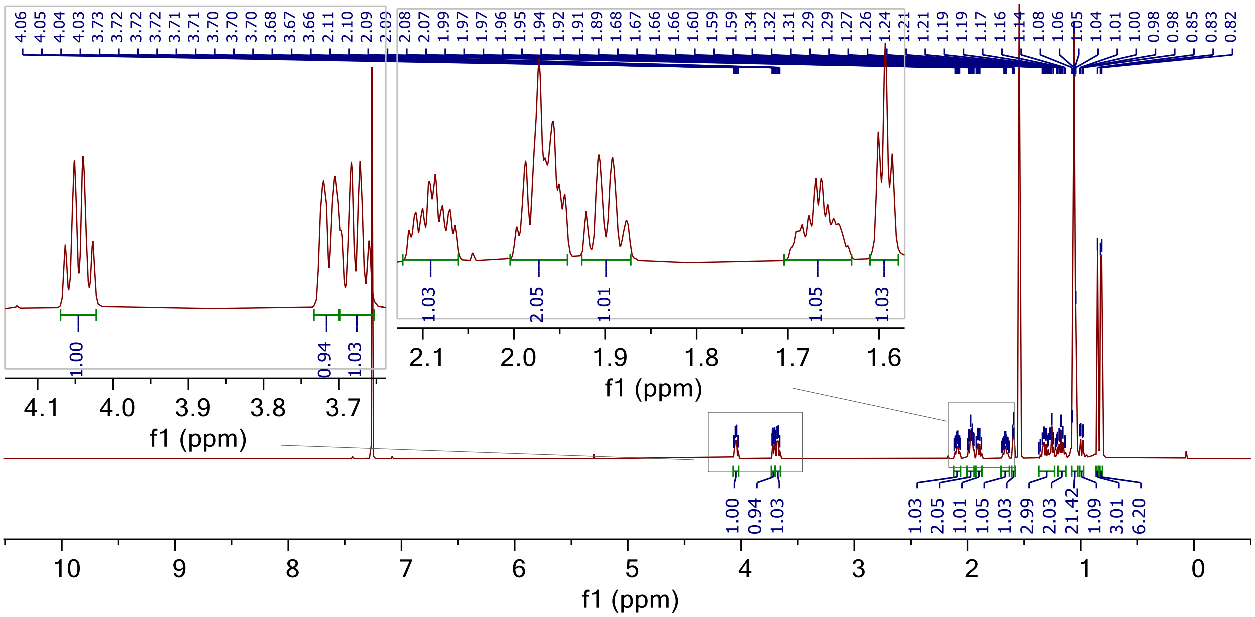

^{13}C NMR (151 MHz, $CDCl_3$):

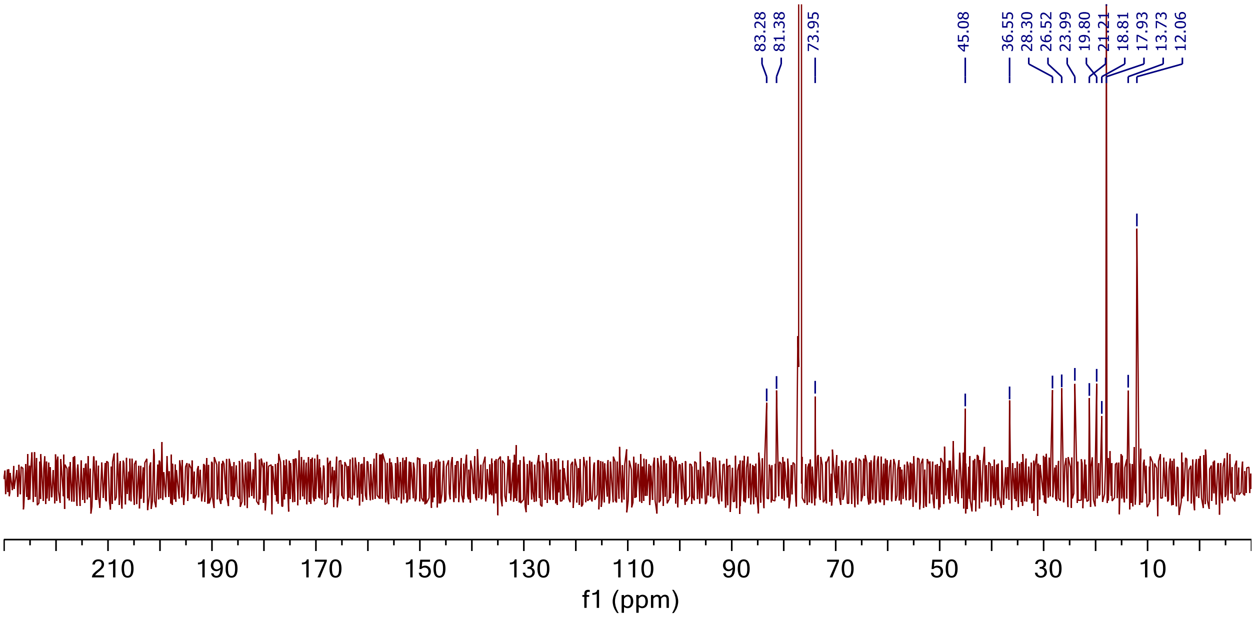

NOESY (600 MHz, $CDCl_3$):

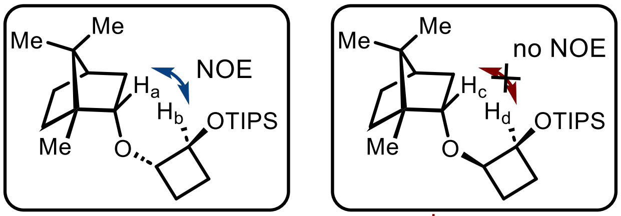

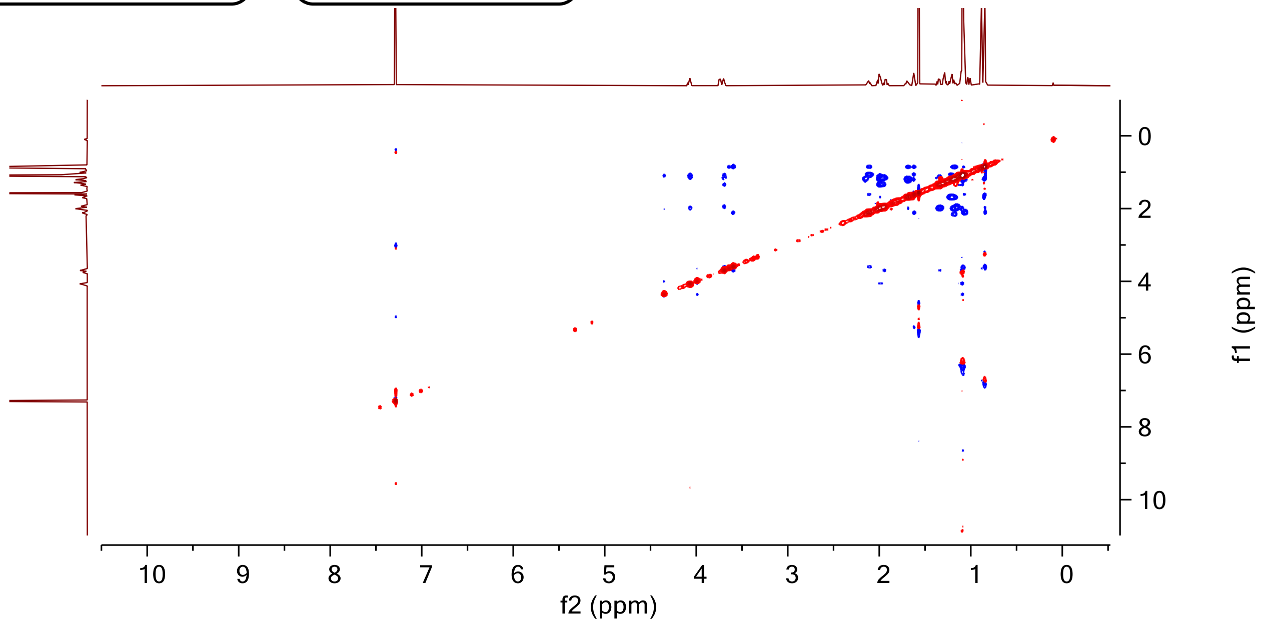

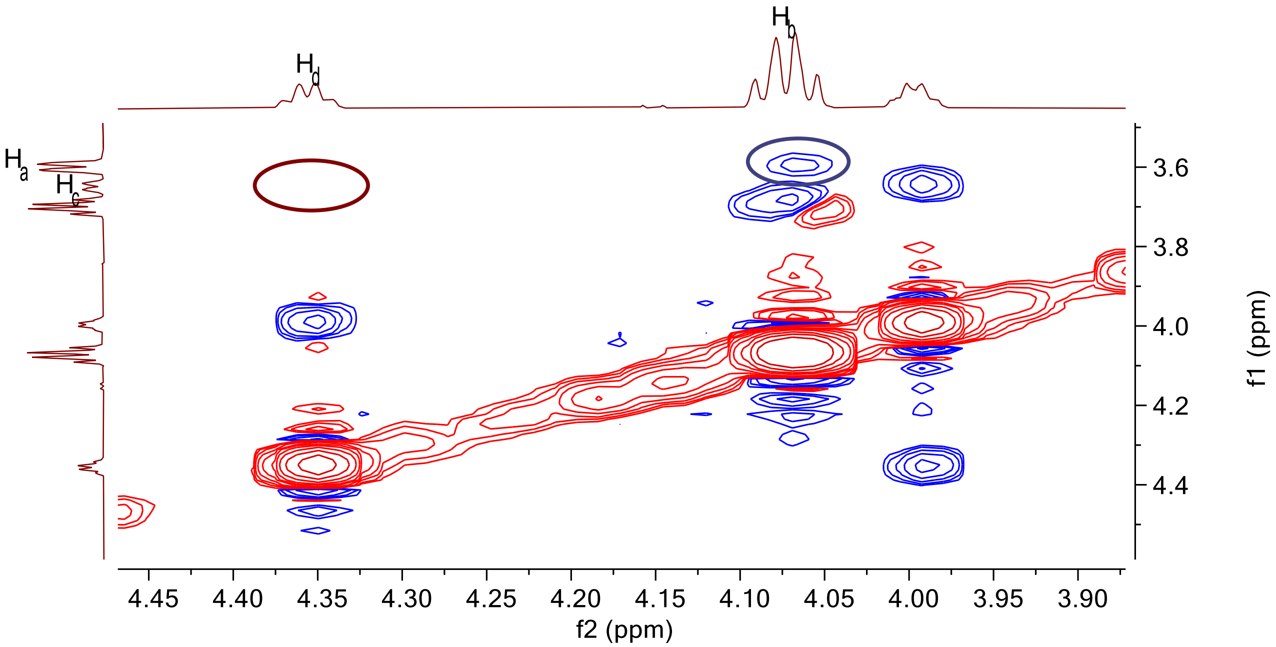

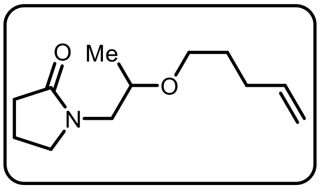

1-(2-(pent-4-en-1-yloxy)propyl)pyrrolidin-2-one (33b).

^{1}H NMR (600 MHz, $CDCl_3$):

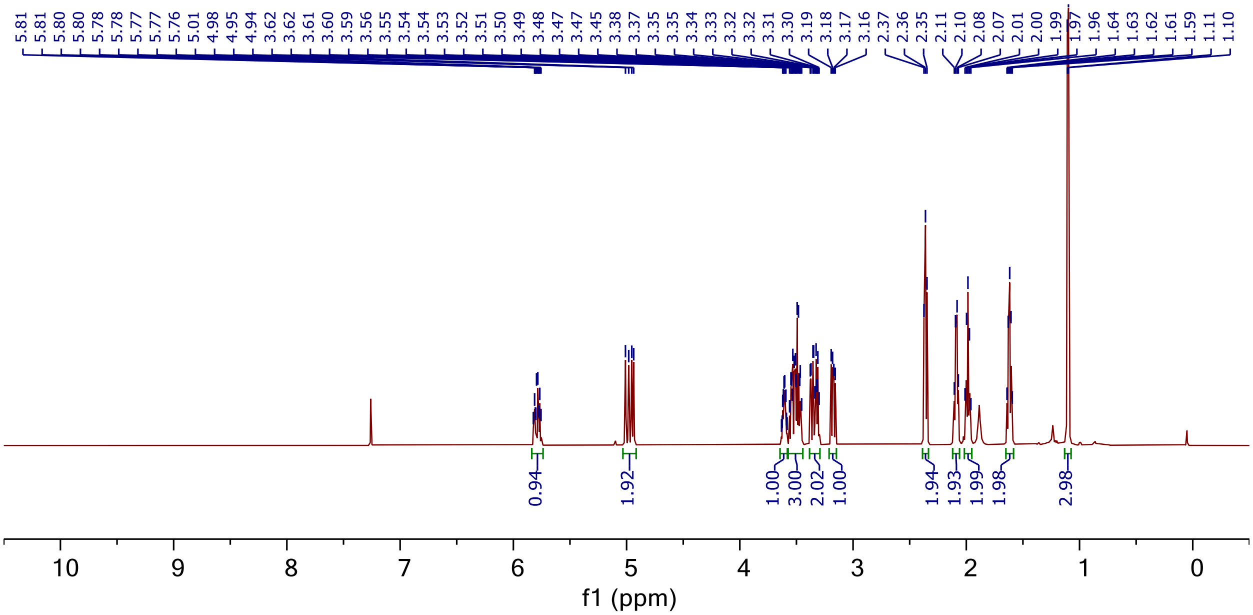

^{13}C NMR (151 MHz, $CDCl_3$):

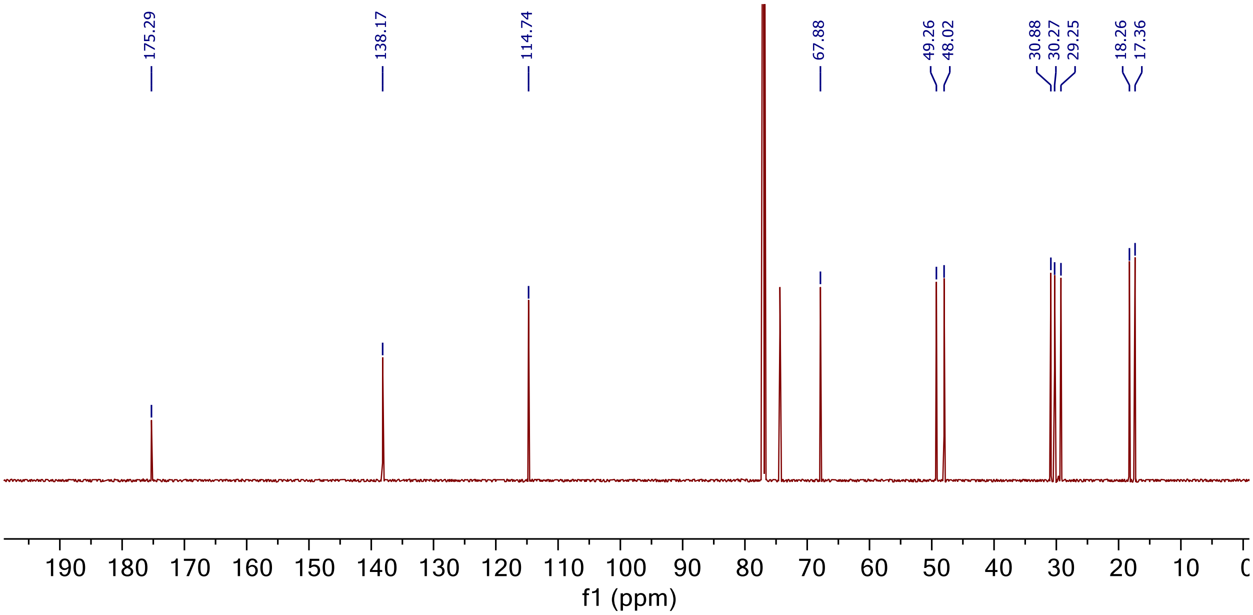

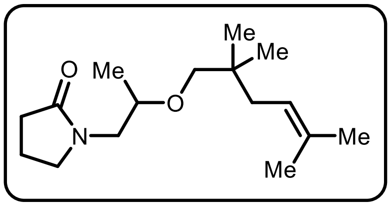

1-(2-((2,2,5-trimethylhex-4-en-1-yl)oxy)propyl)pyrrolidin-2-one (34b).

^{1}*H NMR* (600 MHz, $CDCl_3$):

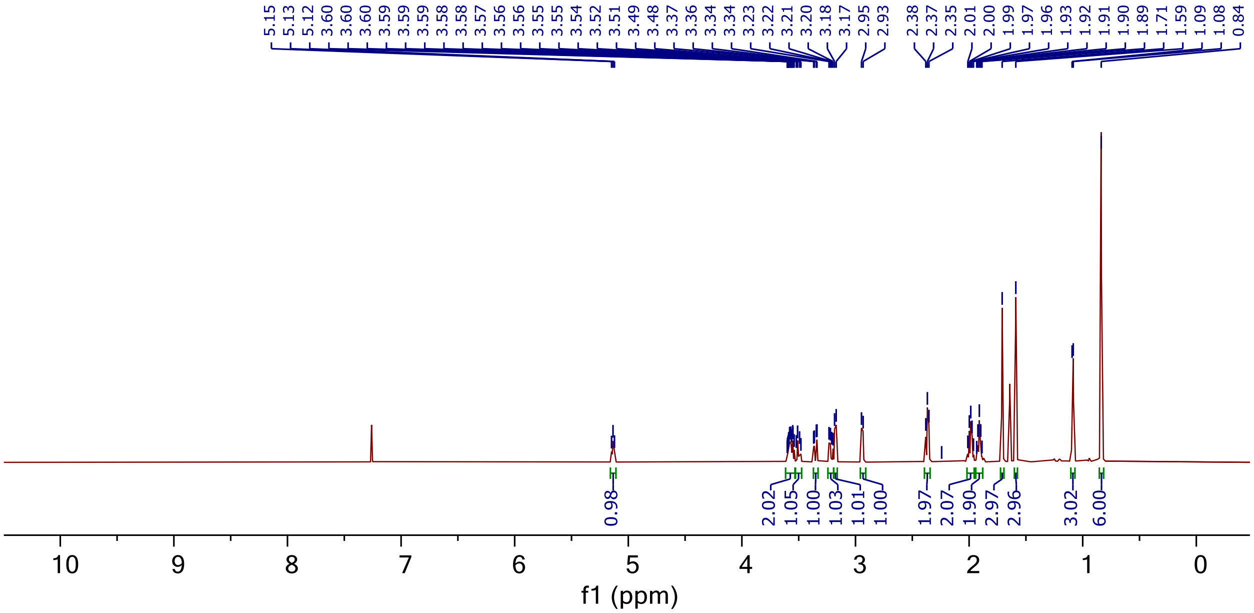

^{13}*C NMR* (151 MHz, $CDCl_3$):

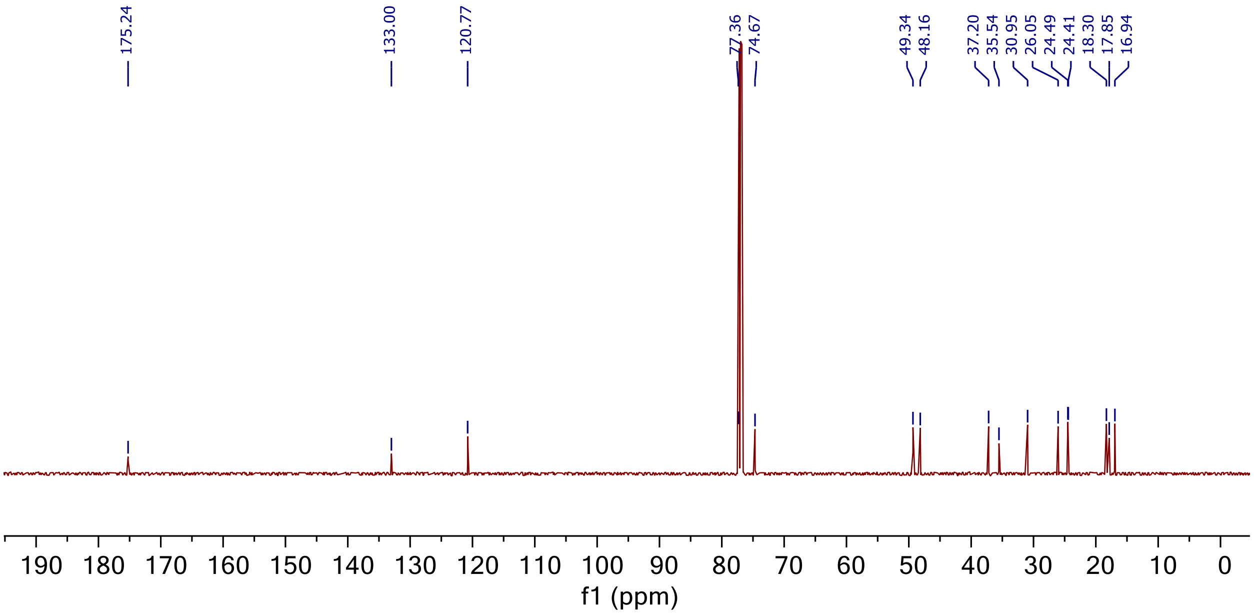

I. References

(1) Zhang, Z.; Smal, V.; Retailleau, P.; Voituriez, A.; Frison, G.; Marinetti, A.; Guinchard, X. Tethered Counterion-Directed Catalysis: Merging the Chiral Ion-Pairing and Bifunctional Ligand Strategies in Enantioselective Gold(I) Catalysis. *J. Am. Chem. Soc.* **2020**, *142* (8), 3797–3805.

(2) Kawasaki, M.; Shimizu, M.; Kuroyanagi, S.; Shishido, Y.; Komiyama, T.; Toyooka, N. Syntheses and Odor Properties of Optically Active Dimethyl Octenone and Its Analogs. *Tetrahedron: Asymmetry* **2016**, *27* (6), 285–293.

(3) Arai, M. A.; Kuraishi, M.; Arai, T.; Sasai, H. A New Asymmetric Wacker-Type Cyclization and Tandem Cyclization Promoted by Pd(II)-Spiro Bis(Isoxazoline) Catalyst. *J. Am. Chem. Soc.* **2001**, *123* (12), 2907–2908.

(4) Yu, Z.-L.; Cheng, Y.-F.; Liu, J.-R.; Yang, W.; Xu, D.-T.; Tian, Y.; Bian, J.-Q.; Li, Z.-L.; Fan, L.-W.; Luan, C.; Gao, A.; Gu, Q.-S.; Liu, X.-Y. Cu(I)-Catalyzed Chemo- and Enantioselective Desymmetrizing C–O Bond Coupling of Acyl Radicals. *J. Am. Chem. Soc.* **2023**, *145* (11), 6535–6545.

(5) Hamilton, D. S.; Nicewicz, D. A. Direct Catalytic Anti-Markovnikov Hydroetherification of Alkenols. *J. Am. Chem. Soc.* **2012**, *134* (45), 18577–18580.

(6) Michael, J.; Nkwelo, M.Heterocyclisations Induced by Thallium(III) Acetate. Effect of Varying the Internal Nucleophile. *Tetrahedron* **1990**, *46* (7), 2549–2560.

(7) Mohammadlou, A.; Joshi, C.; Smith, B. P.; Zheng, L.; Corio, S. A.; Canestraight, V. M.; Kohlbouni, S. T.; Taimoory, S. M.; Borhan, B.; Staples, R.; Vetticatt, M. J.; Wulff, W. D. A Lewis Acid-Controlled Enantiodivergent Epoxidation of Aldehydes. *ACS Catal.* **2023**, *13* (19), 13117–13126.

(8) Wang, W.; He, G. Halogenation-Enabled Intramolecular Deaminative Cyclization. *Org. Biomol. Chem.* **2025**, *23* (23), 5559–5563.

(9) Morita, N.; Yamashita, D.; Hashimoto, Y.; Tamura, O.; Morita, N.; Yamashita, D.; Hashimoto, Y.; Tamura, O. An Efficient Stereoselective Synthesis of Cis-2,6-Disubstituted Tetrahydropyrans via Gold-Catalyzed Meyer–Schuster Rearrangement/Hydration/Oxa-Michael Addition Sequence. *Catalysts* **2024**, *14* (4), 228.

(10) Wang, P.; Blank, D. H.; Spencer, T. A. Synthesis of Benzophenone-Containing Analogues of Phosphatidylcholine. *J. Org. Chem.* **2004**, *69* (8), 2693–2702.

(11) Li, P.; Duan, L.; Lin, Y.; Chu, L.; Zuo, Z. Modulating Electron Transfer via Cerium Photocatalysis for Alkoxy Radical-Mediated Selective Hydroetherification. *Angew. Chem. Int. Ed.* **2025**, *64* (26), e202501949.

(12) Xie, F.; Chen, Y.; Li, Y.; Wang, Z.; Zhang, J.; Tang, Y. Photo-Induced Two-Carbon Ring Expansion of N-Alkenyl Lactams and N-Alkenyl/Phenyl Benzoazetinones. *Org. Chem. Front.* **2023**, *10* (4), 928–935.

(13) Xu, H.-Y.; Zi, Y.; Xu, X.-P.; Wang, S.-Y.; Ji, S.-J. TFA-Catalyzed C–N Bond Activation of Enamides with Indoles: Efficient Synthesis of 3,3-Bisindolylpropanoates and Other Bisindolylalkanes. *Tetrahedron* **2013**, *69* (5), 1600–1605.

(14) Cadge, J. A.; Sparkes, H. A.; Bower, J. F.; Russell, C. A. Oxidative Addition of Alkenyl and Alkynyl Iodides to a AuI Complex. *Angew. Chem. Int. Ed.* **2020**, *59* (16), 6617–6621.

(15) Cai, H.; Tu, Y.-Q.; Niu, Q.; Xie, W.-P.; Wang, B.; Lu, K.; Li, Z.-H.; Zhang, F.-M.; Zhang, X.-M. CCC Pincer Ru Complex-Catalyzed C–H Vinylation/6π-E-Cyclization of Aldimines for Constructing 4H-Pyrido[1,2-a]Pyrimidines. *Chem. Sci.* **2024**, *15* (39), 16216–16221.

(16) Zhan, F.; Liang, G. Formation of Enehydrazine Intermediates through Coupling of Phenylhydrazines with Vinyl Halides: Entry into the Fischer Indole Synthesis. *Angew. Chem. Int. Ed.* **2013**, *52* (4), 1266–1269.

(17) Armstrong, R. J.; García-Ruiz, C.; Myers, E. L.; Aggarwal, V. K. Stereodivergent Olefination of Enantioenriched Boronic Esters. *Angew. Chem. Int. Ed.* **2017**, *56* (3), 786–790.

(18) He, Y.; Song, H.; Chen, J.; Zhu, S. NiH-Catalyzed Asymmetric Hydroarylation of N-Acyl Enamines to Chiral Benzylamines. *Nat Commun.* **2021**, *12* (1), 638.

(19) Hofstra, J. L.; Poremba, K. E.; Shimozono, A. M.; Reisman, S. E. Nickel-Catalyzed Conversion of Enol Triflates into Alkenyl Halides. *Angew. Chem. Int. Ed.* **2019**, *58* (42), 14901–14905.

(20) Miguélez, R.; Semleit, N.; Rodríguez-Arias, C.; Mykhailiuk, P.; González, J. M.; Haberhauer, G.; Barrio, P. C−H Activation of Unbiased C(Sp3)−H Bonds: Gold(I)-Catalyzed Cycloisomerization of 1-Bromoalkynes. *Angew. Chem. Int. Ed.* **2023**, *62* (26), e202305296.

(21) Zlotorzynska, M.; Zhai, H.; Sammis, G. M. Chemoselective Oxygen-Centered Radical Cyclizations onto Silyl Enol Ethers. *Org. Lett.* **2008**, *10* (21), 5083–5086.

(22) Dubowchik, G. M.; Vrudhula, V. M.; Dasgupta, B.; Ditta, J.; Chen, T.; Sheriff, S.; Sipman, K.; Witmer, M.; Tredup, J.; Vyas, D. M.; Verdoorn, T. A.; Bollini, S.; Vinitsky, A. 2-Aryl-2,2-Difluoroacetamide FKBP12 Ligands: Synthesis and X-Ray Structural Studies. *Org. Lett.* **2001**, *3* (25), 3987–3990.

(23) Wu, F.; Guo, Y.; Ren, Z.; Chen, Z.; Liu, X.; Wang, C.; Rong, L. Electrochemical Radical Reactions of Enol Acetates and Free Alcohols Directly Access to α-Alkoxylated Carbonyl Compounds. *J. Org. Chem.* **2023**, *88* (13), 8825–8834.

(24) Mondal, R.; Jacob, N.; Devuyst, M.; Quertenmont, M.; Averochkin, G.; Deri, S.; Galmidi, L.; Gordon-Levitan, D.; Feller, M.; Vantourout, J. C.; Echeverria, P.-G.; Gnaim, S. Electro-Oxidative Platform for Nucleophilic α-Functionalization of Ketones. *Synthesis* **2025**, *57* (13), 2124–2130.

(25) Caravana, A. C.; Nagasing, B.; Dhanju, S.; Reynolds, R. G.; Weiss, E. A.; Thomson, R. J. Electrochemical and Photocatalytic Oxidative Coupling of Ketones via Silyl Bis-Enol Ethers. *J. Org. Chem.* **2021**, *86* (9), 6600–6611.
(26) Tsui, E.; Metrano, A. J.; Tsuchiya, Y.; Knowles, R. R. Catalytic Hydroetherification of Unactivated Alkenes Enabled by Proton-Coupled Electron Transfer. *Angew. Chem. Int. Ed.* **2020**, *59* (29), 11845–11849.
(27) Tu, W.; Farndon, J. J.; Robertson, C. M.; Bower, J. F. An Aza-Prilezhaev-Based Method for Inversion of Regioselectivity in Stereospecific Alkene 1,2-Aminohydroxylations. *Angew. Chem. Int. Ed.* **2024**, *63* (49), e202409836.
